# DNA methylation patterns reflect individual's lifestyle independent of obesity

**DOI:** 10.1002/ctm2.851

**Published:** 2022-06-12

**Authors:** Ireen Klemp, Anne Hoffmann, Luise Müller, Tobias Hagemann, Kathrin Horn, Kerstin Rohde‐Zimmermann, Anke Tönjes, Joachim Thiery, Markus Löffler, Ralph Burkhardt, Yvonne Böttcher, Michael Stumvoll, Matthias Blüher, Knut Krohn, Markus Scholz, Ronny Baber, Paul W Franks, Peter Kovacs, Maria Keller

**Affiliations:** ^1^ Medical Department III—Endocrinology, Nephrology, Rheumatology University of Leipzig Medical Center Leipzig Germany; ^2^ Helmholtz Institute for Metabolic Obesity and Vascular Research (HI‐MAG), Helmholtz Zentrum München, University of Leipzig and University Hospital Leipzig Leipzig Germany; ^3^ Institute for Medical Informatics, Statistics and Epidemiology University of Leipzig Leipzig Germany; ^4^ Medical Faculty University of Kiel Kiel Germany; ^5^ Institute of Clinical Chemistry and Laboratory Medicine University Hospital Regensburg Regensburg Germany; ^6^ Department of Clinical Molecular Biology Institute of Clinical Medicine University of Oslo Oslo Norway; ^7^ Medical Division Akershus University Hospital Lørenskog Norway; ^8^ Deutsches Zentrum für Diabetesforschung Neuherberg Germany; ^9^ Medical Faculty University of Leipzig Leipzig Germany; ^10^ LIFE Leipzig Research Center for Civilization Diseases University of Leipzig Leipzig Germany; ^11^ Genetic and Molecular Epidemiology Unit, Department of Clinical Sciences Lund University Skåne University Hospital Malmö Sweden

**Keywords:** alcohol, diet, DNA methylation, epigenetic clock, epigenetics, lifestyle score, physical activity, smoking

## Abstract

**Objective:**

Obesity is driven by modifiable lifestyle factors whose effects may be mediated by epigenetics. Therefore, we investigated lifestyle effects on blood DNA methylation in participants of the LIFE‐Adult study, a well‐characterised population‐based cohort from Germany.

**Research design and methods:**

Lifestyle scores (LS) based on diet, physical activity, smoking and alcohol intake were calculated in 4107 participants of the LIFE‐Adult study. Fifty subjects with an extremely healthy lifestyle and 50 with an extremely unhealthy lifestyle (5th and 95th percentiles LS) were selected for genome‐wide DNA methylation analysis in blood samples employing Illumina Infinium® Methylation EPIC BeadChip system technology.

**Results:**

Differences in DNA methylation patterns between body mass index groups (<25 vs. >30 kg/m^2^) were rather marginal compared to inter‐lifestyle differences (0 vs. 145 differentially methylated positions [DMPs]), which identified 4682 differentially methylated regions (DMRs; false discovery rate [FDR <5%) annotated to 4426 unique genes. A DMR annotated to the *glutamine‐fructose‐6‐phosphate transaminase 2* (*GFPT2*) locus showed the strongest hypomethylation (∼6.9%), and one annotated to *glutamate rich 1* (*ERICH1*) showed the strongest hypermethylation (∼5.4%) in healthy compared to unhealthy lifestyle individuals. Intersection analysis showed that diet, physical activity, smoking and alcohol intake equally contributed to the observed differences, which affected, among others, pathways related to glutamatergic synapses (adj. *p* < .01) and axon guidance (adj. *p* < .05). We showed that methylation age correlates with chronological age and waist‐to‐hip ratio with lower DNA methylation age (DNAmAge) acceleration distances in participants with healthy lifestyles. Finally, two identified top DMPs for the *alanyl aminopeptidase* (*ANPEP*) locus also showed the strongest expression quantitative trait methylation in blood.

**Conclusions:**

DNA methylation patterns help discriminate individuals with a healthy versus unhealthy lifestyle, which may mask subtle methylation differences derived from obesity.

## Graphical abstract

- Long‐term lifestyle habits including diet, physical activity, smoking and alcohol consumption jointly shape epigenetic patterns and affect methylation age.
- These effects clearly dominate over those driven by age and obesity alone.
- An interplay of lifestyle aspects needs to be considered when analysing epigenetic data with regard to complex metabolic diseases.

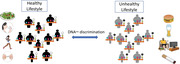

## 1 OBJECTIVE

Obesity is well recognised as a multifactorial disease in most modern societies, with not only individuals’ genetic background contributing to the disease burden but also with a crucial role of lifestyle and environment, strongly influencing epigenetic mechanisms controlling metabolic processes.^1^ However, common lifestyle intervention regimes vary greatly in structure and length and, thus, in their individual success on weight reduction (reviewed in Aronica et al.^2^). Observed direct effects, for example, on DNA methylation patterns after short‐term lifestyle interventions, are often marginal,^2^ which might be due to their short duration and low intensity.^3^ Recent studies have demonstrated that successful short‐term weight loss interventions may reduce methylation age (mAge) to the chronological age level.^4^ Furthermore, DNA methylation patterns may predict the success of lifestyle‐induced weight loss.^5^, ^6^, ^7^

Comprehensive studies investigating the underlying interaction between genetics, epigenetics and especially lifestyle are currently lacking. Therefore, we (1) analysed and compared the human blood DNA methylation patterns between subjects living a healthy lifestyle and those living an unhealthy lifestyle. (2) We further compared obese and nonobese subjects to identify DNA methylation patterns, which are related to an obese phenotype despite a healthy lifestyle or potentially associated with a healthy (lean) phenotype in an allegedly unhealthy obesogenic environment. (3) We elucidated lifestyle‐specific effects on the epigenetic clock. (4) Finally, we investigated the role of genetic variants cis to the identified target regions by methylation quantitative trait loci (meQTL) analyses and addressed the potential consequences of these changes on the blood transcriptome by matrix expression quantitative trait methylations (eQTMs).

## 2 RESEARCH DESIGN AND METHODS

### 2.1 Study population

The present analyses included participants of the LIFE‐Adult study, a population‐based cohort of European ancestry, focusing on lifestyle diseases.^8^, ^9^ The cohort comprised ∼10 000 adult subjects aged from 18 to 80 years (mean ± standard deviation [SD]: age = 57.4 ± 12.5 years, body mass index [BMI] = 27.3 ± 4.9 kg/m^2^) from the region of Leipzig, Germany. All participants underwent extensive phenotyping, including anthropometric measurements, social and lifestyle‐behaviour questionnaires and blood parameters. For most subjects, ethylenediaminetetraacetic acid (EDTA) blood samples are available.^8^ All participants gave written informed consent to participate in the study, and procedures were approved by the University of Leipzig's ethics committee (registration number: 263‐2009‐14122009) and conducted according to the Declaration of Helsinki. Study participation, assessments and interviews were supervised and carried out by trained staff and under supervised standard operation procedures.^8^

### 2.2 Lifestyle score

We created a lifestyle score (LS) as the sum of four different subscores: diet, physical activity (PA), alcohol consumption and smoking.^10^ To calculate the scores, we included data from four self‐reported questionnaires: (1) a German version of the Food Frequency Questionnaire,^11^ (2) the Short‐Form International Physical Activity Questionnaire,^12^ (3) a questionnaire about smoking status and quantity, and (4) a questionnaire about daily alcohol consumption and frequency. The final LS ranged from 3 to 66 (mean ± SD: 27.19 ± 11.2), with low and high LS values representing a healthy and an unhealthy lifestyle, respectively. All subscores showed an inter‐item correlation, as demonstrated by Cronbach's alpha statistic (*α* = 0.64). A detailed description of the individual scoring and an explanation of each subscore can be found in Supporting Information and Table S1. Subjects with any missing questionnaire items were completely excluded from further analyses to avoid potential effects caused by general noncompliance of those subjects. Similarly, participants with pre‐existing diabetic conditions (HbA1c ≥ 6.5%)^13^ or missing BMI measures were also excluded from subsequent analyses. A total of 4107 subjects passed all criteria (Table 1).

**TABLE 1:** Study characteristics

	LIFE cohort	Healthy lifestyle (LS ≤5th percentile)	Unhealthy lifestyle (LS ≥95th percentile)	*p*‐Values (LS 5th vs. 95th percentile)
*N* (total number)	4107	216	207	
Gender (*N*: female/male)	2109/1998	160/56	66/141	
Age (years)	55.9 ± 12.8	60.14 ± 12.76	54.03 ± 10.47	**1.22E‐9**
BMI (kg/m^2^)	27.03 ± 4.65	27.11 ± 4.74	27.31 ± 4.69	.51
BMI category (*N*: lean/obese/overweight)	1488/1659/940	73/95/48	67/93/46	
Waist circumference (cm)	95.71 ± 13.02	93.46 ± 12.18	99.52 ± 13.59	**3.09E‐6**
Waist‐to‐hip ratio	0.93 ± 0.09	0.9 ± 0.08	0.97 ± 0.08	**<2.2E‐16**
Fasting plasma glucose (mmol/L)	5.56 ± 0.79	5.63 ± 0.79	5.61 ± 0.72	.86
Fasting plasma insulin (pmol/L)	63.33 ± 42.82	58.53 ± 36.99	66.66 ± 43.77	.08
Plasma low‐density lipoprotein (mmol/L)	3.5 ± 0.95	3.5 ± 0.93	3.63 ± 0.97	.35
Plasma high‐density lipoprotein (mmol/L)	1.62 ± 0.46	1.79 ± 0.45	1.44 ± 0.42	**1.19E‐15**
Plasma apolipoprotein A1 (g/L)	1.67 ± 0.3	1.76 ± 0.27	1.6 ± 0.3	**1.27E‐9**
Plasma triglycerides (mmol/L)	1.38 ± 1.1	1.17 ± 0.58	1.68 ± 1.03	**2.78E‐9**
Diet score	12.39 ± 3.22	8.69 ± 1.94	15.39 ± 3.08	<2.2E‐16
Physical activity score	7.55 ± 6.47	0.58 ± 1.6	17.25 ± 3.8	<2.2E‐16
Smoking score	5.91 ± 6.70	0.09 ± 0.68	16.81 ± 3.58	<2.2E‐16
Alcohol score	1.34 ± 2.22	0.09 ± 0.68	2.97 ± 2.46	**<2.2E‐16**
LS	27.19 ± 11.02	9.45 ± 1.54	52.42 ± 4.07	**<2.2E‐16**

*Note*: Phenotypic data are described for all in the LS analysis included LIFE‐Adult subjects as well as for the healthy (≤5th percentile) and unhealthy (≥95th percentile) living extreme subgroups as the mean ± standard deviation (SD) (including discovery and validation cohort; detailed in Table S2). *p*‐Values show significant differences between healthy and unhealthy subgroups.

Abbreviations: BMI, body mass index; LS, lifestyle score.

### 2.3 Subset for genome‐wide methylation and validation measurements

Based on the LS calculation, we stratified the cohort into two groups reporting the most healthy and unhealthy lifestyles by selecting the lowest and highest 5% (5th percentile LS ≤ 11; 95th percentile LS ≥ 48). Within these groups (*N* = 234), we found 140 subjects with and 94 subjects without obesity according to BMI criteria.^14^ Based on this and an equal age range (Figure S1B), we further selected 25 subjects without (BMI < 25 kg/m^2^) and 25 subjects with obesity (BMI > 30 kg/m^2^) among each subgroup (5th vs. 95th percentile) (total *N* = 100) for the genome‐wide methylation discovery cohort and included all (with and without obesity) subjects with sufficient available DNA (*N* = 213) for validation analysis. Thus, both groups overlap in *N* = 100 samples and are therefore not independent.

**FIGURE 1:**
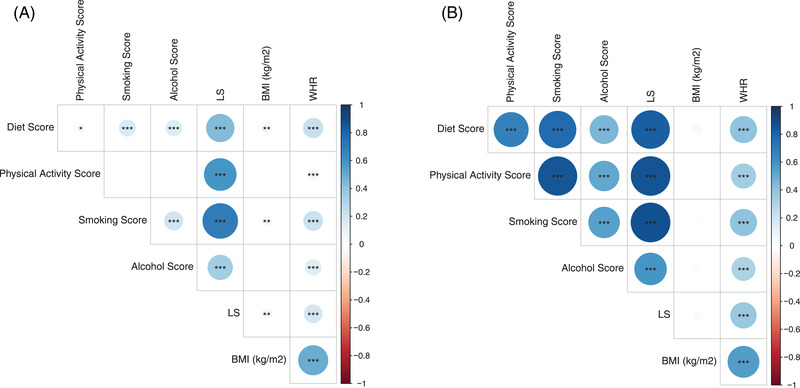
Lifestyle (sub)score correlations. Correlation analysis is shown between lifestyle scores (including subscores). Lifestyle score (LS) and anthropometric measurements are presented as a correlation matrix. The colour and size of the dots represent Spearman's correlation coefficient *r*; *p*‐values are indicated with ***p < .001, **p < .01, *p < .05. (A) Total cohort (*N* = 4107) and (B) significant correlations within the subscores in the validation cohort consisting of the extreme lifestyle edges (*N* = 213).

### 2.4 Sample preparation

All samples were isolated, stored and maintained at the Leipzig Medical Biobank^15^ according to standard protocols. Briefly, blood samples were taken after an overnight fast (mean fasting duration 12.7 ± 1.7 h) during the individuals’ study visit and stored at 4°C–8°C until DNA isolation (within 48 h after blood withdrawal) on the Autopure LS platform (Qiagen, Germany) using chemistry by Qiagen and Stratec Molecular (Stratec, Germany). Genomic DNA samples were stored at ‐80°C prior to integrity control using gel electrophoresis and concentration measurements of double‐stranded DNA using Quant‐iT PicoGreen dsDNA (Invitrogen, ThermoFisher Scientific, Germany) and Quantus (Promega, Germany) technologies.

### 2.5 Genome‐wide DNA methylation analysis

Five hundred nanograms of genomic DNA was taken for bisulphite conversion using an EZ DNA Methylation Gold Kit (Zymo Research, Netherlands). After quality control (QC), amplification and hybridisation on Illumina HumanMethylation850 Bead Chips (Illumina, Inc., San Diego, CA, USA), the Illumina iScan array scanner was used to quantify genome‐wide DNA methylation levels at 850K CpG sites per sample at single‐nucleotide resolution.

Raw data were first quality controlled using the QC report of the minfi R package (version 1.38.0).^16^, ^17^ Two samples that did not pass the badSampleCutoff of 10.5 were excluded during normalisation steps. Beta densities and control probes were within predicted specifications. Probes that did not pass the detection *p*‐value (*p*_detect_ = .01) in more than 1% of all 98 samples were excluded from the analysis (17 375 probes). Cross‐reactive probes (38 924 probes)^18^ and probes containing known single‐nucleotide polymorphisms (SNPs) (29 383 probes) at the CpG site (DNA region where a cytosine nucleotide is followed by a guanine nucleotide) were also filtered out by applying the maxprobes (version 0.0.2) and minfi R packages, respectively. In addition, probes on sex chromosomes were removed from the analysis subset (19 627 probes), as sex represents a larger source of variation in our methylation data. In total, 760 550 probes remained for the analysis. *β*‐Value generation and quantile normalisation were computed using the minfi R package^16^, ^17^ and adjusted for sex‐specific batch effects (see Figure S1A). Furthermore, we analysed the cell type composition using the Houseman approach^19^, ^20^, ^21^ adapted to EPIC arrays by Salas et al.^21^ Possible differences in cell type composition were (see Figure S2) analysed using Wilcoxon tests in R. We corrected *β*‐values for cell type composition in an attempt to reduce noise,^21^ although none of the cell type populations differed strongly between the subgroups (low and high LS) (Figure S2A) and the low and high LS subgroups in individuals without and with obesity (Figure S2B).

Differential methylation analyses were performed between subjects with extremely healthy and unhealthy lifestyles (low vs. high LS/5th vs. 95th percentile) as well as between participants without (BMI < 25 kg/m^2^) and with obesity (BMI > 30 kg/m^2^) within each lifestyle subgroup. The established R package limma (version 3.48.3) was used to identify differentially methylated CpG sites.^22^ Data assignment to technical and biological information by principal component analysis using the R package SWAMP (version 1.5.1)^23^ showed that array slides were the primary batch for which we adjusted accordingly (Figure S3). Differentially methylated positions (DMPs) describe differences in methylation levels of single CpG positions with an adj. *p*‐value <.05. Differentially methylated regions (DMRs) were extracted by applying DMRcate (version 2.6.0),^24^ which uses Gaussian kernel smoothing to find patterns of differential methylation independent of genomic annotation. Only DMRs with more than two CpG sites were reported. DMRs with a minimum smoothed FDR <5% were defined as differentially methylated. DMRs with a mean methylation difference >±2% were further annotated to CpG islands (CpG shores, CpG shelves and inter‐CpG islands (CGIs)) and gene context‐related regions (promoters, 5ʹ untranslated regions (UTRs), exons, introns, 3ʹUTRs and intergenic regions). Genomic annotation was performed using the annotatr R package (version 1.18.1)^25^ with respect to multiple annotations. To elucidate putative drivers of blood DNA methylation, separate analyses with individual covariates (smoking, diet, PA, alcohol, BMI and age) were performed. Intersection analysis for covariate‐specific effects on lifestyle DMRs was performed using the UpsetR (version 1.4.0) package.^26^ EPIC raw data are available at the Leipzig Health Atlas under https://www.health‐atlas.de/studies/57.

### 2.6 Methylation age and telomere length clocks

DNA methylation age (DNAmAge), corresponding DNAmAge acceleration differences according to Horvath's clock (I and II), Levine's clock and the telomere length clock were estimated using the R package methylclock (version 0.7.5).^27^

### 2.7 KEGG pathway overrepresentation

Candidate genes identified by significant DMRs (minimum smoothed FDR <5%) characterising lifestyle‐specific methylation differences (healthy vs. unhealthy living subjects) and differences between subjects without and with obesity were taken forward for a KEGG pathway overrepresentation test using clusterProfiler::enrichKEGG (version 3.18.1).^28^ Enrichment *p*‐values were adjusted using Benjamini–Hochberg correction, and FDR <5% was considered statistically significant.

### 2.8 Validation of candidate CpGs

We selected two top candidate DMPs (Tables 2 and S8) from our discovery cohort (high LS vs. low LS) for additional validation using bisulphite sequencing. Briefly, 300 ng of genomic DNA was bisulphite converted using the EpiTect Fast DNA Bisulfite Kit (Qiagen). After a whole genomic amplification using the EpiTect Whole Bisulfitome Kit (Qiagen), candidate regions were amplified and sequenced using the PyroMark Q24 platform and self‐designed assays for *retinoic acid receptor alpha* (*RARA*) and *F2R like thrombin or trypsin receptor 3* (*F2RL3*) candidate DMPs (Qiagen). Primer sequences are shown in Table S3. All analyses were performed in duplicate, including two non‐template controls per sequence run.

**TABLE 2:** Top lifestyle‐specific differentially methylated positions (DMPs)

Log fold change	Average expression	*t*‐Value	*p*‐Value	Adj. *p*‐value	*β*‐Value	Chromosome	Position	Strand	CpG name	UCSC RefGene name
Top hypomethylated DMPs										
−0.08590341	0.611707079	−5.998155947	3.32E‐08	.001904	7.711471263	chr15	90345999	−	cg06344992	*ANPEP*
−0.078774849	0.42479436	−5.431011449	4.05E‐07	.014379921	5.260694048	chr3	22412124	+	cg05529343	−
−0.076464321	0.588248951	−5.523118419	2.72E‐07	.012223739	5.650244644	chr15	90346089	−	cg02008229	*ANPEP*
−0.062518007	0.406780726	−5.085850571	1.75E‐06	.02663562	3.833220233	chr17	73824396	−	cg07010633	*UNC13D*
−0.060472705	0.627160734	−5.216582762	1.01E‐06	.021387415	4.36768357	chr16	70838524	+	cg16450432	−
−0.058989842	0.656861761	−4.879931872	4.10E‐06	.036747315	3.007614002	chr17	73824620	−	cg23891399	*UNC13D*
−0.058856163	0.721651694	−5.641262144	1.62E‐07	.008069468	6.154917623	chr15	90346094	−	cg23432008	*ANPEP*
−0.05865088	0.2639215	−4.825085	5.12E‐06	.04057961	2.791195	chr2	241976080	−	cg26718213	*SNED1*
−0.058146578	0.501398588	−4.822217419	5.18E‐06	.040621906	2.779921567	chr1	14928945	−	cg08538034	*KIAA1026*
−0.055549608	0.643279716	−4.851889553	4.59E‐06	.038439331	2.896775344	chr4	72119734	+	cg13530673	*SLC4A4*
Top hypermethylated DMPs										
0.193803612	0.681740677	9.330229512	3.34089E‐15	2.48875E‐09	23.67151784	chr5	373378	+	cg05575921	*AHRR*
**0.089458236**	**0.502052675**	**8.742631823**	**6.28569E‐14**	**2.34122E‐08**	**20.75139172**	**chr19**	**17000585**	−	**cg03636183**	***F2RL3***
0.086788359	0.554523661	4.995475984	2.54941E‐06	.030145161	3.468373598	chr1	92947588	+	cg09935388	*GFI1*
0.083336995	0.62903076	7.843482601	5.36488E‐12	7.99298E‐07	16.33498038	chr2	233284661	−	cg21566642	−
0.082181848	0.281667406	7.78234353	7.23702E‐12	8.90834E‐07	16.03813512	chr11	86513429	+	cg14391737	*PRSS23*
**0.078959254**	**0.30069194**	**7.752577018**	**8.37097E‐12**	**8.90834E‐07**	**15.8938087**	**chr17**	**38477572**	−	**cg17739917**	***RARA***
0.07293225	0.467577109	7.867015889	4.78042E‐12	7.99298E‐07	16.44938219	chr2	233284934	−	cg01940273	−
0.062807746	0.693318329	8.305152712	5.51963E‐13	1.37059E‐07	18.5921592	chr19	16998668	+	cg21911711	*F2RL3*
0.060416895	0.182824945	4.908439221	3.64634E‐06	.035276471	3.120689146	chr19	14591033	+	cg20742389	*GIPC1*
0.057960459	0.560933206	5.47153733	3.40315E‐07	.013342801	5.431662125	chr14	77248049	+	cg10387007	*VASH1*

*Note*: Included are the top 10 significantly hypermethylated and hypomethylated DMPs (adj. *p*‐value < .05) between healthy and unhealthy living subjects. Our selected candidates for bisulphite validation are highlighted.

### 2.9 Transcriptome data

Transcriptome data were available from Illumina HT‐12 v4 Expression Bead Chips (Illumina) using whole blood RNA samples from the LIFE‐Adult cohort as described elsewhere.^8^, ^29^ Data processing was performed using R/Bioconductor after extraction of all 47 231 gene‐expression probes using Illumina GenomeStudio without background correction. Furthermore, expression values were log2 transformed and quantile normalised,^30^, ^31^ and batch effect correction was performed using an empirical Bayes method.^32^ Probes were excluded when expressed in less than 5% of the (subgroup‐specific) samples (detected by Illumina GenomeStudio), still being associated with batch effects after Bonferroni correction or not mapping to a gene accordingly^33^ (accessed on 4 April 2019). Additionally, gene probes without available annotation and genes on the X and Y chromosomes were removed to determine the effects introduced by sex. In summary, 20 114 valid gene‐expression probes were identified corresponding to 14 687 single genes in the human genome (hg19). A three‐step procedure was used to remove poor quality samples: (1) first, the number of detected gene‐expression probes of a sample was required to be within ±3 interquartile ranges (IQR) from the median, (2) the Mahalanobis distance of several quality characteristics of each sample (signal of AmbionTM ERCC spike‐in control probes, signal of biotin‐control probes, signal of low‐concentration control probes, signal of medium‐concentration control probes, signal of mismatch control probes, signal of negative control probes and signal of perfect‐match control probes)^34^ had to be within median +3 × IQR, and (3) Euclidean distances of expression values as described^31^ had to be within 4 × IQR from the median. Overall, of the 3526 assayed samples, 107 samples were excluded for quality reasons. Raw transcriptome data are available at the Leipzig Health Atlas under https://www.health‐atlas.de/studies/57.

### 2.10 Genotype data

For genotypes, 7838 LIFE‐Adult participants were genotyped using the genome‐wide SNP array Affymetrix Axiom CEU1 and the software Affymetrix Power Tools (version 1.20.6). QC of the genotyped data was performed following Affymetrix's data analysis guide^35^ as previously described.^36^ QC according to Affymetrix's data analysis guide included dish‐QC (<0.82), sample call rate (<97%), sex mismatch, ambiguous relatedness (e.g., sample mix‐up) and abnormalities of XY intensity plots (e.g., XXY samples filtered for gonosomal analyses). Genetic heterogeneity was evaluated with principal component analyses, and outliers (>6 SD in any of the first 10 principal components) were removed. The criteria call rate, parameters of cluster plot irregularities according to Affymetrix's recommendations, violation of Hardy–Weinberg equilibrium (*p*‐value < 10^‐6^) in an exact test for autosomes (*p*‐value < 10^‐4^), for chromosome X with females only^37^ and batch association (*p*‐value < 10^‐7^) were considered during SNP QC. Subsequently, 7669 samples and 532 676 SNPs were imputed on the reference 1000 Genomes Phase 3,^38^ applying SHAPEIT^39^ v2r900 (prephasing) and IMPUTE2 (version 2.3.2)^39^, ^40^ for genotype estimation. A specific genotype was assigned to a SNP if its corresponding genotype estimation featured a probability of ≥.8.^41^ In 2.5% of the cases, none of the genotypes exceeded that threshold, and the respective SNP was labelled ‘missing’ for that sample. SNPs whose ‘missing’ count over all samples exceeded the upper quartile +1.5 × IQR were removed, resulting in a total of 2830 SNPs.

### 2.11 matrixEQTL analysis

Among samples with significant DMP data (from DMRs healthy vs. unhealthy), additional gene‐expression and SNP data were available for 48 samples. The R package matrixEQTL (version 2.3)^42^ was employed on all three pairs of datasets to identify cis effects (within a range of ±1 kb) between methylation and expression (eQTMs), methylation and SNPs (meQTLs) and expression and SNPs (eQTLs). All three comparisons were performed on the complete data (*N* = 48) and both subgroups with high LS (*N* = 23) and low LS (*N* = 25) separately. Since sex batch effects have been adjusted for in both expression and methylation data, small batch effects remained only for age and BMI. However, including both age and BMI as covariates into the matrixEQTL analysis did not change the overall result, which is why the final matrixEQTL analysis was run without considering any covariances.

### 2.12 Statistics

All statistical analyses were performed using R software (version 4.0.4).^43^ After checking for normal distribution, Mann–Whitney *U*‐test or Welch's *t*‐test was applied to test for differences between the 5th and 95th percentiles as well as between lean and obese subgroups for the following phenotypes: BMI, age, HbA1c, waist‐to‐hip ratio (WHR), fasting plasma glucose and insulin, low‐density lipoprotein (LDL), high‐density lipoprotein (HDL), apolipoprotein A1 (Apo A1) and triglyceride serum levels. Welch's *t*‐test was used to compare methylation differences measured as normalised *β*‐values between low LS versus high LS for each top DMP, respectively. Using the Shapiro–Wilk test to prove the normal distribution of the bisulphite sequencing data, an independent Mann–Whitney *U*‐test was applied to compare methylation differences within the validation cohort. Methylation levels between BMI categories were compared by applying two‐way analysis of variance (ANOVA). Correlation analysis was performed using Spearman's correlation. All respective analyses were adequately corrected for multiple testing.

## 3 RESULTS

### 3.1 Self‐reported lifestyle reflects obesity‐specific phenotypes

We correlated the LS with BMI and WHR in 4107 LIFE‐Adult participants (mean ± SD: age = 56 ± 13 years, BMI = 27.0 ± 4.7 kg/m^2^, LS = 27.19 ± 11.02) (Table 1). LS was related to the obesogenic environment (Figure S4A,B) (all *p‐*value < 1 × 10^‐3^) with significantly higher values in subjects with obesity (Figure S4C). We further demonstrated that all individual scores (diet, PA, smoking, alcohol and total LS) were mutually dependent (Figure 1A), which was particularly marked in the extreme subgroups (5th and 95th percentile, Figure 1B). Finally, our score showed simultaneous negative correlations (all *p‐*value < 1 × 10^‐15^) to the protective lipid parameters HDL cholesterol and Apo A1, which were higher in healthy living subjects (Figure S5A,B).

The discovery cohort comprised 44 males and 56 females with a mean BMI of 28.6 ± 6.5 kg/m^2^. The validation cohort (*N* = 213; 5th/95th percentile = 100/113; lean/obese = 131/82) included 84 males and 129 females with a mean BMI of 27.1 ± 6.2 kg/m^2^ (Table S2). When comparing healthy versus unhealthy subgroups (5th vs. 95th percentile LS, healthy LS: *N* = 216, unhealthy LS: *N* = 207), nominal differences were observed regarding BMI distribution (Figure S5, Table 1) (*N* lean/overweight/obese: healthy LS—73/95/48, unhealthy LS—67/93/46; *p* = .051). Although HbA1c, fasting plasma glucose, fasting plasma insulin and LDL serum levels did not differ between the two groups (all *p‐*value > .05, Table 1), strong differences were found for waist circumference (*p‐*value < 3 × 10^‐6^), age, Apo A1 and triglyceride serum levels (all *p‐*value < 1 × 10^‐9^) and especially HDL levels (*p‐*value < 1 × 10^‐15^) and WHR (*p‐*value < 2.2 × 10^‐16^). Subjects living an extremely healthy lifestyle were older (mean age difference = 6 years) with lower WHR (mean WHR difference = 0.08) and prominently lower lipid serum levels. However, as expected, all phenotypes differed significantly between subjects with and without obesity within healthy and unhealthy subgroups (all *p‐*value < 2 × 10^‐2^), respectively, except for fasting plasma LDL. Regarding sex distribution, it is noticeable that females are overrepresented in the healthy subgroup, whereas males (Table 1) dominate the unhealthy subgroup.

### 3.2 DNA methylation signatures are related to individual's lifestyle

**FIGURE 2:**
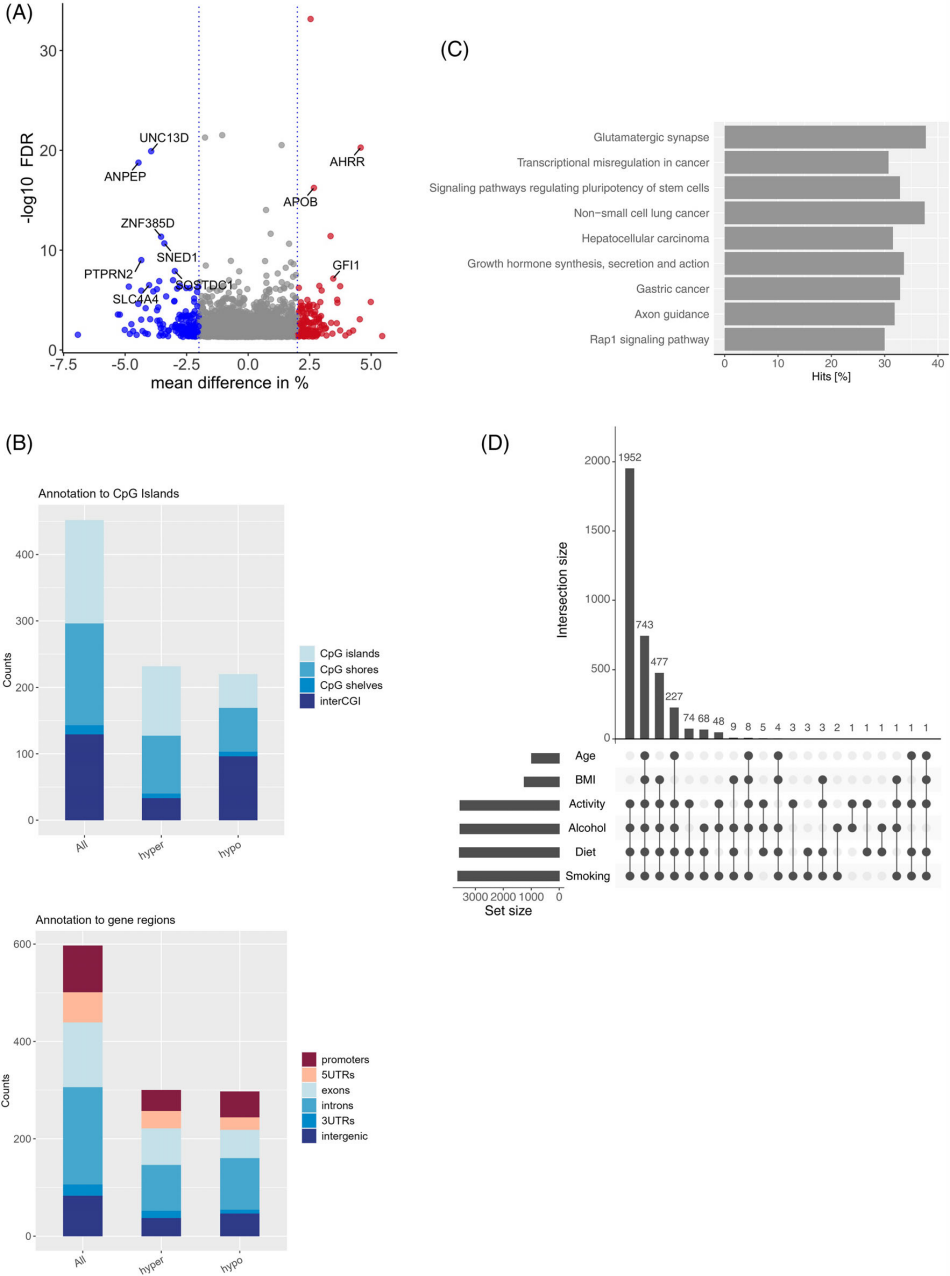
Lifestyle‐specific differentially methylated regions (DMRs) in the discovery cohort. (A) Volcano plot representing the significant DMRs (minimum smoothed FDR <5%) based on the healthy versus unhealthy lifestyle comparison. Positive mean methylation differences ≥|2|% represent hypermethylated DMRs (red dots), and negative methylation differences ≥|2|% represent hypomethylated DMRs (blue dots) in the healthy subgroup. (B) The location of the DMRs in relation to CpG islands (top) and the location of the DMRs in relation to gene regions (bottom). Both plots are presented as the number of counts, including multiple annotations. Hyper: hypermethylation; hypo: hypomethylation. (C) KEGG pathway enrichment analysis presented as the percentage of annotated genes relative to all genes involved in the respective pathway (hits in %) for all enriched pathways with an FDR <5%. (D) Intersection plot illustrating the frequency of significant DMRs driven by any of the included lifestyle aspects (diet, physical activity, smoking and alcohol) and the potential confounders age and body mass index (BMI). The majority of the DMRs are driven by an interaction between all four lifestyle aspects

By comparing genome‐wide blood DNA methylation patterns in subjects with healthy versus unhealthy lifestyles, we identified 4682 significant DMRs annotated to 4426 genes with a minimum smoothed FDR <5%, which included 220 DMRs with FDR <1 × 10^‐4^ (Table S4, Figure 2A). Among the significant DMRs, the mean methylation level differences ranged from ‐6.9% to 5.5%.

Given the rather subtle methylation changes for the majority of the DMRs, we introduced a mean methylation threshold of >2% to further narrow down the potential causal candidate DMRs. Among the 340 DMRs reaching this cut‐off, 164 DMRs were hypermethylated (mean methylation difference ± SD: 2.6 ± 0.6%), whereas 176 DMRs were hypomethylated (mean methylation difference ± SD: ‐2.8 ± 0.8%) in healthy compared to unhealthy living individuals (Figure 2A, Table S4). Taking into account that a DMR can have more than one annotation, most DMRs (46%; counts relative to the number of DMRs) were located in CpG islands, followed by 45% located in CpG shores. In relation to gene regions, most DMRs are located in introns (59%), followed by exons (39%) (Figure 2B).

The top 15 hypomethylated and hypermethylated significant DMRs according to their mean methylation difference are presented in Table 3 with a DMR annotated to the *glutamine‐fructose‐6‐phosphate transaminase 2* (*GFPT2*) gene locus showing the strongest hypomethylation (mean methylation difference DMR = ‐6.9%). A DMR annotated to the *glutamate rich 1* (*ERICH1*) gene showed the strongest hypermethylation (mean methylation difference DMR = 5.4%). Finally, using KEGG pathway analyses, we identified glutamatergic synapses as the most enriched pathway (adj. *p*‐value < .01) followed by axon guidance, another brain‐related pathway (adj. *p*‐value < .05). Most of the nine enriched pathways (Table S5, Figure 2C) are related to various cancer types.

**TABLE 3:** Top lifestyle‐specific differentially methylated regions (DMRs)

Chromosome	Start	End	Number CpGs	Minimum smoothed FDR	Maximum difference	Mean difference	UCSC RefGene name
Top hypomethylated DMRs							
chr5	179740743	179741120	4	0.029977938	−0.115525323	−0.069213857	*GFPT2*
chr3	53700141	53700263	3	0.000280372	−0.058166831	−0.052935571	*CACNA1D*
chr10	130726406	130726701	3	0.000287833	−0.066141442	−0.052150296	*NA*
chr16	55866757	55867072	4	0.009779257	−0.084082934	−0.050156174	*CES1*
chr20	55835831	55836676	4	4.50739E‐07	−0.063006443	−0.048500957	*BMP7*
chr4	169770092	169770406	3	0.024472101	−0.051377777	−0.04809308	*PALLD*
chr1	58898552	58898793	3	0.002601555	−0.062311841	−0.047451925	*DAB1*
chr10	128810484	128810904	3	0.013077709	−0.057183472	−0.046745655	*DOCK1*
chr20	61590751	61591066	3	0.03067276	−0.048455427	−0.045342149	*SLC17A9*
chr3	29377160	29377980	3	2.4182E‐05	−0.072396235	−0.044645175	*RBMS3*
Top hypermethylated DMRs							
chr8	637468	637909	3	0.040227828	0.096584379	0.054484963	*ERICH1*
chr10	90984672	90985062	3	1.55E‐05	0.056039743	0.049841417	*LIPA*
chr6	29648161	29649084	21	1.27008E‐08	0.070683917	0.046436136	*ZFP57*
chr5	373378	374252	4	5.36E‐21	0.193803612	0.045739382	*AHRR*
chr2	113992694	113994035	9	8.42E‐04	0.061941445	0.045375298	*PAX8;PAX8‐AS1*
chr6	291687	292823	9	0.011323863	0.053011157	0.042649384	*DUSP22*
chr10	3282585	3282783	3	0.018194186	0.042427159	0.041105344	*NA*
chr11	6592066	6592585	4	0.035060323	0.04489839	0.039528971	*DNHD1*
chr17	45949677	45949878	5	4.08384E‐07	0.050310751	0.037408698	*NA*
chr20	17595355	17595472	3	0.010978827	0.058269466	0.037403949	*RRBP1*

*Note*: Included are the top 15 significantly hypermethylated and hypomethylated DMRs (minimum smoothed FDR <5%) between healthy and unhealthy living subjects.

As demonstrated by the intersection plot (Figure 2D, Table S6), the majority of the DMRs (*N* = 1952) were driven by all four lifestyle subscores together (diet, PA, smoking and alcohol), followed by a combination of them together with BMI and age (*N* = 743). Obviously, BMI and age alone do not explain any of the identified DMRs. Although this did not indicate a prominent role of smoking (Figure 2D), given the nature of the LS, a comparison between participants with very healthy and very unhealthy lifestyles mirrors differences between nonsmokers and smokers. We therefore further adjusted the complete analyses for smoking as a covariate, which resulted in 629 identified DMRs with a minimum smoothed FDR <5%. Among them, the most significant DMR is located within a CpG island of the *ring finger protein 39* (*RNF39*) locus (Table S7).

### 3.3 Lifestyle‐derived DMPs correlate with metabolic traits related to obesity

**FIGURE 3:**
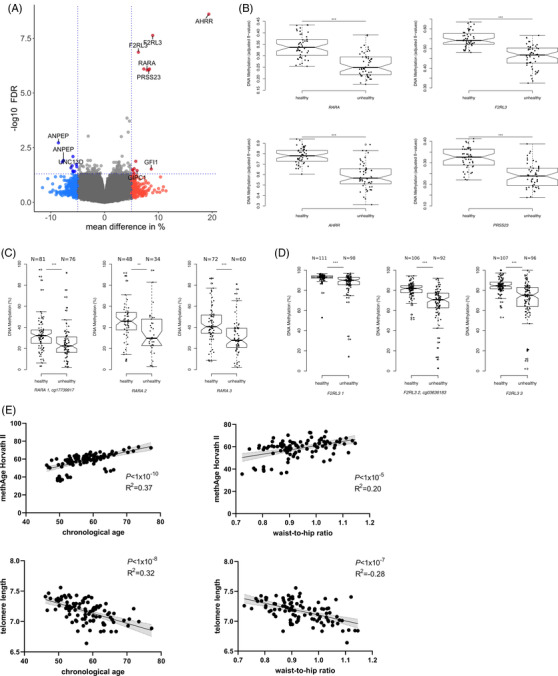
Differentially methylated positions (DMPs) comparing healthy versus unhealthy lifestyle in the discovery (A and B) and validation cohorts (C and D): (A) volcano plot representing the significant DMPs (minimum smoothed FDR <5%) based on the healthy versus unhealthy lifestyle comparison. Positive mean methylation differences ≥|5|% represent hypermethylated DMPs (red dots) and negative methylation differences ≥|5|% represent hypomethylated DMPs (blue dots) in the healthy subgroup. (B) Box plots representing the mean methylation ± standard deviation (SD) for the top four identified genes: *retinoic acid receptor alpha* (*RARA*) (cg17739917); *F2R like thrombin or trypsin receptor 3* (*F2RL3*) (cg03636183); *aryl hydrocarbon receptor repressor* (*AHRR*) (cg05575921); *serine protease 23* (*PRSS23*) (cg14391737) comparing healthy (low lifestyle score [LS]) versus unhealthy (high LS) living subjects; the 95% confidence interval is represented by notches. *p*‐Values indicate statistically significant differences detected using Welch's *t*‐test. (C and D) Box plots are given as the mean methylation ± SD, and the 95% confidence interval is represented by notches for the two validated DMPs (C) *RARA* and (D) *F2RL3* and their surrounding CpGs. *p*‐Values indicate statistical significance between healthy (low LS) and unhealthy (high LS) subjects detected using analysis of variance (ANOVA). *p*‐Values are indicated as *p < .05, **p < .01 and ***p < .001. (E) Linear regression analysis between methyl age (methAge) for the Horvath II, telomere length, chronological age and waist‐to‐hip ratio (WHR) measurements presented as a scatter plot. The light grey area represents the 95% confidence interval, and *R* ^2^ represents the coefficient of determination

DMP‐specific analysis comparing subjects with healthy and unhealthy lifestyles identified 145 significant DMPs (adj. *p*‐value < 5%) (Figure 3A, Table S8). A total of 26 DMPs passed a mean methylation change |(log fold change FC])| ≥5%, 14 of which were hypermethylated log FC ≥5% (0.07 ± 0.04), while 12 showed hypomethylation log FC ≤‐5% (‐0.06 ± 0.01). Of these, 19 DMPs were clearly assigned to a specific gene. However, when considering significance levels (adj. *p‐*value < .05) as well as a mean methylation change ≥5%, the strongest effects were observed for *aryl hydrocarbon receptor repressor* (*AHRR*), *F2RL3*, *RARA* and *serine protease 23* (*PRSS23*) (Figure 3B, all *p*‐value < 1 × 10^‐11^, Table S8), all of which were hypomethylated under unhealthy conditions. Correlation analysis between the identified DMPs and obesity‐related phenotypes (HbA1c, LDL, HDL, blood glucose, insulin, Apo A1, triglycerides, BMI, waist circumference, WHR and age) as well as the LS and its subscores can be found in Table S9. Two of the 20 included DMPs showed a significant correlation with WHR even after Bonferroni correction, although no SNPs within the genomic region have been shown to be associated with BMI‐adjusted WHR in a previous GWAS^44^ (GWAS catalogue, accessed June 2021). Among the identified DMPs, the strongest correlation with WHR was found for *vasohibin 1* (*VASH1*) (*p*‐value = 4.2 × 10^‐5^, *r* = ‐0.4), which is in line with an association with HbA1c (%) (*p*‐value = .03, *r* = ‐0.21), triglycerides (mmol/L) (*p*‐value = .046, *r* = ‐0.2) and waist circumference (cm) (*p*‐value = .04, *r* = ‐0.2), although only the association with WHR was sustained after correction for multiple testing. Finally, two DMPs of *F2RL3*, one of our selected top hits, showed marginal correlations to LDL serum levels (all *p*‐value < .03, *r* = ‐0.22) and triglycerides (mmol/L) (all *p*‐value < .07, *r* = ‐0.27) and one DMP in addition to HbA1c (%) (*p*‐value < .01, *r* = ‐0.3). The selected top DMPs for *F2RL3* showed additional correlations to HDL (mmol/L) (*p‐*value = .03, *r* = 0.23) and Apo A1 (g/L) (*p*‐value = .03, *r* = 0.23), albeit not withstanding corrections for multiple testing.

In summary, 13 of the 20 selected DMPs showed marginal correlations with triglycerides (all *p*‐value < .046), eight with HDL (all *p*‐value < .047) and seven with LDL (all *p*‐value < .033) serum levels (Table S9).

### 3.4 Bisulphite sequencing within *RARA* and *F2RL3* loci supports findings from the discovery stage

Based on these findings and further supported by previously reported data,^45^, ^46^, ^47^ we selected two DMPs (*F2RL3:cg03636183* and *RARA:cg17739917*) for validation (*N* = 213) using bisulphite sequencing and demonstrated directionally consistent differences between subjects with healthy versus unhealthy lifestyles (Figure 3C,D; both *p*‐value < .001, mean methylation difference: *RARA* CpG = 8.37%, *F2RL3* CpG2 = 13.87%). Furthermore, the surrounding CpG position confirmed this effect, with all *p*‐value <.01 and a mean methylation difference of >5.59% (mean methylation difference: *RARA* CpG2 = 11.99%, *RARA* CpG3 = 12.58%, *F2RL3* CpG1 = 5.59%, *F2RL2* CpG3 = 13.2%) (Figure 3C,D). The validation cohort used here did not differ significantly from the discovery cohort regarding age, BMI and sex distribution (Table 1). Spearman's correlation analysis showed a significant correlation between the methylation levels observed in the genome‐wide methylation analysis and bisulphite sequencing (*RARA*: Spearman's *r* = 0.45, *F2RL3*: Spearman's *r* = 0.53, both *p *< 7.9 × 10^‐5^) (Figure S5C,D).

### 3.5 Obesity‐specific methylation marks

**FIGURE 4:**
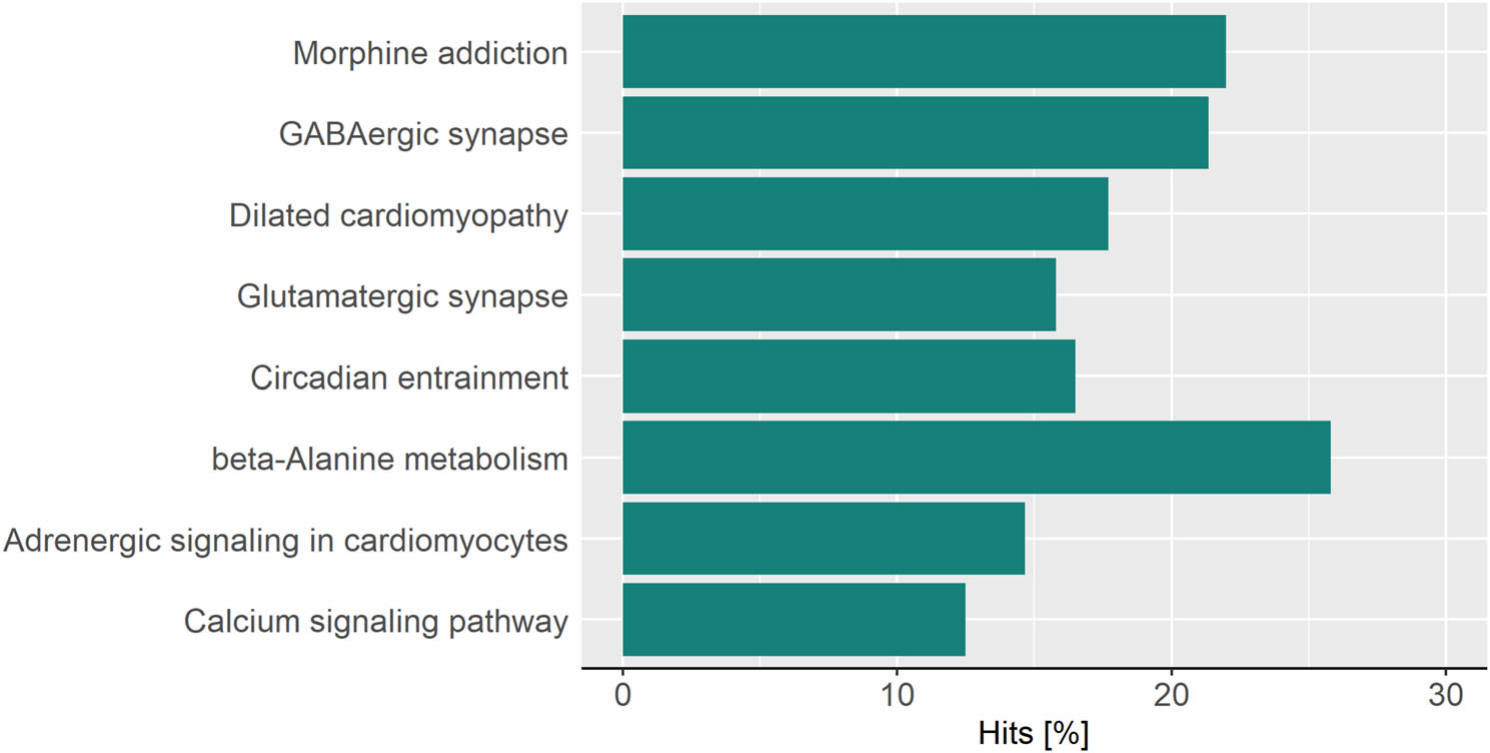
KEGG pathway enrichment analysis of the healthy subgroup. Body mass index (BMI)‐related (nonobese vs. obese) KEGG pathway enrichment in the low lifestyle score (LS) subgroup is presented as hits in percent for all pathways with an FDR <0.05.

Driven by the comparable distribution of subjects with and without obesity between the very healthy and unhealthy lifestyle groups, we aimed to identify lifestyle‐independent obesity‐related methylation marks. Therefore, the blood methylation patterns of subjects with obesity (*N* = 25) were compared with those of subjects without obesity (*N* = 25) within each lifestyle group separately. Interestingly, whereas approximately 1572 DMRs annotated to 1599 different genes were identified in healthy subjects, only 85 DMRs annotated to 101 genes were detected in subjects living an unhealthy lifestyle (Tables S10 and S11) with a minimum smoothed FDR <5%. This further included 10 identical annotations among the *PAX6* and *HOXA9‐10* gene clusters, already known candidates regarding obesity and related comorbidities. However, at CpG levels, no DMPs were sustained after correction for multiple testing (data not shown). Nevertheless, KEGG pathway analysis for the healthy subgroup indicated eight enriched pathways, among them GABAergic synapse, dilated cardiomyopathy and calcium signalling (Figure 4, Table S12), whereas for the unhealthy subgroup, only antigen processing and presentation were enriched (not shown).

### 3.6 Methylation age

We observed the strongest association (*p*‐value < 1 × 10^‐10^, *R* ^2^ = 0.37, Figure 3E) between mAge and subjects’ chronological age within the discovery cohort for Horvath's clock II, which was compared to Horvath's clock I, which was additionally trained on 850K EPIC arrays (Figure S6A–C). Only marginal (*p*‐value = .01) differences in DNAmAge acceleration were observed when comparing individuals with healthy versus unhealthy lifestyles, which was similar to comparing never smokers with previous or current smokers (Figure S6D,F). No difference was observed between subjects with and without obesity (Figure S6E). We further observed a strong negative association between the telomere length clock and chronological age (*p*‐value < 1 × 10^‐8^, *R* ^2^  =  ‐0.32, Figure 3E). Interestingly, both clocks showed an additional linear association with WHR within our discovery cohort (all *p*‐value < 1 × 10^‐4^, Figure 3E).

### 3.7 Underlying genetic predispositions and effects on mRNA levels in blood

Driven by the small overlapping sample size (*N* = 48) and only marginal genetic variation in close proximity (±1 kb) to the identified target DMRs (healthy vs. unhealthy lifestyle), we could not identify any meQTLs or eQTLs. However, we found associations between the methylation levels of eight DMPs with target mRNA expression levels (Table S13) in the combined discovery group (all individuals with healthy and unhealthy lifestyles). Among them, only two eQTMs annotated to the *alanyl aminopeptidase* (*ANPEP*) locus were sustained after correction for multiple testing (matrix FDR = 0.03; Table S13, Figure S7). Four eQTMs were detected in subjects with healthy lifestyle and eight with unhealthy lifestyle; among them, one of our candidate DMP of *F2RL3* was also detected in healthy subjects; however, none maintained after correction for multiple testing.

## 4 DISCUSSION

Epigenetic markers are known to reflect environmental conditions and thereby are affected not only by genetic predisposition but also most strongly by our daily lifestyle. Although this is widely acknowledged by the scientific community, the majority of epigenetic studies in regard to obesity, most of them conducted cross‐sectionally, still lack the inclusion of relevant lifestyle drivers.^48^ Therefore, to the best of our knowledge, this is one of the few studies investigating the potential effects of lifestyle on the respective blood DNA methylation signatures.^49^ Here, we calculated LS scores based on each individual's diet, PA, smoking and alcohol consumption within the LIFE‐Adult study from Germany. Genome‐wide DNA methylation analysis in blood samples of 100 subjects representing healthy and unhealthy lifestyle extremes demonstrated that daily lifestyle is most likely superior to the obesity state itself in associations with blood DNA methylation patterns, as supported by association studies between neonatal blood methylation and the risk of developing obesity later in life.^50^ The study showed that the distribution of obesity categories in extreme lifestyle groups was comparable and that potentially obesity‐associated methylation marks were more frequent in subjects with healthy lifestyles. However, this could also be driven by the general exclusion of subjects suffering from diabetes, which may have inadvertently excluded subjects with unhealthy metabolic obesity. Furthermore, mAge and estimated telomere length showed strong correlations with chronological age and WHR, with observed smaller DNAmAge acceleration distances in healthy subjects. Finally, two DMPs for *ANPEP* also showed the strongest eQTM in blood within the subgroup of 48 subjects.

With this study, we took several lifestyle aspects into account to explore relations between long‐term lifestyle habits and differences in human blood methylation patterns. Our findings imply that dietary habits, PA, smoking habits and alcohol consumption influence epigenetic patterns together, whereas only neglectable effects are attributed to age and BMI alone. This suggests that rather than simply representing the consequence of obesity, differences in blood‐derived methylation marks may be primarily driven by long‐term lifestyle habits. This is further supported by the observed smaller DNAmAge acceleration in the healthy lifestyle group compared to the unhealthy lifestyle group, whereas no significant difference could be observed between subjects with and without obesity.

We identified several candidate genes differentially methylated according to the LS and successfully validated *RARA* and *F2RL3*, already known from previous studies to be influenced by lifestyle aspects and acknowledged for their role in metabolic diseases.^45^, ^47^, ^51^ Both genes were hypermethylated within extremely healthy compared to unhealthy living individuals, which is in line with previously published data.^41^, ^45^, ^52^ In particular, hypomethylation of the DMP within the *F2RL3* locus appears to increase the risk for cardiovascular as well as overall mortality.^45^, ^52^ Translated to our results, this might indicate an increasing mortality risk of an unhealthy lifestyle accompanied by associated diseases, such as obesity, type 2 diabetes, cardiovascular diseases or cancer. Previous studies further showed a hypomethylating effect of smoking on the *F2RL3* DMP identified here.^51^ Moreover, very recently, a strong association with coffee consumption in a large‐scale epigenome‐wide association study (EWAS) was reported.^53^ Consistent with published data on smoking, subjects with unhealthy lifestyle in our study showed a mean methylation of 67% compared to 81% in the healthy lifestyle group, with the majority of subjects within the unhealthy group being actual smokers (validation cohort). In line with this, we further observed a marginal (*p*‐value = .04) positive correlation between this methylation of DMP and *F2RL3* mRNA levels in the healthy lifestyle subgroup.

We found significant methylation differences between the healthy and unhealthy lifestyles for *RARA*, which is known for its role in adipogenesis.^54^ It is noteworthy, however, that based on the findings of the present study, an increase in the *RARA* methylation pattern might be related to higher HDL cholesterol and lower triglyceride serum levels, indicating a link between *RARA* and lipid metabolism.

Although the observed differences in DNA methylation in *RARA* could also be driven by smoking as previously described^46^ and supported by the strong correlation with smoking found here, there is still a prominent influence of other environmental conditions, such as diet and PA, as shown by our present data. Nevertheless, it needs to be acknowledged that in line with our study, the majority of methylation studies on smoking, although lacking any information on diet or activity, identified a similar set of top candidates, especially *F2RL3*, *RARA* and *AHRR*, in human blood cells.^41^, ^45^, ^47^, ^52^ It is also worth mentioning that smoking effects on *F2RL3* methylation were previously also observed in adipose tissue.^47^ Since we included smoking as a lifestyle factor, it is possible that some of the identified genes are indeed related to lung cancer.^55^, ^56^ Consequently, narrowing down our list of potential lifestyle discriminating candidate regions by including an additional adjustment for smoking resulted in the identification of a top DMR on chromosome 6 annotated to the *RNF39*. This DMR overlapped with a larger region very recently described to successfully discriminate responders from nonresponders to a lifestyle intervention based on either a Mediterranean/low‐carbohydrate or low‐fat diet with or without PA.^7^

There are a few key limitations to our study. First, we used a scoring system based on self‐reported questionnaires, which might lead to euphemistic information, including over‐ or underestimation of the real status.^57^ However, our study design is supported and strengthened by findings, which are in line with previously reported data, for example, on lifestyle factors such as smoking.^45^, ^47^ Furthermore, although we excluded individuals suffering from diabetes, we cannot fully exclude effects driven by other non‐diabetes‐related medications. Finally, the observational and cross‐sectional nature of the study does not allow testing the direction of causality at least between methylation and metabolic phenotypes and limits our ability to rule out confounding (e.g., sex), even though it seems unlikely that methylation marks affect lifestyle habits.

Although the identification of reliable and reproducible epigenetic marks for obesity in human blood remains challenging, our study clearly indicates the importance of considering as many lifestyle aspects as possible when analysing epigenetic data with regard to complex diseases such as obesity. We successfully demonstrated that the majority of CpG methylation marks are much more strongly influenced by our daily lifestyle than the obesity state itself.

## CONSENT FOR PUBLICATION

Not applicable.

## CONFLICT OF INTEREST

Matthias Blüher received honoraria as a consultant and speaker from Amgen, AstraZeneca, Bayer, Boehringer‐Ingelheim, Lilly, Novo Nordisk, Novartis and Sanofi. All other authors declare no competing interests.

## Supporting information



Supplemental Figure 1 Sex‐ and age‐specific quality control: (A) Principal component analysis (PCA) of the Illumina 850K methylation arrays. After sex normalisation, no batch effect for sex is visible anymore, but the separation for the healthy (high) and unhealthy (low) living subjects is evident. (B) Estimation plot using an unpaired *t*‐test for age differences between low versus high lifestyle score (LS) (healthy vs. unhealthy) subgroups

Supplemental Figure 2 Cell type distribution: box plots representing the cell type proportions for (A) low and high lifestyle score (LS) subgroups and (B) for low and high LS subgroups in individuals without (two) and with obesity (four) based on Illumina 850K methylation data. CD8 T‐lymphocytes (CD8T), CD4 T‐lymphocytes (CD4T), natural killer cells (NK), B‐lymphocytes (Bcell), monocytes (Mono) and neutrophils (Neu)

Supplemental Figure 3 *β*‐Values densities of the Illumina 850K methylation data: the densities of *β*‐values are shown for (A) the raw data and (B) after adjustment for cell counts, array slides and sex. Since the arrays were run on two batches, differences are visible in the raw data and had to be corrected

Supplemental Figure 4 Lifestyle score (LS) associations in the LIFE cohort: scatter dot plots representing Spearman's correlations between the LS and (A) body mass index (BMI) in kg/m^2^ and (B) waist‐to‐hip ratio (WHR) for the total cohort (*N* = 4107). (C) Bar plot representing the LS values as the mean ± standard error of mean (SEM) for defined BMI categories. Intergroup differences were assessed using Student's *t*‐test; *p < .05

Supplemental Figure 5 Lifestyle score (LS) correlations in the total LIFE cohort (*N* = 4107): scatter plots representing Spearman's correlations between the LS and (A) high‐density lipoprotein (HDL) cholesterol serum levels (mmol/L) and (B) apolipoprotein A (Apo A1) serum levels (mmol/L). Spearman's correlation analysis between methylation levels observed in the Illumina EPIC arrays and the pyrosequencing sequencing validation (*N *= 213) are shown in (C) for the *retinoic acid receptor alpha* (*RARA*) and (D) for the *F2R like thrombin or trypsin receptor 3* (*F2RL3*) identified CpG position

Supplemental Figure 6 Methyl age (MethAge) correlations in the discovery cohort: Spearman's correlation between MethAge for (A) Horvath I, (B) Horvath II and (C) Levine's clock and individuals chronological age in the discovery cohort (*N *= 100) presented as scatter dot plot. (D–F) Plots presenting differences in DNA methylation age (DNAmAge) acceleration as the mean ± standard error of mean (SEM) between (D) healthy and unhealthy living subjects, (E) subjects without and with obesity and (F) never versus current/previous smokers

Supplemental Figure 7 Spearman's correlation between normalised methylation for *alanyl aminopeptidase* (*ANPEP*) differentially methylated positions (DMPs) (cg23422008 and cg02008229) and corresponding normalised mRNA expression (ILMN_1763837) presented as a scatter plot

Figure information

Supplemental Material

Table information

Supplemental Table 1 Detailed scoring system: diet score was assessed using Food Frequency Questionnaire (FFQ) information; favourable food intake got decreased scoring points with increased frequency, and unfavourable food intake reached higher scores with regular consumption (high diet scores indicate unhealthy nutrition intake). Physical activity was rated via activity categories and MET‐min/week divided into quartiles, counted by the Short‐Form International Physical Activity Questionnaire (SF‐IPAQ, less active means higher scores). Smoking assessment is based on actual smoking status and information about pack years (higher nicotine consumption means higher scores). Alcohol consumption was considered harmful by the cut‐offs based on Deutsche Gesellschaft für Ernährung (DGE) recommendations (exceeded consumption means higher scores)
Supplemental Table 2 Study characteristics of the subgroups: phenotypic data are described for discovery and validation cohort subjects within the healthy (≤5th percentile) and unhealthy (≥95th percentile) living extreme subgroups as the mean ± standard deviation (SD)
Supplemental Table 3 Self‐designed primer sequences: self‐designed primer sequences were used to validate candidate CpGs (*F2R like thrombin or trypsin receptor 3* [*F2RL34*] and *retinoic acid receptor alpha* [*RARA*]) with the pyrosequencing technique based on the PyroMark Q24 platform
Supplemental Table 4 All lifestyle‐specific differentially methylated regions (DMRs): overview of all significant (minimum smoothed FDR <5%) DMRs of the healthy versus unhealthy lifestyle analysis
Supplemental Table 5 Lifestyle‐specific KEGG pathway enrichment analysis: KEGG pathway overrepresentation analysis for differentially methylated regions (DMRs) from the healthy versus unhealthy lifestyle score analysis. All significant DMRs were included. Enrichment *p*‐values were adjusted using Benjamini–Hochberg correction, and FDR <5% was considered statistically significant
Supplemental Table 6 Differentially methylated regions (DMRs) of the lifestyle subscores: overview of all significant DMRs of the healthy versus unhealthy lifestyle analysis including individual adjustments for confounders (body mass index [BMI] and age) and all subscores (diet, physical activity [PA], smoking and alcohol)
Supplemental Table 7 Lifestyle‐specific differentially methylated regions (DMRs) after smoking adjustment: overview of all significant DMRs from the healthy versus unhealthy lifestyle analysis with a minimum smoothed FDR <5% after additional adjustment for smoking score are shown
Supplemental Table 8 All lifestyle‐specific differentially methylated positions (DMPs): overview of all statistically significant (adj. *p*‐value < .05) DMPs from the healthy versus unhealthy lifestyle study are given. Our selected candidates (*F2R like thrombin or trypsin receptor 3* [*F2RL34*] and *retinoic acid receptor alpha* [*RARA*]) for bisulphite validation are highlighted
Supplemental Table 9 Lifestyle‐specific differentially methylated positions (DMPs) correlation analysis: correlation analyses between obesity‐related phenotypes and the top 10 hypermethylated and hypomethylated DMPs between healthy versus unhealthy lifestyle within the discovery cohort are given. *p*‐Values maintained after correction for multiple testing [adj. *p*‐values < 1.5 × 10^‐4^; 0.05/(number included DMPs × number obesity‐related phenotypes)], are indicated in bold
Supplemental Table 10 Differentially methylated regions (DMRs) of subjects with healthy lifestyle: overview of all significant DMRs within subjects with healthy lifestyle comparing nonobese versus obese phenotypes
Supplemental Table 11 Differentially methylated regions (DMRs) of subjects with unhealthy lifestyle: overview of all significant DMRs within subjects with unhealthy lifestyle comparing nonobese versus obese phenotypes
Supplemental Table 12 KEGG pathway enrichment analysis of subjects with healthy lifestyle: KEGG pathway overrepresentation analysis for differentially methylated regions (DMRs) from nonobese versus obese phenotypes in subjects with extreme healthy lifestyle. All DMRs with FDR <5% were included. Enrichment *p*‐values were adjusted using Benjamini–Hochberg correction, and only hits with an FDR <0.05 were considered statistically significant
Supplemental Table 13 Expression quantitative trait methylation analysis (cis‐eQTM): cis‐eQTM analysis for the combined discovery cohort and the healthy and unhealthy lifestyle groups is given. All differentially methylated positions (DMPs) with a *p*‐value <.05 are listed, including the corresponding *t*‐test statistic and *β*‐values as effect size estimates according to matrixEQTL, gene locus, strand and (University of California Santa Cruz) UCSC annotation. The associated expression probe is listed with its gene locus and annotation according to Illumina. The DMP *p*‐values were adjusted using Benjamini–Hochberg correction, and only hits with an FDR <5% were considered statistically significant and are highlighted in bold
