# Supplementary material for: DNA methylation patterns reflect individual's lifestyle independent of obesity
Source: Clin Transl Med. 2022 Jun 12;12(6):e851. doi: 10.1002/ctm2.851 (PMC9189420; doi:10.1002/ctm2.851)

Supplemental Figure 1

a)

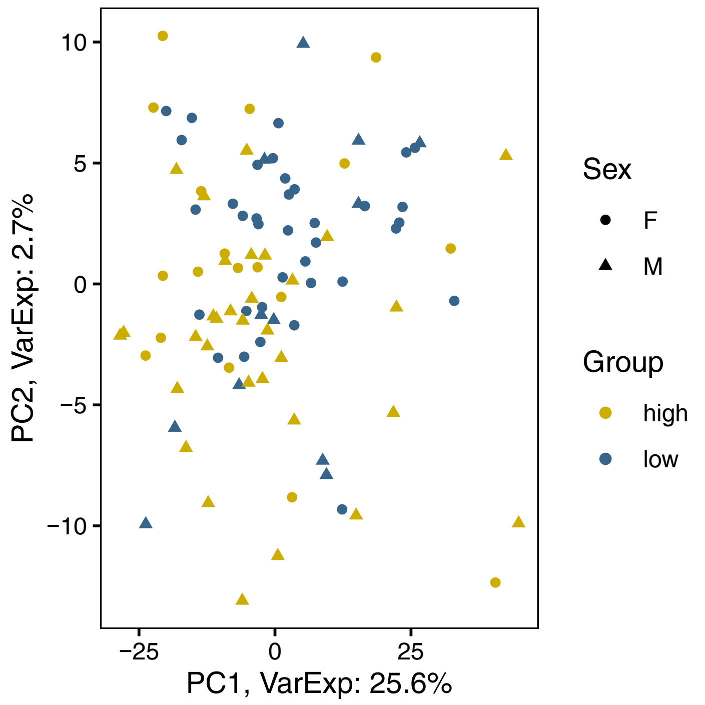

b)

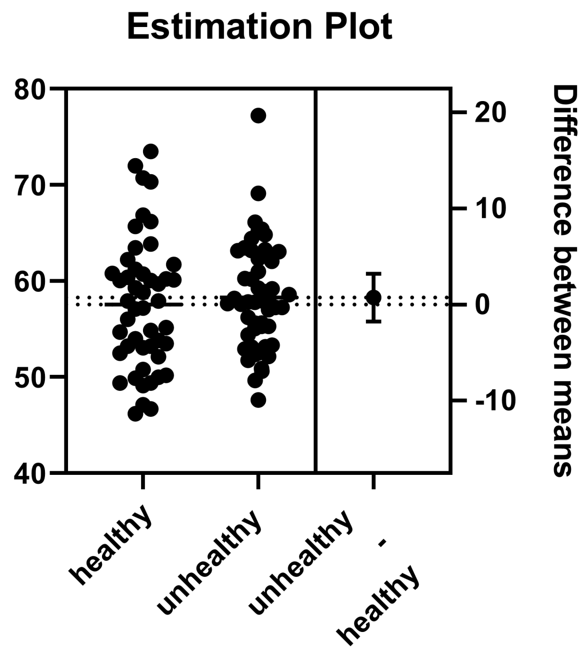

Supplemental Figure 2

a)

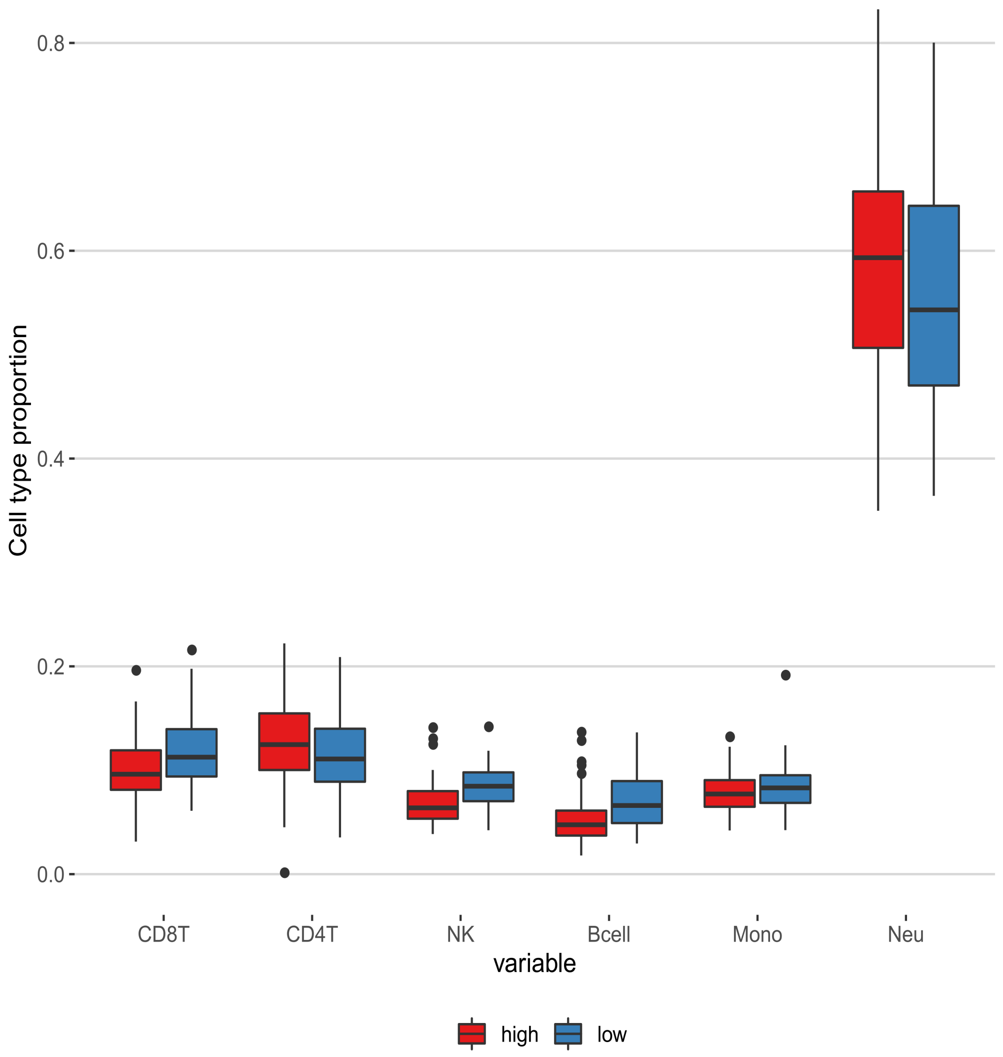

b)

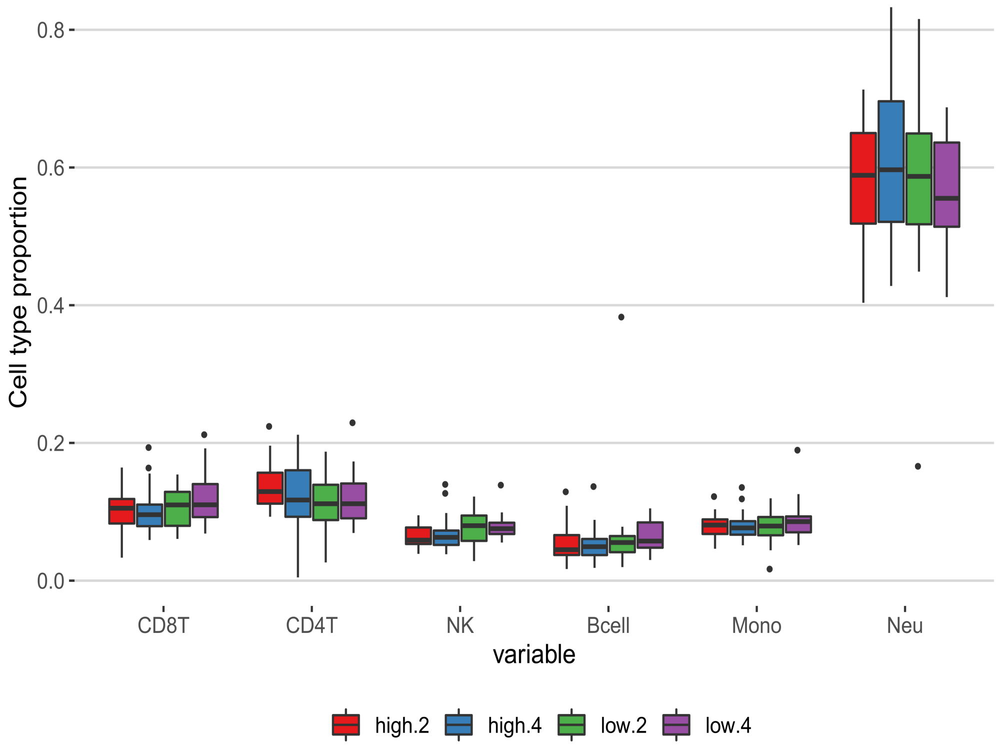

Supplemental Figure 3

a) b)

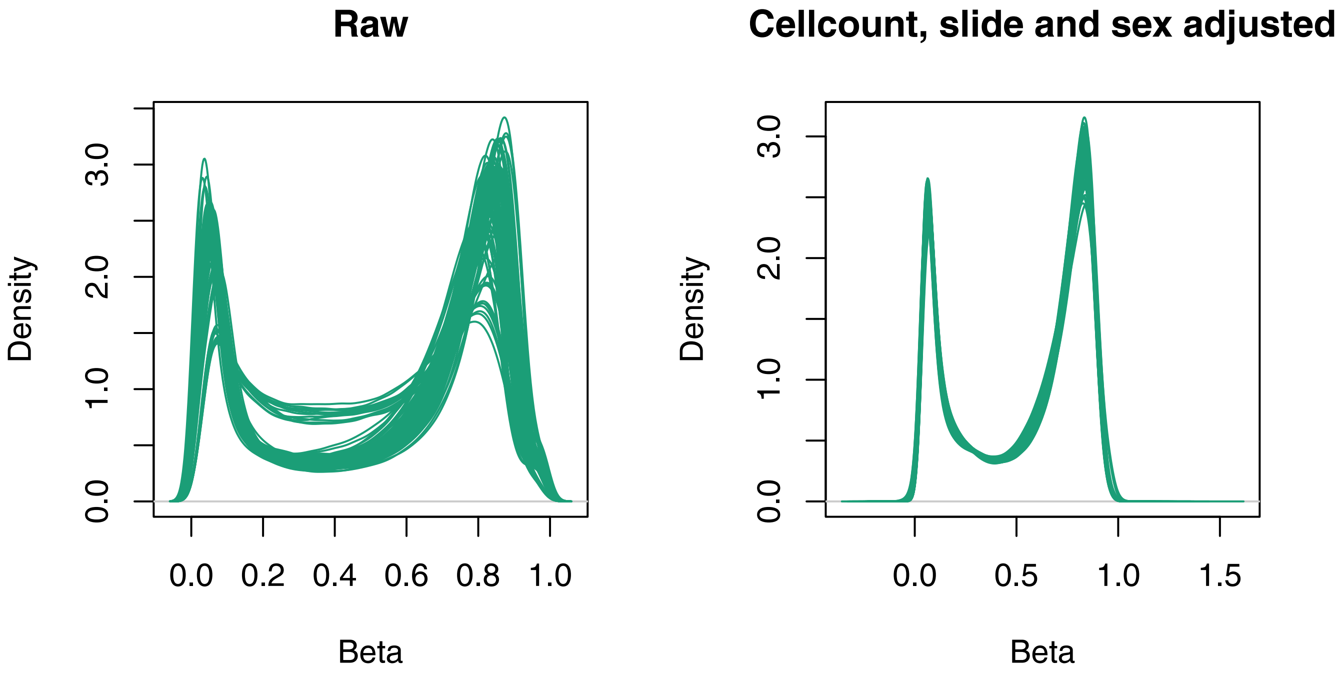

Supplemental Figure 4

a)

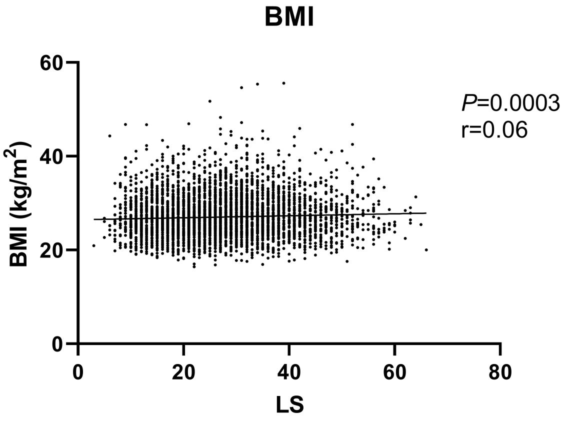

b)

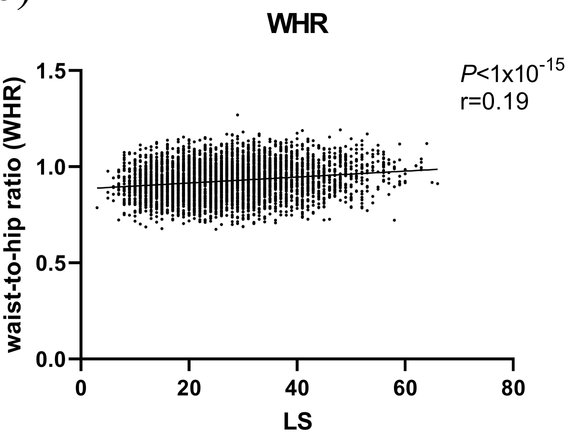

c)

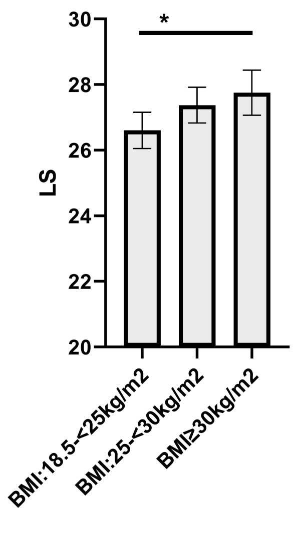

Supplemental Figure 5

a)

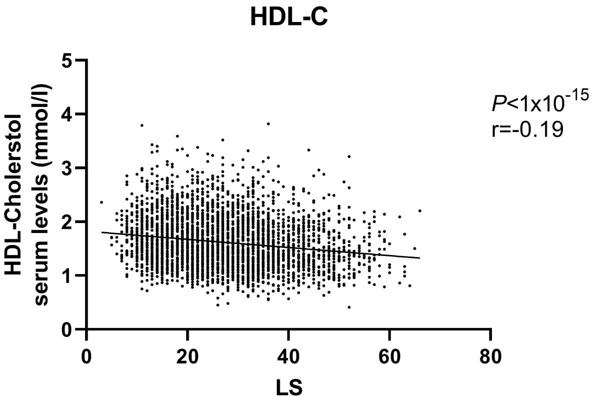

b)

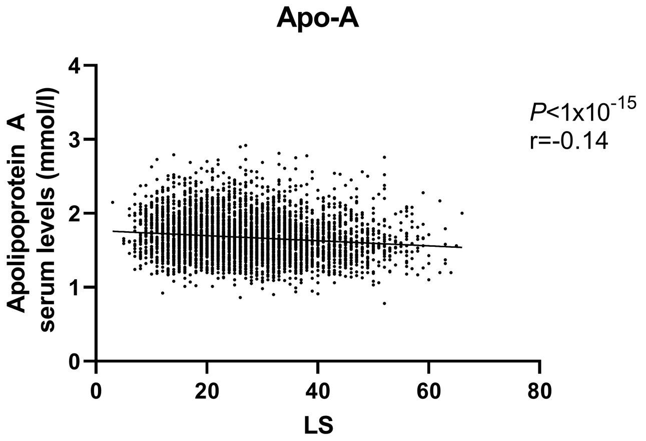

c)

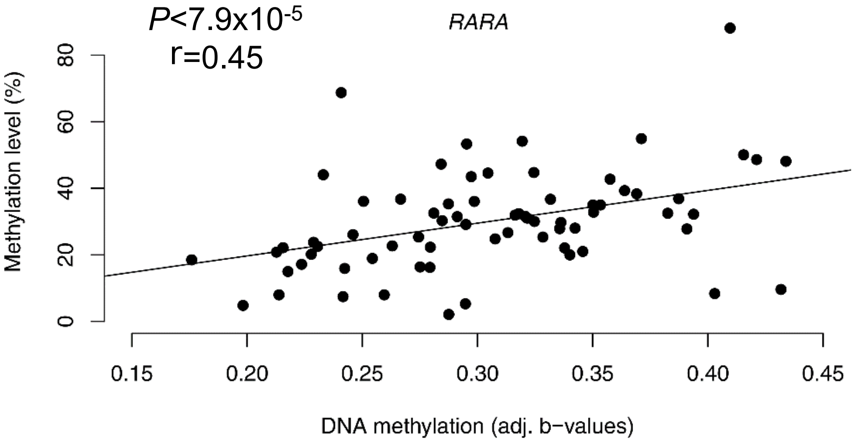

d)

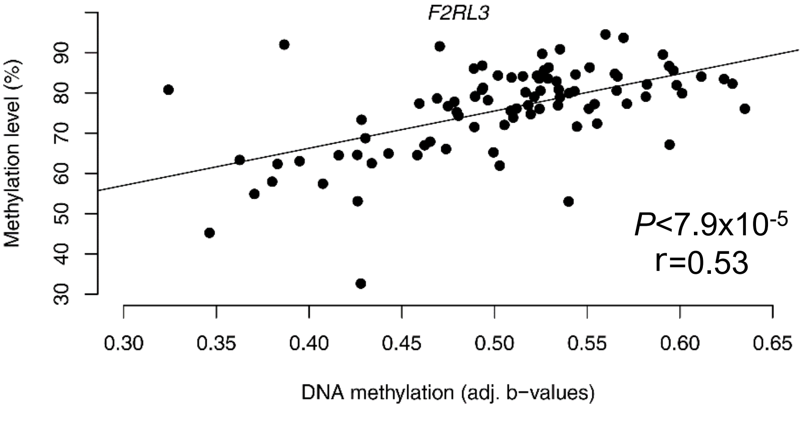

Supplemental Figure 6

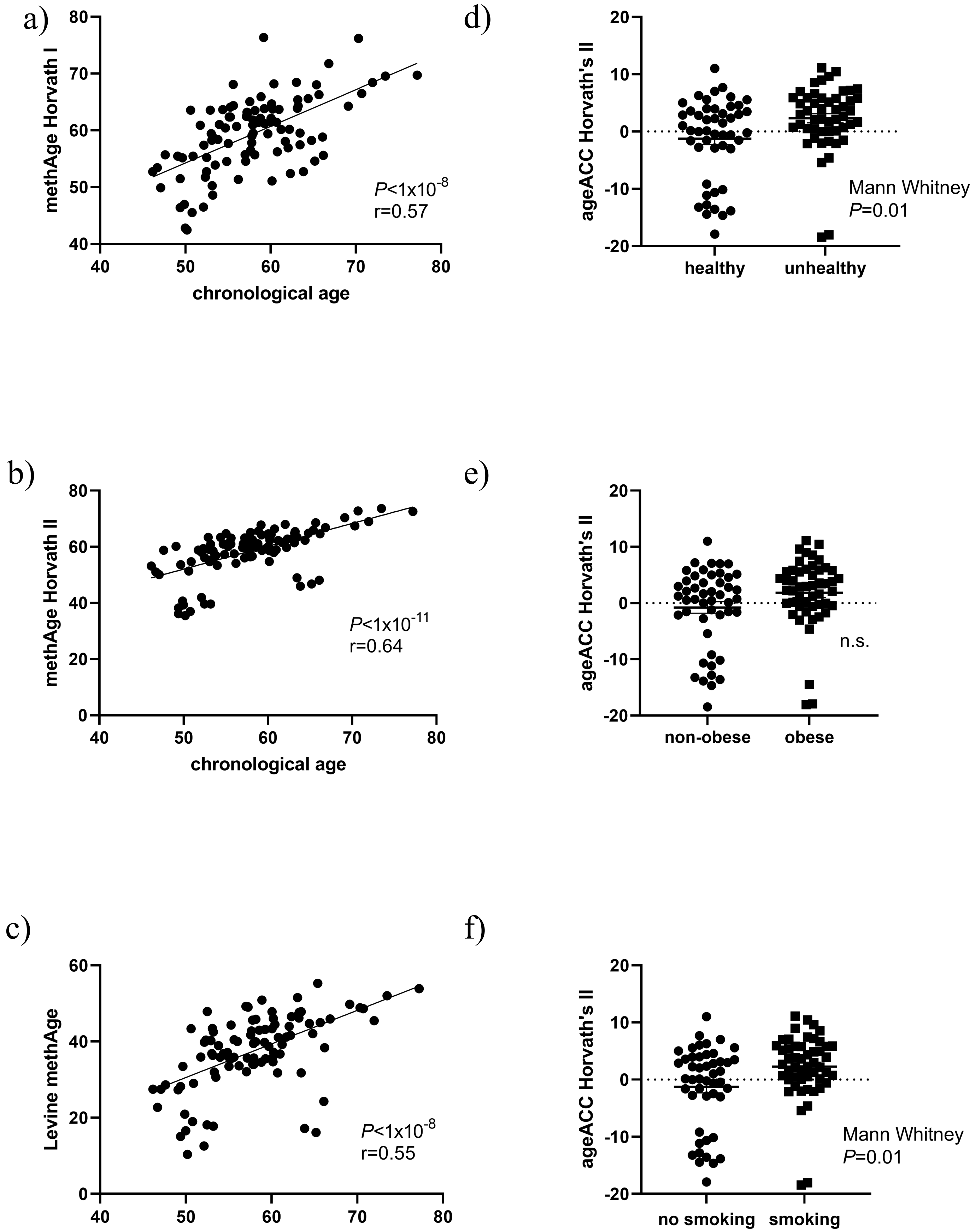

Supplemental Figure 7

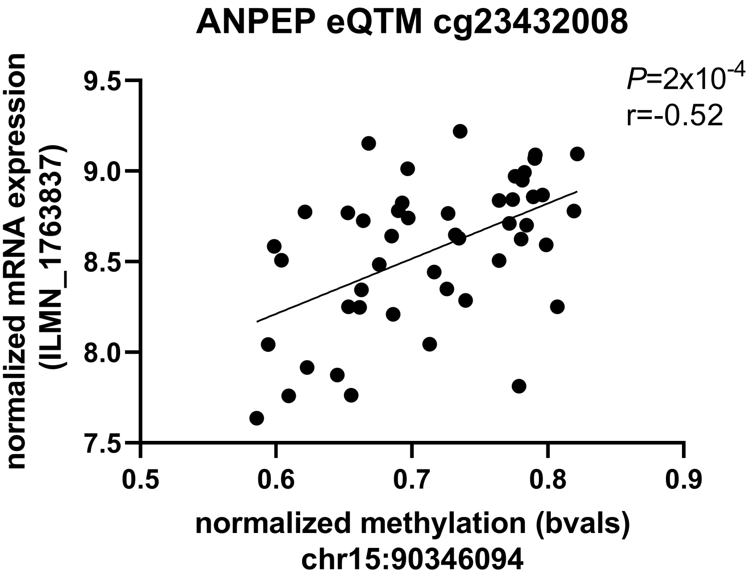

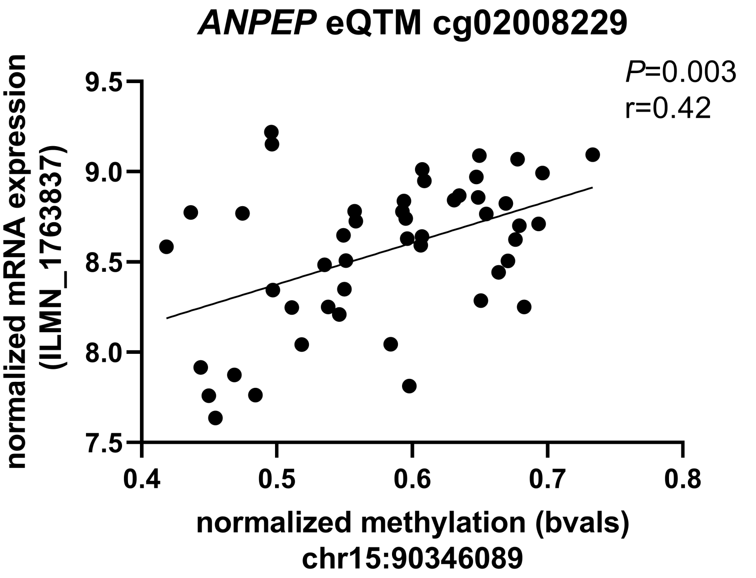

Supplement: Supplementary file 8 — Figure information [file CTM2-12-e851-s009.pdf]
