# Supplementary material for: DNA methylation patterns reflect individual's lifestyle independent of obesity
Source: Clin Transl Med. 2022 Jun 12;12(6):e851. doi: 10.1002/ctm2.851 (PMC9189420; doi:10.1002/ctm2.851)
Supplement: Supplementary file 9 — Supplemental Material [file CTM2-12-e851-s005.pdf]

Supplemental Material 1

Lifestyle sub-scores

Briefly, the diet score was based on the German FFQ where all participants were asked about their frequency in consumption of 34 different food groups and 13 beverages during the last year [1]. Among them we considered six food groups as favorable (e.g. fish, vegetables, and whole grain products) and eight as unfavorable (e.g. pan-fried potato products, meat and cold cuts, fast-food, and instant products), after testing their correlation to BMI. All Participants could choose from several categories: eating each food group a) several times per day, b) each day or almost each day, c) several times per week, d) once per week, e) two or three times per month, f) once or less a month, g) or rather never [2] (Supplemental Table 1). For the diet score, frequently consumed unfavorable foods counted high, whereas frequently consumed favorable foods counted low (Supplemental Table 1). In line with this, a high diet score correspond to an unfavorable eating pattern as previously described [3].

For the physical activity score we used the SF-IPAQ [4] and included the absolute activity measured as Metabolic Equivalent of Task-minutes per week (MET-min/week) and the categorical physical activity level as a) low, b) moderate, and c) intensive, as results of the questionnaire evaluation algorithm [1,5]. We scored the activity levels from intense to moderate to low (0-5-10) and used quartiles from the entire cohort distribution of MET-minutes/week for the absolute activity scoring as shown in Supplemental Table 1. In total, the physical activity score was calculated as sum of absolute and categorical activity levels.

For smoking behavior, we used the available data from the LIFE-Adult cohort regarding the current smoking status (non-smoker, previous smoker and current smoker) as well as the number of pack years (number of years with an average of one cigarette pack per day). Zero points were assigned to non-smoking, five points to previous smoking, and the maximum of 10 points to current smokers. For converting the number of pack years into scoring points, we used again the classification into quartiles as shown in Supplemental Table 1. The sum of the two scores provided the final smoking score.

Finally, based on the recommendation of the German nutrition society (DGE) we set thresholds for alcohol consumption to create the alcohol score at ten grams per day for females and 20 grams for males. Subjects below or equaling these cut offs scored 0 and those above the thresholds were assigned 5 points.

References

1. Loeffler, M. *et al.* The LIFE-Adult-Study: objectives and design of a population-based cohort study with 10,000 deeply phenotyped adults in Germany. *BMC public health* **15,** 691; 10.1186/s12889-015-1983-z (2015).

2. Kroke, A. *et al.* Validation of a self-administered food-frequency questionnaire administered in the European Prospective Investigation into Cancer and Nutrition (EPIC) Study: comparison of energy, protein, and macronutrient intakes estimated with the doubly labeled water, urinary nitrogen, and repeated 24-h dietary recall methods. *Am J Clin Nutr* **70,** 439–447; 10.1093/ajcn/70.4.439 (1999).

3. Nettleton, J. A. *et al.* Meta-analysis investigating associations between healthy diet and fasting glucose and insulin levels and modification by loci associated with glucose homeostasis in data from 15 cohorts. *American journal of epidemiology* **177,** 103–115; 10.1093/aje/kws297 (2013).

4. Craig, C. L. *et al.* International physical activity questionnaire: 12-country reliability and validity. *Medicine and science in sports and exercise* **35,** 1381–1395; 10.1249/01.MSS.0000078924.61453.FB (2003).

5. The IPAQ group. The IPAQ. Available at https://sites.google.com/site/theipaq/.
