# Supplementary material for: DNA methylation patterns reflect individual's lifestyle independent of obesity
Source: Clin Transl Med. 2022 Jun 12;12(6):e851. doi: 10.1002/ctm2.851 (PMC9189420; doi:10.1002/ctm2.851)
Supplement: Supplementary file 10 — Table information [file CTM2-12-e851-s010.pdf]

Supplemental Table 1 - Detailed Scoring System

Lifestyle-Score:	***Diet score***		***Physical activity score***		***Smoking score***		***Alcohol score***			***final lifestyle score***
FFQ-Code	Score unfavorable foods (a)	Score favorable foods (b)	Category (a)	Score	Smoking Status (a)	Score	sex	Alcohol intake (g/d) (a)	Score	*Diet (a+b)*
**1**-several times per day	3	0	**1**- low	10	**Non-Smoking**	0	Female	>10	5	+
**2**-(almost) each day	2	0	**2**- moderate	5	**Previous Smoking**	5		<=10	0	*Physical Activity (a+b)*
**3**-several times per week	2	1	**3**- intense	0	**Current Smoking**	10	Male	>20	5	+
**4**-once per week	1	1	Quartile (MET-min/week) (b)	Score	Quartile (pack-years) (b)	Score		<=20	0	*Smoking (a+b)*
**5**-2–3 times per month	1	2	**1th** (<=1977)	10	**1th** (<=3.9208)	0				+
**6**-once or less a month	0	2	**2nd - 3rd.** (1978-6611)	5	**2nd - 3rd.** (3.9209 – 24.5279)	5				*Alcohol (a+b)*
**7**-rather never	0	3	**4th** (>=6612)	0	**4th** (>=24.4280)	10				
	including: meat incl. chicken; cold cuts/ sausages; roasted/panfried potatoproducts; barbecue; smoked products; preped meals; fast food e.g hamburger, Doener, Bratwurst, Currywurst; sugar sweetened beverages;excluding sweets, chips, butter, cake -> was significant negative correlated with BMI	Fish; fresh/frozen vegetables;canned vegetables; raw vegetables; Whole Grain products; Water consumption; exluding: fruits, grains/corn, pasta/rice -> sign. positive correlated with BMI								

Supplemental Table 2 - Study Characteristics of the subgroups
N=4107 (total cohort)

	**Healthy Lifestyle (LS≤5th pct)**		**Unhealthy Lifestyle (LS≥95th pct)**		***P*-Values (LS 5th pct vs. LS 95th pct)**		***P*-Values (Discovery vs.Validation cohort)**	
	Discovery cohort	Validation cohort	Discovery cohort	Validation cohort	Discovery cohort	Validation cohort	LS 5th pct	LS 95th pct
**N**	48	113	50	100				
**Sex (N: female/male)**	36/12	94/19	18/32	35/65	**$<1x10^{-3}$**	**$<1x10^{-11}$**	0.28	0.91
**Age (years)**	57.6 ± 6.8	57.7 ± 13.3	58.3 ± 5.5	55 ± 10.5	0.56	0.11	0.23	**0.01**
**BMI (kg/m²)**	28.48 ± 6.21	27.01 ± 6.13	28.9 ± 6.69	27.22 ± 6.29	0.75	0.81	0.31	0.13
**BMI category (N: lean/obese/overweight)**	23/-/25	70/-/43	25/-/25	61/-/39	0.99	0.99	0.16	0.2
**WHR**	0.89 ± 0.08	0.88 ± 0.07	0.98 ± 0.09	0.96 ± 0.1	**$<1x10^{-6}$**	**$<1x10^{-9}$**	0.82	0.36
**Waist circumference (cm)**	94.72 ± 15.43	92.31 ± 14.6	103.66 ± 17.28	99.35 ± 17.87	**<0.01**	**<0.01**	0.47	0.14
**Fasting plasma glucose (mmol/l)**	5.60 ± 0.93	5.58 ± 0.86	5.74 ± 0.79	5.62 ± 0.79	0.42	0.69	0.98	0.29
**Fasting plasma insulin (pmol/l)**	64.98 ± 45.56	61.13 ± 44.6	75.54 ± 46.28	67.31 ± 45.83	0.26	0.32	0.86	0.25
**Plasma LDL (mmol/l)**	3.30 ± 0.88	3.24 ± 0.89	3.72 ± 0.94	3.5 ± 0.95	**<0.05**	**<0.05**	0.97	0.17
**Plasma HDL (mmol/l)**	1.72 ± 0.41	1.8 ± 0.4	1.51 ± 0.39	1.48 ± 0.39	**<0.05**	**<1x10-7**	0.23	0.65
**Plasma Apolipoprotein A1 (g/l)**	1.75 ± 0.26	1.78 ± 0.26	1.65 ± 0.26	1.62 ± 0.27	**<0.05**	**$<1x10^{-4}$**	0.38	0.56
**Plasma Triglycerides (mmol/l)**	1.20 ± 0.66	1.1 ± 0.53	1.64 ± 0.91	1.57 ± 1.01	**<0.01**	**$<1x10^{-4}$**	0.69	0.32
**Diet Score (range: 3-27)**	8.48 ± 2.05	8.5 ± 1.96	14.78 ± 2.99	15.09 ± 3.01	**$<1x10^{-15}$**	**$<1x10^{-15}$**	0.72	0.52
**PA Score (range: 0-20)**	0.63 ± 1.67	0.66 ± 1.7	18 ± 2.86	17.25 ± 3.58	**$<1x10^{-15}$**	**$<1x10^{-15}$**	0.9	0.28
**Smoking Score (range: 0-20)**	0.1 ± 0.72	0.09 ± 0.66	17.5 ± 3.07	17.1 ± 3.03	**$<1x10^{-15}$**	**$<1x10^{-15}$**	0.93	0.39
**Alcohol Score (range 0-5)**	0.1 ± 0.72	0.13 ± 0.81	2.8 ± 2.51	3.05 ± 2.45	**$<1x10^{-9}$**	**$<1x10^{-15}$**	0.81	0.56
**LS (range: 3-66)**	9.31 ± 1.82	9.39 ± 1.56	53.08 ± 3.97	52.49 ± 3.9	**$<1x10^{-15}$**	**$<1x10^{-15}$**	0.89	0.36

Supplemental Table 3 - Self-designed primer sequences

**Primer - F2RL3**	**Id**	**Sequence**	**Nt**	**Tm, ºC**	**%GC**
**PCR**	F1	GGGTTGGGTGTTTATTAGGT	20	59.3	45
**PCR**	R1	AAACAACCCCAAAACCAACAAAAAATCA	28	58.4	32.1
**Sequencing**	S1	GGGGTTGTAGGTTAATGG	18	46.3	50
**Target Polymorphisms**	Position4, Position5, Position6				
**Sequence to Analyze**	GTTGGYGTTG TGGGTGTTGG TTAYGTAGGT ATTTYGGTTG TTTTTTATTA TGTTGTTGAT G				
**Primer- RARA**	**Id**	**Sequence**	**Nt**	**Tm, ºC**	**%GC**
**PCR**	F1	GTGGGGATTTAGTGTGAGAA	20	58.4	45
**PCR**	R1	CCTCCTCCTCTCCAAATTC	19	59.2	52.6
**Sequencing**	S1	GTTTTTTGTTTAGTTTGGATTTTG	24	44.6	25
**Target Polymorphisms**	Position8, Position9				
**Sequence to Analyze**	T TTTTYGTTT TAGTTTYGTT TTTTTTTYGT TTTTTTTTAG TTG				

Supplemental Table 4 - All lifestyle specific Differentially Methylated Regions (DMRs)

Chromosome	Start	End	Strand	Number CpGs	Min. smoothed FDR	Max. difference	Mean difference	UCSC RefGene name
chr2	233283010	233286870	*	15	6.97E-34	0.083336995	0.025381192	*NA*
chr20	57425515	57429025	*	66	3.03E-22	-0.030967824	-0.01054477	*GNAS;GNAS-AS1*
chr5	190822	192650	*	16	5.22E-22	-0.062761084	-0.017532387	*LRRC14B*
chr17	48911141	48913424	*	26	8.70E-22	0.053515215	0.018522989	*WFIKKN2*
chr19	16998493	17001305	*	15	2.99E-21	0.089458236	0.013602103	*F2RL3*
chr5	373378	374252	*	4	5.36E-21	0.193803612	0.045739382	*AHRR*
chr17	73823769	73825227	*	7	1.22E-20	-0.062518007	-0.039423523	*UNC13D*
chr15	90345999	90346923	*	5	1.72E-19	-0.08590341	-0.044563113	*ANPEP*
chr2	21265912	21268152	*	17	5.76E-17	0.04909301	0.026733677	*APOB*
chr6	32117379	32123701	*	94	9.47E-15	0.042685712	0.007264093	*LOC100507547;PPT2;PPT2-EGFL8;PRRT1*
chr6	33047185	33049505	*	21	1.20E-13	0.081605453	0.036098647	*HLA-DPA1;HLA-DPB1*
chr15	75018065	75020202	*	28	1.07E-12	0.065322172	0.011364887	*CYP1A1*
chr11	86510915	86512100	*	10	2.27E-12	0.046269909	0.009142299	*PRSS23*
chr3	194014481	194015171	*	7	3.90E-12	0.050323261	0.033498744	*NA*
chr3	22412124	22414843	*	10	4.49E-12	-0.091510824	-0.035382375	*ZNF385D*
chr2	241975035	241976658	*	8	2.10E-11	-0.105742005	-0.03406702	*SNED1*
chr17	14205803	14208363	*	17	2.27E-11	0.03284975	0.016634777	*HS3ST3B1;MGC12916*
chr7	157405965	157406737	*	6	9.89E-10	-0.058499659	-0.043422181	*PTPRN2*
chr22	51065435	51066924	*	12	1.15E-09	0.073242376	0.011650886	*ARSA*
chr5	143190915	143192588	*	9	1.18E-09	-0.025708177	-0.007025216	*HMHB1*
chr14	74226931	74228393	*	13	1.24E-09	0.028740317	0.006867855	*MIDEAS*
chr15	74724562	74725593	*	3	1.68E-09	0.031079071	0.017398248	*SEMA7A*
chr17	77923971	77925938	*	15	2.44E-09	0.048373823	0.018436176	*TBC1D16*
chr1	209978796	209980186	*	15	3.50E-09	-0.04349004	-0.017225962	*IRF6*
chr12	14720100	14722066	*	16	5.75E-09	0.046861936	0.015256198	*PLBD1*
chr7	27142275	27144302	*	21	1.25E-08	0.077524453	0.020087313	*HOXA2*
chr7	16505094	16506521	*	9	1.25E-08	-0.058714476	-0.029811065	*SOSTDC1*
chr6	29648161	29649084	*	21	1.27E-08	0.070683917	0.046436136	*ZFP57*
chr14	38063480	38065435	*	18	1.32E-08	-0.022699236	-0.003645152	*FOXA1*
chr8	93114951	93116100	*	12	1.99E-08	0.025333641	0.004089686	*RUNX1T1*
chr19	1265302	1266391	*	6	2.15E-08	0.027225825	0.011999164	*CIRBP*
chr11	126172682	126174769	*	22	2.53E-08	0.054425888	0.011486857	*DCPS*
chr9	130660399	130661175	*	6	3.01E-08	0.036103536	0.019426583	*ST6GALNAC6*
chr17	80188598	80190154	*	14	3.60E-08	0.025002933	0.007425859	*SLC16A3*
chr17	201737	203392	*	12	4.44E-08	-0.028894119	-0.012671318	*RPH3AL*
chr5	369969	370479	*	4	4.63E-08	0.043914978	0.018254492	*AHRR*
chr1	92946700	92947961	*	6	7.03E-08	0.086788359	0.034577075	*GFI1*
chr5	127872767	127874587	*	19	1.02E-07	-0.021569918	-0.00777935	*FBN2*
chr6	30411932	30412351	*	4	1.02E-07	-0.04290578	-0.030533181	*NA*
chr6	170553133	170555276	*	9	1.27E-07	-0.051956784	-0.036130603	*NA*
chr11	2906981	2908471	*	29	1.29E-07	0.044554642	0.006571949	*CDKN1C*
chr21	40123882	40124848	*	5	1.53E-07	-0.031761561	-0.003996751	*LINC00114*
chr10	125754300	125755854	*	7	1.54E-07	-0.035141155	-0.019689501	*NA*
chr16	8960833	8962449	*	13	1.63E-07	0.048619909	0.013642313	*CARHSP1*
chr20	642436	645199	*	10	1.87E-07	0.03786731	0.007415128	*SCRT2;SRXN1*
chr8	145027226	145028886	*	13	2.58E-07	0.038450766	0.015913412	*PLEC*
chr4	72119734	72120167	*	3	3.19E-07	-0.055549608	-0.040238087	*SLC4A4*
chr7	71868406	71868494	*	3	3.58E-07	-0.044899983	-0.027707169	*CALN1*
chr19	44487436	44488612	*	13	3.74E-07	-0.0272612	-0.009932575	*ZNF155*
chr15	42787188	42788175	*	16	3.83E-07	-0.023238673	-0.010333341	*SNAP23*
chr17	934534	935884	*	9	3.83E-07	0.064513189	0.028923887	*ABR*
chr17	45949677	45949878	*	5	4.08E-07	0.050310751	0.037408698	*NA*
chr2	202483740	202484583	*	11	4.14E-07	0.0332111	0.017443227	*C2CD6*
chr14	54421010	54422357	*	11	4.31E-07	-0.013493888	-0.005645751	*BMP4*
chr20	55835831	55836676	*	4	4.51E-07	-0.063006443	-0.048500957	*BMP7*
chr13	113097140	113097975	*	6	4.75E-07	-0.034031864	-0.020235025	*NA*
chr2	220298547	220300568	*	16	5.68E-07	0.029972256	0.014405211	*SPEG*
chr3	113160071	113161177	*	14	6.00E-07	0.041317942	0.02060467	*CFAP44*
chr15	90734476	90735422	*	4	6.28E-07	-0.033030384	-0.025183655	*SEMA4B*
chr10	134650068	134650743	*	3	6.29E-07	-0.047021896	-0.023709664	*CFAP46*
chr7	27168962	27171528	*	30	6.33E-07	-0.048468303	-0.014310035	*HOXA3;HOXA4*
chr6	30719807	30720484	*	6	6.75E-07	0.046234775	0.014711105	*NA*
chr1	42384564	42385941	*	6	7.07E-07	-0.05336742	-0.028861179	*HIVEP3*

chr1	236557165	236559675	*	19	7.23E-07	0.036713329	0.011232273	*EDARADD*
chr19	58962093	58963245	*	13	7.95E-07	0.011373885	-0.00033706	*ZNF324B*
chr22	32438947	32439391	*	5	8.14E-07	-0.047100633	-0.03707628	*SLC5A1*
chr4	25865178	25866396	*	9	8.64E-07	0.026591795	0.008681116	*SEL1L3*
chr19	291022	292131	*	10	8.89E-07	-0.042365016	-0.015721467	*PLPP2*
chr1	31845904	31846873	*	11	9.22E-07	-0.019396018	-0.01123283	*FABP3*
chr1	111217406	111218554	*	11	9.22E-07	0.043368153	0.008432665	*KCNA3*
chr3	71803339	71804859	*	13	9.60E-07	0.049593348	0.013082778	*EIF4E3;GPR27*
chr19	2290843	2291872	*	8	1.09E-06	0.072666047	0.029868422	*LINGO3;SPPL2B*
chr6	170557102	170558102	*	6	1.14E-06	-0.072104452	-0.043424206	*NA*
chr12	26110666	26112124	*	14	1.19E-06	-0.048083828	-0.012601935	*RASSF8;RASSF8-AS1*
chr16	67879671	67880355	*	3	1.35E-06	0.02344066	0.007239904	*CENPT;NUTF2*
chr7	45002287	45002980	*	4	1.37E-06	-0.081266148	-0.038542708	*MYO1G*
chr1	44430285	44430921	*	3	1.50E-06	-0.037641367	-0.020745803	*DPH2;IPO13*
chr10	130830901	130832393	*	10	1.53E-06	-0.027897521	-0.003521773	*NA*
chr11	2919689	2921176	*	18	1.60E-06	0.037806294	0.008647813	*SLC22A18;SLC22A18AS*
chr3	27772033	27772805	*	6	1.86E-06	-0.018442432	-0.00791487	*NA*
chr17	21156233	21157499	*	12	2.24E-06	-0.04791479	-0.01683793	*NATD1*
chr13	36048892	36051073	*	17	2.77E-06	0.024323249	0.007549083	*MIR548F5;NBEA*
chr4	163084858	163085697	*	12	2.82E-06	-0.025371373	-0.007312016	*FSTL5*
chr2	70311984	70313833	*	12	2.92E-06	0.043339587	0.015627619	*PCBP1;PCBP1-AS1*
chr2	97524719	97525009	*	5	3.49E-06	0.021514418	0.018195091	*ANKRD39*
chr17	74072957	74074118	*	7	3.92E-06	-0.045261974	-0.018933203	*GALR2;SRP68;ZACN*
chr1	163037784	163039184	*	16	4.03E-06	-0.056170542	-0.017332549	*RGS4*
chr12	54954337	54955143	*	6	4.18E-06	-0.040743491	-0.014663833	*PDE1B*
chr1	169677313	169677921	*	4	4.29E-06	-0.048252248	-0.033327148	*C1orf112;SELL*
chr6	56111812	56113034	*	9	4.68E-06	-0.043500121	-0.003376419	*COL21A1*
chr19	52900469	52901639	*	15	4.69E-06	0.030695394	0.006466295	*ZNF528*
chr16	70837763	70838524	*	3	4.91E-06	-0.060472705	-0.016416531	*VAC14*
chr8	144659492	144661051	*	15	5.23E-06	-0.036680318	-0.016541779	*MROH6;NAPRT*
chr6	29691408	29692995	*	24	5.73E-06	0.042327402	0.0101074	*HLA-F*
chr9	98314632	98316500	*	7	5.79E-06	0.033388563	0.0072153	*NA*
chr13	46424943	46425929	*	10	5.80E-06	-0.02230238	-0.009327744	*SIAH3*
chr1	16553267	16554984	*	10	6.05E-06	0.055033991	0.019310538	*NA*
chr16	68269041	68272111	*	23	6.18E-06	0.032406616	0.010141548	*ESRP2*
chr10	115438155	115439884	*	17	6.24E-06	-0.012915594	-0.001872104	*CASP7*
chr16	73099917	73101264	*	6	6.48E-06	0.036219829	0.019478345	*NA*
chr1	151103642	151105478	*	11	6.64E-06	0.041097211	0.019181781	*SEMA6C*
chr16	89181807	89182492	*	7	6.68E-06	-0.014045662	-0.008518713	*ACSF3*
chr15	23809945	23811572	*	14	6.92E-06	-0.040958957	-0.021947534	*MIR4508;MKRN3*
chr17	19881268	19882222	*	11	7.71E-06	-0.032564313	-0.008843135	*AKAP10*
chr20	19193824	19194649	*	7	7.84E-06	0.044327931	0.014119857	*SLC24A3*
chr5	35229788	35231272	*	13	7.87E-06	-0.033615611	-0.010094942	*PRLR*
chr11	6440065	6441289	*	18	7.95E-06	-0.026093996	-0.006968055	*APBB1*
chr3	52811637	52814643	*	16	7.98E-06	-0.024849035	-0.006264133	*ITIH1*
chr6	30710462	30712373	*	50	8.11E-06	-0.024606529	-0.001516747	*FLOT1;IER3*
chr4	1858231	1858883	*	3	8.51E-06	0.0265421	0.009277285	*LETM1*
chr1	24644596	24646205	*	18	8.99E-06	-0.017414465	-0.006235379	*GRHL3*
chr5	171056823	171057575	*	7	9.24E-06	0.042779917	0.036184758	*NA*
chr4	77723057	77723800	*	6	9.61E-06	0.043653208	0.02407009	*NA*
chr1	2144244	2145655	*	10	1.00E-05	0.035436851	0.012959788	*NA*
chr3	137487770	137489312	*	7	1.05E-05	-0.013035861	-0.00184741	*NA*
chr19	3480363	3480940	*	7	1.23E-05	-0.044442104	-0.029951742	*SMIM24*
chr16	57672401	57673993	*	9	1.26E-05	-0.027151559	-0.010535734	*ADGRG1*
chr1	173174650	173176090	*	8	1.29E-05	0.022815842	-0.005139747	*TNFSF4*
chr8	145637966	145639652	*	9	1.33E-05	-0.034767308	-0.017374327	*CPSF1;SLC39A4*
chr1	221509067	221509763	*	3	1.35E-05	-0.033078868	-0.019248184	*LINC02817*
chr4	18320820	18321251	*	4	1.36E-05	-0.0367123	-0.030052878	*NA*
chr10	8373416	8374526	*	7	1.40E-05	-0.029933056	-0.015724415	*NA*
chr5	150161299	150163018	*	4	1.47E-05	0.039618192	0.01496976	*SMIM3*
chr8	144797929	144799376	*	14	1.48E-05	0.052120714	0.005294952	*MAPK15*
chr3	11643341	11643630	*	5	1.49E-05	-0.031554986	-0.021926198	*VGLL4*
chr15	80351213	80352558	*	17	1.50E-05	-0.033580546	-0.010891271	*ZFAND6*
chr10	90984672	90985062	*	3	1.55E-05	0.056039743	0.049841417	*LIPA*
chr21	46714226	46715205	*	5	1.60E-05	0.043076441	0.029325826	*LINC00205*
chr16	8805422	8807308	*	16	1.64E-05	0.043713634	0.021425684	*ABAT*

chr2	201674914	201676437	*	11	1.64E-05	0.019535191	0.004914506	*BZW1*
chr14	88788343	88789655	*	12	1.67E-05	-0.046389737	-0.01270038	*KCNK10*
chr16	57519987	57521330	*	11	1.67E-05	-0.030516643	-0.010410076	*DOK4*
chr1	17766238	17767282	*	6	1.69E-05	0.038734568	0.003420664	*RCC2*
chr11	122451407	122452135	*	3	1.79E-05	-0.019519126	-0.018405058	*NA*
chr4	86395989	86396765	*	14	1.80E-05	-0.0456355	-0.010901102	*ARHGAP24*
chr14	103415458	103416389	*	6	1.86E-05	-0.033035588	-0.01200078	*CDC42BPB*
chr16	85362963	85363209	*	4	1.89E-05	0.050747768	0.036293171	*NA*
chr12	25403102	25405280	*	27	1.89E-05	-0.036273988	-0.006882384	*KRAS*
chr2	66666267	66668012	*	17	1.90E-05	-0.013062251	-0.004328522	*MEIS1*
chr3	187387189	187388737	*	15	1.90E-05	0.032514942	-0.003041652	*SST*
chr18	31802071	31803067	*	10	1.90E-05	-0.010405455	-0.003367162	*NOL4*
chr6	33770212	33772366	*	10	1.96E-05	0.028187791	0.017570121	*MLN*
chr2	74728623	74730047	*	10	2.14E-05	0.03219169	0.014271859	*LBX2;LBX2-AS1*
chr13	51417469	51418614	*	12	2.21E-05	0.021033075	0.011508784	*DLEU7;DLEU7-AS1*
chr6	144385609	144387124	*	19	2.25E-05	0.037719689	0.007680264	*PLAGL1*
chr19	45909124	45910448	*	16	2.28E-05	-0.019712252	-0.003623939	*POLR1G;PPP1R13L*
chr6	138893111	138893718	*	6	2.31E-05	-0.055731808	-0.019135165	*NHSL1*
chr2	225265564	225266960	*	12	2.33E-05	-0.034319444	-0.007677548	*FAM124B*
chr11	7597402	7598673	*	11	2.33E-05	-0.046259743	-0.015330363	*PPFIBP2*
chr13	111226725	111228197	*	7	2.41E-05	0.026988571	0.006032595	*NA*
chr3	29377160	29377980	*	3	2.42E-05	-0.072396235	-0.044645175	*RBMS3*
chr2	163695111	163696187	*	9	2.42E-05	-0.034103147	-0.012627843	*KCNH7*
chr13	67802655	67805065	*	18	2.47E-05	-0.041856974	-0.011136684	*PCDH9*
chr16	85122558	85123778	*	4	2.49E-05	0.023183388	0.007445622	*KIAA0513*
chr11	123524938	123526191	*	10	2.49E-05	-0.027971925	-0.01005003	*SCN3B*
chr4	78978133	78979207	*	12	2.50E-05	-0.013011842	-0.002537353	*FRAS1*
chr20	42142005	42143502	*	29	2.51E-05	-0.023377772	-0.005135601	*L3MBTL1*
chr17	76354621	76355674	*	7	2.52E-05	0.026202873	0.014668819	*SOCS3*
chr15	26108391	26109614	*	29	2.68E-05	0.030597334	0.002474212	*ATP10A*
chr14	77247777	77248049	*	5	2.76E-05	0.057960459	0.024964563	*VASH1*
chr1	206223701	206224554	*	7	2.80E-05	0.034586699	0.002330489	*AVPR1B*
chr2	5831370	5834638	*	13	2.87E-05	-0.017964809	-0.006123935	*SOX11*
chr4	1684739	1686288	*	9	3.03E-05	0.021331166	-0.001925464	*FAM53A*
chr7	1882776	1883760	*	8	3.04E-05	0.049993213	0.026493461	*MAD1L1;MIR4655*
chr7	95025194	95026937	*	24	3.15E-05	-0.036935728	-0.01037563	*PON1;PON3*
chr1	206306094	206307060	*	5	3.35E-05	-0.02217631	-0.011733368	*NA*
chr2	239139911	239140910	*	9	3.37E-05	-0.041315995	-0.019607737	*LINC02610;TARDBPP3*
chr5	28809037	28810645	*	9	3.38E-05	0.029227748	0.018034286	*NA*
chr20	61446962	61447929	*	29	3.41E-05	-0.043058538	-0.008813399	*COL9A3*
chr6	31124978	31126284	*	18	3.46E-05	0.034615081	0.004886477	*CCHCR1;TCF19*
chr5	154998	155199	*	4	3.72E-05	0.02708318	0.022363964	*PLEKHG4B*
chr6	32846924	32847845	*	15	3.84E-05	0.04295651	0.021416406	*NA*
chr1	150254280	150255880	*	14	3.90E-05	0.023440097	0.004242032	*CIART*
chr14	81425912	81426577	*	5	4.02E-05	-0.042071672	-0.020653769	*CEP128;TSHR*
chr5	368394	368843	*	3	4.02E-05	0.037993561	0.024263206	*AHRR*
chr16	75150456	75150880	*	8	4.07E-05	0.02922422	0.024855109	*LDHD*
chr19	51774264	51775111	*	7	4.35E-05	0.047437003	0.027961707	*NA*
chr1	2344426	2346088	*	18	4.42E-05	0.037443726	0.009967219	*PEX10*
chr14	105858487	105859242	*	3	4.49E-05	0.026954194	0.013512786	*PACS2*
chr4	81117853	81119473	*	13	4.63E-05	0.033010762	0.016556667	*PRDM8*
chr4	2469589	2471043	*	16	4.78E-05	0.030932076	0.003766886	*RNF4*
chr1	161128567	161130207	*	18	4.89E-05	0.016539295	0.002581331	*UFC1;USP21*
chr3	147140880	147142415	*	13	5.09E-05	-0.036216608	-0.006263032	*NA*
chr21	40176506	40177189	*	8	5.09E-05	0.061425153	0.011993308	*ETS2*
chr8	65289139	65290484	*	9	5.20E-05	-0.01771503	-0.003910066	*MIR124-2;MIR124-2HG*
chr8	125740425	125741566	*	13	5.22E-05	0.035108003	0.006718675	*MTSS1*
chr20	62084549	62084833	*	3	5.29E-05	-0.044737322	-0.036569781	*KCNQ2*
chr9	137978190	137979399	*	9	5.65E-05	-0.047674747	-0.018412699	*OLFM1*
chr17	79372242	79374741	*	16	5.70E-05	0.03084311	0.005165538	*BAHCC1;MIR4740*
chr5	93953562	93955080	*	22	5.71E-05	0.023735536	-1.22E-05	*KIAA0825;SLF1*
chr14	105647194	105648697	*	9	5.73E-05	-0.046422116	-0.006467101	*NUDT14*
chr9	130639739	130640786	*	12	5.76E-05	0.023422753	0.008904248	*AK1*
chr21	46492095	46494274	*	9	5.82E-05	0.029405092	0.015206772	*ADARB1;SSR4P1*
chr1	167486978	167488503	*	8	6.17E-05	-0.028300312	-0.01832827	*CD247*
chr19	56988398	56990070	*	11	6.26E-05	-0.01931509	-0.008543938	*ZNF667;ZNF667-AS1*

chr6	133561368	133562776	*	36	6.31E-05	-0.022889036	-0.007741311	*EYA4*
chr4	184642625	184642971	*	4	6.52E-05	-0.045481251	-0.041666452	*NA*
chr19	10735474	10736448	*	10	6.69E-05	0.059228868	0.02115741	*SLC44A2*
chr1	151030968	151031323	*	6	6.70E-05	0.01365323	0.003303866	*CDC42SE1;MLLT11*
chr1	244093755	244096161	*	7	6.83E-05	0.042486832	0.021756937	*LINC02774*
chr19	39465821	39467435	*	15	6.98E-05	0.040090757	0.0133187	*FBXO17*
chr2	46465	47716	*	15	7.06E-05	-0.031984981	-0.010287802	*FAM110C*
chr7	101555980	101556938	*	8	7.07E-05	-0.028785504	-0.012041203	*CUX1*
chr5	151150029	151151000	*	7	7.11E-05	0.034637779	0.008132194	*G3BP1;LOC100652758*
chr15	86315115	86315211	*	3	7.15E-05	-0.025163753	-0.014789066	*KLHL25;MIR1276*
chr3	46617183	46619131	*	7	7.31E-05	0.041356874	0.023314871	*LRRC2;TDGF1*
chr14	101348158	101350872	*	26	7.41E-05	0.031945843	0.007365095	*MIR127;MIR136;MIR432;MIR433;RTL1*
chr16	2770211	2771425	*	10	7.49E-05	-0.019911461	0.003327109	*PRSS27*
chr5	138610977	138611766	*	5	7.51E-05	0.022103712	0.017628942	*MATR3;SNHG4;SNORA74A*
chr5	320888	321681	*	3	7.87E-05	0.034529092	0.018014512	*AHRR*
chr17	7164634	7166281	*	13	7.97E-05	0.031716733	0.00588697	*CLDN7*
chr10	129947756	129948776	*	5	8.03E-05	0.037336374	0.011429478	*NA*
chr9	127562983	127563860	*	4	8.57E-05	-0.031605876	-0.018572537	*OLFML2A*
chr12	52207897	52208899	*	4	8.72E-05	0.050652287	0.024880416	*NA*
chr18	46064542	46066011	*	12	8.77E-05	0.031447703	0.002393375	*CTIF*
chr1	241587126	241588176	*	9	9.09E-05	-0.029347452	-0.008692447	*NA*
chr7	1068125	1068617	*	3	9.09E-05	0.029737783	0.011852922	*C7orf50*
chr5	2756361	2757802	*	7	9.21E-05	-0.023308448	-0.006578694	*IRX2*
chr10	126826304	126827067	*	5	9.49E-05	0.0257496	0.012475545	*CTBP2*
chr10	134818842	134819662	*	6	9.58E-05	-0.022476133	-0.01029761	*NA*
chr19	51893804	51894617	*	7	0.000100421	-0.031191834	-0.008380595	*C19orf84;LIM2*
chr1	870791	871546	*	7	0.000105272	-0.04123203	-0.03704767	*SAMD11*
chr1	41156520	41157828	*	19	0.000105651	0.029825582	0.007139181	*NFYC;NFYC-AS1*
chr1	17914070	17914803	*	4	0.000108715	0.026048067	0.01395124	*ARHGEF10L*
chr4	81187906	81189164	*	6	0.000109505	0.021557821	-0.003253121	*FGF5*
chr1	854766	855649	*	12	0.000110973	0.028847375	0.010133205	*LINC02593;SAMD11*
chr16	86852700	86853235	*	3	0.000111949	0.03334279	0.025360569	*NA*
chr7	12443146	12444115	*	13	0.000112584	-0.040342917	-0.013316657	*VWDE*
chr10	32216031	32217076	*	8	0.000112584	0.040267835	0.017232332	*ARHGAP12*
chr7	90224886	90226284	*	15	0.000114911	-0.014892696	-0.006255701	*CDK14*
chr15	90357202	90357992	*	3	0.000114911	0.032193825	0.011920858	*ANPEP*
chr1	7913081	7914017	*	8	0.000115433	0.041360817	0.015396456	*UTS2*
chr16	20084851	20085897	*	10	0.00011585	-0.028330612	-0.011299935	*GPR139*
chr16	19589790	19590554	*	5	0.00011585	-0.031829778	-0.00964568	*VPS35L*
chr20	57582292	57583709	*	23	0.000116893	-0.02522841	-0.003431385	*CTSZ*
chr10	135190860	135192232	*	23	0.000118505	0.019245502	0.004220549	*ECHS1;PAOX*
chr3	39448643	39449544	*	7	0.000118505	0.020986467	0.009770563	*RPSA;SNORA6;SNORA62*
chr16	86591940	86592652	*	5	0.000120603	0.030177701	0.017433912	*MTHFSD*
chr19	17932512	17933786	*	4	0.000121474	0.027062086	0.01259964	*INSL3*
chr20	13975439	13977219	*	19	0.00012181	-0.034659212	-0.008309301	*MACROD2;SEL1L2*
chr1	153232348	153234037	*	6	0.000122165	-0.024683895	-0.011210561	*LORICRIN*
chr14	75535155	75537231	*	15	0.000122766	0.032783628	0.006313922	*ACYP1;ZC2HC1C*
chr7	106685033	106686041	*	4	0.000124229	0.022712205	0.004321165	*PRKAR2B*
chr1	202310824	202311492	*	9	0.000124539	-0.013354766	-0.009005555	*UBE2T*
chr17	9549496	9550545	*	8	0.00012745	0.029579632	0.02214183	*USP43*
chr11	134257744	134258597	*	7	0.000127497	0.032022814	0.013741486	*B3GAT1*
chr10	134597884	134601490	*	36	0.000128749	-0.033847734	-0.009496988	*NKX6-2*
chr20	60294656	60295700	*	6	0.000133006	-0.036503079	-0.021734259	*CDH4*
chr8	626528	627809	*	8	0.000133136	-0.036811151	-0.001020889	*ERICH1*
chr6	105584149	105585490	*	14	0.00013565	0.022287495	0.002278475	*BVES;BVES-AS1*
chr10	134062078	134062614	*	4	0.000136013	-0.065198707	-0.025383616	*STK32C*
chr15	68522181	68523155	*	9	0.000137223	0.031812426	0.008083537	*CLN6*
chr21	45285836	45287137	*	5	0.0001381	-0.029071257	0.005015512	*AGPAT3*
chr5	66253919	66255772	*	9	0.000138113	-0.0240886	-0.013670037	*MAST4*
chr22	22734186	22734960	*	4	0.000139533	0.030406608	0.020591898	*NA*
chr5	114937535	114938640	*	20	0.000142818	-0.026538675	-0.007442297	*TICAM2;TMED7-TICAM2*
chr1	120255318	120256112	*	5	0.000144926	0.034857844	0.017891977	*PHGDH*
chr6	31238388	31239411	*	9	0.000146884	0.083070938	0.027268197	*HLA-B;HLA-C*
chr18	5578880	5579883	*	5	0.000152835	0.033191671	0.014621503	*EPB41L3*
chr6	33129024	33134325	*	58	0.000154785	0.033770907	0.005219513	*COL11A2*
chr1	112045871	112047187	*	11	0.000155149	-0.020639596	-0.004721138	*ADORA3*

chr12	116985025	116986371	*	6	0.000155474	0.022862989	-0.004911984	*NA*
chr6	31695599	31697280	*	31	0.000155986	0.029841824	0.0078678	*DDAH2*
chr9	128651435	128652652	*	5	0.000156635	-0.03867689	-0.017219436	*PBX3*
chr8	53477881	53479270	*	10	0.000157831	-0.017567486	-0.004378815	*ALKAL1*
chr11	120195717	120197726	*	11	0.000160217	-0.04392879	-0.007934281	*TLCD5*
chr22	43043757	43044788	*	5	0.000165353	0.030123419	0.010845309	*CYB5R3*
chr4	154605468	154606177	*	3	0.000167064	-0.010125838	-0.002555729	*TLR2*
chr20	37359533	37359998	*	5	0.000168468	0.014998284	-0.000250261	*NA*
chr12	96252066	96253460	*	17	0.00017332	0.025025187	0.003775394	*SNRPF*
chr11	110296837	110297121	*	3	0.000173999	-0.026077469	-0.019348452	*FDX1*
chr11	105479967	105481702	*	21	0.000175034	-0.03284956	-0.00537266	*GRIA4*
chr17	39184318	39184887	*	4	0.000175861	-0.02114592	-0.003619911	*KRTAP1-5*
chr16	57728173	57729107	*	7	0.000180936	-0.016525388	-0.005604143	*DRC7*
chr1	4193126	4194412	*	5	0.000182821	0.045352991	0.011953529	*NA*
chr13	20160420	20161246	*	5	0.000189313	-0.033235785	-0.017244023	*NA*
chr7	47621223	47622718	*	13	0.000190007	-0.032689411	-0.003829697	*TNS3*
chr1	25256369	25258332	*	28	0.000190448	0.028004326	0.005341543	*RUNX3*
chr3	157217187	157218259	*	13	0.000191495	-0.030111865	-0.005979312	*VEPH1*
chr3	190335248	190336306	*	3	0.000193905	0.050043126	0.022612063	*IL1RAP*
chr15	54270514	54271167	*	6	0.00019655	-0.011931343	-0.00873434	*NA*
chr11	62066533	62067691	*	6	0.000196965	-0.036611968	-0.008624026	*SCGB1D4*
chr6	27635558	27638391	*	10	0.000198319	-0.036731596	-0.015235167	*NA*
chr12	52416192	52417019	*	8	0.000200914	0.026986798	0.010154109	*NR4A1*
chr18	3593461	3595113	*	12	0.000201864	-0.018586938	-0.00505176	*DLGAP1;DLGAP1-AS1*
chr17	41607535	41608691	*	10	0.000202573	-0.017493844	-0.008608516	*ETV4*
chr7	154684051	154685308	*	11	0.000203417	-0.061706642	-0.016884281	*DPP6*
chr4	646853	648752	*	10	0.000205003	0.023847202	0.004177887	*PDE6B*
chr1	91190891	91192803	*	17	0.00020892	0.040465997	0.005216748	*NA*
chr6	29795501	29796614	*	14	0.000209895	0.035645892	0.011489014	*HLA-G;HLA-H*
chr5	55007918	55008611	*	15	0.000212093	-0.022782406	-0.00139176	*SLC38A9*
chr18	23713407	23714084	*	12	0.000216217	0.034665837	0.015831814	*PSMA8*
chr19	294042	295123	*	3	0.0002174	-0.059607734	-0.020105226	*PLPP2*
chr16	66399746	66400599	*	10	0.000217509	-0.016007606	-0.005211816	*CDH5*
chr2	198649443	198650603	*	13	0.000218356	0.032360987	0.017045096	*BOLL*
chr6	32764865	32765402	*	14	0.000218996	-0.019176615	-0.004706811	*NA*
chr19	2545986	2547067	*	5	0.000220699	0.039449521	0.025499378	*GNG7*
chr21	47318899	47319633	*	3	0.000221096	0.030652825	0.02060723	*PCBP3*
chr9	131083986	131085530	*	17	0.000221107	0.01432994	0.000903267	*COQ4;TRUB2*
chr7	128494060	128494696	*	7	0.00022114	0.020399256	0.010122935	*FLNC*
chr7	11871535	11872745	*	10	0.000221437	-0.023530295	-0.011878523	*THSD7A*
chr8	144098507	144099991	*	10	0.000222911	0.030398182	0.007590092	*LY6E;LY6E-DT*
chr3	141144022	141145231	*	5	0.000224158	0.024502554	0.011413504	*ZBTB38*
chr14	104190678	104191347	*	5	0.000225457	-0.029846017	-0.015167134	*ZFYVE21*
chr10	118368264	118368986	*	3	0.000228196	0.052951929	0.023882764	*PNLIPRP1*
chr2	239628381	239629498	*	8	0.00024027	-0.03387592	-0.004495198	*NA*
chr2	87018382	87019612	*	10	0.000241695	0.032739607	0.010980554	*CD8A;RMND5A*
chr1	3527627	3528682	*	12	0.000246624	-0.038699697	-0.010274954	*MEGF6*
chr12	97300410	97301661	*	18	0.000254566	-0.017893339	-0.002022227	*NEDD1*
chr14	76393953	76395305	*	3	0.000256606	-0.024986486	-0.015388443	*TTLL5*
chr2	234215396	234216562	*	12	0.000256985	-0.024001473	-0.001558627	*SAG*
chr5	154230173	154230438	*	5	0.000257683	-0.020356979	-0.016514345	*FAXDC2*
chr1	9488706	9489434	*	6	0.000258984	-0.032119434	-0.003216566	*NA*
chr7	99954042	99955101	*	6	0.000260024	-0.024503196	-0.005375218	*PILRB*
chr10	134150119	134150760	*	11	0.00026569	-0.034855507	-0.022502814	*LRRC27;STK32C*
chr3	128690194	128690824	*	3	0.000270516	-0.033059636	-0.022350841	*CFAP92*
chr3	57456170	57456332	*	3	0.000276796	-0.032164137	-0.009331308	*DNAH12*
chr7	99195655	99196468	*	5	0.000279382	0.037262542	0.027025695	*TMEM225B*
chr7	48129797	48130197	*	5	0.000280212	0.03401411	0.018768199	*UPP1*
chr3	53700141	53700263	*	3	0.000280372	-0.058166831	-0.052935571	*CACNA1D*
chr1	15497910	15498551	*	8	0.000280819	-0.029989749	-0.0095017	*TMEM51*
chr12	50101027	50101564	*	6	0.000286796	-0.021645943	-0.007760641	*FMNL3*
chr10	130726406	130726701	*	3	0.000287833	-0.066141442	-0.052150296	*NA*
chr12	8380001	8380472	*	5	0.000300152	-0.050422973	-0.027899437	*FAM90A1*
chr20	57330819	57331560	*	4	0.00030061	0.029926299	0.010921137	*NA*
chr13	76334000	76334866	*	7	0.000307843	-0.022534072	-0.014092738	*LMO7*
chr5	78907212	78908220	*	9	0.000309876	0.037700715	0.002836381	*TENT2*

chr11	111168594	111169865	*	16	0.000311045	0.029982537	0.008172705	*COLCA1;COLCA2*
chr22	28838631	28839352	*	6	0.000311181	-0.025119502	-0.012490585	*TTC28*
chr21	44817305	44817925	*	4	0.000311643	0.023324554	0.01138908	*NA*
chr4	66535145	66536772	*	22	0.000319586	-0.020787452	-0.004169205	*EPHA5;EPHA5-AS1*
chr10	32734072	32735380	*	12	0.000320134	-0.034667622	-0.005100663	*NA*
chr5	58652602	58652948	*	5	0.00032025	-0.022292049	-0.014118523	*PDE4D*
chr6	41168185	41169391	*	8	0.000320254	-0.020659643	-0.009009859	*TREML2*
chr11	14664598	14665355	*	9	0.000322215	0.017409633	0.004557551	*PDE3B;PSMA1*
chr19	39522548	39523626	*	10	0.000324175	0.057402825	0.016377779	*FBXO27*
chr17	79503292	79504745	*	7	0.000327711	-0.048760393	-0.01820661	*FSCN2*
chr6	30227729	30228431	*	21	0.000332709	0.028597343	0.009258622	*HCG17;HLA-L*
chr14	45722289	45723370	*	13	0.000335526	0.039674063	0.010405464	*MIS18BP1*
chr20	36887672	36889728	*	14	0.00033646	0.047498146	0.005565349	*KIAA1755*
chr10	45494806	45496315	*	12	0.00033646	-0.027333317	-0.007106922	*ZNF22;ZNF22-AS1*
chr3	52351355	52351963	*	6	0.00033646	-0.036266581	-0.026590054	*DNAH1*
chr8	22551655	22552961	*	5	0.000337975	0.027738985	0.004901147	*EGR3*
chr1	19600141	19601069	*	8	0.000341046	-0.06660937	-0.024798758	*AKR7L*
chr12	22590017	22590199	*	3	0.000344944	0.033143664	0.026765701	*NA*
chr22	17955450	17956641	*	9	0.00035102	-0.029810091	-0.013405531	*CECR2*
chr14	106320669	106322986	*	16	0.000360271	0.033299459	0.008944743	*NA*
chr1	19181040	19181419	*	5	0.000364622	-0.058926543	-0.023955871	*TAS1R2*
chr5	122758260	122759733	*	15	0.000370189	-0.009787065	-0.001935496	*CEP120*
chr11	124737554	124738157	*	3	0.000370986	-0.01902558	-0.010002799	*ROBO3*
chr2	5865442	5866470	*	6	0.000374238	-0.016423147	-0.006030566	*NA*
chr11	67397595	67399029	*	8	0.000382064	0.024149859	0.004114541	*NUDT8;TBX10*
chr1	228331879	228332502	*	6	0.000385265	-0.02300722	-0.002332885	*GJC2;GUK1*
chr18	61088726	61090281	*	14	0.00038615	0.03969929	0.002759326	*VPS4B*
chr15	90292730	90295301	*	15	0.000388913	0.017578186	0.006674648	*MESP1*
chr16	31020735	31022521	*	11	0.000390745	-0.036779745	-0.011270121	*STX1B*
chr20	13200685	13201551	*	20	0.000391992	-0.030584912	-0.009123201	*ISM1*
chr9	139903316	139904151	*	5	0.000391992	0.036193655	0.017082889	*ABCA2*
chr12	1714851	1715055	*	3	0.00039425	-0.014122334	-0.010087272	*WNT5B*
chr18	60051870	60052464	*	5	0.000395505	0.046574475	0.029627717	*TNFRSF11A*
chr4	15780238	15781202	*	7	0.000398091	0.01207384	0.00108881	*CD38*
chr19	15342548	15343425	*	11	0.000404385	0.039119938	0.003066686	*EPHX3*
chr2	30144152	30145152	*	11	0.000404786	-0.021706957	-0.007914213	*ALK*
chr1	76261602	76262984	*	11	0.000408907	0.021269035	0.011926125	*MSH4*
chr17	78560478	78560916	*	3	0.000410293	-0.036362411	-0.025141258	*RPTOR*
chr2	70932281	70933416	*	3	0.000411003	0.023046431	0.01777614	*ADD2*
chr3	184032711	184034132	*	12	0.000412989	0.024601017	0.005632364	*EIF4G1*
chr2	31043419	31044164	*	4	0.000416625	0.018948289	0.006411771	*NA*
chr12	56324837	56325736	*	8	0.000419417	0.017118895	0.001686382	*DGKA;PYM1*
chr2	26205095	26206226	*	10	0.000420039	0.03122348	0.002826659	*KIF3C*
chr5	601475	602552	*	7	0.000420039	0.037975813	0.018322595	*LOC100996325*
chr19	14016603	14017658	*	17	0.000424039	0.047036286	0.006884611	*BRME1;CC2D1A*
chr10	1233675	1234983	*	6	0.000424459	-0.019344463	-0.013603738	*ADARB2*
chr17	75789279	75790091	*	6	0.000425602	-0.062730633	-0.027130191	*NA*
chr17	1395371	1396298	*	8	0.000427454	0.022895041	0.012758271	*MYO1C*
chr11	118965121	118966503	*	11	0.000432059	-0.007526761	0.000393608	*H2AX*
chr12	133464665	133465592	*	11	0.000435526	0.018522323	0.003241375	*CHFR*
chr17	25798180	25799447	*	11	0.000436561	0.03118523	0.009225826	*KSR1*
chr16	1143566	1144264	*	4	0.000436561	-0.031168111	-0.025922075	*C1QTNF8*
chr2	79738545	79739278	*	6	0.000442101	-0.038082361	-0.019705643	*CTNNA2*
chr3	139061956	139063360	*	15	0.000446052	0.02870503	0.001779957	*MRPS22*
chr15	60770518	60771505	*	7	0.000447597	0.010950378	0.004330224	*ICE2*
chr19	7561739	7562555	*	7	0.000447862	-0.026405691	-0.004542154	*TEX45*
chr19	14591033	14591345	*	5	0.000447862	0.060416895	0.022290129	*GIPC1;PTGER1*
chr12	57937334	57937454	*	3	0.000453139	-0.030703708	-0.020096767	*DCTN2*
chr12	49730687	49731991	*	12	0.000456974	-0.024560412	-0.004428629	*C1QL4*
chr11	89867385	89868104	*	14	0.000460833	-0.022602513	-0.012644877	*NAALAD2*
chr11	61322107	61324205	*	10	0.000465828	-0.025875239	-0.011058272	*SYT7*
chr10	134821210	134822001	*	4	0.000466482	0.037029778	0.019628198	*NA*
chr4	89978251	89978732	*	7	0.000469395	-0.019984021	-0.012012231	*FAM13A*
chr2	51254102	51255480	*	8	0.000470423	0.039452789	0.016055119	*NRXN1*
chr6	3054085	3054884	*	5	0.00047401	0.036223782	0.009189783	*NA*
chr19	34174345	34175554	*	20	0.000478519	0.031137954	-0.003553191	*CHST8*

chr6	12749567	12750392	*	5	0.000478547	-0.017271339	-0.009304402	*PHACTR1*
chr5	399012	400201	*	5	0.000482098	0.049126206	0.007301223	*AHRR*
chr3	170136159	170137240	*	12	0.00048311	-0.029323869	-0.008881424	*CLDN11*
chr1	166135963	166136996	*	13	0.000484411	-0.018735143	-0.006653587	*FAM78B*
chr4	143766602	143768275	*	13	0.000502104	-0.01366675	-0.002303089	*INPP4B*
chr17	27346732	27347260	*	6	0.000502104	0.021015129	0.012053174	*NA*
chr6	39196071	39197655	*	10	0.00050486	-0.060702873	-0.007600078	*KCNK5*
chr2	69968414	69969591	*	14	0.00050489	-0.024876661	-0.004675091	*ANXA4*
chr6	2999095	3000377	*	14	0.000505326	-0.04187992	-0.009924224	*NQO2*
chr19	18873037	18873268	*	3	0.00051021	-0.037410649	-0.019601823	*CRTC1*
chr5	137773712	137775041	*	13	0.00051318	-0.033355837	-0.008444726	*REEP2*
chr5	140561239	140562562	*	9	0.000513358	-0.041389335	-0.019206295	*PCDHB16*
chr11	76812596	76813364	*	5	0.000513358	0.015513431	-0.000701572	*CAPN5;OMP*
chr12	131715734	131715773	*	3	0.000514574	-0.029639655	-0.013332948	*NA*
chr8	114448659	114450231	*	13	0.000514856	-0.022454211	-0.003267874	*CSMD3*
chr19	2774149	2775228	*	7	0.000518356	0.019781118	0.009786156	*SGTA*
chr10	80872926	80873797	*	3	0.000519548	0.02154592	0.016346406	*ZMIZ1*
chr19	54515169	54515811	*	4	0.000521357	0.038556613	0.028957583	*CACNG6*
chr9	137966584	137967652	*	12	0.000536331	0.040007977	-0.005899971	*OLFM1*
chr15	96909816	96911696	*	8	0.000536331	-0.018430334	0.004383319	*NA*
chr19	36119199	36120543	*	13	0.000546488	-0.034365574	-0.002375933	*RBM42*
chr1	156163116	156164745	*	14	0.000551725	0.030079344	0.005029138	*SLC25A44*
chr17	31254544	31255635	*	12	0.000551855	-0.019503513	-0.006941871	*TMEM98*
chr5	59188112	59188920	*	3	0.000556229	-0.018609487	-0.011957144	*PDE4D*
chr10	42970351	42971732	*	11	0.000559462	0.046086304	0.014766421	*LINC00839*
chr3	147122315	147127662	*	48	0.000561928	0.024896442	-0.002428075	*ZIC1;ZIC4*
chr6	24489671	24491098	*	7	0.000563536	-0.02212257	-0.002654819	*ALDH5A1;GPLD1*
chr7	19183280	19184555	*	8	0.000564726	-0.015069046	-0.005600899	*FERD3L*
chr1	153721986	153722548	*	4	0.000578235	-0.027972017	-0.004813206	*INTS3*
chr6	23177275	23178052	*	5	0.000579664	-0.033928096	-0.015447829	*NA*
chr19	4769001	4769688	*	13	0.000581882	-0.033152191	-0.006948943	*MIR7-3;MIR7-3HG*
chr10	45359611	45360313	*	5	0.000585616	-0.043924993	-0.023658541	*TMEM72-AS1*
chr9	79520919	79521848	*	9	0.00058595	-0.015066451	-0.005738286	*PRUNE2*
chr19	6534760	6535078	*	5	0.00058806	0.032765919	0.023285074	*TNFSF9*
chr15	91414499	91416118	*	14	0.000589333	0.021714884	0.003058598	*FURIN*
chr16	67927351	67928026	*	3	0.000591276	0.028775701	0.008198654	*PSKH1*
chr3	79067596	79069442	*	15	0.000591688	-0.010080206	-0.003202526	*ROBO1*
chr2	43385231	43386412	*	3	0.000594763	-0.020551285	-0.012381288	*NA*
chr1	204485075	204486394	*	12	0.000602934	0.02789188	0.00478865	*MDM4*
chr11	22648074	22648618	*	3	0.000606323	-0.012292459	-0.006032088	*FANCF*
chr5	87439075	87441969	*	13	0.000607218	-0.031794017	-0.011509158	*NA*
chr7	100660212	100660957	*	7	0.000607218	-0.021430735	-0.007445517	*MUC12;MUC17*
chr4	40631741	40633572	*	14	0.000608679	-0.018608373	-0.00453237	*RBM47*
chr12	104443742	104444435	*	11	0.000611341	-0.022854032	-0.009642876	*GLT8D2*
chr16	11680891	11681807	*	10	0.000613977	0.03851188	0.003712217	*LITAF*
chr11	98890677	98892283	*	15	0.00063029	-0.016930313	-0.003347439	*CNTN5*
chr6	108444105	108445534	*	9	0.000634718	-0.049399137	-0.0229392	*NA*
chr7	612458	612876	*	4	0.000637681	0.022717239	0.009844072	*PRKAR1B*
chr11	45392566	45393309	*	9	0.00065425	-0.023326382	-0.008044389	*NA*
chr10	22765645	22766320	*	5	0.000656812	0.04159917	0.018739098	*NA*
chr17	79167630	79167836	*	3	0.000657272	0.014534276	0.010421495	*CEP131*
chr10	124638200	124639782	*	16	0.000659001	0.037514551	0.01195541	*C10orf88B;FAM24B;FAM24B-CUZD1*
chr2	220197274	220197989	*	6	0.000661508	-0.055043332	-0.005452407	*RESP18*
chr11	8228022	8228508	*	3	0.00066326	-0.017385296	-0.009371198	*NA*
chr10	102760724	102761169	*	3	0.000664826	-0.028191615	-0.010327616	*LZTS2*
chr17	38472961	38473789	*	6	0.000666195	0.024213115	0.016650312	*RARA*
chr8	86350278	86351195	*	13	0.000683894	-0.017172481	-0.005738343	*CA13;CA3*
chr6	17280551	17283113	*	22	0.000687986	0.035383734	0.001714355	*RBM24*
chr5	40835193	40835760	*	13	0.000694981	0.050906163	0.009644882	*RPL37;SNORD72*
chr11	32416984	32417943	*	8	0.000694981	-0.031181655	-0.013947304	*WT1*
chr8	27850178	27850772	*	9	0.000699656	-0.021201063	-0.004145203	*SCARA5*
chr4	93225565	93226926	*	11	0.000701269	-0.011538745	-0.000415779	*GRID2*
chr14	29227941	29229249	*	8	0.000705309	-0.018075838	-0.009264715	*NA*
chr17	75765496	75766979	*	3	0.000710338	0.016183661	0.010082673	*NA*
chr16	52580266	52581405	*	6	0.000710479	-0.026007606	-0.01035458	*TOX3*
chr8	143858763	143860090	*	18	0.000713901	-0.037280507	-0.010485788	*LYNX1*

chr11	2720229	2722713	*	34	0.000714112	0.037415523	0.001689735	*KCNQ1;KCNQ1OT1*
chr21	38592612	38593284	*	5	0.000717513	0.031533588	0.008430281	*DSCR9*
chr1	231555522	231556682	*	5	0.000726622	-0.012145046	-0.007223957	*EGLN1*
chr7	47092529	47093015	*	4	0.000729316	0.035281977	0.019524516	*NA*
chr1	156072803	156074182	*	4	0.000730077	0.029373395	0.016465183	*LMNA*
chr20	741723	742683	*	4	0.000732102	0.031621893	0.017702187	*SLC52A3*
chr9	14315886	14316462	*	4	0.000732239	0.020043851	-0.000247437	*NFIB*
chr3	24535656	24537801	*	24	0.000733309	-0.023686024	-0.006212508	*THRB;THRB-AS1*
chr17	41131552	41132971	*	20	0.000733324	-0.019645673	-0.00303426	*PTGES3L;PTGES3L-AARSD1;RUNDC1*
chr11	118977379	118978508	*	13	0.000735977	-0.022213699	0.000576051	*C2CD2L;DPAGT1*
chr17	32581466	32582828	*	7	0.000736512	-0.016753906	-0.006260191	*CCL2*
chr6	30139478	30140713	*	21	0.000738937	-0.045706255	-0.002011705	*TRIM15*
chr6	28757018	28757569	*	4	0.000748643	-0.056615992	-0.028439213	*NA*
chr11	2846681	2847778	*	9	0.000749624	-0.017920288	-0.001359011	*KCNQ1*
chr1	236095453	236096728	*	3	0.000765938	-0.026797394	-0.020264496	*NA*
chr17	77815779	77816711	*	3	0.000768023	0.014299298	0.00450012	*CBX4*
chr20	30308956	30310246	*	5	0.000774686	0.023850931	0.01268471	*BCL2L1*
chr21	27106707	27108257	*	24	0.000775545	-0.024773138	-0.001568793	*ATP5PF;GABPA*
chr2	127412692	127414108	*	13	0.000776949	0.017273401	-0.004884669	*GYPC*
chr16	1122630	1123333	*	8	0.000778132	-0.030992002	-0.005361662	*SSTR5;SSTR5-AS1*
chr8	26370107	26371804	*	13	0.000779955	-0.014244634	-0.004882202	*DPYSL2;PNMA2*
chr1	10709185	10711695	*	10	0.000780283	-0.044508664	-0.017693263	*CASZ1*
chr4	57522493	57524770	*	15	0.000782661	-0.063789047	-0.012605333	*HOPX*
chr11	94134015	94135029	*	13	0.000783861	-0.035156307	-0.006475788	*GPR83*
chr1	24306751	24307535	*	13	0.00078831	0.020796534	0.000805834	*SRSF10*
chr19	18721531	18721840	*	3	0.000789944	0.034576462	0.010762236	*CRLF1;TMEM59L*
chr1	209798721	209799353	*	5	0.000789965	0.035425915	0.013758568	*LAMB3;MIR4260*
chr8	1892271	1892956	*	3	0.000799484	0.038849557	0.028709949	*ARHGEF10*
chr11	65149417	65150358	*	13	0.000806563	-0.01853212	-0.006361682	*FRMD8;SLC25A45*
chr8	74887999	74888597	*	8	0.000815165	0.049786298	0.002635493	*ELOC;TMEM70*
chr4	125634258	125635220	*	5	0.000819483	-0.030724789	-0.013004097	*ANKRD50*
chr7	24797102	24798855	*	16	0.000821295	0.026032977	0.009469819	*GSDME*
chr19	45579378	45580221	*	12	0.000821295	0.015761761	0.005706248	*GEMIN7;ZNF296*
chr4	1750374	1750476	*	3	0.000821295	-0.027836198	-0.011793796	*NA*
chr3	73610847	73611772	*	6	0.00082135	-0.026299237	-0.003999428	*PDZRN3*
chr6	74289464	74290516	*	4	0.000823766	0.037900162	0.011517388	*NA*
chr12	108634147	108634275	*	3	0.000832001	-0.044564667	-0.039830341	*WSCD2*
chr22	31002362	31003655	*	16	0.00083475	0.013732547	-0.004230699	*PES1;TCN2*
chr1	10601389	10601833	*	3	0.000835585	0.023532586	0.004894551	*PEX14*
chr1	10764512	10764896	*	3	0.000837408	0.033582522	0.022887537	*CASZ1*
chr4	104640560	104641554	*	11	0.000837719	-0.01831127	-0.00837507	*TACR3*
chr2	39187533	39188006	*	5	0.000841473	0.019120015	0.011799958	*ARHGEF33;LOC375196*
chr2	113992694	113994035	*	9	0.000841729	0.061941445	0.045375298	*PAX8;PAX8-AS1*
chr13	41706227	41708153	*	11	0.000842278	0.021100926	0.004473947	*KBTBD6*
chr15	51633381	51634583	*	8	0.000849751	-0.018672404	-0.008690056	*CYP19A1;GLDN*
chr6	16217234	16218436	*	5	0.000852747	-0.061487706	-0.027227531	*NA*
chr7	27224700	27226329	*	29	0.000853899	-0.029938445	-0.004693521	*HOXA10;HOXA10-HOXA9;HOXA11;HOXA11-AS*
chr16	88547533	88547861	*	4	0.000854763	0.018953167	0.01260916	*ZFPM1*
chr10	13481846	13482639	*	5	0.000855984	-0.0565927	-0.018909877	*BEND7*
chr21	46954058	46955202	*	5	0.000860372	-0.018104034	-0.006358256	*SLC19A1*
chr7	16625747	16626530	*	4	0.000861159	0.04843541	0.028766928	*NA*
chr14	105131910	105132869	*	7	0.000865208	0.025394202	0.006232595	*NA*
chr19	58094517	58095659	*	12	0.000871735	-0.020912371	-0.005111715	*ZIK1;ZNF416*
chr12	11708469	11709857	*	7	0.000874141	0.03821127	0.027815053	*LINC01252*
chr13	88324169	88325393	*	10	0.000879816	-0.028481997	-0.009883402	*MIR4500HG;SLITRK5*
chr6	44225648	44226520	*	7	0.000895489	0.024631039	0.008782647	*MIR4647;NFKBIE;SLC35B2*
chr17	80673675	80675235	*	13	0.000900453	-0.024538089	-0.007619171	*FN3KRP*
chr19	55013104	55013954	*	7	0.000906925	-0.063000923	-0.01789148	*LAIR2*
chr11	132662455	132662963	*	4	0.000910498	-0.05680831	-0.04346194	*OPCML*
chr1	156785998	156786723	*	9	0.000911622	-0.013890847	-0.005840457	*NTRK1;SH2D2A*
chr10	20104935	20106089	*	11	0.000920541	-0.016880873	-0.005051467	*PLXDC2*
chr10	135333451	135334122	*	7	0.000932104	-0.030406849	-0.01169644	*SCART1*
chr12	88973398	88974665	*	15	0.000935756	-0.01784291	-0.005234392	*KITLG*
chr22	30783074	30783995	*	10	0.000942649	0.052337058	0.007572036	*RNF215*
chr1	113050539	113051171	*	6	0.000943142	-0.025157841	-0.001779789	*WNT2B*
chr17	39890994	39892088	*	10	0.000947299	0.041552653	0.003729327	*HAP1;JUP*

chr17	80407031	80407779	*	8	0.000949888	-0.020627146	-0.00917125	*CYBC1*
chr4	567537	568514	*	4	0.000950617	0.041275476	0.003639563	*NA*
chr21	38069950	38071688	*	16	0.000953731	0.021229181	-0.000128521	*SIM2*
chr22	37750979	37751263	*	3	0.000953731	0.030526302	0.012017073	*NA*
chr18	76737888	76739120	*	10	0.000956068	-0.016549733	0.000863223	*SALL3*
chr21	46964226	46965042	*	7	0.000957846	0.026996098	0.014346153	*SLC19A1*
chr7	2701419	2701802	*	5	0.000958546	0.023847904	0.018044774	*TTYH3*
chr21	37535372	37536685	*	6	0.000971828	-0.024207539	-0.00725457	*DOP1B*
chr20	44033595	44034880	*	11	0.000972305	-0.017035984	-0.002586978	*DBNDD2;SYS1-DBNDD2*
chr11	5601832	5602123	*	4	0.000972305	0.020058328	0.003913098	*HBG2;OR52B6*
chr2	240144335	240146162	*	5	0.000974161	-0.037821177	-0.016658597	*HDAC4*
chr20	24973741	24974513	*	5	0.000983512	-0.036118008	-0.012736438	*APMAP*
chr7	26577897	26579614	*	4	0.0009843	0.039505487	0.017221517	*KIAA0087*
chr2	231732669	231734563	*	4	0.000987439	0.026573189	0.018527269	*ITM2C*
chr11	32451461	32452839	*	12	0.000991826	0.033253902	0.012720579	*WT1;WT1-AS*
chr6	150070030	150070714	*	7	0.000995221	0.025910051	0.013885763	*NUP43;PCMT1*
chr9	36738765	36739781	*	3	0.000995931	0.055219178	0.013379297	*NA*
chr7	157293538	157294502	*	8	0.000998853	0.027641831	0.012027103	*NA*
chr7	157646656	157647847	*	8	0.000999166	-0.01609246	-0.004916435	*PTPRN2;PTPRN2-AS1*
chr11	395625	397613	*	10	0.001006634	-0.046696286	-0.015557992	*PKP3*
chr16	86011615	86012573	*	7	0.00103007	-0.021833811	-0.010327177	*NA*
chr1	230777617	230778096	*	9	0.001031865	0.035740382	0.006389871	*COG2*
chr4	7647940	7649606	*	6	0.001040855	-0.023011845	-0.011349352	*SORCS2*
chr6	79942971	79944186	*	7	0.001054169	-0.033369975	-0.005076281	*HMGN3;HMGN3-AS1*
chr1	24068409	24069431	*	3	0.001054857	-0.011953978	0.001020018	*ELOA*
chr1	211652276	211652741	*	4	0.00105994	-0.034379165	-0.025102059	*RD3*
chr19	36605252	36606395	*	11	0.001061633	-0.008023807	-0.001825627	*POLR2I;TBCB*
chr13	103451435	103452789	*	11	0.001063289	0.006697712	0.000343421	*BIVM;POGLUT2*
chr6	166401018	166402753	*	15	0.001064916	0.014490969	-0.002056443	*LINC00473;LINC00602*
chr15	98835851	98837004	*	12	0.001065657	-0.045740832	-0.00478684	*NA*
chr3	29684415	29685093	*	4	0.001065676	0.016812773	0.004101501	*RBMS3*
chr11	62344463	62344975	*	4	0.001069117	-0.035385689	-0.010370736	*EEF1G;TUT1*
chr19	10697690	10698384	*	9	0.001073297	0.037036472	0.017250281	*AP1M2*
chr8	21988724	21989531	*	7	0.0010764	0.028915505	0.01268217	*HR*
chr6	33091111	33093109	*	18	0.001083282	0.047377572	0.016131642	*HLA-DPB2*
chr2	25427064	25427652	*	6	0.001087026	0.055378249	0.023803618	*NA*
chr20	36530296	36531652	*	9	0.001090013	-0.027896621	-0.006564957	*VSTM2L*
chr11	831584	832821	*	8	0.001096431	0.025008241	0.013662566	*CD151;CRACR2B*
chr4	187125413	187126139	*	5	0.001096431	-0.066502552	-0.035990704	*CYP4V2*
chr20	17943403	17944845	*	5	0.00109867	0.028650722	0.020529128	*MGME1;SNORD17;SNX5*
chr6	74018833	74020214	*	11	0.001100809	-0.027118781	0.000223751	*KHDC1*
chr14	73392648	73394847	*	16	0.001107905	-0.009880076	-0.000674173	*DCAF4*
chr10	45420821	45420996	*	3	0.001114226	-0.021061918	-0.016227622	*TMEM72;TMEM72-AS1*
chr11	107729479	107730551	*	13	0.001115086	-0.026364397	-0.005983208	*SLC35F2*
chr19	5977966	5978790	*	13	0.001115294	0.013209846	0.000540521	*LOC100128568;RANBP3*
chr1	158323482	158324471	*	4	0.00112526	-0.038792614	-0.010712967	*CD1E*
chr10	134120448	134121712	*	11	0.001133681	-0.026046493	-0.010343345	*STK32C*
chr5	149682233	149683334	*	9	0.001142194	0.020403494	0.000570528	*ARSI*
chr12	58011837	58013942	*	25	0.001143213	0.029880483	0.007266215	*SLC26A10*
chr2	139227577	139228296	*	3	0.001145729	-0.021559485	-0.012391821	*NA*
chr10	134790769	134791702	*	10	0.001145806	-0.038785181	-0.008015715	*NA*
chr2	160568689	160569678	*	15	0.00114636	-0.013081032	-0.001727972	*BAZ2B;MARCHF7*
chr2	109236744	109237735	*	5	0.001150743	-0.017785832	-0.01035518	*LIMS1*
chr12	6304557	6305192	*	6	0.001152773	0.019244685	0.008644464	*CD9*
chr2	88751158	88752836	*	13	0.001158827	0.01510685	0.002348803	*FOXI3*
chr4	38868940	38869779	*	15	0.001159377	0.027098787	0.002022644	*FAM114A1;MIR574*
chr14	104689833	104690882	*	8	0.001171434	-0.026021956	-0.011112631	*NA*
chr13	27745469	27746386	*	8	0.001172328	-0.033932047	-0.019386387	*USP12*
chr8	144371144	144372051	*	6	0.001172935	-0.071497867	-0.036936233	*ZNF696*
chr2	237088462	237088919	*	3	0.001173569	-0.017304328	-0.007724607	*NA*
chr16	31453406	31453948	*	4	0.001177932	0.035975484	0.017726073	*ZNF843*
chr7	56118783	56119637	*	13	0.001201086	-0.012211372	-0.000673288	*CCT6A;PSPH*
chr5	134180705	134182149	*	17	0.001207346	-0.032369198	-0.003829447	*C5orf24*
chr17	8868480	8869105	*	7	0.001213949	0.020847074	0.002316089	*PIK3R5*
chr12	12244945	12245710	*	3	0.001215218	0.021706417	0.019686609	*BCL2L14;ETV6*
chr19	54023732	54024435	*	13	0.001218854	0.026542812	-0.002301372	*ZNF331*

chr6	33286790	33291586	*	82	0.001225115	0.027356203	0.000560634	*DAXX;TAPBP;ZBTB22*
chr5	138677941	138678409	*	6	0.00122757	0.008353825	0.000815906	*PAIP2*
chr1	239549600	239550303	*	8	0.001229438	-0.030378547	-0.009534189	*CHRM3*
chr6	28554680	28558113	*	46	0.001239757	0.029802419	-0.002556156	*ZBED9*
chr22	42760647	42761604	*	3	0.001243829	0.036936544	0.0237581	*NA*
chr18	57564897	57567904	*	15	0.001258001	0.038742877	0.010357053	*PMAIP1*
chr15	37190548	37190998	*	5	0.001263864	0.037662564	0.007987883	*MEIS2*
chr14	105885531	105886413	*	11	0.001264752	-0.048467194	-0.019077462	*MTA1*
chr12	131355340	131355834	*	4	0.001274759	0.027834386	0.013772385	*RAN*
chr5	175199915	175200576	*	7	0.001275615	-0.023111889	-0.005896828	*NA*
chr5	176078832	176080248	*	3	0.001275889	0.027745226	0.001618539	*TSPAN17*
chr18	2906750	2907472	*	5	0.001279917	0.031357207	0.006291905	*EMILIN2*
chr22	23160841	23161379	*	4	0.001280756	-0.020049758	-0.008686835	*MIR650*
chr4	69312514	69313197	*	4	0.001281881	-0.034626374	-0.019901022	*TMPRSS11E*
chr7	93550752	93551275	*	9	0.001282922	-0.033764932	-0.010267093	*GNG11*
chr9	137660514	137660670	*	3	0.001284006	0.023383445	0.021074213	*COL5A1*
chr9	72590778	72591441	*	3	0.001288771	-0.030377706	-0.013991823	*NA*
chr8	6481431	6481608	*	3	0.001290472	-0.029215522	-0.020241091	*MCPH1*
chr7	27154537	27155974	*	15	0.001292215	0.022863442	0.011427176	*HOXA3*
chr7	101558597	101559888	*	12	0.001297644	0.023195459	0.006939638	*CUX1*
chr17	1636606	1637391	*	5	0.001298066	0.024814602	0.013645962	*WDR81*
chr3	9773022	9773672	*	6	0.001304477	-0.013933117	-0.004403431	*BRPF1*
chr19	55692003	55693151	*	5	0.00130588	-0.026908816	-0.013530483	*PTPRH;SYT5*
chr1	151300083	151300904	*	12	0.001305974	-0.025611129	0.003594664	*PI4KB*
chr15	96873850	96875830	*	17	0.001309999	0.026841369	0.000392263	*MIR1469;NR2F2;NR2F2-AS1*
chr1	16810670	16810776	*	3	0.001316166	0.024345007	0.018648435	*CROCCP3*
chr16	85427738	85429035	*	5	0.001316604	-0.020216305	0.00694872	*NA*
chr14	100068637	100070564	*	8	0.001329002	0.029627901	0.007990615	*CCDC85C*
chr7	42276810	42277410	*	14	0.001334893	-0.01210334	-0.000623711	*GLI3*
chr22	42679444	42679804	*	3	0.001357524	0.016926656	0.004895865	*NA*
chr17	48636344	48638703	*	31	0.001367276	0.023475186	0.000440425	*CACNA1G;CACNA1G-AS1*
chr5	87962828	87963592	*	7	0.001368533	-0.015188806	-0.006889104	*LINC00461;MIR9-2*
chr3	100053070	100053950	*	12	0.00137738	0.015249742	0.002788108	*NIT2*
chr1	25298480	25298996	*	3	0.00137738	0.020902881	0.007513939	*NA*
chr15	83875649	83877135	*	10	0.001379053	-0.018497447	-0.003474607	*HDGFL3*
chr5	6581774	6583103	*	6	0.001381004	0.034805846	0.009015501	*LINC01018*
chr4	47032325	47034181	*	15	0.001402297	0.031977604	-0.001004205	*GABRB1*
chr10	106087985	106090233	*	9	0.001402297	0.015214316	-0.000996205	*ITPRIP*
chr2	46842958	46843974	*	6	0.001405619	-0.00716525	-0.004107562	*CRIPT;PIGF*
chr5	16179012	16180419	*	16	0.001410583	-0.032320596	-0.009558192	*MARCHF11*
chr15	42119684	42120681	*	11	0.001423591	-0.025398299	-0.004156506	*JMJD7;JMJD7-PLA2G4B;MAPKBP1*
chr6	147091024	147092242	*	6	0.001423924	-0.025593086	-0.008996488	*ADGB*
chr9	29212927	29213880	*	6	0.001427796	-0.0131879	-0.007715192	*LINGO2*
chr5	1122753	1123154	*	5	0.001436365	0.013853369	0.008719538	*NA*
chr10	13204003	13204696	*	4	0.001444987	0.018982896	0.006916943	*MCM10*
chr17	37894014	37895886	*	15	0.001448588	-0.01811323	-0.008984902	*GRB7*
chr1	93544497	93545831	*	10	0.001451341	-0.014148012	-0.004787773	*MTF2*
chr1	44399069	44401857	*	18	0.001451911	-0.026521447	0.008123908	*ARTN*
chr8	145741413	145742229	*	4	0.001467437	0.021325001	0.007023465	*LRRC14;RECQL4*
chr13	93878969	93879769	*	9	0.001477141	-0.032477191	-0.0083831	*GPC6*
chr7	96745696	96746356	*	3	0.001479667	-0.019961465	-0.002158568	*SDHAF3*
chr17	75312165	75313094	*	3	0.001482685	0.025849835	0.019177638	*SEPTIN9*
chr2	191878353	191879164	*	12	0.001487264	0.0152448	-0.00036927	*STAT1*
chr14	61787418	61788345	*	10	0.001487712	0.03249313	0.002694686	*PRKCH*
chr9	139948584	139949577	*	6	0.001493464	0.029443476	0.016095408	*ENTPD2*
chr10	14050455	14052028	*	13	0.001494974	-0.044029766	-0.021908018	*FRMD4A*
chr3	101659630	101660066	*	3	0.001498735	-0.02755122	-0.015724231	*LINC02085*
chr9	136131118	136131739	*	5	0.001505786	0.051422145	0.02753718	*ABO*
chr2	154335184	154336401	*	16	0.001509489	0.023211292	-0.00291657	*RPRM*
chr19	1961017	1961727	*	5	0.001509975	0.017442637	0.0038546	*CSNK1G2*
chr17	56494554	56495283	*	5	0.001517257	0.021633284	0.002320314	*RNF43*
chr14	94423111	94424707	*	11	0.001518808	0.016768262	0.007320827	*ASB2*
chr20	43378953	43379501	*	4	0.001523403	0.042853919	0.019362778	*KCNK15*
chr10	89418800	89420794	*	21	0.001536325	0.017637988	0.001323591	*PAPSS2*
chr12	82152229	82153290	*	16	0.001537432	-0.019133772	-0.006135226	*PPFIA2*
chr6	30921592	30922043	*	3	0.001549836	-0.023278441	-0.008317013	*MUCL3*

chr19	8273505	8274513	*	14	0.001553649	0.046528163	-0.001281061	*CERS4*
chr9	118501557	118502187	*	4	0.001575491	-0.0269065	-0.010760915	*NA*
chr12	46766294	46767289	*	9	0.001578568	-0.035647562	-0.008626276	*SLC38A2*
chr6	53409850	53411302	*	12	0.001593751	0.024079034	0.002392635	*GCLC*
chr17	75032801	75033339	*	4	0.001595194	-0.028149271	-0.015545895	*NA*
chr17	79419366	79420424	*	8	0.001596361	0.02692046	0.005255553	*BAHCC1;MIR3186*
chr12	93835624	93837247	*	14	0.001596521	0.016902058	-0.001279427	*UBE2N*
chr1	95006977	95008479	*	14	0.001608691	-0.00959042	-0.00283054	*F3*
chr14	21904906	21906134	*	11	0.001609326	-0.029542926	-0.003695314	*CHD8*
chr5	176559334	176560265	*	5	0.001614716	0.056127848	0.026537036	*NSD1*
chr9	110249749	110251983	*	8	0.001617123	0.057637292	0.002536416	*KLF4*
chr6	26757644	26758395	*	5	0.001619249	-0.024645574	-0.012179607	*NA*
chr1	15911089	15912213	*	6	0.001619647	0.023487292	0.007224177	*AGMAT;DNAJC16*
chr3	49044317	49045655	*	17	0.001625916	-0.017586949	-0.001226223	*P4HTM;WDR6*
chr12	62860029	62861114	*	13	0.001631822	0.023325101	0.001269795	*MON2*
chr11	32455025	32456069	*	8	0.001631963	0.018591561	0.005218232	*WT1;WT1-AS*
chr3	3151899	3152916	*	6	0.001633743	0.024883609	0.01494043	*IL5RA*
chr1	1196457	1196747	*	3	0.001633743	0.031056586	0.024331172	*UBE2J2*
chr17	45898841	45899862	*	5	0.00163778	-0.031609939	-0.012393574	*OSBPL7*
chr2	220309434	220310232	*	6	0.001640571	0.027809589	0.012258057	*SPEG*
chr14	77294264	77294668	*	6	0.001641333	0.03673689	0.015265484	*LRRC74A*
chr22	25335346	25336286	*	7	0.00164324	0.027360826	0.013605334	*TMEM211*
chr19	14042840	14044197	*	5	0.001643745	0.025197453	0.014965996	*PODNL1*
chr9	34457129	34457500	*	4	0.001650191	0.024125919	0.014177018	*DNAI1;FAM219A*
chr12	31078790	31079406	*	6	0.001653025	0.029885518	0.012355804	*TSPAN11*
chr1	203097234	203098085	*	9	0.001656603	0.02191387	0.00237419	*ADORA1*
chr10	102802857	102803372	*	3	0.001657103	-0.021046521	-0.007257224	*NA*
chr18	44789949	44790841	*	4	0.00165769	-0.018705209	-0.012823761	*NA*
chr1	144995682	144996395	*	5	0.001658409	0.022945495	0.006870187	*LOC653513;NBPF20;NBPF9;PDE4DIP*
chr4	40057241	40059856	*	16	0.001659595	-0.018024138	-0.001895048	*LOC344967;N4BP2*
chr7	134001087	134002746	*	15	0.001672749	0.023826338	0.00546195	*SLC35B4*
chr17	67956864	67957967	*	6	0.001672749	0.021708101	0.005600788	*NA*
chr17	4079262	4079652	*	4	0.001673176	-0.025706732	-0.019608484	*ANKFY1*
chr10	44806558	44807790	*	4	0.001673176	-0.02871397	-0.000932264	*NA*
chr3	72226488	72227063	*	4	0.001683016	-0.015186459	-0.010933012	*NA*
chr17	8056492	8057770	*	5	0.001689885	0.011018108	-0.004197997	*PER1*
chr18	35145353	35147826	*	33	0.001705993	-0.014986158	-0.002899966	*CELF4*
chr14	104345945	104347395	*	8	0.001705993	-0.013490287	-0.009428986	*NA*
chr17	1956958	1958412	*	43	0.001708721	0.03306885	-0.000104482	*HIC1;MIR132;MIR212*
chr8	81397601	81397789	*	8	0.001723976	-0.054975521	-0.006407738	*ZBTB10*
chr2	233246431	233246922	*	3	0.001726458	0.030341555	0.015183595	*ALPP*
chr12	55725479	55725991	*	3	0.001729257	-0.024683619	-0.004299554	*OR6C3*
chr4	5990590	5991345	*	3	0.001733878	-0.022500876	-0.002396878	*NA*
chr19	821008	821565	*	3	0.001735153	0.011293175	-0.001341386	*PLPPR3*
chr2	3583570	3583963	*	3	0.001739438	0.042614949	0.028889713	*NA*
chr16	90014962	90016004	*	8	0.001744822	-0.035935663	-0.010793873	*DEF8*
chr16	66864375	66865626	*	10	0.001759419	0.02062426	0.00322204	*NAE1*
chr12	132862024	132864302	*	13	0.001761153	-0.028028171	-0.009101604	*GALNT9*
chr6	169283989	169285077	*	8	0.001768339	-0.020415524	-0.00712826	*NA*
chr10	79470169	79471118	*	5	0.001770193	-0.03242905	-0.009254766	*NA*
chr7	4874285	4875094	*	8	0.00177121	0.030261248	0.013438089	*RADIL*
chr17	8012578	8013680	*	7	0.001775189	0.035183856	0.012560198	*ALOXE3*
chr7	27212357	27214296	*	16	0.001777537	-0.021311528	-0.005447165	*HOXA10;HOXA10-HOXA9;MIR196B*
chr7	94537347	94538408	*	11	0.001791448	-0.011133411	-0.005388318	*PPP1R9A*
chr19	18509358	18509973	*	3	0.001794452	0.035514416	0.018624529	*LRRC25*
chr4	38320637	38321262	*	6	0.001796136	-0.02854387	-0.010766883	*NA*
chr13	112986575	112986978	*	5	0.001796539	-0.041779157	-0.024121189	*NA*
chr1	3055958	3057366	*	9	0.001804373	0.020664683	0.005501721	*PRDM16*
chr12	72148251	72148853	*	10	0.001813365	0.025588069	0.005172765	*RAB21*
chr5	125758447	125759291	*	10	0.001813753	-0.027522875	-0.004059729	*GRAMD2B*
chr12	113435438	113435980	*	3	0.001813939	0.020544215	0.011406856	*OAS2*
chr16	15950840	15951230	*	6	0.001819587	-0.024353188	-0.011937474	*MYH11*
chr21	45704786	45706101	*	15	0.001833905	0.031807159	0.014942939	*AIRE*
chr5	162992841	162993262	*	4	0.001838945	-0.035203229	-0.012247702	*NA*
chr19	42893103	42894930	*	10	0.001845255	-0.030957719	-0.011439214	*CNFN*
chr17	48919431	48920013	*	3	0.001847995	0.014834621	0.004832951	*WFIKKN2*

chr16	11723154	11724177	*	4	0.00185391	-0.02269908	-0.009767469	*NA*
chr17	19771360	19772219	*	8	0.001854711	0.030285478	0.019618289	*ULK2*
chr4	83719449	83720629	*	13	0.001855182	-0.023681092	-0.00424157	*SCD5*
chr2	33359059	33359688	*	11	0.001857956	-0.019741601	-0.009779196	*LTBP1*
chr16	3077878	3078339	*	3	0.001862066	-0.032272445	-0.010628251	*BICDL2;HCFC1R1;LOC100128770*
chr2	87015813	87016720	*	5	0.001862268	0.028867244	0.011640561	*CD8A;RMND5A*
chr19	38746538	38747292	*	11	0.001866052	0.026700623	0.00354262	*PPP1R14A*
chr11	117051786	117052397	*	4	0.001868138	0.026538725	0.013677999	*SIDT2*
chr6	111195301	111195976	*	9	0.001869621	-0.0198758	-0.003120012	*AMD1*
chr1	166049757	166050477	*	4	0.001873085	-0.019806324	-0.010875808	*FAM78B*
chr6	10382800	10384104	*	11	0.001876906	0.019557683	0.002960429	*NA*
chr2	99438903	99439997	*	5	0.00188014	0.028433128	0.014629671	*CRACDL*
chr3	46447826	46449636	*	16	0.00188161	-0.021152345	-0.008087392	*CCRL2*
chr13	79968174	79968324	*	3	0.00188161	0.030954006	0.025576944	*RBM26*
chr19	17597326	17597999	*	6	0.001883248	0.024119923	0.014178865	*SLC27A1*
chr10	21462441	21463858	*	14	0.001885716	-0.025010992	-0.004239047	*NEBL;NEBL-AS1*
chr6	30034178	30035234	*	18	0.001891411	-0.022309277	-0.002815436	*PPP1R11;ZNRD1ASP*
chr14	38724255	38724945	*	5	0.001893092	-0.019270199	-0.00806063	*CLEC14A*
chr5	1227968	1229127	*	5	0.001896877	-0.036923904	-0.020921319	*SLC6A18*
chr13	23412250	23412996	*	5	0.001901717	0.072909861	0.033717308	*NA*
chr6	18264287	18265299	*	10	0.001909447	-0.011031212	-0.001202153	*DEK*
chr6	166756218	166756786	*	4	0.001916828	-0.021172873	-0.008189349	*LOC100289495;SFT2D1*
chr8	144654887	144655679	*	12	0.001918556	-0.021980872	-0.007948344	*MROH6*
chr4	177713813	177715040	*	3	0.0019275	-0.036556656	-0.011819533	*VEGFC*
chr22	50523686	50524691	*	16	0.001930317	-0.014395262	-0.005846096	*MLC1;MOV10L1*
chr21	45883590	45883864	*	3	0.001933051	0.028632212	0.022825889	*NA*
chr8	11659497	11660968	*	19	0.001941171	0.014286871	0.003771144	*FDFT1*
chr6	31465344	31466722	*	19	0.001943742	-0.009114639	0.000120115	*MICB*
chr9	18473902	18474243	*	5	0.001953826	-0.015461528	-0.008448963	*ADAMTSL1*
chr13	77460298	77461426	*	15	0.001955796	0.027568702	0.00233776	*KCTD12*
chr5	115962086	115962584	*	3	0.001955796	-0.030048851	-0.020849584	*NA*
chr3	157260159	157261595	*	14	0.001960872	-0.015101293	-0.004041874	*SLC66A1L*
chr5	405488	406046	*	4	0.001960872	-0.014948132	-0.012431706	*AHRR*
chr5	132165899	132167026	*	7	0.00196805	0.028721424	0.007599446	*SHROOM1*
chr19	42873162	42873953	*	8	0.001968404	0.020772949	0.006764882	*MEGF8*
chr4	726697	728041	*	5	0.001976337	-0.028484602	-0.00594751	*PCGF3*
chr15	31617459	31617764	*	5	0.001978069	0.020956114	0.008706541	*KLF13*
chr7	81399587	81400745	*	7	0.001990593	-0.023259981	-0.008174373	*HGF*
chr16	84327245	84328719	*	13	0.001995557	0.010429461	-0.002089963	*WFDC1*
chr17	79817963	79819480	*	14	0.00199577	0.013150084	0.000374261	*P4HB*
chr6	4950308	4950673	*	3	0.00199577	-0.013629816	0.001267095	*CDYL*
chr12	132905611	132906443	*	9	0.001997496	-0.022063648	-0.012362503	*GALNT9*
chr2	26726190	26726995	*	5	0.002002139	0.011568411	0.00434033	*OTOF*
chr15	39873060	39874413	*	10	0.002005729	0.021650459	0.00216734	*THBS1*
chr20	45141911	45142576	*	9	0.002008233	-0.019176722	-0.006172155	*ZNF334*
chr7	2082255	2083583	*	9	0.00201604	0.04263867	0.004675991	*MAD1L1*
chr2	119603969	119605441	*	10	0.002018032	-0.025552879	-0.004797143	*EN1*
chr7	2551247	2551996	*	6	0.002018032	-0.01927938	-0.007952996	*LFNG*
chr15	34806592	34807221	*	5	0.00202147	-0.01433401	-0.006707387	*NA*
chr10	129845509	129846082	*	6	0.002023429	-0.022883801	-0.008268095	*PTPRE*
chr6	35479910	35480779	*	7	0.002024834	0.035115136	0.010398422	*TULP1*
chr18	24219872	24220737	*	3	0.002034958	0.027411964	0.014897312	*KCTD1*
chr6	83074812	83075187	*	3	0.002036346	0.030428792	0.015778156	*TPBG*
chr2	66664345	66665027	*	9	0.00204127	-0.015103518	-0.007600574	*MEIS1;MEIS1-AS3*
chr1	6313858	6314113	*	3	0.002045313	0.03055901	0.019840821	*GPR153*
chr1	113216243	113217204	*	13	0.002051012	-0.024562755	-0.005883886	*MOV10*
chr11	22687582	22688474	*	9	0.002061078	0.039720252	0.014662784	*GAS2*
chr6	31632171	31634890	*	52	0.002068472	0.023385127	-0.000991216	*C6orf47;CSNK2B;GPANK1;LY6G5B*
chr16	21963849	21965022	*	13	0.00208175	-0.023270792	-0.006328479	*UQCRC2*
chr16	4986962	4987927	*	12	0.00208175	-0.0187035	-0.00696983	*PPL*
chr20	17950131	17950965	*	5	0.002082153	0.03734606	0.004909582	*MGME1;SNX5*
chr15	85114169	85114479	*	3	0.002082153	0.015332766	0.00172808	*LINC00933;UBE2Q2P1*
chr1	112161618	112162084	*	4	0.002088251	0.065164708	0.03602836	*RAP1A*
chr20	43180850	43181661	*	3	0.00208993	0.032458696	0.025166719	*PKIG*
chr1	983386	984428	*	6	0.002091437	0.021414086	0.012145661	*AGRN*
chr22	50720813	50723019	*	8	0.002102035	0.025318359	0.007357074	*PLXNB2*

chr5	134364583	134364968	*	3	0.002102035	0.027749807	0.00929226	*C5orf66;PITX1*
chr6	41604740	41605994	*	5	0.002102369	0.046526461	0.010620818	*MDFI*
chr5	1489818	1489889	*	3	0.002111462	0.021218261	0.021009135	*LPCAT1*
chr19	53661755	53662958	*	11	0.002116703	-0.017756555	-0.005880606	*ZNF347*
chr20	25603976	25606029	*	19	0.002116844	0.021724287	0.003569033	*NANP*
chr19	58326009	58326618	*	10	0.002128475	-0.012980196	-0.006458637	*ZNF552;ZNF587;ZNF587B*
chr13	46189838	46190198	*	9	0.002128475	-0.014590191	-0.004821478	*ERICH6B*
chr8	146228110	146228920	*	14	0.002135106	-0.020280968	-0.002057481	*ZNF252P;ZNF252P-AS1*
chr18	67624071	67624393	*	3	0.002136426	-0.038377133	-0.016503316	*CD226*
chr2	74779799	74780272	*	7	0.002138369	0.037807542	0.014794946	*DOK1;LOXL3*
chr17	19290353	19291120	*	8	0.002147073	-0.014976798	-0.007909697	*MFAP4*
chr6	32811690	32813715	*	49	0.002156469	-0.012107758	-0.002724538	*PSMB8;PSMB8-AS1;TAP1*
chr4	99064102	99064904	*	9	0.002157865	0.039174896	0.01549373	*STPG2*
chr14	62331131	62331920	*	6	0.002170488	0.039979496	0.021727985	*NA*
chr14	102973717	102975405	*	8	0.002187099	0.027921514	0.011025479	*ANKRD9*
chr5	112072926	112073958	*	16	0.002190045	-0.03632232	-0.006311345	*APC*
chr13	34256111	34256553	*	3	0.002214077	0.015489527	0.012619361	*NA*
chr14	22950395	22951393	*	6	0.002216955	-0.033795263	-0.018242935	*NA*
chr16	1821856	1822795	*	6	0.002231763	0.023945951	0.003543942	*EME2;MRPS34;NME3*
chr1	114696350	114697113	*	14	0.002233925	-0.018511831	-0.00310039	*SYT6*
chr6	111888446	111888870	*	6	0.00224094	-0.017839671	-0.010767745	*TRAF3IP2;TRAF3IP2-AS1*
chr2	204103311	204104171	*	8	0.002242266	-0.033082112	-0.004714489	*CYP20A1*
chr13	113897950	113898827	*	3	0.002242266	0.02383318	0.004934597	*CUL4A*
chr19	3931338	3933856	*	14	0.002254848	0.025660598	-0.001733467	*NMRK2*
chr7	151503515	151503608	*	4	0.00225731	-0.017100579	-0.013548243	*PRKAG2*
chr3	129611515	129613115	*	10	0.002264027	0.028989627	0.00117682	*TMCC1;TMCC1-AS1*
chr22	43505892	43507665	*	30	0.002282624	0.029448524	0.001804404	*BIK*
chr9	101469203	101470884	*	7	0.002282624	0.040320882	0.001517375	*GABBR2*
chr19	42927668	42929067	*	9	0.002307541	0.033829892	0.011536259	*LIPE;LIPE-AS1*
chr1	63988325	63989705	*	18	0.002308411	-0.011682588	-0.003625963	*EFCAB7;ITGB3BP*
chr7	54609534	54610192	*	12	0.002313962	-0.017949835	-0.007005297	*VSTM2A*
chr7	27208577	27210940	*	19	0.002320291	0.02697755	-0.000285194	*HOXA10;HOXA10-AS;HOXA10-HOXA9;HOXA9;MIR196B*
chr12	133083102	133083593	*	4	0.002323066	-0.024825676	-0.016576341	*FBRSL1*
chr7	45613590	45614848	*	9	0.00232501	0.021328064	0.007046372	*ADCY1*
chr21	47169994	47170466	*	5	0.002327381	0.026641508	0.005428195	*PCBP3*
chr19	55995052	55996652	*	11	0.002337376	0.021525636	-0.001113508	*NAT14;SSC5D;ZNF628*
chr20	48184639	48186174	*	9	0.002343019	0.031829267	0.007898463	*PTGIS*
chr3	52231311	52231840	*	5	0.002346519	0.019541054	0.011878955	*ALAS1*
chr21	46798244	46798617	*	3	0.002351952	0.021564814	0.017039429	*NA*
chr17	68164127	68166241	*	17	0.00236618	-0.027685158	-0.005930146	*KCNJ2;KCNJ2-AS1*
chr5	139737425	139738364	*	4	0.002371823	0.014307736	-0.001872031	*SLC4A9*
chr10	75007255	75008351	*	6	0.002375318	0.015654078	0.003904571	*DNAJC9;DNAJC9-AS1;MRPS16*
chr17	76037035	76037562	*	6	0.002382131	-0.043175561	-0.021393495	*TNRC6C*
chr4	7512177	7512528	*	5	0.002385251	-0.045636182	-0.019843886	*SORCS2*
chr21	43327823	43327978	*	3	0.002385251	0.023372883	0.013162771	*C2CD2*
chr4	75230391	75231177	*	9	0.002385305	-0.029842653	-0.008080507	*EREG*
chr7	142582859	142583404	*	4	0.002385473	0.020114611	0.013805231	*TRPV6*
chr13	34250594	34251651	*	6	0.002387385	-0.027563788	-0.014757265	*STARD13*
chr6	106433985	106434623	*	5	0.002418536	0.058343519	0.014705586	*NA*
chr10	6441693	6443025	*	3	0.002423278	-0.019673875	-0.012559872	*NA*
chr11	27744049	27744759	*	7	0.002432485	-0.022067015	-0.006307075	*BDNF*
chr6	22147182	22148082	*	6	0.002439225	-0.015388652	-0.010056853	*CASC15;NBAT1*
chr7	117512803	117513956	*	8	0.002441978	-0.020014152	-0.009408706	*CTTNBP2*
chr12	133066054	133067734	*	20	0.002444211	-0.034488838	-0.011206051	*FBRSL1*
chr12	106974623	106977074	*	22	0.002463673	0.04441265	-9.37E-05	*LOC100287944;RFX4*
chr5	850745	851101	*	3	0.002467217	-0.03096605	-0.013786412	*ZDHHC11*
chr1	19832006	19832487	*	3	0.002487012	-0.021677301	-0.006338553	*NA*
chr8	146276606	146277845	*	5	0.002504977	-0.034452741	-0.001994904	*C8orf33*
chr1	209779784	209779995	*	3	0.002517869	0.030205691	0.011066249	*CAMK1G*
chr19	12776725	12777903	*	21	0.002518769	0.019684059	0.000445281	*MAN2B1;WDR83*
chr14	96000633	96001369	*	13	0.002518769	-0.018318056	-0.00208204	*GLRX5;SNHG10*
chr4	122853693	122854532	*	9	0.002518769	-0.050648821	-0.016167804	*TRPC3*
chr16	2479381	2480552	*	6	0.002518769	0.010308503	0.000766977	*CCNF*
chr20	58568926	58569565	*	4	0.002518769	0.019194229	-0.004203261	*CDH26*
chr5	7348324	7348673	*	3	0.002518769	-0.028060647	-0.020372181	*NA*
chr7	43232981	43233971	*	3	0.002531576	-0.038838848	-0.012581911	*HECW1*

chr11	70439937	70440910	*	5	0.002539802	-0.021832656	-0.001299915	*SHANK2*
chr14	23586582	23586886	*	3	0.002540887	-0.022577354	-0.015600343	*CEBPE*
chr19	8578767	8579199	*	9	0.002543502	-0.013564522	-0.001826123	*ZNF414*
chr6	29594481	29595661	*	13	0.002548837	0.03485652	0.011044366	*GABBR1*
chr19	42702481	42703852	*	5	0.00255692	0.012823906	0.00216578	*DEDD2*
chr11	3647086	3648013	*	7	0.00256622	-0.01579419	-0.00510869	*TRPC2*
chr14	31027639	31028244	*	9	0.00257683	-0.02598412	-0.00386589	*G2E3*
chr16	15818810	15819363	*	3	0.00257683	0.018508541	0.007954615	*MYH11;NDE1*
chr17	27892866	27893731	*	5	0.002577216	0.024674358	0.005239529	*ABHD15;TP53I13*
chr18	74203484	74204135	*	5	0.002579142	-0.012038806	-0.005123942	*ZNF516;ZNF516-DT*
chr1	6639249	6640149	*	10	0.002581248	0.033255894	0.009041698	*TAS1R1;ZBTB48*
chr2	26828219	26829772	*	6	0.00258474	-0.016461525	4.25E-05	*CIB4*
chr10	116696671	116697985	*	10	0.002598634	-0.030536853	-0.004317192	*TRUB1*
chr1	58898552	58898793	*	3	0.002601555	-0.062311841	-0.047451925	*DAB1*
chr10	22623047	22624374	*	9	0.002605382	0.029628392	0.010731096	*NA*
chr10	23632825	23634319	*	8	0.002608165	-0.032146124	-0.007114364	*C10orf67*
chr1	41284582	41285496	*	5	0.002621946	0.034689772	0.015108936	*KCNQ4*
chr5	149545907	149546934	*	8	0.002624186	0.042656255	0.022037603	*CDX1*
chr10	13628544	13629624	*	9	0.002627255	-0.009717767	-1.44E-05	*PRPF18*
chr3	49209160	49210221	*	3	0.002639425	0.022002361	0.010810333	*KLHDC8B*
chr22	19701108	19702664	*	14	0.00263954	0.028479998	-0.005679508	*SEPT5-GP1BB*
chr12	121976655	121976833	*	3	0.002645476	0.027357601	0.014906072	*KDM2B*
chr6	26043820	26044405	*	8	0.002650004	0.020676751	0.008189827	*H2BC3;H3C3*
chr9	130564509	130565470	*	13	0.002676984	-0.05311162	-0.009208801	*FPGS*
chr6	143771318	143772728	*	18	0.002682395	0.013026275	0.000779343	*ADAT2;PEX3*
chr7	45299616	45299766	*	3	0.002689141	-0.026802811	-0.008487408	*NA*
chr11	68637568	68638100	*	3	0.002691042	-0.06288094	-0.034597056	*NA*
chr16	67261905	67263524	*	6	0.002693471	-0.017494288	-0.000534454	*FHOD1;LRRC29;TMEM208*
chr11	116371188	116371487	*	5	0.002693471	-0.047055655	-0.026852001	*NA*
chr6	28583655	28584464	*	14	0.002697312	0.030686555	0.011741129	*NA*
chr8	69243752	69244553	*	7	0.00270055	-0.011811019	-0.005597537	*C8orf34;C8orf34-AS1*
chr10	60936025	60937257	*	19	0.002712832	-0.036960065	-0.007613308	*PHYHIPL*
chr17	7906521	7908103	*	6	0.00271819	0.032989947	0.014252683	*GUCY2D*
chr9	130454217	130454671	*	5	0.002718192	0.023431278	0.020101173	*MIR3911;STXBP1*
chr4	3318625	3319541	*	6	0.002722313	0.036102914	0.015174064	*RGS12*
chr10	105677453	105678654	*	15	0.002730318	-0.041342776	-0.008429161	*STN1*
chr12	125633643	125634553	*	6	0.002738604	-0.038231632	-0.014035111	*NA*
chr1	41444578	41445793	*	19	0.002760386	0.015755331	0.002683446	*CTPS1*
chr6	27258466	27259237	*	9	0.002760386	0.019195174	0.005319562	*NA*
chr16	67430099	67430454	*	3	0.002761574	0.026310391	0.022727295	*TPPP3;ZDHHC1*
chr2	229045958	229047110	*	12	0.002771176	-0.023711735	-0.007297383	*SPHKAP*
chr3	13521634	13522740	*	14	0.002790116	0.013869669	0.002741414	*HDAC11*
chr1	154237531	154238265	*	3	0.002804509	-0.053258057	-0.035874369	*UBAP2L*
chr7	98553760	98554099	*	4	0.002804752	0.019836216	0.013643294	*TRRAP*
chr9	135036217	135037343	*	9	0.002808433	-0.010500877	-0.002796471	*NTNG2*
chr2	25527080	25527366	*	3	0.002810546	-0.035068471	-0.01164316	*DNMT3A*
chr11	8008673	8009242	*	8	0.002828725	-0.060007894	-0.009571003	*EIF3F*
chr22	44464449	44465038	*	9	0.002831699	-0.017701175	-0.009866645	*PARVB*
chr12	122442301	122443562	*	4	0.002832364	-0.038851056	-0.023723107	*NA*
chr9	19103069	19104016	*	8	0.002838247	-0.019587994	-0.006661527	*HAUS6*
chr3	120003292	120003884	*	6	0.002850751	0.063801944	0.020880982	*NA*
chr8	144222015	144222455	*	5	0.002861448	-0.03931834	-0.029452343	*NA*
chr14	71066865	71067802	*	9	0.002863137	-0.038120158	-0.009527109	*MED6*
chr17	73029893	73031159	*	6	0.002863317	0.009237125	0.002617373	*KCTD2*
chr14	50910667	50910968	*	3	0.002872541	-0.037900642	-0.020582222	*MAP4K5*
chr20	21105829	21106966	*	12	0.002888886	-0.024745981	-0.010526045	*KIZ*
chr6	28504908	28505429	*	6	0.002891373	0.020334004	-0.004059525	*NA*
chr13	103425389	103426051	*	5	0.00289796	-0.019032042	-0.00361871	*TEX30*
chr13	96705123	96706104	*	10	0.002898107	-0.017175808	-0.007631618	*UGGT2*
chr6	110796274	110796813	*	3	0.002900125	-0.030356835	-0.014363393	*SLC22A16*
chr3	8774922	8775601	*	6	0.002903001	0.02566784	0.007611341	*CAV3;SSUH2*
chr1	43205605	43206309	*	6	0.002908746	0.015028918	0.009829217	*CLDN19*
chr14	102372393	102372657	*	3	0.0029126	-0.031804535	-0.005067209	*PPP2R5C*
chr18	74276822	74277491	*	3	0.002915986	-0.012578662	-0.008763901	*NA*
chr15	89377583	89378209	*	3	0.002919833	0.032939541	0.019349831	*ACAN*
chr5	111755039	111756360	*	9	0.002921056	0.020239735	0.001296115	*EPB41L4A;EPB41L4A-DT*

chr2	189155551	189156621	*	10	0.002930649	-0.015760434	-0.005679793	*GULP1*
chr1	218523325	218524558	*	3	0.002936033	-0.030940865	-0.014822925	*TGFB2;TGFB2-AS1*
chr15	29862036	29862801	*	5	0.002938502	-0.031342462	-0.014241035	*FAM189A1*
chr20	9496625	9497330	*	3	0.002954111	-0.008404988	-0.004044388	*LAMP5*
chr11	32125186	32125738	*	3	0.00295559	-0.018821548	-0.016168783	*RCN1*
chr6	136172226	136173085	*	7	0.00297616	-0.018366279	-0.010664952	*PDE7B*
chr1	222014008	222014996	*	4	0.002977564	-0.024131701	-0.016251601	*NA*
chr2	3129847	3130349	*	4	0.002979907	0.017906948	0.007541589	*NA*
chr17	72732432	72733290	*	15	0.002986439	0.024922316	0.00439106	*RAB37*
chr11	66034681	66036038	*	51	0.002988333	-0.042399932	-0.003466188	*KLC2;RAB1B*
chr2	167231969	167232696	*	7	0.00299777	-0.007519035	-0.003469714	*SCN9A*
chr2	203776148	203777060	*	13	0.00299792	-0.011510161	-0.001928577	*CARF;WDR12*
chr22	37499853	37500722	*	6	0.003000427	0.014201818	0.004911746	*TMPRSS6*
chr9	14346276	14347122	*	4	0.003009022	-0.009166851	0.001623486	*NFIB*
chr2	238768032	238769419	*	9	0.003011281	0.037675528	0.013815142	*RAMP1*
chr17	59493993	59494444	*	5	0.003011281	-0.00886824	-0.003318718	*NA*
chr10	80008235	80008637	*	4	0.003011281	-0.023781824	-0.012459923	*LINC00856*
chr15	41777661	41778987	*	4	0.00301795	0.041536926	0.017336879	*NA*
chr16	85027930	85028515	*	3	0.00301993	-0.021091056	-0.004056518	*ZDHHC7*
chr2	121223534	121224327	*	7	0.003019968	0.042972886	0.021352739	*LINC01101*
chr20	34129142	34130163	*	14	0.003023738	0.016110188	0.002998069	*ERGIC3*
chr1	23279278	23280477	*	7	0.003029638	-0.027874831	-0.016568917	*NA*
chr5	137367832	137369294	*	11	0.003033508	-0.020371354	-0.00462706	*FAM13B*
chr3	172312523	172313107	*	4	0.003033508	-0.056442666	-0.029414958	*NA*
chr3	88107415	88107754	*	3	0.003041539	-0.007103032	-0.002314554	*CGGBP1*
chr4	186456009	186456976	*	11	0.003045303	0.019413188	-0.003051425	*PDLIM3*
chr22	21213387	21214125	*	6	0.003048307	0.019314397	0.006045683	*PI4KA;SNAP29*
chr11	64405054	64406051	*	8	0.00307053	0.02780507	0.014295221	*NRXN2*
chr2	14772312	14773274	*	10	0.0030768	-0.02612876	-0.006087974	*LRATD1*
chr12	52240368	52242166	*	9	0.003092866	0.016753543	0.003547166	*NA*
chr4	3671067	3671697	*	5	0.003092866	0.026509151	0.018055948	*NA*
chr4	154680314	154680909	*	3	0.003097489	0.030776344	0.014669978	*RNF175*
chr5	55148279	55148974	*	5	0.0030981	0.071971511	0.006021333	*IL31RA*
chr3	51976427	51977385	*	5	0.003125364	0.023305149	0.012423095	*PARP3;RRP9*
chr1	154475068	154475688	*	4	0.003125616	-0.025303685	0.002899445	*SHE;TDRD10*
chr1	53526834	53528224	*	11	0.003129175	-0.012750815	-0.006770286	*PODN*
chr11	69631981	69633070	*	8	0.003129175	-0.020762751	-0.006751878	*FGF3*
chr15	68110824	68112070	*	8	0.003142703	0.042251066	0.018062333	*SKOR1*
chr6	38607245	38608904	*	15	0.003155613	-0.022134159	0.001000166	*BTBD9*
chr12	96336121	96337004	*	11	0.003164955	-0.022162286	-0.001100814	*AMDHD1;CCDC38*
chr1	35913782	35914081	*	3	0.003164955	-0.025138139	-0.017767257	*KIAA0319L*
chr8	68864012	68865187	*	13	0.003166367	-0.012846376	-0.003928452	*PREX2*
chr6	29454623	29455302	*	8	0.003167133	-0.025137769	-0.005908028	*MAS1L*
chr16	10275767	10276801	*	15	0.003170425	0.014322629	0.002869829	*GRIN2A*
chr16	2053627	2054287	*	5	0.003172953	0.031490382	0.020026757	*ZNF598*
chr3	62860802	62861925	*	13	0.0031751	-0.013488462	-0.00218122	*CADPS*
chr10	135341443	135343248	*	12	0.0031751	-0.063121552	-0.035726511	*CYP2E1*
chr5	1881655	1883514	*	11	0.0031751	-0.015879496	1.53E-05	*IRX4*
chr9	138391055	138393651	*	17	0.003184377	0.043442127	0.001473917	*C9orf116;MRPS2*
chr5	174150286	174151498	*	10	0.003187349	0.019420078	-0.001616145	*MSX2*
chr9	136844397	136844768	*	4	0.003191795	0.047338766	0.030025308	*VAV2*
chr7	149416432	149416973	*	9	0.003197885	0.01887315	0.004753536	*KRBA1*
chr5	149569501	149570189	*	5	0.003202901	0.016879463	0.002576328	*SLC6A7*
chr2	32389741	32390937	*	10	0.003207599	0.03263136	-0.00256139	*SLC30A6*
chr10	133775416	133775839	*	6	0.003214305	-0.022132132	-0.008965231	*NA*
chr12	57983971	57985234	*	11	0.00321948	-0.03949958	-0.004675049	*PIP4K2C*
chr4	20253277	20255985	*	20	0.003225038	0.017455716	-0.002044244	*SLIT2*
chr4	56023423	56024434	*	6	0.003225099	0.054620893	0.030831657	*NA*
chr6	18386873	18387809	*	12	0.003225924	-0.018137014	-0.005818798	*RNF144B*
chr1	54881486	54881929	*	6	0.003225951	0.017562175	0.004546897	*NA*
chr19	19416751	19417480	*	5	0.003233359	0.016946541	0.008544177	*SUGP1*
chr9	91148748	91149860	*	5	0.00324399	-0.022046974	-0.008378808	*NXNL2*
chr6	88031886	88032848	*	12	0.003260278	-0.016420051	-0.002080764	*GJB7;SMIM8*
chr12	16758414	16759391	*	14	0.003275516	-0.03020324	-0.005086774	*LMO3*
chr10	32667705	32667923	*	7	0.003275516	-0.010846248	-0.005989165	*EPC1*
chr11	113932106	113934010	*	9	0.003292992	-0.019212127	-0.010493711	*ZBTB16*

chr9	137772282	137772886	*	6	0.003303116	-0.025391536	-0.011380032	*FCN2*
chr17	79920132	79921131	*	7	0.003306496	0.025237765	0.015726017	*NOTUM*
chr17	78831167	78831863	*	5	0.003306592	0.019137451	0.010707271	*RPTOR*
chr10	65388604	65389292	*	4	0.003309315	-0.034572091	-0.008241175	*NA*
chr2	168150602	168151051	*	5	0.003309804	-0.012854188	-0.009333353	*NA*
chr2	24269866	24270726	*	9	0.003324487	0.025805483	-0.004427963	*FKBP1B;WDCP*
chr4	24914441	24914970	*	8	0.003328501	-0.022504258	-0.010722029	*CCDC149*
chr8	6263875	6264959	*	6	0.003328501	-0.011369877	-0.001752786	*LOC100287015;MCPH1*
chr20	2539517	2539876	*	5	0.003333521	-0.034297043	-0.011141126	*TMC2*
chr14	104551481	104552397	*	12	0.003338685	-0.022752698	-0.011532821	*ASPG*
chr5	150631895	150632733	*	11	0.003339018	-0.037428402	-0.012295035	*GM2A*
chr1	222011091	222011349	*	4	0.003353449	-0.030039226	-0.013334396	*NA*
chr11	115860682	115861018	*	5	0.003357518	-0.029148851	-0.024545745	*NA*
chr8	27468166	27469673	*	10	0.003362391	0.026912798	-0.006901208	*CLU*
chr12	58138320	58139148	*	10	0.003371408	-0.021107972	-0.007164567	*AGAP2;TSPAN31*
chr13	44947076	44948827	*	10	0.003382955	0.026357533	0.003730649	*SERP2*
chr11	64780701	64781842	*	8	0.003392652	0.014701718	0.000919907	*ARL2;ARL2-SNX15*
chr16	50347766	50347895	*	3	0.003392847	-0.029408249	-0.017567118	*ADCY7*
chr3	112930675	112931496	*	9	0.00340014	-0.014005951	-0.008428866	*BOC*
chr20	42983727	42984878	*	13	0.003405482	0.041269415	0.007494531	*HNF4A*
chr5	148017424	148018093	*	3	0.003409223	0.02590324	0.013636885	*HTR4;SH3TC2*
chr1	165414189	165415170	*	15	0.003411938	0.026473314	-0.004351462	*RXRG*
chr16	67464483	67465561	*	15	0.003411938	-0.032557685	-0.000820448	*HSD11B2*
chr21	42791867	42792703	*	11	0.003411938	0.017428193	0.003261193	*MX1*
chr12	118498668	118499294	*	4	0.003411938	-0.02295657	-0.005376305	*WSB2*
chr17	21186789	21187553	*	4	0.003414866	0.026834184	0.012664119	*MAP2K3*
chr7	42942369	42942901	*	4	0.003423605	-0.030704029	-0.019080472	*NA*
chr20	61147144	61148218	*	9	0.003433675	0.027414169	0.009320769	*MIR1-1;MIR1-1HG;MIR1-1HG-AS1*
chr8	22132434	22133356	*	14	0.00345146	0.026157156	0.009903607	*PIWIL2*
chr11	67210033	67210812	*	5	0.003464273	0.016039023	0.006448517	*CORO1B;PTPRCAP*
chr19	7894388	7895393	*	11	0.003465527	-0.03071486	-0.013088741	*EVI5L*
chr4	667199	668276	*	15	0.003465959	-0.01574677	-0.00371613	*ATP5ME;MYL5*
chr2	189158094	189158532	*	4	0.003466687	0.041149274	0.026437834	*GULP1;MIR561*
chr7	47293799	47294330	*	5	0.003478166	-0.00840762	-0.002418701	*NA*
chr8	10282549	10283275	*	6	0.003503288	-0.027577883	-0.006179007	*MSRA*
chr20	39799805	39800368	*	3	0.003515432	-0.011972108	-0.003775792	*PLCG1*
chr12	52262736	52263387	*	3	0.003524336	-0.019558604	0.000532969	*NA*
chr17	38023480	38024636	*	7	0.003540399	0.056279644	0.018275963	*IKZF3;ZPBP2*
chr6	28876139	28877723	*	8	0.003547463	-0.041971412	-0.002443116	*TRIM27*
chr14	64760831	64761822	*	11	0.003558699	-0.03273183	-0.004449027	*ESR2*
chr2	61200075	61200229	*	3	0.003558699	0.015827534	0.012898533	*PUS10*
chr4	56211228	56211433	*	3	0.003559908	-0.020461861	-0.005118637	*SRD5A3*
chr2	187713554	187714279	*	11	0.003560378	-0.030392913	-0.007227091	*ZSWIM2*
chr15	65822313	65822992	*	12	0.003562212	0.024192113	0.00176168	*HACD3*
chr20	58325159	58326180	*	3	0.003563008	-0.034792634	-0.009712924	*PHACTR3*
chr20	47895899	47897260	*	8	0.003570152	0.02906904	0.009741399	*SNORD12;SNORD12B;ZFAS1;ZNFX1*
chr2	208889973	208890802	*	12	0.003574244	-0.029014234	-0.008282587	*PLEKHM3*
chr6	33172333	33173581	*	24	0.003584777	0.030986073	0.004259231	*HSD17B8;MIR219A1;RING1;RXRB*
chr16	75589282	75590036	*	7	0.003585455	-0.009656201	-0.001895758	*TMEM231*
chr5	176326365	176327557	*	7	0.003586626	0.016981014	-0.001686893	*HK3*
chr4	53727431	53728510	*	7	0.00359499	-0.034421969	-0.009911899	*RASL11B*
chr1	43919181	43920264	*	17	0.003610053	-0.0183305	-0.004326626	*HYI;SZT2*
chr3	133748115	133748812	*	7	0.003634002	-0.028855596	-0.011075471	*SLCO2A1*
chr15	43809486	43810131	*	7	0.003640004	-0.014115316	-0.00737991	*MAP1A*
chr5	1644520	1645640	*	6	0.003640004	-0.024343826	-0.011877996	*NA*
chr7	25219543	25220412	*	12	0.003655064	-0.027070404	-0.007651863	*C7orf31*
chr2	71017541	71018823	*	12	0.003664775	0.035662127	0.011873534	*FIGLA*
chr20	37353700	37354117	*	3	0.003665955	-0.019544188	-0.001468217	*SLC32A1*
chr12	22093960	22095182	*	15	0.003668182	0.01972376	0.003256874	*ABCC9*
chr16	1574269	1575414	*	8	0.003670237	0.029021841	0.009996801	*IFT140;TMEM204*
chr6	26205212	26205610	*	4	0.003690776	-0.021018548	-0.002402529	*H4C5*
chr7	134832432	134833335	*	11	0.003700447	0.018911415	-0.001728838	*CYREN;TMEM140*
chr10	7708240	7709160	*	11	0.003708678	-0.032240198	-0.011965405	*ITIH5*
chr11	128775026	128776216	*	10	0.003708678	-0.017492693	-0.006301516	*C11orf45;KCNJ5*
chr6	131571435	131571669	*	4	0.00371195	-0.025434527	-0.014789992	*AKAP7*
chr19	38916488	38917025	*	6	0.003727397	-0.019476482	-0.004147405	*RASGRP4*

chr12	62995960	62998134	*	16	0.003741305	-0.015781697	-0.002694918	*LINC01465;MIRLET7I*
chr21	46291868	46292710	*	4	0.003753905	-0.01614045	-0.010944025	*PTTG1IP*
chr17	80621842	80622941	*	5	0.003758973	0.020075466	0.007071087	*RAB40B*
chr6	31164776	31165413	*	11	0.003781004	-0.040998327	-0.01450727	*HCG27*
chr13	61989037	61990113	*	16	0.003791048	-0.016037144	-0.003079401	*PCDH20*
chr11	61247223	61248887	*	9	0.003795138	0.028052998	0.006840728	*PPP1R32*
chr2	158732186	158733332	*	5	0.003797027	-0.029076618	-0.005226633	*ACVR1*
chr4	146401952	146402943	*	6	0.003799339	-0.020337511	-0.007853966	*SMAD1*
chr2	240161815	240162404	*	5	0.003801005	-0.018903045	-0.010686518	*HDAC4*
chr12	12848516	12850082	*	14	0.003803816	0.025049992	0.005606122	*GPR19*
chr3	51428079	51428687	*	11	0.003804211	0.025534128	0.000832231	*RBM15B*
chr1	10713944	10714701	*	5	0.003809572	0.022663913	0.011896263	*CASZ1*
chr6	29717475	29718119	*	5	0.003825975	0.031782155	0.00598489	*HLA-F-AS1*
chr2	179916080	179916205	*	4	0.003858634	0.022624713	0.014296555	*CCDC141*
chr1	1713676	1714271	*	6	0.003870861	0.026589226	0.013320524	*NADK*
chr7	984874	985784	*	9	0.003895336	0.017545603	0.0076486	*ADAP1*
chr12	103889042	103890083	*	14	0.003905778	-0.018028544	-0.00378965	*C12orf42*
chr1	86861147	86862273	*	14	0.003918394	0.01769493	-0.001396349	*ODF2L*
chr19	6502301	6502938	*	11	0.003918394	0.020337063	0.004465638	*TUBB4A*
chr2	47382287	47383720	*	14	0.003929803	0.019403305	-0.005764151	*STPG4*
chr3	184278268	184279593	*	11	0.003946856	-0.010767426	-0.001946655	*EPHB3*
chr21	40984285	40985406	*	12	0.003974197	-0.040715743	-0.011523577	*B3GALT5;B3GALT5-AS1*
chr15	78556178	78557094	*	12	0.00397773	0.026768258	0.003375181	*DNAJA4*
chr6	32945234	32946524	*	9	0.003987352	0.017201686	0.001691898	*BRD2*
chr3	159482413	159483186	*	4	0.004024088	-0.010621363	-0.001597338	*IQCJ-SCHIP1;SCHIP1*
chr6	154359967	154360894	*	12	0.004033203	-0.029977972	-0.001409827	*OPRM1*
chr16	30428842	30430044	*	5	0.004033203	0.023565069	0.010461094	*ZNF771*
chr13	114846655	114847515	*	4	0.004034828	0.013163966	0.005036344	*RASA3*
chr14	65380577	65381608	*	15	0.004036516	-0.034874411	-0.003358899	*CHURC1;CHURC1-FNTB*
chr17	53800041	53800484	*	5	0.004036516	-0.030624108	-0.010412344	*TMEM100*
chr7	933881	934221	*	4	0.004045053	0.015104808	0.005132197	*GET4*
chr6	21856149	21856613	*	3	0.004067202	-0.023640563	-0.011569098	*CASC15*
chr1	48463105	48463807	*	5	0.00407137	-0.050533356	-0.018515333	*TRABD2B*
chr10	97200985	97201222	*	4	0.004075086	-0.023269718	-0.013488124	*SORBS1*
chr13	28712950	28713767	*	7	0.004076137	-0.020307709	-0.004651632	*PAN3;PAN3-AS1*
chr1	150521766	150522965	*	8	0.004080578	0.029904259	0.004162478	*ADAMTSL4;MIR4257*
chr1	28831782	28832782	*	15	0.004081174	0.01614855	-0.003349784	*RCC1;SNHG3*
chr16	56388908	56390830	*	8	0.004092908	-0.020615962	-0.005932087	*GNAO1*
chr6	32140544	32141146	*	4	0.004095781	-0.018443748	0.006313771	*AGPAT1*
chr8	145103309	145103942	*	5	0.004106987	0.01386191	0.002765455	*NA*
chr2	160761085	160762289	*	12	0.004129804	-0.018910212	-0.003319141	*LY75;LY75-CD302*
chr11	84431163	84432312	*	8	0.004134592	-0.013864336	-0.005113305	*DLG2*
chr3	10362520	10363474	*	10	0.004141213	0.01929182	-0.000244343	*SEC13*
chr4	48018263	48019555	*	9	0.004145247	0.028110079	-0.002288379	*CNGA1;NIPAL1*
chr6	119401102	119401660	*	3	0.004149975	0.025760245	0.00107533	*FAM184A*
chr2	160472660	160473496	*	8	0.004154606	-0.020803352	-0.010827186	*BAZ2B*
chr14	60336433	60337477	*	7	0.004154606	-0.016618539	-0.010070623	*RTN1*
chr11	120106598	120107376	*	4	0.004158255	0.016083072	0.000768317	*POU2F3*
chr11	119018927	119019845	*	6	0.004164163	0.028635391	0.012449051	*ABCG4*
chr1	1027520	1028583	*	6	0.00416514	0.015708262	0.010796592	*C1orf159*
chr12	114843188	114845223	*	17	0.004165189	-0.028419359	-0.004656086	*TBX5;TBX5-AS1*
chr3	9744908	9745460	*	8	0.004167865	-0.018576815	-0.00674638	*CPNE9*
chr15	79723997	79725642	*	14	0.004173001	-0.023434615	-0.006388988	*MINAR1*
chr13	24825184	24825973	*	6	0.004181504	-0.031370559	-0.014934785	*SPATA13*
chr1	220101368	220102528	*	18	0.004195105	0.023837484	0.005229938	*RNU5F-1;SLC30A10*
chr5	176307323	176308026	*	6	0.004199073	0.017011839	0.006364794	*HK3;UNC5A*
chr18	74535233	74535991	*	7	0.004205499	0.018875484	0.010412335	*ZNF236;ZNF236-DT*
chr15	42840397	42841312	*	13	0.004208586	-0.009256914	-0.002557325	*HAUS2;LRRC57*
chr10	134900906	134901689	*	8	0.004219619	-0.030484726	-0.010305064	*ADGRA1*
chr12	126467559	126468899	*	9	0.00422921	-0.021491556	-0.007966133	*LINC00939*
chr6	44281348	44282149	*	5	0.004234368	0.032816477	0.010284626	*AARS2;SPATS1*
chr2	163625066	163625295	*	3	0.004235707	-0.052282942	-0.022082792	*KCNH7*
chr14	92572097	92573147	*	12	0.004241034	-0.015921009	-0.003780345	*ATXN3*
chr12	48151974	48153388	*	20	0.004243073	0.024889589	-0.001937068	*RAPGEF3*
chr16	84650990	84651521	*	4	0.004263413	0.009430578	0.00161005	*COTL1*
chr2	74735875	74736118	*	4	0.004274204	-0.021585216	0.001229224	*PCGF1;TLX2*

chr4	2401036	2402046	*	5	0.00427822	0.019430695	0.012672942	*ZFYVE28*
chr1	12508973	12509707	*	4	0.004280724	-0.034375036	-0.017445368	*VPS13D*
chr11	19261879	19263311	*	18	0.004280941	-0.01459494	-0.002041895	*E2F8*
chr12	125478229	125478868	*	7	0.004280941	-0.024742782	-0.006414657	*BRI3BP;DHX37*
chr15	41220951	41222276	*	12	0.004294622	-0.010216422	-0.00292331	*DLL4*
chr6	35490164	35490818	*	4	0.004308713	-0.015393622	0.004141755	*NA*
chr13	73355442	73356506	*	14	0.004309302	-0.011357633	-0.002369648	*DIS3;PIBF1*
chr7	100136013	100136517	*	5	0.004327518	-0.009132551	-0.00167343	*AGFG2*
chr17	55938642	55939590	*	7	0.004332211	-0.027799553	-0.001829544	*CUEDC1*
chr9	21994752	21996207	*	11	0.004335584	-0.030706684	-0.004385089	*CDKN2A;CDKN2B-AS1;MTAP*
chr12	129299088	129299702	*	7	0.004337256	-0.029981073	-0.011657442	*SLC15A4*
chr1	31423023	31423283	*	4	0.004359912	-0.021881192	-0.004701604	*PUM1;SNORD103A*
chr10	31288287	31288688	*	3	0.004363662	-0.025265967	-0.023644405	*ZNF438*
chr15	23933002	23933620	*	4	0.004374876	-0.026764147	-0.001418565	*NDN*
chr3	128721448	128722579	*	5	0.004379081	0.023893098	0.009246167	*EFCC1*
chr14	22938675	22939357	*	5	0.004381358	-0.033887821	-0.022507492	*NA*
chr4	184186563	184186934	*	4	0.004381358	0.023700978	0.018441726	*WWC2*
chr13	74864357	74864782	*	3	0.00438758	-0.041553266	-0.027302837	*NA*
chr1	41131751	41132022	*	3	0.00439299	-0.034616471	-0.00469085	*RIMS3*
chr10	49892203	49893346	*	11	0.00439604	0.017564977	-0.005263238	*WDFY4*
chr6	111926749	111928204	*	7	0.004399737	-0.02686777	-0.012470293	*TRAF3IP2*
chr10	118380116	118381044	*	5	0.004399737	0.03228123	0.018435868	*PNLIPRP2*
chr16	742426	743338	*	4	0.004403654	-0.052268182	-0.001919053	*FBXL16;WDR24*
chr3	173301431	173303193	*	8	0.004409666	-0.016840701	-0.008662872	*NLGN1*
chr11	117747030	117748236	*	18	0.004418335	0.030159309	0.006080946	*FXYD6;FXYD6-FXYD2*
chr6	167047310	167047877	*	3	0.004421301	-0.029344433	-0.020145111	*RPS6KA2*
chr16	58540765	58541612	*	4	0.004424921	0.016605626	-0.002030547	*NDRG4*
chr22	37641093	37641970	*	4	0.004427254	-0.024358931	-0.012600899	*RAC2*
chr15	80444736	80445874	*	11	0.004436136	-0.022379322	-0.01008025	*FAH*
chr3	23244051	23244348	*	8	0.004442599	0.017471401	0.00333476	*UBE2E2*
chr17	42295591	42296315	*	11	0.004442985	-0.042371144	-0.007190693	*UBTF*
chr3	121379745	121380792	*	9	0.004444748	-0.016980673	-0.004802262	*HCLS1*
chr14	74178976	74180391	*	6	0.004445571	0.018274193	0.004278439	*PNMA1*
chr14	65171010	65172436	*	13	0.004446774	0.021222823	0.000643993	*PLEKHG3*
chr17	42092187	42093151	*	14	0.004447835	0.015001235	0.000167411	*TMEM101*
chr8	98880145	98881927	*	15	0.004448408	-0.045434594	-0.004799669	*MATN2*
chr12	3361725	3361996	*	3	0.00445093	-0.012727738	-0.002574259	*TSPAN9*
chr1	28051702	28052784	*	14	0.004453309	0.013361975	-3.55E-05	*FAM76A*
chr3	57331843	57332341	*	3	0.004466452	0.015261649	0.008744005	*DNAH12*
chr16	401986	403022	*	4	0.004476479	-0.03720688	-0.027679705	*AXIN1*
chr9	99145998	99146400	*	7	0.004478006	-0.030231703	-0.005631715	*SLC35D2*
chr8	22560922	22561719	*	6	0.004497392	0.025665828	0.015271101	*NA*
chr12	105380444	105381266	*	3	0.004498803	-0.041446446	-0.006045955	*C12orf45*
chr3	157019345	157019556	*	3	0.004501588	0.023897695	0.008271393	*VEPH1*
chr19	52956310	52957180	*	11	0.00452359	0.03115087	-0.000330413	*ZNF578*
chr3	183893065	183894778	*	8	0.004525259	0.026082927	0.008621123	*AP2M1*
chr2	242003078	242003549	*	3	0.00452696	-0.037954575	-0.028293871	*SNED1*
chr15	41061384	41062224	*	10	0.004544238	-0.013292205	-0.007265303	*C15orf62;DNAJC17*
chr11	112034801	112035434	*	6	0.004549851	-0.023861858	-0.014604767	*IL18;TEX12*
chr6	137815333	137816901	*	11	0.00455534	0.013605456	-0.001986315	*OLIG3*
chr1	32222440	32223040	*	5	0.004555768	0.015641815	0.010426222	*ADGRB2*
chr15	93126401	93127379	*	5	0.004562467	-0.029497784	0.001511543	*NA*
chr5	124043354	124044098	*	5	0.004567925	-0.022571406	-0.015157925	*ZNF608*
chr22	32358655	32359081	*	3	0.004569558	0.017974166	0.008043281	*NA*
chr10	92979600	92980627	*	15	0.004581474	-0.028458085	-0.003753986	*PCGF5*
chr14	105308891	105309812	*	3	0.00458663	0.026252886	0.007042696	*NA*
chr16	20338303	20339420	*	7	0.004587716	0.024388199	0.009836557	*GP2*
chr12	66275403	66276974	*	13	0.004588701	-0.02152373	-0.007203961	*HMGA2*
chr20	3051493	3052483	*	13	0.004588701	0.028018184	0.013337769	*OXT*
chr17	72426469	72427563	*	13	0.004599585	0.013291304	0.000171781	*GPRC5C*
chr19	52799192	52800469	*	11	0.004601154	-0.022249727	-0.008490162	*ZNF480*
chr4	8207058	8207325	*	4	0.004602545	0.032265149	0.012435289	*SH3TC1*
chr2	198364282	198365671	*	23	0.004605458	-0.025641637	-0.002584203	*HSPD1;HSPE1;HSPE1-MOB4*
chr13	50703026	50703841	*	5	0.004606448	0.023883927	0.015650064	*DLEU1;DLEU2*
chr7	95064015	95065139	*	16	0.004619124	0.01357423	0.000376096	*PON2*
chr10	15355978	15356822	*	3	0.004619454	0.018466436	-0.001917502	*FAM171A1*

chr12	65218019	65218728	*	12	0.004622217	0.015826761	-0.001952676	*TBC1D30*
chr5	149339858	149340320	*	11	0.004628907	-0.01793565	-0.001849031	*SLC26A2*
chr9	130540941	130541531	*	6	0.004635858	-0.015178676	-0.00788124	*SH2D3C*
chr2	145090263	145090814	*	8	0.004640908	-0.024305247	-0.000638267	*GTDC1*
chr16	1429545	1430367	*	9	0.004651278	0.031794122	0.012420866	*UNKL*
chr17	37719431	37720009	*	3	0.004652014	-0.0383407	-0.014576074	*NA*
chr17	2595701	2595994	*	3	0.00465914	0.025336316	0.013692634	*CLUH*
chr1	247581408	247582066	*	7	0.004664699	0.021905604	-0.001483574	*NLRP3*
chr3	147072402	147073118	*	5	0.004665495	-0.023462848	-0.012422004	*NA*
chr1	58716018	58717107	*	11	0.004669643	0.029237121	-0.002957602	*DAB1*
chr19	6710658	6711092	*	3	0.004673694	0.033727458	0.022160583	*C3*
chr20	62369445	62370310	*	7	0.004675191	0.026631124	0.006730122	*LIME1;SLC2A4RG;ZGPAT*
chr13	39260874	39262298	*	11	0.004683782	-0.015122333	-0.00323653	*FREM2*
chr5	140580101	140580212	*	3	0.004683844	-0.0592924	-0.026725922	*PCDHB11*
chr2	10183603	10184650	*	11	0.004687405	0.055066521	0.014267179	*KLF11*
chr1	154539523	154540535	*	7	0.00468928	-0.031846852	-0.008829917	*CHRNB2*
chr2	106755003	106755721	*	6	0.004696998	0.029524114	0.015031884	*UXS1*
chr2	36780691	36780705	*	3	0.004698782	-0.024922632	-0.020916364	*FEZ2*
chr8	67940640	67941857	*	12	0.004705928	0.010935754	-0.00026691	*PPP1R42*
chr9	10611904	10612567	*	3	0.004706817	0.01526852	0.002069857	*PTPRD*
chr12	54349169	54350294	*	6	0.004708896	-0.030724779	-0.013152947	*HOXC12*
chr2	51259175	51259703	*	10	0.004713847	-0.025649707	-0.009838068	*NRXN1*
chr14	20903410	20904320	*	12	0.004721942	0.022242562	0.009740032	*KLHL33*
chr3	51742164	51742878	*	4	0.004722932	0.022774664	0.003209832	*GRM2*
chr7	5426831	5427251	*	3	0.004722932	0.018968457	0.006349246	*TNRC18*
chr7	114561493	114562847	*	12	0.004724054	0.042061448	0.009733669	*MDFIC*
chr11	123300323	123301490	*	10	0.004733401	0.012567908	-0.001090712	*NA*
chr5	1494980	1495356	*	4	0.004739199	0.018263718	0.009010531	*LPCAT1*
chr22	30969767	30970729	*	4	0.004744609	-0.049233468	-0.030899037	*GAL3ST1*
chr7	101603298	101603359	*	3	0.004749807	-0.018398564	-0.016781351	*CUX1*
chr1	248902767	248903325	*	7	0.004752525	-0.028797256	-0.012094993	*LYPD8*
chr19	37328842	37329859	*	17	0.004761136	0.018353873	-7.89E-05	*ZNF790*
chr5	160975347	160976417	*	12	0.004763258	-0.010684747	-0.006504189	*GABRB2*
chr19	50269440	50270616	*	12	0.004767734	-0.015841659	-0.00349614	*AP2A1;TSKS*
chr14	93580644	93581139	*	3	0.004779719	0.019612197	0.003210715	*ITPK1*
chr9	130699885	130701076	*	10	0.004800947	0.031604133	0.011593421	*DPM2*
chr21	37092789	37093340	*	4	0.004802323	0.016779656	0.006233831	*MIR802;RUNX1*
chr3	152879564	152880101	*	7	0.004810734	-0.005260317	-0.000588834	*RAP2B*
chr20	33813236	33814184	*	5	0.0048149	-0.018315897	-0.009672585	*EDEM2;MMP24*
chr8	130950848	130951373	*	3	0.0048149	-0.020339123	-0.017064887	*CYRIB*
chr12	132939165	132940939	*	7	0.004816602	0.040156225	-0.00023484	*NA*
chr11	64267330	64268315	*	4	0.004834421	-0.028699916	-0.012844087	*NA*
chr2	220378659	220379968	*	13	0.004842948	0.015052885	0.000545513	*ASIC4*
chr17	170770	171948	*	11	0.004846122	0.031129968	0.02203999	*RPH3AL*
chr5	102089184	102090704	*	8	0.004857449	0.02239253	0.006791579	*NA*
chr6	1624386	1625238	*	6	0.004857825	0.030421961	0.010801416	*GMDS*
chr20	36155979	36157405	*	43	0.0048589	-0.026988915	-0.002727026	*BLCAP*
chr6	32094845	32096219	*	31	0.004868288	-0.022439266	-0.003773863	*ATF6B*
chr1	47184410	47185236	*	13	0.004868288	0.021755198	0.000522267	*EFCAB14*
chr9	841067	841850	*	8	0.004868288	0.024793724	0.004756887	*DMRT1*
chr16	77468970	77470221	*	10	0.004871898	-0.031651229	-0.009833795	*ADAMTS18*
chr5	95196871	95197476	*	5	0.004884088	-0.025233751	-0.014986083	*NA*
chr4	186731926	186732207	*	3	0.004886137	0.024365167	0.010211261	*SORBS2*
chr18	28622474	28623390	*	14	0.004901519	-0.019673397	-0.008680049	*DSC3*
chr5	35924618	35925474	*	4	0.004901698	-0.010833847	-0.003350527	*CAPSL*
chr6	7726645	7726983	*	3	0.00491681	0.022570812	0.00876302	*BMP6*
chr12	6307048	6309025	*	7	0.004928118	0.023668051	0.014081145	*CD9*
chr7	158512380	158512671	*	4	0.004928118	-0.042261601	-0.017257858	*NA*
chr6	29974319	29975675	*	26	0.004930947	0.027779752	-0.001099913	*HLA-G;HLA-H;HLA-J;ZNRD1ASP*
chr12	131872163	131872391	*	3	0.004934687	-0.022672752	-0.015501359	*NA*
chr16	3013025	3013745	*	4	0.004936136	0.022500869	0.00534656	*KREMEN2*
chr1	200009927	200010626	*	4	0.004936136	0.019231715	0.005919159	*NR5A2*
chr19	536244	536929	*	5	0.004943453	0.027903016	0.006425425	*CDC34*
chr12	54408427	54409657	*	12	0.004943934	-0.01841696	-0.006550619	*HOXC4;HOXC5;HOXC6*
chr5	6632973	6633577	*	11	0.004945219	0.013149936	0.002032116	*NSUN2;SRD5A1*
chr19	15121199	15121596	*	11	0.004948501	0.024233034	0.015555753	*CCDC105;SLC1A6*

chr13	113303307	113303719	*	4	0.004948501	0.028025923	0.014208581	*ATP11AUN*
chr5	149996672	149997153	*	4	0.004958271	-0.044691865	-0.012088356	*SYNPO*
chr6	31855695	31857040	*	23	0.004959771	0.018191897	0.004469817	*EHMT2*
chr21	43815685	43816355	*	7	0.004961301	-0.023899291	-0.000265747	*TMPRSS3*
chr1	2382748	2383687	*	6	0.004965536	-0.025852219	-0.014545518	*NA*
chr4	737005	738172	*	6	0.004970095	-0.015717498	-0.00620202	*PCGF3*
chr15	88798666	88799707	*	7	0.004973109	-0.015172045	-0.004871123	*NTRK3;NTRK3-AS1*
chr8	140945782	140945843	*	3	0.004973109	-0.024756137	-0.014891665	*TRAPPC9*
chr4	46390730	46392485	*	17	0.004975417	-0.025739921	-0.00266794	*GABRA2*
chr8	144872060	144872852	*	5	0.004976387	0.023148694	0.008678377	*NA*
chr16	89951702	89951949	*	3	0.004979043	-0.015223	-0.008262476	*TCF25*
chr15	102345708	102345981	*	3	0.004995071	-0.027482485	-0.016883385	*OR4F6*
chr1	227974177	227975220	*	6	0.005003337	-0.031411675	-0.015538843	*NA*
chr1	153651147	153652247	*	8	0.005011851	-0.017421703	-0.004643349	*NPR1*
chr15	35087506	35088005	*	6	0.005014383	-0.017065291	-0.008677238	*ACTC1*
chr17	75445905	75446661	*	9	0.005018118	0.020163665	0.009367473	*SEPTIN9*
chr5	74807815	74808931	*	10	0.005026264	0.018205418	0.001429592	*CERT1;POLK*
chr19	52407881	52408518	*	10	0.005027318	-0.015969595	-0.006585875	*ZNF649*
chr6	36644997	36646551	*	16	0.005031145	0.032686301	0.004985402	*CDKN1A*
chr4	74296058	74296726	*	3	0.005041752	0.020732718	0.008030932	*AFP*
chr5	421317	422114	*	3	0.005045934	-0.035271914	-0.02354353	*AHRR*
chr10	112888790	112889349	*	3	0.005045934	-0.029742148	-0.022654064	*NA*
chr1	47644094	47646024	*	12	0.005047379	-0.035894803	-0.003103402	*LINC00853*
chr1	67773128	67773725	*	6	0.005047541	-0.012166284	-0.004206722	*IL12RB2*
chr3	184098184	184098596	*	4	0.005098828	-0.031039607	-0.018472494	*CHRD;THPO*
chr9	34662282	34663048	*	5	0.005118896	0.021965534	0.007885533	*CCL27*
chr12	8234656	8235314	*	8	0.00512212	-0.008670227	-0.003465856	*NECAP1*
chr2	231737741	231737958	*	5	0.00512212	0.023193671	0.012033168	*ITM2C*
chr2	42968	43812	*	3	0.00512212	-0.033149443	-0.009775241	*FAM110C*
chr6	3258005	3259961	*	15	0.00512349	-0.032041036	-0.005604935	*PSMG4*
chr11	116577961	116578399	*	4	0.00512349	-0.064496594	-0.029821306	*NA*
chr14	101499152	101499893	*	4	0.005125279	0.026081491	0.019422001	*MIR495*
chr2	3652840	3654582	*	7	0.005128172	-0.04079192	-0.008235315	*COLEC11*
chr5	96077898	96078834	*	5	0.005133629	-0.034871655	-0.026403508	*CAST*
chr3	196157895	196158975	*	7	0.005139061	-0.016327126	-0.004019268	*UBXN7*
chr6	83072573	83073619	*	10	0.005148262	-0.018981063	-0.006410955	*TPBG*
chr5	110427072	110427275	*	3	0.005159146	-0.019725356	-0.008418069	*WDR36*
chr3	132755839	132756669	*	6	0.005165643	-0.018032586	-0.006344555	*TMEM108*
chr20	2674548	2675418	*	4	0.005171993	0.040141643	0.018596923	*EBF4*
chr2	43188851	43189254	*	3	0.005177714	-0.017921773	-0.011864373	*NA*
chr5	346365	346987	*	3	0.005183976	-0.023370008	-0.016700038	*AHRR*
chr6	70990187	70991229	*	7	0.005195268	0.043567836	0.021173607	*COL9A1*
chr2	128388876	128389207	*	3	0.005195346	0.017851352	0.01340831	*MYO7B*
chr1	10487953	10488281	*	3	0.005198493	-0.027536609	-0.006315657	*CENPS;CENPS-CORT*
chr18	48345906	48346843	*	11	0.005211665	-0.024738894	-0.007327893	*MRO*
chr7	48702963	48703665	*	4	0.005227335	0.027492606	0.013596207	*NA*
chr6	78172192	78174286	*	15	0.005227971	-0.017360678	-0.004321439	*HTR1B*
chr3	85007959	85008991	*	11	0.005237457	-0.024790368	-0.004900412	*CADM2*
chr4	3726450	3726836	*	3	0.005243282	-0.016694112	-0.01381752	*NA*
chr1	3182620	3183072	*	3	0.005249693	0.026734942	0.003926822	*PRDM16*
chr2	106776571	106777215	*	6	0.0052512	-0.012615471	-0.004703563	*UXS1*
chr6	170596856	170598215	*	7	0.005254784	-0.036532627	-0.015744034	*DLL1*
chr4	20985623	20986246	*	7	0.005256458	-0.027635984	-0.012610029	*KCNIP4*
chr4	42398518	42399484	*	7	0.005268892	-0.011306821	-0.004744814	*SHISA3*
chr19	49636270	49636594	*	3	0.005273815	0.04666002	0.029240507	*PPFIA3*
chr7	92461971	92462936	*	6	0.005282827	-0.011483006	-0.000543237	*CDK6*
chr5	170813996	170814878	*	16	0.005291325	0.025420574	0.004013667	*MIR3912;NPM1*
chr3	101292669	101293068	*	11	0.005298936	-0.010183209	-0.003136446	*PCNP*
chr20	49574938	49575492	*	12	0.005301547	-0.010936097	-0.002388989	*DPM1;MOCS3*
chr5	59064235	59064682	*	11	0.00531136	-0.029351754	-0.010286565	*PDE4D*
chr19	35068221	35068788	*	5	0.005312907	-0.027344883	-0.010562136	*SCGB1B2P*
chr9	138966848	138967872	*	6	0.005329577	0.027274808	0.008081024	*NACC2*
chr12	113795897	113796846	*	9	0.005333839	0.025533521	0.004712014	*PLBD2*
chr2	220348515	220349208	*	6	0.005334716	-0.042265825	-0.010366489	*SPEG*
chr13	113199311	113200144	*	5	0.005334746	0.034524509	0.009444131	*TUBGCP3*
chr16	85539811	85540731	*	3	0.005336297	-0.039651891	-0.018908936	*NA*

chr22	42196285	42197038	*	11	0.005338902	0.01981493	-0.002075806	*CCDC134*
chr3	111717534	111718457	*	14	0.005354906	-0.016564547	-0.009299046	*TAGLN3*
chr10	134844217	134844771	*	5	0.005356794	-0.040860565	-0.014377622	*NA*
chr15	93197833	93199536	*	16	0.005357399	0.023531251	0.000125748	*FAM174B*
chr1	175985115	175986331	*	5	0.005379113	-0.028558787	-0.003294629	*COP1*
chr16	30485296	30485966	*	7	0.005381015	0.02190597	0.01541582	*ITGAL*
chr1	36771493	36773099	*	15	0.005401161	0.032770703	0.001928713	*SH3D21*
chr20	33879989	33881762	*	12	0.005403129	0.025781109	0.010473476	*FAM83C*
chr10	48439216	48439452	*	8	0.005406749	-0.028245603	-0.011184385	*GDF10*
chr6	155450088	155450882	*	4	0.005413381	-0.022446306	-0.00622527	*TIAM2*
chr16	10674008	10675123	*	12	0.005420747	-0.016845768	-0.001539391	*EMP2*
chr21	46685154	46686430	*	6	0.005424448	-0.033653142	0.003059261	*POFUT2*
chr10	23479993	23480237	*	4	0.005428548	-0.016402631	-0.010605902	*PTF1A*
chr19	56651829	56652545	*	11	0.005433568	-0.010373296	-0.00128829	*ZNF444*
chr15	99499605	99499719	*	3	0.005440054	-0.029301838	-0.023460965	*IGF1R*
chr1	48191436	48191880	*	3	0.005440383	-0.029480368	-0.013935714	*NA*
chr14	75469661	75470314	*	5	0.005443242	0.009035051	0.001108955	*EIF2B2*
chr7	30959709	30960418	*	5	0.005458336	-0.025777733	-0.008591388	*AQP1*
chr6	43043516	43044371	*	7	0.005464051	-0.010303851	-0.00212977	*PTK7*
chr2	120005223	120005755	*	6	0.005481572	0.027290809	0.013157874	*STEAP3*
chr12	48275709	48276809	*	8	0.005485878	-0.013774944	-0.00646294	*VDR*
chr7	6048837	6048965	*	4	0.005491803	0.010216119	0.001702778	*AIMP2;PMS2*
chr3	140796013	140796183	*	4	0.005496451	-0.015233255	-0.010879899	*SPSB4*
chr10	108923111	108924867	*	9	0.005501899	-0.022462167	-0.012111205	*SORCS1*
chr18	45457451	45458243	*	7	0.005530342	-0.020431908	-0.011029494	*SMAD2*
chr11	20229517	20230330	*	5	0.005532102	-0.019029312	-0.017087287	*NA*
chr6	27804866	27806842	*	13	0.005537112	-0.046210101	-0.004515597	*H2AC15;H2BC15*
chr12	42326382	42326707	*	3	0.00555627	-0.016791593	-0.012128244	*NA*
chr16	68321421	68321730	*	6	0.005557189	-0.021629005	-0.012784393	*SLC7A6*
chr20	62421032	62421950	*	5	0.005557189	-0.033024587	0.002443608	*ZBTB46*
chr2	38604442	38604853	*	4	0.005559825	-0.024760493	-0.002493097	*ATL2*
chr3	12392792	12393021	*	3	0.005575742	-0.022625419	-0.012691305	*PPARG*
chr7	140103619	140104222	*	8	0.005576911	-0.014858758	-0.007868847	*RAB19*
chr4	52708582	52709895	*	12	0.005579823	-0.03780442	-0.004586596	*DCUN1D4*
chr9	35689475	35690395	*	8	0.005588474	0.039820734	0.012354543	*TPM2*
chr17	79800859	79801920	*	10	0.005602698	-0.030434806	-0.006270666	*P4HB*
chr19	10220013	10220911	*	10	0.005604053	-0.018201847	-0.001937606	*P2RY11;PPAN;PPAN-P2RY11;SNORD105B*
chr1	9351985	9352663	*	5	0.005617419	0.024377349	0.008972804	*SPSB1*
chr6	30042137	30043418	*	26	0.00562156	0.025920003	-0.001425697	*RNF39*
chr6	45389313	45391973	*	20	0.005623479	-0.024754544	-0.004254759	*RUNX2*
chr3	15642568	15643903	*	27	0.005624669	-0.014826175	-8.99E-05	*BTD;HACL1*
chr10	115613834	115614541	*	19	0.005626189	-0.012533412	-0.00210749	*DCLRE1A;NHLRC2*
chr10	88295210	88295591	*	4	0.005626189	0.030941246	0.012452379	*NA*
chr4	101111617	101112395	*	9	0.005633036	-0.016777351	-0.006140492	*DDIT4L*
chr16	55512521	55515248	*	22	0.005633635	-0.029914344	-0.005211354	*MMP2*
chr3	127633887	127634459	*	9	0.005654615	0.032389433	0.00979673	*KBTBD12*
chr15	25319113	25320195	*	4	0.005662715	-0.03342423	-0.01132632	*IPW;SNORD116-10;SNORD116-11;SNORD116-12;SNORD116-13*
chr22	36019252	36019632	*	5	0.005670402	0.008360041	-0.001233801	*MB*
chr12	57587701	57588350	*	9	0.005673121	0.01691118	0.002588796	*LRP1;MIR1228*
chr4	5890358	5890751	*	7	0.005673121	-0.016924453	-0.005082064	*CRMP1*
chr20	707906	708659	*	3	0.005673121	0.027542019	0.006082301	*NA*
chr1	32929715	32931036	*	15	0.005673381	-0.014815195	-0.003616974	*ZBTB8A;ZBTB8B*
chr20	30795258	30796171	*	13	0.005673381	-0.01581903	-0.001404319	*PLAGL2;POFUT1*
chr5	101834136	101835072	*	10	0.005673381	0.025915612	0.011058772	*SLCO6A1*
chr4	2427356	2428172	*	5	0.005678807	0.021736376	0.009378804	*CFAP99*
chr4	183245061	183245618	*	5	0.005694729	0.020965583	-0.001588155	*TENM3*
chr8	26150452	26150784	*	3	0.005698148	0.01139737	0.007071752	*PPP2R2A*
chr5	148651771	148652108	*	3	0.005700091	-0.007477964	-0.004096954	*AFAP1L1*
chr12	752290	753199	*	9	0.005707633	0.021153914	0.009056121	*NINJ2*
chr17	35165324	35166190	*	5	0.005710217	-0.023497235	-0.010033202	*NA*
chr17	48474379	48475619	*	11	0.005716472	-0.034523155	-0.007598907	*LRRC59*
chr12	51403056	51403474	*	5	0.005729254	-0.014868999	-0.006776839	*SLC11A2*
chr1	36553577	36555392	*	11	0.005729716	-0.026552785	-0.001452633	*ADPRS;TEKT2*
chr1	154377091	154377621	*	9	0.005736902	-0.016106676	-0.007327411	*IL6R*
chr22	24819482	24820655	*	7	0.005736902	0.016434561	0.007163568	*ADORA2A;SPECC1L*
chr8	142278132	142278585	*	5	0.005760001	0.018096895	0.00532039	*NA*

chr18	13824072	13824396	*	5	0.005768042	-0.015392239	-0.004189467	*MC5R*
chr13	113121107	113121632	*	4	0.005769802	0.019004812	0.0087651	*NA*
chr15	100272053	100272751	*	3	0.005774582	0.024971029	0.022130216	*LYSMD4*
chr10	102510356	102510568	*	3	0.005796435	-0.033626704	-0.015250466	*PAX2*
chr13	41935539	41936259	*	3	0.005807794	-0.028093082	-0.014707327	*NAA16*
chr20	58629939	58630954	*	7	0.005810528	-0.020400353	-0.007669198	*C20orf197*
chr4	901785	902074	*	3	0.005812164	-0.032787839	-0.013447335	*GAK*
chr19	3785573	3786125	*	3	0.00581327	-0.018512744	-0.009840787	*MATK*
chr5	1033518	1034307	*	5	0.00581707	0.015161421	-0.000924262	*NKD2*
chr14	105553150	105554214	*	5	0.005817833	0.013242895	0.004515884	*NA*
chr3	13008800	13010184	*	16	0.005843129	-0.032646136	-0.004262267	*IQSEC1*
chr7	1908250	1908745	*	5	0.005857899	0.020154449	0.006956925	*MAD1L1*
chr19	2782678	2783156	*	3	0.005864542	-0.008857124	-0.006978628	*SGTA;THOP1*
chr4	88312323	88313064	*	10	0.005865514	-0.048633441	-0.012705978	*HSD17B11*
chr3	107809116	107810687	*	13	0.005875316	0.023459021	0.002423394	*CD47*
chr3	194118478	194119188	*	7	0.005878908	-0.020201986	-0.006193595	*GP5*
chr2	121303344	121304074	*	3	0.005882924	0.023338582	0.00049358	*NA*
chr13	92050483	92051154	*	11	0.005896745	-0.029558447	-0.008453583	*GPC5*
chr13	103719706	103720206	*	3	0.005896745	-0.026235768	-0.004553921	*SLC10A2*
chr17	80315917	80316908	*	4	0.005897592	-0.022575814	-0.006544945	*TEX19*
chr12	34464089	34464939	*	3	0.005899704	-0.025324769	-0.021835354	*NA*
chr10	98588227	98588605	*	4	0.005909442	0.035034165	0.006846575	*LCOR*
chr1	161100749	161101135	*	4	0.00591479	0.016129829	0.008719602	*DEDD*
chr2	50116848	50117117	*	3	0.005920898	-0.037024385	-0.003675729	*NA*
chr7	100423987	100424532	*	3	0.005920898	0.010099976	0.00244131	*EPHB4*
chr3	118891705	118892461	*	7	0.005929926	-0.034261389	-0.016100563	*UPK1B*
chr4	111532410	111533547	*	8	0.005930982	-0.012062524	-0.004132052	*NA*
chr19	11958966	11960208	*	6	0.005936356	0.014223202	-0.001115229	*ZNF439*
chr1	1475143	1476269	*	9	0.005938146	-0.032230402	-0.000740059	*TMEM240*
chr15	42066071	42066822	*	10	0.00595158	0.005898596	0.000517211	*MAPKBP1*
chr2	74668072	74669573	*	13	0.005953825	0.035943079	0.017622867	*RTKN*
chr11	64013441	64015574	*	16	0.005964352	0.020348372	0.001076309	*PLCB3;PPP1R14B*
chr10	135050343	135052004	*	27	0.005976156	0.028732366	0.00747605	*VENTX*
chr1	75198211	75199496	*	15	0.005977158	-0.025586008	-0.009198753	*CRYZ;TYW3*
chr10	79396127	79397075	*	5	0.005978517	0.021328162	0.010632361	*KCNMA1*
chr2	32490589	32491049	*	6	0.005995378	-0.023258427	-0.008732615	*NLRC4*
chr10	125732373	125732842	*	4	0.00599939	-0.034235729	-0.013052595	*NA*
chr1	6187633	6188136	*	5	0.006016966	-0.060834895	-0.028555293	*CHD5*
chr4	100273969	100274565	*	4	0.006016966	0.037245751	0.019428961	*ADH1C*
chr2	236577585	236578808	*	8	0.006019733	0.017218522	-0.00049538	*AGAP1*
chr4	57181855	57182417	*	3	0.006019733	0.027938442	0.01387808	*CRACD*
chr7	22765321	22766155	*	5	0.006020531	0.023648075	0.01161965	*IL6*
chr3	48486890	48487471	*	3	0.006024354	0.021491721	0.016288179	*ATRIP*
chr8	26433537	26434069	*	4	0.006029793	0.030141247	0.02019256	*DPYSL2*
chr8	145082140	145082728	*	3	0.006049135	0.028811718	0.006038664	*SPATC1*
chr20	44419733	44421026	*	17	0.006049926	-0.022153776	-0.003750813	*DNTTIP1;WFDC3*
chr2	33151557	33152037	*	3	0.006049926	-0.02553849	-0.005851795	*LINC00486*
chr15	65203541	65204428	*	13	0.006051237	0.02638892	0.001363721	*ANKDD1A*
chr12	122230490	122230972	*	3	0.00606801	0.015203789	0.006510861	*RHOF*
chr13	112861499	112862112	*	4	0.006073538	-0.039201525	-0.029187203	*NA*
chr1	2207859	2208225	*	3	0.006073538	-0.02668511	-0.015999838	*SKI*
chr4	7048059	7048926	*	6	0.006080249	0.023936675	0.009205125	*CCDC96;LOC100129931;TADA2B*
chr1	95392125	95393138	*	10	0.006085472	-0.02308428	-0.006730732	*CNN3;CNN3-DT*
chr22	22555873	22556540	*	4	0.006085472	-0.026521516	0.000116626	*NA*
chr6	127839822	127840334	*	6	0.006090182	-0.018182991	-0.005835561	*SOGA3*
chr5	139742283	139743303	*	3	0.006098369	0.026373063	0.013505983	*SLC4A9*
chr7	1913505	1914523	*	6	0.006100933	0.027401711	0.011737291	*MAD1L1*
chr10	32344997	32346148	*	13	0.00610879	0.02099327	-0.002922341	*KIF5B*
chr6	10412073	10413394	*	9	0.00610879	0.023228222	0.00213842	*TFAP2A;TFAP2A-AS1*
chr9	129676656	129677882	*	11	0.006118226	-0.024544925	-0.009506366	*RALGPS1*
chr6	30457711	30458730	*	18	0.006120223	-0.017753828	-0.003153817	*HLA-E*
chr2	218898491	218898788	*	3	0.006132479	0.021024622	0.017592625	*RUFY4*
chr19	2307901	2308653	*	8	0.006134209	-0.015382837	-0.005782006	*LINGO3;SPPL2B*
chr19	37340642	37341870	*	18	0.006142801	-0.031690764	-0.001137166	*ZNF345;ZNF790*
chr8	38830814	38831857	*	10	0.00614728	-0.019760589	-0.007718041	*HTRA4;PLEKHA2*
chr18	29598757	29599349	*	4	0.00614728	-0.008726591	-0.002874498	*RNF125*

chr4	89744279	89744963	*	6	0.006176859	-0.011915936	-0.004285224	*FAM13A*
chr1	209957965	209958678	*	9	0.006178619	-0.017758722	-0.005474861	*C1orf74*
chr8	6795989	6796618	*	6	0.006190687	-0.019184348	-0.010714735	*DEFA4*
chr9	139871820	139872839	*	5	0.006194563	0.033644045	0.002236151	*LCNL1;PTGDS*
chr16	85110539	85111583	*	3	0.006194918	0.02259583	0.019103483	*KIAA0513*
chr12	81329566	81330609	*	5	0.006195055	0.034155322	0.010238005	*LIN7A;MIR618*
chr22	33195343	33196425	*	10	0.006198244	0.025588275	0.004325429	*SYN3;TIMP3*
chr8	142950423	142950502	*	3	0.006199685	-0.038403272	-0.016069227	*NA*
chr14	106015750	106016106	*	3	0.006199685	0.019090293	0.00689657	*NA*
chr13	113582793	113583686	*	3	0.00620233	-0.030608621	-0.022183431	*NA*
chr6	159240453	159241461	*	8	0.006218395	-0.017468476	-0.001965617	*EZR;EZR-AS1*
chr3	114012316	114012912	*	5	0.006230947	0.028269623	0.011742083	*TIGIT*
chr5	178957344	178957930	*	4	0.006235153	0.012155648	0.002822701	*NA*
chr7	30028890	30030138	*	16	0.00624941	0.049525781	0.000582477	*SCRN1*
chr1	2426693	2427655	*	4	0.00624941	-0.020984995	-0.000451297	*PLCH2*
chr12	130936355	130937365	*	7	0.006256097	-0.035588384	-0.008994032	*RIMBP2*
chr11	66496615	66497215	*	4	0.006278441	0.024757491	0.017612915	*NA*
chr8	67038980	67039461	*	7	0.006283273	0.025900922	0.009802032	*TRIM55*
chr22	46465717	46466858	*	7	0.006283376	-0.038012172	-0.013681715	*MIRLET7BHG*
chr19	5210505	5210847	*	5	0.006284096	0.012629366	0.009465785	*PTPRS*
chr5	155753336	155754265	*	5	0.006296295	0.039299687	0.012680442	*SGCD*
chr2	467799	468330	*	4	0.006298841	0.027398472	0.005187518	*NA*
chr12	1939964	1940652	*	6	0.006316245	-0.025830422	-0.008264538	*CACNA2D4;LRTM2*
chr8	143580965	143581481	*	3	0.006321081	-0.050235829	-0.035498697	*ADGRB1*
chr4	106816089	106817015	*	10	0.006326615	-0.01839231	-0.004716081	*NPNT*
chr16	81711959	81712102	*	3	0.006332359	0.053729345	0.031636215	*CMIP*
chr14	92587796	92588818	*	12	0.006334195	0.013200989	0.002556803	*CPSF2;NDUFB1*
chr6	3023598	3024178	*	6	0.006340352	-0.018896558	-0.006932732	*HTATSF1P2*
chr8	11302983	11303067	*	3	0.006341817	0.023259371	0.01803937	*FAM167A*
chr1	150551925	150552817	*	17	0.006357925	0.016199337	-0.00154206	*MCL1*
chr5	161274307	161275588	*	17	0.0063673	-0.012108353	-0.005053591	*GABRA1*
chr10	13424851	13425688	*	6	0.006381208	-0.020989313	-0.012017446	*NA*
chr8	6665912	6666452	*	3	0.006381208	0.026534545	0.012796322	*XKR5*
chr17	4900256	4901584	*	18	0.006387514	0.022241432	0.000898204	*INCA1;KIF1C*
chr7	157451243	157451618	*	4	0.006394197	0.022682959	0.00408384	*PTPRN2*
chr19	5752188	5753221	*	3	0.006394358	0.021992492	0.009862964	*CATSPERD*
chr12	6874105	6875642	*	9	0.006397703	0.035666938	-0.002146202	*MLF2;PTMS*
chr1	47691301	47691845	*	6	0.00641148	-0.026977785	-0.006006681	*TAL1*
chr19	55549414	55550348	*	11	0.006415298	0.033576906	0.021400132	*GP6;RDH13*
chr4	73178533	73179086	*	3	0.006426401	0.018292472	0.005290326	*ADAMTS3*
chr6	42927940	42928773	*	25	0.00643739	-0.022363948	-0.007355975	*GNMT*
chr16	87492071	87492801	*	5	0.006449139	0.015727015	-0.000800119	*ZCCHC14*
chr17	58225949	58227745	*	11	0.006458652	0.026220716	0.000246978	*CA4*
chr7	116592956	116594430	*	21	0.006462068	0.02502798	-0.001477084	*ST7;ST7-AS1;ST7-OT4*
chr5	133772283	133773142	*	5	0.006472413	-0.01983199	-0.009345467	*NA*
chr8	145024637	145025178	*	7	0.006477839	0.024645005	0.011303763	*PLEC*
chr12	39538604	39539558	*	6	0.006479627	0.019374437	0.016246767	*NA*
chr2	239036809	239037533	*	6	0.006479656	-0.039916903	-0.024482411	*ESPNL*
chr14	24550845	24551212	*	6	0.00648876	0.027231777	0.010364726	*NRL*
chr1	3620638	3621445	*	5	0.006501995	-0.027947903	-0.016117193	*TP73*
chr2	97651765	97652806	*	9	0.006529285	0.025596862	0.007291823	*FAM178B*
chr16	1198408	1199222	*	6	0.006539868	0.017075478	0.008046679	*CACNA1H*
chr11	59210634	59211431	*	3	0.006544938	0.021258318	0.008789848	*OR5A1*
chr1	151019727	151020868	*	11	0.006570192	0.009607081	0.002146511	*BNIPL;C1orf56*
chr1	56724194	56725058	*	5	0.006574638	0.024437059	0.010331429	*NA*
chr10	103879827	103880419	*	9	0.006577668	-0.008019827	-0.004184326	*LDB1*
chr3	11195673	11196445	*	7	0.006582668	-0.021148399	0.000541813	*HRH1*
chr6	32036530	32038155	*	27	0.006591555	0.022878852	0.005356237	*TNXB*
chr7	100239172	100239764	*	4	0.006593109	-0.025556506	-0.013140158	*TFR2*
chr7	22893924	22894898	*	8	0.006597785	0.019049135	0.005308693	*SNORD93*
chr16	18812258	18813278	*	14	0.006600905	-0.031417926	-0.003450229	*ARL6IP1*
chr6	101846791	101847656	*	13	0.006601991	0.024788078	0.002787613	*GRIK2*
chr5	72596701	72597316	*	6	0.006601991	-0.015727545	-0.00788054	*NA*
chr17	27070369	27071119	*	9	0.006603717	0.027942496	0.002375077	*TRAF4*
chr21	19858957	19859472	*	3	0.006617658	0.019347596	0.001996817	*NA*
chr4	89642483	89642760	*	3	0.006619128	0.013640596	0.008679472	*FAM13A-AS1*

chr2	236504134	236504635	*	3	0.006632354	-0.016980722	-0.008775778	*AGAP1*
chr19	13133343	13133825	*	4	0.006644878	-0.019227921	-0.010886422	*NFIX*
chr6	32820355	32822043	*	45	0.006648488	-0.029626704	-0.00153454	*PSMB9;TAP1*
chr12	32111966	32113101	*	13	0.006653562	-0.024793478	-0.002155175	*RESF1*
chr1	14219640	14220614	*	7	0.006653562	-0.035607078	-0.007006692	*NA*
chr1	9970042	9971115	*	16	0.006659918	-0.02115584	-0.005711814	*CTNNBIP1*
chr21	44072610	44073555	*	5	0.006664863	0.020847216	0.008236089	*PDE9A*
chr7	1057285	1057944	*	4	0.006667486	0.029890063	0.016278403	*C7orf50*
chr19	11093459	11094931	*	8	0.006677984	0.012566448	-0.001132179	*SMARCA4*
chr16	426536	427756	*	6	0.006688625	0.030015267	0.010256582	*LOC100134368;PGAP6*
chr11	119055760	119056215	*	3	0.006688625	-0.019469356	0.005213119	*PDZD3*
chr10	114911442	114912149	*	6	0.006691019	-0.017197279	-0.009897289	*TCF7L2*
chr14	74352920	74353709	*	11	0.006692778	0.018664349	0.002317824	*ZNF410*
chr10	103648988	103649718	*	3	0.006692778	-0.032728066	-0.00477993	*ARMH3*
chr15	91499369	91500193	*	6	0.006701219	0.030800007	0.019167047	*RCCD1*
chr9	90112086	90114156	*	21	0.006723832	-0.020498677	-0.005728172	*DAPK1*
chr6	28602513	28603437	*	34	0.006730098	-0.027361498	-0.002707009	*NA*
chr4	171011099	171011760	*	15	0.006752832	-0.016790705	-0.002457716	*AADAT*
chr1	158223034	158223934	*	5	0.006773501	-0.046446769	-0.008441203	*CD1A*
chr19	3162628	3163454	*	7	0.00679015	0.023242133	0.012135477	*GNA15*
chr3	194868427	194868843	*	6	0.006794637	-0.033494415	-0.015609631	*XXYLT1;XXYLT1-AS2*
chr4	101596281	101596970	*	6	0.006798355	0.031568376	0.01410541	*LINC01216*
chr5	42423947	42425093	*	13	0.006806946	-0.020826152	-0.006048988	*GHR*
chr8	143915496	143916385	*	11	0.006806946	0.041054901	0.014141221	*GML*
chr17	46018654	46019184	*	14	0.006814354	-0.024040173	-0.006886146	*PNPO*
chr19	14196326	14197848	*	12	0.006820979	0.036611319	0.009019095	*C19orf67*
chr5	16737972	16738723	*	5	0.006822347	0.024545045	0.013065608	*MYO10*
chr22	30818470	30819402	*	4	0.006824119	-0.011545593	-0.000457666	*MTFP1;SEC14L2*
chr11	63764177	63764925	*	5	0.006827473	-0.015202502	-0.002078004	*OTUB1*
chr12	7818585	7819180	*	4	0.006827473	-0.035795637	0.007572476	*APOBEC1*
chr16	11369940	11370688	*	10	0.006827778	0.027930982	0.009918114	*PRM2;PRM3;RMI2*
chr8	145747659	145748901	*	6	0.006830009	0.02266069	0.003960548	*LRRC14;LRRC24;RECQL4*
chr17	1268443	1269083	*	4	0.006837216	-0.026339451	-0.008238117	*YWHAE*
chr16	85074359	85075249	*	6	0.006839347	0.032826677	0.005336808	*KIAA0513*
chr13	28024329	28024921	*	16	0.006862052	-0.028609138	0.000156386	*MTIF3*
chr14	104195181	104196038	*	6	0.006867779	0.027488202	-0.004913069	*ZFYVE21*
chr9	140195998	140197048	*	8	0.006868233	-0.014687067	-0.001500532	*NRARP*
chr12	4140243	4140993	*	4	0.006874935	0.017893227	0.004557169	*NA*
chr9	138436220	138437177	*	3	0.0068764	0.020351268	0.007824418	*OBP2A*
chr6	168122077	168123805	*	9	0.006884677	-0.04601952	0.001047006	*NA*
chr20	36662217	36663290	*	5	0.006888669	0.024498967	0.007179377	*RPRD1B;TTI1*
chr2	3496821	3497214	*	3	0.006938171	0.028948303	-0.000555259	*NA*
chr4	159592191	159593775	*	18	0.006940216	-0.011000518	-0.001548961	*C4orf46;ETFDH*
chr4	21305490	21306181	*	8	0.006945821	-0.020741544	-0.006917072	*KCNIP4*
chr2	239432223	239432329	*	3	0.006957554	-0.035856098	-0.025955557	*LINC01107*
chr9	136075279	136076059	*	4	0.006970061	0.028350642	-0.007575892	*NA*
chr18	77442923	77443784	*	3	0.006970061	-0.031416228	-0.024272818	*CTDP1*
chr17	10600507	10602034	*	16	0.006970096	-0.028972424	0.000444215	*ADPRM;SCO1*
chr7	122527351	122527522	*	4	0.006970535	-0.013435309	-0.006183421	*CADPS2*
chr15	89455860	89456239	*	3	0.006975725	-0.036742216	-0.014029093	*MFGE8*
chr3	196755973	196756877	*	6	0.00699477	-0.025939733	-0.008571144	*MELTF*
chr2	95831031	95831943	*	10	0.007019807	-0.013081779	-0.002104868	*ZNF2*
chr5	175298571	175298869	*	3	0.007045222	-0.035224905	-0.013551148	*CPLX2*
chr14	96505296	96505874	*	13	0.007051786	-0.010137051	-0.000447002	*C14orf132*
chr5	140718283	140719142	*	10	0.007062731	-0.027954479	-0.006137542	*PCDHGA1;PCDHGA2;PCDHGA3*
chr12	52827847	52828840	*	8	0.00707535	0.021765649	0.001633142	*KRT75*
chr15	52861545	52862406	*	8	0.007082289	0.015853492	0.004046584	*ARPP19*
chr17	4692162	4692790	*	5	0.007088078	0.030862945	0.010111291	*GLTPD2;VMO1*
chr1	27989987	27990967	*	3	0.007101192	0.01595944	0.000555753	*NA*
chr9	95857577	95858671	*	13	0.007110248	-0.034389509	-0.007346657	*CARD19*
chr3	167813835	167814230	*	5	0.00711108	-0.005620645	-0.00233205	*GOLIM4*
chr1	160681404	160682655	*	9	0.007120874	-0.01087152	-0.005765485	*CD48*
chr6	75982940	75983278	*	3	0.007127134	-0.01750491	-0.015766057	*TMEM30A*
chr7	157579211	157579880	*	3	0.007135693	0.021397262	0.015937989	*PTPRN2*
chr1	68150193	68151160	*	9	0.007136745	-0.00960796	-0.002358654	*GADD45A*
chr1	38259884	38260410	*	5	0.007144141	0.011684588	0.003745892	*MANEAL*

chr16	89005476	89006877	*	11	0.007147894	-0.020093333	-0.005133275	*CBFA2T3*
chr8	41167278	41168186	*	9	0.007187199	-0.031822188	-0.003386987	*SFRP1*
chr9	135285195	135286214	*	11	0.007197487	0.023820155	0.003381464	*CFAP77;TTF1*
chr18	56886915	56887785	*	12	0.007213202	-0.012021943	-0.005066046	*GRP*
chr2	45236267	45237431	*	9	0.007221844	-0.011571203	-0.001835958	*SIX2*
chr3	52091063	52091829	*	6	0.007242981	0.014632564	0.001641669	*DUSP7*
chr13	79169714	79171230	*	14	0.007247149	-0.0179797	-0.003177439	*OBI1-AS1*
chr20	1246100	1247058	*	9	0.007248175	0.043969624	0.010555	*SNPH*
chr7	142630597	142631266	*	5	0.007263024	0.015301769	0.008371306	*TRPV5*
chr9	138985942	138986379	*	4	0.007266051	0.017731575	0.009571731	*NACC2*
chr4	150998949	150999949	*	15	0.007271705	-0.011865669	-0.003296367	*DCLK2*
chr17	79633202	79634204	*	16	0.00729943	-0.01457236	-0.003347523	*CCDC137;OXLD1*
chr12	51717674	51719349	*	14	0.007314773	-0.04011549	-0.009058176	*BIN2*
chr22	39953557	39954239	*	5	0.00731919	0.0233839	-0.000777304	*NA*
chr10	73516760	73517463	*	5	0.007350087	-0.020748568	-0.012294563	*CDH23;VSIR*
chr12	14132940	14133887	*	9	0.007357326	-0.032402116	-0.004266192	*GRIN2B*
chr7	2646782	2648785	*	12	0.007374193	0.024859322	0.005280171	*IQCE*
chr10	90640622	90641389	*	3	0.007383476	0.019742746	0.00282255	*STAMBPL1*
chr19	49149933	49150388	*	3	0.007389498	0.012834776	0.001568976	*CA11;SEC1P*
chr15	91497412	91498367	*	11	0.007395313	-0.01751918	-0.001568275	*RCCD1*
chr7	128864205	128864907	*	6	0.007395683	-0.007494367	-0.000236375	*AHCYL2*
chr16	12995603	12996419	*	6	0.00739644	-0.016651241	-0.005858924	*SHISA9*
chr12	133405852	133406309	*	3	0.007396854	-0.013471117	-0.001777497	*GOLGA3*
chr8	144922348	144923606	*	9	0.007399524	-0.038988989	-0.008666314	*NRBP2*
chr2	239046275	239047336	*	8	0.007404473	0.028669133	0.007593885	*KLHL30*
chr19	35629273	35630651	*	17	0.00740831	0.02312439	0.002326144	*FXYD1;FXYD7;LGI4*
chr3	172859699	172860467	*	4	0.007412295	0.043220935	0.015455402	*SPATA16*
chr2	129202959	129203409	*	3	0.007414928	0.025147445	0.009634568	*NA*
chr15	71952725	71953221	*	3	0.007414928	0.015375002	0.012925101	*THSD4*
chr5	125514187	125514911	*	3	0.007425751	0.025322404	0.000694155	*NA*
chr12	116756805	116756948	*	3	0.007427494	-0.020649333	-0.010970685	*NA*
chr6	33283770	33285470	*	24	0.007434326	0.015995916	0.005494177	*TAPBP;ZBTB22*
chr10	88728947	88729861	*	7	0.007446092	0.026667448	0.01314635	*ADIRF;AGAP11*
chr5	157001092	157002175	*	5	0.007451956	0.026810893	0.008026186	*ADAM19*
chr11	18416535	18418093	*	12	0.007457488	-0.009127717	-0.001696838	*LDHA*
chr6	30657882	30659043	*	20	0.007459369	-0.031559188	-0.003195452	*NRM;PPP1R18*
chr19	17830116	17831088	*	11	0.007462633	-0.013427724	-0.00393098	*MAP1S*
chr8	13423496	13424483	*	10	0.007462836	-0.013675802	-0.003597934	*C8orf48*
chr1	166853278	166853960	*	6	0.007463833	0.019370489	0.005365686	*NA*
chr15	89560878	89561135	*	3	0.007466691	-0.031682771	-0.009386004	*NA*
chr2	45159663	45161333	*	13	0.007477544	-0.027290224	-0.007073125	*NA*
chr3	126242379	126243401	*	13	0.007480481	-0.022973069	0.001214413	*CHST13*
chr18	24764966	24765880	*	11	0.007481814	-0.018644169	-0.008443693	*AQP4-AS1;CHST9*
chr22	38851884	38852154	*	4	0.007489488	-0.017637069	-0.012034453	*KCNJ4*
chr1	34641601	34642609	*	12	0.007491787	0.023682886	0.010901916	*C1orf94*
chr20	57267176	57267680	*	7	0.007500868	0.030077834	0.016370234	*NPEPL1*
chr5	34007809	34008663	*	14	0.007510412	-0.028468761	-0.005006052	*AMACR*
chr1	4832380	4832865	*	5	0.007512025	0.018669365	0.012815455	*AJAP1*
chr5	133450709	133452188	*	11	0.007521281	0.01971501	0.002472368	*TCF7*
chr6	41374532	41375153	*	4	0.007534749	0.050549935	0.031350482	*NA*
chr19	12901966	12902806	*	8	0.007539824	-0.007995591	-0.00344583	*JUNB*
chr1	119531857	119532925	*	14	0.007553275	0.026473831	0.000215977	*TBX15*
chr7	751830	753007	*	15	0.007567225	-0.049538186	-0.003619059	*PRKAR1B*
chr9	139096080	139096889	*	5	0.007567225	-0.03409565	-0.014585973	*LHX3*
chr9	125795488	125796611	*	8	0.007581751	-0.015756381	-0.010240707	*GPR21;RABGAP1*
chr4	172733760	172734843	*	13	0.00758248	-0.038315158	-0.008704695	*GALNTL6*
chr18	10414355	10414962	*	4	0.007584474	0.016863735	0.013477621	*NA*
chr20	62130111	62131189	*	7	0.007604211	-0.030459379	-0.011102257	*EEF1A2*
chr10	3145966	3146549	*	6	0.007606421	0.020962629	0.010688058	*PFKP*
chr22	41346948	41348222	*	9	0.007609622	-0.017665246	-0.003419521	*RBX1;XPNPEP3*
chr16	1349178	1349861	*	5	0.007612885	0.020536638	0.003987196	*NA*
chr14	35590656	35591840	*	20	0.007627647	0.012742182	9.39E-05	*PPP2R3C;PRORP*
chr3	64430390	64431038	*	7	0.007632054	-0.015592364	-0.003413822	*NA*
chr19	51980685	51980908	*	3	0.007632054	-0.067249187	-0.034346322	*CEACAM18*
chr2	121411622	121412432	*	5	0.007653683	-0.03461369	-0.01803478	*NA*
chr13	53312966	53313529	*	3	0.007664958	-0.007440138	-0.002943065	*CNMD*

chr1	208041879	208042623	*	5	0.007665102	-0.013261763	-0.001462388	*NA*
chr14	37641191	37642356	*	12	0.007674999	0.03257627	-0.001053433	*SLC25A21;SLC25A21-AS1*
chr1	203241948	203242950	*	9	0.007695809	0.021610465	0.004258889	*CHIT1*
chr3	143691715	143692356	*	12	0.007753037	0.016192009	0.003560512	*DIPK2A*
chr3	148804272	148805294	*	25	0.007763244	0.022829111	0.002118098	*HLTF;HLTF-AS1*
chr5	73937141	73937762	*	8	0.007763244	0.01627545	0.000712006	*ENC1;HEXB*
chr22	36903611	36904394	*	3	0.007763244	0.017423597	-0.003409226	*FOXRED2*
chr8	6785692	6786083	*	6	0.00777264	-0.04226664	-0.006032297	*DEFA6*
chr17	79225309	79226323	*	7	0.007791106	-0.034336811	-0.011055029	*SLC38A10*
chr17	6347791	6348225	*	7	0.007791106	0.037714156	0.008268248	*PIMREG*
chr17	73043062	73043736	*	10	0.007823836	-0.010398315	-0.00071943	*ATP5PD;KCTD2*
chr21	45936193	45937406	*	8	0.007833672	-0.026446833	-0.004389876	*TSPEAR;TSPEAR-AS2*
chr10	128994297	128995478	*	11	0.007837608	-0.024409878	-0.010776012	*DOCK1;INSYN2A*
chr2	233739771	233740203	*	3	0.007837608	0.01760348	0.006679865	*SNORC*
chr19	14800564	14801245	*	10	0.007854796	-0.026130678	-0.005950252	*ZNF333*
chr7	151330008	151330288	*	5	0.007855446	-0.008266771	-0.005974838	*PRKAG2*
chr2	206546299	206547163	*	9	0.007856618	-0.016452874	-0.004336596	*NRP2*
chr2	175204249	175205716	*	5	0.00786044	-0.009850297	-0.000616299	*NA*
chr8	142401373	142403574	*	9	0.007868104	0.040385548	0.018250824	*NA*
chr19	4277333	4278055	*	4	0.007869259	0.035362351	0.00512388	*SHD*
chr11	6947759	6948211	*	5	0.007869919	0.016064403	-0.000534251	*ZNF215*
chr1	1099630	1100557	*	7	0.007905924	0.032461413	0.017305351	*MIR200A;MIR200B;MIR429*
chr2	26986235	26986550	*	5	0.007912581	-0.023235652	0.002435292	*SLC35F6*
chr11	69456412	69457121	*	5	0.007938659	-0.008824265	-0.003999349	*CCND1*
chr17	59476790	59477846	*	14	0.007964104	0.026289972	-0.001086315	*TBX2*
chr9	132934195	132935030	*	8	0.007973025	0.017746495	-0.002453656	*NCS1*
chr16	595486	596185	*	8	0.007977565	0.025393404	0.012733758	*CAPN15*
chr4	1736433	1737129	*	4	0.007977698	0.018204293	0.013672737	*TACC3*
chr1	226288408	226288782	*	3	0.007981616	-0.020946235	-0.007021031	*NA*
chr2	118980919	118981783	*	5	0.007991824	0.022311955	-0.002328692	*NA*
chr1	247653903	247654811	*	6	0.007993337	0.018999939	0.006894012	*OR2W5P*
chr6	33178942	33180841	*	16	0.007994361	0.026265218	0.003869618	*RING1*
chr2	1291410	1291494	*	3	0.007999453	0.035262548	0.007898514	*SNTG2*
chr7	92076509	92077858	*	11	0.008009014	-0.021470313	-0.00260966	*GATAD1*
chr16	62070306	62071129	*	9	0.008009014	-0.018023759	-0.003602689	*CDH8*
chr18	78005180	78006141	*	12	0.008014422	-0.050891394	-0.010194208	*PARD6G*
chr11	17097203	17097511	*	3	0.008014511	0.019201351	0.003686319	*RPS13*
chr13	95364333	95364993	*	13	0.008020643	-0.008766522	-0.00336444	*SOX21*
chr1	155880507	155881569	*	9	0.008030728	-0.035566981	-0.005160604	*RIT1*
chr16	12096413	12096947	*	3	0.008033773	-0.027763547	-0.006386535	*SNX29*
chr5	2751822	2752882	*	12	0.008042856	-0.019330519	-0.008472496	*C5orf38;IRX2*
chr16	1160642	1160722	*	3	0.008042856	-0.032616077	-0.013944598	*NA*
chr7	2149259	2150016	*	4	0.008046205	-0.015608355	0.001135409	*MAD1L1*
chr5	138629998	138630502	*	5	0.008049882	-0.038427228	-0.005910693	*MATR3*
chr11	76571458	76571610	*	5	0.008056623	0.020048438	0.006595271	*ACER3*
chr16	31558017	31558891	*	3	0.008061308	0.015123132	0.012145777	*NA*
chr1	1377525	1378060	*	4	0.008065176	0.027528823	0.013481801	*VWA1*
chr12	133185615	133185655	*	3	0.008065176	0.0285389	0.024514796	*LRCOL1*
chr11	71725245	71725590	*	4	0.008066855	-0.013130544	-0.010000034	*NUMA1*
chr1	45805261	45807214	*	23	0.008071168	0.015770935	-0.000126356	*MUTYH;TOE1*
chr6	3160502	3160634	*	4	0.008074953	-0.023847373	-0.018478361	*TUBB2A*
chr11	58980377	58980665	*	3	0.008086991	-0.024968989	-0.013787844	*MPEG1*
chr6	21593587	21594320	*	10	0.008087312	-0.027530892	-0.001115231	*SOX4*
chr14	105766064	105767520	*	17	0.008094806	-0.020824176	-0.004037513	*BRF1;PACS2*
chr13	20692403	20693122	*	6	0.008101773	-0.032988158	0.007083346	*NA*
chr18	22932076	22932673	*	5	0.008101773	-0.009138286	-0.00411721	*ZNF521*
chr2	149894539	149895362	*	7	0.008103758	0.033317013	0.01183519	*LYPD6B*
chr11	94500612	94500965	*	3	0.008103758	0.03164574	0.019991656	*AMOTL1*
chr2	206627521	206628773	*	12	0.008105759	0.042102516	0.019974143	*NRP2*
chr5	146256776	146257484	*	4	0.008121305	0.006912674	-0.000452736	*PPP2R2B*
chr3	74662850	74663024	*	3	0.008122101	0.028271298	0.021282272	*NA*
chr5	33440240	33441304	*	13	0.008128017	-0.016855577	-0.001097088	*TARS1*
chr9	37029364	37030262	*	4	0.008135031	0.01782029	0.003598777	*PAX5*
chr19	52263233	52264429	*	7	0.008145043	-0.027567668	-0.000783526	*FPR2*
chr11	60282398	60282972	*	12	0.008145564	0.028245465	0.010836232	*MS4A13*
chr11	45672100	45673410	*	9	0.008169198	-0.03578356	-0.003497661	*CHST1*

chr20	44746392	44747351	*	9	0.008177555	-0.019797166	-0.006120395	*CD40*
chr2	74153795	74154363	*	10	0.008184584	-0.013667211	-0.00372982	*DGUOK*
chr13	113928287	113929010	*	3	0.008190105	-0.019689112	-0.007788617	*NA*
chr1	203320297	203321087	*	10	0.008191524	-0.030599525	-0.020739448	*FMOD*
chr1	1981657	1983033	*	7	0.008227276	0.045937578	0.012026338	*PRKCZ*
chr1	197871470	197872227	*	11	0.00827366	-0.011484669	-0.000724556	*C1orf53*
chr11	117703496	117704267	*	3	0.00827579	0.031207541	0.009295486	*FXYD2;FXYD6-FXYD2*
chr3	127006287	127006803	*	4	0.008295151	-0.018224167	0.002236808	*NA*
chr16	1034059	1034527	*	3	0.008298076	0.025040527	0.019704881	*LMF1;SOX8*
chr14	23503697	23504921	*	19	0.008300777	-0.016143354	-0.003849143	*PSMB5*
chr3	196594273	196595444	*	11	0.008318586	-0.028624509	-0.00426637	*SENP5*
chr19	33622856	33623230	*	10	0.008318586	0.028572112	0.016645512	*WDR88*
chr5	132112449	132112975	*	7	0.008318586	0.010457986	0.000632762	*SEPTIN8*
chr13	114282093	114282491	*	3	0.008318586	0.013601453	0.01277746	*TFDP1*
chr8	59571055	59571961	*	5	0.008322249	0.006087701	-0.000589082	*NSMAF*
chr17	45918221	45919419	*	11	0.00832315	0.026222396	0.001041195	*SCRN2*
chr14	25045625	25046267	*	6	0.008324433	-0.018392495	-0.006167425	*CTSG*
chr7	48075568	48076004	*	5	0.008326226	-0.015406858	-0.008631762	*C7orf57*
chr15	60285234	60287537	*	11	0.008336859	0.021044389	0.004164242	*NA*
chr12	124414964	124415398	*	3	0.008337979	0.020475613	0.017123823	*DNAH10*
chr13	111317935	111318640	*	5	0.00835265	-0.016187515	-0.012254162	*CARS2*
chr12	54354162	54354590	*	3	0.008356036	-0.026126374	-0.015707286	*NA*
chr11	73498113	73498948	*	8	0.008367236	0.024657566	0.007145731	*MRPL48*
chr17	48132714	48133402	*	8	0.008371233	-0.013710185	-0.005602081	*ITGA3;PICART1*
chr12	34755568	34756440	*	5	0.008371233	-0.02228955	-0.019172617	*NA*
chr6	42878931	42879409	*	3	0.008385696	0.031084304	0.019578175	*PTCRA*
chr21	38362230	38363160	*	9	0.008390185	0.050519275	0.01796048	*HLCS*
chr18	3262499	3263082	*	8	0.008416671	-0.026447243	-0.005245314	*MYL12B*
chr19	17531239	17531746	*	3	0.008426388	0.020505445	0.006115413	*MVB12A*
chr5	138289138	138289233	*	3	0.008430498	-0.007516429	-0.005882973	*SIL1*
chr17	74524371	74525597	*	6	0.008437772	0.018831735	0.007538448	*CYGB;PRCD*
chr4	8588716	8589313	*	6	0.008441867	0.026746096	0.013200911	*CPZ;GPR78*
chr17	79952142	79952487	*	3	0.008441867	0.028460565	0.013776868	*ASPSCR1*
chr19	44668862	44669612	*	13	0.00844547	-0.024866471	-0.006333557	*ZNF226*
chr10	94607768	94608332	*	11	0.008469451	0.023043849	-0.00322636	*EXOC6*
chr11	78672632	78673290	*	5	0.008473029	0.020510491	0.000125772	*TENM4*
chr5	150325582	150326642	*	11	0.008482686	-0.020876094	-0.003210582	*ZNF300P1*
chr2	220264753	220265305	*	4	0.008496258	-0.01825112	-0.009475871	*DNPEP*
chr6	31368461	31369119	*	3	0.008496734	-0.032640233	-0.015021247	*MICA*
chr11	34580349	34580491	*	3	0.008497643	0.037051009	0.024385929	*NA*
chr1	21877524	21877781	*	5	0.008499527	0.054197187	0.015584069	*ALPL*
chr14	103243389	103244199	*	3	0.008499527	-0.049441522	-0.020719442	*TRAF3*
chr22	50515323	50515985	*	4	0.008522135	0.027053577	0.015271327	*MLC1*
chr22	47022471	47023209	*	8	0.008525535	0.037107266	0.01372117	*GRAMD4*
chr11	66631012	66631894	*	6	0.008525535	0.029745298	0.009782165	*PC*
chr13	107001538	107001893	*	4	0.008543648	-0.014282376	-0.006983627	*NA*
chr15	72978347	72979048	*	13	0.008578116	-0.016479031	-3.63E-06	*BBS4;HIGD2B*
chr7	139320346	139320964	*	5	0.008594558	-0.020124567	-0.013715319	*HIPK2*
chr19	10120386	10121323	*	7	0.008594768	-0.023843137	-0.00616789	*COL5A3;RDH8*
chr3	52001778	52002969	*	9	0.00859637	-0.04230244	-0.008218717	*ABHD14B;PCBP4*
chr13	114291735	114292740	*	11	0.008622795	0.015436737	0.000863617	*TFDP1*
chr7	19812570	19813525	*	15	0.008624604	-0.01573788	-0.005540911	*TMEM196*
chr1	32665582	32666481	*	11	0.008629746	-0.014870058	-0.002120736	*CCDC28B;IQCC*
chr7	100025489	100026182	*	4	0.008640789	0.019226131	0.005121358	*MEPCE;ZCWPW1*
chr11	7535611	7535983	*	4	0.00866702	-0.038043503	-0.020282835	*PPFIBP2*
chr1	151689203	151690075	*	10	0.008668834	-0.025584055	-0.010169207	*CELF3;RIIAD1*
chr2	241807924	241808757	*	7	0.008676328	-0.017378026	0.003772825	*AGXT*
chr7	28998009	28998860	*	11	0.008682039	-0.01772376	-0.001217955	*TRIL*
chr19	4832695	4832993	*	3	0.008698583	0.025699153	0.008017035	*TICAM1*
chr2	219154857	219155790	*	5	0.008700807	0.011050642	0.006115779	*PNKD;TMBIM1*
chr2	68546467	68547655	*	16	0.008713325	-0.019845877	-0.004148175	*CNRIP1*
chr8	85097031	85097429	*	6	0.008713325	-0.044578092	-0.008523606	*RALYL*
chr2	218712695	218712871	*	4	0.008739714	-0.013939361	-0.001321486	*TNS1*
chr19	3960982	3961765	*	12	0.008744412	0.02605262	0.009588999	*DAPK3;MIR637*
chr1	33336618	33337583	*	5	0.008757113	0.03024698	0.01410008	*FNDC5*
chr10	81904244	81904814	*	3	0.008766822	-0.012321333	-0.010430406	*PLAC9*

chr6	7142024	7142638	*	5	0.008773128	0.023918856	0.015938608	*RREB1*
chr6	28863395	28864832	*	30	0.00877868	-0.03377355	-0.004176422	*NA*
chr5	180625089	180625956	*	7	0.008792188	0.025672072	0.005182481	*TRIM7*
chr20	52789646	52791541	*	15	0.008799081	-0.039208872	-0.005441161	*CYP24A1*
chr7	4814154	4814713	*	3	0.008813206	0.013665549	0.008447474	*AP5Z1*
chr14	34931026	34931822	*	7	0.008816105	-0.03661761	-0.013661976	*SPTSSA*
chr1	55464084	55465282	*	9	0.008835969	0.016961862	0.009200849	*BSND*
chr14	34529059	34529242	*	3	0.008836367	0.040167392	0.019876264	*NA*
chr13	28395271	28396059	*	7	0.008847177	-0.023428513	-0.008384611	*NA*
chr14	23478970	23479801	*	7	0.008869115	0.011828577	0.007228543	*C14orf93*
chr4	109540778	109542037	*	15	0.008871277	-0.019131957	-0.003598489	*RPL34;RPL34-DT*
chr2	102802929	102804405	*	11	0.008871277	0.028726114	0.003064161	*IL1RL2*
chr7	99005923	99006858	*	14	0.008878417	0.018076153	0.000550514	*BUD31;PDAP1*
chr12	6809127	6810055	*	15	0.008880929	-0.030683899	-0.00721823	*PIANP*
chr10	14995476	14996626	*	16	0.00888543	0.010176576	0.000634299	*DCLRE1C;MEIG1*
chr20	1471884	1472712	*	7	0.008887447	0.013181446	-0.006013614	*SIRPB2*
chr17	15848828	15849556	*	3	0.008898288	0.037508922	0.004473505	*ADORA2B*
chr6	31869518	31870840	*	27	0.008915258	-0.01500263	-0.002270733	*C2;EHMT2;ZBTB12*
chr12	3393711	3393872	*	3	0.008918111	0.02881371	0.009158709	*TSPAN9*
chr12	49688844	49689212	*	4	0.00893019	0.034369769	0.019944102	*PRPH*
chr21	42538388	42539633	*	7	0.008938139	0.031988645	0.005565033	*BACE2;MIR3197*
chr2	149855950	149856468	*	3	0.008944509	-0.021975456	-0.018493581	*KIF5C*
chr14	97091902	97092205	*	3	0.008944509	0.023144163	0.013664536	*NA*
chr14	73704007	73704343	*	5	0.008949549	0.025864181	0.008757616	*PAPLN*
chr11	2440032	2440386	*	3	0.008951296	0.018654606	0.014716597	*TRPM5*
chr16	73419299	73420528	*	5	0.008957003	0.030173729	0.014107052	*LINC01568*
chr6	31093846	31094557	*	5	0.008990778	0.017119784	0.008445181	*PSORS1C1*
chr12	125549535	125550542	*	14	0.008994711	-0.018411132	-0.004586151	*AACS*
chr14	36989588	36990534	*	9	0.008994711	0.012103167	-0.001439443	*NKX2-1;SFTA3*
chr11	101743355	101744168	*	5	0.008997833	0.032297488	0.004373047	*NA*
chr1	22140769	22141400	*	6	0.009005107	0.027273902	0.011017246	*LDLRAD2*
chr5	113697330	113698664	*	14	0.009007072	-0.014867941	-0.005956093	*KCNN2*
chr1	151319512	151320381	*	10	0.009012305	0.008169028	-0.000957884	*RFX5*
chr7	92861286	92861825	*	12	0.009046362	-0.023470488	-0.004554464	*VPS50*
chr8	61496287	61497168	*	4	0.009046649	-0.019958651	-0.00926934	*RAB2A*
chr15	41575421	41576837	*	14	0.009048268	0.017991371	0.00169477	*OIP5-AS1*
chr6	30644235	30644478	*	3	0.00906232	0.021785909	0.005900766	*DHX16;PPP1R18*
chr10	94831650	94834219	*	19	0.009076291	0.014885282	-0.000525959	*CYP26A1*
chr16	87090731	87091652	*	7	0.009076291	0.0231966	0.011991173	*NA*
chr8	41523177	41523968	*	4	0.009076291	-0.015325774	-0.006686825	*ANK1;NKX6-3*
chr15	102189106	102189763	*	3	0.009078263	-0.028613334	-0.010003173	*TM2D3*
chr15	99936927	99937249	*	3	0.009091231	-0.026289057	-0.012677651	*NA*
chr6	27776350	27776566	*	4	0.009097006	0.033502446	0.013443941	*H2BC13;H3C10*
chr8	9762716	9763159	*	4	0.009105386	-0.008352475	-0.003078797	*MIR124-1;MIR124-1HG*
chr16	57843964	57844572	*	8	0.009107613	-0.015563923	-0.005779005	*KIFC3;LOC388282*
chr22	31217230	31217665	*	3	0.009115649	0.016846275	0.013234803	*OSBP2*
chr4	13545639	13546673	*	12	0.00912193	-0.02356533	-0.007811775	*NKX3-2*
chr2	102927397	102927898	*	4	0.009138687	-0.021049967	-0.009271587	*IL18R1;IL1RL1*
chr3	14345227	14346268	*	6	0.009140907	0.033396617	0.014418948	*NA*
chr4	187064964	187066505	*	16	0.009152231	-0.030381157	-0.00578427	*FAM149A*
chr3	45076240	45077254	*	5	0.009160197	0.028987262	0.002779363	*CLEC3B*
chr16	1071049	1072056	*	4	0.009168299	0.027678981	0.018142234	*NA*
chr1	86047239	86048585	*	8	0.009170864	0.02792169	0.005762302	*CCN1;DDAH1*
chr9	93563033	93564033	*	12	0.009185936	-0.022900051	-0.004973692	*SYK*
chr10	3507462	3508379	*	3	0.009210031	-0.017265204	-0.004129602	*NA*
chr1	207818003	207819196	*	9	0.009219402	0.039244582	-0.000920038	*CR1L*
chr5	1886956	1888218	*	18	0.009231889	-0.048625753	-0.009320779	*IRX4*
chr2	196520930	196522377	*	18	0.009231889	0.026264173	0.00220968	*SLC39A10*
chr12	50355995	50357142	*	7	0.009231889	0.026873752	0.012513418	*AQP5;AQP6*
chr1	156675322	156675879	*	13	0.009234317	0.006729952	0.001102695	*CRABP2*
chr14	74100086	74100788	*	6	0.009236479	-0.027808154	-0.008951063	*NA*
chr3	195477854	195478715	*	5	0.009240171	0.020200592	0.013901563	*MUC4*
chr7	99691385	99691942	*	13	0.00924487	0.022059572	0.003082489	*MCM7;MIR106B;MIR25;MIR93*
chr16	85207569	85208316	*	3	0.009246786	0.012361076	0.010369206	*NA*
chr6	29910101	29911095	*	9	0.009252406	-0.023940895	0.005558357	*HLA-A;HLA-G;HLA-H;HLA-J*
chr12	56211383	56212269	*	17	0.009257843	0.017997136	-0.001264997	*DNAJC14;ORMDL2;SARNP*

chr16	85783863	85785218	*	7	0.009272857	0.027431389	0.005561107	*C16orf74*
chr19	54693755	54694791	*	16	0.009275111	-0.024875829	-0.004882145	*MBOAT7;TSEN34*
chr8	1897020	1897487	*	4	0.009277949	-0.030697303	-0.015985906	*ARHGEF10*
chr16	75894736	75895031	*	5	0.009288021	-0.041396733	-0.02771533	*NA*
chr12	111618925	111619455	*	8	0.009304416	-0.038788319	-0.012905898	*CUX2*
chr22	38507451	38508132	*	3	0.009304416	-0.018601838	0.000797524	*BAIAP2L2;PLA2G6*
chr3	138763424	138764372	*	12	0.009317037	0.048911677	0.012927687	*PRR23C*
chr2	220282914	220284395	*	9	0.009317037	0.038994379	0.011637096	*DES*
chr2	108994116	108994528	*	7	0.009317037	-0.016686238	-0.010305073	*SULT1C4*
chr3	38691243	38691506	*	6	0.009320811	0.022270775	-0.001038256	*SCN5A*
chr19	37997105	37997867	*	14	0.009342713	-0.013018764	-0.004325528	*ZNF793*
chr13	79980322	79980793	*	9	0.009366289	-0.007414514	-0.001812243	*RBM26;RBM26-AS1*
chr6	139694265	139694674	*	4	0.009370039	-0.008159412	-0.002622424	*CITED2*
chr15	89973756	89974397	*	6	0.009377354	-0.025365251	-0.008011849	*NA*
chr3	50649994	50650410	*	3	0.009380589	0.040505962	0.020887155	*CISH;MAPKAPK3*
chr14	77964712	77965757	*	8	0.009396011	-0.017102581	-0.005212562	*ISM2*
chr16	21170107	21170814	*	5	0.009396011	0.011601088	-0.001258483	*DNAH3;TMEM159*
chr1	45138784	45140266	*	10	0.009405985	0.021006833	0.004302785	*ARMH1;TMEM53*
chr15	67546975	67548075	*	15	0.009407815	-0.010939514	-0.001061121	*AAGAB;IQCH*
chr11	17297732	17298537	*	10	0.009419193	-0.018839149	-0.005640405	*NUCB2*
chr14	58618986	58619480	*	11	0.009438936	0.012013575	0.000645461	*ARMH4*
chr3	49130732	49131405	*	7	0.009438936	-0.032483137	-0.006111613	*QRICH1*
chr3	49315355	49316322	*	4	0.009452414	0.013516469	0.006885686	*C3orf62;USP4*
chr16	79804561	79805381	*	6	0.009459588	0.011521049	0.000878614	*NA*
chr21	34755681	34756016	*	4	0.009459588	-0.006264878	-0.00441657	*NA*
chr18	77960683	77960807	*	4	0.009459588	0.030906473	0.017004222	*PARD6G*
chr7	8481994	8483374	*	9	0.009461984	-0.022151444	-0.004216268	*NXPH1*
chr1	45476456	45477041	*	7	0.00946718	0.010786987	0.000761986	*HECTD3;UROD*
chr16	69166316	69168101	*	15	0.009471688	-0.024108017	-0.003003126	*CHTF8;UTP4*
chr6	31670195	31671391	*	36	0.009477675	0.025210936	-0.000194526	*ABHD16A;LY6G6F;MIR4646*
chr1	111748053	111748268	*	3	0.009482229	-0.021912874	-0.011949566	*DENND2D*
chr17	40260053	40260548	*	5	0.009510099	0.017926822	0.002708865	*DHX58*
chr17	72897623	72898015	*	4	0.009522119	-0.018578003	-0.014224271	*NA*
chr17	80372912	80373608	*	7	0.009525635	0.026382764	0.00799865	*HEXD;OGFOD3*
chr1	206729034	206729685	*	7	0.009542417	0.023378109	0.006404335	*RASSF5*
chr20	4666423	4666916	*	9	0.009552185	0.016852619	0.001647684	*PRNP*
chr8	110693195	110693461	*	4	0.009564908	-0.038623213	-0.007747142	*SYBU*
chr17	29151515	29152249	*	7	0.009569072	0.017316281	-0.002475058	*CRLF3*
chr2	136577303	136577747	*	5	0.009571749	-0.015148764	-0.005159125	*LCT;LCT-AS1*
chr8	12990213	12991259	*	12	0.009576175	-0.023549485	-0.007826822	*DLC1*
chr12	10324843	10325287	*	5	0.009582659	0.03465219	-0.002050179	*OLR1*
chr19	6425207	6425737	*	4	0.00960154	0.016853504	0.00333132	*KHSRP*
chr19	44711560	44711915	*	4	0.009602855	-0.007403618	-0.006231823	*ZNF227*
chr3	126750108	126751404	*	4	0.009616411	0.021715203	0.003532147	*PLXNA1*
chr13	77900505	77901478	*	12	0.009632255	-0.017812587	-0.004728204	*MYCBP2*
chr5	92909429	92910052	*	4	0.009636473	-0.014361744	-0.010855904	*NR2F1-AS1*
chr15	28341068	28342183	*	9	0.009642893	0.018762546	0.001451889	*OCA2*
chr19	38713050	38713373	*	4	0.009654411	-0.03159306	-0.018232213	*DPF1*
chr17	4853232	4853717	*	5	0.009657025	-0.01726607	-0.002811184	*ENO3;PFN1*
chr17	73641675	73642080	*	3	0.009657025	0.026823088	0.014682792	*RECQL5;SMIM6*
chr7	1990554	1992159	*	10	0.00966625	0.020408334	0.009821288	*MAD1L1*
chr6	35181888	35182910	*	5	0.009677302	0.015947116	0.005820406	*SCUBE3*
chr18	30051012	30051803	*	19	0.009691996	-0.017606414	-0.002816583	*GAREM1*
chr1	193090350	193091399	*	11	0.009702055	-0.017005023	-0.000557233	*CDC73*
chr9	124009589	124009784	*	3	0.009717649	-0.019840918	-0.012546498	*GSN*
chr16	3311857	3313282	*	4	0.009738115	0.032899203	0.016205806	*LINC00921;ZNF263*
chr9	80850389	80850856	*	3	0.009767246	-0.020282688	-0.008037109	*CEP78*
chr3	154796701	154798079	*	18	0.00977705	-0.019985215	-0.006453693	*MME*
chr16	55866757	55867072	*	4	0.009779257	-0.084082934	-0.050156174	*CES1*
chr11	32355324	32355899	*	4	0.009787958	0.025482871	0.017415288	*NA*
chr2	97533635	97534450	*	4	0.009793644	-0.021770389	0.000734114	*SEMA4C*
chr12	110687347	110687977	*	3	0.009798341	0.027426584	0.007619899	*NA*
chr12	49373240	49374710	*	7	0.009799942	-0.00885292	-0.001245087	*WNT1*
chr3	194905391	194906452	*	5	0.009800996	0.01470255	-0.002956495	*XXYLT1*
chr11	71752705	71753240	*	4	0.009811507	-0.026849899	-0.012069453	*NUMA1*
chr13	111805314	111805930	*	7	0.009838622	0.024259028	0.006373182	*ARHGEF7*

chr1	210501532	210502792	*	13	0.009839793	0.020723879	-0.003070556	*HHAT*
chr10	102469807	102470340	*	4	0.009842118	-0.011053485	-0.005413929	*NA*
chr6	169422647	169423797	*	5	0.009842513	0.025349558	0.016286033	*NA*
chr6	71123675	71124075	*	6	0.009842673	-0.024705514	-0.011999235	*FAM135A*
chr17	79010968	79012396	*	5	0.009848992	0.047083443	0.006413171	*BAIAP2;BAIAP2-DT*
chr4	108745001	108745771	*	10	0.009865197	0.017454113	0.005149391	*SGMS2*
chr6	32115979	32116317	*	9	0.009880484	-0.02247884	-0.006656741	*PPT2;PRRT1*
chr8	135725874	135726595	*	4	0.00988931	0.025503128	0.010609275	*ZFAT*
chr16	88716846	88717989	*	16	0.009890407	-0.025711154	-0.004953177	*CYBA*
chr22	35795274	35795394	*	5	0.009890407	0.020568046	0.013101943	*MCM5*
chr11	33758121	33759043	*	8	0.009899078	-0.019104865	-0.006745771	*CD59*
chr15	80215376	80216491	*	15	0.009905544	-0.017779195	-0.003192833	*ST20;ST20-AS1;ST20-MTHFS*
chr17	53341098	53343075	*	12	0.009905544	-0.02657738	-0.005600106	*HLF*
chr17	1421079	1421517	*	3	0.009905544	0.023924298	0.009054789	*INPP5K;PITPNA;PITPNA-AS1*
chr11	1967857	1968643	*	9	0.009932584	-0.041889197	-0.004562304	*MRPL23*
chr11	122525644	122526355	*	7	0.00995537	-0.044359081	-0.003935058	*UBASH3B*
chr1	112259992	112260326	*	3	0.009957212	0.025159446	0.008430497	*NA*
chr16	71929343	71930048	*	8	0.009962353	-0.017156925	-0.001615384	*IST1*
chr7	87257356	87258443	*	11	0.009972563	0.028861141	0.001954974	*ABCB1;RUNDC3B*
chr12	46386648	46387141	*	3	0.009973023	0.04554405	0.020697784	*SCAF11*
chr2	228497892	228498185	*	3	0.009977966	0.038567607	0.008373383	*C2orf83*
chr20	46286436	46286980	*	3	0.009978341	0.016992349	0.003197604	*SULF2*
chr19	3942158	3942969	*	5	0.010000373	0.021233898	0.008590878	*NMRK2*
chr16	27761089	27761488	*	3	0.010000373	0.014913666	-0.000805409	*KATNIP*
chr16	89022688	89023633	*	5	0.010015015	-0.027084973	-0.014455237	*CBFA2T3*
chr7	128488744	128488920	*	3	0.010015015	0.023402773	0.015900822	*FLNC*
chr22	20863268	20864312	*	3	0.010016584	0.019692031	0.013214421	*MED15*
chr6	109761938	109762635	*	8	0.01002103	0.005482639	-0.001008559	*PPIL6;SMPD2*
chr16	1754752	1755402	*	3	0.010027961	-0.023615904	0.001340014	*MAPK8IP3*
chr7	1552941	1553444	*	4	0.010033513	-0.012292919	-0.007938035	*NA*
chr6	7468364	7469052	*	6	0.010040698	-0.032701574	-0.02400501	*NA*
chr1	24126017	24126641	*	6	0.010047109	0.016542909	0.006291743	*GALE*
chr15	96884100	96884532	*	4	0.010047109	-0.023471275	-0.005663297	*NA*
chr8	10382921	10383763	*	6	0.010092163	-0.02258179	-0.00385072	*PRSS55*
chr1	113525753	113526539	*	3	0.010110846	-0.023940112	-0.009554291	*NA*
chr9	125109046	125110034	*	4	0.010113454	0.024055836	0.011108967	*NA*
chr17	5095165	5095970	*	10	0.010127515	-0.016870508	-0.005256927	*LOC100130950;ZNF594*
chr1	154155675	154156895	*	9	0.010144793	-0.014599172	-0.000922639	*TPM3*
chr7	116502333	116503366	*	12	0.010163726	-0.01973141	-0.004491201	*CAPZA2*
chr10	118924342	118924759	*	3	0.010172923	-0.018317948	-0.005796211	*NA*
chr16	2836681	2836845	*	4	0.010174824	0.028373638	0.016592357	*PRSS33*
chr1	38100732	38101266	*	7	0.010242123	0.034168511	0.007650643	*RSPO1*
chr3	4863817	4864036	*	3	0.010243256	-0.024810387	-0.014090694	*ITPR1*
chr17	46233714	46234525	*	6	0.010243282	0.021630013	-0.006179598	*MIR1203;SKAP1*
chr21	40140783	40141756	*	4	0.010248488	0.026799248	0.007429052	*LINC00114*
chr14	100631741	100631897	*	3	0.010250485	0.049129421	0.029166432	*NA*
chr17	11143830	11144509	*	4	0.010254684	-0.036437526	-0.012628314	*SHISA6*
chr17	45726416	45727226	*	12	0.010256854	0.022532107	0.001750099	*KPNB1*
chr9	80647290	80647629	*	4	0.01025715	-0.00644998	-0.002583641	*GNAQ*
chr14	89506875	89507624	*	4	0.010264588	-0.030745772	-0.013919182	*NA*
chr1	40781617	40782458	*	5	0.010270083	0.036146155	0.004368502	*COL9A2*
chr13	20531619	20532174	*	4	0.01027444	-0.012672491	-0.000155063	*ZMYM2*
chr16	66994879	66995461	*	7	0.010275479	0.023200419	0.011773361	*CES3*
chr22	19512712	19513176	*	9	0.010288533	0.032935954	0.017097268	*CLDN5*
chr17	79495006	79495953	*	9	0.010296635	-0.030710926	-0.011635304	*FSCN2*
chr6	43736827	43737749	*	5	0.010300188	0.015222496	0.004749667	*VEGFA*
chr2	227660067	227660451	*	3	0.01030881	0.013934681	0.010123743	*IRS1*
chr2	32502226	32503219	*	14	0.010313859	-0.020655228	-0.001871903	*YIPF4*
chr14	104639580	104640667	*	6	0.010315158	0.015883666	0.007061933	*KIF26A*
chr14	57278084	57278729	*	4	0.010324782	0.040906752	0.008641182	*OTX2;OTX2-AS1*
chr9	34956991	34958141	*	9	0.010328125	-0.022185314	0.002508205	*PHF24*
chr8	27449470	27449755	*	4	0.010328273	0.020703084	0.005402461	*NA*
chr7	73256360	73256761	*	4	0.010328273	0.02432307	0.010644545	*METTL27*
chr22	24950917	24951987	*	20	0.010351434	0.012108621	0.000315326	*GUCD1;SNRPD3*
chr8	26240309	26241120	*	12	0.010359802	0.016557475	0.00062874	*BNIP3L*
chr15	27111993	27112902	*	9	0.010368879	-0.050157207	-0.011767097	*GABRA5;GABRB3*

chr20	30073209	30073576	*	6	0.010368879	0.038780449	0.023529187	*LINC00028*
chr20	50179398	50180224	*	6	0.010375543	-0.012301201	0.000651203	*NFATC2*
chr11	64727184	64728252	*	4	0.010380376	-0.027062389	-0.010740748	*MAJIN*
chr7	2151462	2152625	*	5	0.010384715	-0.025359876	-0.00969318	*MAD1L1*
chr8	144610080	144611701	*	6	0.010388595	0.020847972	0.00322599	*ZC3H3*
chr15	90030316	90030679	*	3	0.010389689	-0.022914394	-0.02005859	*RHCG*
chr6	30688035	30689568	*	27	0.010395737	0.028024475	0.001632176	*MDC1;TUBB*
chr17	3499165	3499671	*	3	0.01039957	0.017543647	0.015175446	*TRPV1*
chr1	156130293	156131379	*	4	0.010406944	-0.01726433	-0.00803745	*SEMA4A*
chr16	10133433	10133501	*	3	0.010408789	-0.030818142	-0.019122228	*GRIN2A*
chr5	59559062	59559236	*	4	0.010413631	-0.016226405	-0.010122121	*PDE4D*
chr14	78869751	78870232	*	6	0.010419315	-0.042523426	-0.020940675	*NRXN3*
chr3	31750433	31751191	*	5	0.010419315	-0.022371239	-0.008477215	*OSBPL10;OSBPL10-AS1*
chr7	127807832	127808848	*	5	0.01044386	-0.026243569	-0.001739446	*NA*
chr11	13983273	13984596	*	15	0.010451114	0.042452684	0.014452902	*SPON1*
chr8	141108607	141109731	*	5	0.010453781	-0.030803453	0.002453918	*TRAPPC9*
chr12	119616880	119617656	*	4	0.010460594	-0.031397479	-0.007719997	*HSPB8*
chr20	30071726	30072118	*	3	0.010473707	0.020439253	0.01894986	*LINC00028;REM1*
chr12	81471194	81472177	*	14	0.010481587	-0.012566137	-0.005994877	*ACSS3*
chr6	96025679	96026116	*	4	0.010481587	0.01741902	0.00649177	*MANEA*
chr16	1507634	1509206	*	7	0.010492065	-0.05270492	-0.019635673	*CLCN7*
chr8	37962497	37963526	*	19	0.010506365	-0.035344492	-0.00278734	*ASH2L*
chr3	138433416	138434145	*	4	0.010517005	0.020508311	0.00498848	*PIK3CB*
chr9	116356058	116356457	*	3	0.01052186	-0.018398861	-0.004045748	*RGS3*
chr17	4842919	4844613	*	17	0.010522114	-0.01899848	-0.000924484	*RNF167;SLC25A11*
chr17	77019777	77020549	*	12	0.010542759	0.009069816	0.001078275	*C1QTNF1;C1QTNF1-AS1*
chr2	25920087	25920923	*	4	0.010545374	0.050117592	0.024386477	*NA*
chr3	45984585	45985168	*	7	0.010555495	-0.021372242	-0.008767914	*CXCR6;FYCO1*
chr8	134114834	134115661	*	9	0.01058962	0.018844352	0.000396873	*SLA;TG*
chr17	19355650	19355814	*	4	0.010598661	0.027477127	0.013206946	*NA*
chr17	73285181	73285885	*	11	0.010599062	0.005156628	-0.000465742	*SLC25A19*
chr2	67623673	67624930	*	11	0.010605644	0.018940872	-0.001545831	*ETAA1*
chr7	27190235	27190849	*	8	0.01060902	-0.016838812	-0.004184225	*HOXA-AS3;HOXA6*
chr12	96883202	96883863	*	8	0.010609052	0.01325075	0.002732424	*NA*
chr1	8483475	8485666	*	16	0.010611075	0.032537856	0.006246492	*RERE*
chr19	38876250	38877739	*	6	0.010611949	0.031170451	0.018886389	*GGN;SPRED3*
chr10	103910870	103911881	*	7	0.010612159	0.0212165	0.008664071	*NOLC1*
chr13	77601257	77601924	*	9	0.010629576	0.00825837	0.001936225	*FBXL3*
chr10	134549665	134550058	*	5	0.010666201	0.018981635	0.010762297	*INPP5A*
chr1	41487940	41488323	*	4	0.010690496	0.022364383	0.014170307	*SLFNL1;SLFNL1-AS1*
chr4	141419169	141419915	*	10	0.010727584	-0.015676996	-0.002938713	*MGAT4D*
chr6	28911464	28912166	*	10	0.010731326	0.030160814	0.007992293	*NA*
chr5	56205718	56206494	*	7	0.010735604	0.014602362	0.000110382	*SETD9*
chr9	136819239	136819694	*	3	0.010735977	0.023074864	0.014324301	*VAV2*
chr17	79425877	79426432	*	5	0.010739442	-0.023650687	-0.008286704	*BAHCC1*
chr3	138654924	138655775	*	5	0.010752119	0.022957493	0.000813906	*NA*
chr3	194030520	194030977	*	6	0.010757647	-0.014788783	-0.005563056	*LINC00887*
chr17	79792334	79793439	*	12	0.010761187	-0.022087671	-0.006119198	*MCRIP1;PPP1R27*
chr7	117067416	117068162	*	10	0.010771558	0.029587975	0.012912119	*ASZ1*
chr14	79745154	79746056	*	11	0.010774044	-0.014012424	-0.005685087	*NRXN3*
chr7	150674012	150675308	*	6	0.010774044	-0.04152913	-0.01528923	*KCNH2*
chr7	99716514	99717359	*	14	0.01080467	-0.017692407	-0.005359481	*CNPY4;TAF6*
chr6	78359965	78360731	*	4	0.01080467	0.020668819	0.004353735	*NA*
chr12	71314144	71314315	*	3	0.010811388	-0.022477586	-0.01317684	*PTPRR*
chr11	27721222	27722889	*	21	0.010832504	-0.021354574	-0.002900767	*BDNF*
chr4	111558988	111559355	*	5	0.01085413	0.021451686	-0.001293049	*PITX2*
chr15	93614970	93616424	*	9	0.010857218	0.042611699	0.009119029	*RGMA*
chr7	150689873	150689889	*	3	0.010857218	-0.032563257	-0.012183816	*NOS3*
chr9	112887376	112889386	*	10	0.010869044	-0.026583753	-0.008048248	*PALM2AKAP2*
chr16	23480974	23481476	*	3	0.010880636	0.015340479	0.002182176	*GGA2*
chr13	24144257	24144985	*	7	0.010892622	-0.031423521	-0.01399479	*TNFRSF19*
chr8	56851846	56852290	*	5	0.010898579	-0.009471273	-0.004192588	*LYN*
chr17	70981390	70982380	*	3	0.010904203	0.017863122	-0.001241598	*SLC39A11*
chr17	6899085	6899888	*	14	0.010905646	0.047519809	0.018834252	*ALOX12;ALOX12-AS1*
chr14	95026888	95028105	*	9	0.010923341	0.022265982	0.012029263	*SERPINA4*
chr21	26980353	26980614	*	3	0.010932186	-0.007127004	-0.001358026	*MRPL39*

chr3	194826197	194826983	*	6	0.010936125	-0.02238672	-0.016048537	*XXYLT1*
chr15	89764836	89765425	*	7	0.010938842	0.021050848	0.012322577	*RLBP1*
chr20	50807767	50808668	*	9	0.010947912	0.00704211	-9.01E-05	*ZFP64*
chr13	103514655	103515035	*	5	0.010954864	0.013589035	0.004847795	*BIVM-ERCC5;ERCC5*
chr6	125420338	125421084	*	5	0.01097881	0.013949822	-0.001181572	*NA*
chr20	17595355	17595472	*	3	0.010978827	0.058269466	0.037403949	*RRBP1*
chr7	120628572	120629404	*	9	0.010992957	-0.010475535	-0.005086442	*CPED1*
chr4	3043199	3043752	*	3	0.010998066	-0.033366633	-0.032402212	*NA*
chr15	83349171	83349864	*	5	0.011006903	-0.008438343	-0.004824157	*AP3B2;CPEB1-AS1*
chr15	23893742	23894273	*	5	0.011058029	-0.033089589	-0.009680256	*MAGEL2*
chr17	66031448	66032328	*	12	0.011062149	0.017899723	0.000289741	*KPNA2*
chr20	34242979	34243249	*	3	0.01106516	-0.022257561	-0.014296174	*CPNE1;RBM12*
chr14	24024869	24025797	*	15	0.011075892	0.019369434	0.00171861	*THTPA;ZFHX2*
chr11	104839074	104839858	*	4	0.011094196	0.024111392	0.000496632	*CASP4*
chr8	38323742	38324522	*	6	0.011100714	0.012209575	0.00302919	*FGFR1*
chr2	133426129	133426653	*	4	0.011100714	-0.010801614	-0.00618969	*LYPD1*
chr3	196730265	196731150	*	8	0.011107667	0.016373988	-0.001777254	*MELTF;MELTF-AS1*
chr11	67895682	67896186	*	4	0.011107772	-0.011389404	-0.001520038	*NA*
chr2	240876536	240876920	*	3	0.011112837	-0.030065987	-0.015410511	*NA*
chr7	872480	872797	*	4	0.011141131	-0.024666082	-0.007870039	*SUN1*
chr16	87755225	87755754	*	4	0.011141131	0.022367962	0.008952623	*KLHDC4*
chr4	95372810	95373403	*	8	0.011143987	-0.026727062	-0.001424606	*PDLIM5*
chr11	63606263	63607025	*	11	0.011152944	-0.018761619	-0.00546825	*MARK2*
chr13	99194582	99195339	*	3	0.011162585	-0.020179146	-0.006487859	*STK24*
chr1	231114438	231115458	*	14	0.01116506	0.013903389	0.000479013	*ARV1;TTC13*
chr2	238535538	238536315	*	10	0.011177723	0.032848864	-0.001266456	*LRRFIP1*
chr4	675137	675936	*	4	0.011179706	-0.051385788	-0.032192005	*MYL5;SLC49A3*
chr2	177133722	177134737	*	10	0.011180103	0.013376411	0.000998549	*MTX2*
chr11	31821064	31821764	*	7	0.01118123	0.023113956	0.002887248	*PAX6*
chr5	43313384	43313815	*	8	0.011187139	-0.013050687	-0.001235462	*HMGCS1*
chr12	65515350	65516002	*	6	0.011187139	0.037463427	0.014134308	*WIF1*
chr2	228160709	228160922	*	3	0.011216956	-0.019133187	-0.006379696	*COL4A3;MFF-DT*
chr18	12377893	12378044	*	4	0.011219323	-0.01401716	-0.006214084	*AFG3L2*
chr15	25068564	25069376	*	8	0.011227869	-0.028729723	-0.010361519	*SNRPN*
chr7	157129064	157130065	*	12	0.011238515	-0.008256637	-0.002641732	*DNAJB6*
chr4	1318845	1319171	*	5	0.011240869	0.013604577	0.000401271	*MAEA*
chr3	160117013	160117717	*	9	0.011254141	-0.018828729	-0.001080326	*IFT80;MIR15B;MIR16-2;SMC4*
chr6	33176234	33176673	*	18	0.011256884	-0.021466411	-0.005426797	*RING1*
chr19	34263644	34264111	*	5	0.011295415	0.027647311	0.017939695	*CHST8*
chr8	142517494	142518155	*	4	0.011295497	-0.03012326	-0.007009289	*MROH5*
chr16	31438767	31439681	*	6	0.011297946	0.034239007	0.027462185	*COX6A2*
chr1	19407878	19408371	*	3	0.011302787	-0.017580044	-0.012791442	*UBR4*
chr18	40857256	40857693	*	5	0.011308481	-0.016864312	-0.00285683	*SYT4*
chr14	99786193	99786226	*	3	0.011313368	0.027314465	0.013971074	*NA*
chr3	27765484	27766382	*	8	0.011316162	-0.026974307	-0.003484203	*EOMES*
chr6	291687	292823	*	9	0.011323863	0.053011157	0.042649384	*DUSP22*
chr16	67038709	67039332	*	5	0.011323863	0.016241803	0.006352084	*CES4A*
chr19	44405433	44406241	*	5	0.011330926	0.031695904	0.008323755	*LOC100505715*
chr14	51410260	51410689	*	3	0.01134423	-0.015617239	-0.008496137	*PYGL*
chr1	214557246	214558155	*	9	0.01135319	0.02643835	0.005343294	*PTPN14*
chr9	100459097	100460584	*	9	0.011358764	0.024648288	0.001720554	*XPA*
chr19	18717641	18718202	*	7	0.01137948	-0.019888128	-0.003702794	*CRLF1*
chr1	214160412	214161251	*	4	0.011382635	-0.024328845	-0.00904329	*PROX1*
chr22	29137385	29138453	*	15	0.01139426	-0.013012115	-0.001047466	*CHEK2;HSCB*
chr4	8159729	8161093	*	13	0.011395585	0.016749397	-0.001182964	*ABLIM2*
chr7	4901337	4901798	*	8	0.011400234	-0.038990943	-0.026568381	*PAPOLB;RADIL*
chr1	229478916	229479495	*	7	0.011401209	-0.042285229	-0.009304149	*CCSAP*
chr7	128046088	128046797	*	3	0.011405966	0.012081629	0.004454049	*IMPDH1*
chr18	29170465	29170604	*	3	0.011407163	-0.018191918	-0.001299699	*TTR*
chr4	174429263	174430141	*	9	0.011418548	-0.014153871	-0.004991531	*NA*
chr17	26554221	26554610	*	3	0.011418548	0.024029434	0.015052986	*PYY2*
chr16	53536813	53537952	*	12	0.011419333	0.014660305	0.001026961	*AKTIP*
chr12	52344971	52345646	*	6	0.011426355	-0.050957234	-0.008584288	*ACVR1B*
chr15	50646438	50647583	*	16	0.011427412	-0.015602141	-0.001099706	*GABPB1;GABPB1-AS1;GABPB1-IT1;MIR4712*
chr8	49647884	49648519	*	8	0.011439509	-0.012510554	-0.004307976	*EFCAB1*
chr14	72925867	72926359	*	4	0.011440346	-0.014463479	0.001808033	*RGS6*

chr16	23724428	23724901	*	8	0.011446914	-0.026663515	0.00045018	*ERN2*
chr6	100056686	100057160	*	5	0.011450844	-0.037817921	-0.012177744	*PRDM13*
chr21	47421947	47422256	*	3	0.011450844	0.019510679	0.015913495	*COL6A1*
chr15	70766226	70767649	*	6	0.011457512	-0.022436751	-0.011958174	*NA*
chr11	64814948	64816327	*	10	0.011464485	0.017676751	0.002613459	*NAALADL1*
chr14	21994785	21995727	*	5	0.011467908	-0.015005412	-0.005889186	*SALL2*
chr17	81018426	81018559	*	3	0.011467908	-0.022951972	-0.006959761	*NA*
chr16	15766245	15767006	*	5	0.011470253	-0.014956573	-0.010031779	*NDE1*
chr6	163834452	163835582	*	7	0.011473098	-0.026194887	-0.009291725	*CAHM;QKI*
chr5	178204356	178204766	*	3	0.01147408	-0.017904456	-0.013912886	*AACSP1*
chr12	107077503	107078653	*	7	0.011489294	-0.013303334	-0.00232621	*LOC100287944;LOC100505978;RFX4*
chr15	94406157	94406247	*	3	0.011491653	-0.011163471	-0.009174716	*NA*
chr16	25702150	25702614	*	3	0.011500083	-0.019499001	-0.008537532	*HS3ST4*
chr3	123519116	123520027	*	4	0.011500779	0.02336721	0.018532657	*MYLK*
chr15	85143675	85144807	*	14	0.01151254	0.013506346	-0.001557501	*ZSCAN2*
chr17	6734940	6735539	*	8	0.011515877	-0.012658181	-0.006020896	*TEKT1*
chr17	62492736	62493599	*	6	0.011520181	-0.005437553	-0.001680664	*POLG2*
chr6	28828946	28830041	*	22	0.011520479	0.019908018	0.008289532	*LINC01623*
chr17	38821155	38821487	*	8	0.011529114	-0.022033835	-0.014357277	*KRT222*
chr16	18994601	18995637	*	13	0.011554716	0.018511741	0.00066586	*TMC7*
chr17	3289363	3290164	*	8	0.011572204	-0.049048685	-0.004363973	*NA*
chr7	23529999	23530810	*	4	0.011635824	-0.027699561	-0.02134813	*RPS2P32*
chr2	44588702	44589694	*	17	0.011656216	0.011602691	-0.000852543	*CAMKMT;PREPL*
chr16	29973734	29974702	*	8	0.011656216	0.030631921	0.008256386	*BOLA2;TMEM219*
chr20	45169840	45170494	*	4	0.011660164	-0.027714503	-0.012829363	*OCSTAMP*
chr1	24525983	24526095	*	3	0.011666512	-0.027223496	-0.020268492	*LINC02800*
chr17	38375119	38375600	*	10	0.011684849	0.011651829	-0.001895748	*WIPF2*
chr3	134031551	134031948	*	4	0.011701653	0.035569652	0.022182878	*NA*
chr8	93107298	93108262	*	10	0.011720345	-0.030945832	-0.005172472	*RUNX1T1*
chr6	31105337	31105744	*	6	0.011722911	-0.02261888	-0.008881332	*PSORS1C1;PSORS1C2*
chr20	2635105	2635586	*	4	0.011725669	0.026391434	0.013699231	*NOP56;SNORA51*
chr22	27695418	27696203	*	3	0.011725669	-0.016026949	-0.006793518	*NA*
chr13	111290667	111291316	*	6	0.011746011	0.025417657	0.010086047	*NAXD*
chr2	10442817	10443758	*	13	0.011762128	-0.030251085	0.00040992	*HPCAL1*
chr1	89664260	89664619	*	7	0.011763952	-0.052592227	-0.01084292	*GBP4*
chr20	42193485	42194053	*	8	0.011767936	0.019988641	0.005167279	*SGK2*
chr15	91475537	91475925	*	10	0.011768687	0.013808111	4.75E-05	*HDDC3;UNC45A*
chr16	66585210	66585924	*	8	0.011769347	-0.021353399	-0.00233379	*CKLF;CKLF-CMTM1;TK2*
chr13	78492568	78493365	*	20	0.011780871	0.019698081	-7.07E-06	*EDNRB;OBI1-AS1*
chr5	79287118	79288180	*	8	0.01178255	0.033354315	0.007014129	*MTX3*
chr2	126155953	126156962	*	3	0.01178442	0.032417615	0.007151066	*NA*
chr2	242665153	242666066	*	8	0.011787751	0.014240096	0.000576664	*ING5*
chr15	85201039	85202042	*	10	0.011793225	0.041251742	0.003693989	*NMB;WDR73*
chr9	136506306	136506883	*	3	0.011799156	0.019937347	0.01491606	*DBH*
chr17	79077169	79077745	*	5	0.011809962	0.015145647	0.00681111	*BAIAP2*
chr1	151966448	151967023	*	10	0.011812314	-0.039325626	-0.001980489	*S100A10*
chr6	134373485	134374706	*	7	0.011814847	-0.015908305	-0.006577453	*SLC2A12*
chr7	4210949	4211395	*	5	0.011816628	0.021016212	0.004878618	*SDK1*
chr1	66999304	66999933	*	9	0.011823601	-0.018850626	-0.010808915	*SGIP1*
chr12	122711720	122712381	*	10	0.011831005	-0.02223471	-0.008898963	*DIABLO;VPS33A*
chr17	43246622	43246982	*	3	0.011851855	0.026545418	0.023281272	*HEXIM2*
chr15	33359788	33360329	*	6	0.011896595	-0.023168626	-0.013475736	*FMN1*
chr6	36915498	36916123	*	5	0.011896595	-0.028571903	-0.014001482	*PI16*
chr12	121570559	121571068	*	7	0.011901621	-0.015042138	-0.006526087	*P2RX7*
chr2	237415899	237416899	*	10	0.011903406	0.024436137	-0.00097971	*IQCA1*
chr7	2557319	2558200	*	9	0.011903406	0.02728863	0.011470015	*LFNG*
chr6	156952100	156952588	*	5	0.011903406	0.019606786	0.007506946	*NA*
chr2	230451648	230452045	*	7	0.011906152	-0.03444528	-0.014926764	*DNER*
chr15	49255672	49256258	*	8	0.011915606	0.025118277	-0.001235791	*SHC4*
chr13	22254743	22255230	*	4	0.011935872	0.030695527	0.001963947	*FGF9*
chr14	23770452	23771256	*	5	0.011940868	0.006893303	0.003637092	*BCL2L2;BCL2L2-PABPN1;HOMEZ;PPP1R3E*
chr14	56777076	56777875	*	7	0.011945574	-0.041749424	-0.020107193	*NA*
chr7	134575306	134576239	*	8	0.011949975	0.026948998	0.004103057	*CALD1*
chr1	182642748	182643312	*	5	0.011949975	-0.030778354	-0.005318622	*RGS8*
chr10	75490061	75490334	*	5	0.011960711	0.011447228	0.003632227	*AGAP5;BMS1P4;GLUD1P3*
chr4	57844912	57845825	*	9	0.011966815	0.021116579	0.002130313	*NOA1;POLR2B*

chr8	76316585	76317040	*	3	0.011983626	-0.009770782	-0.009081182	*HNF4G*
chr1	114471173	114471974	*	12	0.011991085	-0.008242813	-0.001231839	*HIPK1*
chr6	29599319	29600462	*	13	0.012006242	-0.017458335	-0.000985434	*GABBR1*
chr17	48350468	48350867	*	7	0.012020925	-0.011601681	-0.001159094	*TMEM92*
chr1	224517614	224518531	*	16	0.012043673	0.013351703	0.000210363	*NVL*
chr2	173420046	173421107	*	12	0.012047184	-0.022143796	-0.001684212	*PDK1*
chr9	124982087	124982834	*	4	0.012047184	0.035047404	0.020237839	*LHX6*
chr9	138130682	138130794	*	3	0.012047184	0.021185263	0.004681174	*NA*
chr7	27252541	27253521	*	5	0.012057475	-0.031165767	0.002123036	*NA*
chr12	56390073	56391059	*	10	0.012075887	-0.017819563	-0.008548335	*RAB5B;SUOX*
chr1	165204410	165205201	*	11	0.012078449	-0.013282266	-0.004559828	*LMX1A*
chr6	167809876	167810395	*	4	0.012079668	0.029693214	0.016088148	*NA*
chr1	12538341	12538678	*	6	0.012080278	-0.024990966	-0.011722182	*VPS13D*
chr12	72057638	72058485	*	16	0.012103397	-0.012794992	-0.00074059	*THAP2;ZFC3H1*
chr3	107940784	107941759	*	9	0.012113977	0.027222009	0.003160592	*IFT57*
chr4	105411756	105412832	*	11	0.012127262	0.021336872	-0.00022752	*CXXC4*
chr6	35995251	35996074	*	16	0.012130093	-0.011726404	-0.000810309	*MAPK14;SLC26A8*
chr22	41032662	41033637	*	14	0.012131777	-0.015292761	-0.003609954	*MRTFA*
chr1	1895061	1895206	*	3	0.012139387	0.0187576	0.010760868	*CFAP74*
chr3	147130477	147131860	*	8	0.012148004	0.016600024	0.000600341	*ZIC1*
chr17	61818420	61819127	*	4	0.012156428	0.010361447	0.002571818	*STRADA*
chr5	119799531	119800742	*	10	0.012160969	-0.016326929	-0.006933809	*PRR16*
chr14	106743465	106744098	*	4	0.012160969	0.025874884	0.02161079	*LINC00226*
chr5	139553863	139555009	*	16	0.012166314	-0.017652255	0.000277664	*CYSTM1*
chr7	157980803	157981888	*	6	0.012166314	0.036217832	0.019052433	*PTPRN2*
chr6	152011399	152012474	*	5	0.012166314	-0.02438072	-0.017866364	*ESR1*
chr16	49564354	49564462	*	3	0.012166771	-0.030716981	-0.022094713	*ZNF423*
chr22	50097381	50098317	*	4	0.012178638	0.023497591	0.018413709	*NA*
chr19	44115850	44116266	*	3	0.012179383	0.025801709	0.007024186	*SRRM5;ZNF428;ZNF576*
chr10	134914910	134915859	*	10	0.012186544	-0.023566116	-0.011506584	*ADGRA1*
chr15	82335947	82336571	*	5	0.012189672	0.029672624	0.019398756	*MEX3B*
chr9	139258938	139260203	*	4	0.01219849	0.042515577	0.014361694	*CARD9;DNLZ*
chr1	207226824	207227076	*	3	0.01219849	0.029768364	0.007387089	*PFKFB2;YOD1*
chr10	88123169	88123687	*	3	0.012207171	-0.011183068	-0.007744124	*GRID1*
chr6	30851417	30852354	*	12	0.012208509	-0.017216566	-0.00236516	*DDR1*
chr20	62126068	62126827	*	3	0.01223498	0.030571869	0.025955202	*EEF1A2*
chr2	75144945	75145387	*	3	0.012244664	0.022671454	0.015497581	*NA*
chr6	159359940	159360209	*	3	0.012251309	-0.042359426	-0.029453089	*NA*
chr11	2443869	2445442	*	18	0.012262561	0.020857936	0.005621567	*TRPM5*
chr8	95003548	95003953	*	4	0.012271681	-0.005994868	-0.003215936	*NA*
chr3	49942076	49942468	*	4	0.012286149	0.01997455	0.005751211	*MST1R*
chr5	168006544	168007339	*	9	0.012291245	-0.019860045	0.000466914	*PANK3*
chr17	60972789	60973151	*	3	0.012295399	-0.026153812	-0.013840628	*NA*
chr5	1037979	1038605	*	5	0.012299347	0.019726814	0.00354698	*NKD2*
chr2	129076914	129077561	*	4	0.012310448	0.042463874	0.025989877	*HS6ST1*
chr1	61869210	61869595	*	4	0.012311688	-0.019260039	-0.011456381	*NFIA*
chr13	110380326	110380729	*	6	0.012330928	0.031664114	0.012993145	*NA*
chr15	81588897	81589273	*	7	0.012339716	-0.026535019	-0.0063931	*IL16*
chr3	183898691	183899151	*	7	0.012356818	0.025888844	-0.000906371	*ABCF3;AP2M1*
chr6	146056770	146057241	*	7	0.012373363	-0.011895971	-0.006124629	*EPM2A;FBXO30-DT*
chr6	15248611	15249351	*	5	0.012373363	-0.036076738	-0.019716778	*JARID2*
chr12	132324688	132325615	*	6	0.01237848	0.024760398	0.009920424	*MMP17*
chr11	94963145	94963674	*	6	0.012380717	-0.00488393	-0.002872801	*SESN3*
chr11	66277720	66278839	*	10	0.01238483	-0.030493194	-0.015061676	*BBS1*
chr13	44543031	44543954	*	4	0.012398699	0.016449141	0.009612676	*NA*
chr21	43161312	43162018	*	4	0.012409482	0.030621938	0.015895519	*RIPK4*
chr3	26664115	26665093	*	8	0.012419221	-0.015876485	-0.002996913	*LRRC3B*
chr19	8115260	8116316	*	5	0.012419221	-0.027849124	-0.0101734	*CCL25*
chr3	50336673	50337494	*	17	0.012423855	-0.029703823	-0.003541337	*HYAL1;HYAL3;NAA80*
chr7	27778680	27779713	*	15	0.012439483	0.014813426	-0.000787211	*TAX1BP1*
chr16	2867434	2868001	*	6	0.012439483	0.05479533	0.024454479	*PRSS21*
chr15	96596177	96596924	*	6	0.01246199	-0.018205991	-0.00936876	*NA*
chr7	41137006	41137309	*	3	0.012475873	0.030992203	0.006612286	*NA*
chr9	75567483	75568073	*	4	0.012483695	-0.012451743	-0.002928317	*ALDH1A1*
chr1	41119283	41119988	*	4	0.01252571	0.029738753	0.02081654	*RIMS3*
chr12	7282186	7283214	*	18	0.012557288	-0.023146486	-0.006401815	*CLSTN3;RBP5*

chr6	144471985	144473325	*	4	0.012561992	0.018459886	0.000940459	*STX11*
chr1	243753445	243754323	*	3	0.012567486	-0.023003883	0.000303682	*AKT3*
chr11	45354316	45355222	*	4	0.012575817	-0.026249965	-0.012625461	*NA*
chr11	133777831	133777981	*	3	0.012575817	0.011716064	0.004116078	*NA*
chr6	31110639	31111270	*	5	0.012576947	0.035285008	0.008520036	*CCHCR1;PSORS1C2*
chr2	27193211	27193588	*	6	0.012581326	-0.009128803	-0.004268227	*MAPRE3*
chr3	108019540	108020451	*	3	0.012581326	-0.022655693	-0.011770015	*HHLA2*
chr16	70286430	70286779	*	3	0.012613498	0.022400812	0.012181103	*AARS1;EXOSC6*
chr1	156548584	156549533	*	6	0.012615812	0.020313816	0.009277263	*TTC24*
chr4	126234763	126236816	*	8	0.012622464	-0.020933178	-0.006344997	*FAT4*
chr11	108799210	108799922	*	3	0.012638113	0.016240527	0.010660437	*DDX10*
chr19	39402823	39403373	*	7	0.01263985	-0.033591015	-0.016894621	*CCER2*
chr7	124569440	124570181	*	9	0.012668979	-0.018341181	-0.002464898	*POT1*
chr7	148903126	148903301	*	3	0.012672457	0.015775363	0.000253837	*ZNF282*
chr8	21899862	21900698	*	8	0.01267687	-0.028157458	-0.009544894	*FGF17*
chr15	89920887	89922071	*	13	0.012681329	0.022944784	0.009344564	*MIR9-3HG*
chr12	6553092	6553937	*	6	0.012683571	-0.022251905	-0.007545173	*CD27;CD27-AS1*
chr3	142720700	142720940	*	3	0.012683571	0.009377949	0.001052342	*LOC100289361;U2SURP*
chr2	128173222	128173750	*	5	0.012688567	-0.015569154	-0.007618809	*PROC*
chr16	81875718	81876162	*	3	0.012688914	0.018739799	0.016524429	*PLCG2*
chr1	212803761	212804203	*	3	0.012703388	-0.01949499	-0.00783829	*NA*
chr1	205600246	205600634	*	4	0.012711816	-0.025161039	-0.009971262	*ELK4;SLC45A3*
chr4	7787960	7788342	*	4	0.01272403	-0.033966878	-0.013014889	*AFAP1*
chr19	50553022	50553708	*	6	0.012750147	0.030611454	0.009899624	*LOC400710*
chr1	192917648	192918151	*	3	0.012765484	-0.025480723	0.00038673	*NA*
chr21	34913894	34915275	*	19	0.012785272	0.012480802	-0.000912564	*GART;SON*
chr3	137906196	137906843	*	7	0.012789159	0.005976055	2.08E-05	*ARMC8*
chr21	46237743	46238210	*	3	0.012796654	-0.02249846	-0.013815627	*SUMO3*
chr9	140388081	140388604	*	3	0.012804398	0.015755642	0.013310997	*PNPLA7*
chr3	160822911	160823781	*	14	0.012809661	-0.015205527	-0.004221624	*B3GALNT1*
chr3	196351986	196352455	*	4	0.012813986	-0.021296594	-0.01144138	*NA*
chr2	74754237	74755125	*	3	0.012819228	0.0139278	0.004213339	*AUP1;DQX1;HTRA2*
chr10	134332277	134332442	*	3	0.012827793	-0.051679361	-0.042922001	*NA*
chr6	162683857	162683961	*	3	0.012834102	-0.011669772	-0.007999311	*PRKN*
chr19	29583604	29584548	*	6	0.012847361	0.024747673	0.018482231	*NA*
chr5	139536678	139537207	*	5	0.012852143	0.015101335	0.001175863	*NA*
chr5	1524227	1525455	*	7	0.01285529	0.026418476	0.008036532	*LPCAT1*
chr12	58145750	58146835	*	20	0.012856807	-0.013835674	-0.002652305	*CDK4;MARCHF9*
chr16	88102747	88103629	*	7	0.012856807	-0.028762312	-0.004936401	*BANP*
chr5	129239273	129240801	*	5	0.012862681	-0.021526459	-0.003619484	*CHSY3*
chr12	103344336	103344685	*	5	0.012877699	-0.013485271	-0.002904991	*NA*
chr16	20416684	20417094	*	4	0.012895819	-0.017875828	-0.009092184	*ACSM5;PDILT*
chr5	6755026	6755843	*	7	0.012902935	0.037578489	0.017174702	*TENT4A*
chr17	79026049	79026354	*	5	0.012907236	0.029594001	0.018261855	*BAIAP2*
chr12	133194624	133195520	*	8	0.012913449	-0.043212889	-0.011465485	*P2RX2*
chr14	104761941	104762067	*	3	0.012957857	0.018678565	0.01027791	*NA*
chr16	8941568	8941958	*	5	0.012959026	0.021310775	0.006493613	*PMM2*
chr9	6645468	6645883	*	6	0.012965951	-0.012917396	-0.001918511	*GLDC*
chr1	22263347	22264489	*	9	0.012981323	-0.024909744	-0.013812682	*HSPG2*
chr17	26698549	26699551	*	8	0.012989328	0.011831138	-0.001079944	*SARM1;VTN*
chr2	121104187	121105175	*	5	0.012993219	0.018420969	-0.000397001	*INHBB*
chr7	15601624	15602593	*	4	0.013004357	-0.043118456	-0.018692702	*AGMO*
chr1	26332153	26332550	*	3	0.013011734	0.020132995	0.013883122	*NA*
chr7	3096281	3096561	*	4	0.013022755	-0.016703871	-0.00455502	*NA*
chr14	96115739	96116699	*	3	0.013037328	0.022447574	0.016560749	*TCL6*
chr1	27190639	27191573	*	4	0.01304059	-0.016423061	-0.009145356	*SFN*
chr21	45583245	45584432	*	5	0.013047615	0.038190005	0.018097506	*NA*
chr17	38938637	38938797	*	3	0.013047615	0.021676172	0.006145141	*KRT27*
chr15	100026370	100026867	*	3	0.013057136	-0.014067281	-0.005443153	*NA*
chr12	53399093	53399999	*	9	0.013066888	0.023108147	0.008502329	*EIF4B*
chr17	7380990	7381683	*	3	0.013069052	0.04385392	0.01093495	*SLC35G6;ZBTB4*
chr10	128810484	128810904	*	3	0.013077709	-0.057183472	-0.046745655	*DOCK1*
chr17	75567407	75567709	*	3	0.013100308	-0.009385681	-0.00716431	*NA*
chr18	10862	11855	*	10	0.013134818	0.038117615	0.008585572	*NA*
chr20	3189311	3190132	*	9	0.013151591	-0.024707598	-0.001315572	*DDRGK1;ITPA*
chr9	19050206	19050735	*	3	0.013155829	0.02485462	0.013460908	*RRAGA*

chr19	58458572	58459358	*	13	0.013165471	-0.015141464	-0.002442294	*ZNF256*
chr9	23820979	23822390	*	15	0.013198775	-0.019391761	-0.005176304	*ELAVL2*
chr1	11724048	11724943	*	10	0.013215192	-0.028600254	-0.004279479	*FBXO6*
chr5	170763642	170764118	*	8	0.013234038	-0.033331102	-0.013579482	*NA*
chr1	110284423	110285277	*	5	0.013241692	0.012211215	0.005677444	*GSTM3*
chr11	26353484	26354057	*	8	0.013246689	-0.020279199	-0.011652826	*ANO3*
chr14	105779532	105780431	*	9	0.013251223	-0.019780937	-0.003533005	*BRF1;PACS2*
chr16	89600764	89601214	*	3	0.013256615	-0.017907119	-0.011620113	*SPG7*
chr1	24151609	24152077	*	7	0.01325878	-0.021171187	-0.006875227	*HMGCL*
chr5	149677228	149677639	*	6	0.013265907	0.026311214	0.010425354	*ARSI*
chr12	57483513	57483793	*	3	0.013276409	0.014662717	0.004517439	*NAB2*
chr1	167522981	167523769	*	10	0.013279249	-0.024621066	-0.003672589	*CREG1*
chr1	161193393	161193991	*	3	0.013280095	-0.010423698	0.002929299	*APOA2;MIR5187;TOMM40L*
chr17	5403805	5404581	*	10	0.013314344	-0.028256501	-0.002508521	*LOC728392*
chr3	25469430	25470113	*	12	0.013327246	-0.021824366	-0.006565757	*RARB*
chr2	127782813	127783506	*	8	0.013329868	0.017368614	0.003010514	*NA*
chr13	113426962	113428332	*	8	0.013329868	0.02639654	0.01518674	*ATP11A*
chr22	39240094	39240704	*	7	0.013331391	0.019561737	-0.000985713	*NPTXR*
chr4	55198258	55198664	*	3	0.013339426	-0.017261587	-0.001317472	*NA*
chr7	157605569	157606434	*	3	0.013344815	0.022818521	0.013354639	*PTPRN2*
chr1	208083913	208084255	*	5	0.013348265	-0.010685728	-0.004480106	*CD34*
chr2	74055599	74056290	*	11	0.013362985	-0.020067654	0.001484216	*STAMBP*
chr19	49828293	49828833	*	10	0.013372242	0.038428241	0.014740868	*SLC6A16*
chr1	155161161	155161833	*	4	0.013372242	0.022167875	0.014282719	*MIR92B;MUC1*
chr5	134093972	134094528	*	12	0.013400851	0.011328698	0.000603793	*DDX46*
chr16	619677	620796	*	10	0.013407392	-0.020678752	-0.008099718	*PIGQ*
chr11	31848828	31849262	*	6	0.013407392	-0.025018156	-0.005202663	*PAX6-AS1;RCN1*
chr5	36151174	36151934	*	7	0.013415447	0.01250036	0.002244527	*LMBRD2;MIR580;SKP2*
chr6	70506028	70506758	*	6	0.013415447	0.01286072	0.001178902	*LMBRD1*
chr12	132686912	132687376	*	4	0.013415447	0.022427331	0.013576852	*GALNT9*
chr19	47163979	47164782	*	12	0.013425908	0.011484604	0.001086776	*DACT3;DACT3-AS1*
chr8	600233	600940	*	4	0.013425908	0.048959441	0.02726608	*NA*
chr2	63285097	63285846	*	7	0.013448117	-0.007553457	-0.001215696	*NA*
chr4	153896981	153897705	*	7	0.013448117	0.019793134	0.009925306	*FHDC1*
chr6	30174845	30175327	*	6	0.013448117	0.026314577	0.013382235	*TRIM26*
chr3	69248890	69249718	*	3	0.013452149	-0.025349667	-0.016809815	*FRMD4B*
chr19	55670015	55670466	*	4	0.013456804	0.022985353	0.014074746	*DNAAF3;TNNI3*
chr7	41741320	41741671	*	3	0.013456804	0.022043153	0.010857905	*INHBA;INHBA-AS1*
chr20	1165065	1165703	*	5	0.013456944	-0.018469593	0.000702847	*TMEM74B*
chr1	236156587	236157492	*	6	0.013459484	0.024628706	0.006585031	*NID1*
chr9	123691584	123691850	*	8	0.013463354	0.031069937	0.005887187	*TRAF1*
chr7	1269385	1270134	*	5	0.013463354	-0.015396871	-0.007697616	*UNCX*
chr15	57595298	57595798	*	5	0.013463354	0.01681498	0.000304947	*LINC00926*
chr17	72357992	72358722	*	6	0.01346359	0.017627923	0.012003553	*BTBD17;GPR142*
chr3	52570248	52571040	*	6	0.013480307	-0.009915432	-0.005320337	*NT5DC2;SMIM4*
chr2	97303065	97303765	*	5	0.013487056	-0.020357582	-0.008515275	*FER1L5;KANSL3*
chr2	70484379	70485387	*	17	0.013500753	-0.034960092	-0.005811184	*PCYOX1*
chr11	125772649	125774406	*	12	0.01351936	-0.046172349	-0.008027893	*DDX25;PUS3*
chr1	213224145	213224618	*	9	0.013528103	0.03117484	0.010819463	*RPS6KC1*
chr12	113590790	113591127	*	3	0.013529397	-0.009374338	-0.004520064	*CFAP73*
chr3	51746670	51747098	*	4	0.013562652	0.008582059	0.000661765	*GRM2*
chr12	80083252	80085120	*	14	0.013578925	0.012599635	-0.002794436	*PAWR*
chr1	178455607	178456270	*	5	0.013590973	0.037327088	0.024825651	*NA*
chr16	857576	858499	*	4	0.013590973	-0.051785469	-0.026184066	*PRR25*
chr22	46263018	46263463	*	3	0.013592048	-0.014509844	-0.002658629	*NA*
chr17	19804748	19805143	*	3	0.013595998	0.013813977	0.004966051	*NA*
chr19	3907913	3908397	*	4	0.013607788	0.035695432	0.022009779	*ATCAY*
chr10	132363501	132364391	*	3	0.013609809	-0.030330754	-0.002587444	*NA*
chr3	112737885	112738800	*	15	0.013613103	-0.019326809	-0.002723272	*NEPRO*
chr20	56227286	56227686	*	3	0.013614666	0.011465761	0.008641071	*PMEPA1*
chr22	36894057	36894576	*	6	0.013619659	0.024803437	0.009639268	*FOXRED2*
chr22	17488875	17489762	*	11	0.013622448	0.026950004	0.015543126	*GAB4*
chr8	10261641	10262221	*	6	0.01365872	0.023589207	0.016389206	*MSRA*
chr22	43538857	43539309	*	4	0.013670648	-0.029324006	-0.007025795	*MCAT*
chr4	57773768	57774406	*	7	0.013670869	0.021859292	0.005022823	*REST*
chr2	17699243	17699980	*	9	0.013677953	-0.027094971	-0.007903026	*RAD51AP2*

chr7	100028974	100029306	*	3	0.013677953	-0.025591555	-0.002458335	*MEPCE;ZCWPW1*
chr16	4664854	4665704	*	8	0.013681722	0.023237614	0.004095001	*UBALD1*
chr10	26503817	26504580	*	5	0.013681722	-0.017913928	-0.005274674	*GAD2*
chr14	105612753	105613102	*	3	0.013681722	-0.014002045	0.000144612	*JAG2*
chr5	146832933	146833665	*	5	0.013681986	-0.006298864	-0.000590985	*DPYSL3*
chr15	57025816	57026315	*	8	0.01368458	0.027286226	0.009853586	*ZNF280D*
chr18	42643290	42643746	*	3	0.01368458	0.022325143	0.011146464	*SETBP1*
chr17	1942040	1942194	*	3	0.013719527	-0.020652941	-0.00270316	*DPH1;OVCA2*
chr11	118213272	118213651	*	5	0.013723781	-0.020767116	-0.010227215	*CD3D;CD3G*
chr11	58344749	58345717	*	8	0.013733041	-0.030611479	-0.00166075	*LPXN;ZFP91;ZFP91-CNTF*
chr20	30777790	30778628	*	5	0.01375436	-0.011941806	-0.00262083	*TSPY26P*
chr7	128922791	128922979	*	3	0.013759239	0.024893853	0.011715557	*AHCYL2*
chr1	19972628	19972777	*	3	0.013765124	0.02962816	0.02255287	*MICOS10-NBL1;NBL1*
chr2	44001018	44001411	*	6	0.013765926	-0.025835035	-0.011533369	*DYNC2LI1*
chr8	144680893	144681482	*	5	0.013769974	0.020681023	0.012133677	*EEF1D;TIGD5*
chr17	4634022	4634211	*	4	0.013776222	0.034316322	0.021440755	*MED11*
chr11	119251690	119252980	*	11	0.013809274	-0.023737281	-0.005774169	*USP2;USP2-AS1*
chr6	32938781	32940060	*	25	0.013810037	-0.03751703	-0.000120196	*BRD2*
chr7	35077598	35078364	*	13	0.013815665	-0.042270288	-0.006237773	*DPY19L1*
chr19	1478715	1479573	*	11	0.013821857	0.009252601	0.000768211	*C19orf25*
chr11	57529255	57529614	*	5	0.013828686	-0.01655788	-0.012602087	*CTNND1;TMX2-CTNND1*
chr14	69725831	69726916	*	15	0.013831323	0.017253156	-0.000647747	*GALNT16*
chr4	57301897	57302310	*	8	0.013843489	0.005565151	0.000900945	*PAICS;PPAT*
chr17	78735268	78735596	*	5	0.013843489	-0.02629022	-0.011290118	*RPTOR*
chr5	6449001	6450051	*	6	0.013844979	-0.01887339	0.001165965	*UBE2QL1*
chr1	51442861	51443457	*	6	0.013850902	-0.015316091	-0.001948537	*NA*
chr12	113659329	113660045	*	3	0.013874774	0.016328562	0.006629912	*IQCD;TPCN1*
chr17	41465966	41467066	*	12	0.013884618	-0.016972814	-0.004500566	*LINC00910*
chr14	38066873	38067622	*	3	0.013884618	-0.009288479	-0.006842947	*FOXA1*
chr17	76129331	76130139	*	6	0.013886562	-0.017513821	-0.011182258	*TMC6;TMC8*
chr1	55266578	55267293	*	11	0.013899297	-0.012235376	-0.003135419	*LEXM;TTC22*
chr14	96670738	96671262	*	10	0.01391206	-0.015627039	-0.006457803	*BDKRB2*
chr1	149870975	149872165	*	13	0.013924696	0.03280671	0.00638661	*BOLA1*
chr6	28493019	28493601	*	6	0.013937184	0.024428371	-0.005990673	*GPX5*
chr8	785815	786897	*	7	0.013963634	0.01514868	0.005193054	*NA*
chr6	106429610	106430134	*	4	0.01396577	0.042190245	0.018213129	*NA*
chr6	148663353	148663964	*	11	0.013969201	-0.01691373	-0.001127529	*SASH1*
chr22	21271192	21271516	*	6	0.014016569	-0.009089698	-0.003803574	*CRKL*
chr3	130613286	130614013	*	9	0.014024546	-0.012353331	-0.004352481	*ATP2C1*
chr14	91141279	91141833	*	4	0.014026275	0.033059	0.01122603	*TTC7B*
chr19	54384822	54385441	*	10	0.014036854	0.009222524	-0.002274884	*PRKCG*
chr6	106958474	106960170	*	10	0.014039939	-0.027122989	-0.007294015	*CRYBG1*
chr4	174442784	174443865	*	7	0.014043322	-0.021978218	-0.004709884	*NA*
chr15	89951404	89952499	*	8	0.014060831	-0.016418087	-0.005980816	*NA*
chr7	112089921	112090672	*	11	0.014064224	0.025867181	0.001735034	*IFRD1*
chr2	201727403	201728010	*	4	0.014064393	-0.004387659	-0.0034721	*CLK1*
chr17	42402738	42403239	*	5	0.014124743	-0.011978272	-0.005205882	*SLC25A39*
chr6	43336481	43336734	*	3	0.014136196	0.008989935	0.001089905	*ZNF318*
chr17	55362520	55363072	*	4	0.014152972	-0.01942998	-0.012968719	*MSI2*
chr10	76969779	76971052	*	16	0.014153039	-0.018502573	-0.003186262	*VDAC2*
chr17	1992166	1992876	*	5	0.014191244	0.018471958	0.009957232	*SMG6*
chr7	150499080	150499493	*	4	0.014193868	0.017270723	0.013826354	*TMEM176A;TMEM176B*
chr16	89069935	89070924	*	8	0.014213206	-0.020405546	-0.00437574	*NA*
chr7	55430948	55431276	*	3	0.014213206	0.033155983	0.024060544	*LANCL2*
chr10	65225927	65226682	*	9	0.014213581	-0.021947448	-0.001835834	*JMJD1C;JMJD1C-AS1*
chr5	36693068	36693415	*	3	0.014215554	-0.010970622	-0.007783657	*NA*
chr6	27342060	27342632	*	6	0.014221854	-0.012387996	-0.003909834	*ZNF204P*
chr10	134621538	134622019	*	3	0.014237826	-0.033296525	-0.013190078	*CFAP46*
chr1	53557926	53559001	*	7	0.014246923	0.024808405	0.008923629	*SLC1A7*
chr11	31845639	31846819	*	5	0.014251877	-0.015615395	-0.010449048	*PAX6-AS1;RCN1*
chr16	56665855	56666640	*	7	0.014254414	-0.032796775	-0.00779341	*MT1A;MT1JP;MT1M*
chr6	32804628	32806022	*	14	0.014256889	0.030836577	0.006366892	*TAP2*
chr1	110307811	110307912	*	3	0.014256889	0.018560008	0.010598018	*EPS8L3*
chr9	111929547	111930197	*	6	0.014261511	0.029312687	0.014623718	*FRRS1L*
chr20	46130617	46131560	*	8	0.014271609	-0.032635807	-0.007350472	*NCOA3*
chr16	341185	341404	*	3	0.014273415	0.016746281	0.006744855	*AXIN1;LUC7L*

chr19	17631836	17631991	*	3	0.014281196	0.020344993	0.012508814	*NIBAN3;PGLS*
chr17	48211226	48211745	*	3	0.014287117	-0.02860513	-0.007999406	*PPP1R9B;SAMD14*
chr16	34208209	34209247	*	4	0.01429754	0.040351923	0.027122161	*NA*
chr1	23111011	23111493	*	5	0.014307625	0.020279378	0.013312703	*EPHB2*
chr16	2076057	2076818	*	6	0.014314048	-0.024594984	-0.010674692	*SLC9A3R2*
chr17	58469253	58469739	*	7	0.014315253	0.01439757	-0.000135735	*USP32*
chr7	1491280	1492018	*	7	0.014319799	0.014909544	0.005810545	*MICALL2*
chr2	239059379	239060032	*	5	0.014336401	0.026405123	0.013909654	*KLHL30*
chr16	2132245	2133032	*	5	0.014340935	-0.018382378	-0.006988619	*TSC2*
chr4	84456671	84457956	*	25	0.01438094	-0.021596009	-0.001936121	*GPAT3*
chr2	240884646	240884925	*	4	0.014387675	-0.017397559	-0.010115737	*MIR4786*
chr12	111536320	111537211	*	6	0.014389374	-0.012101773	-0.001682178	*CUX2*
chr6	5026164	5026559	*	6	0.014397687	-0.026319206	-0.011922164	*NA*
chr19	35939523	35940174	*	3	0.014401537	-0.02790452	-0.015243163	*FFAR2*
chr12	112152862	112153283	*	3	0.014401537	-0.02682651	0.000701893	*ACAD10*
chr15	41135432	41136797	*	15	0.014410763	-0.022444572	0.001377423	*SPINT1*
chr6	30796199	30796312	*	4	0.014442372	0.043715712	0.021105698	*NA*
chr1	51795862	51796003	*	3	0.014442372	-0.026552909	-0.01952831	*TTC39A*
chr3	44753069	44754201	*	10	0.014450373	0.028613243	-0.00050368	*ZNF502*
chr1	52869541	52870289	*	12	0.014491037	0.014670341	0.001873977	*ORC1;PRPF38A*
chr9	120659642	120660089	*	3	0.014514605	0.043652879	0.003709319	*NA*
chr11	325964	327178	*	5	0.014515467	-0.044846521	-0.013628395	*NA*
chr18	46287850	46288073	*	4	0.014518019	0.016809412	0.00624362	*CTIF*
chr6	10838284	10839508	*	12	0.014532768	0.018368978	0.001737519	*MAK;SYCP2L;TMEM14B*
chr17	80454611	80455059	*	3	0.014532768	-0.017578813	-0.009473813	*NA*
chr12	6451035	6451316	*	5	0.014542392	-0.006562901	-0.00406223	*TNFRSF1A*
chr2	241611931	241612327	*	4	0.014543368	-0.036850609	-0.025346206	*NA*
chr7	6055517	6056470	*	5	0.014544035	0.036738896	0.013696827	*AIMP2*
chr12	120703085	120704034	*	11	0.01454932	-0.044302892	-0.012228006	*PXN*
chr12	110010943	110011803	*	23	0.014559751	-0.009935017	-0.00206404	*MMAB;MVK*
chr5	172068692	172069173	*	3	0.014574376	-0.019296625	-0.003264223	*NEURL1B*
chr3	48955643	48956213	*	11	0.014581076	0.010945003	0.000381247	*ARIH2;ARIH2OS*
chr6	86303159	86304240	*	13	0.014592513	-0.006491215	-0.000908658	*SNX14*
chr5	163723233	163724070	*	10	0.014594905	-0.034908138	-0.013928053	*NA*
chr22	38092643	38093207	*	12	0.01460411	-0.019186124	-0.008529228	*TRIOBP*
chr1	1275578	1276019	*	3	0.014618938	-0.012467718	-0.001175521	*DVL1*
chr12	13044495	13044862	*	3	0.014626468	0.031794296	0.012614607	*GPRC5A*
chr18	21718458	21718979	*	11	0.014637738	-0.012372507	-0.00275105	*CABYR*
chr2	26624228	26625047	*	12	0.014648711	-0.021272503	-0.005171161	*DRC1*
chr20	48531381	48532315	*	9	0.014658636	0.011848964	-0.001195967	*SPATA2*
chr2	25015896	25016582	*	16	0.014665128	-0.010329772	-0.000777552	*CENPO;PTRHD1*
chr19	2281919	2282928	*	9	0.014665603	-0.036431994	-0.010306299	*PEAK3;SPPL2B*
chr7	73704188	73704691	*	4	0.014684719	-0.046058642	-0.016256182	*CLIP2*
chr5	137609681	137610988	*	13	0.014742575	0.013776257	-0.002825481	*GFRA3*
chr8	70747067	70748063	*	11	0.014742575	0.012047866	-0.001030138	*SLCO5A1*
chr19	1718954	1719126	*	3	0.014742575	-0.01461795	-0.005944917	*NA*
chr3	42948816	42949540	*	3	0.014742575	0.02507518	0.014767528	*ZNF662*
chr10	43892790	43893073	*	9	0.014747203	-0.021770464	-0.006745121	*HNRNPF*
chr4	82136116	82136940	*	10	0.014755267	-0.024370882	-0.00497467	*PRKG2*
chr16	21133049	21133312	*	4	0.01477502	-0.018916847	-0.001549595	*DNAH3*
chr1	231761688	231762737	*	11	0.014784723	-0.016855365	-0.00546985	*DISC1;TSNAX-DISC1*
chr1	26346675	26347092	*	4	0.014788166	-0.011836725	-0.0004805	*EXTL1*
chr2	2735923	2736144	*	3	0.014809164	0.028611744	0.013084746	*NA*
chr16	1206750	1207623	*	5	0.014819439	0.026797741	0.013775736	*CACNA1H*
chr18	55712976	55714393	*	9	0.014825527	-0.025715554	-0.00554801	*NEDD4L*
chr17	27054409	27055188	*	4	0.014825527	-0.024129571	-0.003898915	*NEK8;TLCD1*
chr10	102744788	102745518	*	4	0.014825527	0.049906537	0.018951715	*MRPL43;SEMA4G;TWNK*
chr4	2794616	2794637	*	3	0.014828546	0.012062461	0.004498015	*SH3BP2*
chr20	44098223	44098724	*	7	0.014831954	0.01351641	0.006467206	*WFDC2*
chr7	158649018	158649327	*	8	0.014839411	-0.006892835	0.00010241	*DYNC2I1*
chr20	61810348	61810902	*	5	0.014839411	-0.009428965	-0.001230697	*NA*
chr16	87252369	87253391	*	6	0.014868058	0.014715396	0.011656189	*NA*
chr10	82295394	82295864	*	4	0.01486847	-0.049782025	-0.023170785	*SH2D4B*
chr7	29724677	29725196	*	4	0.014873066	-0.009527668	-0.002633149	*DPY19L2P3;LOC646762;MIR550A3*
chr18	22929205	22930271	*	4	0.014879626	-0.032155999	-0.011307789	*ZNF521*
chr12	12867669	12867916	*	6	0.014884474	0.020110245	0.004714179	*CDKN1B*

chr2	113914442	113914844	*	6	0.014894707	-0.024382934	-0.004915493	*NA*
chr16	81772202	81773196	*	9	0.014910454	0.019267128	-0.000249894	*NA*
chr4	185655229	185655968	*	11	0.014911941	-0.021999152	-0.001593716	*CENPU*
chr4	109097253	109097522	*	3	0.014918855	0.030259788	0.015484353	*LEF1-AS1*
chr22	24122661	24123385	*	4	0.01492082	0.024210971	0.006297171	*MMP11*
chr1	3400412	3400780	*	3	0.014969793	-0.042001989	-0.020048505	*NA*
chr22	38240259	38241182	*	11	0.01500444	-0.021724768	-0.00496468	*ANKRD54;EIF3L;MIR658*
chr6	131949010	131949743	*	10	0.015017454	-0.018181678	-0.004354448	*ENPP3;MED23*
chr10	122610059	122611141	*	10	0.015033829	-0.028001699	-0.008186148	*WDR11;WDR11-AS1*
chr16	850240	850880	*	9	0.015039017	-0.019068261	-0.006550694	*GNG13;PRR25*
chr5	174412095	174412496	*	3	0.015054121	0.019891762	0.004376542	*LINC01951*
chr15	40384779	40385714	*	3	0.015054849	0.025598689	0.015749075	*BMF*
chr19	14785593	14785849	*	5	0.015067354	-0.01910282	-0.010243851	*ADGRE3*
chr2	192015279	192015996	*	10	0.015072685	-0.015679986	-0.006066056	*STAT4*
chr16	2963569	2964815	*	3	0.015087082	0.022183521	0.016428305	*FLYWCH1*
chr20	56725695	56726185	*	12	0.015094127	-0.016800104	0.001054162	*C20orf85*
chr2	176120799	176121126	*	3	0.015110622	-0.028984777	-0.007070403	*NA*
chr1	17445660	17446545	*	12	0.015112615	0.01857413	-0.001682466	*PADI2*
chr7	102988186	102989081	*	5	0.015115772	-0.021164153	-0.011220718	*DNAJC2;PSMC2*
chr11	66838934	66839363	*	5	0.015123639	0.032039886	0.01731722	*RHOD*
chr19	6739268	6740123	*	10	0.015124411	-0.026155777	-0.004188812	*GPR108;TRIP10*
chr6	138104026	138104345	*	3	0.015124411	0.022043926	0.004509472	*NA*
chr5	122424059	122424706	*	6	0.015126618	-0.007791783	-0.000833007	*PRDM6*
chr6	105627412	105628179	*	13	0.015138006	-0.010722148	-0.002887197	*POPDC3*
chr15	28362214	28362747	*	6	0.015147345	0.018963879	0.009380118	*HERC2*
chr20	326934	327623	*	6	0.015147345	0.038278732	0.013434589	*NRSN2*
chr22	17564994	17565612	*	4	0.015147345	-0.010235938	0.00067181	*IL17RA*
chr1	108507910	108508626	*	7	0.015160105	-0.024081519	-0.004567098	*VAV3;VAV3-AS1*
chr17	5973366	5973545	*	3	0.015168	-0.026382987	-0.005841253	*WSCD1*
chr12	131493506	131494244	*	4	0.01518002	0.012024549	0.003221399	*ADGRD1*
chr4	159091762	159092193	*	5	0.015180542	-0.029482702	-0.013167538	*GASK1B*
chr12	133706128	133706422	*	3	0.015180542	-0.040712063	-0.021036067	*ZNF10;ZNF268;ZNF891*
chr7	2594836	2595269	*	3	0.015187714	-0.009888007	-0.001915334	*BRAT1;IQCE*
chr2	3621757	3622594	*	4	0.015190669	-0.017956518	-0.01377825	*RPS7*
chr11	85375153	85376223	*	5	0.015203984	-0.010869025	-0.005553543	*CREBZF*
chr1	2246464	2247066	*	4	0.015246757	0.021305126	0.007404082	*NA*
chr5	108082637	108083489	*	6	0.015253465	-0.02501695	-0.006668381	*FER*
chr2	42652272	42652321	*	3	0.015256149	-0.020565656	-0.015032486	*NA*
chr1	25439789	25440431	*	4	0.015265134	0.016666332	0.001093546	*NA*
chr14	36993517	36994153	*	4	0.015271068	0.010994044	-0.00289728	*NKX2-1;SFTA3*
chr19	44555412	44556485	*	10	0.015306227	-0.020393789	-0.005770178	*ZNF223;ZNF284*
chr13	32605187	32605952	*	13	0.015322811	-0.012014009	-0.002288369	*FRY*
chr4	183713468	183714417	*	6	0.015323338	-0.015213486	-0.001342464	*TENM3*
chr14	42073791	42074635	*	6	0.015323338	-0.014552968	0.000932209	*LRFN5*
chr1	151170706	151171057	*	7	0.015334775	-0.00750867	-0.002884493	*PIP5K1A*
chr17	1157257	1157446	*	5	0.01533545	-0.040151491	-0.016043464	*NA*
chr5	122971759	122972409	*	3	0.015349138	0.014632094	0.013795595	*NA*
chr8	99985888	99986344	*	3	0.015349177	-0.011574938	-0.004524945	*NA*
chr13	33113010	33114005	*	11	0.01535447	-0.028049558	-0.005359328	*N4BP2L2*
chr14	105951604	105952171	*	5	0.015370545	0.019133895	0.004368354	*CRIP1;TEDC1*
chr1	11823776	11824383	*	4	0.01540144	0.013908954	0.00800906	*NA*
chr16	17562419	17563300	*	4	0.015454853	0.024204385	0.017142112	*XYLT1*
chr7	29873786	29874354	*	3	0.015459217	0.022510056	0.015562159	*WIPF3*
chr2	202791155	202791994	*	4	0.015469225	0.019441352	0.003087581	*NA*
chr7	6577467	6577538	*	3	0.015486704	0.017568812	0.013154927	*GRID2IP*
chr7	55313012	55313442	*	3	0.015500026	0.027698784	0.000642195	*NA*
chr1	19746405	19746730	*	3	0.015500026	0.029581022	0.020410742	*CAPZB*
chr16	87669476	87671160	*	9	0.015525023	-0.027901632	0.002545155	*JPH3*
chr2	121670282	121670634	*	3	0.015530163	0.01733509	0.011791595	*GLI2*
chr13	20735819	20736342	*	8	0.015554319	0.027704369	0.00764551	*GJA3*
chr15	64648609	64649139	*	5	0.015565952	0.026684625	0.010151436	*CSNK1G1*
chr12	2144198	2144456	*	3	0.015577371	-0.017733632	-0.01054087	*NA*
chr12	81107990	81108034	*	3	0.0155799	-0.020615756	-0.016492119	*MYF5*
chr4	52916851	52917875	*	12	0.015588807	0.035943058	0.000841697	*SPATA18*
chr10	64578469	64579646	*	10	0.015588807	0.030732584	0.010277401	*EGR2*
chr6	111407833	111409547	*	14	0.015611012	0.012210619	4.80E-05	*SLC16A10*

chr7	97755534	97755792	*	6	0.015613896	-0.022728012	-0.012435471	*LMTK2*
chr2	172289750	172289953	*	3	0.015614554	0.026072842	0.009682355	*DCAF17;METTL8*
chr5	50265600	50266394	*	9	0.015635516	-0.035803721	-0.006214607	*NA*
chr2	25391274	25392556	*	12	0.015649503	0.020298174	0.006461689	*POMC*
chr18	63417605	63418672	*	7	0.015649503	-0.010108681	-0.005303876	*CDH7*
chr14	104263779	104264228	*	3	0.015670475	-0.021198238	-0.019126426	*PPP1R13B*
chr2	29320166	29320905	*	9	0.015673688	-0.018740775	-0.002872542	*CLIP4*
chr19	1295582	1295929	*	4	0.015677342	-0.015067347	-0.0084535	*EFNA2*
chr1	22974053	22975587	*	7	0.01567774	0.026309555	0.012660411	*C1QB;C1QC*
chr11	77185354	77185761	*	5	0.01568025	-0.026977406	-0.008610754	*PAK1*
chr2	112918222	112918552	*	3	0.015691156	0.026542521	0.016267937	*FBLN7*
chr8	142264072	142264769	*	7	0.015693308	0.020723778	0.005379178	*SLC45A4*
chr10	89266785	89267405	*	5	0.015699255	0.026610622	0.009310995	*MINPP1*
chr2	208104158	208104518	*	3	0.015707532	-0.015494818	-0.010826447	*NA*
chr11	67169070	67169884	*	12	0.015710918	-0.046887515	-0.007277251	*PPP1CA;TBC1D10C*
chr3	169377818	169379266	*	9	0.015717961	-0.011590706	-0.005224765	*MECOM*
chr3	71592056	71592501	*	3	0.015722826	-0.021273103	-0.017382423	*FOXP1;MIR1284*
chr11	17229012	17229662	*	5	0.015730437	-0.010513323	-0.002433849	*PIK3C2A*
chr7	1498641	1499734	*	11	0.015777693	-0.020167433	-0.008059633	*MICALL2*
chr11	47511134	47511307	*	3	0.015784784	-0.01383469	-0.007644797	*CELF1*
chr4	1350275	1350545	*	4	0.015790653	-0.035949811	-0.012244029	*UVSSA*
chr2	47796781	47797963	*	9	0.015805613	-0.020706331	-0.001843915	*KCNK12;MSH2*
chr16	86016317	86017380	*	4	0.015805613	-0.020357819	-0.014070663	*NA*
chr10	106074540	106075560	*	5	0.015818829	0.020055466	0.010092295	*ITPRIP*
chr17	2075719	2076468	*	4	0.01582005	0.024122546	-0.000663497	*SMG6*
chr17	6543996	6544903	*	16	0.015838378	0.007035378	-0.001022808	*KIAA0753;TXNDC17*
chr2	217236040	217237043	*	15	0.015838499	-0.012421226	-0.00342155	*MARCHF4*
chr22	39745530	39745743	*	5	0.01585182	-0.016142994	-0.007809739	*SYNGR1;TAB1*
chr4	69215865	69217218	*	13	0.015865126	-0.034068167	-0.004755765	*YTHDC1*
chr7	29603056	29603541	*	10	0.015881383	-0.012755054	-0.002462706	*PRR15*
chr19	1466497	1467032	*	5	0.015910407	-0.027584093	-0.010799686	*APC2;C19orf25*
chr15	78527113	78527410	*	3	0.015918653	0.021072339	0.012097764	*ACSBG1*
chr22	50511322	50511738	*	4	0.015940067	0.017901394	0.0134704	*MLC1*
chr7	138720457	138721065	*	8	0.015974105	0.013242762	0.001531656	*ZC3HAV1L*
chr15	93182492	93182916	*	4	0.015975149	0.036812633	0.011303565	*FAM174B*
chr3	67345234	67345761	*	3	0.015976425	-0.01461824	-0.003517782	*NA*
chr8	144461411	144462235	*	6	0.016029982	0.021769343	0.011467957	*RHPN1*
chr19	46009775	46010241	*	5	0.01605191	0.01076674	0.001513763	*VASP*
chr17	1104627	1104805	*	3	0.01605191	0.021135135	0.01606965	*NA*
chr11	995292	996011	*	4	0.016052788	0.015004112	0.012689371	*AP2A2*
chr10	38382849	38383804	*	7	0.01605825	-0.007998821	-0.001022333	*ZNF37A*
chr3	196014280	196015218	*	12	0.016059809	0.017760704	0.001106246	*PCYT1A*
chr6	32062554	32063126	*	6	0.01607147	-0.011645117	0.002235441	*TNXB*
chr9	23826065	23826507	*	6	0.01607695	-0.014070601	-0.00721824	*ELAVL2*
chr13	36920332	36920813	*	7	0.016079977	-0.014445753	-0.004110771	*SPART;SPART-AS1*
chr21	39576893	39577464	*	3	0.016086671	0.021811522	0.00352942	*DSCR10*
chr12	130823570	130824529	*	7	0.016102975	-0.054632501	-0.02054944	*PIWIL1*
chr11	637885	638974	*	6	0.016111862	0.042733292	0.027023697	*DRD4*
chr15	74218217	74218921	*	11	0.016118906	0.019318807	0.007303093	*LOXL1;LOXL1-AS1*
chr1	222987498	222988866	*	11	0.016126087	-0.036110765	-0.005931124	*DISP1*
chr10	43476985	43477278	*	4	0.016126196	0.022559727	0.012409395	*NA*
chr4	26861261	26862605	*	11	0.016131417	0.03784561	0.000279439	*STIM2*
chr2	217351940	217352368	*	4	0.016133929	0.02802152	-0.000855483	*NA*
chr1	85527754	85528044	*	9	0.016135762	-0.019498357	-0.006805399	*DNAI3*
chr7	149569946	149571732	*	22	0.016153509	-0.027520029	-0.004830772	*ATP6V0E2;ATP6V0E2-AS1*
chr12	41582961	41583721	*	4	0.016226917	0.023257777	0.001282095	*PDZRN4*
chr4	75859672	75860346	*	5	0.016242323	-0.017558601	-0.002236307	*PARM1*
chr12	122617356	122618405	*	10	0.016251873	0.022620421	0.002689206	*MLXIP*
chr2	240112249	240113010	*	8	0.016251873	0.053765824	0.018335193	*HDAC4*
chr2	99061081	99061575	*	7	0.016251873	-0.016358762	-0.003103006	*INPP4A*
chr19	51412615	51412749	*	3	0.016251873	-0.008154485	-0.004306374	*KLK4*
chr1	1897585	1897785	*	3	0.016267642	0.032652794	0.016022089	*CFAP74*
chr11	9780844	9781412	*	4	0.016286908	0.029284102	0.012922106	*LINC02709;SBF2-AS1*
chr19	19322527	19323330	*	9	0.01633783	-0.026506579	-0.001080694	*NCAN*
chr4	157996959	157997750	*	13	0.016354798	-0.02281362	-0.004185701	*GLRB*
chr17	14203652	14204593	*	8	0.016355837	-0.010844978	-0.004262622	*HS3ST3B1;MGC12916*

chr12	53183508	53184277	*	7	0.016357285	-0.021994736	-0.010344412	*KRT3*
chr19	51920885	51921812	*	5	0.016369159	-0.016334607	-0.001889941	*SIGLEC10*
chr4	141047768	141048293	*	3	0.016384514	0.018791505	0.011157601	*MAML3*
chr2	129079038	129080120	*	7	0.016388851	0.021776011	0.007163689	*HS6ST1*
chr6	28191910	28193011	*	8	0.016409569	0.019901405	0.002081703	*ZSCAN9*
chr10	118563568	118563859	*	3	0.016448702	0.020533592	0.004800514	*HSPA12A*
chr20	61038949	61039348	*	3	0.016448916	0.02917538	0.01759386	*GATA5*
chr19	47288915	47289611	*	4	0.016454982	0.02102271	0.010685495	*SLC1A5*
chr5	1128038	1128391	*	3	0.016459527	0.018528048	0.006060769	*NA*
chr9	127273145	127273395	*	3	0.016470557	0.014478378	0.000299654	*NR5A1*
chr1	91172930	91173232	*	4	0.016478384	-0.01711019	-0.009511949	*NA*
chr7	84815088	84815940	*	6	0.01648163	0.016914797	0.00045142	*SEMA3D*
chr12	133020746	133021713	*	5	0.016494302	-0.031105061	-0.009368539	*NA*
chr3	111392915	111393685	*	11	0.01650544	-0.015026806	-0.005003891	*PLCXD2*
chr4	941966	942342	*	4	0.016513859	0.020849089	0.008442057	*TMEM175*
chr12	122658311	122659253	*	8	0.016525724	0.020100984	0.009105564	*IL31;LRRC43*
chr22	18924903	18925277	*	3	0.016526937	0.021649004	0.011175563	*PRODH*
chr2	213401544	213403678	*	16	0.016536416	-0.013473177	-0.00440682	*ERBB4*
chr19	46142357	46143348	*	10	0.016550535	-0.03393861	-0.009503436	*EML2;EML2-AS1;MIR330*
chr4	41114478	41115076	*	3	0.016572505	0.023842404	0.006893878	*APBB2*
chr3	15466687	15467412	*	5	0.016583476	-0.028927395	-0.002115854	*EAF1;METTL6*
chr22	31522682	31522712	*	3	0.016589053	0.01660448	0.012253233	*INPP5J*
chr10	86087948	86088681	*	11	0.016606641	-0.029675272	-0.011798635	*CCSER2*
chr21	15442816	15443107	*	3	0.016623568	0.018681825	0.014141887	*NA*
chr17	27048223	27048708	*	4	0.016651439	0.036635622	0.017142658	*RAB34;RPL23A;SNORD42A;SNORD4A;SNORD4B*
chr14	22362071	22362473	*	5	0.016666263	0.0116121	7.80E-05	*NA*
chr17	139511	140192	*	3	0.016666263	0.034447463	0.023274305	*RPH3AL*
chr7	44142953	44143290	*	4	0.016668292	-0.016297759	-0.002524579	*AEBP1*
chr22	33454623	33455507	*	4	0.016674801	0.036663809	-0.002032481	*SYN3*
chr14	73602752	73603844	*	16	0.016682628	0.018218811	0.002379164	*PSEN1*
chr13	112187145	112187396	*	4	0.016682628	-0.0234475	-0.017324296	*NA*
chr7	156796145	156796929	*	3	0.016710722	0.00922675	0.005988325	*MNX1*
chr16	33964783	33965573	*	5	0.016742999	0.040383601	0.023394655	*LINC00273*
chr10	101190311	101191565	*	12	0.016749364	-0.030298277	-0.001564959	*GOT1*
chr3	74571102	74571266	*	3	0.016766462	-0.017289563	-0.004798573	*CNTN3*
chr5	173043505	173044411	*	13	0.016787162	-0.030893511	-0.000348991	*BOD1*
chr13	95355117	95355869	*	6	0.016788243	-0.021130435	-0.007552447	*NA*
chr2	172016605	172017432	*	6	0.016793113	-0.01132714	-0.002847247	*TLK1*
chr1	59483475	59484803	*	5	0.016804743	-0.03552659	-0.01652936	*NA*
chr11	16628335	16629289	*	8	0.016816268	-0.013051322	-0.006822585	*SOX6*
chr5	180477311	180478185	*	4	0.016818272	-0.032745269	-0.007701129	*BTNL9*
chr10	134771944	134772055	*	3	0.016828338	-0.03493304	-0.014793249	*NA*
chr1	247275326	247276096	*	12	0.01684015	0.02617706	0.003993814	*C1orf229*
chr1	244013668	244014035	*	5	0.016844667	-0.01761009	-0.006385227	*NA*
chr10	131200784	131200834	*	3	0.016849089	-0.011932233	-0.004786383	*NA*
chr1	160808959	160809639	*	4	0.016856701	-0.027002684	-0.012691501	*CD244*
chr7	100491714	100492700	*	7	0.016867182	0.012700099	0.005301492	*ACHE;UFSP1*
chr17	1509247	1510080	*	6	0.016895659	0.021340685	0.013276123	*SLC43A2*
chr2	107501733	107502146	*	3	0.016923254	-0.007793039	-0.001851372	*ST6GAL2*
chr7	97804327	97804838	*	3	0.016926456	-0.024426028	-0.007530598	*LMTK2*
chr10	6962119	6962454	*	6	0.016934532	-0.027269922	-0.010176799	*NA*
chr1	32891825	32892367	*	3	0.01696712	-0.024788067	0.000197265	*NA*
chr17	16310297	16310862	*	3	0.017003069	-0.019698936	-0.006723236	*NA*
chr16	81253692	81254719	*	9	0.01702391	0.041991051	0.016327684	*PKD1L2*
chr8	117767944	117769166	*	9	0.017046069	-0.026708775	-0.000508285	*EIF3H*
chr8	145550361	145550989	*	8	0.017064322	-0.012324353	-0.003511989	*DGAT1*
chr1	116270626	116271272	*	4	0.017072496	0.02534952	-0.004029566	*CASQ2*
chr5	124064272	124064481	*	3	0.017076714	-0.01824658	-0.00312009	*ZNF608*
chr17	79086355	79086564	*	4	0.017080996	0.015654972	0.010809934	*BAIAP2*
chr10	22609629	22610507	*	8	0.017086309	-0.024031625	-0.006108234	*BMI1;COMMD3-BMI1*
chr7	157637416	157637560	*	3	0.017094674	-0.020549724	-0.004167866	*PTPRN2*
chr19	4152651	4152810	*	3	0.017108224	-0.016747944	-0.00878935	*CREB3L3*
chr16	89988910	89989486	*	7	0.017115229	0.022317945	-0.000888394	*TUBB3*
chr1	24882347	24882991	*	7	0.017125944	-0.030667112	-0.011883686	*NCMAP*
chr19	42787762	42788966	*	9	0.01714754	-0.028071049	-0.008931107	*CIC*
chr18	2912735	2913254	*	6	0.017177858	0.019462273	0.013789838	*EMILIN2*

chr4	13543730	13544301	*	3	0.017206514	-0.015525409	-0.007623959	*NKX3-2*
chr5	56471120	56471598	*	3	0.017206514	-0.006448515	-0.000889711	*GPBP1*
chr5	72415900	72416677	*	11	0.017218797	-0.025169123	-0.008992028	*TMEM171*
chr13	114100469	114100961	*	3	0.017225917	0.021524846	-0.000609043	*ADPRHL1*
chr4	1348302	1348867	*	3	0.017229062	-0.015157962	-0.004349205	*UVSSA*
chr5	1316038	1316636	*	3	0.017238788	-0.021690578	-0.014272679	*NA*
chr18	44676421	44677236	*	12	0.017241699	-0.01021176	-0.001235376	*HDHD2*
chr5	54467244	54467799	*	3	0.017244858	-0.032665537	-0.013055571	*CDC20B;MIR449A;MIR449B*
chr18	48085453	48086162	*	6	0.017253809	0.026191382	0.000240705	*MAPK4*
chr11	105893010	105893693	*	9	0.017280649	-0.016183888	-0.004286383	*MSANTD4*
chr12	123458650	123459401	*	15	0.017325873	-0.014227306	-0.001808053	*ABCB9;OGFOD2*
chr16	87798736	87799191	*	3	0.017338757	-0.025673856	-0.008444809	*KLHDC4*
chr13	28498878	28499045	*	3	0.017338757	0.029504644	0.009500049	*PDX1*
chr7	5553266	5553627	*	5	0.01737238	-0.01410206	-0.000603523	*FBXL18*
chr1	17846708	17846815	*	4	0.01737238	-0.025788521	-0.013006767	*ARHGEF10L*
chr16	570658	571091	*	4	0.01737238	-0.013920436	-0.000552017	*RAB11FIP3*
chr19	42509801	42510093	*	4	0.01737543	0.028232972	0.018276016	*GRIK5*
chr15	62358531	62359539	*	10	0.017377839	-0.009804463	-0.00338342	*C2CD4A*
chr3	128967393	128968397	*	8	0.017393077	0.010786235	0.001081439	*COPG1*
chr1	1552883	1553405	*	3	0.017394906	0.014715774	0.001738407	*MIB2*
chr4	113970506	113971324	*	8	0.017397617	-0.019701653	-0.008737109	*ANK2*
chr15	75181100	75181724	*	3	0.017424097	0.01724503	0.012686805	*MPI*
chr1	235667507	235668421	*	10	0.017429291	-0.015330754	-0.001583664	*B3GALNT2*
chr18	9334488	9334792	*	7	0.017470344	-0.032551032	-0.020711958	*TWSG1*
chr14	105973846	105974580	*	6	0.017485814	0.018134073	0.004044643	*NA*
chr17	75318211	75319068	*	5	0.017485814	-0.021389832	-0.006742646	*SEPTIN9*
chr6	42846967	42847910	*	14	0.017492174	-0.015510619	-0.004673888	*RPL7L1*
chr6	139095001	139095535	*	5	0.017492707	0.013168295	0.002768892	*CCDC28A;CCDC28A-AS1*
chr11	120080780	120081089	*	3	0.017524834	-0.011749118	-0.005973976	*OAF*
chr5	157078082	157078881	*	6	0.017532081	0.018705158	0.010636499	*SOX30*
chr13	108520039	108520566	*	6	0.01753373	-0.022739501	-0.005545751	*FAM155A*
chr7	97978189	97978730	*	4	0.01753373	0.011917337	0.009343492	*BAIAP2L1*
chr1	25235737	25236445	*	5	0.01753406	0.027438739	0.009460198	*RUNX3*
chr4	141489799	141490428	*	11	0.017552687	-0.022318942	-0.007174007	*UCP1*
chr12	63544752	63545288	*	7	0.017562911	-0.018019331	-0.009632492	*AVPR1A*
chr1	118472455	118473064	*	7	0.017562911	0.024445515	0.001650884	*GDAP2;WDR3*
chr9	139565026	139565154	*	4	0.017562911	0.077078558	0.026867265	*EGFL7;MIR126*
chr16	70323425	70323915	*	11	0.017565018	0.018998532	0.000210156	*AARS1;DDX19B*
chr9	139222119	139222968	*	4	0.017584235	-0.025576965	-0.016630387	*DKFZP434A062;GPSM1*
chr1	151693199	151693503	*	4	0.017584852	-0.020680079	-0.008653909	*CELF3;RIIAD1*
chr3	71180064	71180859	*	8	0.017597879	-0.025243793	-0.003848981	*FOXP1*
chr19	57741934	57742444	*	12	0.017602545	0.040156479	0.023228562	*AURKC*
chr11	107460942	107462721	*	15	0.017605429	0.027570624	-0.002674614	*ELMOD1*
chr17	42147463	42148292	*	14	0.017639446	-0.017628326	-0.006098875	*G6PC3;LSM12*
chr19	13049949	13050931	*	4	0.01764904	0.013240294	0.004200455	*CALR*
chr3	158526734	158527481	*	3	0.017649451	-0.031153534	-0.016262298	*MFSD1*
chr18	74844767	74845422	*	6	0.017668054	0.052602463	0.004255687	*MBP*
chr22	29976613	29977322	*	7	0.017695847	-0.010845002	-0.002449108	*NIPSNAP1*
chr4	90815958	90816310	*	3	0.017742718	-0.024335518	-0.016234938	*MMRN1*
chr20	61309080	61309743	*	4	0.01774277	-0.03134785	-0.019275108	*NA*
chr6	44191036	44191600	*	15	0.017770756	-0.0302223	-0.008236384	*SLC29A1*
chr13	47370918	47371987	*	14	0.017770756	-0.01323312	-0.005188019	*ESD*
chr5	140018682	140019269	*	10	0.017772391	0.028779392	0.002539135	*TMCO6*
chr10	483387	483555	*	3	0.017778432	-0.021023073	-0.015206421	*DIP2C*
chr9	71394802	71395167	*	6	0.01779383	-0.011553688	-0.000484875	*FAM122A;PIP5K1B*
chr5	178899789	178899850	*	3	0.017809749	0.030844676	0.019349239	*NA*
chr3	50192725	50193465	*	5	0.017819133	0.016588168	0.001704089	*SEMA3F*
chr4	77069145	77070069	*	10	0.01783348	0.019694653	0.002164286	*NUP54*
chr11	746771	747111	*	5	0.017840028	-0.007493793	-0.002849206	*TALDO1*
chr17	7486551	7487249	*	15	0.017847256	0.033402822	0.005875189	*MPDU1*
chr14	93034791	93035095	*	3	0.017850399	-0.02409529	-0.00144076	*RIN3*
chr2	207802453	207803339	*	4	0.017854338	0.019771825	0.006583908	*CPO*
chr1	68299388	68299854	*	7	0.017857488	0.041363477	0.016028062	*GNG12;GNG12-AS1*
chr15	32965273	32966463	*	5	0.017869019	-0.023439475	-0.004585734	*SCG5*
chr12	121905538	121905955	*	3	0.017869019	0.020108317	0.004577443	*KDM2B*
chr21	47333731	47334174	*	4	0.017899042	0.018942923	0.010475115	*PCBP3*

chr2	101618129	101619257	*	18	0.017899138	-0.022393115	0.000546501	*RPL31*
chr15	25491792	25492469	*	6	0.017899895	-0.024339552	-0.003292659	*SNORD115-10;SNORD115-11;SNORD115-44*
chr11	55736968	55737423	*	3	0.017948955	-0.024726708	-0.012476345	*OR10AG1*
chr5	159434503	159435433	*	4	0.017950098	-0.014936158	-0.006069213	*TTC1*
chr5	14325937	14326531	*	5	0.0179577	-0.027014315	-0.008624694	*TRIO*
chr17	42286849	42288203	*	6	0.017960025	-0.025504554	0.002184046	*UBTF*
chr1	186344058	186344890	*	11	0.017963748	0.006558264	-0.000396433	*MIR548F1;ODR4;TPR*
chr13	45564065	45564444	*	5	0.017963748	-0.014119142	-0.00616195	*GPALPP1;NUFIP1*
chr5	126625742	126626092	*	4	0.017963748	-0.011893257	-0.003818342	*MEGF10*
chr1	247580074	247580310	*	3	0.017963748	-0.011673929	-0.005882867	*NLRP3*
chr6	28481660	28482191	*	4	0.01796893	-0.016919063	-0.006714411	*GPX6*
chr19	17970232	17971045	*	14	0.018001052	-0.006530033	-0.002774835	*RPL18A;SNORA68*
chr2	85581378	85581920	*	12	0.018008299	0.009131038	0.000745285	*ELMOD3;RETSAT*
chr15	40633124	40633416	*	7	0.018018707	-0.018355189	-0.006445242	*CCDC9B*
chr7	134854206	134855024	*	7	0.01802838	0.031493405	0.00962584	*CYREN*
chr2	219263149	219263515	*	4	0.018036704	-0.005661267	-0.00312489	*CTDSP1;MIR26B*
chr8	144242029	144242904	*	8	0.018058049	0.028915429	-0.00273884	*LY6H*
chr19	47968997	47969574	*	6	0.018063899	0.025940207	0.01628001	*SLC8A2*
chr8	143545789	143546524	*	4	0.018070376	-0.027831799	-0.014799656	*ADGRB1*
chr11	17099044	17099688	*	10	0.01811509	-0.008133086	-0.002612872	*PIK3C2A;RPS13*
chr5	72111910	72112008	*	5	0.018117465	0.01000012	0.000466212	*TNPO1*
chr13	100258165	100258641	*	3	0.018117465	-0.014930533	-0.007317534	*CLYBL*
chr12	131265728	131266253	*	3	0.018155343	0.024187098	0.015900557	*NA*
chr6	154677796	154678326	*	4	0.018170306	-0.019058015	-0.006560056	*IPCEF1*
chr22	45809244	45809952	*	14	0.018179026	-0.050377792	-0.006554153	*RIBC2;SMC1B*
chr1	167905644	167906565	*	14	0.018182207	-0.022254794	-0.00072028	*DCAF6;MPC2*
chr15	44969244	44969539	*	4	0.018191271	-0.021904301	-0.013259162	*PATL2*
chr14	101513572	101514051	*	4	0.018191348	0.018971144	0.010657421	*MIR487A;MIR539;MIR655;MIR889*
chr10	3282585	3282783	*	3	0.018194186	0.042427159	0.041105344	*NA*
chr10	49514029	49514546	*	9	0.018225888	-0.028071	-0.00395198	*MAPK8*
chr19	37568732	37569273	*	8	0.018238788	0.005794685	-0.001068179	*ZNF420*
chr17	27506970	27508027	*	12	0.018261471	0.014840225	0.003544726	*MYO18A*
chr15	93588328	93588851	*	3	0.01828131	0.019301097	0.012093028	*RGMA*
chr19	10668268	10668565	*	4	0.018293618	0.025910973	0.014096203	*KRI1*
chr17	46709858	46710112	*	4	0.018299231	0.026733942	0.01358634	*MIR196A1*
chr19	46105314	46106056	*	9	0.018310884	-0.038073998	-0.003920695	*GPR4;OPA3*
chr7	135346802	135347371	*	10	0.01831216	0.008259255	0.00043898	*STMP1*
chr5	172661803	172662463	*	8	0.018314647	-0.020279373	-0.004234509	*NKX2-5*
chr14	68162436	68163086	*	9	0.018315587	-0.015267949	-0.006403505	*RDH11*
chr1	3352257	3352986	*	6	0.018318273	0.023997365	0.003134458	*PRDM16*
chr11	85469037	85470458	*	7	0.018327106	-0.024005596	-0.009379114	*SYTL2*
chr11	124709223	124709925	*	6	0.018327106	-0.012588612	-0.001721694	*NA*
chr1	24828096	24828968	*	9	0.018341652	-0.01471887	-0.001928776	*RCAN3;RCAN3AS*
chr18	43684692	43685360	*	8	0.018371617	0.023011904	0.006261931	*ATP5F1A;HAUS1*
chr11	2292751	2293665	*	21	0.018397182	-0.029224987	0.006426938	*ASCL2*
chr12	132312106	132312875	*	9	0.018397182	-0.020014227	-0.004569365	*MMP17*
chr4	4867164	4867979	*	6	0.018397182	-0.027378867	-0.005196785	*NA*
chr1	16533422	16533907	*	5	0.018397182	-0.011502003	-0.004609876	*ARHGEF19*
chr5	56718310	56718431	*	3	0.018397182	0.015191855	-0.003048562	*NA*
chr2	178013511	178013807	*	3	0.018404061	-0.039487952	-0.014305909	*NA*
chr6	2916614	2917294	*	3	0.018405801	0.033161089	0.011261725	*NA*
chr10	71562314	71562858	*	4	0.018412417	0.010012561	0.003433891	*COL13A1*
chr8	494913	496128	*	8	0.018413901	-0.026377795	-0.000877672	*TDRP*
chr1	154600131	154600788	*	9	0.018415494	0.015632681	0.003706114	*ADAR*
chr14	105181035	105181902	*	6	0.018437635	0.019250939	0.006062707	*INF2*
chr16	88797119	88797395	*	3	0.018445716	-0.013562964	-0.007302537	*PIEZO1*
chr15	67068074	67068378	*	3	0.018446775	0.018072919	0.010147396	*SMAD6*
chr14	56045827	56046098	*	4	0.018453144	-0.035106922	-0.023657188	*KTN1;KTN1-AS1*
chr17	1028394	1030700	*	9	0.018457625	0.02181186	0.01193168	*ABR*
chr6	28641651	28642394	*	11	0.01845801	-0.025523405	-0.008004312	*NA*
chr12	49657537	49658425	*	5	0.01848741	-0.018910069	-0.00215486	*TUBA1C*
chr17	746502	746641	*	3	0.01848741	-0.032685744	-0.02570277	*NXN*
chr19	5838731	5838999	*	7	0.018487464	-0.021411411	-0.01144221	*FUT6*
chr2	131113015	131113730	*	5	0.018493918	-0.008546816	0.001116627	*PTPN18*
chr14	57263369	57264639	*	7	0.018528793	-0.020299915	-0.010033053	*NA*
chr1	9687064	9687471	*	4	0.018555905	0.007602746	-0.001390112	*NA*

chr22	37257124	37257594	*	3	0.018576396	0.018086884	0.01301716	*NCF4*
chr19	11909183	11910049	*	8	0.018579646	-0.010877961	-0.006526617	*ZNF491*
chr1	62660624	62660861	*	4	0.018583069	0.034600267	0.014282321	*L1TD1*
chr14	57046206	57046857	*	9	0.018643335	-0.008527562	-0.002294002	*TMEM260*
chr11	124823283	124824151	*	13	0.018667037	0.034506519	0.004541203	*CCDC15*
chr15	73734744	73735694	*	14	0.018680517	0.036714619	0.008652229	*REC114*
chr22	47370132	47370307	*	3	0.018687238	0.021387229	0.015457873	*TBC1D22A*
chr6	86387318	86388072	*	6	0.01868845	-0.051306562	-0.013005225	*SNHG5;SNORD50A;SNORD50B*
chr17	3539439	3540440	*	18	0.018690066	0.012570791	0.000123372	*CTNS;SHPK*
chr16	89408076	89408567	*	4	0.018698258	0.016608334	0.010433756	*ANKRD11*
chr2	85956392	85957232	*	3	0.018710888	-0.014491553	-0.008831565	*NA*
chr12	58003378	58004248	*	8	0.0187119	0.018255546	0.003108438	*ARHGEF25;DTX3*
chr19	35605465	35605867	*	4	0.018724474	-0.018084851	-0.01096561	*FXYD3*
chr16	1420317	1420537	*	3	0.018742964	0.024061021	0.017596268	*UNKL*
chr13	110438256	110438937	*	7	0.018766271	-0.028219043	-0.012984824	*IRS2*
chr6	28870818	28871376	*	3	0.018772601	0.019304977	0.014981184	*TRIM27*
chr19	48917816	48918712	*	4	0.018774603	0.02034029	0.001176329	*GRIN2D*
chr20	12225088	12225421	*	5	0.018818314	0.014789948	0.009492125	*NA*
chr15	45003560	45004577	*	11	0.018831132	-0.011963863	-0.001326619	*B2M;PATL2*
chr16	3629620	3630518	*	4	0.018831132	0.031715863	0.007440333	*NLRC3*
chr1	41350014	41350406	*	3	0.018831132	0.02991423	0.018782291	*NA*
chr15	83316908	83317632	*	10	0.018844245	0.030067072	0.010610198	*CPEB1;CPEB1-AS1*
chr2	53994559	53995389	*	11	0.018845039	0.01365091	0.002544032	*ASB3;CHAC2*
chr1	39284267	39284609	*	4	0.018845039	0.019346523	0.00664152	*NA*
chr13	50569769	50570250	*	4	0.018853576	-0.016196007	-0.009859161	*DLEU2;TRIM13*
chr14	104645554	104646441	*	6	0.018857	0.020308419	0.013643876	*KIF26A*
chr6	50683593	50683782	*	4	0.018875399	-0.01663975	-0.013335497	*TFAP2D*
chr12	114837572	114838359	*	5	0.018878985	0.020341052	0.002552369	*TBX5*
chr22	30987482	30988090	*	9	0.018883593	-0.009904914	-0.00111616	*PES1*
chr15	69687028	69687372	*	3	0.018883593	-0.006681902	-0.006351084	*PAQR5*
chr5	149864599	149865429	*	3	0.018904766	0.027080098	0.014744109	*NA*
chr22	31158835	31159371	*	4	0.018908586	0.026544852	0.010670688	*OSBP2*
chr1	227746882	227747468	*	3	0.018916628	0.03183157	0.021092763	*ZNF678*
chr1	201617042	201617355	*	4	0.018959853	0.045409489	0.020483596	*NAV1*
chr12	54331892	54332222	*	5	0.018972664	-0.022347657	-0.008680415	*HOXC13;HOXC13-AS*
chr1	2936277	2937278	*	6	0.018978728	0.015868961	0.006858361	*ACTRT2*
chr4	182717776	182717992	*	3	0.018990589	-0.027983776	-0.01135156	*NA*
chr22	36011405	36012221	*	5	0.019010226	0.022207841	-0.00238176	*MB*
chr21	46895183	46895659	*	3	0.019013089	0.018929169	0.013792537	*COL18A1*
chr4	160699894	160700056	*	3	0.019062917	-0.012524292	-0.008322909	*NA*
chr20	57556052	57556415	*	5	0.019096026	-0.030062269	-0.015942028	*NELFCD*
chr11	68450618	68451280	*	3	0.019099042	0.024650615	0.013113029	*GAL*
chr5	133967994	133968721	*	10	0.019103417	-0.020283571	-0.001466476	*SAR1B*
chr6	34112244	34114384	*	14	0.019110843	0.023672887	0.006354086	*GRM4*
chr1	230850821	230851153	*	3	0.019115753	-0.018717456	-0.004069258	*AGT*
chr13	114005848	114006027	*	5	0.019120763	-0.022700884	-0.006503834	*GRTP1*
chr11	64127791	64128339	*	3	0.019143524	-0.014980397	-0.006197829	*RPS6KA4*
chr12	83080208	83080830	*	6	0.019152157	-0.005479191	-0.001020409	*TMTC2*
chr4	108910565	108911521	*	12	0.019168186	-0.010847322	-0.001368338	*HADH*
chr2	231712722	231712981	*	4	0.019188117	0.032604284	0.007389663	*NA*
chr10	60094109	60094570	*	5	0.019194509	0.023983487	0.009779717	*UBE2D1*
chr8	142238452	142239055	*	8	0.019233596	-0.019662935	0.003369726	*SLC45A4*
chr6	49430596	49431435	*	15	0.019236081	0.014942901	0.002518525	*CENPQ;MMUT*
chr20	58660759	58661301	*	3	0.019243304	0.030136344	0.015323037	*NA*
chr20	37075704	37076176	*	3	0.019257014	0.017781756	0.00769438	*SNHG11;SNORA71E*
chr15	68346336	68346924	*	11	0.019278062	0.006871305	-0.000596404	*PIAS1*
chr6	40345751	40346299	*	5	0.019308891	0.039603901	0.017396618	*TDRG1*
chr6	32056058	32056735	*	10	0.019319408	0.01814622	0.003481858	*TNXB*
chr5	52095495	52095961	*	6	0.019324709	0.009313601	0.004391185	*ITGA1;PELO*
chr4	7282819	7283433	*	5	0.019329087	-0.015679343	0.002148375	*SORCS2*
chr22	20004356	20004881	*	9	0.019342766	-0.016421655	-0.004107403	*ARVCF;TANGO2*
chr16	85854840	85855299	*	4	0.019363379	-0.035906856	-0.015104477	*NA*
chr1	21929913	21930336	*	6	0.019380891	0.018778044	0.010099387	*RAP1GAP*
chr5	114514636	114515104	*	4	0.019380891	0.031940357	0.017625546	*TRIM36*
chr9	3526190	3526944	*	7	0.019387879	0.007018373	-0.001614871	*RFX3*
chr10	124133582	124134343	*	11	0.0193979	-0.022475452	-0.004824347	*PLEKHA1*

chr5	65891878	65892575	*	10	0.0193979	-0.030781413	-0.002835335	*MAST4*
chr1	216244716	216245074	*	3	0.019412619	0.025448987	-0.001149739	*USH2A*
chr17	908690	909300	*	3	0.019429985	0.026234352	0.000663521	*ABR*
chr13	112908450	112908629	*	3	0.019438032	-0.041104682	-0.012398541	*NA*
chr16	12010179	12010751	*	7	0.019442005	0.02913588	0.011088394	*GSPT1*
chr5	151138064	151138797	*	13	0.019453923	0.008259714	0.001589713	*ATOX1*
chr7	155250210	155251040	*	6	0.019479155	-0.010843202	-0.003728023	*EN2*
chr2	11885266	11885560	*	3	0.019480229	-0.027889515	-0.017287791	*LPIN1*
chr6	36808207	36808894	*	4	0.0194932	0.014374095	0.00781272	*CPNE5*
chr22	24828642	24829053	*	4	0.019504291	0.031182915	0.001574401	*ADORA2A;ADORA2A-AS1;SPECC1L*
chr1	161015733	161016165	*	9	0.019525207	-0.010222303	-0.004313962	*USF1*
chr6	32634276	32634362	*	4	0.019587166	-0.032026099	-0.016432133	*HLA-DQB1*
chr10	105127632	105128183	*	7	0.019595862	0.022655676	0.004824201	*TAF5*
chr5	57754512	57756187	*	16	0.019596975	-0.020245136	-0.00625867	*PLK2*
chr13	99737364	99737785	*	4	0.019596975	0.037029623	0.014524897	*DOCK9*
chr13	38172803	38173002	*	3	0.019621072	-0.031299603	-0.015998375	*POSTN*
chr17	77708928	77709291	*	4	0.019622182	0.020649883	0.007176177	*ENPP7*
chr12	133018862	133019135	*	3	0.019622182	0.013554854	0.012625436	*NA*
chr4	71337658	71337873	*	4	0.019649803	-0.027700346	-0.02320101	*MUC7*
chr3	45635690	45636386	*	10	0.019661859	0.015444614	0.004183083	*LIMD1*
chr11	126081116	126082534	*	17	0.019681826	-0.0149171	-0.001683272	*FAM118B;RPUSD4*
chr17	3796782	3797333	*	3	0.019689998	0.011931396	0.006998288	*CAMKK1*
chr2	16908218	16908766	*	3	0.019691758	0.020025771	0.006815213	*NA*
chr17	38599056	38599763	*	9	0.01971263	0.025999361	0.001122714	*IGFBP4*
chr20	2797023	2797721	*	4	0.019724978	0.024439525	0.011671566	*TMEM239*
chr1	59280290	59280952	*	5	0.019727626	0.025052862	0.014112217	*LINC01135*
chr2	30371369	30371990	*	3	0.019732839	0.034018548	0.005522425	*YPEL5*
chr19	2702863	2703115	*	9	0.019733487	-0.025315116	-0.005922626	*GNG7*
chr3	48263670	48264021	*	4	0.019752235	0.011993528	0.007928263	*CAMP*
chr2	23607015	23607254	*	3	0.019755876	0.025882658	0.015402585	*KLHL29*
chr2	101033546	101033799	*	7	0.019757883	0.005751915	0.000820763	*CHST10*
chr6	42981650	42982891	*	11	0.019764712	0.015410981	0.00111769	*KLHDC3;MEA1*
chr11	107992005	107992418	*	11	0.019773977	0.022765058	0.00176581	*ACAT1*
chr11	42275352	42275722	*	3	0.019779839	-0.017465329	-0.009751329	*LINC02740*
chr3	107393225	107393456	*	3	0.019779839	-0.017851266	-0.00486201	*BBX*
chr7	130020011	130020328	*	4	0.019787661	0.018917043	0.014238865	*CPA1*
chr2	242713790	242714091	*	3	0.019787661	0.029463777	0.021938722	*GAL3ST2*
chr12	54582519	54583403	*	13	0.019799475	-0.026894601	-0.008896417	*SMUG1*
chr6	166128575	166128682	*	3	0.01981472	0.017076464	0.007521531	*NA*
chr11	67372071	67373114	*	7	0.019829253	0.019961503	0.008143862	*NDUFV1*
chr5	41510116	41511150	*	11	0.01982942	0.041109401	-0.001509573	*PLCXD3*
chr5	496081	496476	*	4	0.019842442	-0.037029198	-0.028423259	*SLC9A3*
chr20	1924201	1924888	*	3	0.019860081	-0.02303479	-0.000847532	*NA*
chr6	147234727	147235737	*	5	0.01987674	-0.019503254	-0.010873229	*STXBP5-AS1*
chr16	88518659	88519557	*	6	0.019897418	-0.027343077	-0.005864082	*ZFPM1*
chr6	119471177	119471418	*	3	0.019902635	-0.014340025	-0.009647411	*FAM184A*
chr11	57103544	57103951	*	6	0.019909273	-0.028992821	-0.006213292	*P2RX3;SSRP1*
chr8	18067331	18067770	*	6	0.019921379	-0.01825331	-0.004157709	*NAT1*
chr16	88679600	88679919	*	4	0.019922911	-0.034559082	-0.011625713	*ZC3H18*
chr17	46683409	46684004	*	4	0.019934883	0.026229549	0.014155454	*HOXB-AS3;HOXB6*
chr5	169724656	169725100	*	6	0.019957618	-0.017735163	-0.006532635	*LCP2*
chr2	95718982	95720106	*	6	0.019969494	-0.019000589	-0.003517435	*MAL*
chr16	30041884	30042541	*	5	0.019986273	0.011349135	-0.002778293	*BOLA2;TLCD3B*
chr19	48833062	48833729	*	5	0.019996649	-0.015067633	-0.004544963	*EMP3*
chr1	44441199	44441770	*	3	0.019996649	-0.004736837	-0.003251694	*ATP6V0B;B4GALT2*
chr17	27332559	27333125	*	7	0.020040843	-0.026690054	-0.003541301	*SEZ6*
chr16	88513285	88513513	*	3	0.020048218	0.048036869	0.024109338	*NA*
chr11	68437843	68438384	*	4	0.020052896	0.017889097	0.008001838	*NA*
chr13	25182575	25183053	*	3	0.020052896	0.014572777	0.008030691	*NA*
chr7	32111062	32111453	*	6	0.020053063	-0.014857312	-0.003404118	*PDE1C*
chr6	170462469	170462947	*	3	0.02006462	-0.018183202	-0.013730026	*NA*
chr4	679526	681509	*	12	0.020089692	-0.021908283	0.002601495	*SLC49A3*
chr7	25900167	25900355	*	3	0.020120454	-0.014833831	-0.010349077	*NA*
chr6	170531180	170531435	*	4	0.020126114	0.02210752	-0.002226027	*NA*
chr1	51983496	51983979	*	5	0.020140994	-0.021854941	-0.007130344	*EPS15*
chr11	61722539	61722937	*	4	0.020140994	-0.011690539	-0.006501567	*BEST1*

chr3	42002230	42004036	*	15	0.020143296	-0.020911251	-0.003722231	*ULK4*
chr1	226855568	226856447	*	4	0.020148096	0.02997479	0.00784226	*ITPKB*
chr22	38840560	38841396	*	4	0.020158284	0.028055671	0.016052464	*KCNJ4*
chr18	904243	904523	*	5	0.020162406	-0.025170657	-0.008588492	*ADCYAP1*
chr11	119203814	119204876	*	4	0.020163681	-0.019134832	-0.007508908	*RNF26*
chr5	150004235	150004508	*	3	0.020166925	-0.034075116	-0.015777318	*SYNPO*
chr17	67410253	67410995	*	10	0.020176133	-0.051832866	-0.009857924	*MAP2K6*
chr15	75248086	75248768	*	3	0.020176715	0.038748671	0.015830289	*RPP25*
chr16	55850985	55851398	*	3	0.020185765	-0.030046892	-0.002205686	*CES1*
chr12	49628356	49628520	*	3	0.020193329	-0.019437965	-0.013059235	*TUBA1C*
chr5	43019773	43020627	*	9	0.020193842	-0.020276447	-0.004987362	*LOC648987*
chr12	54424964	54425634	*	4	0.020212392	0.028352073	0.008188151	*HOXC4;HOXC5;MIR615*
chr14	66974032	66975053	*	12	0.020213942	-0.021144217	-0.007323522	*GPHN*
chr16	30102457	30102919	*	6	0.02022278	0.02637984	0.011555998	*BOLA2;TBX6*
chr5	122180440	122181324	*	11	0.020227739	-0.009099518	-0.001266708	*SNX24*
chr2	145248570	145248942	*	3	0.020241585	-0.022815764	-0.002396926	*ZEB2*
chr11	45793222	45793643	*	3	0.020244963	-0.028034922	-0.013883784	*LINC02716*
chr6	167356786	167358034	*	3	0.020272301	-0.028320423	-0.014592085	*RNASET2*
chr1	173793075	173795086	*	21	0.020277307	0.023450956	-0.000515284	*CENPL;DARS2*
chr12	32908175	32908941	*	10	0.02028299	-0.00910854	0.000267997	*YARS2*
chr11	117186472	117187284	*	11	0.02029762	-0.014881576	-0.00272914	*BACE1*
chr1	101004934	101005509	*	5	0.02029762	0.017171457	0.001585851	*GPR88*
chr17	14201321	14201938	*	4	0.02029762	0.034921629	0.027269393	*HS3ST3B1*
chr17	46459980	46460509	*	3	0.020302414	-0.019665215	-0.001089838	*SKAP1*
chr1	24195347	24195659	*	4	0.020337307	-0.040157551	-0.026872658	*FUCA1*
chr6	30654410	30654845	*	16	0.020338755	0.011739183	-0.001151283	*PPP1R18*
chr5	1800542	1802207	*	12	0.020360629	0.020712881	-0.000223567	*MRPL36;NDUFS6*
chr17	8213195	8214081	*	7	0.020397189	0.025449468	0.014658062	*ARHGEF15*
chr6	32861863	32862547	*	23	0.020431251	-0.0163915	-0.001902844	*LOC100294145*
chr6	170123723	170124346	*	3	0.020449409	-0.021811235	-0.016369757	*PHF10*
chr1	1096717	1097270	*	6	0.020465331	0.021001763	0.010339246	*NA*
chr17	38657842	38658096	*	7	0.020470535	0.030851913	0.007514005	*TNS4*
chr4	37245254	37246054	*	6	0.020477169	-0.020738656	-0.009739672	*MIR4801;NWD2*
chr5	160278962	160279389	*	3	0.020485035	0.022185149	0.003316952	*ATP10B*
chr8	41386012	41386864	*	7	0.02050861	0.013386064	0.005336212	*GINS4*
chr15	45740221	45741008	*	9	0.02050963	-0.015930315	-0.005453649	*NA*
chr2	8714248	8714417	*	3	0.020579941	0.033607942	0.01485531	*NA*
chr8	118532775	118533254	*	7	0.020597656	-0.007003576	-0.003492362	*MED30*
chr3	55515541	55516596	*	7	0.020613909	-0.011065034	-0.004278285	*WNT5A*
chr22	46440394	46441127	*	8	0.020634793	-0.019603641	-0.00873668	*LINC00899*
chr7	100728527	100729412	*	9	0.020654257	-0.013441044	-0.005646315	*TRIM56*
chr14	105658949	105659807	*	3	0.020690817	0.028015185	0.013839387	*NA*
chr19	15947148	15947555	*	3	0.020696992	0.012740479	0.006089747	*NA*
chr5	140800761	140801482	*	5	0.020702244	-0.025701037	-0.006527491	*DHGA5;PCDHGA6;PCDHGA7;PCDHGA8;PCDHGA9;PCDHGB1;PCDHGB2;PCDHGB3;PCDHGB4;PCDHGB5;PCDHGB6;PCDHGB7;PCDHGB8P*
chr12	6930613	6931058	*	3	0.020702244	-0.005377271	-0.00440628	*GPR162*
chr10	119302540	119303225	*	5	0.020706735	0.016215339	-0.001174255	*EMX2;EMX2OS*
chr1	40505264	40506212	*	11	0.020716185	-0.013400766	-0.002853391	*CAP1*
chr3	36985516	36986154	*	4	0.020729869	0.004918618	0.000387128	*TRANK1*
chr4	15657657	15657883	*	3	0.020738227	0.034828139	0.017155798	*FBXL5*
chr22	45147515	45148385	*	7	0.02075174	-0.02275869	-0.008014638	*ARHGAP8;PRR5-ARHGAP8*
chr3	117716071	117717026	*	8	0.020761995	0.021007511	0.004119014	*NA*
chr17	38224456	38225121	*	5	0.020770982	0.027890139	0.006130452	*THRA*
chr7	32338950	32339497	*	10	0.020776922	-0.02486298	-0.013867672	*PDE1C*
chr7	11012871	11013816	*	14	0.02078819	-0.025865603	-0.005120602	*PHF14*
chr7	6476003	6476274	*	3	0.020810208	-0.0176018	-0.01504488	*DAGLB*
chr6	33395290	33396029	*	8	0.020812545	-0.025656454	-0.009831695	*SYNGAP1*
chr17	70710154	70711001	*	5	0.020813261	-0.023557451	0.000966808	*SLC39A11*
chr1	150121441	150122023	*	7	0.020831199	-0.009216209	-0.001878212	*PLEKHO1*
chr19	8934543	8934852	*	5	0.020831199	0.01118514	-0.000808564	*ZNF558*
chr12	132896338	132896939	*	8	0.020858757	0.028145437	0.009230143	*GALNT9*
chr21	43809237	43809848	*	8	0.020872305	-0.015904543	-0.009568003	*TMPRSS3*
chr5	68665600	68666075	*	9	0.020887339	-0.010571767	-0.001192711	*RAD17;TAF9*
chr12	57630834	57631687	*	5	0.020919062	-0.014290816	0.004274257	*NDUFA4L2*
chr20	61903495	61904700	*	13	0.02093291	-0.020160804	-0.003496723	*ARFGAP1*
chr9	139922545	139923402	*	6	0.020952405	0.005537312	3.15E-05	*ABCA2;C9orf139*
chr3	186647950	186648392	*	7	0.0209557	-0.018379405	-0.000986067	*ST6GAL1*

chr14	73137874	73138006	*	3	0.020966872	-0.044421494	-0.017274995	*DPF3*
chr14	105318279	105318494	*	3	0.020994779	-0.029639075	-0.02158895	*NA*
chr11	116643957	116644448	*	5	0.021010656	-0.009060667	-0.002692063	*BUD13*
chr7	15726166	15727441	*	10	0.021018111	-0.0187944	-0.009990715	*MEOX2*
chr7	158072687	158073079	*	3	0.021059779	0.020023348	0.015085934	*PTPRN2*
chr11	34535281	34536181	*	10	0.021065688	0.023844002	0.00316597	*ELF5*
chr10	63212815	63213510	*	9	0.021069664	-0.013535644	-0.007381664	*TMEM26*
chr13	46679079	46679242	*	3	0.0210749	-0.016443378	-0.0131667	*CPB2*
chr7	51539514	51539678	*	3	0.021094037	-0.036587316	-0.035403618	*NA*
chr2	27865068	27865335	*	4	0.021125372	0.013811303	0.001231444	*GPN1*
chr7	100611492	100612155	*	5	0.021139078	-0.047893936	-0.01694595	*MUC12*
chr19	56615895	56616780	*	6	0.021159513	0.016005185	-0.001599767	*ZNF787*
chr17	78753756	78754372	*	6	0.021172786	0.036623032	0.006414228	*RPTOR*
chr5	39074123	39075257	*	13	0.02119075	-0.010453991	-0.000724951	*RICTOR*
chr6	150311305	150311869	*	5	0.021219213	0.016762626	0.004313529	*NA*
chr10	33294358	33294889	*	5	0.021255357	-0.031732622	-0.011561118	*NA*
chr15	93606698	93607465	*	3	0.021259624	0.019777892	0.012215928	*RGMA*
chr12	85305677	85306323	*	4	0.021266099	-0.019037103	-0.005482774	*SLC6A15*
chr1	159868242	159868562	*	3	0.021272578	0.024573981	0.012488334	*CFAP45*
chr12	132943983	132944284	*	3	0.021284115	-0.040297571	-0.014182111	*NA*
chr12	102036140	102036563	*	5	0.02130042	0.024357746	0.011775075	*MYBPC1*
chr1	116710227	116710606	*	4	0.021315984	0.02641652	0.017810522	*NA*
chr8	1321333	1321883	*	5	0.02131899	-0.067702901	-0.041799678	*NA*
chr16	1131266	1131634	*	5	0.021321302	-0.042073623	-0.01040951	*SSTR5;SSTR5-AS1*
chr2	219773416	219774183	*	7	0.021348421	-0.02736604	-0.007124997	*NA*
chr13	29105493	29105629	*	3	0.021350487	-0.012937842	-0.008307113	*NA*
chr17	74965245	74965708	*	5	0.021358222	-0.008408359	-0.003597257	*NA*
chr19	24269525	24269919	*	4	0.021358688	0.026693599	0.004253621	*ZNF254*
chr8	102149430	102149991	*	5	0.021373193	-0.008443822	-0.004918613	*NA*
chr15	100016836	100017287	*	3	0.021390227	-0.015580746	-0.010459561	*NA*
chr5	71402641	71403502	*	9	0.021427761	0.020588795	-0.004314542	*MAP1B*
chr4	160024219	160024674	*	4	0.021428579	-0.012232691	-0.002620665	*C4orf45*
chr2	7057945	7058432	*	3	0.021436657	-0.006370356	0.000766992	*GRASLND;RNF144A*
chr7	98476174	98476815	*	5	0.021443321	-0.022452638	-0.009292206	*MIR3609;TRRAP*
chr6	99872940	99873747	*	11	0.021449865	-0.013758422	-0.001835401	*PNISR*
chr16	72993161	72993541	*	3	0.02145211	0.030274301	0.014266763	*ZFHX3*
chr7	27289195	27289829	*	4	0.021483865	-0.026552019	-0.009916222	*NA*
chr5	140345680	140346921	*	12	0.021487647	0.019752464	-0.004998546	*2;PCDHA13;PCDHA2;PCDHA3;PCDHA4;PCDHA5;PCDHA6;PCDHA7;PCDHA8;PCDHA9;PCDHAC1;PCDHAC2*
chr17	9862147	9862900	*	10	0.021487779	0.016505082	-0.00436741	*GAS7*
chr13	37247048	37247537	*	4	0.021487779	0.018177162	0.007776725	*SERTM1*
chr11	68923088	68923365	*	4	0.021490685	0.025175422	0.014468943	*NA*
chr3	184209995	184210194	*	3	0.021495585	-0.028384796	-0.013058773	*NA*
chr2	66660038	66661280	*	7	0.021510388	-0.009142912	-0.004496101	*MEIS1;MEIS1-AS3*
chr7	135194601	135195508	*	10	0.021518391	0.011212707	0.000766017	*CNOT4*
chr12	132268896	132270218	*	7	0.021557156	0.02426166	0.005683445	*SFSWAP*
chr3	52857612	52857931	*	4	0.021572078	0.016210397	0.003758798	*ITIH4*
chr20	60540388	60541082	*	5	0.021588547	-0.018441748	-0.013260903	*NA*
chr19	17326059	17326726	*	11	0.02159573	0.014935202	0.001124458	*USE1*
chr3	35706038	35706161	*	3	0.021607664	-0.030641198	-0.025740562	*ARPP21*
chr14	94547301	94547988	*	16	0.021623426	0.016717764	0.000881931	*DDX24;IFI27L1*
chr17	73083426	73084978	*	16	0.021623426	0.080662268	0.003726549	*SLC16A5*
chr11	71791545	71792206	*	12	0.021623426	-0.005424045	-0.001812236	*LRTOMT;NUMA1*
chr7	124430753	124431308	*	6	0.021623426	-0.033717812	-0.013748044	*C7orf77*
chr12	133303923	133304233	*	3	0.021632582	0.024837193	0.012499934	*ANKLE2*
chr17	48784765	48785602	*	8	0.021634164	-0.01015692	-0.003219696	*ANKRD40*
chr19	35644570	35644996	*	4	0.021636636	0.020462345	-0.001259402	*FXYD5;FXYD7*
chr4	69047717	69048001	*	3	0.021652847	0.024007702	0.020377061	*FTLP10*
chr16	57770390	57771195	*	6	0.021664754	0.01948435	-0.001864598	*KATNB1*
chr11	134025929	134026755	*	4	0.021682419	0.04479702	0.018041959	*NCAPD3*
chr16	88961784	88961936	*	3	0.021688961	0.021224766	0.019330356	*CBFA2T3*
chr17	6916889	6917600	*	6	0.021696254	0.027489126	0.008382731	*ALOX12-AS1;RNASEK-C17orf49*
chr6	5132887	5133630	*	7	0.02171693	0.04031265	0.006195151	*LYRM4*
chr17	56833855	56834361	*	3	0.021723496	-0.007058905	-0.004486493	*PPM1E*
chr14	36004683	36005164	*	3	0.021742176	-0.016477537	-0.005343971	*INSM2*
chr21	27750452	27750811	*	3	0.021745165	0.013691472	0.004486192	*NA*
chr14	37903900	37904126	*	3	0.021771268	0.021591423	-0.00277171	*MIPOL1*

chr8	127600783	127601070	*	3	0.021776906	0.022949784	0.011000156	*NA*
chr16	85589349	85590266	*	6	0.02179297	-0.005832076	-0.00174244	*NA*
chr16	50744993	50745102	*	3	0.021826991	-0.014884594	-0.001529495	*NOD2*
chr11	1095156	1095607	*	3	0.021881211	0.025158889	0.00612771	*MUC2*
chr6	30156892	30157507	*	4	0.021889219	-0.024669804	-0.001634181	*TRIM26*
chr17	79859809	79859999	*	8	0.02188951	0.014391884	0.007084686	*NPB*
chr18	57069690	57070086	*	3	0.021900825	-0.022806604	-0.013703521	*NA*
chr9	4679684	4679986	*	3	0.021911977	-0.008433513	-0.000407954	*CDC37L1*
chr13	45009596	45010051	*	4	0.021920793	-0.01673995	-0.00552093	*TSC22D1*
chr21	37852334	37852979	*	6	0.021932015	0.0178167	0.001529102	*CLDN14*
chr12	117100401	117100900	*	3	0.021953141	-0.02036536	-0.013838811	*NA*
chr11	77790964	77791508	*	8	0.021957975	0.036477689	0.006772189	*NDUFC2;NDUFC2-KCTD14*
chr16	1742021	1742458	*	3	0.021962893	0.015846274	0.005959791	*JPT2*
chr17	80048811	80049347	*	5	0.02198753	0.016259783	0.007154832	*FASN*
chr2	103334922	103335429	*	3	0.02199008	0.013633112	0.007736434	*MFSD9*
chr14	51562057	51562890	*	13	0.02199864	-0.008173006	-0.003562701	*TRIM9*
chr17	3474461	3474950	*	7	0.02199864	0.024220723	0.01511187	*TRPV1*
chr16	83964775	83965208	*	4	0.0220039	0.013505045	0.004617553	*NA*
chr2	68289972	68290812	*	8	0.022023722	-0.03384618	-0.014149236	*C1D*
chr2	241644166	241644240	*	3	0.022031377	-0.022127169	-0.015657463	*NA*
chr5	14108303	14108781	*	4	0.022044072	-0.025163078	-0.016688455	*NA*
chr3	79816794	79817592	*	12	0.022065778	0.024428926	-0.001775106	*ROBO1*
chr4	186049687	186050047	*	3	0.022065778	0.00651549	0.00180989	*NA*
chr20	50700982	50701649	*	4	0.022079847	0.020248318	0.012169627	*ZFP64*
chr19	1175076	1175444	*	3	0.022104392	0.021499479	0.015220969	*SBNO2*
chr17	26732120	26732836	*	6	0.022104664	0.018232145	0.007997092	*SLC46A1*
chr1	40942625	40942841	*	5	0.022112714	0.010565534	0.001082359	*ZFP69*
chr11	8703871	8704637	*	12	0.022119435	0.010008818	-0.00096365	*RPL27A;SNORA3A;SNORA3B*
chr1	76539604	76540438	*	10	0.022133186	-0.007431766	-0.002344207	*ST6GALNAC3*
chr6	31760345	31761076	*	11	0.022135017	-0.032331311	-0.010277671	*VARS1*
chr16	84538885	84539110	*	3	0.022135017	0.040707999	0.02991394	*MEAK7*
chr9	108006611	108006799	*	6	0.022154667	-0.035614739	-0.012112682	*SLC44A1*
chr16	85496172	85496275	*	3	0.022164239	-0.043633297	-0.021684644	*NA*
chr11	1781505	1781710	*	5	0.022166665	-0.012636692	-0.008639856	*CTSD;MOB2*
chr17	7210097	7210806	*	13	0.022169981	0.018328834	-0.0002927	*EIF5A*
chr14	61655040	61655919	*	5	0.022177172	-0.041511802	-0.019721183	*PRKCH*
chr5	60139708	60140432	*	6	0.022186598	-0.012879054	-0.001735869	*ELOVL7*
chr11	63949188	63949212	*	3	0.022191774	-0.015930068	-0.013294025	*STIP1*
chr5	40798287	40798812	*	13	0.022201437	-0.014251732	4.21E-05	*PRKAA1*
chr1	172501480	172502226	*	9	0.022219632	-0.030724032	-0.004072559	*SUCO*
chr12	125197501	125197730	*	3	0.022238391	-0.022602634	-0.008876519	*NA*
chr18	52989025	52989604	*	9	0.022255369	-0.014244651	-0.003717384	*TCF4*
chr20	3730680	3731334	*	4	0.022276049	0.026948454	0.024534794	*HSPA12B*
chr2	111556206	111556607	*	4	0.022279111	0.027971995	0.006957909	*ACOXL*
chr20	44992995	44993770	*	9	0.022345968	-0.010185597	-0.001770407	*SLC35C2*
chr18	42259977	42260794	*	9	0.022345968	0.016105797	0.000509894	*SETBP1*
chr1	67390377	67391144	*	14	0.022357711	-0.015955859	-0.003437989	*DNAI4;MIER1*
chr17	73257138	73258208	*	14	0.022381874	-0.007138046	-0.002126012	*GGA3;MRPS7*
chr6	112318395	112318507	*	3	0.022381874	-0.02275625	-0.012971936	*NA*
chr8	145728138	145728630	*	11	0.022388642	0.027257457	0.007802368	*GPT*
chr1	1244356	1245708	*	10	0.022388642	0.017984997	0.007023102	*ACAP3;PUSL1*
chr3	184529753	184530595	*	12	0.022411931	0.026159119	0.000829558	*VPS8*
chr2	7016969	7018153	*	7	0.022500105	0.028991647	-0.002113306	*RSAD2*
chr21	44830846	44831151	*	5	0.022506699	0.02701348	0.015456757	*NA*
chr10	48391701	48392199	*	3	0.022513872	0.015515943	0.011403085	*RBP3*
chr1	211688462	211689136	*	6	0.022516694	0.006804371	0.001117305	*NA*
chr1	34100737	34101256	*	4	0.022531993	0.020231582	0.008424213	*CSMD2*
chr1	39491882	39492570	*	9	0.022534009	-0.017217054	-0.004428669	*NDUFS5*
chr16	46962962	46963261	*	3	0.022534223	-0.019153442	-0.001778291	*GPT2*
chr22	50314650	50315243	*	6	0.022548203	0.0231822	0.006074406	*ALG12;CRELD2*
chr12	720995	721502	*	4	0.022556513	-0.032366459	-0.013282766	*NINJ2*
chr10	4891654	4892269	*	3	0.022561201	-0.017848523	-0.010901172	*NA*
chr8	29172607	29172998	*	4	0.022566198	-0.033780121	-0.018957359	*NA*
chr3	46924739	46924956	*	4	0.022573966	0.005719336	0.003130877	*PTH1R*
chr9	6924962	6925355	*	3	0.022587023	0.020623682	0.001275179	*KDM4C*
chr12	49488082	49488898	*	9	0.022605824	-0.023124575	-0.007539401	*DHH*

chr6	3625139	3625684	*	3	0.022640217	0.015704763	0.003350119	*NA*
chr13	53029355	53030145	*	10	0.022665373	0.007894052	-0.000235393	*CKAP2;VPS36*
chr4	519009	519713	*	4	0.022665373	-0.011315963	-0.000555796	*PIGG*
chr7	83823911	83824481	*	8	0.022667664	-0.014065672	-0.004600635	*SEMA3A*
chr6	30525976	30526487	*	3	0.022675629	-0.018261797	-0.013915526	*GNL1;PRR3*
chr13	113701892	113702436	*	4	0.022700831	0.023907592	0.010686664	*MCF2L*
chr19	45348917	45349685	*	9	0.022754336	-0.022649375	-0.007454979	*NECTIN2*
chr16	88922618	88923231	*	5	0.022758107	0.012131075	0.003835718	*GALNS;TRAPPC2L*
chr2	135675569	135675773	*	3	0.022760632	0.010657005	0.005412269	*CCNT2;CCNT2-AS1*
chr19	51051861	51052080	*	4	0.022774382	0.02946249	0.017571525	*LRRC4B*
chr2	120281999	120282685	*	8	0.022775441	0.019730844	0.009280859	*SCTR*
chr17	7233596	7234027	*	3	0.022789703	-0.037086355	-0.02054517	*NEURL4*
chr3	125075895	125076372	*	4	0.022816985	-0.014649341	-0.007541594	*ZNF148*
chr19	46476963	46478091	*	9	0.02281736	-0.028968795	-0.012710849	*NOVA2*
chr1	231004374	231004784	*	9	0.022821159	-0.01417232	-0.000333738	*C1orf198*
chr1	3435887	3436219	*	5	0.022844425	0.023680645	0.008096042	*MEGF6*
chr10	101088508	101089149	*	10	0.022852301	0.026293687	-0.00389852	*CNNM1*
chr1	228463260	228463734	*	3	0.022868748	-0.028981827	-0.008349463	*OBSCN*
chr10	23484025	23484326	*	4	0.022873303	0.01007842	0.001193069	*NA*
chr11	60719942	60720229	*	5	0.022896638	-0.016697038	-0.009328236	*SLC15A3*
chr7	4174482	4175200	*	6	0.022902683	0.019954585	0.010025714	*SDK1*
chr7	86273685	86274133	*	4	0.022955215	-0.011445406	-0.007019648	*GRM3*
chr11	1771555	1772006	*	6	0.022959888	0.029908887	0.014281426	*IFITM10;MOB2*
chr13	111164367	111164966	*	4	0.022973552	0.03391682	0.023314581	*COL4A2;COL4A2-AS1*
chr2	28550531	28551104	*	3	0.022975536	0.018600588	0.008447167	*BABAM2*
chr10	115510718	115511504	*	5	0.022995232	0.01315938	0.010672678	*PLEKHS1*
chr14	20773409	20774219	*	11	0.023035568	-0.021383953	-0.007396563	*TTC5*
chr10	831247	831947	*	4	0.023035568	-0.024291792	0.00707632	*NA*
chr10	743761	744747	*	4	0.0230477	0.024745629	0.016687811	*NA*
chr1	213562740	213562923	*	3	0.023073095	0.076288159	0.034254866	*NA*
chr12	4488749	4489221	*	7	0.023075629	-0.027384857	-0.009816448	*FGF23*
chr17	6023675	6024017	*	4	0.023075629	0.025562971	0.013347635	*WSCD1*
chr4	7940579	7940843	*	4	0.02310334	0.009637054	0.005573726	*AFAP1*
chr4	123929378	123929846	*	3	0.023128603	-0.019079803	-0.009470249	*SPATA5*
chr16	4465731	4466818	*	8	0.023128701	-0.010636744	-0.00440155	*CORO7;CORO7-PAM16*
chr10	45470023	45470562	*	4	0.023132108	0.017908824	-0.002088607	*RASSF4*
chr6	127796287	127796683	*	3	0.023133876	0.035555648	0.026542256	*SOGA3*
chr8	22421913	22422380	*	4	0.023135756	0.015812614	0.007689636	*SORBS3*
chr3	3840514	3841853	*	10	0.023144427	-0.011981434	-0.005448165	*LRRN1;SUMF1*
chr8	61545334	61545646	*	3	0.023149144	0.034034083	0.00236984	*NA*
chr8	144408506	144408955	*	5	0.023160963	-0.013938951	0.000124698	*TOP1MT*
chr16	87099957	87100606	*	5	0.023167907	0.027838153	-0.003091624	*NA*
chr10	101769626	101770384	*	4	0.023184586	-0.018717596	-0.013959502	*DNMBP*
chr19	38806145	38806874	*	11	0.023187911	-0.009835603	1.60E-05	*KCNK6;YIF1B*
chr6	39760607	39760833	*	5	0.023199131	-0.036496586	-0.011747522	*DAAM2*
chr9	131902135	131902471	*	4	0.023245726	-0.007040828	0.000450113	*PTPA*
chr1	109373104	109373498	*	3	0.023251236	-0.032754099	-0.020453801	*AKNAD1*
chr6	167544901	167545232	*	3	0.023255614	0.025103202	0.022068613	*CCR6*
chr9	16870464	16871106	*	7	0.023262132	-0.031478402	-0.013611933	*BNC2*
chr12	132380903	132381984	*	4	0.023268102	-0.02388973	-0.013387036	*ULK1*
chr2	85765766	85766572	*	9	0.023276555	-0.00755426	-0.000118481	*MAT2A;PARTICL*
chr22	50438356	50438769	*	4	0.023276555	0.026049363	0.014938898	*IL17REL*
chr13	114057070	114058019	*	5	0.023352647	0.013884462	0.009562224	*NA*
chr17	19185638	19186341	*	4	0.023353326	0.015496311	0.002719708	*EPN2*
chr18	43408770	43408983	*	3	0.023353326	0.020389876	0.002286433	*SIGLEC15*
chr10	3207620	3208485	*	3	0.023362866	-0.028862457	-0.01846208	*PITRM1*
chr8	130995990	130996306	*	4	0.023368743	0.021107532	0.017267699	*CYRIB*
chr14	68283005	68283326	*	4	0.023371429	-0.008920413	-0.005487345	*RAD51B;ZFYVE26*
chr6	158994197	158994509	*	3	0.023373682	0.027497515	0.01717291	*TMEM181*
chr14	39900677	39902004	*	15	0.023380091	-0.014513307	-0.00287216	*FBXO33*
chr19	55685292	55685577	*	3	0.023390808	-0.006746496	-0.005568788	*SYT5*
chr14	61119419	61120134	*	8	0.023392368	-0.010791886	-0.004389656	*SIX1*
chr1	182991254	182991847	*	4	0.023414108	0.01488291	0.003914066	*LAMC1*
chr13	114107554	114108433	*	10	0.023416204	-0.026632122	0.000850331	*ADPRHL1*
chr2	97426921	97427445	*	5	0.023427732	-0.010152483	-0.000531357	*CNNM4*
chr16	23568302	23569012	*	16	0.023444348	0.015563436	0.003124506	*EARS2;UBFD1*

chr1	30112256	30112807	*	3	0.023451923	-0.02593472	-0.006551786	*NA*
chr6	31781967	31782374	*	3	0.023483234	0.010406333	0.002322764	*HSPA1A;HSPA1L*
chr15	48009928	48010592	*	4	0.023495087	-0.010010132	-0.002339207	*SEMA6D*
chr1	180922629	180923340	*	5	0.023501591	-0.052517517	-0.024435756	*NA*
chr5	176169542	176170138	*	5	0.023503733	0.018420429	0.002791991	*NA*
chr3	49908706	49909109	*	3	0.023507431	0.028235403	0.015801167	*CAMKV*
chr16	30034426	30034487	*	3	0.023544342	0.017874791	0.012184657	*BOLA2;C16orf92*
chr1	241694967	241695616	*	4	0.023553198	0.046729577	0.010435694	*KMO*
chr16	73096842	73097364	*	6	0.023564662	-0.023999223	-0.005642974	*ZFHX3*
chr11	22850891	22851422	*	8	0.023579839	-0.035547889	-0.015848134	*SVIP*
chr20	60757239	60757919	*	6	0.023595696	0.020054046	0.002737464	*MTG2;SS18L1*
chr10	123286125	123286731	*	4	0.023596767	-0.040326741	-0.023266155	*FGFR2*
chr5	31193985	31194749	*	7	0.023624389	-0.013380821	-0.006889886	*CDH6*
chr6	30038254	30039025	*	15	0.023625717	0.022891789	0.008600461	*RNF39*
chr15	40453091	40453778	*	10	0.02363706	-0.014588059	-0.001990964	*BUB1B*
chr4	54975250	54975894	*	5	0.023644997	0.013997897	-0.001295401	*PDGFRA*
chr8	143808432	143809371	*	6	0.023660705	-0.016101431	-0.005070751	*LNCOC1;THEM6*
chr13	112075959	112076633	*	5	0.023668396	0.024453732	0.004069816	*NA*
chr15	101606267	101606897	*	3	0.023677953	0.017040244	0.007401494	*LRRK1*
chr2	28668002	28668518	*	3	0.023679454	0.02815081	-0.000709874	*NA*
chr6	52363697	52363783	*	3	0.023693551	0.017849944	0.011801131	*EFHC1;TRAM2*
chr19	16986822	16988083	*	4	0.023720709	0.038034741	0.022891785	*SIN3B*
chr10	119117341	119117529	*	3	0.02372844	-0.03159878	-0.018097974	*PDZD8*
chr19	3025347	3025829	*	5	0.023740711	0.02553882	0.016391741	*TLE2*
chr1	233248709	233249314	*	5	0.023772923	-0.013815822	-0.010857722	*PCNX2*
chr19	1408136	1408619	*	4	0.023822272	-0.010570725	-0.005328946	*DAZAP1*
chr16	90039076	90039755	*	12	0.023832557	-0.019119334	-0.001830024	*AFG3L1P;CENPBD1*
chr6	33969752	33970166	*	4	0.023835733	-0.017134764	-0.001916124	*MIR1275*
chr2	113967479	113967644	*	3	0.023848976	0.019259163	0.004016918	*NA*
chr11	58731154	58731433	*	3	0.023854873	0.043030153	0.023604255	*LOC283194*
chr1	170114243	170114782	*	3	0.023863702	0.053024819	0.008971284	*METTL11B*
chr14	21271115	21272204	*	9	0.023874319	0.018784698	0.000974603	*RNASE1*
chr2	178129548	178130284	*	9	0.02394275	-0.006824045	-0.002122758	*NFE2L2*
chr5	1952807	1953127	*	3	0.02395371	-0.044109212	-0.023357947	*NA*
chr1	9340416	9340583	*	3	0.023965557	-0.022094372	-0.013995119	*NA*
chr19	40871421	40872142	*	5	0.023974719	0.050043365	0.014528636	*PLD3*
chr9	131872542	131872920	*	3	0.023990814	-0.008331078	-0.004453139	*CRAT;PTPA*
chr15	23086598	23086979	*	9	0.024019267	-0.013281567	-0.005209354	*NIPA1*
chr3	81557365	81557839	*	3	0.02403408	-0.030213491	-0.021519421	*GBE1*
chr9	95526812	95527341	*	4	0.024037295	-0.006361723	0.000483837	*BICD2*
chr3	67022555	67023309	*	4	0.024049145	-0.035346873	-0.023924741	*NA*
chr13	46626275	46626866	*	6	0.02405804	-0.007592878	-0.003516584	*CPB2-AS1;ZC3H13*
chr13	36052768	36053408	*	4	0.024065489	0.012022204	0.005177437	*MIR548F5;NBEA*
chr2	231276363	231276668	*	4	0.024078936	-0.022023142	-0.012380011	*SP100*
chr19	13226942	13227421	*	6	0.024104561	-0.028837906	-0.002614246	*NACC1;TRMT1*
chr17	34122943	34123326	*	5	0.02412043	0.017621776	0.0088314	*MMP28*
chr5	19886482	19886875	*	3	0.024133103	-0.030270726	-0.012150146	*CDH18*
chr1	173833450	173834831	*	6	0.024157139	0.023269854	0.00939177	*GAS5;SNORD47;SNORD78;SNORD79;SNORD80;SNORD81;ZBTB37*
chr16	51796185	51796557	*	5	0.024157139	-0.014178671	-0.005584734	*NA*
chr1	173683037	173683938	*	6	0.024164113	0.019737867	-0.003412947	*KLHL20*
chr14	62550745	62550978	*	3	0.024178852	0.028925291	0.005649265	*SYT16*
chr5	159343468	159344281	*	6	0.024183248	-0.026476754	-0.003588278	*ADRA1B*
chr16	21222863	21223048	*	4	0.024183248	-0.015011082	-0.010747525	*ZP2*
chr12	277213	278159	*	6	0.02418362	0.023849918	-0.001227543	*IQSEC3*
chr10	29698152	29699043	*	9	0.024187951	-0.029063745	-0.018344406	*PTCHD3P1*
chr15	22955157	22955872	*	9	0.024191871	0.025961285	0.006584417	*CYFIP1*
chr15	94623896	94624459	*	4	0.024200971	0.021804996	0.016762132	*NA*
chr14	21550211	21550253	*	3	0.024201248	0.015603213	0.01214595	*ARHGEF40*
chr1	33772519	33772997	*	3	0.024225821	-0.020670465	-0.005892481	*A3GALT2*
chr9	139011703	139011834	*	5	0.02423821	0.014987886	0.010794332	*TMEM250*
chr4	149363450	149363912	*	9	0.024246818	-0.005650537	0.000182162	*NR3C2*
chr1	167683730	167683897	*	3	0.024253891	0.017819267	-0.001183194	*NA*
chr16	48844754	48844808	*	3	0.024259405	-0.014249576	-0.009343767	*NA*
chr7	107578250	107578588	*	3	0.024275497	-0.024234301	-0.010133745	*LAMB1*
chr5	22854640	22854718	*	4	0.024285351	-0.023081725	-0.011789089	*CDH12*
chr4	103421931	103422246	*	5	0.024288227	0.017424062	0.003947905	*NFKB1*

chr2	48010097	48010362	*	5	0.024299737	-0.012562638	-0.003601376	*MSH6*
chr1	38230769	38231980	*	11	0.024299842	0.018541976	0.001043877	*EPHA10*
chr7	132937623	132937996	*	10	0.024310624	-0.021558667	-0.007927604	*EXOC4*
chr15	75917792	75918631	*	12	0.024314551	-0.011871083	-0.002399407	*SNUPN*
chr1	248191944	248192376	*	4	0.024320769	0.022733143	0.005263851	*OR2L13*
chr9	71819016	71819562	*	3	0.024331829	-0.013778676	-0.008365666	*TJP2*
chr6	41888233	41889049	*	10	0.024332361	0.018866189	-0.000637106	*BYSL;MED20*
chr15	69323876	69324156	*	5	0.024340087	-0.019360817	-0.008597363	*MIR548H4;NOX5*
chr8	10907718	10907788	*	3	0.024354826	-0.026951159	-0.009453563	*XKR6*
chr14	101532170	101532938	*	9	0.024356962	0.015951262	0.006962594	*MEG9;MIR410;MIR656*
chr5	178413560	178414540	*	5	0.024362102	0.028867768	0.010985766	*GRM6*
chr8	49468296	49469040	*	9	0.024368229	0.028252922	-0.000565315	*NA*
chr1	55271284	55271927	*	9	0.024394204	0.026072684	0.013542193	*LEXM;TTC22*
chr11	372999	373615	*	5	0.024397671	0.008601026	0.006896504	*B4GALNT4*
chr16	89692360	89692715	*	4	0.024401674	-0.014520971	-0.006341301	*DPEP1*
chr17	72967968	72969066	*	10	0.024440547	-0.029338591	-0.007472728	*HID1*
chr7	101882646	101883319	*	4	0.024465389	0.016833226	-0.000364371	*CUX1*
chr4	169770092	169770406	*	3	0.024472101	-0.051377777	-0.04809308	*PALLD*
chr15	99193466	99194172	*	5	0.024481856	-0.015893046	-0.002720946	*IGF1R*
chr1	1250665	1251017	*	3	0.024494502	0.04056694	0.021183302	*INTS11*
chr14	55221338	55221487	*	3	0.024506886	-0.015255763	-0.003288359	*SAMD4A*
chr5	15928089	15928686	*	3	0.024513167	0.024844057	0.015241517	*FBXL7*
chr19	13113455	13113668	*	3	0.024522449	-0.017269042	0.002394347	*NFIX*
chr16	188651	189100	*	9	0.024532306	-0.019431342	-0.004833621	*NPRL3*
chr2	75787718	75789090	*	13	0.024567713	0.017150827	0.000749865	*EVA1A*
chr6	143832855	143833421	*	11	0.024573578	-0.012717236	-0.001164942	*FUCA2*
chr21	45502757	45502963	*	5	0.024601201	-0.023967764	-0.007122097	*TRAPPC10*
chr15	56757108	56757806	*	12	0.024632941	-0.013936528	-0.001271515	*MNS1*
chr1	159796231	159796434	*	3	0.024632941	-0.014990393	-0.01071243	*SLAMF8*
chr16	68572342	68572999	*	7	0.024674575	-0.020380943	-0.000516355	*ZFP90*
chr1	154974950	154975574	*	4	0.02467511	0.009771549	0.003699023	*ZBTB7B*
chr19	30865486	30866365	*	6	0.024715764	-0.022666196	-0.010751679	*ZNF536*
chr12	54982281	54982903	*	10	0.024727733	0.012686918	-0.001647419	*PPP1R1A*
chr15	80986274	80986965	*	3	0.024727733	-0.021538625	-0.011065656	*ABHD17C*
chr16	77755944	77756064	*	3	0.024757013	-0.019934743	-0.013841951	*NUDT7*
chr8	102179442	102179532	*	3	0.024757013	0.01846259	0.006770977	*NA*
chr8	146078935	146079781	*	8	0.024758052	0.023330266	-0.004283665	*COMMD5*
chr6	36515153	36515856	*	7	0.024772091	0.024870096	0.003661252	*STK38*
chr14	92413497	92414300	*	13	0.024789548	-0.022484811	-0.006445213	*FBLN5*
chr9	1050430	1050903	*	7	0.024799459	-0.013333181	-0.006270251	*DMRT2*
chr20	33291892	33292450	*	9	0.024828472	-0.022471021	-0.001544115	*TP53INP2*
chr14	23540584	23541335	*	6	0.024842341	0.015777153	-0.001532588	*ACIN1*
chr16	72127130	72127738	*	13	0.024843059	-0.016816219	-0.00235918	*DHX38;TXNL4B*
chr2	200320455	200321016	*	7	0.024877723	0.009737732	-0.000575352	*SATB2*
chr7	73008007	73008295	*	3	0.02488519	0.017602017	0.007933533	*MLXIPL*
chr15	74495109	74495657	*	9	0.024886326	0.024516791	0.012774345	*STRA6*
chr7	145813008	145813494	*	10	0.024889397	-0.045306852	-0.010128303	*CNTNAP2*
chr19	40005351	40006292	*	13	0.024911602	-0.016577915	-0.005501509	*SELENOV*
chr17	39507615	39508274	*	4	0.024911602	-0.028152687	-0.010586159	*KRT33A*
chr8	134202353	134202467	*	4	0.024911602	0.020165945	0.001172466	*CCN4*
chr1	11821740	11822362	*	11	0.024933024	0.022412722	-7.26E-05	*NA*
chr4	176922556	176923081	*	5	0.024936514	-0.008263226	-3.85E-05	*GPM6A*
chr10	132912784	132913255	*	5	0.024940918	0.03782205	0.004950551	*TCERG1L*
chr1	50574837	50575374	*	5	0.024943011	-0.017322337	-0.006178289	*ELAVL4*
chr17	52978014	52978583	*	7	0.024955115	-0.016833589	-0.001627217	*TOM1L1*
chr6	137241923	137242316	*	4	0.024955115	0.023974425	0.006653464	*SLC35D3*
chr15	25201020	25201732	*	4	0.024960028	-0.013025604	-0.005693289	*SNRPN;SNURF*
chr10	111966029	111966886	*	4	0.024972278	-0.04594774	-0.026670442	*MXI1*
chr4	47487081	47487587	*	9	0.024982956	-0.030108128	-0.008909825	*ATP10D*
chr17	37754160	37754424	*	4	0.024991171	-0.016710105	-0.004281261	*NA*
chr10	131577638	131577741	*	3	0.024991171	0.022587564	0.013052818	*NA*
chr17	56408688	56409011	*	5	0.025005343	-0.018912318	-0.008275355	*MIR142;TSPOAP1;TSPOAP1-AS1*
chr5	122434472	122435081	*	5	0.025007316	0.025998467	0.00778337	*PRDM6*
chr19	17608110	17608394	*	3	0.025029831	0.033184835	0.028366925	*SLC27A1*
chr2	197792289	197792853	*	4	0.025038229	0.016739893	0.007401751	*PGAP1*
chr19	9965044	9965490	*	6	0.025047148	0.025131601	0.00791512	*OLFM2*

chr1	52456918	52457247	*	3	0.025082882	0.023163527	0.011444023	*RAB3B*
chr8	96085385	96085994	*	4	0.025097994	-0.038378871	-0.024207136	*MIR3150B;NDUFAF6*
chr19	35801014	35801215	*	3	0.025110505	0.03280158	0.023418461	*MAG*
chr20	44718510	44718948	*	10	0.025121123	-0.011696411	-0.003000267	*NCOA5*
chr6	33216585	33217303	*	20	0.025123725	0.020235244	0.001951646	*HCG25*
chr3	195638330	195638936	*	3	0.025136766	0.01686719	0.010950212	*TNK2*
chr6	168066888	168067665	*	3	0.025145542	0.031162048	0.014596939	*NA*
chr1	232940329	232940591	*	6	0.02514705	-0.019422222	-0.001808698	*MAP10*
chr2	136742919	136743460	*	8	0.025156527	-0.010763039	-0.000961733	*DARS1*
chr17	71419646	71420454	*	7	0.025169932	0.011963974	0.001841447	*SDK2*
chr7	100861711	100862300	*	3	0.025195065	-0.016358288	-0.009996633	*PLOD3;ZNHIT1*
chr11	133837265	133838053	*	4	0.025216649	-0.033165618	-0.017550313	*NA*
chr4	184825842	184826314	*	5	0.025217423	0.018181619	0.002320703	*STOX2*
chr2	1895961	1896720	*	3	0.02522228	0.013041819	0.006076357	*MYT1L*
chr17	16229787	16230051	*	3	0.025227247	-0.012460536	-0.006323037	*NA*
chr6	30737007	30737270	*	4	0.025240537	-0.018462671	-0.007460381	*NA*
chr5	131563015	131563953	*	13	0.025278233	-0.018186217	-0.000353116	*P4HA2*
chr8	139734443	139734844	*	4	0.02529673	0.029237917	0.018024075	*COL22A1*
chr1	70686743	70686970	*	3	0.02530547	0.011788309	0.002796617	*SRSF11*
chr16	24621214	24621578	*	4	0.025321504	-0.013827499	-0.007095153	*NA*
chr9	36572411	36572905	*	10	0.025337905	0.014465437	0.000425482	*MELK*
chr19	19572954	19573143	*	3	0.025348815	-0.01829077	-0.013602342	*GATAD2A*
chr8	144358566	144359074	*	3	0.025360093	-0.063059387	-0.040757177	*GLI4;ZFP41*
chr8	56792171	56792545	*	6	0.025386816	0.014564309	0.001531371	*LYN*
chr8	2091201	2091425	*	3	0.02538793	0.017784014	7.79E-05	*MYOM2*
chr5	5140372	5140646	*	3	0.025390365	-0.025111887	-0.01628176	*ADAMTS16*
chr10	49719302	49719922	*	4	0.025399177	0.011759515	0.000511951	*ARHGAP22*
chr11	65029061	65030075	*	13	0.025427012	-0.007975267	-0.000156772	*POLA2*
chr11	70455278	70455309	*	3	0.025436463	-0.024966313	-0.022009565	*SHANK2*
chr6	158402912	158403038	*	3	0.025460336	-0.008153169	-0.003639287	*SYNJ2*
chr12	75723785	75724205	*	12	0.02550852	-0.007599444	-0.003381148	*CAPS2;GLIPR1L1*
chr15	41836038	41836937	*	11	0.025563257	-0.020577128	-0.003475972	*RPAP1*
chr20	33460767	33461536	*	7	0.025565649	0.014755576	0.001071164	*ACSS2;GGT7*
chr22	18985632	18985691	*	3	0.025580392	0.038719635	0.027931054	*DGCR5*
chr2	70995440	70996021	*	9	0.025600399	-0.044533432	-0.010566277	*ADD2*
chr20	657283	657694	*	4	0.025600399	-0.006374721	-0.00314698	*SCRT2;SRXN1*
chr7	2738728	2738961	*	3	0.025628029	-0.008096122	-0.00502115	*AMZ1*
chr11	101980355	101980807	*	4	0.025644887	-0.014328125	-0.002497409	*YAP1*
chr1	226734755	226735384	*	3	0.025644887	0.027225521	0.009254811	*STUM*
chr17	782910	783451	*	4	0.025660049	-0.034554277	-0.013755538	*NXN*
chr17	79668281	79669156	*	5	0.025670324	0.019937687	0.008605858	*HGS;MRPL12;SLC25A10*
chr18	33767393	33767797	*	5	0.025678932	-0.014572275	-0.007899912	*MOCOS*
chr5	79331477	79331850	*	4	0.025698443	0.022865588	0.011114264	*THBS4*
chr7	40174653	40175036	*	3	0.025711656	0.013230155	0.006069509	*MPLKIP;SUGCT*
chr5	1269992	1270598	*	5	0.025717569	0.029378928	0.009173986	*TERT*
chr2	231084523	231084965	*	6	0.025718482	-0.019651241	-0.008198637	*SP110*
chr19	36980659	36980975	*	11	0.02573449	-0.015306605	-0.001939222	*LOC728752;ZNF566*
chr8	21881852	21882587	*	8	0.025735631	0.007936846	0.002912551	*NPM2*
chr1	15478280	15479095	*	10	0.025753474	-0.018960765	-3.28E-05	*TMEM51;TMEM51-AS1*
chr7	96653183	96653747	*	3	0.025773857	0.013876997	-0.001089756	*DLX5*
chr5	67521596	67521733	*	3	0.025783118	0.020361895	0.008838668	*PIK3R1*
chr6	30523072	30524468	*	33	0.025784482	-0.024756424	-0.004556293	*GNL1;PRR3*
chr16	15982174	15983042	*	10	0.025792532	0.018846581	0.000391675	*CEP20*
chr12	89744150	89744701	*	8	0.025792532	-0.020425111	-0.00956793	*DUSP6*
chr13	36787885	36788462	*	3	0.025792532	0.023161444	0.011043061	*CCDC169-SOHLH2;SOHLH2*
chr19	36346914	36347626	*	4	0.025792723	-0.009805396	-0.00251473	*KIRREL2;NPHS1*
chr16	89686885	89687052	*	5	0.025796024	0.036704255	0.01265787	*DPEP1*
chr10	118886758	118886914	*	3	0.02581048	0.016311195	0.012694085	*SHTN1*
chr19	2038527	2038600	*	3	0.02581048	0.022424479	0.020078266	*MKNK2*
chr2	106226918	106227214	*	4	0.025822024	-0.020475879	-0.001492392	*LOC285000*
chr16	68278423	68278969	*	5	0.025829513	-0.013241507	-0.003012436	*PLA2G15*
chr7	18535499	18535899	*	4	0.02585145	-0.008622491	-0.003881623	*HDAC9*
chr16	31159558	31159920	*	5	0.025873145	0.032871097	0.024530984	*PRSS36*
chr19	1083007	1083429	*	4	0.025882189	0.024418207	0.01071721	*ARHGAP45*
chr11	102401438	102402091	*	4	0.025896815	-0.015013855	-0.000686757	*MMP7*
chr10	1285332	1285535	*	3	0.025920301	0.012717308	0.002938243	*ADARB2*

chr4	154709756	154710224	*	4	0.025989848	0.005248862	0.000795373		*SFRP2*
chr4	185570396	185570979	*	10	0.02603161	-0.024222017	-0.012454018		*CASP3;PRIMPOL*
chr20	58179996	58180616	*	4	0.026031712	-0.018846572	-0.003534909		*PHACTR3*
chr12	131568718	131569289	*	4	0.026041191	0.025804989	0.007917313		*ADGRD1*
chr16	48419131	48419812	*	5	0.026043168	-0.013949308	-0.005535146		*MIR548AE2;SIAH1*
chr8	145649036	145649678	*	5	0.026067638	0.02019504	0.013776397		*VPS28*
chr1	182360683	182361507	*	11	0.026068688	-0.018728156	-0.004987561		*GLUL*
chr4	53588360	53588850	*	5	0.026076586	-0.004204611	-0.002549488		*NA*
chr15	68570336	68570942	*	5	0.026088351	-0.004364426	-0.000417786		*FEM1B*
chr12	10183167	10183850	*	7	0.026114426	-0.025131691	-0.014865204		*CLEC9A*
chr8	28196872	28196932	*	3	0.026136116	-0.01269803	-0.010709763		*PNOC*
chr17	74733438	74734280	*	14	0.026170008	-0.00822292	-0.001353888		*MFSD11;MIR636;SRSF2*
chr2	63271927	63272334	*	4	0.026211473	0.028929387	0.014489676		*EHBP1;LOC100132215;OTX1*
chr1	1115575	1115920	*	4	0.026212113	0.01612516	0.010443677		*TTLL10*
chr2	241508090	241508108	*	3	0.026226195	-0.02897753	-0.022036917		*RNPEPL1*
chr17	35289351	35289677	*	4	0.026226732	-0.012927644	-0.004345511		*NA*
chr22	24032426	24032999	*	5	0.02624579	0.013077847	0.008259086		*GUSBP11;RGL4*
chr6	128841559	128842318	*	13	0.026274293	-0.017282825	-0.001470883		*PTPRK*
chr8	10987869	10988117	*	4	0.026351129	-0.013950697	0.004069803		*XKR6*
chr2	171627721	171628203	*	4	0.026355848	-0.020170287	-0.008160153		*NA*
chr19	5237246	5237619	*	4	0.026357332	0.02025144	0.010795233		*PTPRS*
chr14	96566573	96566671	*	3	0.026357332	0.012925155	0.007166994		*NA*
chr11	132813362	132814117	*	7	0.026370532	-0.024569333	-0.009080603		*OPCML*
chr11	71189385	71189582	*	3	0.026437153	-0.021112881	-0.014006844		*NADSYN1*
chr6	30613282	30613506	*	3	0.02644018	0.015533539	0.008499061		*ATAT1;C6orf136*
chr10	50341989	50342347	*	4	0.026448213	0.025148268	0.007788431		*FAM170B;FAM170B-AS1*
chr5	59481426	59481615	*	3	0.026456055	-0.029867731	-0.010552711		*PDE4D*
chr6	158437741	158438419	*	10	0.026462159	0.019502477	0.007034899		*SYNJ2*
chr6	42110188	42110867	*	8	0.026472382	-0.021358816	-0.008441319		*C6orf132*
chr9	140444429	140445006	*	5	0.02652657	-0.006717344	-0.005792238		*MRPL41;PNPLA7*
chr12	106495651	106496122	*	3	0.026536772	-0.025583764	-0.009345546		*NUAK1*
chr12	32552765	32553273	*	5	0.026548463	-0.016157697	-0.003269305		*NA*
chr4	1294432	1295078	*	8	0.02656048	-0.015800579	-0.009713442		*MAEA*
chr1	29138919	29139121	*	4	0.026606119	-0.020867678	-0.010684691		*OPRD1*
chr22	25960673	25961523	*	5	0.026607724	-0.033582876	-0.012285211		*GRK3*
chr16	11348611	11349023	*	5	0.026615323	-0.019501756	-0.003928521		*RMI2;SOCS1*
chr15	94840793	94841112	*	4	0.026635492	-0.018910781	-0.013303779		*MCTP2*
chr3	101546589	101547296	*	8	0.026638687	-0.013092727	-0.003965935		*NFKBIZ;NXPE3*
chr19	47524222	47524483	*	3	0.026638687	0.005774856	0.002385419		*NPAS1*
chr22	50739843	50740298	*	4	0.026640159	0.034904991	0.007803084		*PLXNB2*
chr17	66985719	66986095	*	3	0.026644113	-0.020897306	-0.009259785		*ABCA9*
chr1	24393149	24393584	*	4	0.026648569	0.024118434	0.004441647		*MYOM3*
chr19	44616907	44617506	*	10	0.026657051	-0.009739627	-0.003529649		*LOC100379224;ZNF225*
chr19	5829172	5829415	*	3	0.026660227	0.033119882	0.014746095		*NA*
chr11	2159853	2160129	*	3	0.026685403	-0.022092829	-0.007647097		*IGF2;IGF2-AS;INS-IGF2;MIR483*
chr19	14260587	14260651	*	3	0.026710897	-0.011069193	-0.007709552		*ADGRL1;LOC100507373*
chr20	30639305	30640164	*	13	0.026718472	-0.018513779	-0.003926139		*HCK*
chr15	99789622	99790022	*	7	0.026744015	-0.025739608	-0.015078565		*LRRC28;TTC23*
chr5	150585032	150585210	*	4	0.026768602	0.020957315	0.004811991		*CCDC69*
chr2	60781129	60781640	*	5	0.026775825	-0.018642681	-0.008543933		*BCL11A*
chr11	76902992	76903183	*	3	0.026779211	-0.035014812	-0.033618581		*MYO7A*
chr12	125398559	125399214	*	7	0.026789559	-0.016185413	-0.003422395		*MIR5188;UBC*
chr1	149889330	149889544	*	3	0.026796885	-0.026349425	-0.010167697		*SV2A*
chr3	40547540	40547855	*	5	0.026832981	0.013754923	0.003472028		*ZNF620*
chr14	100610407	100610667	*	3	0.026845681	0.019894719	0.015748547		*EVL*
chr17	41738893	41739326	*	5	0.026872684	0.050350495	0.018222959		*MEOX1*
chr17	76464687	76464784	*	3	0.026872684	0.022516627	0.016963212		*DNAH17*
chr12	65048810	65049149	*	4	0.026891509	-0.027989958	-0.013678446		*RASSF3*
chr2	215275757	215276156	*	4	0.026894565	-0.030084109	-0.015109885		*VWC2L*
chr14	90847024	90847382	*	3	0.026895108	0.022505032	0.01144166		*NA*
chr4	96470887	96471143	*	4	0.026896723	-0.013437111	-0.001935798		*UNC5C*
chr16	3931286	3931885	*	4	0.026896723	0.03410584	0.016123103		*CREBBP*
chr1	28157569	28157761	*	4	0.026904809	-0.005515317	-0.003727893		*PPP1R8;SCARNA1*
chr10	131365266	131365343	*	3	0.026906831	-0.016868756	-0.00861347		*MGMT*
chr11	66033194	66033456	*	4	0.026911399	0.013095351	0.00525014		*KLC2;RAB1B*
chr3	18480242	18480706	*	6	0.026913378	-0.017963529	-0.008783414		*SATB1*

chr7	5518071	5518918	*	5	0.026931266	-0.015197123	-0.00661515	*FBXL18*
chr4	7665770	7666259	*	6	0.026946036	0.021077344	0.008544666	*SORCS2*
chr4	25915329	25916067	*	7	0.02695353	-0.014948757	-0.001650402	*SMIM20*
chr19	11215861	11216364	*	5	0.026954299	0.030582628	0.01343758	*LDLR*
chr12	69202638	69202842	*	7	0.027005058	-0.017439357	-0.001403803	*LOC100130075;MDM2*
chr12	1772230	1773108	*	4	0.027007191	0.033546382	0.02296519	*MIR3649*
chr10	128593624	128594144	*	8	0.027012022	-0.021700115	-0.011043993	*DOCK1*
chr20	2451474	2452132	*	9	0.02702842	0.03851361	0.002954366	*SNRPB*
chr18	67955537	67955968	*	6	0.02702842	-0.014753915	-0.007404371	*SOCS6*
chr5	14871469	14872207	*	9	0.027064163	-0.018846413	-0.002671564	*ANKH*
chr16	3161669	3162480	*	9	0.0270702	0.034338554	0.014277761	*ZNF205;ZNF205-AS1*
chr1	241519213	241519756	*	3	0.027082009	0.017420941	0.004231743	*RGS7*
chr16	769848	770896	*	7	0.027103885	0.019591027	-0.001387583	*ANTKMT*
chr5	17444061	17444401	*	5	0.027125702	-0.02324667	-0.014670833	*NA*
chr3	50541011	50541552	*	8	0.027165823	-0.023864448	-0.00527199	*CACNA2D2*
chr3	125094085	125094680	*	7	0.027176952	0.003720134	0.001318986	*ZNF148*
chr3	27756039	27756421	*	5	0.027202894	0.038679496	0.014674123	*NA*
chr1	43637799	43638475	*	18	0.02720407	-0.006387962	-0.00089307	*CFAP57;EBNA1BP2*
chr1	154744758	154745202	*	4	0.027204495	0.057424084	0.013482467	*KCNN3*
chr12	133015383	133015982	*	4	0.027208901	0.011255465	0.001902523	*NA*
chr6	43970084	43970475	*	3	0.027260095	-0.009280665	-0.001873685	*C6orf223*
chr11	66444738	66444806	*	3	0.027280268	-0.010269193	-0.003755307	*RBM4B*
chr11	62340381	62340981	*	3	0.027280605	0.015475887	0.00446674	*EEF1G*
chr9	129885291	129885938	*	3	0.027296473	0.030971603	0.003943705	*ANGPTL2;RALGPS1*
chr3	146261991	146262700	*	10	0.027313932	-0.023493314	-0.008914698	*PLSCR1*
chr7	2020495	2020583	*	4	0.02733579	0.017240951	0.010353182	*MAD1L1*
chr1	21059114	21059745	*	11	0.027335952	-0.014342888	0.001936912	*SH2D5*
chr6	43395289	43396116	*	6	0.027338626	0.014648544	0.002067858	*ABCC10*
chr7	50468399	50469042	*	4	0.027344611	0.010612443	0.00284731	*IKZF1*
chr10	15255055	15255429	*	4	0.027344819	0.028643747	0.017950242	*FAM171A1*
chr13	26042382	26043165	*	6	0.027380172	-0.01913996	-0.007151122	*ATP8A2*
chr9	115095793	115096032	*	5	0.027398161	-0.026319395	-0.012424127	*MIR3134;PTBP3*
chr16	3067357	3068128	*	6	0.027400658	0.018666343	0.009845262	*CLDN6;TNFRSF12A*
chr15	77364519	77364890	*	5	0.027400658	0.015837768	-0.001351913	*TSPAN3*
chr11	63783359	63784126	*	3	0.027429263	-0.024522048	-0.004808094	*MACROD1*
chr8	96145073	96145483	*	3	0.027440799	0.025188738	0.009950586	*PLEKHF2*
chr15	83477608	83477965	*	6	0.027453628	0.035616173	0.017411385	*FSD2;WHAMM*
chr18	6414974	6415922	*	9	0.027473037	-0.020381365	-0.005358693	*L3MBTL4*
chr8	97273534	97274070	*	10	0.027526171	-0.008684956	-0.00159386	*MTERF3;PTDSS1*
chr17	42785078	42785378	*	3	0.027526547	0.024321601	0.006637557	*DBF4B*
chr18	56338220	56338661	*	11	0.027531763	0.00998408	-0.001024696	*MALT1*
chr15	67390734	67390982	*	4	0.027531763	-0.008716118	-0.005438939	*SMAD3*
chr1	1509833	1510598	*	8	0.027549514	0.005214081	0.000190752	*SSU72*
chr4	146099474	146099544	*	3	0.027576894	0.027518277	0.008174641	*OTUD4*
chr11	117299121	117299471	*	3	0.027582933	0.018415629	0.011348795	*DSCAML1*
chr8	42994955	42995566	*	5	0.027596516	-0.015260919	-0.00453431	*HGSNAT*
chr7	27282112	27282756	*	10	0.027606381	0.018800716	0.002875867	*EVX1*
chr19	51017855	51018414	*	7	0.027631511	-0.023619189	-0.009103332	*ASPDH;JOSD2*
chr8	135812896	135813202	*	3	0.027687982	-0.039091657	-0.014649614	*MIR30B*
chr18	43915649	43915901	*	3	0.027692732	0.014272206	0.010421789	*RNF165*
chr14	67982456	67983363	*	3	0.027711056	-0.020563575	0.000949689	*TMEM229B*
chr22	29168135	29168792	*	12	0.027712106	-0.021211336	-0.000688802	*CCDC117*
chr19	46271962	46272079	*	3	0.027731655	-0.019955445	-0.013302543	*SIX5*
chr8	80802929	80803365	*	5	0.027736516	-0.009834599	-0.003205515	*NA*
chr3	33481942	33482718	*	10	0.027774692	-0.020052909	-0.005389272	*UBP1*
chr4	2820428	2820479	*	3	0.027785588	-0.0237495	-0.020958187	*SH3BP2*
chr14	69951422	69952104	*	6	0.027789722	0.027016821	0.001063608	*PLEKHD1*
chr6	75913786	75914333	*	5	0.027796497	-0.021865471	-0.010998053	*COL12A1*
chr16	86570343	86570503	*	3	0.027831188	0.019015576	0.009551389	*MTHFSD*
chr10	3374141	3374366	*	4	0.027867867	0.017706322	0.008680846	*NA*
chr9	140081916	140082149	*	3	0.027872631	-0.01925443	-0.005286571	*ANAPC2;SSNA1*
chr17	77127426	77127605	*	3	0.027872631	0.023149293	0.017230184	*RBFOX3*
chr8	11567691	11567896	*	3	0.027894535	-0.042067338	-0.016450419	*GATA4*
chr10	105253001	105253342	*	4	0.027916985	0.020062188	0.009453206	*NEURL1*
chr12	54653065	54653427	*	4	0.027922862	0.029867685	0.004364873	*CBX5*
chr2	113402615	113404078	*	11	0.02794329	0.013367346	0.000368061	*FLJ42351;SLC20A1*

chr2	175190675	175191374	*	8	0.027950353	-0.016283886	-0.006880979	*LINC01305*
chr10	114206433	114207009	*	13	0.027956743	-0.019776256	-0.001897324	*VTI1A;ZDHHC6*
chr3	46250357	46250809	*	3	0.027970216	-0.027125546	-0.017373629	*CCR1;CCR3*
chr19	8840459	8841140	*	6	0.027974565	-0.021321392	-0.012111324	*OR2Z1*
chr16	57505058	57506038	*	3	0.027975331	0.022943401	0.000546799	*DOK4;POLR2C*
chr14	104387210	104387923	*	7	0.027978035	-0.003048637	-0.000870262	*ATP5MPL*
chr20	40619057	40619244	*	3	0.027979324	0.039352939	0.02192647	*NA*
chr1	246958862	246959476	*	5	0.02800432	-0.017348121	0.000318908	*NA*
chr6	117585741	117586817	*	10	0.028009493	-0.008869309	-0.001421516	*VGLL2*
chr2	61371880	61372316	*	8	0.028010286	-0.020474328	-0.010340156	*C2orf74;LOC339803*
chr7	116312265	116312904	*	6	0.028011589	0.015535965	0.001731784	*MET*
chr6	41703332	41704003	*	9	0.028016435	-0.017118751	0.002925844	*TFEB*
chr1	47082691	47082869	*	6	0.028017725	-0.031094859	-0.007816433	*MKNK1;MOB3C*
chr14	90527378	90527606	*	3	0.02803928	-0.02227698	-0.005702776	*KCNK13*
chr17	77900680	77901224	*	4	0.028117334	0.021680584	0.010962197	*NA*
chr1	160254759	160255036	*	5	0.028118748	-0.043590066	-0.013081983	*DCAF8;PEX19*
chr7	2632080	2632689	*	6	0.028165271	0.022799074	0.011634954	*IQCE*
chr21	31130263	31130566	*	3	0.028189212	0.035729861	0.021369535	*GRIK1;GRIK1-AS1*
chr15	50473854	50474388	*	9	0.028191581	0.031725506	0.00853738	*SLC27A2*
chr1	155099264	155099843	*	6	0.028198991	0.007497728	0.000788102	*EFNA1*
chr13	80917220	80917432	*	3	0.028198991	-0.017546091	-0.009404361	*SPRY2*
chr19	1510494	1510692	*	4	0.028208256	0.03098925	0.023262851	*ADAMTSL5*
chr6	31743769	31744339	*	10	0.02823108	0.01839612	0.005141487	*VWA7*
chr10	18428843	18429760	*	10	0.028256074	-0.02380766	-0.00614826	*CACNB2*
chr19	1789060	1789618	*	4	0.028256074	-0.024029362	-0.015842675	*ATP8B3*
chr14	70186289	70186639	*	4	0.028279059	-0.019471243	-0.011964006	*NA*
chr17	77194008	77194259	*	4	0.028293885	0.024358751	0.012007339	*RBFOX3*
chr15	82379655	82379947	*	3	0.028296291	0.019963768	0.009022839	*NA*
chr15	52043657	52044314	*	7	0.02829914	0.017443284	0.00277478	*LYSMD2;TMOD2*
chr16	3225044	3225686	*	7	0.02830634	-0.021365698	-0.009946532	*NA*
chr2	134169656	134169851	*	3	0.02831005	-0.027559599	-0.009421863	*NCKAP5*
chr18	8784363	8784658	*	3	0.028324331	0.023331362	0.015719445	*MTCL1*
chr1	160231874	160232248	*	6	0.028333123	-0.008741907	-0.001099271	*DCAF8*
chr4	81951446	81952330	*	8	0.028338593	-0.015615629	-0.004593513	*BMP3*
chr11	87908558	87909062	*	8	0.02834054	-0.016783279	-0.004944887	*RAB38*
chr21	31311797	31312154	*	8	0.028346453	-0.007661835	-0.004859584	*GRIK1*
chr15	67813698	67814090	*	5	0.028346453	-0.010337238	-0.001149543	*C15orf61;IQCH-AS1*
chr18	74691097	74691423	*	4	0.028351725	-0.023286266	0.002024155	*MBP*
chr17	38520139	38520653	*	4	0.028355238	0.02491255	0.017331383	*GJD3;RARA*
chr12	57505529	57505882	*	3	0.028372143	-0.009859702	-0.005716636	*STAT6*
chr13	112760929	112761740	*	10	0.02840043	-0.018151246	-0.009518714	*NA*
chr14	95876455	95877009	*	5	0.028409182	-0.011086849	-0.000683463	*NA*
chr7	45024931	45025620	*	3	0.028409182	0.023395053	0.01339354	*SNHG15;SNORA9*
chr4	2278261	2278810	*	5	0.028409265	0.013561189	0.008608275	*ZFYVE28*
chr14	36988217	36988446	*	4	0.0284139	-0.02208383	-0.001958583	*NKX2-1;SFTA3*
chr2	234112793	234113372	*	4	0.028449414	0.035104081	0.018503125	*INPP5D*
chr8	37756942	37757338	*	8	0.028454613	-0.009909806	-0.003063796	*RAB11FIP1*
chr19	30107968	30108105	*	3	0.02846739	0.020164741	0.006764762	*POP4*
chr2	47142867	47143656	*	13	0.028468861	0.019673486	0.000153221	*MCFD2;TTC7A*
chr1	29508578	29508888	*	9	0.02847283	0.027101032	0.004084229	*SRSF4*
chr9	130487227	130487287	*	3	0.028499428	-0.020717263	-0.020068743	*PTRH1;TTC16*
chr1	197115755	197116190	*	7	0.028507786	-0.014674451	-0.007842348	*ASPM*
chr7	54794693	54794760	*	3	0.028531321	-0.028048536	-0.004211623	*NA*
chr17	66511045	66511248	*	5	0.028557429	-0.021621671	-0.014656881	*PRKAR1A*
chr8	67782943	67783229	*	3	0.028569343	-0.014406303	-0.008987848	*MCMDC2*
chr15	27109950	27110089	*	3	0.028609066	-0.006243236	-0.005516015	*GABRA5;GABRB3*
chr16	54967024	54967786	*	5	0.028615667	0.025724826	0.006886091	*CRNDE;IRX5*
chr3	49893745	49894441	*	13	0.028648191	-0.02216899	-0.00076672	*TRAIP*
chr1	112532078	112532258	*	3	0.0286502	-0.05184735	-0.016048621	*KCND3;LINC01750*
chr1	11986612	11986842	*	5	0.028664858	-0.022836594	-0.005419617	*KIAA2013*
chr7	2671535	2671838	*	3	0.028664858	-0.011116767	-0.005301637	*TTYH3*
chr6	17707018	17707630	*	7	0.028677906	0.009550708	-0.000510114	*NUP153*
chr2	8711042	8711256	*	6	0.028714131	-0.014343388	-0.001986116	*NA*
chr15	69707986	69708380	*	3	0.028721676	0.017586694	0.003615261	*KIF23*
chr17	47208515	47208775	*	3	0.028728775	0.021475168	0.019049398	*B4GALNT2*
chr8	38192449	38192662	*	3	0.028735932	0.040937097	0.019285607	*NSD3*

chr8	146176420	146176685	*	5	0.028743356	0.020561607	0.001328731	*ZNF16*
chr8	8749074	8749494	*	4	0.028774538	0.018278083	0.008572856	*MFHAS1*
chr1	3125906	3126365	*	4	0.028825472	-0.021980895	-0.012175299	*PRDM16*
chr1	28422834	28423139	*	4	0.028846612	-0.017685456	-0.002232013	*NA*
chr2	105469514	105470050	*	5	0.028878744	0.016820828	8.06E-05	*PANTR1;POU3F3*
chr2	24583121	24583610	*	6	0.028892341	-0.014468324	-0.00553518	*ITSN2*
chr1	156561334	156561947	*	10	0.028915172	-0.011186463	-0.001537656	*NAXE*
chr3	147106489	147106890	*	4	0.028951847	-0.019039332	-0.006707663	*ZIC4*
chr3	161089247	161090118	*	11	0.028955693	0.010288138	0.000893935	*SPTSSB*
chr1	64240654	64241013	*	5	0.028957523	-0.009646267	-0.00435374	*ROR1*
chr19	675390	676009	*	5	0.028958212	0.030758288	0.013883262	*FSTL3*
chr11	120856646	120856918	*	3	0.028958212	-0.014542675	-0.005195828	*GRIK4*
chr11	61511574	61512109	*	4	0.028962179	0.016796383	0.008786536	*DAGLA*
chr15	81195587	81195797	*	4	0.028970499	-0.011230484	0.003711398	*CEMIP*
chr13	111301487	111301774	*	3	0.028975376	0.020670275	0.009290364	*CARS2*
chr6	82460558	82461621	*	6	0.028989627	-0.017265318	-0.006207207	*TENT5A*
chr11	575426	576060	*	4	0.029016108	-0.025815326	-0.007958924	*LOC143666;PHRF1*
chr5	60240481	60241003	*	10	0.029020194	-0.016313511	-0.000932564	*ERCC8;NDUFAF2*
chr20	62716332	62716606	*	4	0.029021418	0.019376646	0.012807274	*LKAAEAR1;OPRL1*
chr1	66258022	66258760	*	7	0.029035662	-0.023017034	-0.009378316	*PDE4B*
chr17	39680217	39680662	*	4	0.029071195	0.02703785	0.010580367	*JUP;KRT15;KRT19*
chr19	54327437	54327847	*	5	0.029077108	-0.011969927	-0.006402619	*NLRP12*
chr6	1410167	1410497	*	4	0.029103117	-0.031898676	-0.014479707	*NA*
chr12	6472963	6473643	*	6	0.029125936	-0.030092022	-0.007454871	*SCNN1A*
chr7	151504844	151505116	*	3	0.029125936	-0.027189495	-0.016232028	*PRKAG2*
chr17	4459073	4459774	*	3	0.029125936	0.014045551	0.007784668	*MYBBP1A*
chr16	2013110	2013573	*	3	0.029128349	0.021975511	0.020211221	*RNF151;RPS2;SNHG9;SNORA10;SNORA64;SNORA78*
chr9	139091378	139091875	*	4	0.029150871	-0.026567428	-0.011403034	*LHX3*
chr1	54411742	54412009	*	9	0.029157653	-0.032127476	-0.007012401	*HSPB11;LRRC42*
chr15	101970070	101971041	*	3	0.029198058	0.019041231	-0.001944302	*PCSK6*
chr6	108489795	108490485	*	5	0.029202968	0.01790888	0.002431313	*NR2E1*
chr10	105361068	105362057	*	4	0.029213126	0.025320675	0.020362099	*SH3PXD2A*
chr1	203763196	203763498	*	4	0.029240696	-0.015656597	-0.004232487	*ZBED6;ZC3H11A*
chr1	153971125	153971763	*	3	0.029269256	-0.0122661	-0.004016378	*NUP210L*
chr8	11560779	11561724	*	12	0.029289583	0.016927423	0.002931022	*GATA4*
chr5	153418202	153418977	*	18	0.029313509	0.012833524	-0.000399894	*FAM114A2;MFAP3*
chr1	33909498	33909802	*	3	0.029313509	0.016599602	0.009864421	*NA*
chr1	228295437	228295568	*	3	0.029333835	0.015530713	0.014013205	*C1orf35;MRPL55*
chr16	30023515	30024074	*	7	0.029350301	0.03829099	0.011797657	*BOLA2;DOC2A*
chr17	26899172	26899686	*	5	0.02935059	0.025977827	0.001137725	*PIGS*
chr1	100503364	100503886	*	8	0.029381303	0.008236455	0.001908403	*MFSD14A*
chr1	3159568	3159645	*	3	0.029402934	0.020495503	0.006656644	*PRDM16*
chr15	85933153	85933934	*	5	0.029405947	0.014443466	0.002674097	*AKAP13*
chr22	29702335	29702866	*	11	0.029414762	-0.010361619	0.00088938	*GAS2L1*
chr1	61519888	61520099	*	3	0.029423783	-0.015226037	-0.008252401	*NA*
chr10	124906421	124906859	*	5	0.029440079	-0.015914989	-0.006993044	*HMX2*
chr6	27648004	27648605	*	5	0.029440701	-0.030777399	-0.014949771	*NA*
chr21	40451371	40451934	*	3	0.029444486	-0.017734239	-0.016450906	*NA*
chr19	45997414	45998083	*	4	0.029454401	0.024917738	0.012228511	*PPM1N;RTN2*
chr22	29707524	29708028	*	3	0.029454401	0.023494894	0.004804479	*GAS2L1*
chr19	19550083	19550799	*	4	0.029456679	0.011600755	0.004584158	*GATAD2A*
chr1	16068861	16069045	*	3	0.02947721	0.027529249	0.017204285	*TMEM82*
chr17	7217969	7219403	*	15	0.029479167	0.015448188	0.002004325	*GPS2;NEURL4*
chr12	124118982	124119250	*	3	0.029492191	0.020455644	0.008276056	*EIF2B1;GTF2H3*
chr14	68141349	68142077	*	10	0.029495009	0.018317212	-0.000232968	*VTI1B*
chr11	57044759	57045318	*	3	0.029510127	-0.012743016	-0.002773663	*NA*
chr3	15482242	15482647	*	5	0.029524761	0.015074589	0.000699606	*EAF1*
chr9	78504994	78505312	*	4	0.029527161	-0.005049474	-0.003620281	*PCSK5*
chr7	96641558	96642163	*	3	0.0295368	-0.038076866	-0.020067191	*DLX6-AS1*
chr6	109768377	109768634	*	3	0.029594865	-0.015416083	-0.011510647	*MICAL1*
chr14	54413371	54413931	*	6	0.029637616	-0.011706437	-0.007908881	*NA*
chr20	33759753	33760017	*	4	0.029644767	-0.013920821	-0.006463776	*EDEM2;PROCR*
chr15	40268610	40269214	*	5	0.029672394	0.031719617	0.027310763	*EIF2AK4*
chr1	16009778	16010345	*	3	0.029672394	0.034612036	0.024069889	*PLEKHM2*
chr8	36641771	36641924	*	4	0.029684959	-0.018613599	-0.001553438	*KCNU1*
chr8	143781204	143781406	*	7	0.029686957	-0.034789703	-0.012581983	*LY6K*

chr16	30106682	30107106	*	4	0.029696793	-0.023638898	-0.008998128	*BOLA2;TBX6;YPEL3*
chr11	70395310	70395513	*	3	0.029716844	0.014561611	-0.000820699	*SHANK2*
chr21	46351920	46352193	*	3	0.029722714	0.015107852	0.003139738	*ITGB2*
chr6	146349312	146349638	*	4	0.029733215	0.012013698	-0.001707413	*GRM1*
chr1	20959617	20960202	*	8	0.029746063	-0.013369197	-0.006041828	*PINK1*
chr12	89703393	89703686	*	3	0.029746063	0.020214376	0.017376078	*NA*
chr16	50717571	50718139	*	3	0.029746083	0.01362288	0.005454341	*SNX20*
chr7	24323559	24323939	*	8	0.029759656	-0.008002321	-0.002126572	*NPY*
chr15	37170381	37170554	*	3	0.029832795	-0.028923358	-0.013996329	*LOC145845*
chr20	62317412	62317640	*	3	0.029834484	0.015305503	0.011858207	*RTEL1;RTEL1-TNFRSF6B*
chr10	82363213	82363434	*	5	0.029843247	-0.026933593	-0.007914514	*SH2D4B*
chr6	158447877	158448134	*	3	0.029849483	-0.008910671	-0.007252425	*SYNJ2*
chr11	76493692	76494226	*	8	0.029865613	-0.014253806	-0.004599785	*TSKU*
chr5	135188170	135188477	*	4	0.029865613	0.021351559	0.015687722	*SLC25A48*
chr7	73496735	73497616	*	5	0.029867913	0.019675877	0.010568295	*LIMK1*
chr9	116971578	116972431	*	4	0.029875128	0.025096832	0.014215663	*COL27A1;MIR455*
chr16	71571613	71572050	*	3	0.029875128	-0.036627581	-0.011015132	*CHST4*
chr7	64342247	64342997	*	4	0.029889977	-0.023608657	-0.005749007	*ZNF273*
chr1	2885085	2885244	*	4	0.029889977	0.028902019	0.018888181	*NA*
chr20	43935222	43935551	*	10	0.029894555	0.034710732	0.015866414	*MATN4;RBPJL*
chr17	65242413	65242892	*	5	0.029894555	0.015760958	0.009158553	*HELZ*
chr3	122645347	122645440	*	3	0.029926473	0.016486865	0.012104312	*SEMA5B*
chr14	23351487	23351876	*	3	0.029927738	-0.011163564	-0.008582318	*REM2*
chr11	94226637	94227380	*	13	0.029941271	0.007312282	-0.000473679	*ANKRD49;MRE11*
chr19	5338789	5339092	*	4	0.029957968	0.014734245	-0.001032934	*PTPRS*
chr6	41254219	41254885	*	6	0.0299685	-0.018731218	-0.006306112	*TREM1*
chr5	179740743	179741120	*	4	0.029977938	-0.115525323	-0.069213857	*GFPT2*
chr1	28905410	28905573	*	3	0.029982373	0.036542955	0.012063524	*SNHG12;SNORD99*
chr11	69970464	69970618	*	3	0.030005298	-0.017481942	-0.001617358	*ANO1*
chr7	128094710	128095027	*	3	0.030032227	0.024062294	0.018810017	*HILPDA*
chr12	48396670	48397225	*	4	0.030033385	-0.008438982	-0.001703918	*COL2A1*
chr5	138940694	138941220	*	6	0.030045825	-0.00538204	-0.000404539	*UBE2D2*
chr8	141290075	141290228	*	3	0.030062671	0.018167098	0.007159651	*TRAPPC9*
chr12	48394962	48395490	*	3	0.030065275	-0.007648168	-0.001808491	*COL2A1*
chr19	11376205	11376638	*	3	0.03009105	0.028368804	0.021985525	*DOCK6*
chr21	40685679	40686388	*	4	0.03013088	0.004459199	-0.000251131	*BRWD1;BRWD1-AS1*
chr16	55404939	55405200	*	3	0.030141337	-0.02459302	-0.012679047	*NA*
chr19	42680337	42680534	*	3	0.030147459	-0.00667763	-0.001602672	*NA*
chr1	227750845	227751935	*	15	0.030151137	0.031178345	0.003093844	*ZNF678*
chr2	106362217	106362717	*	3	0.030151137	-0.007778981	-0.004149135	*NCK2*
chr20	44649524	44650449	*	10	0.030185454	-0.029542831	-0.00739349	*SLC12A5*
chr15	32162415	32162783	*	3	0.030189834	-0.043340438	-0.023662815	*OTUD7A*
chr13	113636679	113636953	*	3	0.030226325	0.01798394	0.012927475	*MCF2L*
chr1	235529879	235530995	*	10	0.030258721	0.02977905	-0.000580201	*TBCE*
chr19	11032005	11032172	*	3	0.030262906	0.022526325	0.011682472	*CARM1*
chr10	125652310	125652421	*	3	0.030272951	0.015812871	0.009677763	*CPXM2*
chr18	59560091	59560289	*	3	0.030278782	-0.016539787	-0.005236506	*RNF152*
chr9	138194071	138195156	*	4	0.030304553	0.026678363	0.006700532	*NA*
chr19	48018185	48018329	*	4	0.030310327	0.003240931	0.000709732	*NAPA*
chr7	27188364	27188770	*	3	0.030310327	0.027328566	0.019452948	*HOXA-AS3;HOXA6*
chr1	38516658	38517272	*	4	0.030311007	0.015927104	0.00809988	*POU3F1*
chr12	53074257	53074328	*	3	0.030311998	-0.022206198	-0.000460075	*KRT1*
chr1	242161780	242161881	*	3	0.030313305	-0.027798518	-0.015442242	*MAP1LC3C*
chr19	8008671	8009199	*	10	0.030313569	-0.011628952	-0.001211703	*TIMM44*
chr16	87866696	87866833	*	3	0.030332129	-0.019726346	-0.014384428	*SLC7A5*
chr17	39387925	39388156	*	4	0.030373323	0.022086921	0.011516711	*KRTAP9-3*
chr2	9563077	9563880	*	14	0.030401485	-0.018156315	-0.00291149	*CPSF3;ITGB1BP1*
chr9	72043637	72044252	*	4	0.030409399	0.024438098	0.006938915	*APBA1*
chr16	125896	126451	*	5	0.030409594	0.018121984	-0.001228797	*MPG;RHBDF1*
chr3	151986151	151986610	*	5	0.030414419	-0.014956329	-0.001373791	*MBNL1;MBNL1-AS1*
chr4	1803148	1803443	*	3	0.030425704	0.02047968	0.01308936	*FGFR3*
chr21	40145404	40146149	*	5	0.030426893	0.014836953	-0.001167419	*LINC00114*
chr12	122458893	122460105	*	7	0.030430443	-0.023163962	-0.013354815	*BCL7A*
chr17	7145208	7145538	*	5	0.030435145	-0.032447621	-0.007706823	*GABARAP;PHF23*
chr1	235256497	235257141	*	6	0.030499983	0.026804003	0.012820792	*NA*
chr11	121466430	121466629	*	3	0.030503553	-0.0262608	2.20E-05	*SORL1*

chr3	62358980	62359677	*	6	0.030509531	0.007785329	0.001299736	*FEZF2*
chr15	51398063	51398559	*	4	0.030517064	0.022313985	-0.002196713	*TNFAIP8L3*
chr5	132362086	132362900	*	10	0.030527289	-0.019462491	-0.005812187	*ZCCHC10*
chr7	175423	175713	*	3	0.030527289	0.021075591	0.010839824	*NA*
chr14	91770052	91770418	*	4	0.030540656	0.01609889	0.007758082	*CCDC88C*
chr7	98424185	98424289	*	3	0.03054397	-0.028813037	-0.025140263	*NA*
chr6	80816077	80816322	*	8	0.030551307	-0.009871442	-0.001573831	*BCKDHB*
chr3	51749782	51749897	*	4	0.030551307	0.020297444	0.012384295	*GRM2*
chr19	52207200	52207792	*	8	0.030562383	0.009054088	-0.001230815	*SPACA6*
chr19	46094809	46095562	*	5	0.030602291	0.025870524	0.006925277	*GPR4;OPA3*
chr20	43540202	43540309	*	3	0.030606563	-0.023420126	-0.016595714	*PABPC1L*
chr17	9479058	9479665	*	9	0.030643841	-0.018608822	-0.002935117	*CFAP52;STX8*
chr12	53574537	53574937	*	5	0.030663154	0.013005645	0.000655225	*CSAD;ZNF740*
chr20	61590751	61591066	*	3	0.03067276	-0.048455427	-0.045342149	*SLC17A9*
chr4	117847088	117847545	*	4	0.030752861	0.016344772	0.011076481	*NA*
chr8	125384727	125384848	*	3	0.030752861	0.013864045	0.005223416	*TMEM65*
chr20	23401804	23402586	*	11	0.030773428	-0.008394252	-0.002098262	*NAPB*
chr1	59250642	59250966	*	7	0.030774043	0.012708329	0.005214965	*JUN;LINC01135*
chr6	29894940	29895260	*	8	0.030789462	-0.061304133	-0.02246705	*HCG4B;HLA-G;HLA-H;HLA-J*
chr19	12551876	12552341	*	5	0.030789462	0.016735295	0.000626181	*ZNF443*
chr6	91053343	91054324	*	3	0.03080384	0.017972612	0.005893401	*NA*
chr12	55378169	55378884	*	7	0.030828286	-0.017547797	-0.005574284	*TESPA1*
chr16	4837760	4838530	*	6	0.030829925	0.020044853	0.009022516	*SEPTIN12;SMIM22*
chr4	114683176	114683375	*	5	0.03084226	-0.030225283	-0.005726198	*CAMK2D*
chr6	45631106	45631407	*	4	0.030869353	0.013200907	0.011047917	*NA*
chr11	118530412	118530628	*	3	0.030914209	-0.018801083	-0.012274285	*TREH*
chr1	63787170	63787283	*	4	0.030937239	0.036512572	0.014372139	*FOXD3;LINC00466*
chr15	66796922	66797614	*	9	0.030940556	-0.026362539	-0.006667629	*RPL4;SNORD16;SNORD18A;SNORD18B;SNORD18C;ZWILCH*
chr7	105662697	105662884	*	3	0.03097759	-0.017180499	-0.011501154	*CDHR3*
chr3	194934291	194934653	*	3	0.031009583	0.016238869	0.003581092	*XXYLT1*
chr14	105881346	105881968	*	3	0.031035282	0.009609438	0.002960075	*MTA1*
chr19	45884900	45886078	*	5	0.031061712	0.033513054	0.017663118	*PPP1R13L*
chr5	10485347	10485736	*	3	0.031066211	-0.016323517	-0.012686585	*NA*
chr21	32255112	32255313	*	3	0.031102072	0.024653589	0.007135673	*KRTAP11-1*
chr14	35098784	35099524	*	8	0.031127331	0.016592966	0.001641891	*SNX6*
chr19	10613180	10613553	*	6	0.031127331	0.00810497	0.001395361	*KEAP1*
chr1	53067365	53067832	*	4	0.031127331	0.018956473	0.008803736	*GPX7*
chr2	44065964	44067042	*	10	0.031145145	0.02185351	0.003292802	*ABCG5;ABCG8*
chr2	225450612	225450859	*	3	0.031168276	-0.007193798	-0.001430755	*CUL3*
chr17	39769012	39769294	*	5	0.031189314	-0.020195204	-0.006987253	*JUP;KRT16*
chr14	104643224	104643907	*	4	0.031197156	0.017507297	0.009883217	*KIF26A*
chr4	119809687	119810066	*	9	0.031209407	-0.01648762	-0.007696774	*SYNPO2*
chr21	43823604	43824262	*	10	0.031232305	-0.022285005	-0.010665385	*UBASH3A*
chr5	3602558	3603280	*	3	0.031232391	-0.024176417	-0.014273102	*NA*
chr7	149120109	149120687	*	4	0.031256651	0.023025757	0.002451422	*NA*
chr7	143017866	143018410	*	5	0.031289791	-0.015676343	0.005101362	*CLCN1*
chr2	95663959	95664359	*	4	0.031308829	-0.01781282	-0.011087972	*NA*
chr7	79083635	79084166	*	10	0.031315105	-0.017150916	-0.007516171	*MAGI2;MAGI2-AS3*
chr11	557273	557803	*	5	0.031336459	0.030156541	0.013111688	*LMNTD2;RASSF7*
chr14	54082109	54082227	*	3	0.031346729	-0.023278997	-0.01110378	*NA*
chr13	31039577	31040351	*	9	0.031364733	-0.019097619	-0.005948374	*HMGB1*
chr1	57111122	57111499	*	4	0.031391159	-0.024419417	-0.008055676	*PRKAA2*
chr14	21092936	21094481	*	7	0.03140627	0.0249397	0.006519761	*NA*
chr17	38717206	38717275	*	3	0.031435204	-0.023638299	-0.017741396	*CCR7*
chr20	36932377	36932553	*	5	0.031450546	-0.014136786	-0.007631805	*BPI*
chr7	23508189	23508734	*	4	0.031468141	-0.008834215	-0.001379758	*IGF2BP3*
chr7	734765	734968	*	3	0.031483399	0.016368381	0.01048164	*PRKAR1B*
chr16	89132868	89133406	*	4	0.031489845	0.014733402	0.00433511	*NA*
chr21	34907001	34907910	*	3	0.031491404	0.015136959	0.003988023	*GART*
chr1	33283384	33283867	*	7	0.031505976	-0.012342326	-0.002475688	*S100PBP;YARS1*
chr5	153865895	153866216	*	3	0.031510577	-0.01201226	-0.010142184	*NA*
chr11	47664006	47664550	*	10	0.031534868	0.014220055	0.001106558	*MTCH2*
chr17	46622154	46622817	*	9	0.031562434	0.019691966	0.006201643	*HOXB-AS1;HOXB2*
chr7	155674731	155675480	*	4	0.031562434	0.035306729	0.015630866	*NA*
chr16	88850218	88850755	*	3	0.03159997	0.012162399	0.006268679	*PIEZO1*
chr14	76734327	76734850	*	6	0.031628444	-0.04300609	-0.020972573	*NA*

chr10	132461229	132461281	*	3	0.031645606	0.026774429	0.013764381	*NA*
chr12	39115589	39116462	*	4	0.031657122	-0.024212078	0.002803964	*CPNE8*
chr22	19928616	19929286	*	11	0.031699913	0.023962111	-0.000187055	*COMT;TXNRD2*
chr19	19302345	19303269	*	10	0.031768208	-0.014554204	-0.006391018	*BORCS8;BORCS8-MEF2B;RFXANK*
chr1	212003977	212004804	*	14	0.031775697	0.011742845	0.000466434	*LPGAT1*
chr15	89089946	89090510	*	6	0.031775697	0.011497187	-0.003473096	*DET1*
chr17	73316280	73316721	*	8	0.03183553	0.032284434	0.007843728	*GRB2*
chr2	95787254	95787596	*	3	0.03184334	-0.016770086	-0.002534724	*MRPS5*
chr11	31391482	31391912	*	8	0.031855739	-0.029292376	-0.005978775	*DCDC1;DNAJC24*
chr16	1493343	1493859	*	5	0.031855739	0.028147889	0.015903582	*CCDC154*
chr6	31674553	31674897	*	3	0.031855739	-0.025082099	-0.001079677	*ABHD16A;LY6G6F*
chr19	519609	519885	*	3	0.031855739	0.032073561	0.028070296	*TPGS1*
chr1	150207467	150208087	*	3	0.031871094	-0.018182047	-0.005294488	*ANP32E*
chr17	38973872	38974206	*	3	0.03189763	-0.014998887	-0.012339542	*TMEM99*
chr5	147647678	147647955	*	3	0.031906616	0.029191575	0.00867258	*SPINK13*
chr22	51016839	51017432	*	9	0.031908124	0.026858066	0.016977349	*CHKB;CHKB-CPT1B;CHKB-DT;CPT1B*
chr19	32896872	32897255	*	7	0.031910443	-0.01254897	-0.002429017	*DPY19L3;LOC400684*
chr11	68192511	68192736	*	4	0.031918024	0.020285565	0.015002548	*LRP5*
chr5	179771539	179771851	*	3	0.031940677	0.018063718	0.013408206	*GFPT2*
chr19	54134711	54135150	*	6	0.031942503	0.025038705	0.011408307	*DPRX*
chr2	200333445	200334137	*	5	0.031950281	0.019919129	0.002597025	*SATB2;SATB2-AS1*
chr7	127983655	127984071	*	9	0.031979787	-0.050290391	-0.007134812	*RBM28*
chr11	1241885	1242427	*	4	0.032033451	0.011391057	0.005290052	*MUC5B*
chr10	101295724	101296221	*	4	0.032057425	0.028047267	0.016230261	*NKX2-3*
chr8	17941088	17941982	*	8	0.032097699	-0.010835641	-0.000714329	*ASAH1*
chr19	44084271	44084933	*	6	0.03209998	0.021660499	0.014324692	*PINLYP;XRCC1*
chr17	13504399	13504687	*	4	0.032109751	-0.019806991	-0.006510591	*HS3ST3A1*
chr4	175132842	175133103	*	3	0.032109751	-0.00915772	-0.008311281	*NA*
chr7	7605808	7606396	*	4	0.032111552	0.012840488	0.001029126	*MIOS*
chr19	48949061	48949463	*	4	0.032111611	-0.008133895	-0.005316061	*GRWD1*
chr12	45269992	45271935	*	21	0.03213021	0.033339867	0.001840688	*NELL2*
chr10	98741495	98741960	*	4	0.032172344	0.015910795	0.000122475	*LCOR*
chr7	100809985	100810273	*	3	0.032172344	-0.012903946	-0.005857474	*VGF*
chr4	6694923	6695698	*	9	0.032180016	-0.015350401	-0.006102305	*S100P*
chr10	80126725	80127261	*	5	0.032213325	0.030442394	0.010001399	*LINC00595;LINC00856*
chr16	4898226	4898667	*	6	0.032216268	0.015205145	0.000925856	*GLYR1;UBN1*
chr20	45439769	45440228	*	4	0.032216403	-0.026880026	-0.003211895	*NA*
chr1	36689760	36690560	*	9	0.032261972	-0.004319152	-0.001381293	*THRAP3*
chr16	67360520	67361708	*	9	0.032273908	-0.019828364	-0.008416368	*KCTD19;LRRC36*
chr6	33273011	33273128	*	3	0.032280072	-0.021091579	-0.008673458	*TAPBP*
chr18	47807503	47807700	*	4	0.032307788	-0.018733274	-0.013168314	*MBD1*
chr13	43294435	43294719	*	3	0.032355523	-0.035117227	-0.021245479	*NA*
chr2	583872	583975	*	3	0.032376731	0.024731418	0.011848212	*NA*
chr6	100442489	100442603	*	3	0.032382065	-0.030390039	-0.022949817	*MCHR2;MCHR2-AS1*
chr1	205391381	205391881	*	4	0.032402854	-0.017082981	-0.005152855	*LEMD1*
chr17	38975396	38975449	*	3	0.03242678	-0.026014292	-0.018462941	*KRT10;TMEM99*
chr2	86831057	86831407	*	3	0.032438597	-0.023188175	-0.009352716	*RNF103;RNF103-CHMP3*
chr10	133999471	134000009	*	3	0.032440967	-0.014200207	-0.008838411	*DPYSL4*
chr15	67706106	67706575	*	3	0.032460914	0.02520781	0.000999272	*IQCH;IQCH-AS1*
chr2	101434611	101435167	*	3	0.032482214	-0.016987834	-0.000530411	*NPAS2*
chr14	61448125	61448281	*	4	0.032483559	-0.006312199	-0.003588241	*SLC38A6;TRMT5*
chr14	70883772	70883883	*	4	0.032510986	-0.039152484	-0.013135409	*SYNJ2BP;SYNJ2BP-COX16*
chr10	134362678	134363419	*	4	0.03255435	0.020644989	0.008791729	*INPP5A*
chr17	39642907	39643395	*	3	0.032557103	-0.022658652	-0.012222057	*KRT36*
chr17	72879418	72879892	*	3	0.032561408	-0.012324163	-0.004300171	*FADS6*
chr6	138820503	138820840	*	3	0.032577511	-0.014266708	-0.009157631	*NHSL1*
chr8	103424275	103424477	*	3	0.032585911	-0.006444095	-0.00080643	*UBR5*
chr11	71814220	71814729	*	10	0.032595071	0.009802243	-0.00181854	*LAMTOR1;LRTOMT*
chr1	236246704	236247215	*	3	0.03261184	0.028147033	0.002360012	*NA*
chr12	109162390	109162748	*	6	0.032653087	0.016672376	0.005434378	*NA*
chr15	39887718	39888093	*	3	0.032656729	0.007877939	0.001720259	*THBS1*
chr8	145137755	145138225	*	6	0.032689114	-0.009072529	0.001262262	*GPAA1*
chr4	54243303	54243829	*	9	0.032690018	-0.022458462	-0.005180041	*FIP1L1;PDGFRA*
chr5	121647154	121647642	*	7	0.032703463	-0.034498925	-0.011862445	*SNCAIP*
chr2	242926331	242926621	*	3	0.032734989	0.013604119	0.012526101	*NA*
chr15	29424197	29424512	*	3	0.032828444	-0.020646733	-0.00925718	*FAM189A1*

chr19	1242098	1242323	*	3	0.032833718	0.019310409	0.008244108	*ATP5F1D;CBARP*
chr16	84150400	84150746	*	7	0.032854842	-0.019089346	-0.004521305	*MBTPS1*
chr1	2262232	2262474	*	3	0.032859704	0.018516872	0.009328395	*MORN1*
chr2	105472193	105472469	*	3	0.032958447	-0.018964875	-0.002762663	*PANTR1;POU3F3*
chr2	208489070	208489448	*	7	0.032958983	-0.009786126	-0.001170491	*METTL21A*
chr19	49991271	49991852	*	5	0.032968407	-0.007938361	-0.003045593	*RPL13A;SNORD32A;SNORD33;SNORD34;SNORD35A*
chr2	241458886	241459227	*	4	0.032975491	-0.028119053	-0.018664044	*ANKMY1*
chr11	65189075	65189896	*	4	0.032975491	-0.007178553	-0.001867598	*NEAT1*
chr19	50192542	50192739	*	3	0.032987205	0.02755296	0.020178553	*ADM5;CPT1C*
chr5	146568193	146568702	*	4	0.032990514	-0.01229483	-0.004496016	*NA*
chr14	77923881	77924595	*	12	0.033019295	-0.008432674	-0.000820279	*AHSA1;VIPAS39*
chr2	101086756	101086963	*	4	0.033032183	-0.019701846	-0.002310254	*NMS*
chr2	61403867	61405051	*	11	0.033033674	-0.013975274	-0.003401434	*AHSA2P*
chr3	48541379	48541507	*	3	0.03303852	0.010357231	0.007390297	*SHISA5*
chr13	37006063	37006439	*	8	0.033081971	-0.013208198	-0.005473948	*CCNA1*
chr1	27719310	27719463	*	3	0.033095909	0.011977958	-0.001138383	*GPR3*
chr19	46498049	46498389	*	7	0.033109637	-0.012632539	-0.004430401	*CCDC61*
chr1	23894390	23894902	*	4	0.033112162	-0.023049902	-0.011023078	*NA*
chr3	122640625	122640935	*	3	0.033190012	-0.018553507	-0.016565145	*SEMA5B*
chr13	27580727	27580970	*	4	0.033214503	-0.034505058	-0.016424029	*NA*
chr9	14992085	14992232	*	3	0.033227936	-0.037264126	-0.020375954	*LOC389705*
chr20	51109896	51110040	*	3	0.033234018	-0.015847481	-0.00878205	*NA*
chr11	75901632	75902270	*	3	0.0332497	-0.027081932	-0.005879633	*WNT11*
chr6	157469646	157470150	*	5	0.033250046	-0.014501562	-0.006798926	*ARID1B*
chr14	21511112	21511676	*	3	0.033263743	0.016104836	0.011362524	*NDRG2;RNASE7*
chr3	184880078	184880944	*	7	0.033265683	0.022928818	0.00415158	*EHHADH-AS1*
chr9	16277012	16277601	*	3	0.033265964	0.017990456	0.011671252	*C9orf92*
chr2	121992375	121992797	*	3	0.033276534	-0.015126659	-0.006418228	*TFCP2L1*
chr15	100939779	100940134	*	3	0.033316932	-0.018002308	-0.006577861	*NA*
chr17	73761301	73761710	*	9	0.033323493	-0.02649213	-0.00546209	*GALK1*
chr7	27149139	27150403	*	5	0.033330975	0.027305897	0.01346367	*HOXA3*
chr10	105036495	105036863	*	8	0.033331061	0.008864659	-0.000156398	*INA*
chr11	108337942	108338461	*	10	0.03334832	-0.0116472	-0.002966107	*C11orf65*
chr10	3823790	3824555	*	7	0.03334832	0.018487117	0.010873415	*KLF6*
chr4	139345773	139345911	*	3	0.03334832	0.021184725	0.014205785	*NA*
chr17	80289437	80289701	*	5	0.033377844	0.023551132	0.014475124	*SECTM1*
chr12	48197060	48197491	*	3	0.033381285	-0.020954724	-0.016547803	*HDAC7*
chr16	69354837	69355091	*	5	0.033392185	0.01413665	0.007890717	*VPS4A*
chr22	41939981	41940480	*	5	0.033410441	-0.030956328	-0.003758143	*POLR3H*
chr4	83483483	83483735	*	5	0.033415608	0.011556689	0.001707628	*TMEM150C*
chr11	22696087	22696443	*	4	0.033453453	0.022600951	0.003125727	*GAS2*
chr8	41654331	41654557	*	3	0.033495921	0.022294392	0.014230371	*ANK1*
chr20	45179226	45179413	*	5	0.033522866	-0.013370147	-0.008482843	*OCSTAMP*
chr12	93963292	93963396	*	4	0.03355116	-0.030855695	-0.007538985	*SOCS2;SOCS2-AS1*
chr8	143210309	143210482	*	3	0.033552254	-0.023371334	-0.018626772	*NA*
chr1	109656145	109656325	*	4	0.033560883	0.019670595	0.003067586	*C1orf194;ELAPOR1*
chr17	18163183	18163513	*	3	0.033566861	-0.010200092	-0.000505621	*FLII;MIEF2*
chr3	6003083	6003401	*	3	0.033598188	0.021458646	0.000607768	*NA*
chr2	74212731	74212961	*	3	0.033599797	-0.010393677	-0.005987409	*TET3*
chr18	47341300	47341307	*	3	0.033600662	-0.01487054	-0.01165083	*ACAA2*
chr10	114711288	114712217	*	5	0.033649612	-0.020274013	-0.007167963	*TCF7L2*
chr7	150872023	150872218	*	3	0.033649612	-0.029302973	-0.020509235	*NA*
chr15	76440405	76440448	*	3	0.033650295	-0.038916548	-0.022120532	*TMEM266*
chr6	32363121	32363268	*	4	0.033654471	-0.020512706	-0.005303117	*BTNL2*
chr21	46876385	46876904	*	3	0.033673455	0.027442978	0.009200032	*COL18A1*
chr2	234741670	234741841	*	3	0.033675342	0.024147213	0.015255563	*NA*
chr17	80809619	80809670	*	3	0.033698311	0.017700303	0.010581398	*TBCD*
chr2	130345044	130345405	*	4	0.033699129	-0.031422881	-0.020194978	*NA*
chr5	68788469	68788497	*	4	0.03370325	-0.010970489	-0.008061541	*OCLN*
chr7	157932168	157932715	*	4	0.033732755	-0.03276976	0.004004633	*PTPRN2*
chr17	71160748	71161160	*	5	0.033780323	-0.010167201	-0.00699832	*SSTR2*
chr19	37099006	37099702	*	4	0.033787769	-0.012530152	-0.004058204	*ZNF382;ZNF529*
chr5	76382539	76383494	*	12	0.033857849	-0.01217975	-0.001871147	*ZBED3;ZBED3-AS1*
chr17	2240844	2241027	*	4	0.033866535	-0.006961218	-0.003057638	*SGSM2;TSR1*
chr1	1689452	1689887	*	3	0.033866535	-0.028180615	-0.010058138	*NADK*
chr19	36499326	36499839	*	5	0.033868387	0.023123096	0.013912677	*SYNE4*

chr19	1287428	1287884	*	3	0.033868387	0.02628147	0.017964915	*EFNA2*
chr10	11047269	11047586	*	6	0.03390863	-0.029282379	-0.005132967	*CELF2*
chr15	66546648	66546713	*	3	0.033922525	-0.011973978	-0.008323357	*MEGF11*
chr11	68768879	68769194	*	3	0.033923519	0.017912588	0.007486966	*NA*
chr6	150921939	150922227	*	4	0.033935481	0.007800496	0.004006551	*PLEKHG1*
chr17	1385559	1385822	*	3	0.033950387	0.01286082	0.004854165	*MYO1C*
chr4	54931064	54931210	*	4	0.033952915	-0.008932937	-0.004035985	*CHIC2;PDGFRA*
chr1	212606756	212607111	*	3	0.033957561	0.010881202	0.00717468	*NENF*
chr16	279345	279746	*	9	0.03395803	0.028301403	0.006849952	*LUC7L*
chr17	1478463	1479213	*	5	0.033973831	-0.016047354	-0.005455974	*SLC43A2*
chr11	130184747	130185384	*	9	0.034006286	0.031013168	0.001693557	*ZBTB44*
chr1	24974498	24974715	*	3	0.03401115	0.030067818	-0.000491292	*SRRM1*
chr2	7171869	7172297	*	7	0.034027447	-0.012077267	-0.003506177	*RNF144A*
chr8	144631524	144631921	*	6	0.034046555	0.027008864	0.017783008	*GSDMD*
chr1	154151169	154151980	*	5	0.034046555	0.030726322	0.015524666	*TPM3*
chr13	112720427	112720927	*	4	0.034060188	-0.006911178	-0.00077239	*SOX1*
chr12	31742448	31743367	*	7	0.034061562	-0.019739206	-0.007197851	*DENND5B;DENND5B-AS1*
chr8	52811707	52812171	*	10	0.034081565	0.00597347	0.000582498	*PCMTD1*
chr19	17559384	17559666	*	7	0.034114897	-0.016780646	-0.008648346	*TMEM221*
chr1	109506658	109506963	*	4	0.034115288	-0.020847655	-0.009283361	*AKNAD1;CLCC1*
chr14	93813495	93813777	*	5	0.034175987	0.023883697	0.013052708	*COX8C;UNC79*
chr18	47866348	47866831	*	6	0.034187498	0.021095508	0.009098701	*NA*
chr18	268031	268453	*	9	0.034193997	0.006577647	0.00135564	*THOC1*
chr10	5932453	5932700	*	3	0.034198204	-0.03173348	-0.020061614	*ANKRD16;FBH1*
chr20	4990973	4991394	*	3	0.034216352	0.020517076	0.017973112	*SLC23A2*
chr8	54164879	54165363	*	3	0.03423453	-0.024667279	-0.016233213	*OPRK1*
chr20	18122816	18123352	*	6	0.034236459	0.016031096	0.003575625	*KAT14;PET117*
chr16	48269424	48269819	*	4	0.034289759	0.022516935	0.006575337	*ABCC11;MIR548AE2*
chr10	118935095	118935377	*	3	0.034295232	-0.007149036	-0.002428187	*NA*
chr19	19026245	19026362	*	3	0.034295232	0.022627962	0.006132459	*COPE;DDX49;HOMER3*
chr17	4643257	4643401	*	6	0.034322252	0.027878222	0.007200809	*CXCL16;ZMYND15*
chr17	71189054	71189320	*	6	0.034322252	0.031051497	0.013415092	*COG1*
chr5	1556703	1556929	*	3	0.03433844	0.02111823	0.012637353	*NA*
chr2	110371593	110371903	*	11	0.034338997	-0.024313248	-0.012698151	*SEPTIN10;SOWAHC*
chr10	6245036	6245271	*	4	0.034354212	0.006938824	0.003405947	*PFKFB3*
chr2	131089926	131090346	*	4	0.034358891	-0.031535977	-0.014061915	*NA*
chr16	88941173	88941576	*	4	0.034369126	0.0230685	0.010829094	*CBFA2T3*
chr2	99771159	99771627	*	11	0.034400191	-0.008622308	-0.00186646	*LIPT1;MRPL30;TSGA10*
chr6	142409323	142409831	*	5	0.034429475	0.020092772	0.004458836	*NMBR*
chr17	71365482	71366201	*	5	0.034463255	-0.022123244	-0.004331205	*SDK2*
chr11	62539321	62539719	*	3	0.034488457	0.016730447	0.01001377	*TAF6L*
chr1	10363631	10364025	*	3	0.034501163	-0.020126839	-0.007881082	*KIF1B*
chr3	168865089	168865475	*	4	0.034501878	-0.023331771	-0.011698243	*MECOM*
chr6	12716655	12717192	*	6	0.034512562	-0.020461627	-0.000911256	*PHACTR1*
chr7	96635624	96636192	*	4	0.034528177	-0.01172691	-0.0081055	*DLX6;DLX6-AS1*
chr11	36296196	36296397	*	4	0.03453386	0.022195329	0.008781222	*COMMD9*
chr6	30094960	30095419	*	19	0.034539689	0.027461791	0.014957476	*NA*
chr9	109040403	109040631	*	4	0.034556152	0.024611843	0.007841506	*NA*
chr16	67427183	67427811	*	13	0.034569863	-0.020074078	-0.006487284	*TPPP3*
chr2	70528671	70529197	*	4	0.03457531	-0.029866479	-0.004753146	*FAM136A*
chr3	194304935	194305124	*	4	0.034578632	0.017582992	0.000737028	*TMEM44-AS1*
chr19	18901666	18902221	*	10	0.034580551	0.016273381	-0.004042353	*COMP*
chr9	92316223	92316413	*	3	0.03458333	0.007792879	0.001065904	*UNQ6494*
chr4	115519436	115519920	*	7	0.034605325	-0.017947249	-0.007698623	*UGT8*
chr11	65639579	65639869	*	3	0.034628385	0.015514264	0.011218007	*EFEMP2*
chr1	202857904	202858430	*	9	0.03464046	-0.010966572	-0.000756578	*RABIF*
chr14	102695264	102695515	*	5	0.034649556	0.0207039	0.008064817	*MOK*
chr9	71940287	71940578	*	3	0.034650961	-0.008200627	-0.005417649	*FAM189A2*
chr2	217277275	217277648	*	8	0.034688316	0.013797453	0.001195219	*SMARCAL1*
chr3	178789226	178789848	*	10	0.034701332	-0.034327052	-0.005347467	*ZMAT3*
chr20	48658164	48658258	*	4	0.034754441	-0.01652776	-0.01018811	*TRERNA1*
chr19	35531150	35531417	*	7	0.034755595	-0.010601989	-0.005333176	*HPN;SCN1B*
chr4	6203560	6203869	*	4	0.034757075	-0.020129241	0.00395011	*JAKMIP1;LINC02495*
chr6	43251876	43252017	*	3	0.034768545	0.021728054	0.011818079	*TTBK1*
chr6	64308512	64308590	*	3	0.034780408	-0.011859268	-0.009081478	*NA*
chr8	145114875	145115260	*	3	0.034793277	0.036593828	0.026824094	*OPLAH*

chr1	10489962	10490346	*	9	0.034798849	0.03189052	0.00822146	*CENPS;CENPS-CORT*
chr6	33588875	33590458	*	10	0.034814199	0.019768548	0.000438635	*ITPR3*
chr10	1507138	1508061	*	3	0.034825588	-0.034038597	-0.001976043	*ADARB2*
chr20	44509606	44509853	*	9	0.034827411	-0.019814534	-0.007516642	*ZSWIM1*
chr22	45899292	45899736	*	3	0.034831596	0.015293529	0.011026696	*FBLN1*
chr9	91933073	91933654	*	8	0.034841776	0.007034089	-0.001589019	*SECISBP2*
chr10	1692910	1693107	*	3	0.034879398	-0.025814451	-0.009681801	*ADARB2*
chr6	32159527	32159607	*	3	0.034887265	0.023923915	0.014727447	*GPSM3;PBX2*
chr8	23540828	23541309	*	5	0.034901573	0.011510652	3.31E-05	*NKX3-1*
chr6	109169469	109169698	*	5	0.034905614	0.00759714	0.002461285	*ARMC2*
chr1	177134087	177134454	*	8	0.034925713	-0.021459633	-0.005815339	*ASTN1*
chr13	114311898	114312327	*	3	0.034945574	0.038928483	0.01619034	*ATP4B*
chr17	34957477	34957969	*	8	0.034951396	-0.010082446	-0.00270135	*MRM1*
chr17	80898525	80899158	*	5	0.035003328	0.026246739	0.011898921	*TBCD*
chr5	10251089	10251252	*	3	0.035023794	0.004475199	-0.000143412	*ATPSCKMT;CCT5*
chr7	2250248	2250448	*	3	0.035060278	-0.011588287	-0.000808657	*MAD1L1*
chr11	6592066	6592585	*	4	0.035060323	0.04489839	0.039528971	*DNHD1*
chr6	10723028	10723588	*	10	0.035066652	-0.007841981	-0.002477207	*TMEM14C*
chr2	27294264	27295120	*	9	0.035067952	-0.00768369	-0.003121762	*OST4*
chr1	181056912	181057362	*	4	0.035067952	0.023195367	0.011920802	*IER5*
chr19	36630557	36631318	*	11	0.035071429	-0.006970624	-0.000428136	*CAPNS1*
chr19	54368958	54369133	*	4	0.035109952	0.017438887	0.009664098	*MYADM*
chr2	121257646	121257734	*	3	0.035145893	0.016774076	0.011504554	*NA*
chr2	214148664	214149035	*	9	0.035173147	-0.014707615	-0.001101592	*SPAG16;SPAG16-DT*
chr20	34544049	34544356	*	4	0.035200375	0.023625009	0.005520503	*SCAND1*
chr5	137474700	137474905	*	3	0.035223395	-0.0237452	-0.013504239	*NME5*
chr4	3208639	3209105	*	3	0.035225095	0.01998449	0.008405128	*HTT*
chr10	126303604	126303795	*	3	0.035225219	0.036070239	0.019619438	*NA*
chr12	4417093	4417170	*	3	0.035242787	0.031931196	0.031843467	*NA*
chr20	62367108	62367632	*	4	0.035276202	-0.026454851	-0.00912489	*LIME1;SLC2A4RG;ZGPAT*
chr13	113803336	113803579	*	3	0.035283249	-0.015998867	0.000799309	*F10*
chr1	986560	987502	*	6	0.035286791	0.010969569	0.002162574	*AGRN*
chr2	200336014	200336200	*	4	0.035293459	-0.007041066	-0.00101874	*SATB2;SATB2-AS1*
chr17	38020599	38021496	*	5	0.035302436	0.027732045	0.003741104	*IKZF3;ZPBP2*
chr7	75624427	75624848	*	5	0.035328208	0.02274275	0.009989819	*TMEM120A*
chr11	40315337	40315861	*	4	0.035340202	-0.034060722	-0.018763295	*LRRC4C*
chr10	125034571	125034818	*	3	0.035340202	-0.014143954	-0.011445446	*NA*
chr11	123986026	123986166	*	9	0.035343837	-0.011352545	-0.002440744	*VWA5A*
chr8	1847999	1848614	*	4	0.035344524	-0.024547229	-0.015193665	*ARHGEF10*
chr7	4245377	4245514	*	3	0.035368176	-0.013473574	-0.004427342	*SDK1*
chr1	246859889	246860416	*	5	0.035368559	0.044586377	0.021667662	*NA*
chr1	16693479	16693683	*	8	0.035392487	0.015165648	0.001653893	*SZRD1*
chr4	157891938	157892087	*	3	0.035421546	-0.005727198	-0.004321825	*PDGFC*
chr1	104068093	104068301	*	6	0.035425005	-0.010999177	-0.006744231	*RNPC3*
chr14	21539025	21539444	*	7	0.035433965	0.030940122	0.002582058	*ARHGEF40;NDRG2*
chr3	194783274	194783413	*	5	0.035433965	0.016521073	0.007168374	*NA*
chr17	34087153	34087579	*	3	0.035447454	0.014367219	0.012622133	*C17orf50;MMP28*
chr6	100016130	100016838	*	10	0.035449628	-0.010840884	-0.002355626	*CCNC;TSTD3*
chr20	62439117	62439296	*	5	0.035457325	0.030109035	0.016225549	*ZBTB46*
chr7	1216824	1216933	*	3	0.035470037	-0.052435105	-0.034609304	*NA*
chr11	1229901	1230144	*	4	0.035520074	0.017697343	0.012608602	*MUC5B*
chr1	115824285	115824630	*	3	0.035526447	0.022653339	0.011204919	*NA*
chr17	37783220	37784024	*	7	0.035590389	0.011462848	-0.000425657	*PPP1R1B*
chr6	36561383	36561606	*	3	0.035610802	0.009933741	0.001398222	*SRSF3*
chr5	140571882	140572039	*	5	0.035613518	-0.023358136	-0.010118373	*PCDHB10*
chr16	56224901	56225059	*	4	0.035632751	-0.01888388	-0.013919621	*GNAO1;LOC283856*
chr19	3720612	3721383	*	4	0.035643269	-0.026542461	-0.008969686	*TJP3*
chr11	1273661	1274140	*	4	0.035647742	0.020412121	0.012188679	*MUC5B*
chr4	76751599	76751767	*	3	0.035647742	-0.021547102	-0.005246575	*NA*
chr10	13771383	13771465	*	3	0.035707183	-0.013012982	-0.006620504	*FRMD4A*
chr12	7126735	7126979	*	3	0.035713061	0.021737195	0.001909673	*C1S;LPCAT3*
chr8	41507621	41508117	*	4	0.035714616	0.016909715	0.012721163	*NKX6-3*
chr14	54687160	54687202	*	3	0.035739903	-0.006212946	-0.004825191	*NA*
chr1	176998778	176999034	*	4	0.035764341	0.016482837	0.004461356	*ASTN1;MIR488*
chr7	152591581	152592038	*	6	0.035778056	-0.034431066	-0.003467763	*NA*
chr21	37757070	37757883	*	10	0.035781809	-0.021084523	-0.002390941	*CHAF1B*

chr9	112542443	112542779	*	4	0.035789457	0.024241895	0.001925971	*PALM2AKAP2*
chr13	45885891	45886303	*	3	0.035790202	-0.012744119	-0.008230611	*NA*
chr6	28792778	28793329	*	4	0.035798796	0.011136555	-0.000623326	*NA*
chr11	393648	394262	*	6	0.035820795	0.016299785	0.006441082	*PKP3*
chr1	78354224	78354382	*	4	0.035824109	-0.009180138	-0.006442231	*NEXN*
chr11	67007442	67007811	*	11	0.035826951	-0.020881006	-0.007891354	*KDM2A*
chr1	156828693	156829287	*	8	0.035857152	0.02269278	0.01086185	*INSRR;NTRK1*
chr8	67089388	67089599	*	3	0.035873755	-0.017883359	-0.013291496	*CRH*
chr1	3623102	3623605	*	4	0.035887896	0.019357298	0.012985115	*TP73*
chr2	231809311	231809697	*	5	0.035898325	-0.025404896	-0.001569988	*GPR55*
chr7	194861	195478	*	3	0.035898325	0.009265471	0.005453825	*FAM20C*
chr6	84569161	84569456	*	8	0.035903712	0.007470382	0.001022857	*CYB5R4*
chr11	111700061	111700634	*	3	0.035915338	0.013309487	0.003652924	*ALG9*
chr19	41304072	41304468	*	5	0.035916515	0.005267464	-0.00069074	*EGLN2;RAB4B-EGLN2*
chr11	63656088	63656342	*	4	0.035938015	-0.008913699	-0.005673842	*MARK2*
chr8	30891077	30891773	*	17	0.035944843	-0.017920558	-0.006402376	*PURG;WRN*
chr1	114522498	114522670	*	3	0.03597632	-0.01614182	-0.00747423	*OLFML3*
chr3	4534791	4535154	*	9	0.035979815	-0.014542472	-0.001624929	*ITPR1*
chr2	190526173	190526769	*	5	0.036007187	-0.005029545	-0.002880966	*ASNSD1*
chr9	112810400	112810803	*	5	0.036020503	0.010484564	0.002484217	*PALM2AKAP2*
chr12	16430768	16431562	*	5	0.036033283	-0.028784786	-0.002164935	*SLC15A5*
chr9	135465030	135465435	*	3	0.036041311	-0.017736345	-0.001740105	*BARHL1*
chr13	25745406	25745784	*	3	0.036054797	-0.010685948	-0.008277758	*AMER2*
chr11	69590282	69590332	*	4	0.036060716	-0.018873329	-0.009505968	*FGF4*
chr10	134956217	134956411	*	3	0.036096598	0.019391849	0.016555187	*NA*
chr6	136847105	136848313	*	9	0.036120983	-0.034200254	-0.009633014	*MAP7*
chr16	73092391	73093652	*	9	0.036170754	-0.014016527	0.000547918	*ZFHX3*
chr8	10966192	10966251	*	3	0.036191093	-0.016682031	-0.006129888	*XKR6*
chr9	35102784	35103350	*	6	0.03619251	0.016410327	0.003772287	*STOML2*
chr12	12491411	12491924	*	4	0.03619251	0.014832149	-0.00153236	*MANSC1*
chr6	31599751	31600351	*	8	0.036199224	0.015661094	0.000216932	*PRRC2A*
chr9	129375812	129376185	*	4	0.036206423	-0.016868553	-0.006541065	*LMX1B*
chr3	11293681	11294225	*	5	0.036274062	0.035091051	0.022274804	*HRH1*
chr10	134222053	134222581	*	5	0.036286722	-0.01941357	-0.012341209	*PWWP2B*
chr13	53419596	53419729	*	3	0.036293393	0.024050506	0.014388008	*PCDH8*
chr5	443987	444228	*	3	0.036335382	-0.023327137	0.000327619	*EXOC3;EXOC3-AS1*
chr8	41556933	41557710	*	3	0.036354938	0.022326203	0.016856625	*ANK1;NKX6-3*
chr1	236306311	236306767	*	3	0.036380464	-0.007047128	-0.000799739	*GPR137B*
chr17	77766335	77766817	*	3	0.036382756	0.018851863	0.013790929	*NA*
chr12	4274034	4274683	*	4	0.036410815	0.013720722	0.00737194	*NA*
chr1	201708419	201708558	*	4	0.036413515	-0.027654112	-0.015500358	*NAV1*
chr7	123673679	123673926	*	5	0.036428761	-0.024290402	-0.010420956	*TMEM229A*
chr6	32157150	32157230	*	3	0.036431566	-0.013239187	-0.00927794	*PBX2*
chr17	30469102	30469339	*	8	0.03645618	-0.007916241	-0.000761421	*RHOT1*
chr2	2617199	2617432	*	3	0.036460196	-0.028843588	-0.015327968	*NA*
chr14	99707721	99708104	*	3	0.036496669	-0.030147778	-0.001808633	*BCL11B*
chr19	10514734	10515211	*	3	0.036526801	0.003615476	0.002757413	*CDC37;MIR1181*
chr17	38295894	38296698	*	10	0.036541002	-0.014174909	-0.004088793	*CASC3*
chr12	108954266	108954532	*	4	0.03655175	-0.003925679	-0.001609284	*ISCU;SART3*
chr8	70404514	70405008	*	8	0.036582802	-0.023677599	-0.00397529	*SULF1*
chr5	73928997	73929069	*	3	0.036582802	0.02101799	0.013574012	*ENC1*
chr1	201476557	201476775	*	5	0.03661608	0.015177405	0.003942416	*CSRP1*
chr5	149380656	149381021	*	6	0.036616425	-0.021934305	-0.005236682	*HMGXB3;TIGD6*
chr7	141438329	141438468	*	5	0.036622252	-0.013697995	-0.0069475	*SSBP1;WEE2-AS1*
chr17	263705	263921	*	4	0.036705926	-0.007680125	-0.0050688	*C17orf97*
chr11	57176211	57176720	*	3	0.036712192	0.02480505	0.014916832	*PRG2;SLC43A3*
chr17	72667005	72667394	*	8	0.03673148	-0.028341132	-0.015959782	*RAB37*
chr19	9731901	9732542	*	8	0.03673148	-0.031782717	-0.0063766	*ZNF561;ZNF561-AS1*
chr11	64851426	64851957	*	14	0.036733674	-0.027912699	-0.003147071	*CDCA5;ZFPL1*
chr6	13574034	13574296	*	5	0.036737378	0.020065072	0.000202559	*SIRT5*
chr19	10370450	10370920	*	3	0.036778598	0.022666472	0.012842032	*MRPL4*
chr6	10882043	10882326	*	6	0.036828608	0.018241002	-0.00316279	*GCM2;SYCP2L*
chr2	157292764	157292955	*	6	0.036859061	-0.018341366	-0.010701888	*GPD2*
chr2	3699195	3699563	*	5	0.036871192	-0.034957004	-0.022503168	*NA*
chr6	160241519	160241556	*	3	0.036873313	-0.038729886	-0.036039607	*PNLDC1*
chr2	74880850	74881465	*	10	0.036878925	0.01429135	-0.00271514	*SEMA4F*

chr2	226264545	226264927	*	4	0.03688575	-0.032580819	-0.015482418	*NYAP2*
chr6	30594113	30595372	*	19	0.036895238	0.025799461	-0.001669698	*ATAT1;MRPS18B*
chr10	125617197	125618545	*	6	0.036895292	0.023764407	0.011735039	*CPXM2*
chr2	218770208	218770270	*	3	0.036905539	-0.011061681	-0.006800919	*TNS1*
chr12	57481609	57482022	*	4	0.036915666	-0.002980893	-0.00124671	*NAB2*
chr3	105085519	105086213	*	10	0.036916026	-0.014709871	-0.005137569	*ALCAM*
chr17	80255419	80256046	*	7	0.0369215	-0.03183407	-0.010485709	*NA*
chr7	98988566	98988729	*	3	0.036928208	0.018736907	0.005758331	*ARPC1B*
chr2	219866218	219866816	*	9	0.036939703	-0.030773039	-0.001867786	*LOC100129175;MIR375*
chr3	137483328	137483617	*	6	0.036942677	-0.017351803	-0.007858822	*SOX14*
chr1	59165685	59166243	*	6	0.036942677	-0.014689588	-0.004184715	*MYSM1*
chr2	28113213	28114044	*	16	0.03700742	0.013132473	0.000755084	*BABAM2;BABAM2-AS1;RBKS*
chr5	76114812	76114933	*	3	0.037037233	0.009316297	0.006753573	*F2RL1*
chr22	19691974	19692540	*	3	0.037048915	0.014397558	0.011843931	*NA*
chr1	115397374	115397616	*	8	0.03707538	0.046726639	0.020139012	*SYCP1*
chr1	49224754	49224970	*	7	0.037077132	0.016245845	0.010297357	*AGBL4;BEND5*
chr4	184679386	184679835	*	4	0.037119827	0.035123747	0.018179442	*NA*
chr17	77997198	77997453	*	3	0.0371376	0.011648113	0.003824371	*TBC1D16*
chr11	47546068	47546214	*	5	0.037162734	0.014038767	0.011668269	*CELF1*
chr17	77076092	77076219	*	3	0.037167448	-0.013392476	-0.000100689	*ENGASE*
chr12	32831002	32831428	*	3	0.037171018	0.015843407	-0.002243568	*DNM1L*
chr4	85419768	85420311	*	4	0.037191141	-0.010401692	-0.005096717	*NKX6-1*
chr17	41174035	41174642	*	7	0.037208933	0.018929597	-0.001276959	*RND2;VAT1*
chr15	59505941	59506153	*	3	0.037246599	0.030520797	0.004385428	*MYO1E*
chr3	126752841	126753478	*	4	0.037275623	0.019209509	0.015168811	*PLXNA1*
chr11	43604758	43604860	*	3	0.037275623	-0.018227704	-0.012351269	*NA*
chr6	138187482	138187594	*	3	0.037275623	0.007391444	0.004117893	*TNFAIP3;WAKMAR2*
chr8	145821975	145822119	*	3	0.037282357	-0.013933354	-0.002250844	*ARHGAP39*
chr22	23605330	23605797	*	5	0.037320259	0.025068901	0.010994312	*BCR;FBXW4P1*
chr5	137667061	137667555	*	7	0.037341403	-0.005328526	-0.002706958	*CDC25C*
chr6	166650317	166650404	*	3	0.037345403	-0.015757487	-0.013232869	*NA*
chr11	130079200	130079565	*	3	0.037345403	0.009370637	0.005586993	*ST14*
chr17	73584068	73584617	*	6	0.037383557	-0.013006204	-0.002595697	*MYO15B*
chr15	91259684	91260404	*	7	0.037389341	0.021715369	0.004377145	*BLM*
chr20	62436995	62437035	*	3	0.0374153	0.025886908	0.015391356	*ZBTB46*
chr3	126075894	126077046	*	10	0.037427278	0.011696444	-0.000579833	*KLF15*
chr11	33721903	33722598	*	9	0.037450584	0.017210358	0.004220539	*C11orf91*
chr19	58693874	58694300	*	3	0.037460613	-0.033415925	-0.013526516	*ZNF274*
chr11	129034245	129034338	*	3	0.037488827	0.02033089	0.00704237	*ARHGAP32*
chr18	55816295	55816677	*	3	0.037503481	0.026453151	0.009983188	*NEDD4L*
chr2	180137570	180137781	*	3	0.037508771	-0.016237459	-0.00982502	*NA*
chr1	201915757	201915999	*	7	0.037517414	-0.009131453	-0.004664506	*LMOD1*
chr6	962684	962950	*	3	0.037547269	0.014096747	0.003729113	*LINC01622*
chr14	51706162	51706980	*	9	0.037555068	0.017011749	-0.002805528	*TMX1*
chr14	21769105	21770255	*	5	0.037558505	0.022606631	0.000680242	*RPGRIP1*
chr3	46621556	46621936	*	5	0.03756828	0.031174649	0.015985747	*LRRC2;TDGF1*
chr19	49122488	49123013	*	15	0.037596697	-0.015111036	-0.00298286	*RPL18;SPHK2*
chr12	52888292	52888480	*	3	0.037605691	-0.021040463	-0.015662297	*KRT6A*
chr10	133929461	133929736	*	3	0.037605691	0.01579746	0.007651549	*JAKMIP3*
chr7	79081807	79082417	*	6	0.037629048	-0.01294896	-0.005810011	*MAGI2;MAGI2-AS3*
chr5	73399492	73399693	*	3	0.037634601	-0.018269208	-0.013913952	*NA*
chr1	223883793	223884247	*	3	0.037651133	0.02525319	-0.001030928	*NA*
chr17	57784442	57784854	*	9	0.037664279	0.012879185	0.002609608	*PTRH2;VMP1*
chr6	19837350	19837743	*	3	0.037673518	-0.012837762	-0.007357873	*ID4*
chr9	138090360	138090903	*	3	0.037697432	-0.030062128	-0.002770672	*NA*
chr19	58609269	58610131	*	15	0.037707912	-0.022587863	-0.005323097	*ZSCAN18*
chr16	21608352	21608593	*	5	0.037710552	-0.028991396	-0.006505551	*METTL9*
chr7	27178861	27179432	*	5	0.037719578	0.018683235	0.009887425	*HOXA-AS3*
chr11	121028592	121028969	*	4	0.03772736	0.017675171	0.01282939	*TECTA*
chr2	113032661	113033173	*	8	0.037731024	-0.010012991	4.18E-05	*ZC3H6*
chr2	218809254	218810263	*	5	0.037741637	0.016859595	0.003488647	*TNS1*
chr7	156930564	156931170	*	3	0.037741637	-0.017973461	0.003136415	*UBE3C*
chr22	44576869	44577265	*	8	0.037748668	-0.015350811	-0.00723702	*PARVG*
chr7	158243369	158243632	*	3	0.037748668	-0.037118685	-0.018443973	*PTPRN2*
chr12	104990337	104990555	*	3	0.03777779	0.015352917	0.004386307	*CHST11*
chr17	80145540	80145575	*	3	0.03778094	0.023303731	0.020251332	*CCDC57*

chr9	132020949	132021283	*	4	0.037781177	-0.021478248	-0.009301359	*NA*
chr1	245533472	245533949	*	3	0.037799367	0.01896654	-3.79E-05	*KIF26B*
chr12	72332386	72332964	*	7	0.037827046	-0.029005023	-0.007282642	*TPH2*
chr4	110481083	110481312	*	7	0.037827046	0.006390043	0.00087271	*MCUB*
chr9	12775640	12776046	*	4	0.037827046	-0.012348222	-0.005839385	*LURAP1L*
chr4	83294070	83294407	*	3	0.03784496	-0.016652366	-0.009489328	*HNRNPD*
chr5	95768016	95768418	*	3	0.037866085	-0.013561913	-0.007728808	*PCSK1*
chr19	6590801	6591287	*	6	0.037891808	-0.006200027	-0.002503492	*CD70*
chr12	51567063	51567310	*	5	0.037891808	-0.027069536	-0.00815965	*TFCP2*
chr1	8394347	8394401	*	3	0.037899246	0.021138849	0.009167905	*SLC45A1*
chr7	107220372	107220760	*	5	0.037904708	-0.013911959	-0.004222502	*BCAP29*
chr20	1873496	1873877	*	3	0.037971031	0.016850485	0.007028727	*SIRPA*
chr22	19710386	19710712	*	3	0.037971031	0.014379959	0.007165199	*SEPT5-GP1BB*
chr8	144571936	144572413	*	3	0.037994794	0.021874225	0.012995008	*ZC3H3*
chr8	28242918	28243029	*	3	0.037997441	-0.028515914	-0.012763578	*ZNF395*
chr17	5372003	5372492	*	10	0.038071405	0.024330369	0.002380154	*DHX33*
chr12	104350630	104351390	*	11	0.038076174	0.013161164	-0.002701228	*C12orf73*
chr2	71503482	71504232	*	11	0.038097065	-0.009256445	-0.003270335	*ZNF638*
chr1	41847267	41847668	*	3	0.038137311	0.037337057	0.011616336	*NA*
chr6	30951917	30952175	*	4	0.038139403	0.025727122	0.00981226	*MUC21*
chr6	28887141	28887514	*	4	0.038191039	-0.015948508	-0.008503784	*TRIM27*
chr10	133918232	133918387	*	3	0.038248814	0.022351133	0.009173367	*JAKMIP3*
chr11	67120966	67121358	*	9	0.038261242	-0.00896685	-0.000930865	*LOC100130987;POLD4*
chr17	38118571	38118787	*	4	0.038288531	0.019410427	0.011758374	*GSDMA*
chr22	44350726	44350883	*	4	0.03830103	0.025053205	0.008467782	*PNPLA3;SAMM50*
chr14	24033018	24033309	*	3	0.038305243	-0.013865317	-0.000376516	*AP1G2*
chr3	46718941	46719380	*	4	0.038305965	-0.019422834	-0.011406916	*ALS2CL*
chr3	49761723	49762132	*	3	0.038306082	-0.01288578	-0.005449991	*AMIGO3;GMPPB;IP6K1*
chr1	32403619	32404326	*	3	0.038309154	-0.010632326	0.001144596	*PTP4A2*
chr11	15672200	15672271	*	3	0.038315326	-0.02099884	-0.014180413	*NA*
chr12	1025772	1026392	*	5	0.038328205	0.015919716	0.005536547	*RAD52*
chr19	47217237	47217696	*	6	0.038331271	0.014724397	-0.000761236	*MIR320E;PRKD2*
chr10	71151778	71152020	*	3	0.038353657	0.022578426	0.01289217	*HK1*
chr5	81460188	81460226	*	3	0.038410662	-0.018685986	-0.006124744	*ATG10*
chr16	1358964	1359397	*	12	0.03842059	-0.020009376	-0.002358102	*UBE2I*
chr11	118272665	118273051	*	6	0.038427175	-0.008453142	-0.003993624	*ATP5MG;LOC100131626*
chr10	75384974	75385537	*	6	0.038438879	-0.021232721	-0.004345669	*NA*
chr21	35445161	35446012	*	11	0.038448175	-0.037000658	-0.011140844	*MRPS6;SLC5A3*
chr6	31748762	31749338	*	3	0.038488049	0.015970669	0.010902984	*VARS1;VWA7*
chr1	230468464	230468773	*	5	0.038488087	-0.035603638	-0.027382664	*PGBD5*
chr12	54379056	54379609	*	5	0.038488087	0.031336056	0.00275464	*HOXC10*
chr15	97862882	97863101	*	3	0.038495129	0.019865309	0.010577901	*NA*
chr5	157099032	157099197	*	3	0.038518307	0.027035068	0.017244717	*C5orf52;SOX30*
chr14	101005248	101005680	*	3	0.038521036	0.026342859	0.016156418	*BEGAIN*
chr17	1837588	1837848	*	6	0.038561202	0.024610415	0.014644439	*NA*
chr19	36132842	36132912	*	4	0.038638578	0.026418081	0.007184806	*ETV2*
chr6	17102310	17102678	*	6	0.038646665	-0.026781647	-0.011238093	*STMND1*
chr4	699224	699448	*	7	0.038690342	-0.037999822	-0.01259149	*PCGF3*
chr1	145027892	145028465	*	3	0.038703297	0.015662664	0.0052344	*LOC653513;NBPF20;NBPF9;PDE4DIP*
chr17	57297306	57297815	*	11	0.038716395	-0.039821005	-0.00673633	*GDPD1*
chr1	226829496	226829776	*	5	0.038775753	0.015964481	0.002927792	*ITPKB*
chr20	18359238	18359605	*	4	0.038783981	0.032139427	0.018160148	*LINC00851*
chr12	124086193	124086477	*	6	0.038800782	-0.014954188	-0.003406045	*DDX55*
chr11	77921274	77921503	*	4	0.038824655	0.019390224	0.009719956	*USP35*
chr3	29322819	29323531	*	6	0.038844472	0.015894617	-0.000966125	*RBMS3*
chr4	41867178	41867562	*	6	0.038856345	-0.01478013	-0.006258818	*NA*
chr7	1163456	1163642	*	3	0.038856345	0.023418616	0.01857478	*C7orf50*
chr1	36306804	36307244	*	4	0.038892488	0.010463369	0.002588893	*AGO4*
chr11	102979833	102980466	*	12	0.038950058	-0.017410564	-0.004446497	*DYNC2H1*
chr17	72856825	72857154	*	5	0.038950058	0.015417288	0.006693591	*GRIN2C*
chr7	149112887	149113196	*	4	0.038950058	-0.03935054	-0.024040316	*NA*
chr6	28129313	28129656	*	8	0.038957568	-0.035388616	-0.010813477	*ZNF192P1*
chr5	49962509	49962613	*	3	0.038981332	0.007568618	0.003925007	*PARP8*
chr15	58723657	58724225	*	5	0.039061997	0.041441787	0.018287401	*LIPC*
chr8	1054048	1054717	*	4	0.039061997	0.017787949	0.009299432	*NA*
chr19	40889848	40890114	*	4	0.039163876	0.027979375	0.018863529	*HIPK4*

chr7	29185545	29186501	*	13	0.039167247	-0.022519209	-0.00482653	*CHN2;CPVL*
chr19	49964960	49965263	*	4	0.039190444	0.021599269	0.008051592	*ALDH16A1*
chr5	140012660	140012769	*	3	0.039190444	-0.024425932	-0.014509571	*CD14*
chr17	57918262	57918682	*	4	0.039218677	-0.021942606	-0.014946127	*MIR21*
chr7	20256996	20257420	*	4	0.039241779	0.016040606	0.000837905	*MACC1*
chr2	122547604	122547895	*	4	0.039266466	0.010240914	0.00823578	*NA*
chr20	62220778	62221039	*	3	0.039325628	0.013590585	0.005286342	*GMEB2*
chr7	2645542	2645764	*	3	0.039332934	0.020500589	0.010747256	*IQCE*
chr11	31841768	31841980	*	3	0.039337575	0.011869435	0.000286812	*PAX6;PAX6-AS1;RCN1*
chr7	102937535	102938279	*	9	0.039341422	-0.009895447	-0.000112002	*PMPCB*
chr19	4007529	4008018	*	6	0.039344002	-0.013848697	-0.00592429	*PIAS4*
chr11	86748738	86749237	*	7	0.039351124	-0.029121253	-0.005800806	*TMEM135*
chr6	33378502	33378912	*	3	0.039364566	0.015202657	0.000378459	*PHF1*
chr1	236046939	236047034	*	3	0.039405801	-0.028809159	-0.017294654	*LYST*
chr12	53835350	53835506	*	4	0.039563598	0.016797615	0.003344634	*PCBP2;PRR13*
chr9	706712	707166	*	5	0.039567069	-0.013489416	-0.007311275	*KANK1*
chr16	27413786	27414418	*	6	0.039591567	-0.014320216	-0.009549302	*IL21R*
chr5	50673730	50674203	*	5	0.039596471	-0.01307442	-0.010131664	*ISL1;LOC642366*
chr14	50778273	50778592	*	3	0.039596471	-0.012634226	-0.005168375	*DMAC2L;L2HGDH*
chr6	10419846	10420287	*	7	0.039613794	-0.013243616	-0.002319029	*TFAP2A*
chr17	7757933	7758256	*	10	0.039711234	0.020511883	0.005671886	*CYB5D1;KDM6B;TMEM88*
chr10	134527606	134528050	*	4	0.039711234	0.011577589	0.004516511	*INPP5A*
chr20	31171386	31172652	*	4	0.03971239	-0.015726372	-0.002308273	*NOL4L;NOL4L-DT*
chr18	29769532	29770081	*	3	0.039741285	0.020810398	-0.001714792	*MEP1B*
chr2	27071246	27071332	*	3	0.039744205	-0.026226588	-0.011066498	*DPYSL5*
chr10	6214016	6214079	*	3	0.039821396	-0.01971921	-0.019139466	*PFKFB3*
chr13	37633548	37634004	*	8	0.039906648	-0.010186474	-0.00164054	*SUPT20H*
chr16	89939442	89939812	*	9	0.039932741	0.005975401	0.000929159	*TCF25*
chr17	79615552	79615861	*	4	0.039932741	0.038683516	0.018356236	*TSPAN10*
chr19	41880968	41881163	*	3	0.039963691	0.021727697	0.006762161	*BCKDHA;TMEM91*
chr3	113465791	113465925	*	3	0.040010666	-0.00893973	-0.001892508	*ATP6V1A;NAA50*
chr6	26595975	26596088	*	3	0.040019106	-0.005377779	-0.002312844	*ABT1*
chr2	223916148	223916639	*	6	0.040060517	0.028294944	0.013167442	*KCNE4*
chr13	20702922	20703121	*	3	0.040060517	0.007083221	0.005980826	*NA*
chr1	36642995	36643743	*	3	0.040081837	0.040095582	0.021100854	*MAP7D1*
chr17	18918311	18918472	*	4	0.040117413	0.027477998	0.012190034	*SLC5A10*
chr11	63754305	63754708	*	3	0.040117569	0.029910282	0.006162648	*OTUB1*
chr12	6887543	6888116	*	4	0.040124207	0.025476033	0.00291413	*LAG3*
chr19	41902940	41903381	*	8	0.040128575	-0.007975464	-0.000986202	*BCKDHA;EXOSC5*
chr17	5322267	5322660	*	5	0.040138666	-0.005561583	-0.00049406	*NUP88;RPAIN*
chr17	48614046	48614436	*	3	0.040143296	0.012438315	0.010938865	*EPN3*
chr11	27384596	27385277	*	10	0.040146709	-0.014362182	-0.003965828	*CCDC34*
chr7	91510314	91510597	*	6	0.040175094	0.045395058	0.013000924	*MTERF1*
chr2	85107426	85107751	*	3	0.040194192	-0.007942925	-0.001588155	*TRABD2A*
chr19	35485361	35485586	*	4	0.040199287	-0.016456079	-0.00819769	*GRAMD1A*
chr3	44038869	44038990	*	3	0.040205614	-0.012523197	0.002331002	*NA*
chr10	665304	665510	*	3	0.040206679	0.016842237	0.011194184	*DIP2C*
chr22	46318765	46319013	*	3	0.040211541	0.020403306	0.019103739	*WNT7B*
chr14	52327240	52327486	*	6	0.040218637	-0.008319901	-0.004446574	*GNG2*
chr21	48018608	48018981	*	4	0.040222326	0.017500652	0.009650839	*S100B*
chr8	637468	637909	*	3	0.040227828	0.096584379	0.054484963	*ERICH1*
chr20	37554840	37555079	*	6	0.040244609	-0.014255205	-0.003419193	*FAM83D*
chr2	1858740	1859146	*	4	0.040248732	-0.032409383	-0.015068402	*MYT1L*
chr11	69468789	69468991	*	4	0.040260488	0.015052316	0.007129153	*CCND1*
chr1	160312929	160313075	*	5	0.040283185	-0.018648385	-0.00912176	*COPA;NCSTN*
chr6	28891358	28891843	*	9	0.040292568	0.015481339	-0.001450351	*TRIM27*
chr5	140709986	140710237	*	5	0.040322201	-0.021997573	-0.009661406	*PCDHGA1*
chr12	99039011	99039302	*	7	0.040353822	-0.010950848	-0.002280908	*APAF1;IKBIP*
chr19	37960306	37960542	*	5	0.040366614	-0.007023787	-0.00149338	*ZNF569;ZNF570*
chr11	67811114	67811641	*	4	0.040374947	-0.014255608	-0.000106822	*TCIRG1*
chr17	12569322	12569901	*	4	0.04039271	0.016067539	0.008179753	*MYOCD*
chr1	44870888	44871634	*	10	0.040403343	0.011930355	-6.11E-05	*RNF220*
chr19	5997013	5997434	*	4	0.04042102	0.03038116	0.010628083	*LOC100128568;RFX2*
chr1	201096289	201096354	*	3	0.040437947	0.020109199	0.011456396	*NA*
chr8	41655789	41656079	*	4	0.040450096	0.030770269	0.009362779	*ANK1*
chr1	39456490	39457270	*	12	0.040464584	-0.015294779	-0.002506846	*AKIRIN1*

chr8	42249271	42249569	*	4	0.040482984	-0.010588696	-0.004707562	*VDAC3*
chr20	21492558	21492914	*	4	0.040485142	-0.016986389	0.001008235	*NKX2-2*
chr1	111991555	111991956	*	9	0.040486435	0.019177903	0.003080546	*ATP5PB;WDR77*
chr6	62996627	62996702	*	4	0.040491736	-0.011542779	-0.008428713	*KHDRBS2*
chr15	65321822	65322308	*	7	0.040496453	-0.018948145	-0.003658553	*MTFMT*
chr1	183155437	183155644	*	3	0.040505405	-0.01347395	-0.012991853	*LAMC2*
chr22	46409089	46409469	*	4	0.040529175	-0.01499206	-0.008019049	*NA*
chr11	6584886	6585323	*	5	0.040563978	0.018673836	0.008372323	*DNHD1*
chr19	860565	861071	*	3	0.040563978	0.025434989	0.018161861	*CFD*
chr16	49312033	49312560	*	3	0.040611782	-0.019548213	-0.008463418	*CBLN1*
chr19	2721800	2722300	*	3	0.040673482	0.027157126	0.00985797	*DIRAS1*
chr4	113441944	113442346	*	3	0.040673813	-0.009378528	-0.009015239	*NEUROG2*
chr2	55845619	55846091	*	5	0.040696912	0.01773513	0.003682299	*PPP4R3B*
chr22	50966077	50966123	*	3	0.040708523	0.014881232	0.011952708	*SCO2;TYMP*
chr11	66112695	66112880	*	4	0.040715573	0.014458571	0.000229755	*B4GAT1;BRMS1*
chr16	87445524	87445639	*	3	0.040749769	0.017057531	0.012868443	*ZCCHC14*
chr19	17728330	17728637	*	4	0.040769967	0.027635014	-0.001864026	*UNC13A*
chr2	177921240	177921624	*	4	0.040814456	0.017028942	0.002345527	*NA*
chr7	75676696	75676910	*	5	0.040817403	-0.006570729	-0.002396644	*MDH2;STYXL1*
chr1	31296597	31297444	*	10	0.040821168	-0.025788386	-0.005586844	*NA*
chr19	1812466	1812732	*	3	0.040829978	-0.025006806	-0.014685745	*ATP8B3*
chr12	56618626	56618648	*	3	0.040831274	-0.010078363	-0.007714287	*NABP2;RNF41*
chr9	125132413	125132716	*	4	0.040844151	0.015679974	0.002489268	*PTGS1*
chr2	98351769	98352002	*	3	0.040854961	0.020758742	0.011482405	*ZAP70*
chr4	3387281	3387341	*	3	0.040856169	0.011667964	0.005739365	*RGS12*
chr2	58655444	58655463	*	3	0.040888425	-0.011540225	-0.006817166	*NA*
chr10	3276519	3277192	*	6	0.040904676	0.015379672	-0.000822848	*NA*
chr5	138897043	138897583	*	4	0.040912777	0.015707417	0.008304342	*NA*
chr16	89283569	89283996	*	5	0.040920184	-0.007872719	-0.001670741	*ZNF778*
chr11	46402949	46403087	*	3	0.040924431	-0.038237268	-0.022782523	*MDK*
chr1	45120147	45120431	*	4	0.040935708	0.033076664	0.01878666	*TMEM53*
chr2	95992655	95992762	*	3	0.040938966	0.01896462	0.016937885	*KCNIP3*
chr7	158250469	158250978	*	3	0.040994455	-0.076269387	-0.024649367	*PTPRN2*
chr5	102455306	102455989	*	11	0.041018479	0.011181095	0.00060723	*GIN1;PPIP5K2*
chr2	85842750	85843380	*	10	0.041031987	0.007921925	-0.000494098	*C2orf68;USP39*
chr15	102029948	102030215	*	3	0.041086312	0.034442108	0.014566088	*PCSK6*
chr17	76900295	76900812	*	3	0.041110179	0.024936697	0.01434235	*CEP295NL;TIMP2*
chr16	30996377	30996564	*	5	0.041132687	-0.024046598	-0.007727973	*HSD3B7*
chr9	136019673	136020089	*	4	0.041140521	-0.007713808	-5.47E-05	*RALGDS*
chr1	2115951	2116287	*	4	0.041145213	0.009625424	0.004328719	*FAAP20;PRKCZ*
chr2	70188512	70188691	*	3	0.041167474	0.025731566	0.015187526	*ASPRV1*
chr14	53258566	53258684	*	4	0.041172792	0.021437738	0.013580207	*GNPNAT1*
chr1	36235406	36235882	*	14	0.041211207	-0.01045679	-0.000409469	*CLSPN*
chr11	66314196	66314378	*	8	0.041215386	0.039332099	0.007697353	*ACTN3;ZDHHC24*
chr17	42988784	42989323	*	6	0.041216212	0.022891943	0.008720752	*GFAP*
chr16	3493423	3494094	*	12	0.041269157	0.020275518	-0.001087532	*NAA60;ZNF597*
chr6	7728888	7729080	*	4	0.041271635	-0.015736834	-0.004970426	*BMP6*
chr10	131361472	131361540	*	3	0.041277984	0.028750829	0.001968075	*MGMT*
chr9	107689793	107689848	*	3	0.041357352	0.006506877	-0.000886824	*ABCA1*
chr15	85258669	85258977	*	3	0.041395976	-0.005387759	-0.000273794	*SEC11A*
chr19	436541	437061	*	3	0.041395976	0.025184293	0.013251327	*SHC2*
chr19	3006438	3006720	*	3	0.04142955	0.037462663	0.023649914	*TLE2*
chr2	11273075	11273291	*	4	0.041449694	-0.009960204	-0.005145308	*C2orf50;FLJ33534*
chr2	159312599	159312727	*	3	0.041449694	-0.021280463	-0.015232812	*CCDC148;PKP4*
chr22	46647235	46647620	*	3	0.04145726	0.022367339	0.013994054	*CDPF1*
chr5	179588440	179588838	*	3	0.041482412	-0.036176918	-0.03253321	*RASGEF1C*
chr7	70597599	70598282	*	6	0.041489192	-0.016881235	-0.007074313	*GALNT17*
chr14	102172004	102172296	*	3	0.041489192	-0.023815783	0.001682194	*NA*
chr1	54665230	54665660	*	5	0.041504109	-0.003194599	-0.001542557	*CYB5RL;MRPL37*
chr11	1091487	1092321	*	5	0.041504109	0.018190014	0.000572416	*MUC2*
chr3	181441384	181441680	*	4	0.041515898	-0.011919218	-0.010016338	*SOX2-OT*
chr19	19569139	19569574	*	4	0.041530609	-0.024361446	-0.008947769	*GATAD2A*
chr16	83841105	83841755	*	10	0.041545661	-0.009386465	-0.000109075	*HSBP1*
chr6	27279917	27280423	*	9	0.041559751	0.014241379	0.000978037	*POM121L2*
chr6	31707151	31707667	*	17	0.041607087	0.01715528	0.000229773	*CLIC1;MSH5-SAPCD1*
chr7	6629350	6629457	*	3	0.041621699	0.010345171	0.002122513	*C7orf26*

chr6	133137810	133137893	*	3	0.041624244	0.017109199	0.008679424	*RPS12;SNORA33*
chr7	25893749	25894005	*	4	0.041643125	0.022919893	0.016213204	*NA*
chr2	39348512	39348582	*	3	0.041643125	0.005990127	0.00121524	*SOS1*
chr1	88928362	88928839	*	5	0.04165317	-0.024997766	0.000387812	*NA*
chr11	64793631	64793846	*	3	0.04167798	0.018787036	0.006040619	*ARL2-SNX15*
chr10	85761623	85761783	*	3	0.041685483	0.016758717	0.004624386	*NA*
chr11	115631143	115631452	*	4	0.04169008	0.013109574	0.002093735	*LINC00900*
chr3	11685307	11685606	*	6	0.041712901	-0.018122168	-0.008188536	*VGLL4*
chr4	6989536	6990245	*	3	0.041747371	0.012233906	0.002766998	*TBC1D14*
chr3	9291465	9291773	*	4	0.041768849	-0.014461571	-0.008785167	*SRGAP3*
chr19	41769929	41770192	*	9	0.041781741	-0.005697024	-0.000924027	*HNRNPUL1*
chr16	58914220	58914345	*	3	0.041785343	-0.023892277	-0.011599462	*NA*
chr4	184242831	184243024	*	4	0.041806986	0.027076543	0.006207725	*CLDN22;CLDN24*
chr1	2179352	2179933	*	3	0.041849855	0.014526116	0.007432149	*SKI*
chr2	9778653	9778812	*	3	0.041851266	0.018938343	0.00162913	*NA*
chr12	12715028	12715628	*	7	0.04186508	-0.008946312	-0.001474828	*DUSP16*
chr19	24097411	24097753	*	3	0.041891732	0.015266094	0.013612747	*ZNF726*
chr14	103989189	103989629	*	9	0.041894254	-0.011899836	-0.001556479	*CKB*
chr20	57765943	57766332	*	3	0.041894254	0.01317714	0.000649817	*ZNF831*
chr11	102496104	102496159	*	3	0.041908692	0.02132405	0.014585898	*MMP20*
chr2	172377981	172378036	*	3	0.041918479	0.021950104	0.005889092	*CYBRD1*
chr6	31047558	31047822	*	9	0.041926273	-0.013126144	-0.004011486	*NA*
chr6	32015635	32015773	*	5	0.041946871	0.020158358	0.006653808	*TNXB*
chr8	143535437	143535580	*	3	0.041967091	0.02595624	0.016335482	*NA*
chr1	222560672	222560921	*	4	0.041978039	0.02400142	-0.000527389	*NA*
chr4	185395852	185396180	*	9	0.041995069	0.005206899	0.001431179	*IRF2*
chr1	1663924	1664080	*	3	0.042008337	0.031583777	0.012496197	*SLC35E2A;SLC35E2B*
chr11	77899565	77900088	*	11	0.042029136	0.007373447	0.000501401	*KCTD21;USP35*
chr2	45168507	45168924	*	6	0.042046982	-0.005001618	-0.003147131	*SIX3*
chr4	141177756	141178298	*	3	0.04208711	0.01906357	0.010037399	*SCOC*
chr11	115044050	115044454	*	4	0.042089569	-0.027613396	-0.001684864	*CADM1*
chr16	68013968	68014374	*	5	0.042122677	0.031342434	0.02245363	*DPEP3*
chr8	144510753	144510966	*	3	0.042122677	0.025810035	0.004355851	*MAFA*
chr2	240142589	240142806	*	3	0.042217915	-0.026100789	-0.021806123	*HDAC4*
chr5	176295797	176295891	*	3	0.042234176	0.015854912	0.009009472	*UNC5A*
chr14	72703540	72703626	*	3	0.042378602	0.025053495	0.007206478	*RGS6*
chr1	212965581	212965701	*	3	0.042437091	-0.016550729	-0.005100965	*NSL1;TATDN3*
chr16	1764111	1764432	*	3	0.042437983	0.02652585	0.016808397	*MAPK8IP3*
chr5	86564371	86564790	*	9	0.042460348	0.00837339	-0.000963795	*RASA1*
chr15	52310788	52311136	*	4	0.042474701	-0.009083047	-0.005542963	*MAPK6*
chr14	106833861	106834320	*	3	0.04253249	-0.016469101	-0.003554149	*NA*
chr1	990293	990557	*	5	0.042583527	0.017855617	0.012231823	*AGRN*
chr1	112282121	112282390	*	8	0.042624736	0.007071493	-0.000183397	*INKA2;INKA2-AS1*
chr11	58949291	58949463	*	4	0.042630226	0.021080839	0.009422681	*DTX4*
chr19	6135505	6135519	*	3	0.042637732	0.017847294	0.009215385	*ACSBG2*
chr6	165241087	165241210	*	3	0.042658769	0.022215071	0.008672972	*NA*
chr14	23066421	23067088	*	6	0.042679942	-0.007847255	-0.003030314	*ABHD4*
chr20	48099146	48099479	*	6	0.04269719	0.014199592	0.004089447	*KCNB1*
chr20	48732460	48733074	*	9	0.042716221	-0.038101031	-0.009722309	*PEDS1-UBE2V1;UBE2V1*
chr16	85214676	85215140	*	3	0.042716221	0.020875426	0.014618721	*NA*
chr1	58525572	58525635	*	3	0.042772002	-0.025630439	-0.017949296	*DAB1*
chr17	38497494	38497627	*	3	0.042780246	-0.005697483	-0.000624229	*RARA*
chr3	156273277	156273486	*	5	0.042782121	-0.037016287	-0.022369535	*SSR3*
chr17	78755379	78755442	*	3	0.042782121	0.027551031	0.016867561	*RPTOR*
chr6	909612	909903	*	4	0.042786478	0.014488601	0.010632945	*NA*
chr3	42577680	42577884	*	4	0.042813599	0.023623788	0.010943401	*VIPR1*
chr16	22959819	22959875	*	3	0.042842909	-0.038175027	-0.024065377	*NA*
chr7	23053549	23053912	*	7	0.04290336	-0.029662174	-0.003308483	*FAM126A*
chr12	108732994	108733716	*	9	0.042933122	-0.013288739	-0.008001033	*CMKLR1*
chr19	50528391	50529485	*	16	0.042969808	-0.008769603	-0.002389184	*VRK3;ZNF473*
chr12	131590393	131590596	*	3	0.042989294	-0.016361523	-0.013407779	*ADGRD1*
chr5	180615482	180615650	*	3	0.043008398	0.016250678	0.003321958	*NA*
chr5	133860141	133860686	*	4	0.043018968	0.008680082	0.001429704	*JADE2*
chr2	10972607	10973007	*	6	0.043020044	-0.010358703	-0.006039865	*PDIA6*
chr14	89028798	89029076	*	4	0.043067434	0.018491835	0.005427555	*ZC3H14*
chr2	223521309	223521683	*	3	0.043075648	0.02172299	0.010606362	*FARSB*

chr6	30079090	30079280	*	8	0.043126389	-0.045817056	-0.02096631	*TRIM31*
chr9	137298339	137298390	*	4	0.043126929	0.022452009	0.010915654	*RXRA*
chr10	118900324	118900464	*	3	0.043222789	0.02437157	0.008628052	*VAX1*
chr5	11903441	11903681	*	4	0.043243943	-0.010302292	-0.004473938	*CTNND2*
chr2	179387456	179388064	*	4	0.043249622	-0.03307654	-0.017869025	*MIR548N;TTN-AS1*
chr3	11732893	11733395	*	3	0.043279234	-0.010223582	-0.003738523	*VGLL4*
chr9	99540324	99540647	*	9	0.04329621	0.009705591	0.002301218	*ZNF510*
chr5	43602380	43602519	*	3	0.04330046	0.008798647	0.002986251	*NNT;NNT-AS1*
chr2	10588825	10589054	*	5	0.043356672	0.005262304	0.001225021	*ODC1;SNORA80B*
chr7	142027996	142028043	*	3	0.043378242	-0.015674481	-0.013983083	*NA*
chr1	55247140	55247408	*	3	0.043381549	0.034412773	0.022580304	*TTC22*
chr20	31037186	31037515	*	3	0.043409175	-0.010783795	-0.007375906	*NOL4L*
chr19	54290523	54290913	*	7	0.043419506	-0.02876966	-0.004348023	*MIR371B;MIR372;MIR373*
chr16	58425569	58425711	*	3	0.043469577	0.012673051	0.004102397	*GINS3*
chr14	67879101	67879385	*	5	0.04347015	0.040588798	0.018190306	*PLEK2*
chr3	49936552	49936623	*	3	0.043488197	0.015755904	0.011855622	*MST1R*
chr19	49956333	49956506	*	5	0.043491957	0.014626205	0.004398734	*ALDH16A1;PIH1D1*
chr2	74710205	74710667	*	13	0.043495348	0.011262059	-0.000411231	*CCDC142;TTC31*
chr16	1222028	1222626	*	4	0.043495348	0.016982683	0.010302722	*CACNA1H*
chr3	196750158	196750506	*	3	0.043577086	-0.028762981	-0.022721881	*MELTF*
chr11	622021	622229	*	3	0.043577086	0.024133197	0.014702073	*CDHR5*
chr2	75836347	75836410	*	3	0.043618452	-0.017485309	-0.011963938	*NA*
chr1	2159351	2159373	*	4	0.043620504	-0.008652506	-0.002850911	*SKI*
chr10	30024742	30024894	*	4	0.043635886	-0.019146389	-0.008617626	*SVIL*
chr16	79634410	79634708	*	4	0.043653269	-0.00308436	-0.001525782	*MAF*
chr18	44237619	44237801	*	3	0.043676597	0.024265939	0.016091964	*LOXHD1*
chr2	182522257	182522425	*	3	0.04368353	0.029722219	0.01822126	*CERKL*
chr7	158319029	158319204	*	3	0.043688735	0.025071634	0.019768536	*PTPRN2*
chr14	23775663	23775794	*	5	0.043699773	-0.014670932	-0.008188166	*BCL2L2;BCL2L2-PABPN1;PPP1R3E*
chr3	33700962	33701166	*	4	0.043702487	-0.020032011	-0.014131117	*CLASP2*
chr19	4305072	4305239	*	3	0.043764542	0.027821069	0.025900214	*FSD1;TMIGD2*
chr4	8043170	8043258	*	3	0.043766734	0.029845775	0.018199987	*ABLIM2*
chr1	3191660	3191876	*	5	0.043777541	0.015692666	0.003474362	*PRDM16*
chr17	4074126	4074299	*	4	0.043777541	0.025293554	0.010687482	*ANKFY1*
chr6	14755099	14755359	*	3	0.043777945	0.025942623	0.012312201	*NA*
chr14	76127290	76127843	*	10	0.043785603	-0.015958958	-0.002896009	*ERG28;TTLL5*
chr1	16062894	16063122	*	3	0.043792693	0.01567695	0.004315308	*SLC25A34*
chr8	85094842	85095275	*	3	0.043821947	-0.003629145	-0.002697073	*RALYL*
chr12	57881178	57881799	*	6	0.04383851	0.013372614	0.002258447	*ARHGAP9;MARS1*
chr19	1490414	1490631	*	6	0.043864165	-0.010817291	-0.000209574	*PCSK4;REEP6*
chr19	55580852	55581012	*	4	0.043864165	-0.014497617	-0.005560279	*EPS8L1;RDH13*
chr11	72065390	72065489	*	3	0.043898668	0.021109908	0.013864455	*CLPB*
chr16	90114063	90114307	*	6	0.043940868	-0.030203047	-0.019578943	*URAHP*
chr3	148721527	148721868	*	3	0.043955117	0.037478476	0.027590758	*GYG1*
chr2	207139066	207139471	*	12	0.043987662	0.013397998	0.001819055	*ZDBF2*
chr19	3135131	3135364	*	3	0.04401741	0.011762193	0.000972259	*GNA15*
chr3	185407094	185407276	*	4	0.044039318	0.020072392	0.009648987	*IGF2BP2*
chr3	128273476	128273509	*	3	0.044048624	0.021336479	0.015330876	*NA*
chr11	74109674	74110145	*	6	0.044088378	0.021578988	0.002494614	*PGM2L1*
chr12	133417190	133417338	*	4	0.044115211	0.038846113	0.024621417	*CHFR*
chr9	2844149	2844396	*	6	0.044146852	-0.004827723	-0.002747422	*PUM3*
chr11	116706051	116706562	*	6	0.044204078	-0.029200083	-0.005716449	*APOA1*
chr1	156261200	156261403	*	6	0.044228653	0.033071614	0.012296068	*TMEM79*
chr22	41613692	41614127	*	4	0.044246489	-0.017535671	-0.011104743	*L3MBTL2*
chr12	53694011	53694132	*	4	0.044311482	-0.022752692	-0.002169381	*MYG1*
chr17	38804673	38805157	*	7	0.044317708	0.019870778	0.003969711	*SMARCE1*
chr1	203255843	203256315	*	5	0.044320227	-0.011430603	-0.007007755	*NA*
chr10	111682910	111683347	*	6	0.044342082	-0.005349437	-0.002668773	*XPNPEP1*
chr6	116989951	116990951	*	7	0.044343666	0.023223734	0.006107622	*ZUP1*
chr1	151805634	151805773	*	3	0.044364025	0.018604289	0.000777726	*RORC*
chr16	66876847	66876938	*	3	0.044368638	0.022929489	0.018323188	*CA7*
chr11	3400108	3400543	*	6	0.044410675	-0.011702953	-0.00014541	*TSSC2;ZNF195*
chr9	130829748	130830096	*	4	0.044445933	-0.0048728	-0.001093519	*NAIF1;SLC25A25*
chr19	19774756	19774961	*	4	0.044469767	-0.012842602	-0.004952224	*ATP13A1;ZNF101*
chr12	103351855	103352000	*	3	0.044476933	-0.010464664	-0.004832686	*ASCL1*
chr11	65337543	65338038	*	7	0.044508614	-0.00601561	-0.0009684	*FAM89B;ZNRD2;ZNRD2-AS1*

chr4	57976431	57976561	*	3	0.044585957	-0.016858064	-0.011900816	*IGFBP7;IGFBP7-AS1*
chr20	60119508	60119664	*	3	0.044600433	0.028418698	0.014240703	*CDH4*
chr2	24240148	24240246	*	3	0.044618356	0.016529835	0.011818516	*MFSD2B*
chr11	45737711	45737771	*	3	0.044636913	0.023795381	0.022739922	*NA*
chr17	47492263	47492548	*	10	0.044676009	-0.032829472	-0.009996867	*PHB*
chr13	95248638	95248771	*	5	0.04468196	0.014238632	0.002357735	*TGDS*
chr11	48001761	48001954	*	4	0.044690047	-0.03402126	-0.00441092	*PTPRJ*
chr6	30698584	30698843	*	8	0.044700407	-0.029691403	-0.010700956	*FLOT1*
chr12	107974139	107974453	*	7	0.044700407	0.03587105	0.015284991	*BTBD11*
chr12	46660255	46660338	*	3	0.044700407	0.029314029	0.016243091	*SLC38A1*
chr5	76787720	76788595	*	11	0.044734744	-0.024761531	-0.00529113	*WDR41*
chr5	79551031	79551238	*	3	0.044768694	-0.006579534	-0.004069444	*SERINC5*
chr2	240505261	240505399	*	3	0.04477554	0.018262707	0.012489381	*NA*
chr7	116859066	116859594	*	3	0.044781265	0.019032006	0.006269847	*ST7*
chr1	111307148	111307245	*	3	0.044830761	0.030601205	0.01705927	*NA*
chr2	8818884	8819227	*	6	0.04483109	0.012766449	0.005044818	*ID2*
chr11	57078976	57079362	*	5	0.044842583	-0.01406064	-0.007200272	*TNKS1BP1*
chr14	70233693	70233918	*	5	0.044848303	-0.010506517	-0.001595659	*LOC100289511;SRSF5*
chr19	40732784	40732902	*	4	0.044848303	0.022375255	0.004090995	*CCNP*
chr19	39616041	39616465	*	9	0.044905955	-0.011426244	-0.002822803	*PAK4*
chr3	121468645	121468984	*	8	0.04494649	-0.022491428	-0.005439506	*GOLGB1*
chr1	109585022	109585161	*	4	0.044952625	-0.011200108	-0.004195452	*WDR47*
chr20	23077706	23077908	*	3	0.044953601	-0.015148027	-0.004838594	*NA*
chr3	38663937	38664174	*	3	0.044953601	0.017049272	0.007529966	*SCN5A*
chr3	33839997	33840313	*	8	0.04495473	-0.006994336	-0.000472691	*PDCD6IP*
chr17	41909601	41909716	*	4	0.04496648	0.01727926	0.010539829	*MPP3*
chr20	30865285	30865674	*	8	0.044979022	-0.003930215	-0.001627227	*KIF3B*
chr20	8638980	8639258	*	4	0.04499396	-0.018326671	-0.000507752	*PLCB1*
chr17	64575563	64575592	*	3	0.04499396	-0.013334678	-0.01165192	*PRKCA*
chr10	134943246	134943309	*	3	0.045005326	-0.042040222	-0.021650158	*ADGRA1*
chr6	170408355	170408669	*	3	0.04500588	-0.01776162	0.00218355	*NA*
chr12	108079012	108079950	*	14	0.045038401	-0.009439431	-0.001383987	*PWP1*
chr2	200189694	200189929	*	3	0.045078407	0.018738996	0.004083941	*SATB2*
chr1	31190863	31191003	*	4	0.045112079	-0.020580001	-0.010850925	*MATN1;MATN1-AS1*
chr18	47808805	47809075	*	3	0.045121269	0.025576605	0.015439535	*CXXC1;MBD1*
chr11	8285724	8286017	*	3	0.045136028	-0.015183458	-0.00541699	*LMO1*
chr6	57181701	57181802	*	3	0.045137188	-0.023675625	-0.007202224	*PRIM2*
chr3	125677491	125677724	*	3	0.045192411	0.032534752	0.026372597	*NA*
chr1	228112806	228113207	*	5	0.04522103	0.022957627	0.016713922	*WNT9A*
chr8	22500401	22500482	*	3	0.045228473	-0.017488014	-0.012115232	*BIN3;BIN3-IT1*
chr19	16284242	16284421	*	5	0.045248261	0.019813442	0.003196952	*CIB3*
chr19	13207239	13207602	*	4	0.045296362	0.015601322	0.001284153	*NFIX*
chr1	61548115	61548309	*	5	0.045298664	-0.015075982	-0.002928877	*NFIA*
chr13	112733670	112733739	*	3	0.0454913	-0.031083442	-0.021470204	*NA*
chr5	173488447	173488460	*	3	0.045513658	0.029614922	0.020603133	*NSG2*
chr8	142377303	142378116	*	10	0.045517356	-0.016658181	-0.006511329	*GPR20*
chr6	31940522	31940855	*	10	0.045538851	-0.022299826	-0.004990818	*DXO;STK19*
chr3	193808583	193808625	*	3	0.045544518	0.014060555	0.00911988	*NA*
chr2	45150055	45150218	*	3	0.04564963	-0.023104932	-0.000960485	*NA*
chr6	126660245	126660315	*	3	0.04570955	0.014166291	0.00619049	*CENPW*
chr11	71163853	71164189	*	5	0.045738953	-0.007037004	-0.003470826	*DHCR7;NADSYN1*
chr20	30619244	30619522	*	4	0.045750274	-0.010561129	-0.003905951	*CCM2L*
chr3	171528273	171528828	*	5	0.045751478	0.024159774	-0.001037799	*PLD1*
chr19	2650727	2650951	*	5	0.045774234	0.025150471	0.011277568	*GNG7*
chr3	194795378	194795573	*	3	0.04578152	0.018236379	0.012811796	*XXYLT1*
chr13	41345183	41345689	*	12	0.045838374	-0.004701264	-0.000340756	*MRPS31*
chr1	174967663	174968015	*	3	0.045843116	0.020127593	0.013229388	*CACYBP*
chr19	41836950	41837123	*	3	0.045868437	0.020626779	0.016316419	*TGFB1*
chr2	98329942	98330493	*	4	0.045878107	-0.010799347	-0.007278064	*ZAP70*
chr19	52005153	52005188	*	3	0.045944023	-0.012102958	-0.005880624	*SIGLEC12*
chr19	35992791	35992835	*	3	0.045969096	0.025810858	0.018952163	*DMKN*
chr15	75119018	75119335	*	3	0.046017094	-0.025921405	-0.017170704	*CPLX3;LMAN1L*
chr3	116164242	116164576	*	5	0.046031971	-0.01860319	-0.009465541	*LSAMP*
chr16	89977652	89977815	*	3	0.04605974	0.008450682	0.006361185	*TCF25*
chr20	57226061	57226202	*	4	0.046097128	-0.009944485	-0.003550436	*STX16*
chr1	876249	876551	*	5	0.046113833	0.017547941	0.004545351	*SAMD11*

chr19	46389466	46389607	*	7	0.046170634	0.005170721	0.001499673	*IRF2BP1*
chr6	31865084	31865118	*	4	0.046188504	-0.011155955	-0.00388585	*C2;EHMT2*
chr12	56660626	56660921	*	5	0.046244563	-0.006795771	-0.001141829	*COQ10A*
chr17	9548019	9548120	*	3	0.046244563	-0.00542131	-0.002697652	*USP43*
chr11	1380305	1380355	*	3	0.046296372	0.013517353	0.007899203	*NA*
chr14	105750359	105750676	*	3	0.046318823	-0.014889103	-0.01026285	*BRF1*
chr17	47270388	47270556	*	3	0.046327705	-0.005296212	-0.002942239	*NA*
chr9	102668802	102669115	*	6	0.046327824	-0.013950474	0.000109564	*STX17;STX17-AS1*
chr15	41708908	41709156	*	6	0.046332613	0.009950184	-0.00029168	*RTF1*
chr6	30313517	30313742	*	3	0.046337778	-0.013134659	-0.01097087	*RPP21;TRIM39-RPP21*
chr19	50250529	50250595	*	3	0.04638561	0.016295109	0.011212988	*TSKS*
chr17	10741601	10742003	*	4	0.046442279	-0.010555503	-0.006502292	*PIRT*
chr1	197744516	197744787	*	8	0.046473181	0.017840913	0.000751056	*DENND1B*
chr18	31158234	31158549	*	7	0.046478114	-0.019520779	-0.006976343	*ASXL3*
chr6	163612896	163613008	*	3	0.04647853	-0.021749212	-0.012730399	*PACRG*
chr10	43277745	43277913	*	5	0.046487214	-0.019980149	-0.001330764	*BMS1*
chr14	77786981	77787055	*	4	0.046607721	-0.015302482	-0.007917654	*GSTZ1;POMT2*
chr8	48744522	48744602	*	3	0.04664893	0.022297792	0.013118874	*PRKDC*
chr6	33160869	33161148	*	8	0.046682272	0.014769325	-0.000363257	*COL11A2*
chr4	3449663	3449904	*	3	0.046706383	0.018601872	0.012791019	*HGFAC*
chr17	72580567	72581014	*	4	0.046767268	0.021826724	0.012989341	*C17orf77;CD300LD*
chr15	42387193	42387290	*	3	0.046814533	0.019089914	0.007890921	*PLA2G4D*
chr6	30293841	30294255	*	13	0.046876019	-0.012222471	-0.001973708	*HCG17;HCG18;TRIM39;TRIM39-RPP21*
chr21	43735412	43735760	*	5	0.046954058	0.016939391	0.001956454	*TFF3*
chr13	36045352	36045633	*	4	0.046967308	0.025024963	0.009979487	*NBEA*
chr13	111090669	111090821	*	3	0.047044244	0.019600135	0.008288627	*COL4A2*
chr11	2165425	2165656	*	3	0.047106171	-0.014381142	-0.009597226	*IGF2;IGF2-AS;INS-IGF2*
chr12	7260546	7260877	*	6	0.04716395	0.016335342	0.001973679	*C1RL;C1RL-AS1*
chr17	1090809	1090905	*	3	0.047238822	0.012129026	0.007197664	*ABR*
chr19	39421606	39421969	*	7	0.047249748	-0.01580336	-0.006666233	*MRPS12;SARS2*
chr7	28220498	28220533	*	4	0.047259122	0.005293812	0.0026241	*JAZF1;JAZF1-AS1*
chr3	197239184	197239698	*	3	0.047365669	0.018721289	0.009296704	*BDH1*
chr5	145317340	145317425	*	3	0.047407063	0.042580091	0.015585704	*SH3RF2*
chr9	117150236	117150495	*	4	0.04741385	-0.012196253	-0.009146297	*AKNA*
chr14	58765243	58765609	*	3	0.047458822	-0.006853802	-0.003050376	*ARID4A;ARMH4;PSMA3-AS1*
chr8	1304855	1305125	*	3	0.047503286	0.014627665	-0.001269905	*NA*
chr5	133305347	133305704	*	4	0.04758472	0.030679527	0.01854528	*C5orf15*
chr15	79383385	79383644	*	6	0.047806731	0.010973043	0.001307268	*RASGRF1*
chr21	45079144	45079336	*	6	0.048004176	0.016193996	0.000366877	*HSF2BP;RRP1B*
chr14	105452552	105452712	*	4	0.048006978	0.010430482	-0.000149178	*CLBA1*
chr17	78925172	78925278	*	3	0.048010974	0.012873287	0.006965867	*RPTOR*
chr7	149559370	149559471	*	3	0.048040963	0.012573554	0.007299955	*ZNF862*
chr6	36842801	36842865	*	3	0.048048273	-0.004521313	-3.09E-05	*C6orf89;PPIL1*
chr11	62192276	62192381	*	3	0.048071178	-0.023327387	-0.016754082	*NA*
chr17	40995321	40995583	*	3	0.0480717	-0.01912736	-0.008146057	*AOC2;PSME3*
chr16	10837597	10837677	*	5	0.048146546	-0.015367427	-0.012022918	*NUBP1*
chr11	1848691	1848742	*	3	0.048167515	0.018477096	0.013817109	*SYT8*
chr1	2518104	2518194	*	4	0.04829853	-0.025373989	-0.016307391	*PRXL2B*
chr20	62680986	62681064	*	5	0.048398386	-0.03876124	-0.026460766	*SOX18*
chr19	45150513	45150725	*	4	0.048435053	0.023526003	0.01589017	*PVR*
chr11	915093	915440	*	8	0.048490661	-0.02067467	-0.001161107	*CHID1*
chr21	27945413	27945542	*	3	0.048492151	-0.023779446	-0.015630847	*CYYR1*
chr12	103358649	103358708	*	3	0.048674044	-0.0094215	-0.006918751	*NA*
chr1	156265275	156265456	*	4	0.048777936	-0.01455783	-0.009034689	*GLMP*
chr5	132202169	132202281	*	4	0.048829896	0.0054486	-2.32E-05	*GDF9;UQCRQ*
chr6	33422516	33422529	*	3	0.048897806	-0.020785648	-0.004329101	*ZBTB9*
chr6	42749794	42749811	*	3	0.04901293	-0.022623761	-0.008332268	*BICRAL*
chr16	1265487	1265606	*	3	0.049085364	0.028088424	0.007409778	*CACNA1H*
chr20	61754869	61755045	*	3	0.049107194	0.037717139	0.019239472	*NA*
chr4	42659286	42659458	*	5	0.049118333	-0.005976617	-0.001070897	*ATP8A1*
chr6	85474188	85474313	*	3	0.049235898	-0.008027972	-0.00461179	*TBX18*
chr3	61547104	61547211	*	5	0.049285746	-0.016616711	-0.005991205	*PTPRG*
chr5	80256738	80256867	*	3	0.049312579	0.015568483	0.008956073	*RASGRF2*
chr17	73569211	73569370	*	3	0.049438397	0.026175562	0.013594608	*LLGL2*
chr19	41869688	41869719	*	3	0.049487122	0.010985649	0.00158734	*B9D2;TMEM91*

Supplemental Table 5 - Lifestyle specific KEGG pathway enrichment analysis

ID	Description	Gene ratio	Bg ratio	*P*-value	FDR	*q*-value	Gene ID	Count	Fold enrichment
hsa04724	Glutamatergic synapse	43/1682	114/8105	2.12E-05	0.00691	0.00564	CACNA1D/GNG7/ADCY7/GRIN2A/SHANK2/ITPR1/GRIK4/PRKCA/GRIK1/GRM3/HOMER3/GRIK5/SLC38A1/PLA2G4D/GRM2/PLCB1/GRIN2D/GNAQ/GNG11/GRM6/GNAO1/GRIN2C/GRM1/GRK3/SLC38A2/GNG2/SLC1A7/JMJD7-PLA2G4B/GNG12/ADCY1/GNG13/DLGAP1/GRIN2B/PLD1/SLC1A6/GLUL/PRKCG/GRIK2/GRM4/ITPR3/PLCB3/GRIA4/GNAS	43	1.817567849
hsa04360	Axon guidance	58/1682	182/8105	0.00025135	0.03105	0.02536	SEMA7A/SEMA4B/BMP7/WNT5B/PLCG1/EPHB4/PLCG2/RGMA/PLXNA1/SEMA4C/PLXNB2/DPYSL2/PRKCA/MYL5/RAC2/SEMA4A/EFNA2/SEMA5B/SRGAP3/PIK3R1/RGS3/PARD6G/PAK1/DPYSL5/SEMA4G/LRRC4C/ABLIM2/SEMA6C/ROBO3/PIK3CB/UNC5A/PRKCZ/RHOD/EPHB2/UNC5C/NCK2/SEMA3F/LIMK1/SEMA3D/MET/BOC/SEMA6D/EFNA1/CAMK2D/TRPC3/WNT5A/MYL12B/NFATC2/NTNG2/ROBO1/SEMA4F/SEMA3A/EPHB3/RASA1/EPHA5/PAK4/SLIT2/KRAS	58	1.535619553
hsa05226	Gastric cancer	49/1682	149/8105	0.00032806	0.03105	0.02536	WNT5B/TGFB2/AXIN1/FGF5/AKT3/CTNNA2/SOS1/WNT7B/SHC2/DVL1/TGFB1/FGFR2/SMAD3/PIK3R1/WNT11/FGF4/CCND1/FGF9/JUP/TCF7L2/APC2/LRP5/PIK3CB/WNT1/FGF3/MUC2/RXRA/CDKN1B/SMAD2/WNT9A/MET/HGF/TERT/WNT5A/WNT2B/POLK/GADD45A/TCF7/SHC4/FGF17/FGF23/GRB2/RARB/RXRG/CDKN1A/APC/ABCB1/KRAS/RXRB	49	1.584662714
hsa04550	Signaling pathways regulating pluripotency of stem cells	47/1682	143/8105	0.00043666	0.03105	0.02536	WNT5B/IGF1R/KLF4/AXIN1/MYF5/AKT3/FGFR3/WNT7B/PCGF3/INHBA/DVL1/FGFR2/SMAD3/PIK3R1/WNT11/PCGF1/ZFHX3/DLX5/BMP4/JARID2/APC2/SMAD1/PAX6/PIK3CB/WNT1/OTX1/SMAD2/MEIS1/WNT9A/ACVR1/FGFR1/ISL1/INHBB/ID4/WNT5A/ACVR1B/WNT2B/REST/ID2/TCF7/BMI1/COMMD3-BMI1/GRB2/PCGF5/APC/KRAS/MAPK14	47	1.583758097
hsa05225	Hepatocellular carcinoma	53/1682	168/8105	0.00059792	0.03105	0.02536	WNT5B/PLCG1/IGF1R/TGFB2/PLCG2/AXIN1/AKT3/PHF10/IGF2/SOS1/PRKCA/BCL2L1/WNT7B/SHC2/DVL1/TGFB1/SMAD3/DPF1/PIK3R1/CDK6/WNT11/CCND1/TCF7L2/APC2/LRP5/PIK3CB/WNT1/SMAD2/DPF3/SMARCA4/WNT9A/MET/HGF/TERT/ARID1B/GSTM3/KEAP1/WNT5A/WNT2B/POLK/CDKN2A/GADD45A/TCF7/SHC4/NFE2L2/SMARCE1/PRKCG/GRB2/TXNRD2/CDKN1A/APC/KRAS/CDK4	53	1.520175103

hsa05202	Transcriptional misregulation in cancer	59/1682	192/8105	0.00064011	0.03105	0.02536	PBX3/CEBPE/ETV6/IGF1R/PTCRA/PPARG/ASPSCR1/RARA/BCL2L1/CCNT2/RUNX1/CD14/ZBTB16/PAX8/JUP/PAX5/SMAD1/RUNX1T1/BCL11B/IL6/H3C10/RXRA/ELK4/SLC45A3/MAF/CDKN1B/MEIS1/WT1/DEFA4/NFKB1/MET/ETV4/NTRK1/H3C3/DEFA6/MDM2/CD40/POLK/ID2/GADD45A/TRAF1/SIX1/NFKBIZ/CCNA1/DUSP6/BMI1/COMMD3-BMI1/HMGA2/LDB1/CDK14/JMJD1C/SPINT1/RXRG/HOXA10/CDKN1A/RUNX2/HOXA9/RXRB/HOXA11	59	1.480736598
hsa04935	Growth hormone synthesis, secretion and action	40/1682	119/8105	0.00068867	0.03105	0.02536	CACNA1D/PLCG1/ADCY7/SOCS3/PLCG2/IRS1/AKT3/ITPR1/SOS1/PRKCA/SHC2/CREBBP/PIK3R1/SSTR2/MAP2K3/CREB3L3/PLCB1/GNAQ/SSTR5/PIK3CB/SOCS2/SOCS1/SST/IRS2/CRKL/ADCY1/MAPK8/SHC4/JUNB/STAT1/MAP2K6/GHR/PRKCG/GRB2/ITPR3/PLCB3/GNAS/KRAS/MAPK14/ATF6B	40	1.619720421
hsa05223	Non-small cell lung cancer	27/1682	72/8105	0.00077671	0.03105	0.02536	PLCG1/PLCG2/KIF5C/AKT3/SOS1/PRKCA/PIK3R1/CDK6/CCND1/PIK3CB/RXRA/MET/HGF/RASSF5/ALK/POLK/CDKN2A/GADD45A/KIF5B/PRKCG/GRB2/RARB/RXRG/CDKN1A/KRAS/CDK4/RXRB	27	1.807000595
hsa04015	Rap1 signaling pathway	63/1682	210/8105	0.00085717	0.03105	0.02536	F2RL3/PLCG1/ADCY7/VEGFC/IGF1R/GRIN2A/VAV2/RAP1A/ADORA2B/FGF5/AKT3/EVL/PRKCA/FGFR3/RAC2/EFNA2/THBS1/FGFR2/PIK3R1/SKAP1/PARD6G/FGF4/ADORA2A/CTNND1/ITGB2/FGF9/MAP2K3/PDGFRA/PLCB1/ITGAL/GNAQ/PIK3CB/FGF3/PRKCZ/RALGDS/GNAO1/VASP/PDGFC/FGFR1/VEGFA/LCP2/PRKD2/PFN1/MET/HGF/VAV3/EFNA1/RAP1GAP/RASSF5/CRKL/ADCY1/GRIN2B/MAGI2/FGF17/FGF23/MAP2K6/KITLG/PRKCG/PLCB3/GNAS/KRAS/RAPGEF3/MAPK14	63	1.445600476

Supplemental Table 6 - Differentially Methylated Regions (DMRs) of the life style subscores

Chromosome	Start	End	UCSC RefGene name	Number CpGs	Min. smoothed FDR	Mean difference	Number CpGs	Min. smoothed FDR	Mean difference	Number CpGs	Min. smoothed FDR	Mean difference	Number CpGs	Min. smoothed FDR	Mean difference	Number CpGs	Min. smoothed FDR	Mean difference	Number CpGs	Min. smoothed FDR	Mean difference	Number CpGs	Min. smoothed FDR	Mean difference
							Alcohol adjusted			BMI adjusted			Physical activity adjusted			Age adjusted			Diet adjusted			Smoking adjusted		
chr6	74289464	74290516	-	4	0.000823766	0.011517388	-	-	-	4	0.001065115	0.011313714	-	-	-	4	0.001149473	0.011026843	-	-	-	-	-	-
chr7	28998009	28998860	*TRIL*	11	0.008682039	-0.001217955	-	-	-	11	0.010011386	-0.001295534	-	-	-	11	0.010864563	-0.001326629	-	-	-	-	-	-
chr5	368394	368843	*AHRR*	3	4.02E-05	0.024263206	3	0.000360628	0.027188054	3	4.12E-05	0.024451752	3	0.008917182	0.073260603	3	1.82E-05	0.024976901	-	-	-	-	-	-
chr9	138985942	138986379	*NACC2*	4	0.007266051	0.009571731	-	-	-	4	0.008215582	0.009586516	-	-	-	4	0.006428649	0.009726848	-	-	-	-	-	-
chr1	222011091	222011349	-	4	0.003353449	-0.013334396	-	-	-	4	0.004266579	-0.013094208	-	-	-	4	0.004473019	-0.012719536	-	-	-	-	-	-
chr18	76737888	76739120	*SALL3*	10	0.000956068	0.000863223	-	-	-	-	-	-	-	-	-	-	-	-	-	-	-	-	-	-
chr3	147072402	147073118	-	5	0.004665495	-0.012422004	-	-	-	5	0.005894565	-0.012296776	-	-	-	5	0.006671854	-0.012123829	-	-	-	-	-	-
chr7	16505094	16506521	*SOSTDC1*	9	1.25E-08	-0.029811065	-	-	-	9	1.02E-08	-0.030079954	-	-	-	9	2.55E-08	-0.029482963	-	-	-	-	-	-
chr3	159482413	159483186	*IQCJ-SCHIP1*																					
chr20	52789646	52791541	*CYP24A1*	15	0.008799081	-0.005441161	-	-	-	-	-	-	-	-	-	-	-	-	-	-	-	-	-	-
chr11	70439937	70440910	*SHANK2*	5	0.002539802	-0.001299915	5	0.003392681	-0.003223105	5	0.00274725	-0.00121328	-	-	-	5	0.002409282	-0.001443875	-	-	-	-	-	-
chr12	22590017	22590199	-	3	0.000344944	0.026765701	3	0.000410992	0.035083492	3	0.000455746	0.026515258	3	0.036612482	0.074556507	3	0.000305865	0.027054857	-	-	-	-	-	-
chr9	137978190	137979399	*OLFM1*	9	5.65E-05	-0.018412699	-	-	-	9	7.84E-05	-0.018253981	-	-	-	-	-	-	-	-	-	-	-	-
chr5	2756361	2757802	*IRX2*	7	9.21E-05	-0.006578694	-	-	-	7	0.000117834	-0.006555567	-	-	-	7	9.32E-05	-0.006531327	-	-	-	-	-	-
chr2	88751158	88752836	*FOXI3*	13	0.001158827	0.002348803	-	-	-	-	-	-	-	-	-	13	0.000675498	0.002567711	-	-	-	-	-	-
chr11	65149417	65150358	*FRMD8*																					
chr15	68522181	68523155	*CLN6*	9	0.000137223	0.008083537	-	-	-	9	0.00019312	0.008030527	-	-	-	9	0.000161999	0.007997863	-	-	-	-	-	-
chr6	32764865	32765402	-	14	0.000218996	-0.004706811	-	-	-	14	0.000321612	-0.004652521	-	-	-	14	0.000379792	-0.004720963	14	0.025644317	-0.005736354	-	-	-
chr19	42873162	42873953	*MEGF8*	8	0.001968404	0.006764882	-	-	-	8	0.002185497	0.006751221	-	-	-	8	0.002263371	0.006804488	-	-	-	-	-	-
chr14	106015750	106016106	-	3	0.006199685	0.00689657	-	-	-	3	0.007053725	0.006963862	-	-	-	3	0.006093495	0.006956357	-	-	-	-	-	-
chr9	35689475	35690395	*TPM2*	8	0.005588474	0.012354543	-	-	-	8	0.006312562	0.012312468	-	-	-	8	0.004088735	0.012650758	-	-	-	-	-	-
chr7	2082255	2083583	*MAD1L1*	9	0.00201604	0.004675991	-	-	-	9	0.002484788	0.004615433	-	-	-	9	0.001603163	0.004848867	-	-	-	-	-	-
chr3	194868427	194868843	*XXYLT1*																					
chr1	211652276	211652741	*RD3*	4	0.00105994	-0.025102059	-	-	-	4	0.00112281	-0.025157921	-	-	-	4	0.001572779	-0.02476213	-	-	-	-	-	-
chr6	78172192	78174286	*HTR1B*	15	0.005227971	-0.004321439	-	-	-	15	0.006677603	-0.004261633	-	-	-	15	0.007485408	-0.004185173	-	-	-	-	-	-
chr18	78005180	78006141	*PARD6G*	12	0.008014422	-0.010194208	-	-	-	12	0.01015066	-0.010094364	-	-	-	12	0.008332987	-0.010480231	-	-	-	-	-	-
chr3	157217187	157218259	*VEPH1*	13	0.000191495	-0.005979312	-	-	-	13	0.0002552	-0.005938308	-	-	-	13	0.000194682	-0.006053019	-	-	-	-	-	-
chr12	96336121	96337004	*AMDHD1*																					
chr1	19600141	19601069	*AKR7L*	8	0.000341046	-0.024798758	-	-	-	8	0.000179416	-0.025337593	-	-	-	8	0.00017818	-0.025352351	-	-	-	-	-	-
chr19	49636270	49636594	*PPFIA3*	3	0.005273815	0.029240507	-	-	-	3	0.005433578	0.029321964	-	-	-	3	0.003238448	0.030102031	-	-	-	-	-	-
chr2	242003078	242003549	*SNED1*	3	0.00452696	-0.028293871	-	-	-	3	0.005134368	-0.028280912	-	-	-	3	0.004794879	-0.028356623	-	-	-	-	-	-
chr6	108444105	108445534	-	9	0.000634718	-0.0229392	9	0.000920336	-0.02648672	9	0.000921458	-0.022596925	-	-	-	9	0.000498734	-0.023255478	-	-	-	-	-	-
chr2	118980919	118981783	-	5	0.007991824	-0.002328692	-	-	-	5	0.009367404	-0.002280551	-	-	-	5	0.008992785	-0.002557113	-	-	-	-	-	-
chr11	76571458	76571610	*ACER3*	5	0.008056623	0.006595271	-	-	-	5	0.009516008	0.006550464	-	-	-	5	0.006708737	0.006823279	-	-	-	-	-	-
chr1	42384564	42385941	*HIVEP3*	6	7.07E-07	-0.028861179	-	-	-	6	9.91E-07	-0.028782283	-	-	-	-	-	-	-	-	-	-	-	-
chr7	1882776	1883760	*MAD1L1*																					
chr1	155880507	155881569	*RIT1*	9	0.008030728	-0.005160604	-	-	-	9	0.009927301	-0.005178875	-	-	-	9	0.008683535	-0.005242962	-	-	-	-	-	-
chr13	113121107	113121632	-	4	0.005769802	0.0087651	-	-	-	-	-	-	-	-	-	4	0.006364911	0.008843537	-	-	-	-	-	-
chr10	135190860	135192232	*ECHS1*																					
chr6	23177275	23178052	-	5	0.000579664	-0.015447829	5	0.005083309	-0.020211748	5	0.000699633	-0.015109604	-	-	-	5	0.000588523	-0.015713717	-	-	-	-	-	-
chr19	2774149	2775228	*SGTA*	7	0.000518356	0.009786156	-	-	-	7	0.00059134	0.009873848	-	-	-	7	0.000810031	0.009535064	-	-	-	-	-	-
chr20	47895899	47897260	*SNORD12*																					
chr17	4079262	4079652	*ANKFY1*	4	0.001673176	-0.019608484	-	-	-	4	0.001804409	-0.019696955	-	-	-	4	0.001322346	-0.019720543	-	-	-	-	-	-
chr1	28831782	28832782	*RCC1*																					
chr2	160472660	160473496	*BAZ2B*	8	0.004154606	-0.010827186	-	-	-	8	0.005525773	-0.010548943	-	-	-	8	0.006534168	-0.010413277	-	-	-	-	-	-
chr3	12392792	12393021	*PPARG*	3	0.005575742	-0.012691305	-	-	-	3	0.00689073	-0.012555123	-	-	-	3	0.005574548	-0.012918467	-	-	-	-	-	-
chr14	23586582	23586886	*CEBPE*	3	0.002540887	-0.015600343	-	-	-	3	0.003137006	-0.015332643	-	-	-	3	0.002096112	-0.015907374	-	-	-	-	-	-
chr14	75469661	75470314	*EIF2B2*	5	0.005443242	0.001108955	-	-	-	5	0.006427642	0.001050329	-	-	-	5	0.005423763	0.000968419	-	-	-	-	-	-
chr1	209779784	209779995	*CAMK1G*	3	0.002517869	0.011066249	-	-	-	3	0.002960101	0.011101684	-	-	-	3	0.003167106	0.010932743	-	-	-	-	-	-
chr10	92979600	92980627	*PCGF5*	15	0.004581474	-0.003753986	-	-	-	15	0.006056693	-0.003767891	-	-	-	15	0.005644472	-0.00379355	-	-	-	-	-	-
chr1	120255318	120256112	*PHGDH*	5	0.000144926	0.017891977	-	-	-	5	0.000172793	0.017493439	-	-	-	5	0.000247527	0.017564829	-	-	-	-	-	-
chr20	642436	645199	*SCRT2*																					
chr3	11195673	11196445	*HRH1*	7	0.006582668	0.000541813	-	-	-	7	0.006416487	0.000657723	7	0.006332761	-0.008734874	7	0.00638803	0.000659821	-	-	-	-	-	-
chr9	841067	841850	*DMRT1*	8	0.004868288	0.004756887	-	-	-	8	0.005730024	0.004742483	-	-	-	8	0.005885765	0.004881057	-	-	-	-	-	-
chr12	133464665	133465592	*CHFR*	11	0.000435526	0.003241375	-	-	-	-	-	-	-	-	-	11	0.000510798	0.003237138	-	-	-	-	-	-
chr11	101743355	101744168	-	5	0.008997833	0.004373047	-	-	-	5	0.010403064	0.004315493	-	-	-	5	0.010466391	0.003943465	-	-	-	-	-	-
chr16	86011615	86012573	-	7	0.00103007	-0.010327177	-	-	-	7	0.001430468	-0.010225331	-	-	-	7	0.001439911	-0.010256501	7	0.00060287	-0.018642395	-	-	-
chr9	131083986	131085530	*COQ4*																					
chr8	142401373	142403574	-	9	0.007868104	0.018250824	-	-	-	9	0.008876626	0.018049783	-	-	-	9	0.011552423	0.017837241	-	-	-	-	-	-
chr19	55013104	55013954	*LAIR2*	7	0.000906925	-0.01789148	-	-	-	7	0.000977972	-0.017980516	-	-	-	7	0.001172182	-0.01781189	-	-	-	-	-	-
chr14	96505296	96505874	*C14orf132*	13	0.007051786	-0.000447002	-	-	-	13	0.008317282	-0.000396729	-	-	-	13	0.007918534	-0.000359177	-	-	-	-	-	-
chr21	45936193	45937406	*TSPEAR*																					
chr15	89973756	89974397	-	6	0.009377354	-0.008011849	-	-	-	6	0.010193119	-0.008058497	-	-	-	6	0.01052015	-0.008106013	-	-	-	-	-	-
chr13	41935539	41936259	*NAA16*	3	0.005807794	-0.014707327	-	-	-	3	0.007113391	-0.014550968	-	-	-	3	0.005461291	-0.014668554	-	-	-	-	-	-
chr14	88788343	88789655	*KCNK10*	12	1.67E-05	-0.01270038	-	-	-	12	2.05E-05	-0.012673785	-	-	-	12	2.50E-05	-0.012515821	-	-	-	-	-	-
chr12	93835624	93837247	*UBE2N*	14	0.001596521	-0.001279427	-	-	-	14	0.001959727	-0.001294564	-	-	-	14	0.001018003	-0.001409836	-	-	-	-	-	-
chr11	60282398	60282972	*MS4A13*	12	0.008145564	0.010836232	-	-	-	12	0.010543622	0.010720218	12	0.014966702	0.034137553	12	0.008248339	0.010983513	-	-	-	-	-	-
chr1	983386	984428	*AGRN*	6	0.002091437	0.012145661	-	-	-	-	-	-	-	-	-	6	0.002402519	0.01239299	-	-	-	-	-	-
chr20	13975439	13977219	*MACROD2*																					
chr10	102760724	102761169	*LZTS2*	3	0.000664826	-0.010327616	-	-	-	3	0.000703543	-0.010112952	-	-	-	3	0.00091281	-0.010154982	-	-	-	-	-	-
chr2	163625066	163625295	*KCNH7*	3	0.004235707	-0.022082792	3	0.028324026	-0.024143326	3	0.005040427	-0.021989706	-	-	-	3	0.0033512	-0.022510158	-	-	-	-	-	-
chr15	42119684	42120681	*JMJD7*																					
chr6	31632171	31634890	*C6orf47*																					
chr2	3652840	3654582	*COLEC11*	7	0.005128172	-0.008235315	-	-	-	7	0.005948612	-0.008268263	-	-	-	7	0.005621435	-0.008057924	-	-	-	-	-	-
chr14	76393953	76395305	*TTLL5*	3	0.000256606	-0.015388443	-	-	-	-	-	-	-	-	-	-	-	-	-	-	-	-	-	-
chr1	1713676	1714271	*NADK*	6	0.003870861	0.013320524	-	-	-	6	0.004696163	0.013226358	-	-	-	6	0.002829495	0.013643644	-	-	-	-	-	-
chr19	6534760	6535078	*TNFSF9*	5	0.00058806	0.023285074	-	-	-	5	0.000665747	0.023335569	5	0.007349997	0.079420568	5	0.000287939	0.023960007	-	-	-	-	-	-
chr6	29717475	29718119	*HLA-F-AS1*	5	0.003825975	0.00598489	-	-	-	5	0.004811251	0.005834757	-	-	-	5	0.004367959	0.005887384	-	-	-	-	-	-
chr10	81904244	81904814	*PLAC9*	3	0.008766822	-0.010430406	-	-	-	3	0.010292676	-0.01034837	-	-	-	3	0.009774325	-0.010386797	-	-	-	-	-	-
chr6	39196071	39197655	*KCNK5*	10	0.00050486	-0.007600078	-	-	-	10	0.000570964	-0.007675379	-	-	-	10	0.000402769	-0.007734554	-	-	-	-	-	-
chr12	54954337	54955143	*PDE1B*	6	4.18E-06	-0.014663833	-	-	-	6	6.21E-06	-0.014517293	-	-	-	6	6.22E-06	-0.014462071	-	-	-	-	-	-
chr6	159240453	159241461	*EZR*																					
chr14	105131910	105132869	-	7	0.000865208	0.006232595	-	-	-	7	0.001090324	0.006145727	-	-	-	7	0.00094323	0.006166533	-	-	-	-	-	-
chr19	45909124	45910448	*POLR1G*																					
chr17	27892866	27893731	*ABHD15*																					
chr11	831584	832821	*CD151*																					
chr20	62130111	62131189	*EEF1A2*	7	0.007604211	-0.011102257	-	-	-	7	0.009986553	-0.011155079	-	-	-	7	0.006050567	-0.01130156	-	-	-	-	-	-
chr10	118368264	118368986	*PNLIPRP1*	3	0.000228196	0.023882764	3	0.001694802	0.028528093	3	0.00030373	0.023735922	-	-	-	3	0.000173758	0.024170728	-	-	-	-	-	-
chr15	96909816	96911696	-	8	0.000536331	0.004383319	-	-	-	8	0.000639682	0.004351141	-	-	-	8	0.000645046	0.004978072	-	-	-	-	-	-
chr1	209957965	209958678	*C1orf74*	9	0.006178619	-0.005474861	-	-	-	-	-	-	-	-	-	-	-	-	-	-	-	-	-	-
chr6	28554680	28558113	*ZBED9*	46	0.001239757	-0.002556156	-	-	-	46	0.00155236	-0.002551692	-	-	-	46	0.001707656	-0.002459099	-	-	-	-	-	-
chr2	189158094	189158532	*GULP1*																					
chr7	96745696	96746356	*SDHAF3*	3	0.001479667	-0.002158568	-	-	-	3	0.001705906	-0.002247237	-	-	-	3	0.001924313	-0.002040589	-	-	-	-	-	-
chr1	19832006	19832487	-	3	0.002487012	-0.006338553	-	-	-	3	0.003098629	-0.006315478	-	-	-	3	0.003080266	-0.00643116	-	-	-	-	-	-
chr5	149545907	149546934	*CDX1*	8	0.002624186	0.022037603	-	-	-	8	0.003077806	0.022003566	8	0.002289988	0.080728729	8	0.002785081	0.022238642	-	-	-	-	-	-
chr4	89978251	89978732	*FAM13A*	7	0.000469395	-0.012012231	-	-	-	7	0.000618342	-0.011852876	-	-	-	7	0.000719042	-0.011741918	7	0.028880471	-0.012365464	-	-	-
chr4	18320820	18321251	-	4	1.36E-05	-0.030052878	4	0.001905609	-0.030548534	4	1.88E-05	-0.029817682	-	-	-	4	7.72E-06	-0.030343915	-	-	-	-	-	-
chr6	26205212	26205610	*H4C5*	4	0.003690776	-0.002402529	-	-	-	4	0.004544523	-0.002278552	-	-	-	4	0.00447635	-0.002562536	-	-	-	-	-	-
chr6	29974319	29975675	*HLA-G*																					
chr6	2999095	3000377	*NQO2*	14	0.000505326	-0.009924224	-	-	-	14	0.000729723	-0.009993006	-	-	-	-	-	-	-	-	-	-	-	-
chr13	107001538	107001893	-	4	0.008543648	-0.006983627	-	-	-	4	0.010543622	-0.006929096	-	-	-	4	0.012561659	-0.006711663	-	-	-	-	-	-
chr6	29454623	29455302	*MAS1L*	8	0.003167133	-0.005908028	-	-	-	-	-	-	-	-	-	8	0.003549113	-0.005941441	-	-	-	-	-	-
chr14	25045625	25046267	*CTSG*	6	0.008324433	-0.006167425	-	-	-	-	-	-	-	-	-	-	-	-	-	-	-	-	-	-
chr12	111618925	111619455	*CUX2*	8	0.009304416	-0.012905898	-	-	-	8	0.010913953	-0.012927214	-	-	-	8	0.012926519	-0.012747751	-	-	-	-	-	-
chr11	94500612	94500965	*AMOTL1*	3	0.008103758	0.019991656	-	-	-	3	0.009823758	0.019905024	-	-	-	3	0.010418235	0.01966956	-	-	-	-	-	-
chr20	44033595	44034880	*DBNDD2*																					
chr11	120195717	120197726	*TLCD5*	11	0.000160217	-0.007934281	-	-	-	-	-	-	-	-	-	-	-	-	-	-	-	-	-	-

chr17	27346732	27347260	-	6	0.000502104	0.012053174	-	-	-	6	0.000605919	0.012066163	-	-	-	6	0.000684401	0.011977156	6	0.032622081	0.017719069	-	-	-
chr10	118380116	118381044	*PNLIPRP2*	5	0.004399737	0.018435868	-	-	-	5	0.004982223	0.018436892	-	-	-	5	0.004688486	0.018507754	-	-	-	-	-	-
chr7	117512803	117513956	*CTTNBP2*	8	0.002441978	-0.009408706	-	-	-	8	0.003171547	-0.009315347	-	-	-	8	0.003858264	-0.009098293	-	-	-	-	-	-
chr19	51980685	51980908	*CEACAM18*	3	0.007632054	-0.034346322	-	-	-	3	0.009055446	-0.034292955	-	-	-	3	0.009316818	-0.033758496	-	-	-	-	-	-
chr1	2344426	2346088	*PEX10*	18	4.42E-05	0.009967219	-	-	-	-	-	-	-	-	-	-	-	-	-	-	-	-	-	-
chr5	175199915	175200576	-	7	0.001275615	-0.005896828	-	-	-	7	0.001585328	-0.005917221	-	-	-	7	0.001891166	-0.005874176	-	-	-	-	-	-
chr2	231737741	231737958	*ITM2C*	5	0.00512212	0.012033168	-	-	-	5	0.006334383	0.011874069	-	-	-	5	0.007268564	0.01121829	-	-	-	-	-	-
chr6	150070030	150070714	*NUP43*																					
chr3	49209160	49210221	*KLHDC8B*	3	0.002639425	0.010810333	-	-	-	-	-	-	-	-	-	3	0.003460115	0.010767362	-	-	-	-	-	-
chr14	35590656	35591840	*PPP2R3C*																					
chr6	74018833	74020214	*KHDC1*	11	0.001100809	0.000223751	-	-	-	11	0.001557581	0.000195778	-	-	-	11	0.001350108	0.000153185	-	-	-	-	-	-
chr10	13628544	13629624	*PRPF18*	9	0.002627255	-1.44E-05	-	-	-	9	0.002766446	-2.30E-05	-	-	-	9	0.001722964	-6.02E-05	-	-	-	-	-	-
chr4	1684739	1686288	*FAM53A*	9	3.03E-05	-0.001925464	-	-	-	9	3.79E-05	-0.00183305	-	-	-	9	3.94E-05	-0.001865756	-	-	-	-	-	-
chr7	48075568	48076004	*C7orf57*	5	0.008326226	-0.008631762	-	-	-	5	0.008895477	-0.008612122	-	-	-	5	0.010978327	-0.008318028	-	-	-	-	-	-
chr20	36530296	36531652	*VSTM2L*	9	0.001090013	-0.006564957	-	-	-	9	0.001446798	-0.006447535	-	-	-	9	0.001322752	-0.006539157	-	-	-	-	-	-
chr20	44746392	44747351	*CD40*	9	0.008177555	-0.006120395	-	-	-	9	0.009594451	-0.006110048	-	-	-	9	0.007967902	-0.006010291	-	-	-	-	-	-
chr1	32222440	32223040	*ADGRB2*	5	0.004555768	0.010426222	-	-	-	5	0.005381915	0.010414647	-	-	-	5	0.006262908	0.010459726	-	-	-	-	-	-
chr19	17932512	17933786	*INSL3*	4	0.000121474	0.01259964	-	-	-	-	-	-	-	-	-	4	6.06E-05	0.012818042	-	-	-	-	-	-
chr14	73392648	73394847	*DCAF4*	16	0.001107905	-0.000674173	-	-	-	-	-	-	-	-	-	16	0.000921464	-0.000604971	-	-	-	-	-	-
chr19	38916488	38917025	*RASGRP4*	6	0.003727397	-0.004147405	-	-	-	6	0.004635519	-0.004065945	-	-	-	6	0.005554874	-0.00407871	-	-	-	-	-	-
chr1	44430285	44430921	*DPH2*																					
chr3	13008800	13010184	*IQSEC1*	16	0.005843129	-0.004262267	-	-	-	-	-	-	-	-	-	-	-	-	-	-	-	-	-	-
chr11	64780701	64781842	*ARL2*																					
chr2	46465	47716	*FAM110C*	15	7.06E-05	-0.010287802	-	-	-	15	0.000114018	-0.010225948	-	-	-	15	0.000127189	-0.009979946	-	-	-	-	-	-
chr10	45494806	45496315	*ZNF22*																					
chr3	184278268	184279593	*EPHB3*	11	0.003946856	-0.001946655	-	-	-	-	-	-	-	-	-	-	-	-	-	-	-	-	-	-
chr3	10362520	10363474	*SEC13*	10	0.004141213	-0.000244343	-	-	-	10	0.005072214	-0.000257219	-	-	-	10	0.002677945	-0.000327657	-	-	-	-	-	-
chr10	134062078	134062614	*STK32C*	4	0.000136013	-0.025383616	-	-	-	4	0.000179112	-0.025315337	-	-	-	4	0.000143703	-0.025323738	-	-	-	-	-	-
chr3	127006287	127006803	-	4	0.008295151	0.002236808	-	-	-	4	0.00910888	0.002175492	-	-	-	4	0.008860861	0.002414427	-	-	-	-	-	-
chr7	22893924	22894898	*SNORD93*	8	0.006597785	0.005308693	-	-	-	8	0.007191929	0.005140288	-	-	-	8	0.005226916	0.005139654	-	-	-	-	-	-
chr16	21963849	21965022	*UQCRC2*	13	0.00208175	-0.006328479	-	-	-	-	-	-	-	-	-	-	-	-	-	-	-	-	-	-
chr4	78978133	78979207	*FRAS1*	12	2.50E-05	-0.002537353	-	-	-	12	3.68E-05	-0.002512355	-	-	-	12	2.82E-05	-0.00247627	-	-	-	-	-	-
chr10	45420821	45420996	*TMEM72*																					
chr2	33359059	33359688	*LTBP1*	11	0.001857956	-0.009779196	11	0.008488239	-0.012963252	11	0.002112078	-0.009794686	-	-	-	11	0.001585608	-0.01005506	-	-	-	-	-	-
chr17	79800859	79801920	*P4HB*	10	0.005602698	-0.006270666	-	-	-	10	0.006004842	-0.006266506	-	-	-	10	0.005947268	-0.006277411	-	-	-	-	-	-
chr17	76037035	76037562	*TNRC6C*	6	0.002382131	-0.021393495	-	-	-	6	0.003219171	-0.02113589	-	-	-	6	0.00393044	-0.020615036	-	-	-	-	-	-
chr21	42538388	42539633	*BACE2*																					
chr21	46685154	46686430	*POFUT2*	6	0.005424448	0.003059261	-	-	-	6	0.006109547	0.002974898	-	-	-	6	0.004978556	0.00306875	-	-	-	-	-	-
chr22	39953557	39954239	-	5	0.00731919	-0.000777304	-	-	-	5	0.009049184	-0.000725765	-	-	-	5	0.010257745	-0.000766067	-	-	-	-	-	-
chr16	1071049	1072056	-	4	0.009168299	0.018142234	-	-	-	-	-	-	-	-	-	-	-	-	-	-	-	-	-	-
chr1	175985115	175986331	*COP1*	5	0.005379113	-0.003294629	-	-	-	-	-	-	-	-	-	-	-	-	-	-	-	-	-	-
chr19	14800564	14801245	*ZNF333*	10	0.007854796	-0.005950252	-	-	-	10	0.009832068	-0.005917687	-	-	-	10	0.006534168	-0.006074822	-	-	-	-	-	-
chr9	36738765	36739781	-	3	0.000995931	0.013379297	-	-	-	3	0.000998005	0.012966194	-	-	-	3	0.000765252	0.013687824	-	-	-	-	-	-
chr5	134180705	134182149	*C5orf24*	17	0.001207346	-0.003829447	-	-	-	17	0.001613063	-0.003797361	-	-	-	17	0.001056392	-0.003830125	-	-	-	-	-	-
chr3	71803339	71804859	*EIF4E3*																					
chr7	4874285	4875094	*RADIL*	8	0.00177121	0.013438089	-	-	-	-	-	-	8	0.00901817	0.045432338	8	0.00252962	0.013124095	-	-	-	-	-	-
chr1	22140769	22141400	*LDLRAD2*	6	0.009005107	0.011017246	-	-	-	6	0.011067847	0.010946876	-	-	-	6	0.010115695	0.010940271	-	-	-	-	-	-
chr11	2440032	2440386	*TRPM5*	3	0.008951296	0.014716597	-	-	-	3	0.010335092	0.014626657	-	-	-	3	0.011040464	0.014312117	-	-	-	-	-	-
chr14	22950395	22951393	-	6	0.002216955	-0.018242935	-	-	-	6	0.00272409	-0.018097645	-	-	-	6	0.003229457	-0.017832282	-	-	-	-	-	-
chr4	150998949	150999949	*DCLK2*	15	0.007271705	-0.003296367	-	-	-	-	-	-	-	-	-	-	-	-	-	-	-	-	-	-
chr2	136577303	136577747	*LCT*																					
chr22	31002362	31003655	*PES1*																					
chr12	62995960	62998134	*LINC01465*																					
chr22	35795274	35795394	*MCM5*	5	0.009890407	0.013101943	-	-	-	5	0.009062569	0.01326634	-	-	-	5	0.009008482	0.013141502	-	-	-	-	-	-
chr19	5210505	5210847	*PTPRS*	5	0.006284096	0.009465785	-	-	-	5	0.006788458	0.009504673	-	-	-	5	0.005563903	0.00975936	-	-	-	-	-	-
chr11	7597402	7598673	*PPFIBP2*	11	2.33E-05	-0.015330363	-	-	-	11	3.18E-05	-0.015292411	-	-	-	11	5.35E-06	-0.015879877	-	-	-	-	-	-
chr2	106776571	106777215	*UXS1*	6	0.0052512	-0.004703563	-	-	-	6	0.005994021	-0.004749541	-	-	-	6	0.004908556	-0.004813538	-	-	-	-	-	-
chr7	984874	985784	*ADAP1*	9	0.003895336	0.0076486	-	-	-	-	-	-	-	-	-	-	-	-	-	-	-	-	-	-
chr17	37894014	37895886	*GRB7*	15	0.001448588	-0.008984902	-	-	-	-	-	-	-	-	-	-	-	-	-	-	-	-	-	-
chr12	752290	753199	*NINJ2*	9	0.005707633	0.009056121	-	-	-	9	0.007224661	0.008977952	-	-	-	9	0.006607468	0.008790181	-	-	-	-	-	-
chr5	138629998	138630502	*MATR3*	5	0.008049882	-0.005910693	-	-	-	5	0.010411059	-0.005958394	-	-	-	5	0.008777624	-0.006008789	-	-	-	-	-	-
chr4	86395989	86396765	*ARHGAP24*	14	1.80E-05	-0.010901102	-	-	-	14	3.21E-05	-0.01083825	-	-	-	14	2.57E-05	-0.010783717	-	-	-	-	-	-
chr7	16625747	16626530	-	4	0.000861159	0.028766928	-	-	-	4	0.00105006	0.028771223	-	-	-	4	0.001033768	0.02872903	-	-	-	-	-	-
chr7	1913505	1914523	*MAD1L1*	6	0.006100933	0.011737291	-	-	-	6	0.007597337	0.011687319	-	-	-	6	0.008574283	0.01163632	-	-	-	-	-	-
chr6	33286790	33291586	*DAXX*																					
chr20	13200685	13201551	*ISM1*	20	0.000391992	-0.009123201	-	-	-	20	0.000545615	-0.009057015	-	-	-	20	0.000660339	-0.008758556	-	-	-	-	-	-
chr12	50101027	50101564	*FMNL3*	6	0.000286796	-0.007760641	-	-	-	6	0.000311141	-0.007820546	-	-	-	6	0.000209957	-0.00787575	-	-	-	-	-	-
chr7	2701419	2701802	*TTYH3*	5	0.000958546	0.018044774	-	-	-	5	0.001191454	0.018034106	5	0.001274046	0.063294872	5	0.001285017	0.018016081	-	-	-	-	-	-
chr14	77247777	77248049	*VASH1*	5	2.76E-05	0.024964563	-	-	-	5	3.58E-05	0.024918993	5	0.007441474	0.082037846	5	3.18E-05	0.025232947	-	-	-	-	-	-
chr7	27142275	27144302	*HOXA2*	21	1.25E-08	0.020087313	-	-	-	-	-	-	-	-	-	21	1.21E-08	0.020191425	-	-	-	-	-	-
chr4	81187906	81189164	*FGF5*	6	0.000109505	-0.003253121	-	-	-	6	8.16E-05	-0.003200339	-	-	-	6	0.000128923	-0.003286294	-	-	-	-	-	-
chr15	42840397	42841312	*HAUS2*																					
chr9	98314632	98316500	-	7	5.79E-06	0.0072153	-	-	-	7	8.33E-06	0.007194713	-	-	-	7	6.58E-06	0.007211806	-	-	-	-	-	-
chr1	158223034	158223934	*CD1A*	5	0.006773501	-0.008441203	-	-	-	5	0.007930541	-0.008225012	-	-	-	5	0.006309363	-0.008380901	-	-	-	-	-	-
chr2	120005223	120005755	*STEAP3*	6	0.005481572	0.013157874	-	-	-	6	0.006931783	0.013033259	-	-	-	6	0.007709085	0.01284589	-	-	-	-	-	-
chr19	58962093	58963245	*ZNF324B*	13	7.95E-07	-0.00033706	-	-	-	13	1.22E-06	-0.000464391	-	-	-	13	9.22E-07	-0.000455238	-	-	-	-	-	-
chr16	88547533	88547861	*ZFPM1*	4	0.000854763	0.01260916	4	0.017551606	0.013575228	4	0.000986309	0.012580077	-	-	-	4	0.000934531	0.012596706	-	-	-	-	-	-
chr16	62070306	62071129	*CDH8*	9	0.008009014	-0.003602689	-	-	-	9	0.00910888	-0.003512718	-	-	-	-	-	-	-	-	-	-	-	-
chr12	12848516	12850082	*GPR19*	14	0.003803816	0.005606122	-	-	-	14	0.004604703	0.005493513	-	-	-	14	0.003836303	0.005543975	-	-	-	-	-	-
chr13	112986575	112986978	-	5	0.001796539	-0.024121189	-	-	-	5	0.002132676	-0.023689971	-	-	-	5	0.002600763	-0.023673818	-	-	-	-	-	-
chr2	203776148	203777060	*CARF*																					
chr5	150631895	150632733	*GM2A*	11	0.003339018	-0.012295035	-	-	-	11	0.004423027	-0.012413344	-	-	-	11	0.0033512	-0.012400138	-	-	-	-	-	-
chr2	239036809	239037533	*ESPNL*	6	0.006479656	-0.024482411	-	-	-	6	0.007806967	-0.02444832	-	-	-	6	0.009127316	-0.024117724	6	0.009770086	-0.043260198	-	-	-
chr16	58540765	58541612	*NDRG4*	4	0.004424921	-0.002030547	-	-	-	4	0.004974282	-0.002114205	-	-	-	4	0.006331998	-0.002181895	-	-	-	-	-	-
chr7	1990554	1992159	*MAD1L1*	10	0.00966625	0.009821288	-	-	-	-	-	-	-	-	-	10	0.010046733	0.009955732	-	-	-	-	-	-
chr16	66399746	66400599	*CDH5*	10	0.000217509	-0.005211816	-	-	-	10	0.000326825	-0.005132778	-	-	-	10	0.000259069	-0.00543253	-	-	-	-	-	-
chr21	42791867	42792703	*MX1*	11	0.003411938	0.003261193	-	-	-	11	0.004588837	0.003235783	-	-	-	11	0.00412539	0.003232739	-	-	-	-	-	-
chr20	2539517	2539876	*TMC2*	5	0.003333521	-0.011141126	-	-	-	5	0.003424211	-0.011273862	-	-	-	5	0.002939653	-0.011294965	-	-	-	-	-	-
chr15	75018065	75020202	*CYP1A1*	28	1.07E-12	0.011364887	-	-	-	-	-	-	-	-	-	28	8.51E-13	0.011405388	-	-	-	-	-	-
chr16	86591940	86592652	*MTHFSD*	5	0.000120603	0.017433912	-	-	-	5	0.000153917	0.017310464	-	-	-	5	0.000104662	0.01765568	-	-	-	-	-	-
chr5	134364583	134364968	*C5orf66*																					
chr1	150551925	150552817	*MCL1*	17	0.006357925	-0.00154206	-	-	-	17	0.008071769	-0.001570595	-	-	-	17	0.007221429	-0.001754082	-	-	-	-	-	-
chr1	10709185	10711695	*CASZ1*	10	0.000780283	-0.017693263	-	-	-	10	0.000804166	-0.01781168	-	-	-	10	0.000425458	-0.018876933	-	-	-	-	-	-
chr6	31124978	31126284	*CCHCR1*																					
chr5	16179012	16180419	*MARCHF11*	16	0.001410583	-0.009558192	-	-	-	-	-	-	-	-	-	-	-	-	-	-	-	-	-	-
chr9	139096080	139096889	*LHX3*	5	0.007567225	-0.014585973	-	-	-	5	0.009304131	-0.014293752	-	-	-	-	-	-	-	-	-	-	-	-
chr5	1227968	1229127	*SLC6A18*	5	0.001896877	-0.020921319	-	-	-	5	0.001497971	-0.021308255	-	-	-	5	0.002266464	-0.020809165	-	-	-	-	-	-
chr4	88312323	88313064	*HSD17B11*	10	0.005865514	-0.012705978	-	-	-	10	0.007235125	-0.012769877	-	-	-	10	0.005708065	-0.012991737	-	-	-	-	-	-
chr10	22765645	22766320	-	5	0.000656812	0.018739098	-	-	-	5	0.000746405	0.018722462	-	-	-	5	0.000523144	0.019099093	-	-	-	-	-	-
chr4	154605468	154606177	*TLR2*	3	0.000167064	-0.002555729	3	2.87E-05	0.00093235	3	0.000207847	-0.002202394	-	-	-	3	0.000219229	-0.002661547	-	-	-	-	-	-
chr8	11302983	11303067	*FAM167A*	3	0.006341817	0.01803937	-	-	-	3	0.007284505	0.018001836	-	-	-	3	0.006528413	0.017969913	-	-	-	-	-	-
chr13	112861499	112862112	-	4	0.006073538	-0.029187203	-	-	-	4	0.007555718	-0.028958906	-	-	-	4	0.00598386	-0.029560981	-	-	-	-	-	-
chr5	1881655	1883514	*IRX4*	11	0.0031751	1.53E-05	-	-	-	11	0.004048672	2.81E-05	-	-	-	11	0.003175377	0.000354451	-	-	-	-	-	-
chr2	154335184	154336401	*RPRM*	16	0.001509489	-0.00291657	-	-	-	16	0.002172472	-0.002936122	-	-	-	16	0.001669931	-0.003119339	-	-	-	-	-	-
chr12	96252066	96253460	*SNRPF*	17	0.00017332	0.003775394	-	-	-	17	0.000248522	0.003778555	-	-	-	17	0.000229622	0.003686131	-	-	-	-	-	-
chr11	66631012	66631894	*PC*	6	0.008525535	0.009782165	-	-	-	6	0.00925292	0.009735828	-	-	-	6	0.008638144	0.009833646	-	-	-	-	-	-
chr7	158512380	158512671	-	4	0.004928118	-0.017257858	-	-	-	4	0.005757566	-0.017366314	-	-	-	4	0.0063227	-0.01718762	4	0.025898015	-0.030158445	-	-	-
chr4	163084858	163085697	*FSTL5*	12	2.82E-06	-0.007312016	12	0.000100412	-0.009131713	12	4.08E-06	-0.007292164	-	-	-	12	6.87E-06	-0.007112825	-	-	-	-	-	-
chr14	64760831	64761822	*ESR2*	11	0.003558699	-0.004449027	-	-	-	11	0.004663925	-0.004464033	-	-	-	11	0.002409282	-0.004652438	-	-	-	-	-	-
chr2	196520930	196522377	*SLC39A10*	18	0.009231889	0.00220968	-	-	-	-	-	-	-	-	-	18	0.008839175	0.002259689	-	-	-	-	-	-
chr5	176326365	176327557	*HK3*	7	0.003586626	-0.001686893	-	-	-	7	0.004583021	-0.001624906	-	-	-	-	-	-	-	-	-	-	-	-

chr19	14042840	14044197	*PODNL1*	5	0.001643745	0.014965996	-	-	-	5	0.001964328	0.015007524	-	-	-	5	0.001567392	0.015127266	-	-	-	-	-	-
chr10	45359611	45360313	*TMEM72-AS1*	5	0.000585616	-0.023658541	-	-	-	5	0.000781727	-0.023484302	-	-	-	5	0.0008176	-0.023455318	-	-	-	-	-	-
chr1	17914070	17914803	*ARHGEF10L*	4	0.000108715	0.01395124	-	-	-	4	0.000130808	0.013941869	4	0.002189384	0.024077757	4	0.000141005	0.013937193	-	-	-	-	-	-
chr19	7561739	7562555	*TEX45*	7	0.000447862	-0.004542154	-	-	-	7	0.000584753	-0.004473018	-	-	-	7	0.000428932	-0.00468671	-	-	-	-	-	-
chr7	27208577	27210940	*HOXA10*																					
chr7	30959709	30960418	*AQP1*	5	0.005458336	-0.008591388	-	-	-	5	0.006840995	-0.008528146	-	-	-	5	0.004809946	-0.009026808	-	-	-	-	-	-
chr1	25298480	25298996	-	3	0.00137738	0.007513939	-	-	-	3	0.001509462	0.007661065	-	-	-	3	0.001602958	0.007537671	-	-	-	-	-	-
chr1	67773128	67773725	*IL12RB2*	6	0.005047541	-0.004206722	-	-	-	-	-	-	-	-	-	6	0.006326175	-0.004215858	-	-	-	-	-	-
chr22	47022471	47023209	*GRAMD4*	8	0.008525535	0.01372117	-	-	-	8	0.011139923	0.01354112	-	-	-	8	0.0101757	0.013591608	-	-	-	-	-	-
chr17	42295591	42296315	*UBTF*	11	0.004442985	-0.007190693	-	-	-	11	0.005084899	-0.007105392	-	-	-	11	0.003097014	-0.007056964	-	-	-	-	-	-
chr17	40260053	40260548	*DHX58*	5	0.009510099	0.002708865	-	-	-	5	0.009765922	0.00271353	-	-	-	5	0.008453913	0.002680718	-	-	-	-	-	-
chr9	139871820	139872839	*LCNL1*																					
chr3	74662850	74663024	-	3	0.008122101	0.021282272	-	-	-	3	0.009611583	0.021124839	-	-	-	3	0.010925839	0.020668525	3	0.000143771	0.044012522	-	-	-
chr8	21988724	21989531	*HR*	7	0.0010764	0.01268217	-	-	-	-	-	-	-	-	-	7	0.000970031	0.012675873	-	-	-	-	-	-
chr3	196157895	196158975	*UBXN7*	7	0.005139061	-0.004019268	-	-	-	-	-	-	-	-	-	7	0.007224717	-0.004062282	-	-	-	-	-	-
chr12	122442301	122443562	-	4	0.002832364	-0.023723107	-	-	-	-	-	-	-	-	-	-	-	-	-	-	-	-	-	-
chr6	31465344	31466722	*MICB*	19	0.001943742	0.000120115	-	-	-	19	0.002132676	0.000168529	-	-	-	19	0.001679196	0.000159437	-	-	-	-	-	-
chr18	77960683	77960807	*PARD6G*	4	0.009459588	0.017004222	-	-	-	4	0.011222907	0.016850213	-	-	-	4	0.011147506	0.016770549	-	-	-	-	-	-
chr4	56023423	56024434	-	6	0.003225099	0.030831657	-	-	-	6	0.004399173	0.030304585	-	-	-	6	0.005068449	0.030219337	-	-	-	-	-	-
chr14	65380577	65381608	*CHURC1*																					
chr6	32820355	32822043	*PSMB9*																					
chr19	10697690	10698384	*AP1M2*	9	0.001073297	0.017250281	-	-	-	9	0.001357169	0.017218203	-	-	-	9	0.000598957	0.017905614	-	-	-	-	-	-
chr2	238768032	238769419	*RAMP1*	9	0.003011281	0.013815142	-	-	-	-	-	-	-	-	-	-	-	-	-	-	-	-	-	-
chr10	94831650	94834219	*CYP26A1*	19	0.009076291	-0.000525959	-	-	-	-	-	-	-	-	-	19	0.007117579	-0.000501011	-	-	-	-	-	-
chr13	39260874	39262298	*FREM2*	11	0.004683782	-0.00323653	-	-	-	11	0.005293389	-0.003193906	-	-	-	-	-	-	-	-	-	-	-	-
chr9	129676656	129677882	*RALGPS1*	11	0.006118226	-0.009506366	-	-	-	-	-	-	-	-	-	-	-	-	-	-	-	-	-	-
chr15	67546975	67548075	*AAGAB*																					
chr2	108994116	108994528	*SULT1C4*	7	0.009317037	-0.010305073	-	-	-	7	0.011763602	-0.010245571	-	-	-	7	0.01409963	-0.009981318	-	-	-	-	-	-
chr2	220197274	220197989	*RESP18*	6	0.000661508	-0.005452407	-	-	-	6	0.000692051	-0.005399861	-	-	-	6	0.000531445	-0.005829907	-	-	-	-	-	-
chr13	51417469	51418614	*DLEU7*																					
chr18	3593461	3595113	*DLGAP1*																					
chr2	43188851	43189254	-	3	0.005177714	-0.011864373	-	-	-	3	0.006035591	-0.011711559	-	-	-	3	0.006702083	-0.011440464	-	-	-	-	-	-
chr16	2770211	2771425	*PRSS27*	10	7.49E-05	0.003327109	-	-	-	10	7.82E-05	0.003367562	-	-	-	10	6.85E-05	0.003404338	-	-	-	-	-	-
chr21	38069950	38071688	*SIM2*	16	0.000953731	-0.000128521	-	-	-	16	0.001082277	-8.57E-05	-	-	-	16	0.00082265	-1.19E-05	-	-	-	-	-	-
chr11	32451461	32452839	*WT1*																					
chr7	2646782	2648785	*IQCE*	12	0.007374193	0.005280171	-	-	-	-	-	-	-	-	-	-	-	-	-	-	-	-	-	-
chr12	3361725	3361996	*TSPAN9*	3	0.00445093	-0.002574259	-	-	-	3	0.005317399	-0.002552039	-	-	-	3	0.00333412	-0.003189759	-	-	-	-	-	-
chr2	66664345	66665027	*MEIS1*																					
chr22	31217230	31217665	*OSBP2*	3	0.009115649	0.013234803	-	-	-	3	0.010866014	0.013160489	-	-	-	3	0.010042707	0.013185873	-	-	-	-	-	-
chr5	35924618	35925474	*CAPSL*	4	0.004901698	-0.003350527	-	-	-	4	0.006061069	-0.003360086	-	-	-	4	0.005332038	-0.003390044	-	-	-	-	-	-
chr14	61787418	61788345	*PRKCH*	10	0.001487712	0.002694686	-	-	-	10	0.001934738	0.002662638	-	-	-	10	0.001332698	0.002650298	-	-	-	-	-	-
chr4	24914441	24914970	*CCDC149*	8	0.003328501	-0.010722029	-	-	-	8	0.004139412	-0.010792325	-	-	-	8	0.00424614	-0.010600308	-	-	-	-	-	-
chr1	12508973	12509707	*VPS13D*	4	0.004280724	-0.017445368	-	-	-	4	0.005085501	-0.017374909	-	-	-	4	0.004142051	-0.017530932	-	-	-	-	-	-
chr4	183245061	183245618	*TENM3*	5	0.005694729	-0.001588155	-	-	-	5	0.00623375	-0.001610399	5	0.005486602	0.000391107	5	0.004456849	-0.001755161	-	-	-	-	-	-
chr19	6425207	6425737	*KHSRP*	4	0.00960154	0.00333132	-	-	-	4	0.01119258	0.003127688	-	-	-	4	0.010962749	0.003500344	-	-	-	-	-	-
chr20	37359533	37359998	-	5	0.000168468	-0.000250261	-	-	-	5	0.00021141	-0.000241921	-	-	-	5	0.000214084	-0.000284149	5	0.00803455	-0.002215233	-	-	-
chr21	44817305	44817925	-	4	0.000311643	0.01138908	4	0.015972154	0.012602144	4	0.000342979	0.011321988	4	0.005971356	0.008799911	4	0.000297223	0.011338202	-	-	-	-	-	-
chr1	24068409	24069431	*ELOA*	3	0.001054857	0.001020018	-	-	-	-	-	-	-	-	-	-	-	-	-	-	-	-	-	-
chr1	10764512	10764896	*CASZ1*	3	0.000837408	0.022887537	-	-	-	3	0.001043515	0.022831618	-	-	-	3	0.000355834	0.023586789	-	-	-	-	-	-
chr1	10713944	10714701	*CASZ1*	5	0.003809572	0.011896263	-	-	-	5	0.004877414	0.011790672	-	-	-	5	0.004344961	0.012038428	-	-	-	-	-	-
chr15	23933002	23933620	*NDN*	4	0.004374876	-0.001418565	-	-	-	4	0.005055421	-0.001440407	-	-	-	4	0.004317861	-0.001981924	-	-	-	-	-	-
chr4	74296058	74296726	*AFP*	3	0.005041752	0.008030932	-	-	-	3	0.005741484	0.007880729	-	-	-	3	0.006804723	0.00772031	-	-	-	-	-	-
chr12	34755568	34756440	-	5	0.008371233	-0.019172617	-	-	-	5	0.008395043	-0.019264053	-	-	-	5	0.00826767	-0.019217215	-	-	-	-	-	-
chr2	128388876	128389207	*MYO7B*	3	0.005195346	0.01340831	-	-	-	3	0.006381737	0.013317835	-	-	-	3	0.00540365	0.013415128	-	-	-	-	-	-
chr17	2595701	2595994	*CLUH*	3	0.00465914	0.013692634	-	-	-	3	0.005618704	0.013584565	-	-	-	3	0.004768906	0.013729413	-	-	-	-	-	-
chr7	98553760	98554099	*TRRAP*	4	0.002804752	0.013643294	-	-	-	4	0.003570936	0.013551157	-	-	-	4	0.003053732	0.013727688	-	-	-	-	-	-
chr1	33336618	33337583	*FNDC5*	5	0.008757113	0.01410008	-	-	-	5	0.008492066	0.014096796	-	-	-	5	0.010546419	0.013936563	-	-	-	-	-	-
chr10	115613834	115614541	*DCLRE1A*																					
chr10	32344997	32346148	*KIF5B*	13	0.00610879	-0.002922341	-	-	-	-	-	-	-	-	-	13	0.006065736	-0.003263081	-	-	-	-	-	-
chr5	149339858	149340320	*SLC26A2*	11	0.004628907	-0.001849031	-	-	-	11	0.005133494	-0.001869002	-	-	-	-	-	-	-	-	-	-	-	-
chr5	161274307	161275588	*GABRA1*	17	0.0063673	-0.005053591	-	-	-	17	0.00772153	-0.005035077	-	-	-	17	0.009519622	-0.00484323	-	-	-	-	-	-
chr8	114448659	114450231	*CSMD3*	13	0.000514856	-0.003267874	-	-	-	-	-	-	-	-	-	-	-	-	-	-	-	-	-	-
chr5	154230173	154230438	*FAXDC2*	5	0.000257683	-0.016514345	-	-	-	5	0.000326825	-0.016048551	-	-	-	5	0.000550407	-0.015603017	5	0.003962356	-0.024910643	-	-	-
chr1	226288408	226288782	-	3	0.007981616	-0.007021031	-	-	-	3	0.009410795	-0.006997698	-	-	-	3	0.007843659	-0.007121422	-	-	-	-	-	-
chr5	111755039	111756360	*EPB41L4A*																					
chr19	18509358	18509973	*LRRC25*	3	0.001794452	0.018624529	-	-	-	3	0.00219932	0.018498153	-	-	-	3	0.002617515	0.018101287	-	-	-	-	-	-
chr17	1395371	1396298	*MYO1C*	8	0.000427454	0.012758271	-	-	-	-	-	-	-	-	-	-	-	-	-	-	-	-	-	-
chr6	105584149	105585490	*BVES*																					
chr21	40176506	40177189	*ETS2*	8	5.09E-05	0.011993308	-	-	-	8	7.13E-05	0.011886369	-	-	-	8	6.71E-05	0.011863368	-	-	-	-	-	-
chr13	61989037	61990113	*PCDH20*	16	0.003791048	-0.003079401	-	-	-	16	0.005005173	-0.00302962	-	-	-	16	0.004985426	-0.002882071	-	-	-	-	-	-
chr4	159592191	159593775	*C4orf46*																					
chr7	2149259	2150016	*MAD1L1*	4	0.008046205	0.001135409	-	-	-	4	0.009452805	0.001023146	-	-	-	4	0.007145147	0.001297754	-	-	-	-	-	-
chr12	26110666	26112124	*RASSF8*																					
chr14	97091902	97092205	-	3	0.008944509	0.013664536	-	-	-	3	0.010416427	0.013480848	-	-	-	3	0.009048978	0.013711997	-	-	-	-	-	-
chr9	138966848	138967872	*NACC2*	6	0.005329577	0.008081024	-	-	-	6	0.006840404	0.007921293	6	0.001444878	0.048429859	6	0.007594246	0.008130818	-	-	-	-	-	-
chr11	27744049	27744759	*BDNF*	7	0.002432485	-0.006307075	-	-	-	7	0.003245524	-0.006257122	-	-	-	7	0.003018659	-0.006201082	-	-	-	-	-	-
chr1	248902767	248903325	*LYPD8*	7	0.004752525	-0.012094993	-	-	-	7	0.005551646	-0.012080869	-	-	-	7	0.005595929	-0.012186202	-	-	-	-	-	-
chr19	52263233	52264429	*FPR2*	7	0.008145043	-0.000783526	-	-	-	7	0.010134659	-0.000701854	-	-	-	7	0.009431859	-0.000525224	-	-	-	-	-	-
chr3	121379745	121380792	*HCLS1*	9	0.004444748	-0.004802262	-	-	-	9	0.005251234	-0.004710646	-	-	-	9	0.005040226	-0.004789486	-	-	-	-	-	-
chr1	150521766	150522965	*ADAMTSL4*																					
chr6	31093846	31094557	*PSORS1C1*	5	0.008990778	0.008445181	-	-	-	5	0.011121377	0.008375544	-	-	-	5	0.012546706	0.008037744	-	-	-	-	-	-
chr16	85122558	85123778	*KIAA0513*	4	2.49E-05	0.007445622	-	-	-	4	3.23E-05	0.007462746	-	-	-	4	2.78E-05	0.007338914	-	-	-	-	-	-
chr16	89005476	89006877	*CBFA2T3*	11	0.007147894	-0.005133275	-	-	-	11	0.007068725	-0.005172884	-	-	-	11	0.006804723	-0.005180575	-	-	-	-	-	-
chr9	37029364	37030262	*PAX5*	4	0.008135031	0.003598777	-	-	-	4	0.009653328	0.003612432	-	-	-	4	0.009337987	0.003605828	-	-	-	-	-	-
chr8	626528	627809	*ERICH1*	8	0.000133136	-0.001020889	-	-	-	8	0.000168616	-0.000990921	-	-	-	8	9.85E-05	-0.00098173	-	-	-	-	-	-
chr11	110296837	110297121	*FDX1*	3	0.000173999	-0.019348452	-	-	-	3	0.000212732	-0.019288334	-	-	-	3	0.000211405	-0.019012017	-	-	-	-	-	-
chr19	17597326	17597999	*SLC27A1*	6	0.001883248	0.014178865	-	-	-	6	0.002153457	0.014132499	6	0.027051059	0.044768014	6	0.002184579	0.014332039	-	-	-	-	-	-
chr2	1291410	1291494	*SNTG2*	3	0.007999453	0.007898514	-	-	-	3	0.009675337	0.008006219	-	-	-	3	0.007609849	0.008146541	-	-	-	-	-	-
chr17	39184318	39184887	*KRTAP1-5*	4	0.000175861	-0.003619911	-	-	-	4	0.000183398	-0.003689287	-	-	-	4	0.00019223	-0.003657458	-	-	-	-	-	-
chr4	2427356	2428172	*CFAP99*	5	0.005678807	0.009378804	-	-	-	5	0.006373497	0.009330721	-	-	-	5	0.006213897	0.009519761	-	-	-	-	-	-
chr12	66275403	66276974	*HMGA2*	13	0.004588701	-0.007203961	-	-	-	13	0.006066103	-0.007123536	-	-	-	13	0.006445592	-0.007220783	-	-	-	-	-	-
chr2	233246431	233246922	*ALPP*	3	0.001726458	0.015183595	-	-	-	3	0.002115565	0.014785956	-	-	-	3	0.002371135	0.014818049	-	-	-	-	-	-
chr16	1198408	1199222	*CACNA1H*	6	0.006539868	0.008046679	-	-	-	6	0.006566086	0.008052165	-	-	-	6	0.006374193	0.00810991	-	-	-	-	-	-
chr2	50116848	50117117	-	3	0.005920898	-0.003675729	-	-	-	3	0.006830061	-0.003730363	3	0.029185187	-0.011982754	3	0.00412043	-0.003985383	-	-	-	-	-	-
chr4	177713813	177715040	*VEGFC*	3	0.0019275	-0.011819533	-	-	-	3	0.002378064	-0.011708748	-	-	-	3	0.002292937	-0.011651975	-	-	-	-	-	-
chr13	114282093	114282491	*TFDP1*	3	0.008318586	0.01277746	-	-	-	3	0.010270448	0.012579581	-	-	-	3	0.008012921	0.0128547	-	-	-	-	-	-
chr1	161128567	161130207	*UFC1*																					
chr13	88324169	88325393	*MIR4500HG*																					
chr11	22648074	22648618	*FANCF*	3	0.000606323	-0.006032088	-	-	-	3	0.000481048	-0.006132186	-	-	-	3	0.000644799	-0.006178532	-	-	-	-	-	-
chr10	80872926	80873797	*ZMIZ1*	3	0.000519548	0.016346406	3	0.003935307	0.024277194	3	0.000677034	0.016226683	-	-	-	3	0.000753441	0.016201771	-	-	-	-	-	-
chr14	104689833	104690882	-	8	0.001171434	-0.011112631	-	-	-	8	0.001412763	-0.010961275	-	-	-	8	0.001425866	-0.010862855	8	0.015115644	-0.019708587	-	-	-
chr5	180625089	180625956	*TRIM7*	7	0.008792188	0.005182481	7	0.002337317	0.008572994	-	-	-	-	-	-	-	-	-	-	-	-	-	-	-
chr11	2906981	2908471	*CDKN1C*	29	1.29E-07	0.006571949	-	-	-	29	1.37E-07	0.006619044	-	-	-	29	1.85E-07	0.006441154	-	-	-	-	-	-
chr20	49574938	49575492	*DPM1*																					
chr6	168122077	168123805	-	9	0.006884677	0.001047006	-	-	-	9	0.008175319	0.001033678	-	-	-	9	0.007639183	0.001078722	-	-	-	-	-	-
chr5	6632973	6633577	*NSUN2*																					
chr17	73043062	73043736	*ATP5PD*																					
chr13	103425389	103426051	*TEX30*	5	0.00289796	-0.00361871	-	-	-	-	-	-	-	-	-	-	-	-	-	-	-	-	-	-
chr13	36048892	36051073	*MIR548F5*																					
chr16	11723154	11724177	-	4	0.00185391	-0.009767469	-	-	-	4	0.002343968	-0.009672426	-	-	-	4	0.002681009	-0.009556233	-	-	-	-	-	-
chr13	113897950	113898827	*CUL4A*	3	0.002242266	0.004934597	-	-	-	3	0.002527967	0.004964297	-	-	-	3	0.002245161	0.005121001	-	-	-	-	-	-

chr1	15911089	15912213	*AGMAT*																					
chr1	228331879	228332502	*GJC2*																					
chr14	23478970	23479801	*C14orf93*	7	0.008869115	0.007228543	-	-	-	7	0.009822129	0.007226963	-	-	-	7	0.006460509	0.007231527	-	-	-	-	-	-
chr6	28863395	28864832	-	30	0.00877868	-0.004176422	-	-	-	30	0.012920373	-0.004178063	-	-	-	30	0.008374637	-0.004283897	-	-	-	-	-	-
chr1	38259884	38260410	*MANEAL*	5	0.007144141	0.003745892	-	-	-	5	0.008547891	0.003735194	-	-	-	5	0.007842297	0.003671477	-	-	-	-	-	-
chr1	3527627	3528682	*MEGF6*	12	0.000246624	-0.010274954	-	-	-	12	0.000359349	-0.010274753	-	-	-	12	0.000207456	-0.010432585	-	-	-	-	-	-
chr15	91497412	91498367	*RCCD1*	11	0.007395313	-0.001568275	-	-	-	11	0.008312633	-0.001558747	-	-	-	11	0.008489402	-0.001592798	-	-	-	-	-	-
chr3	114012316	114012912	*TIGIT*	5	0.006230947	0.011742083	-	-	-	5	0.007631244	0.011638268	-	-	-	5	0.007695616	0.011291988	-	-	-	-	-	-
chr3	88107415	88107754	*CGGBP1*	3	0.003041539	-0.002314554	-	-	-	3	0.00380714	-0.002307924	-	-	-	3	0.002909886	-0.00226371	-	-	-	-	-	-
chr17	74524371	74525597	*CYGB*																					
chr10	134120448	134121712	*STK32C*	11	0.001133681	-0.010343345	-	-	-	11	0.001568612	-0.010291582	-	-	-	11	0.001106754	-0.010625406	-	-	-	-	-	-
chr8	22560922	22561719	-	6	0.004497392	0.015271101	-	-	-	6	0.004714391	0.015324433	-	-	-	6	0.004616407	0.015370305	-	-	-	-	-	-
chr10	13204003	13204696	*MCM10*	4	0.001444987	0.006916943	-	-	-	4	0.00182897	0.00694539	-	-	-	4	0.001599109	0.006947271	-	-	-	-	-	-
chr17	19290353	19291120	*MFAP4*	8	0.002147073	-0.007909697	-	-	-	8	0.002913612	-0.007825988	-	-	-	8	0.004084287	-0.007555615	-	-	-	-	-	-
chr17	15848828	15849556	*ADORA2B*	3	0.008898288	0.004473505	-	-	-	3	0.009990747	0.004421693	-	-	-	3	0.008814264	0.004429008	-	-	-	-	-	-
chr2	220378659	220379968	*ASIC4*	13	0.004842948	0.000545513	-	-	-	13	0.006696515	0.000552535	-	-	-	13	0.006027548	0.000608079	-	-	-	-	-	-
chr7	42942369	42942901	-	4	0.003423605	-0.019080472	-	-	-	4	0.003712381	-0.019081954	-	-	-	4	0.004468645	-0.018888386	-	-	-	-	-	-
chr1	3182620	3183072	*PRDM16*	3	0.005249693	0.003926822	-	-	-	3	0.006260792	0.003775692	-	-	-	3	0.005644964	0.003587878	-	-	-	-	-	-
chr3	52091063	52091829	*DUSP7*	6	0.007242981	0.001641669	-	-	-	6	0.008035371	0.001654461	6	0.000554263	0.009566835	6	0.007561466	0.001580956	6	0.002282331	-0.000651244	-	-	-
chr1	24306751	24307535	*SRSF10*	13	0.00078831	0.000805834	-	-	-	-	-	-	-	-	-	13	0.000519088	0.000823711	-	-	-	-	-	-
chr16	10674008	10675123	*EMP2*	12	0.005420747	-0.001539391	-	-	-	12	0.006468074	-0.001554116	-	-	-	12	0.005844299	-0.001621767	-	-	-	-	-	-
chr20	25603976	25606029	*NANP*	19	0.002116844	0.003569033	-	-	-	19	0.002696255	0.003554019	-	-	-	19	0.002137748	0.003610548	-	-	-	-	-	-
chr17	59493993	59494444	-	5	0.003011281	-0.003318718	-	-	-	5	0.003572616	-0.00321582	-	-	-	5	0.004106564	-0.003170569	-	-	-	-	-	-
chr5	405488	406046	*AHRR*	4	0.001960872	-0.012431706	-	-	-	4	0.001420437	-0.012575736	-	-	-	4	0.001718433	-0.012515294	-	-	-	-	-	-
chr17	73029893	73031159	*KCTD2*	6	0.002863317	0.002617373	-	-	-	6	0.00324994	0.002586995	-	-	-	6	0.003808029	0.002544995	-	-	-	-	-	-
chr10	90984672	90985062	*LIPA*	3	1.55E-05	0.049841417	3	0.02746289	0.045071429	3	2.10E-05	0.049294161	3	0.015042613	0.137645564	3	2.37E-05	0.049361175	-	-	-	-	-	-
chr4	646853	648752	*PDE6B*	10	0.000205003	0.004177887	-	-	-	10	0.000285799	0.004074178	-	-	-	10	0.000228952	0.00386911	-	-	-	-	-	-
chr13	50703026	50703841	*DLEU1*																					
chr21	34755681	34756016	-	4	0.009459588	-0.00441657	4	0.01398969	-0.005087554	4	0.011360214	-0.004390675	-	-	-	4	0.009825096	-0.004454831	-	-	-	-	-	-
chr2	237088462	237088919	-	3	0.001173569	-0.007724607	-	-	-	3	0.001436466	-0.007672485	-	-	-	3	0.001535829	-0.007417545	-	-	-	-	-	-
chr22	30969767	30970729	*GAL3ST1*	4	0.004744609	-0.030899037	-	-	-	4	0.005733657	-0.030732817	-	-	-	4	0.006111467	-0.029798596	4	0.000406185	-0.062223645	-	-	-
chr17	21186789	21187553	*MAP2K3*	4	0.003414866	0.012664119	-	-	-	4	0.003959189	0.012738767	-	-	-	4	0.003194523	0.012872763	-	-	-	-	-	-
chr2	218898491	218898788	*RUFY4*	3	0.006132479	0.017592625	3	0.000211595	0.026884936	3	0.007478065	0.017479349	-	-	-	3	0.005280788	0.017897122	-	-	-	-	-	-
chr17	79817963	79819480	*P4HB*	14	0.00199577	0.000374261	-	-	-	14	0.002378064	0.000358069	-	-	-	14	0.00227372	0.000251219	-	-	-	-	-	-
chr13	44947076	44948827	*SERP2*	10	0.003382955	0.003730649	-	-	-	-	-	-	-	-	-	10	0.002544937	0.003797076	-	-	-	-	-	-
chr10	21462441	21463858	*NEBL*																					
chr3	62860802	62861925	*CADPS*	13	0.0031751	-0.00218122	-	-	-	-	-	-	-	-	-	13	0.004788811	-0.001816918	-	-	-	-	-	-
chr12	97300410	97301661	*NEDD1*	18	0.000254566	-0.002022227	-	-	-	18	0.00026522	-0.001988003	-	-	-	18	0.000195524	-0.001936584	-	-	-	-	-	-
chr5	146256776	146257484	*PPP2R2B*	4	0.008121305	-0.000452736	-	-	-	4	0.008836699	-0.000495321	-	-	-	4	0.006774169	-0.000344141	-	-	-	-	-	-
chr19	8273505	8274513	*CERS4*	14	0.001553649	-0.001281061	-	-	-	14	0.002172587	-0.001364269	14	0.011231563	-0.018041954	14	0.001079198	-0.00136269	-	-	-	-	-	-
chr6	41374532	41375153	-	4	0.007534749	0.031350482	-	-	-	4	0.006800941	0.031751457	-	-	-	4	0.006183738	0.03201517	-	-	-	-	-	-
chr6	26757644	26758395	-	5	0.001619249	-0.012179607	-	-	-	5	0.00182367	-0.012095891	-	-	-	5	0.002648367	-0.011642151	-	-	-	-	-	-
chr8	140945782	140945843	*TRAPPC9*	3	0.004973109	-0.014891665	-	-	-	3	0.005948612	-0.014843714	-	-	-	3	0.006328856	-0.014665452	-	-	-	-	-	-
chr5	113697330	113698664	*KCNN2*	14	0.009007072	-0.005956093	-	-	-	-	-	-	-	-	-	-	-	-	-	-	-	-	-	-
chr11	132662455	132662963	*OPCML*	4	0.000910498	-0.04346194	-	-	-	4	0.00118181	-0.043269023	-	-	-	4	0.000514964	-0.044539039	-	-	-	-	-	-
chr15	89377583	89378209	*ACAN*	3	0.002919833	0.019349831	-	-	-	-	-	-	-	-	-	3	0.002749609	0.019594372	-	-	-	-	-	-
chr20	21105829	21106966	*KIZ*	12	0.002888886	-0.010526045	-	-	-	12	0.003431975	-0.010579021	-	-	-	12	0.003101713	-0.010652698	-	-	-	-	-	-
chr1	236095453	236096728	-	3	0.000765938	-0.020264496	-	-	-	3	0.000794428	-0.020292218	-	-	-	3	0.000913922	-0.02017022	-	-	-	-	-	-
chr19	33622856	33623230	*WDR88*	10	0.008318586	0.016645512	-	-	-	10	0.011067847	0.016472788	-	-	-	-	-	-	-	-	-	-	-	-
chr10	23479993	23480237	*PTF1A*	4	0.005428548	-0.010605902	-	-	-	4	0.005769213	-0.01061709	-	-	-	4	0.007123176	-0.010463596	-	-	-	-	-	-
chr17	170770	171948	*RPH3AL*	11	0.004846122	0.02203999	-	-	-	11	0.006560985	0.021800614	-	-	-	-	-	-	-	-	-	-	-	-
chr10	102469807	102470340	-	4	0.009842118	-0.005413929	-	-	-	4	0.011100679	-0.005454677	-	-	-	4	0.011034795	-0.005358398	-	-	-	-	-	-
chr1	16553267	16554984	-	10	6.05E-06	0.019310538	-	-	-	10	8.46E-06	0.019304408	-	-	-	10	5.53E-06	0.019719992	-	-	-	-	-	-
chr20	1471884	1472712	*SIRPB2*	7	0.008887447	-0.006013614	-	-	-	7	0.011633235	-0.005943664	-	-	-	7	0.013963192	-0.005833352	-	-	-	-	-	-
chr14	105766064	105767520	*BRF1*																					
chr5	149682233	149683334	*ARSI*	9	0.001142194	0.000570528	-	-	-	9	0.001365597	0.000584766	-	-	-	9	0.001353913	0.000338175	-	-	-	-	-	-
chr7	106685033	106686041	*PRKAR2B*	4	0.000124229	0.004321165	-	-	-	-	-	-	-	-	-	4	0.000135426	0.004497476	-	-	-	-	-	-
chr7	24797102	24798855	*GSDME*	16	0.000821295	0.009469819	-	-	-	16	0.000982339	0.009475491	-	-	-	16	0.000895402	0.009455917	-	-	-	-	-	-
chr1	16810670	16810776	*CROCCP3*	3	0.001316166	0.018648435	-	-	-	3	0.001586466	0.018586435	-	-	-	3	0.001599109	0.01872704	3	0.033517899	0.021552674	-	-	-
chr9	21994752	21996207	*CDKN2A*																					
chr5	95196871	95197476	-	5	0.004884088	-0.014986083	-	-	-	5	0.005716136	-0.01490165	-	-	-	5	0.00581408	-0.014520992	-	-	-	-	-	-
chr4	20985623	20986246	*KCNIP4*	7	0.005256458	-0.012610029	-	-	-	7	0.006482093	-0.012543194	-	-	-	7	0.00633009	-0.0124336	-	-	-	-	-	-
chr3	183893065	183894778	*AP2M1*	8	0.004525259	0.008621123	-	-	-	8	0.005495374	0.008571381	-	-	-	8	0.00389166	0.008672952	-	-	-	-	-	-
chr8	145082140	145082728	*SPATC1*	3	0.006049135	0.006038664	-	-	-	3	0.007006445	0.005992043	-	-	-	3	0.007709085	0.005737554	-	-	-	-	-	-
chr6	88031886	88032848	*GJB7*																					
chr15	80444736	80445874	*FAH*	11	0.004436136	-0.01008025	-	-	-	11	0.005176025	-0.010101836	-	-	-	11	0.003964281	-0.010207629	-	-	-	-	-	-
chr12	133405852	133406309	*GOLGA3*	3	0.007396854	-0.001777497	-	-	-	3	0.00891097	-0.001904626	-	-	-	3	0.008431733	-0.001363365	-	-	-	-	-	-
chr20	9496625	9497330	*LAMP5*	3	0.002954111	-0.004044388	-	-	-	3	0.003215901	-0.004108455	-	-	-	3	0.003945646	-0.003964832	-	-	-	-	-	-
chr3	29377160	29377980	*RBMS3*	3	2.42E-05	-0.044645175	-	-	-	3	2.83E-05	-0.044011851	-	-	-	3	2.91E-05	-0.044669096	-	-	-	-	-	-
chr19	52900469	52901639	*ZNF528*	15	4.69E-06	0.006466295	-	-	-	15	6.37E-06	0.006450617	-	-	-	15	2.44E-06	0.006837631	-	-	-	-	-	-
chr3	154796701	154798079	*MME*	18	0.00977705	-0.006453693	-	-	-	-	-	-	-	-	-	-	-	-	-	-	-	-	-	-
chr4	101596281	101596970	*LINC01216*	6	0.006798355	0.01410541	-	-	-	6	0.007684808	0.014095429	-	-	-	6	0.006856717	0.014243568	-	-	-	-	-	-
chr17	75312165	75313094	*SEPTIN9*	3	0.001482685	0.019177638	-	-	-	3	0.001779121	0.019117623	3	0.041962188	0.05675445	3	0.001405064	0.019100039	-	-	-	-	-	-
chr13	76334000	76334866	*LMO7*	7	0.000307843	-0.014092738	-	-	-	7	0.000261072	-0.014227263	-	-	-	-	-	-	-	-	-	-	-	-
chr10	13481846	13482639	*BEND7*	5	0.000855984	-0.018909877	-	-	-	-	-	-	-	-	-	-	-	-	-	-	-	-	-	-
chr19	14591033	14591345	*GIPC1*																					
chr18	74535233	74535991	*ZNF236*																					
chr7	19812570	19813525	*TMEM196*	15	0.008624604	-0.005540911	-	-	-	-	-	-	-	-	-	-	-	-	-	-	-	-	-	-
chr2	231732669	231734563	*ITM2C*	4	0.000987439	0.018527269	-	-	-	4	0.001249194	0.018391945	-	-	-	4	0.00121397	0.018647557	-	-	-	-	-	-
chr14	50910667	50910968	*MAP4K5*	3	0.002872541	-0.020582222	-	-	-	3	0.003622367	-0.020266494	-	-	-	3	0.003674887	-0.020266014	3	0.040033561	-0.028609541	-	-	-
chr10	128994297	128995478	*DOCK1*																					
chr6	155450088	155450882	*TIAM2*	4	0.005413381	-0.00622527	-	-	-	-	-	-	-	-	-	4	0.004052768	-0.006451561	-	-	-	-	-	-
chr13	111805314	111805930	*ARHGEF7*	7	0.009838622	0.006373182	-	-	-	7	0.011794876	0.006377729	7	0.011588594	0.022911613	7	0.008117059	0.006575626	-	-	-	-	-	-
chr1	47644094	47646024	*LINC00853*	12	0.005047379	-0.003103402	-	-	-	12	0.00593399	-0.0030118	-	-	-	12	0.003316914	-0.003276768	-	-	-	-	-	-
chr12	54349169	54350294	*HOXC12*	6	0.004708896	-0.013152947	-	-	-	6	0.005705336	-0.013268611	-	-	-	6	0.006563432	-0.012825387	-	-	-	-	-	-
chr14	105885531	105886413	*MTA1*	11	0.001264752	-0.019077462	-	-	-	11	0.001558947	-0.019203715	-	-	-	11	0.000945653	-0.019474126	-	-	-	-	-	-
chr8	146228110	146228920	*ZNF252P*																					
chr1	231555522	231556682	*EGLN1*	5	0.000726622	-0.007223957	-	-	-	5	0.00092777	-0.007194395	-	-	-	5	0.000956983	-0.006990332	-	-	-	-	-	-
chr7	114561493	114562847	*MDFIC*	12	0.004724054	0.009733669	-	-	-	12	0.005293389	0.009747202	-	-	-	12	0.005400352	0.00952979	-	-	-	-	-	-
chr20	19193824	19194649	*SLC24A3*	7	7.84E-06	0.014119857	7	1.24E-06	0.01786724	7	6.92E-06	0.014357135	-	-	-	7	3.82E-06	0.014292772	-	-	-	-	-	-
chr6	30411932	30412351	-	4	1.02E-07	-0.030533181	4	0.027842315	-0.020196303	4	9.60E-08	-0.030769699	-	-	-	4	1.07E-07	-0.030695694	-	-	-	-	-	-
chr9	139903316	139904151	*ABCA2*	5	0.000391992	0.017082889	-	-	-	5	0.000449593	0.016939978	-	-	-	5	0.000557224	0.017063097	-	-	-	-	-	-
chr4	73178533	73179086	*ADAMTS3*	3	0.006426401	0.005290326	-	-	-	3	0.006847857	0.005264634	-	-	-	3	0.00774143	0.005000212	-	-	-	-	-	-
chr18	31802071	31803067	*NOL4*	10	1.90E-05	-0.003367162	10	0.000218636	-0.002377066	10	2.80E-05	-0.003354078	-	-	-	10	3.28E-05	-0.003253295	-	-	-	-	-	-
chr1	86861147	86862273	*ODF2L*	14	0.003918394	-0.001396349	-	-	-	14	0.005390762	-0.001420541	-	-	-	-	-	-	-	-	-	-	-	-
chr7	93550752	93551275	*GNG11*	9	0.001282922	-0.010267093	9	0.006321858	-0.010916041	9	0.001526773	-0.010240645	-	-	-	9	0.001069013	-0.010361588	-	-	-	-	-	-
chr3	45076240	45077254	*CLEC3B*	5	0.009160197	0.002779363	-	-	-	5	0.010752101	0.002777642	-	-	-	5	0.009768904	0.002650962	-	-	-	-	-	-
chr19	50269440	50270616	*AP2A1*																					
chr21	43815685	43816355	*TMPRSS3*	7	0.004961301	-0.000265747	-	-	-	7	0.005290551	-0.000342946	-	-	-	7	0.004555741	-0.000393177	-	-	-	-	-	-
chr17	48919431	48920013	*WFIKKN2*	3	0.001847995	0.004832951	-	-	-	-	-	-	-	-	-	-	-	-	-	-	-	-	-	-
chr10	60936025	60937257	*PHYHIPL*	19	0.002712832	-0.007613308	-	-	-	-	-	-	-	-	-	-	-	-	-	-	-	-	-	-
chr1	151019727	151020868	*BNIPL*																					
chr3	143691715	143692356	*DIPK2A*	12	0.007753037	0.003560512	-	-	-	12	0.007958162	0.003569538	-	-	-	12	0.009107721	0.003559201	12	0.014501442	0.006070056	-	-	-
chr19	536244	536929	*CDC34*	5	0.004943453	0.006425425	-	-	-	5	0.006017162	0.006360804	-	-	-	5	0.005512029	0.006460169	-	-	-	-	-	-
chr19	4769001	4769688	*MIR7-3*																					
chr22	43043757	43044788	*CYB5R3*	5	0.000165353	0.010845309	-	-	-	5	0.000199099	0.010862747	-	-	-	5	0.000216023	0.0108109	-	-	-	-	-	-
chr4	737005	738172	*PCGF3*	6	0.004970095	-0.00620202	-	-	-	-	-	-	-	-	-	6	0.004147251	-0.006191132	-	-	-	-	-	-
chr2	225265564	225266960	*FAM124B*	12	2.33E-05	-0.007677548	-	-	-	12	2.96E-05	-0.007669033	-	-	-	12	4.72E-05	-0.007392916	-	-	-	-	-	-
chr1	44399069	44401857	*ARTN*	18	0.001451911	0.008123908	-	-	-	-	-	-	-	-	-	-	-	-	-	-	-	-	-	-
chr1	47184410	47185236	*EFCAB14*	13	0.004868288	0.000522267	-	-	-	13	0.006141959	0.000525834	-	-	-	13	0.005633114	0.00052639	-	-	-	-	-	-
chr7	54609534	54610192	*VSTM2A*	12	0.002313962	-0.007005297	12	7.03E-05	-0.012838437	12	0.002885188	-0.006943761	-	-	-	12	0.003603731	-0.006690969	-	-	-	-	-	-

chr20	61446962	61447929	*COL9A3*	29	3.41E-05	-0.008813399	-	-	-	29	2.88E-05	-0.00897678	-	-	-	29	1.21E-05	-0.009134374	-	-	-	-	-	-
chr16	1429545	1430367	*UNKL*	9	0.004651278	0.012420866	-	-	-	9	0.006139372	0.012216576	9	0.005771423	0.044818216	9	0.005551167	0.012188231	-	-	-	-	-	-
chr19	13133343	13133825	*NFIX*	4	0.006644878	-0.010886422	-	-	-	4	0.008172091	-0.01069771	-	-	-	4	0.010096185	-0.010396807	-	-	-	-	-	-
chr3	46447826	46449636	*CCRL2*	16	0.00188161	-0.008087392	16	0.000475417	-0.00950657	16	0.002266851	-0.008055501	-	-	-	16	0.002456557	-0.008053734	-	-	-	-	-	-
chr2	24269866	24270726	*FKBP1B*																					
chr15	71952725	71953221	*THSD4*	3	0.007414928	0.012925101	-	-	-	3	0.008087713	0.012869099	-	-	-	3	0.008058655	0.012777679	-	-	-	-	-	-
chr6	33178942	33180841	*RING1*	16	0.007994361	0.003869618	-	-	-	-	-	-	-	-	-	-	-	-	-	-	-	-	-	-
chr6	119401102	119401660	*FAM184A*	3	0.004149975	0.00107533	-	-	-	3	0.004920276	0.00118735	-	-	-	3	0.004856006	0.000764727	-	-	-	-	-	-
chr6	32117379	32123701	*LOC100507547*																					
chr8	1892271	1892956	*ARHGEF10*	3	0.000799484	0.028709949	-	-	-	-	-	-	-	-	-	3	0.000992996	0.028936815	-	-	-	-	-	-
chr17	38472961	38473789	*RARA*	6	0.000666195	0.016650312	-	-	-	6	0.000555221	0.01679812	-	-	-	6	0.000660339	0.016826115	-	-	-	-	-	-
chr12	104443742	104444435	*GLT8D2*	11	0.000611341	-0.009642876	-	-	-	11	0.000783553	-0.009648778	-	-	-	11	0.000859419	-0.009576292	-	-	-	-	-	-
chr12	46766294	46767289	*SLC38A2*	9	0.001578568	-0.008626276	-	-	-	9	0.002146279	-0.008686687	-	-	-	9	0.001157149	-0.008878478	-	-	-	-	-	-
chr7	157646656	157647847	*PTPRN2*																					
chr5	150325582	150326642	*ZNF300P1*	11	0.008482686	-0.003210582	-	-	-	11	0.009927301	-0.003197523	-	-	-	-	-	-	-	-	-	-	-	-
chr2	26986235	26986550	*SLC35F6*	5	0.007912581	0.002435292	-	-	-	5	0.008956832	0.002338498	-	-	-	5	0.005910397	0.002649456	-	-	-	-	-	-
chr19	2545986	2547067	*GNG7*	5	0.000220699	0.025499378	-	-	-	5	0.000104493	0.026006231	-	-	-	5	5.36E-05	0.02639914	-	-	-	-	-	-
chr1	48191436	48191880	-	3	0.005440383	-0.013935714	-	-	-	3	0.005793886	-0.014024332	-	-	-	3	0.00724882	-0.013670888	-	-	-	-	-	-
chr15	28341068	28342183	*OCA2*	9	0.009642893	0.001451889	-	-	-	-	-	-	-	-	-	9	0.010297946	0.001448751	-	-	-	-	-	-
chr4	7048059	7048926	*CCDC96*																					
chr12	125478229	125478868	*BRI3BP*																					
chr1	151103642	151105478	*SEMA6C*	11	6.64E-06	0.019181781	-	-	-	11	7.29E-06	0.019242502	-	-	-	11	7.37E-06	0.019359498	-	-	-	-	-	-
chr18	13824072	13824396	*MC5R*	5	0.005768042	-0.004189467	-	-	-	5	0.00725503	-0.004171547	-	-	-	5	0.004179583	-0.004382189	-	-	-	-	-	-
chr18	24764966	24765880	*AQP4-AS1*																					
chr1	41444578	41445793	*CTPS1*	19	0.002760386	0.002683446	-	-	-	19	0.004007919	0.00265312	-	-	-	19	0.002674578	0.00268136	-	-	-	-	-	-
chr6	133561368	133562776	*EYA4*	36	6.31E-05	-0.007741311	-	-	-	36	0.000126825	-0.007623778	-	-	-	36	0.000142362	-0.00759311	-	-	-	-	-	-
chr12	133083102	133083593	*FBRSL1*	4	0.002323066	-0.016576341	-	-	-	4	0.003002484	-0.01624645	-	-	-	4	0.00348605	-0.016204169	-	-	-	-	-	-
chr10	103648988	103649718	*ARMH3*	3	0.006692778	-0.00477993	-	-	-	3	0.008006449	-0.004813531	-	-	-	3	0.0076305	-0.004690957	-	-	-	-	-	-
chr1	154377091	154377621	*IL6R*	9	0.005736902	-0.007327411	-	-	-	9	0.007270836	-0.007320797	-	-	-	9	0.00581408	-0.007450759	-	-	-	-	-	-
chr6	7142024	7142638	*RREB1*	5	0.008773128	0.015938608	-	-	-	5	0.009896487	0.015960709	5	0.012900705	0.056016879	5	0.009014494	0.016179995	-	-	-	5	0.021336037	0.06717488
chr20	55835831	55836676	*BMP7*	4	4.51E-07	-0.048500957	4	0.000330236	-0.049874076	4	6.14E-07	-0.048256276	-	-	-	4	5.95E-07	-0.04841495	-	-	-	-	-	-
chr6	35479910	35480779	*TULP1*	7	0.002024834	0.010398422	-	-	-	7	0.002543928	0.010318712	-	-	-	7	0.001833672	0.010508659	-	-	-	-	-	-
chr3	173301431	173303193	*NLGN1*	8	0.004409666	-0.008662872	-	-	-	-	-	-	-	-	-	-	-	-	-	-	-	-	-	-
chr4	154680314	154680909	*RNF175*	3	0.003097489	0.014669978	-	-	-	3	0.003726925	0.014694619	-	-	-	3	0.00392471	0.014615553	-	-	-	-	-	-
chr4	726697	728041	*PCGF3*	5	0.001976337	-0.00594751	-	-	-	5	0.002303704	-0.005823744	-	-	-	5	0.002540794	-0.005782032	-	-	-	-	-	-
chr9	14346276	14347122	*NFIB*	4	0.003009022	0.001623486	-	-	-	-	-	-	-	-	-	4	0.003231713	0.001792718	-	-	-	-	-	-
chr9	128651435	128652652	*PBX3*	5	0.000156635	-0.017219436	-	-	-	5	0.000186588	-0.017006332	-	-	-	5	0.000168632	-0.017045896	-	-	-	-	-	-
chr12	113435438	113435980	*OAS2*	3	0.001813939	0.011406856	-	-	-	3	0.001595863	0.011521475	-	-	-	3	0.000878198	0.011818034	-	-	-	-	-	-
chr17	75789279	75790091	-	6	0.000425602	-0.027130191	-	-	-	6	0.00054108	-0.026585966	-	-	-	6	0.000567668	-0.026938398	-	-	-	-	-	-
chr21	46954058	46955202	*SLC19A1*	5	0.000860372	-0.006358256	-	-	-	5	0.001135445	-0.006358622	-	-	-	5	0.001239346	-0.006140355	-	-	-	-	-	-
chr2	25527080	25527366	*DNMT3A*	3	0.002810546	-0.01164316	-	-	-	3	0.003294059	-0.01152749	-	-	-	3	0.003652248	-0.011256463	-	-	-	-	-	-
chr13	46189838	46190198	*ERICH6B*	9	0.002128475	-0.004821478	-	-	-	9	0.002664022	-0.004699517	-	-	-	9	0.002851336	-0.004452803	-	-	-	-	-	-
chr6	170596856	170598215	*DLL1*	7	0.005254784	-0.015744034	-	-	-	7	0.004406731	-0.016061408	-	-	-	7	0.007079479	-0.015569211	-	-	-	-	-	-
chr3	9773022	9773672	*BRPF1*	6	0.001304477	-0.004403431	-	-	-	6	0.001387983	-0.004390214	-	-	-	6	0.000711392	-0.004532466	-	-	-	-	-	-
chr22	42196285	42197038	*CCDC134*	11	0.005338902	-0.002075806	-	-	-	11	0.006254809	-0.002108813	11	0.005668312	-0.00890323	11	0.005136041	-0.002319302	-	-	-	-	-	-
chr13	28712950	28713767	*PAN3*																					
chr5	73937141	73937762	*ENC1*																					
chr9	130639739	130640786	*AK1*	12	5.76E-05	0.008904248	-	-	-	12	6.50E-05	0.008975988	-	-	-	12	2.98E-05	0.009114073	-	-	-	-	-	-
chr2	43385231	43386412	-	3	0.000594763	-0.012381288	-	-	-	3	0.000700486	-0.01234823	-	-	-	3	0.000650624	-0.012463365	-	-	-	-	-	-
chr8	6263875	6264959	*LOC100287015*																					
chr3	49130732	49131405	*QRICH1*	7	0.009438936	-0.006111613	-	-	-	7	0.010822669	-0.006143428	-	-	-	-	-	-	-	-	-	-	-	-
chr2	127412692	127414108	*GYPC*	13	0.000776949	-0.004884669	-	-	-	13	0.001166695	-0.004788579	13	0.005252921	-0.008890721	13	0.001167481	-0.004722516	-	-	-	-	-	-
chr8	145024637	145025178	*PLEC*	7	0.006477839	0.011303763	-	-	-	7	0.007924386	0.011237424	-	-	-	7	0.008243369	0.011210298	7	0.010275215	0.016382157	-	-	-
chr16	2053627	2054287	*ZNF598*	5	0.003172953	0.020026757	-	-	-	5	0.003647581	0.020043044	-	-	-	5	0.003204022	0.020146794	-	-	-	-	-	-
chr22	37499853	37500722	*TMPRSS6*	6	0.003000427	0.004911746	-	-	-	-	-	-	-	-	-	6	0.003215201	0.004894413	-	-	-	-	-	-
chr14	23503697	23504921	*PSMB5*	19	0.008300777	-0.003849143	-	-	-	19	0.011126194	-0.003849943	-	-	-	19	0.008723993	-0.003900877	-	-	-	-	-	-
chr3	15642568	15643903	*BTD*																					
chr21	46291868	46292710	*PTTG1IP*	4	0.003753905	-0.010944025	-	-	-	4	0.004896302	-0.010825846	-	-	-	-	-	-	-	-	-	-	-	-
chr5	171056823	171057575	-	7	9.24E-06	0.036184758	-	-	-	7	1.02E-05	0.036352722	-	-	-	7	1.24E-05	0.035936429	7	0.000217002	0.049984711	-	-	-
chr13	34256111	34256553	-	3	0.002214077	0.012619361	-	-	-	3	0.00269992	0.012585028	-	-	-	3	0.002099515	0.012971874	-	-	-	-	-	-
chr11	20229517	20230330	-	5	0.005532102	-0.017087287	-	-	-	5	0.006240484	-0.017015005	-	-	-	5	0.007107039	-0.01683258	-	-	-	-	-	-
chr6	75982940	75983278	*TMEM30A*	3	0.007127134	-0.015766057	-	-	-	3	0.007869869	-0.015825327	-	-	-	3	0.009636811	-0.015310399	-	-	-	-	-	-
chr16	84650990	84651521	*COTL1*	4	0.004263413	0.00161005	-	-	-	4	0.005068404	0.00161429	-	-	-	4	0.00526474	0.001537364	-	-	-	-	-	-
chr16	71929343	71930048	*IST1*	8	0.009962353	-0.001615384	-	-	-	-	-	-	-	-	-	-	-	-	-	-	-	-	-	-
chr13	77460298	77461426	*KCTD12*	15	0.001955796	0.00233776	-	-	-	15	0.002448145	0.00233473	-	-	-	15	0.001260339	0.002439873	-	-	-	-	-	-
chr17	68164127	68166241	*KCNJ2*																					
chr16	595486	596185	*CAPN15*	8	0.007977565	0.012733758	-	-	-	8	0.010299278	0.012637216	-	-	-	8	0.00769974	0.01292687	-	-	-	-	-	-
chr17	38023480	38024636	*IKZF3*																					
chr20	62084549	62084833	*KCNQ2*	3	5.29E-05	-0.036569781	-	-	-	3	6.91E-05	-0.036396873	-	-	-	3	4.09E-05	-0.03706915	3	0.004744438	-0.051033493	-	-	-
chr10	65388604	65389292	-	4	0.003309315	-0.008241175	-	-	-	-	-	-	-	-	-	-	-	-	-	-	-	-	-	-
chr18	48345906	48346843	*MRO*	11	0.005211665	-0.007327893	-	-	-	-	-	-	-	-	-	-	-	-	-	-	-	-	-	-
chr6	110796274	110796813	*SLC22A16*	3	0.002900125	-0.014363393	-	-	-	3	0.003445423	-0.014342137	-	-	-	3	0.003337928	-0.014359595	-	-	-	-	-	-
chr9	136844397	136844768	*VAV2*	4	0.003191795	0.030025308	4	0.01362301	0.037457919	4	0.003817187	0.029942754	-	-	-	4	0.001408862	0.031310689	-	-	-	-	-	-
chr19	37328842	37329859	*ZNF790*	17	0.004761136	-7.89E-05	-	-	-	17	0.006054357	-0.000111536	-	-	-	17	0.005962992	0.000105042	-	-	-	-	-	-
chr14	103243389	103244199	*TRAF3*	3	0.008499527	-0.020719442	-	-	-	3	0.009511182	-0.021026938	-	-	-	3	0.007721637	-0.021074656	-	-	-	-	-	-
chr6	33770212	33772366	*MLN*	10	1.96E-05	0.017570121	-	-	-	10	2.42E-05	0.017518425	-	-	-	10	2.24E-05	0.017744176	-	-	-	-	-	-
chr11	98890677	98892283	*CNTN5*	15	0.00063029	-0.003347439	-	-	-	-	-	-	-	-	-	-	-	-	-	-	-	-	-	-
chr9	91148748	91149860	*NXNL2*	5	0.00324399	-0.008378808	-	-	-	5	0.003878472	-0.008263117	-	-	-	5	0.003957992	-0.008476825	-	-	-	-	-	-
chr2	239046275	239047336	*KLHL30*	8	0.007404473	0.007593885	-	-	-	8	0.009413493	0.007516838	-	-	-	8	0.009989641	0.007137894	-	-	-	-	-	-
chr12	22093960	22095182	*ABCC9*	15	0.003668182	0.003256874	-	-	-	15	0.004285445	0.003246601	-	-	-	15	0.003953591	0.003422076	-	-	-	-	-	-
chr7	140103619	140104222	*RAB19*	8	0.005576911	-0.007868847	-	-	-	8	0.006881783	-0.007730763	-	-	-	8	0.004966297	-0.008004283	-	-	-	-	-	-
chr6	166401018	166402753	*LINC00473*																					
chr6	71123675	71124075	*FAM135A*	6	0.009842673	-0.011999235	-	-	-	6	0.012864788	-0.0119497	-	-	-	-	-	-	-	-	-	-	-	-
chr12	121976655	121976833	*KDM2B*	3	0.002645476	0.014906072	3	0.01595648	0.015137559	3	0.00283959	0.014778654	-	-	-	3	0.00351268	0.014488048	-	-	-	-	-	-
chr20	3051493	3052483	*OXT*	13	0.004588701	0.013337769	-	-	-	13	0.005917335	0.013206154	-	-	-	-	-	-	-	-	-	-	-	-
chr5	40835193	40835760	*RPL37*																					
chr7	139320346	139320964	*HIPK2*	5	0.008594558	-0.013715319	-	-	-	5	0.010817813	-0.013537808	-	-	-	5	0.014399606	-0.012704401	-	-	-	-	-	-
chr11	116577961	116578399	-	4	0.00512349	-0.029821306	-	-	-	4	0.006214033	-0.029627922	-	-	-	4	0.003964919	-0.030553573	-	-	-	-	-	-
chr12	16758414	16759391	*LMO3*	14	0.003275516	-0.005086774	-	-	-	14	0.003668124	-0.005043306	-	-	-	14	0.005554107	-0.004836408	-	-	-	-	-	-
chr1	15497910	15498551	*TMEM51*	8	0.000280819	-0.0095017	-	-	-	8	0.000345095	-0.009230095	8	0.010693303	-0.035327005	8	0.000154095	-0.009668484	-	-	-	-	-	-
chr1	114696350	114697113	*SYT6*	14	0.002233925	-0.00310039	-	-	-	14	0.002726942	-0.003118534	-	-	-	14	0.003169217	-0.003015045	-	-	-	-	-	-
chr12	12244945	12245710	*BCL2L14*																					
chr5	132165899	132167026	*SHROOM1*	7	0.00196805	0.007599446	-	-	-	7	0.002396281	0.007621126	-	-	-	7	0.001990066	0.007725335	-	-	-	-	-	-
chr19	35068221	35068788	*SCGB1B2P*	5	0.005312907	-0.010562136	-	-	-	5	0.006381995	-0.010244593	-	-	-	5	0.007609849	-0.010163585	-	-	-	-	-	-
chr1	14219640	14220614	-	7	0.006653562	-0.007006692	-	-	-	7	0.008653806	-0.006902457	-	-	-	7	0.008776932	-0.006677458	-	-	-	-	-	-
chr8	142950423	142950502	-	3	0.006199685	-0.016069227	3	0.016615464	-0.02611997	3	0.007465498	-0.015909764	-	-	-	3	0.00764474	-0.015241812	-	-	-	-	-	-
chr7	81399587	81400745	*HGF*	7	0.001990593	-0.008174373	-	-	-	7	0.002401375	-0.008215577	-	-	-	7	0.002606088	-0.008275426	-	-	-	-	-	-
chr16	3077878	3078339	*BICDL2*																					
chr8	26150452	26150784	*PPP2R2A*	3	0.005698148	0.007071752	-	-	-	3	0.006678507	0.007028211	-	-	-	3	0.006702014	0.007047312	-	-	-	-	-	-
chr7	92076509	92077858	*GATAD1*	11	0.008009014	-0.00260966	-	-	-	11	0.010000068	-0.002646797	-	-	-	11	0.009539122	-0.002719633	-	-	-	-	-	-
chr13	113928287	113929010	-	3	0.008190105	-0.007788617	-	-	-	3	0.009778561	-0.007853608	-	-	-	3	0.009636811	-0.007714464	-	-	-	-	-	-
chr12	125549535	125550542	*AACS*	14	0.008994711	-0.004586151	-	-	-	14	0.010866014	-0.004575009	-	-	-	14	0.007525355	-0.004777813	-	-	-	-	-	-
chr14	103415458	103416389	*CDC42BPB*	6	1.86E-05	-0.01200078	-	-	-	6	2.48E-05	-0.011862466	-	-	-	6	2.76E-05	-0.011837301	-	-	-	-	-	-
chr6	7726645	7726983	*BMP6*	3	0.00491681	0.00876302	-	-	-	3	0.005728443	0.008725944	-	-	-	3	0.005223203	0.008750913	-	-	-	-	-	-
chr7	48129797	48130197	*UPP1*	5	0.000280212	0.018768199	-	-	-	5	0.000227261	0.018913691	-	-	-	5	0.000244127	0.018984876	5	0.025011705	0.023700026	-	-	-
chr12	8234656	8235314	*NECAP1*	8	0.00512212	-0.003465856	-	-	-	-	-	-	-	-	-	-	-	-	-	-	-	-	-	-
chr18	74203484	74204135	*ZNF516*																					
chr16	15818810	15819363	*MYH11*																					
chr5	34007809	34008663	*AMACR*	14	0.007510412	-0.005006052	-	-	-	14	0.009661651	-0.005054178	-	-	-	14	0.006859853	-0.005128001	-	-	-	-	-	-
chr7	128494060	128494696	*FLNC*	7	0.00022114	0.010122935	-	-	-	7	0.00028194	0.009983552	-	-	-	7	0.000349163	0.009996686	-	-	-	-	-	-
chr12	31078790	31079406	*TSPAN11*	6	0.001653025	0.012355804	-	-	-	-	-	-	-	-	-	-	-	-	-	-	-	-	-	-

chr22	22555873	22556540	-	4	0.006085472	0.000116626	-	-	-	4	0.004589128	-0.000234317	-	-	-	4	0.005139554	-0.000486969	-	-	-	-	-	-
chr21	43327823	43327978	*C2CD2*	3	0.002385251	0.013162771	-	-	-	3	0.002958501	0.01308959	-	-	-	3	0.00291511	0.013146845	-	-	-	-	-	-
chr5	66253919	66255772	*MAST4*	9	0.000138113	-0.013670037	-	-	-	9	0.000170218	-0.013642203	-	-	-	9	0.000226554	-0.01356146	-	-	-	-	-	-
chr12	82152229	82153290	*PPFIA2*	16	0.001537432	-0.006135226	-	-	-	-	-	-	-	-	-	-	-	-	-	-	-	-	-	-
chr4	667199	668276	*ATP5ME*																					
chr5	59064235	59064682	*PDE4D*	11	0.00531136	-0.010286565	-	-	-	11	0.006931206	-0.010225922	-	-	-	11	0.008845164	-0.010136173	-	-	-	-	-	-
chr7	134832432	134833335	*CYREN*																					
chr2	33151557	33152037	*LINC00486*	3	0.006049926	-0.005851795	-	-	-	3	0.007017835	-0.005817562	-	-	-	3	0.005773547	-0.005901744	-	-	-	-	-	-
chr22	32438947	32439391	*SLC5A1*	5	8.14E-07	-0.03707628	5	5.49E-06	-0.043332674	5	4.73E-07	-0.037563771	-	-	-	5	8.64E-07	-0.036926807	-	-	-	-	-	-
chr2	139227577	139228296	-	3	0.001145729	-0.012391821	-	-	-	3	0.001417156	-0.012234648	-	-	-	3	0.001069013	-0.012709034	-	-	-	-	-	-
chr22	46465717	46466858	*MIRLET7BHG*	7	0.006283376	-0.013681715	-	-	-	-	-	-	-	-	-	7	0.007285925	-0.013667866	-	-	-	-	-	-
chr1	9488706	9489434	-	6	0.000258984	-0.003216566	-	-	-	-	-	-	-	-	-	6	0.000225798	-0.003245365	-	-	-	-	-	-
chr7	45002287	45002980	*MYO1G*	4	1.37E-06	-0.038542708	-	-	-	4	1.96E-06	-0.038365497	4	0.003184446	-0.104211108	4	1.52E-06	-0.038742788	-	-	-	-	-	-
chr17	72732432	72733290	*RAB37*	15	0.002986439	0.00439106	-	-	-	15	0.003887726	0.00439578	-	-	-	15	0.004052284	0.004391813	-	-	-	-	-	-
chr5	87439075	87441969	-	13	0.000607218	-0.011509158	-	-	-	13	0.000861046	-0.011301223	-	-	-	13	0.001087775	-0.011101127	-	-	-	-	-	-
chr17	79633202	79634204	*CCDC137*																					
chr14	65171010	65172436	*PLEKHG3*	13	0.004446774	0.000643993	-	-	-	13	0.004660764	0.000645601	-	-	-	13	0.005094776	0.000628257	-	-	-	-	-	-
chr1	9970042	9971115	*CTNNBIP1*	16	0.006659918	-0.005711814	-	-	-	16	0.008515941	-0.005668497	16	0.002964866	-0.009519038	16	0.007684875	-0.005791828	-	-	-	-	-	-
chr5	58652602	58652948	*PDE4D*	5	0.00032025	-0.014118523	5	0.006573059	-0.016639209	5	0.000372317	-0.014134403	-	-	-	5	0.00046636	-0.01408883	-	-	-	-	-	-
chr1	151300083	151300904	*PI4KB*	12	0.001305974	0.003594664	-	-	-	12	0.001655224	0.003600742	-	-	-	12	0.001277248	0.003651063	-	-	-	-	-	-
chr1	241587126	241588176	-	9	9.09E-05	-0.008692447	-	-	-	9	7.69E-05	-0.00870751	9	0.018070733	-0.020689784	9	8.12E-05	-0.008695554	-	-	-	-	-	-
chr7	142582859	142583404	*TRPV6*	4	0.002385473	0.013805231	-	-	-	4	0.002835703	0.013850418	-	-	-	4	0.003492336	0.013480288	-	-	-	-	-	-
chr14	93580644	93581139	*ITPK1*	3	0.004779719	0.003210715	-	-	-	3	0.005761987	0.003206165	-	-	-	3	0.004917956	0.00333697	-	-	-	-	-	-
chr19	52799192	52800469	*ZNF480*	11	0.004601154	-0.008490162	-	-	-	11	0.005794214	-0.008425588	-	-	-	11	0.004206375	-0.008671264	-	-	-	-	-	-
chr1	1377525	1378060	*VWA1*	4	0.008065176	0.013481801	-	-	-	4	0.009866383	0.013326506	-	-	-	4	0.009093063	0.013460224	-	-	-	-	-	-
chr12	133066054	133067734	*FBRSL1*	20	0.002444211	-0.011206051	-	-	-	-	-	-	-	-	-	20	0.001977923	-0.01145507	-	-	-	-	-	-
chr7	157293538	157294502	-	8	0.000998853	0.012027103	-	-	-	8	0.001347325	0.011890591	-	-	-	8	0.001511002	0.011706498	-	-	-	-	-	-
chr16	10275767	10276801	*GRIN2A*	15	0.003170425	0.002869829	-	-	-	15	0.003275784	0.002915155	-	-	-	15	0.003580214	0.00291895	-	-	-	-	-	-
chr19	36605252	36606395	*POLR2I*																					
chr2	97533635	97534450	*SEMA4C*	4	0.009793644	0.000734114	-	-	-	4	0.008879962	0.000727089	-	-	-	4	0.01045215	0.000788669	-	-	-	-	-	-
chr1	55464084	55465282	*BSND*	9	0.008835969	0.009200849	-	-	-	-	-	-	-	-	-	-	-	-	-	-	-	-	-	-
chr16	1821856	1822795	*EME2*																					
chr1	2207859	2208225	*SKI*	3	0.006073538	-0.015999838	-	-	-	3	0.006607388	-0.015769689	-	-	-	3	0.008315538	-0.015543206	3	0.00671018	-0.027928695	-	-	-
chr19	55549414	55550348	*GP6*																					
chr11	8008673	8009242	*EIF3F*	8	0.002828725	-0.009571003	-	-	-	8	0.003209605	-0.009651309	-	-	-	8	0.002383173	-0.009715115	-	-	-	-	-	-
chr2	187713554	187714279	*ZSWIM2*	11	0.003560378	-0.007227091	11	0.009464802	-0.009422945	11	0.004579866	-0.007206371	-	-	-	11	0.006094302	-0.007018024	-	-	-	-	-	-
chr7	45299616	45299766	-	3	0.002689141	-0.008487408	-	-	-	3	0.00322606	-0.00849524	-	-	-	3	0.002936951	-0.008486385	-	-	-	-	-	-
chr19	4832695	4832993	*TICAM1*	3	0.008698583	0.008017035	-	-	-	3	0.010220861	0.00796417	-	-	-	3	0.010404618	0.007673329	-	-	-	-	-	-
chr19	2290843	2291872	*LINGO3*																					
chr15	35087506	35088005	*ACTC1*	6	0.005014383	-0.008677238	-	-	-	6	0.00649481	-0.008610578	-	-	-	6	0.006111467	-0.008690034	-	-	-	-	-	-
chr3	152879564	152880101	*RAP2B*	7	0.004810734	-0.000588834	-	-	-	-	-	-	-	-	-	7	0.004540244	-0.000663507	-	-	-	-	-	-
chr15	25319113	25320195	*IPW*																					
chr12	46386648	46387141	*SCAF11*	3	0.009973023	0.020697784	-	-	-	-	-	-	-	-	-	3	0.01030621	0.020833348	-	-	-	-	-	-
chr1	156785998	156786723	*NTRK1*																					
chr3	184032711	184034132	*EIF4G1*	12	0.000412989	0.005632364	-	-	-	-	-	-	-	-	-	-	-	-	-	-	-	-	-	-
chr9	124009589	124009784	*GSN*	3	0.009717649	-0.012546498	-	-	-	3	0.011067847	-0.012551889	-	-	-	3	0.012415217	-0.01226217	-	-	-	-	-	-
chr6	18386873	18387809	*RNF144B*	12	0.003225924	-0.005818798	-	-	-	-	-	-	-	-	-	-	-	-	-	-	-	-	-	-
chr7	30028890	30030138	*SCRN1*	16	0.00624941	0.000582477	-	-	-	16	0.008023744	0.000604095	-	-	-	16	0.006596183	0.000687642	-	-	-	-	-	-
chr20	30308956	30310246	*BCL2L1*	5	0.000774686	0.01268471	-	-	-	-	-	-	-	-	-	-	-	-	-	-	-	-	-	-
chr11	66496615	66497215	-	4	0.006278441	0.017612915	-	-	-	4	0.006667046	0.017594848	-	-	-	4	0.007589576	0.017598849	-	-	-	-	-	-
chr6	26043820	26044405	*H2BC3*																					
chr22	43505892	43507665	*BIK*	30	0.002282624	0.001804404	-	-	-	30	0.003085604	0.001771818	-	-	-	30	0.001940318	0.001837137	-	-	-	-	-	-
chr13	34250594	34251651	*STARD13*	6	0.002387385	-0.014757265	-	-	-	6	0.002545933	-0.014904557	-	-	-	6	0.002071093	-0.014988558	-	-	-	-	-	-
chr11	32455025	32456069	*WT1*																					
chr2	202483740	202484583	*C2CD6*	11	4.14E-07	0.017443227	11	2.12E-08	0.023115481	11	3.89E-07	0.017535965	-	-	-	11	2.67E-07	0.017697265	-	-	-	-	-	-
chr3	129611515	129613115	*TMCC1*																					
chr9	130564509	130565470	*FPGS*	13	0.002676984	-0.009208801	-	-	-	13	0.003722026	-0.00917259	-	-	-	13	0.003137269	-0.00939426	-	-	-	-	-	-
chr4	187125413	187126139	*CYP4V2*	5	0.001096431	-0.035990704	-	-	-	5	0.001407498	-0.035732838	-	-	-	5	0.001454765	-0.035336581	-	-	-	-	-	-
chr8	98880145	98881927	*MATN2*	15	0.004448408	-0.004799669	-	-	-	15	0.004990234	-0.004887668	-	-	-	15	0.004188919	-0.004901441	-	-	-	-	-	-
chr10	114911442	114912149	*TCF7L2*	6	0.006691019	-0.009897289	-	-	-	6	0.00777876	-0.009874721	-	-	-	6	0.005849592	-0.009986081	-	-	-	-	-	-
chr11	2919689	2921176	*SLC22A18*																					
chr3	27772033	27772805	-	6	1.86E-06	-0.00791487	-	-	-	6	2.67E-06	-0.007815475	-	-	-	6	3.39E-06	-0.007689823	6	3.99E-06	-0.009246447	-	-	-
chr2	68546467	68547655	*CNRIP1*	16	0.008713325	-0.004148175	-	-	-	16	0.010914315	-0.004093176	-	-	-	16	0.009849652	-0.004002914	-	-	-	-	-	-
chr10	14050455	14052028	*FRMD4A*	13	0.001494974	-0.021908018	-	-	-	13	0.001830655	-0.021919607	-	-	-	13	0.002857208	-0.021352568	-	-	-	-	-	-
chr4	109540778	109542037	*RPL34*																					
chr19	2782678	2783156	*SGTA*																					
chr19	54515169	54515811	*CACNG6*	4	0.000521357	0.028957583	-	-	-	4	0.000636475	0.028902778	-	-	-	4	0.000393829	0.029374109	-	-	-	-	-	-
chr1	43919181	43920264	*HYI*																					
chr8	1897020	1897487	*ARHGEF10*	4	0.009277949	-0.015985906	-	-	-	4	0.010289687	-0.015681626	-	-	-	4	0.011820463	-0.015717402	-	-	-	-	-	-
chr1	31423023	31423283	*PUM1*																					
chr4	122853693	122854532	*TRPC3*	9	0.002518769	-0.016167804	-	-	-	9	0.003194709	-0.0161606	-	-	-	9	0.002578146	-0.016149971	-	-	-	-	-	-
chr10	22623047	22624374	-	9	0.002605382	0.010731096	-	-	-	9	0.001702803	0.010922943	-	-	-	-	-	-	-	-	-	-	-	-
chr1	151030968	151031323	*CDC42SE1*																					
chr11	62066533	62067691	*SCGB1D4*	6	0.000196965	-0.008624026	-	-	-	6	0.000211549	-0.008676572	-	-	-	6	0.000193923	-0.008564243	-	-	-	-	-	-
chr9	125795488	125796611	*GPR21*																					
chr16	52580266	52581405	*TOX3*	6	0.000710479	-0.01035458	-	-	-	6	0.00098542	-0.010193101	-	-	-	6	0.00089683	-0.010071524	-	-	-	-	-	-
chr10	129947756	129948776	-	5	8.03E-05	0.011429478	-	-	-	-	-	-	-	-	-	5	9.34E-05	0.011648537	-	-	-	-	-	-
chr14	104190678	104191347	*ZFYVE21*	5	0.000225457	-0.015167134	-	-	-	5	0.000246191	-0.015284962	-	-	-	5	0.000179113	-0.01539228	-	-	-	-	-	-
chr1	19181040	19181419	*TAS1R2*	5	0.000364622	-0.023955871	5	0.005343388	-0.031850637	5	0.000432483	-0.024149552	-	-	-	5	0.00025612	-0.024470425	-	-	-	-	-	-
chr7	933881	934221	*GET4*	4	0.004045053	0.005132197	-	-	-	4	0.004427562	0.005237351	-	-	-	4	0.004190208	0.005319953	-	-	-	-	-	-
chr2	229045958	229047110	*SPHKAP*	12	0.002771176	-0.007297383	-	-	-	-	-	-	-	-	-	-	-	-	-	-	-	-	-	-
chr1	58716018	58717107	*DAB1*	11	0.004669643	-0.002957602	-	-	-	11	0.005293389	-0.00284032	-	-	-	-	-	-	-	-	-	-	-	-
chr1	3055958	3057366	*PRDM16*	9	0.001804373	0.005501721	-	-	-	9	0.002129135	0.005432693	-	-	-	9	0.001691709	0.00535585	-	-	-	-	-	-
chr6	31368461	31369119	*MICA*	3	0.008496734	-0.015021247	-	-	-	3	0.007104114	-0.015224234	-	-	-	3	0.008491375	-0.015164447	-	-	-	-	-	-
chr3	101292669	101293068	*PCNP*	11	0.005298936	-0.003136446	-	-	-	11	0.0067646	-0.003130315	-	-	-	11	0.005829355	-0.003175886	-	-	-	-	-	-
chr4	21305490	21306181	*KCNIP4*	8	0.006945821	-0.006917072	-	-	-	8	0.008663302	-0.006814121	-	-	-	8	0.010037809	-0.00678091	-	-	-	-	-	-
chr5	139742283	139743303	*SLC4A9*	3	0.006098369	0.013505983	-	-	-	-	-	-	-	-	-	-	-	-	-	-	-	-	-	-
chr2	87018382	87019612	*CD8A*																					
chr16	31558017	31558891	-	3	0.008061308	0.012145777	-	-	-	3	0.009709794	0.012022722	-	-	-	3	0.007109856	0.012352649	-	-	-	-	-	-
chr7	101558597	101559888	*CUX1*	12	0.001297644	0.006939638	-	-	-	12	0.001597916	0.006881907	-	-	-	-	-	-	-	-	-	-	-	-
chr6	38607245	38608904	*BTBD9*	15	0.003155613	0.001000166	-	-	-	15	0.003906969	0.001000013	-	-	-	15	0.003139123	0.000943897	-	-	-	-	-	-
chr8	22551655	22552961	*EGR3*	5	0.000337975	0.004901147	-	-	-	-	-	-	-	-	-	5	0.000203579	0.005048434	-	-	-	-	-	-
chr18	5578880	5579883	*EPB41L3*	5	0.000152835	0.014621503	-	-	-	5	0.000211947	0.014483333	-	-	-	5	0.000110388	0.014735034	-	-	-	-	-	-
chr1	151689203	151690075	*CELF3*																					
chr2	87015813	87016720	*CD8A*																					
chr16	12995603	12996419	*SHISA9*	6	0.00739644	-0.005858924	-	-	-	6	0.007073233	-0.005917453	-	-	-	-	-	-	-	-	-	-	-	-
chr3	139061956	139063360	*MRPS22*	15	0.000446052	0.001779957	-	-	-	15	0.00052608	0.00180943	15	0.002294438	0.007333135	15	0.000657951	0.001661026	-	-	-	-	-	-
chr6	41604740	41605994	*MDFI*	5	0.002102369	0.010620818	-	-	-	-	-	-	-	-	-	5	0.001963148	0.01075953	-	-	-	-	-	-
chr22	50523686	50524691	*MLC1*																					
chr13	95364333	95364993	*SOX21*	13	0.008020643	-0.00336444	-	-	-	13	0.010179667	-0.003293189	13	0.01724127	-0.016762261	13	0.009465718	-0.003323017	-	-	-	-	-	-
chr3	148804272	148805294	*HLTF*																					
chr2	233283010	233286870	-	15	6.97E-34	0.025381192	-	-	-	15	8.18E-34	0.025256917	-	-	-	15	8.87E-34	0.025639498	-	-	-	-	-	-
chr19	39465821	39467435	*FBXO17*	15	6.98E-05	0.0133187	-	-	-	15	9.38E-05	0.013295596	-	-	-	15	2.24E-05	0.013748104	-	-	-	-	-	-
chr16	79804561	79805381	-	6	0.009459588	0.000878614	-	-	-	6	0.011689234	0.000897495	-	-	-	-	-	-	-	-	-	-	-	-
chr12	81329566	81330609	*LIN7A*																					
chr18	56886915	56887785	*GRP*	12	0.007213202	-0.005066046	-	-	-	12	0.00814513	-0.005061758	-	-	-	12	0.011302828	-0.004917613	-	-	-	-	-	-
chr1	6187633	6188136	*CHD5*	5	0.006016966	-0.028555293	-	-	-	5	0.007703598	-0.028369108	-	-	-	5	0.008902225	-0.027691261	-	-	-	-	-	-
chr19	10120386	10121323	*COL5A3*																					
chr14	102372393	102372657	*PPP2R5C*	3	0.0029126	-0.005067209	-	-	-	3	0.003469827	-0.005029191	-	-	-	3	0.003582667	-0.004942403	3	0.032481976	-0.004227392	-	-	-
chr5	137367832	137369294	*FAM13B*	11	0.003033508	-0.00462706	-	-	-	11	0.003777663	-0.004691202	-	-	-	11	0.003119331	-0.004720393	-	-	-	-	-	-
chr3	9744908	9745460	*CPNE9*	8	0.004167865	-0.00674638	-	-	-	8	0.003413015	-0.00687631	-	-	-	8	0.003965502	-0.006596363	-	-	-	-	-	-

chr7	48702963	48703665	-	4	0.005227335	0.013596207	-	-	-	4	0.006177788	0.013525068	-	-	-	4	0.00577375	0.013623929	-	-	-	-	-	-
chr12	108634147	108634275	*WSCD2*	3	0.000832001	-0.039830341	3	0.043793243	-0.04090917	3	0.001049739	-0.039224242	-	-	-	3	0.00101305	-0.039486698	-	-	-	-	-	-
chr19	14196326	14197848	*C19orf67*	12	0.006820979	0.009019095	-	-	-	-	-	-	-	-	-	-	-	-	-	-	-	-	-	-
chr16	68269041	68272111	*ESRP2*	23	6.18E-06	0.010141548	-	-	-	23	8.69E-06	0.010121786	-	-	-	23	1.89E-07	0.010566703	-	-	-	-	-	-
chr12	57983971	57985234	*PIP4K2C*	11	0.00321948	-0.004675049	-	-	-	-	-	-	-	-	-	-	-	-	-	-	-	-	-	-
chr20	37353700	37354117	*SLC32A1*	3	0.003665955	-0.001468217	-	-	-	3	0.003521444	-0.00151045	3	0.017544984	-0.02782675	3	0.00273176	-0.001606554	-	-	-	-	-	-
chr15	83875649	83877135	*HDGFL3*	10	0.001379053	-0.003474607	-	-	-	10	0.001688847	-0.003426692	-	-	-	10	0.001202001	-0.003423248	-	-	-	-	-	-
chr18	35145353	35147826	*CELF4*	33	0.001705993	-0.002899966	-	-	-	33	0.002288124	-0.002887425	-	-	-	33	0.002431997	-0.002848882	-	-	-	-	-	-
chr9	137660514	137660670	*COL5A1*	3	0.001284006	0.021074213	3	0.003280416	0.02669225	3	0.001389515	0.021159402	-	-	-	3	0.001675688	0.020856848	-	-	-	-	-	-
chr12	56211383	56212269	*DNAJC14*																					
chr9	72590778	72591441	-	3	0.001288771	-0.013991823	-	-	-	3	0.001484136	-0.013917584	-	-	-	3	0.000777556	-0.014089787	-	-	-	-	-	-
chr5	127872767	127874587	*FBN2*	19	1.02E-07	-0.00777935	-	-	-	19	1.75E-07	-0.007633072	-	-	-	19	2.22E-07	-0.007114257	-	-	-	-	-	-
chr19	18721531	18721840	*CRLF1*																					
chr6	28876139	28877723	*TRIM27*	8	0.003547463	-0.002443116	-	-	-	-	-	-	-	-	-	8	0.002374895	-0.002449746	-	-	-	-	-	-
chr4	81117853	81119473	*PRDM8*	13	4.63E-05	0.016556667	-	-	-	13	1.86E-05	0.016979773	-	-	-	13	1.20E-05	0.017217393	-	-	-	-	-	-
chr22	44464449	44465038	*PARVB*	9	0.002831699	-0.009866645	-	-	-	9	0.003496526	-0.009838826	-	-	-	9	0.004572312	-0.009670531	-	-	-	-	-	-
chr14	71066865	71067802	*MED6*	9	0.002863137	-0.009527109	-	-	-	9	0.002982475	-0.00963637	-	-	-	9	0.001898253	-0.009699898	-	-	-	-	-	-
chr10	42970351	42971732	*LINC00839*	11	0.000559462	0.014766421	11	1.13E-06	0.022847944	11	0.000616937	0.014737868	-	-	-	11	0.000337938	0.015090777	-	-	-	-	-	-
chr7	92461971	92462936	*CDK6*	6	0.005282827	-0.000543237	-	-	-	6	0.006464445	-0.000510523	-	-	-	6	0.006630512	-0.000629178	-	-	-	-	-	-
chr1	4832380	4832865	*AJAP1*	5	0.007512025	0.012815455	-	-	-	5	0.009299222	0.012707889	-	-	-	5	0.010359365	0.012593549	-	-	-	-	-	-
chr17	80621842	80622941	*RAB40B*	5	0.003758973	0.007071087	-	-	-	-	-	-	-	-	-	-	-	-	-	-	-	-	-	-
chr8	144371144	144372051	*ZNF696*	6	0.001172935	-0.036936233	-	-	-	6	0.001181054	-0.037352797	-	-	-	6	0.001597813	-0.036586479	-	-	-	-	-	-
chr1	31845904	31846873	*FABP3*	11	9.22E-07	-0.01123283	-	-	-	11	1.66E-06	-0.011146496	-	-	-	11	3.11E-06	-0.011065496	-	-	-	-	-	-
chr16	75894736	75895031	-	5	0.009288021	-0.02771533	-	-	-	5	0.009359069	-0.027942903	-	-	-	5	0.011166893	-0.02745798	-	-	-	-	-	-
chr7	2551247	2551996	*LFNG*	6	0.002018032	-0.007952996	-	-	-	6	0.002440899	-0.007990509	-	-	-	6	0.001268386	-0.008336146	-	-	-	-	-	-
chr15	89455860	89456239	*MFGE8*	3	0.006975725	-0.014029093	-	-	-	3	0.008654264	-0.013985944	-	-	-	3	0.006939353	-0.013923567	3	0.0084297	-0.024200076	-	-	-
chr16	88716846	88717989	*CYBA*	16	0.009890407	-0.004953177	-	-	-	-	-	-	-	-	-	16	0.009700941	-0.005259213	-	-	-	-	-	-
chr6	28504908	28505429	-	6	0.002891373	-0.004059525	-	-	-	6	0.003564634	-0.004093061	-	-	-	6	0.003990562	-0.003337881	-	-	-	-	-	-
chr13	77900505	77901478	*MYCBP2*	12	0.009632255	-0.004728204	-	-	-	12	0.011406527	-0.004755308	12	0.012449482	-0.019750269	12	0.007157205	-0.004871803	-	-	-	-	-	-
chr5	125758447	125759291	*GRAMD2B*	10	0.001813753	-0.004059729	-	-	-	10	0.002221786	-0.00409584	-	-	-	10	0.002411586	-0.004024465	-	-	-	-	-	-
chr10	79396127	79397075	*KCNMA1*	5	0.005978517	0.010632361	-	-	-	5	0.007080588	0.010504743	-	-	-	5	0.004607896	0.010784205	-	-	-	-	-	-
chr12	88973398	88974665	*KITLG*	15	0.000935756	-0.005234392	-	-	-	15	0.001241692	-0.00520616	-	-	-	15	0.001467855	-0.005046206	-	-	-	-	-	-
chr4	46390730	46392485	*GABRA2*	17	0.004975417	-0.00266794	-	-	-	-	-	-	-	-	-	-	-	-	-	-	-	-	-	-
chr4	38320637	38321262	-	6	0.001796136	-0.010766883	-	-	-	6	0.002222143	-0.010794519	-	-	-	6	0.001660037	-0.010962419	-	-	-	-	-	-
chr18	3262499	3263082	*MYL12B*	8	0.008416671	-0.005245314	-	-	-	8	0.010472988	-0.005257186	-	-	-	8	0.0079357	-0.005521493	-	-	-	-	-	-
chr1	202310824	202311492	*UBE2T*	9	0.000124539	-0.009005555	9	0.000531476	-0.011553339	9	0.000149776	-0.009049559	-	-	-	9	0.000163534	-0.009009494	-	-	-	-	-	-
chr16	85027930	85028515	*ZDHHC7*	3	0.00301993	-0.004056518	-	-	-	3	0.003277436	-0.004007994	-	-	-	3	0.002945801	-0.003943767	-	-	-	-	-	-
chr8	12990213	12991259	*DLC1*	12	0.009576175	-0.007826822	-	-	-	-	-	-	-	-	-	-	-	-	-	-	-	-	-	-
chr4	5990590	5991345	-	3	0.001733878	-0.002396878	-	-	-	3	0.002115332	-0.002435584	-	-	-	3	0.001549387	-0.002505085	-	-	-	-	-	-
chr4	106816089	106817015	*NPNT*	10	0.006326615	-0.004716081	-	-	-	10	0.006855135	-0.004658308	-	-	-	10	0.00674214	-0.004399963	-	-	-	-	-	-
chr22	30783074	30783995	*RNF215*	10	0.000942649	0.007572036	10	1.99E-06	0.014202837	10	0.001310385	0.007486451	-	-	-	10	0.001415601	0.007245233	-	-	-	-	-	-
chr20	62421032	62421950	*ZBTB46*	5	0.005557189	0.002443608	-	-	-	-	-	-	-	-	-	5	0.006336329	0.002563237	-	-	-	-	-	-
chr9	101469203	101470884	*GABBR2*	7	0.002282624	0.001517375	-	-	-	7	0.002745547	0.00162417	-	-	-	7	0.00082265	0.001480603	-	-	-	-	-	-
chr6	33283770	33285470	*TAPBP*																					
chr5	101834136	101835072	*SLCO6A1*	10	0.005673381	0.011058772	-	-	-	10	0.006802131	0.010985956	-	-	-	10	0.005805457	0.011194696	10	0.017138315	0.017268132	-	-	-
chr8	125740425	125741566	*MTSS1*	13	5.22E-05	0.006718675	-	-	-	13	5.74E-05	0.006758184	-	-	-	13	6.09E-05	0.006631666	-	-	-	-	-	-
chr4	89642483	89642760	*FAM13A-AS1*	3	0.006619128	0.008679472	-	-	-	3	0.006313561	0.008671141	-	-	-	3	0.006832182	0.008693009	-	-	-	-	-	-
chr1	153651147	153652247	*NPR1*	8	0.005011851	-0.004643349	-	-	-	8	0.006139372	-0.004593114	-	-	-	8	0.005842508	-0.004777422	-	-	-	-	-	-
chr14	104195181	104196038	*ZFYVE21*	6	0.006867779	-0.004913069	-	-	-	6	0.00844993	-0.004729308	-	-	-	6	0.00581408	-0.00520111	-	-	-	-	-	-
chr3	85007959	85008991	*CADM2*	11	0.005237457	-0.004900412	-	-	-	11	0.006294421	-0.004812245	-	-	-	-	-	-	-	-	-	-	-	-
chr17	79372242	79374741	*BAHCC1*																					
chr13	74864357	74864782	-	3	0.00438758	-0.027302837	-	-	-	3	0.005023233	-0.0274112	-	-	-	3	0.005374838	-0.027138297	-	-	-	-	-	-
chr1	222014008	222014996	-	4	0.002977564	-0.016251601	-	-	-	4	0.00376452	-0.016104698	-	-	-	4	0.004303749	-0.01585932	-	-	-	-	-	-
chr4	3726450	3726836	-	3	0.005243282	-0.01381752	-	-	-	3	0.006305227	-0.013768105	-	-	-	3	0.00513586	-0.014030782	-	-	-	-	-	-
chr7	43232981	43233971	*HECW1*	3	0.002531576	-0.012581911	-	-	-	3	0.001502199	-0.012725576	-	-	-	-	-	-	-	-	-	-	-	-
chr6	43043516	43044371	*PTK7*	7	0.005464051	-0.00212977	-	-	-	7	0.00692688	-0.002066116	-	-	-	7	0.007718866	-0.002185222	-	-	-	-	-	-
chr15	96873850	96875830	*MIR1469*																					
chr17	78831167	78831863	*RPTOR*	5	0.003306592	0.010707271	-	-	-	5	0.003107438	0.010638642	-	-	-	5	0.002061326	0.010748872	-	-	-	-	-	-
chr12	42326382	42326707	-	3	0.00555627	-0.012128244	-	-	-	3	0.007190052	-0.012000239	-	-	-	3	0.007609849	-0.012017722	-	-	-	-	-	-
chr1	68150193	68151160	*GADD45A*	9	0.007136745	-0.002358654	-	-	-	9	0.009161202	-0.002331045	-	-	-	9	0.006556835	-0.002389314	-	-	-	-	-	-
chr8	41167278	41168186	*SFRP1*	9	0.007187199	-0.003386987	-	-	-	-	-	-	-	-	-	-	-	-	-	-	-	-	-	-
chr4	1858231	1858883	*LETM1*	3	8.51E-06	0.009277285	-	-	-	3	1.14E-05	0.009241713	-	-	-	3	1.25E-05	0.009077553	-	-	-	-	-	-
chr7	99691385	99691942	*MCM7*																					
chr12	116985025	116986371	-	6	0.000155474	-0.004911984	-	-	-	6	0.00021375	-0.004960916	-	-	-	6	0.000245793	-0.004699102	-	-	-	-	-	-
chr1	209978796	209980186	*IRF6*	15	3.50E-09	-0.017225962	-	-	-	15	4.91E-09	-0.017301232	-	-	-	15	1.61E-09	-0.017443599	-	-	-	-	-	-
chr15	51633381	51634583	*CYP19A1*																					
chr2	74153795	74154363	*DGUOK*	10	0.008184584	-0.00372982	-	-	-	10	0.009577193	-0.003704056	-	-	-	10	0.008116418	-0.00390655	-	-	-	-	-	-
chr1	166135963	166136996	*FAM78B*	13	0.000484411	-0.006653587	-	-	-	13	0.000649202	-0.006604276	-	-	-	13	0.000546664	-0.006641968	-	-	-	-	-	-
chr11	19261879	19263311	*E2F8*	18	0.004280941	-0.002041895	-	-	-	-	-	-	-	-	-	18	0.003613269	-0.002048295	-	-	-	-	-	-
chr6	79942971	79944186	*HMGN3*																					
chr2	233739771	233740203	*SNORC*	3	0.007837608	0.006679865	-	-	-	3	0.008897033	0.006537711	-	-	-	3	0.009347663	0.006658259	-	-	-	-	-	-
chr1	6639249	6640149	*TAS1R1*																					
chr2	239432223	239432329	*LINC01107*	3	0.006957554	-0.025955557	-	-	-	3	0.008531428	-0.025785685	-	-	-	3	0.008012921	-0.025904169	-	-	-	-	-	-
chr6	29795501	29796614	*HLA-G*																					
chr8	11659497	11660968	*FDFT1*	19	0.001941171	0.003771144	-	-	-	19	0.001961657	0.003753227	-	-	-	19	0.002552222	0.003578107	-	-	-	-	-	-
chr4	8207058	8207325	*SH3TC1*	4	0.004602545	0.012435289	-	-	-	4	0.005248444	0.012236822	-	-	-	4	0.006321268	0.01223369	-	-	-	-	-	-
chr5	132112449	132112975	*SEPTIN8*	7	0.008318586	0.000632762	-	-	-	7	0.009799048	0.000589038	-	-	-	7	0.007843659	0.000561717	-	-	-	-	-	-
chr11	5601832	5602123	*HBG2*																					
chr6	170557102	170558102	-	6	1.14E-06	-0.043424206	-	-	-	6	1.37E-06	-0.043579817	-	-	-	6	1.49E-06	-0.043468966	-	-	-	-	-	-
chr9	127562983	127563860	*OLFML2A*	4	8.57E-05	-0.018572537	-	-	-	-	-	-	-	-	-	4	5.25E-05	-0.019018546	-	-	-	-	-	-
chr16	68321421	68321730	*SLC7A6*	6	0.005557189	-0.012784393	-	-	-	6	0.006497375	-0.012782407	-	-	-	6	0.009738982	-0.012187849	6	0.003366318	-0.022046925	-	-	-
chr19	35629273	35630651	*FXYD1*																					
chr19	37340642	37341870	*ZNF345*																					
chr7	95064015	95065139	*PON2*	16	0.004619124	0.000376096	-	-	-	16	0.005289576	0.000342251	-	-	-	16	0.003079155	0.00035748	-	-	-	-	-	-
chr15	90357202	90357992	*ANPEP*	3	0.000114911	0.011920858	-	-	-	3	0.000146182	0.011878231	-	-	-	3	8.69E-05	0.012031763	-	-	-	-	-	-
chr6	33129024	33134325	*COL11A2*	58	0.000154785	0.005219513	-	-	-	58	0.000172368	0.005135106	-	-	-	-	-	-	-	-	-	-	-	-
chr10	23632825	23634319	*C10orf67*	8	0.002608165	-0.007114364	-	-	-	8	0.002878331	-0.007297198	-	-	-	8	0.002819942	-0.007141801	-	-	-	-	-	-
chr1	150254280	150255880	*CIART*	14	3.90E-05	0.004242032	-	-	-	14	4.39E-05	0.004327662	-	-	-	14	2.81E-05	0.004704928	-	-	-	-	-	-
chr1	23279278	23280477	-	7	0.003029638	-0.016568917	-	-	-	7	0.00323913	-0.016603617	-	-	-	7	0.002900277	-0.016644393	-	-	-	-	-	-
chr15	91414499	91416118	*FURIN*	14	0.000589333	0.003058598	-	-	-	-	-	-	-	-	-	14	0.000723128	0.002997111	-	-	-	-	-	-
chr18	2906750	2907472	*EMILIN2*	5	0.001279917	0.006291905	-	-	-	5	0.001542765	0.006185478	-	-	-	5	0.001509876	0.005815589	-	-	-	-	-	-
chr3	118891705	118892461	*UPK1B*	7	0.005929926	-0.016100563	-	-	-	7	0.005939005	-0.01624069	7	0.00311921	-0.072148099	7	0.006451452	-0.016135158	-	-	-	-	-	-
chr2	45159663	45161333	-	13	0.007477544	-0.007073125	-	-	-	-	-	-	-	-	-	-	-	-	-	-	-	-	-	-
chr15	85114169	85114479	*LINC00933*																					
chr6	24489671	24491098	*ALDH5A1*																					
chr20	42142005	42143502	*L3MBTL1*	29	2.51E-05	-0.005135601	-	-	-	29	3.44E-05	-0.005139685	-	-	-	29	1.07E-05	-0.005348975	-	-	-	-	-	-
chr4	3318625	3319541	*RGS12*	6	0.002722313	0.015174064	-	-	-	6	0.003088374	0.015150929	-	-	-	6	0.003085149	0.015344832	-	-	-	-	-	-
chr6	169283989	169285077	-	8	0.001768339	-0.00712826	-	-	-	8	0.001579823	-0.0072671	-	-	-	8	0.002531658	-0.006913725	-	-	-	-	-	-
chr11	1967857	1968643	*MRPL23*	9	0.009932584	-0.004562304	-	-	-	-	-	-	-	-	-	9	0.009369084	-0.004807067	-	-	-	-	-	-
chr15	100272053	100272751	*LYSMD4*	3	0.005774582	0.022130216	-	-	-	3	0.006151165	0.022006259	-	-	-	3	0.003798607	0.022821111	-	-	-	-	-	-
chr4	89744279	89744963	*FAM13A*	6	0.006176859	-0.004285224	-	-	-	6	0.007080534	-0.004270532	-	-	-	6	0.008375339	-0.004171707	-	-	-	-	-	-
chr11	64013441	64015574	*PLCB3*																					
chr2	467799	468330	-	4	0.006298841	0.005187518	-	-	-	-	-	-	-	-	-	4	0.00654755	0.005381984	-	-	-	-	-	-
chr10	73516760	73517463	*CDH23*																					
chr6	138893111	138893718	*NHSL1*	6	2.31E-05	-0.019135165	-	-	-	6	2.97E-05	-0.019146077	-	-	-	6	3.68E-05	-0.018686145	-	-	-	-	-	-
chr5	170813996	170814878	*MIR3912*																					
chr15	39873060	39874413	*THBS1*	10	0.002005729	0.00216734	-	-	-	10	0.002364594	0.002157017	-	-	-	-	-	-	-	-	-	-	-	-
chr13	20160420	20161246	-	5	0.000189313	-0.017244023	-	-	-	5	0.000172793	-0.01729071	-	-	-	5	0.000285894	-0.016709836	-	-	-	-	-	-
chr6	30227729	30228431	*HCG17*																					
chr19	38746538	38747292	*PPP1R14A*	11	0.001866052	0.00354262	-	-	-	11	0.002348995	0.003489344	-	-	-	-	-	-	-	-	-	-	-	-
chr3	39448643	39449544	*RPSA*																					

chr2	239628381	239629498	-	8	0.00024027	-0.004495198	-	-	-	8	0.000326008	-0.004295297	-	-	-	8	0.000362947	-0.004348848	-	-	-	-	-	-
chr8	144222015	144222455	-	5	0.002861448	-0.029452343	-	-	-	5	0.003274489	-0.029455576	-	-	-	5	0.00184187	-0.030312687	-	-	-	5	0.013498256	-0.122093527
chr7	612458	612876	*PRKAR1B*	4	0.000637681	0.009844072	-	-	-	4	0.000776612	0.009760439	-	-	-	4	0.000775494	0.009789491	-	-	-	-	-	-
chr15	54270514	54271167	-	6	0.00019655	-0.00873434	-	-	-	6	0.000248092	-0.008714455	-	-	-	6	0.000297223	-0.008714881	-	-	-	-	-	-
chr5	115962086	115962584	-	3	0.001955796	-0.020849584	-	-	-	3	0.002419109	-0.020420527	-	-	-	3	0.001533056	-0.021399644	3	0.01420393	-0.033741238	-	-	-
chr12	72148251	72148853	*RAB21*	10	0.001813365	0.005172765	-	-	-	10	0.002067393	0.004962341	-	-	-	10	0.002269981	0.005118147	-	-	-	-	-	-
chr1	203097234	203098085	*ADORA1*	9	0.001656603	0.00237419	-	-	-	-	-	-	-	-	-	-	-	-	-	-	-	-	-	-
chr1	1099630	1100557	*MIR200A*																					
chr3	52351355	52351963	*DNAH1*	6	0.00033646	-0.026590054	-	-	-	6	0.000423145	-0.026468587	-	-	-	6	0.000508998	-0.026224887	6	0.025278038	-0.0345592	-	-	-
chr16	20338303	20339420	*GP2*	7	0.004587716	0.009836557	-	-	-	7	0.005740346	0.009747745	-	-	-	7	0.004798494	0.010066209	-	-	-	-	-	-
chr2	74779799	74780272	*DOK1*																					
chr1	208041879	208042623	-	5	0.007665102	-0.001462388	-	-	-	-	-	-	-	-	-	5	0.00756064	-0.001489931	-	-	-	-	-	-
chr14	31027639	31028244	*G2E3*	9	0.00257683	-0.00386589	-	-	-	-	-	-	-	-	-	9	0.002548275	-0.003982944	-	-	-	-	-	-
chr14	104551481	104552397	*ASPG*	12	0.003338685	-0.011532821	-	-	-	12	0.004374464	-0.011419729	-	-	-	12	0.003067738	-0.011532606	-	-	-	12	0.003378588	-0.049895178
chr5	78907212	78908220	*TENT2*	9	0.000309876	0.002836381	-	-	-	-	-	-	-	-	-	-	-	-	-	-	-	-	-	-
chr1	210501532	210502792	*HHAT*	13	0.009839793	-0.003070556	-	-	-	-	-	-	-	-	-	-	-	-	-	-	-	-	-	-
chr11	34580349	34580491	-	3	0.008497643	0.024385929	-	-	-	3	0.010266193	0.02422142	-	-	-	3	0.010143261	0.02426309	-	-	-	-	-	-
chr3	64430390	64431038	-	7	0.007632054	-0.003413822	-	-	-	7	0.009357137	-0.003360269	-	-	-	7	0.010139856	-0.003048879	-	-	-	-	-	-
chr15	41220951	41222276	*DLL4*	12	0.004294622	-0.00292331	-	-	-	-	-	-	-	-	-	12	0.003955078	-0.002981933	-	-	-	-	-	-
chr1	160681404	160682655	*CD48*	9	0.007120874	-0.005765485	-	-	-	9	0.0080644	-0.005768408	-	-	-	9	0.005319728	-0.005873186	-	-	-	-	-	-
chr5	139737425	139738364	*SLC4A9*	4	0.002371823	-0.001872031	-	-	-	4	0.002878331	-0.001928169	-	-	-	4	0.00263129	-0.001979979	-	-	-	-	-	-
chr2	201674914	201676437	*BZW1*	11	1.64E-05	0.004914506	-	-	-	11	2.26E-05	0.004869225	-	-	-	11	1.71E-05	0.004838323	-	-	-	-	-	-
chr19	45579378	45580221	*GEMIN7*																					
chr7	47621223	47622718	*TNS3*	13	0.000190007	-0.003829697	-	-	-	13	0.000280016	-0.003758435	-	-	-	13	0.000273408	-0.003600928	-	-	-	-	-	-
chr6	29910101	29911095	*HLA-A*																					
chr14	34931026	34931822	*SPTSSA*	7	0.008816105	-0.013661976	-	-	-	7	0.010913953	-0.013809849	-	-	-	7	0.007956481	-0.013918466	-	-	-	-	-	-
chr8	145747659	145748901	*LRRC14*																					
chr20	48184639	48186174	*PTGIS*	9	0.002343019	0.007898463	-	-	-	9	0.002406123	0.007960277	-	-	-	9	0.003469355	0.007538279	-	-	-	-	-	-
chr17	39890994	39892088	*HAP1*																					
chr3	132755839	132756669	*TMEM108*	6	0.005165643	-0.006344555	-	-	-	6	0.006604711	-0.006339149	-	-	-	6	0.005263263	-0.00649879	-	-	-	-	-	-
chr11	67210033	67210812	*CORO1B*																					
chr9	93563033	93564033	*SYK*	12	0.009185936	-0.004973692	-	-	-	12	0.012251098	-0.004869905	-	-	-	12	0.011535916	-0.005051809	-	-	-	-	-	-
chr9	137772282	137772886	*FCN2*	6	0.003303116	-0.011380032	-	-	-	6	0.004486578	-0.011243315	-	-	-	6	0.003286268	-0.01161991	-	-	-	-	-	-
chr19	6502301	6502938	*TUBB4A*	11	0.003918394	0.004465638	-	-	-	11	0.005299549	0.004322842	-	-	-	11	0.004012489	0.004515386	-	-	-	-	-	-
chr19	37997105	37997867	*ZNF793*	14	0.009342713	-0.004325528	-	-	-	-	-	-	-	-	-	-	-	-	-	-	-	-	-	-
chr8	110693195	110693461	*SYBU*	4	0.009564908	-0.007747142	-	-	-	4	0.010114038	-0.007897132	-	-	-	4	0.009090818	-0.008011071	-	-	-	-	-	-
chr21	45285836	45287137	*AGPAT3*	5	0.0001381	0.005015512	-	-	-	5	0.00018153	0.004944485	-	-	-	5	0.000228952	0.004501188	-	-	-	-	-	-
chr6	143771318	143772728	*ADAT2*																					
chr5	174150286	174151498	*MSX2*	10	0.003187349	-0.001616145	-	-	-	10	0.004057219	-0.00152893	-	-	-	10	0.004571265	-0.001375207	-	-	-	-	-	-
chr21	37092789	37093340	*MIR802*																					
chr4	40057241	40059856	*LOC344967*																					
chr7	100660212	100660957	*MUC12*																					
chr8	146276606	146277845	*C8orf33*	5	0.002504977	-0.001994904	-	-	-	-	-	-	-	-	-	-	-	-	-	-	-	-	-	-
chr7	151330008	151330288	*PRKAG2*	5	0.007855446	-0.005974838	-	-	-	5	0.009646377	-0.00597489	-	-	-	5	0.009011881	-0.006034278	-	-	-	-	-	-
chr3	141144022	141145231	*ZBTB38*	5	0.000224158	0.011413504	-	-	-	5	0.000273909	0.01140675	-	-	-	5	0.00032612	0.010959561	-	-	-	-	-	-
chr1	35913782	35914081	*KIAA0319L*	3	0.003164955	-0.017767257	-	-	-	3	0.003670801	-0.017754591	-	-	-	3	0.00366096	-0.01763321	-	-	-	-	-	-
chr3	52231311	52231840	*ALAS1*	5	0.002346519	0.011878955	-	-	-	5	0.002773654	0.011768451	-	-	-	5	0.0028739	0.01148915	-	-	-	-	-	-
chr6	44225648	44226520	*MIR4647*																					
chr4	69312514	69313197	*TMPRSS11E*	4	0.001281881	-0.019901022	-	-	-	4	0.00156499	-0.019682473	-	-	-	4	0.001087293	-0.020075298	-	-	-	-	-	-
chr17	4853232	4853717	*ENO3*																					
chr2	228497892	228498185	*C2orf83*	3	0.009977966	0.008373383	-	-	-	3	0.0112791	0.008223933	-	-	-	3	0.009572129	0.008514505	-	-	-	-	-	-
chr1	93544497	93545831	*MTF2*	10	0.001451341	-0.004787773	-	-	-	10	0.001536871	-0.004728298	-	-	-	10	0.001720429	-0.004784804	-	-	-	-	-	-
chr5	369969	370479	*AHRR*	4	4.63E-08	0.018254492	4	1.31E-05	0.015506826	4	4.03E-08	0.018189538	4	0.005745548	0.057257065	4	6.54E-08	0.018213751	-	-	-	-	-	-
chr1	203241948	203242950	*CHIT1*	9	0.007695809	0.004258889	-	-	-	9	0.009502922	0.004221976	-	-	-	9	0.008636802	0.004309509	-	-	-	-	-	-
chr18	46064542	46066011	*CTIF*	12	8.77E-05	0.002393375	-	-	-	12	0.000103251	0.002401345	-	-	-	12	2.88E-05	0.002537755	-	-	-	-	-	-
chr5	72596701	72597316	-	6	0.006601991	-0.00788054	-	-	-	6	0.008187148	-0.007827548	-	-	-	6	0.007426806	-0.007735277	-	-	-	-	-	-
chr9	14315886	14316462	*NFIB*	4	0.000732239	-0.000247437	-	-	-	4	0.000913675	-2.26E-05	-	-	-	4	0.001012772	7.58E-05	-	-	-	-	-	-
chr3	57456170	57456332	*DNAH12*	3	0.000276796	-0.009331308	-	-	-	3	0.000323528	-0.009294687	-	-	-	3	0.000271108	-0.009272709	-	-	-	-	-	-
chr5	6581774	6583103	*LINC01018*	6	0.001381004	0.009015501	-	-	-	6	0.001711236	0.008886149	-	-	-	6	0.001595063	0.008841998	-	-	-	-	-	-
chr6	45389313	45391973	*RUNX2*	20	0.005623479	-0.004254759	-	-	-	-	-	-	-	-	-	20	0.007572446	-0.004252337	-	-	-	-	-	-
chr1	206306094	206307060	-	5	3.35E-05	-0.011733368	-	-	-	5	4.78E-05	-0.011539601	-	-	-	5	5.08E-05	-0.011633644	-	-	-	-	-	-
chr3	8774922	8775601	*CAV3*																					
chr8	144098507	144099991	*LY6E*																					
chr16	1349178	1349861	-	5	0.007612885	0.003987196	-	-	-	5	0.008897114	0.003831008	-	-	-	5	0.007562561	0.003922932	-	-	-	-	-	-
chr3	194014481	194015171	-	7	3.90E-12	0.033498744	7	2.01E-09	0.039948287	7	6.53E-12	0.033424646	7	0.009200644	0.068477077	7	2.72E-13	0.034422102	-	-	-	-	-	-
chr15	102345708	102345981	*OR4F6*	3	0.004995071	-0.016883385	-	-	-	3	0.006206055	-0.016984065	-	-	-	3	0.005332416	-0.017005342	-	-	-	-	-	-
chr10	32667705	32667923	*EPC1*	7	0.003275516	-0.005989165	-	-	-	7	0.004065389	-0.005977544	-	-	-	7	0.003729434	-0.00601522	-	-	-	-	-	-
chr12	132905611	132906443	*GALNT9*	9	0.001997496	-0.012362503	-	-	-	9	0.002659333	-0.012208385	-	-	-	9	0.002956282	-0.012120301	-	-	-	-	-	-
chr2	30144152	30145152	*ALK*	11	0.000404786	-0.007914213	-	-	-	11	0.000476769	-0.007901626	-	-	-	-	-	-	-	-	-	-	-	-
chr21	45883590	45883864	-	3	0.001933051	0.022825889	3	0.008283665	0.029211841	3	0.002469165	0.022549574	-	-	-	3	0.002165177	0.022877234	-	-	-	-	-	-
chr1	112161618	112162084	*RAP1A*	4	0.002088251	0.03602836	-	-	-	4	0.002563519	0.035678186	-	-	-	4	0.002679588	0.03584149	-	-	-	-	-	-
chr4	42398518	42399484	*SHISA3*	7	0.005268892	-0.004744814	-	-	-	7	0.006576562	-0.004727021	-	-	-	7	0.007925312	-0.004535775	-	-	-	-	-	-
chr16	57672401	57673993	*ADGRG1*	9	1.26E-05	-0.010535734	-	-	-	9	1.80E-05	-0.010517592	-	-	-	9	2.39E-05	-0.01020589	-	-	-	-	-	-
chr19	56988398	56990070	*ZNF667*																					
chr13	113303307	113303719	*ATP11AUN*	4	0.004948501	0.014208581	-	-	-	4	0.006017586	0.014072719	-	-	-	4	0.005902802	0.014385378	-	-	-	-	-	-
chr16	742426	743338	*FBXL16*																					
chr17	72426469	72427563	*GPRC5C*	13	0.004599585	0.000171781	-	-	-	13	0.005948883	0.000187832	-	-	-	13	0.005708065	0.000198216	-	-	-	-	-	-
chr3	172859699	172860467	*SPATA16*	4	0.007412295	0.015455402	-	-	-	4	0.00799954	0.015421375	-	-	-	4	0.008709366	0.015645916	-	-	-	-	-	-
chr1	161100749	161101135	*DEDD*	4	0.00591479	0.008719602	-	-	-	4	0.00688008	0.008661714	-	-	-	4	0.007467826	0.008487815	-	-	-	-	-	-
chr7	90224886	90226284	*CDK14*	15	0.000114911	-0.006255701	-	-	-	15	0.000154421	-0.00616849	-	-	-	15	0.000153633	-0.006137424	-	-	-	-	-	-
chr2	25427064	25427652	-	6	0.001087026	0.023803618	-	-	-	6	0.001486075	0.023546383	-	-	-	6	0.001199991	0.023897924	-	-	-	-	-	-
chr6	27776350	27776566	*H2BC13*																					
chr21	45704786	45706101	*AIRE*	15	0.001833905	0.014942939	-	-	-	15	0.002276082	0.014932892	-	-	-	15	0.001710691	0.015090896	-	-	-	-	-	-
chr12	1714851	1715055	*WNT5B*	3	0.00039425	-0.010087272	3	0.0014986	-0.012406099	3	0.000511446	-0.009989845	-	-	-	3	0.000613458	-0.00976427	-	-	-	-	-	-
chr7	94537347	94538408	*PPP1R9A*	11	0.001791448	-0.005388318	-	-	-	11	0.001907646	-0.005384179	-	-	-	11	0.00226822	-0.005447875	-	-	-	-	-	-
chr15	26108391	26109614	*ATP10A*	29	2.68E-05	0.002474212	-	-	-	29	4.10E-05	0.002425334	29	0.001567	-0.001741066	29	4.85E-05	0.002274509	-	-	-	-	-	-
chr11	122451407	122452135	-	3	1.79E-05	-0.018405058	-	-	-	3	2.13E-05	-0.018523386	-	-	-	3	2.04E-05	-0.018439252	-	-	-	-	-	-
chr9	140195998	140197048	*NRARP*	8	0.006868233	-0.001500532	-	-	-	8	0.008829894	-0.001489398	-	-	-	8	0.008295602	-0.001506522	-	-	-	-	-	-
chr19	5752188	5753221	*CATSPERD*	3	0.006394358	0.009862964	-	-	-	3	0.00725503	0.009741342	-	-	-	3	0.007823422	0.009618239	-	-	-	-	-	-
chr2	26828219	26829772	*CIB4*	6	0.00258474	4.25E-05	-	-	-	6	0.00322606	-1.27E-05	-	-	-	6	0.003579572	-0.000123467	-	-	-	-	-	-
chr10	134650068	134650743	*CFAP46*	3	6.29E-07	-0.023709664	-	-	-	3	8.05E-07	-0.023623941	-	-	-	3	3.57E-07	-0.023893484	-	-	-	-	-	-
chr6	4950308	4950673	*CDYL*	3	0.00199577	0.001267095	-	-	-	3	0.001568612	0.001164356	-	-	-	3	0.002318417	0.001386209	-	-	-	-	-	-
chr2	175204249	175205716	-	5	0.00786044	-0.000616299	-	-	-	-	-	-	-	-	-	-	-	-	-	-	-	-	-	-
chr11	58980377	58980665	*MPEG1*	3	0.008086991	-0.013787844	-	-	-	3	0.009830865	-0.013801185	-	-	-	3	0.008996088	-0.013831048	-	-	-	-	-	-
chr16	57843964	57844572	*KIFC3*																					
chr7	99954042	99955101	*PILRB*	6	0.000260024	-0.005375218	-	-	-	-	-	-	-	-	-	6	0.000362947	-0.005125398	-	-	-	-	-	-
chr7	56118783	56119637	*CCT6A*																					
chr12	54408427	54409657	*HOXC4*																					
chr12	106974623	106977074	*LOC100287944*																					
chr1	204485075	204486394	*MDM4*	12	0.000602934	0.00478865	-	-	-	12	0.000778661	0.004790869	-	-	-	12	0.000787529	0.004731593	-	-	-	-	-	-
chr2	3129847	3130349	-	4	0.002979907	0.007541589	-	-	-	4	0.00354019	0.007541729	-	-	-	4	0.004059199	0.007335388	-	-	-	-	-	-
chr17	35165324	35166190	-	5	0.005710217	-0.010033202	-	-	-	5	0.006666152	-0.010024756	-	-	-	5	0.006073959	-0.010141801	-	-	-	-	-	-
chr6	147091024	147092242	*ADGB*	6	0.001423924	-0.008996488	-	-	-	6	0.001772861	-0.009014305	-	-	-	6	0.002256101	-0.008712586	-	-	-	-	-	-
chr9	130699885	130701076	*DPM2*	10	0.004800947	0.011593421	10	0.000562483	0.014094658	10	0.005937017	0.011553849	-	-	-	10	0.003797074	0.011845243	-	-	-	-	-	-
chr10	89418800	89420794	*PAPSS2*	21	0.001536325	0.001323591	-	-	-	21	0.001942953	0.001331437	-	-	-	21	0.002081937	0.001294657	-	-	-	-	-	-
chr2	106755003	106755721	*UXS1*	6	0.004696998	0.015031884	-	-	-	6	0.004820319	0.015103298	-	-	-	6	0.003966442	0.015201903	-	-	-	-	-	-
chr2	38604442	38604853	*ATL2*	4	0.005559825	-0.002493097	-	-	-	4	0.005842857	-0.002619493	-	-	-	4	0.005960583	-0.002592178	-	-	-	-	-	-
chr16	85074359	85075249	*KIAA0513*	6	0.006839347	0.005336808	-	-	-	-	-	-	-	-	-	6	0.006520747	0.004735233	-	-	-	-	-	-
chr4	66535145	66536772	*EPHA5*																					
chr2	32389741	32390937	*SLC30A6*	10	0.003207599	-0.00256139	-	-	-	-	-	-	-	-	-	-	-	-	-	-	-	-	-	-
chr12	11708469	11709857	*LINC01252*	7	0.000874141	0.027815053	-	-	-	7	0.001208803	0.027514619	-	-	-	7	0.000774357	0.028407382	-	-	-	-	-	-

chr7	45613590	45614848	*ADCY1*	9	0.00232501	0.007046372	-	-	-	9	0.002967671	0.007015803	-	-	-	9	0.001465228	0.007334892	-	-	-	-	-	-
chr10	133775416	133775839	-	6	0.003214305	-0.008965231	-	-	-	6	0.004153315	-0.008664361	-	-	-	6	0.004280052	-0.008452285	-	-	-	-	-	-
chr21	38592612	38593284	*DSCR9*	5	0.000717513	0.008430281	-	-	-	5	0.000945214	0.008324126	-	-	-	5	0.000500342	0.008601665	-	-	-	-	-	-
chr7	12443146	12444115	*VWDE*	13	0.000112584	-0.013316657	-	-	-	13	0.000129876	-0.01335698	-	-	-	13	0.000113569	-0.013582365	-	-	-	-	-	-
chr6	31164776	31165413	*HCG27*	11	0.003781004	-0.01450727	-	-	-	-	-	-	-	-	-	11	0.004076824	-0.014595924	-	-	-	-	-	-
chr10	32734072	32735380	-	12	0.000320134	-0.005100663	-	-	-	12	0.00040744	-0.00512847	-	-	-	12	0.000316427	-0.005141089	-	-	-	-	-	-
chr7	1057285	1057944	*C7orf50*	4	0.006667486	0.016278403	-	-	-	4	0.008274103	0.016134067	-	-	-	4	0.006901309	0.016341019	-	-	-	-	-	-
chr11	66034681	66036038	*KLC2*																					
chr1	28051702	28052784	*FAM76A*	14	0.004453309	-3.55E-05	-	-	-	14	0.005855776	-8.53E-05	-	-	-	14	0.005017932	-4.15E-05	-	-	-	-	-	-
chr6	22147182	22148082	*CASC15*																					
chr9	79520919	79521848	*PRUNE2*	9	0.00058595	-0.005738286	-	-	-	9	0.000770103	-0.005793343	-	-	-	9	0.001003363	-0.005604669	-	-	-	-	-	-
chr8	6785692	6786083	*DEFA6*	6	0.00777264	-0.006032297	-	-	-	6	0.009046283	-0.005892522	-	-	-	6	0.008360841	-0.006000262	-	-	-	-	-	-
chr17	6347791	6348225	*PIMREG*	7	0.007791106	0.008268248	-	-	-	-	-	-	-	-	-	-	-	-	-	-	-	-	-	-
chr1	1196457	1196747	*UBE2J2*	3	0.001633743	0.024331172	-	-	-	3	0.001911332	0.024318723	3	0.012875295	0.083350261	3	0.001119203	0.024842644	-	-	-	-	-	-
chr15	90292730	90295301	*MESP1*	15	0.000388913	0.006674648	-	-	-	15	0.000542456	0.006694794	-	-	-	15	0.000417606	0.006677163	-	-	-	-	-	-
chr1	24644596	24646205	*GRHL3*	18	8.99E-06	-0.006235379	-	-	-	18	1.63E-05	-0.006048979	-	-	-	18	2.04E-05	-0.006044688	-	-	-	-	-	-
chr12	54354162	54354590	-	3	0.008356036	-0.015707286	-	-	-	3	0.009141727	-0.015790413	-	-	-	3	0.010840974	-0.015550912	-	-	-	-	-	-
chr10	48439216	48439452	*GDF10*	8	0.005406749	-0.011184385	-	-	-	8	0.00640773	-0.011163375	-	-	-	8	0.005761023	-0.011242051	-	-	-	-	-	-
chr8	135725874	135726595	*ZFAT*	4	0.00988931	0.010609275	-	-	-	4	0.01065259	0.010789525	-	-	-	4	0.008013552	0.011093671	-	-	-	-	-	-
chr10	124638200	124639782	*C10orf88B*																					
chr6	27635558	27638391	-	10	0.000198319	-0.015235167	-	-	-	10	0.000242094	-0.015155718	-	-	-	10	0.000297368	-0.01493822	-	-	-	-	-	-
chr3	184098184	184098596	*CHRD*																					
chr7	42276810	42277410	*GLI3*	14	0.001334893	-0.000623711	-	-	-	14	0.001469699	-0.000653099	-	-	-	14	0.000971301	-0.000617988	-	-	-	-	-	-
chr14	58618986	58619480	*ARMH4*	11	0.009438936	0.000645461	-	-	-	11	0.012178899	0.000617572	-	-	-	11	0.011186611	0.000632694	-	-	-	-	-	-
chr1	144995682	144996395	*LOC653513*																					
chr15	99936927	99937249	-	3	0.009091231	-0.012677651	-	-	-	3	0.011208151	-0.012824119	-	-	-	3	0.010053103	-0.012611801	-	-	-	-	-	-
chr10	1233675	1234983	*ADARB2*	6	0.000424459	-0.013603738	-	-	-	6	0.000452319	-0.013679437	-	-	-	6	0.000591778	-0.013419018	-	-	-	-	-	-
chr17	72897623	72898015	-	4	0.009522119	-0.014224271	-	-	-	4	0.010836708	-0.014233258	-	-	-	4	0.010528435	-0.014065172	-	-	-	-	-	-
chr5	1886956	1888218	*IRX4*	18	0.009231889	-0.009320779	-	-	-	-	-	-	-	-	-	-	-	-	-	-	-	-	-	-
chr19	15342548	15343425	*EPHX3*	11	0.000404385	0.003066686	-	-	-	11	0.000542323	0.002944237	-	-	-	11	0.000386648	0.003155303	-	-	-	-	-	-
chr16	84327245	84328719	*WFDC1*	13	0.001995557	-0.002089963	-	-	-	13	0.002815596	-0.002083624	-	-	-	13	0.002608754	-0.002058368	-	-	-	-	-	-
chr2	31043419	31044164	-	4	0.000416625	0.006411771	-	-	-	4	0.000366012	0.006416117	-	-	-	-	-	-	-	-	-	-	-	-
chr15	79723997	79725642	*MINAR1*	14	0.004173001	-0.006388988	-	-	-	-	-	-	-	-	-	-	-	-	-	-	-	-	-	-
chr17	42092187	42093151	*TMEM101*	14	0.004447835	0.000167411	-	-	-	14	0.005956318	0.000178935	-	-	-	14	0.00615968	0.000267154	-	-	-	-	-	-
chr2	70932281	70933416	*ADD2*	3	0.000411003	0.01777614	-	-	-	3	0.000483177	0.017786793	-	-	-	3	0.000431376	0.017866032	-	-	-	-	-	-
chr12	105380444	105381266	*C12orf45*	3	0.004498803	-0.006045955	-	-	-	3	0.005290602	-0.006180565	3	0.016982908	-0.022256097	3	0.004908556	-0.005986126	-	-	-	-	-	-
chr2	66666267	66668012	*MEIS1*	17	1.90E-05	-0.004328522	-	-	-	17	2.41E-05	-0.004287906	-	-	-	17	3.40E-05	-0.00408214	-	-	-	-	-	-
chr6	33091111	33093109	*HLA-DPB2*	18	0.001083282	0.016131642	-	-	-	18	0.001569049	0.016038849	-	-	-	18	0.000998106	0.016338798	-	-	-	-	-	-
chr7	4814154	4814713	*AP5Z1*	3	0.008813206	0.008447474	-	-	-	3	0.009690329	0.008529558	-	-	-	3	0.006534168	0.008719419	-	-	-	-	-	-
chr22	50515323	50515985	*MLC1*	4	0.008522135	0.015271327	-	-	-	4	0.009971351	0.015199039	-	-	-	4	0.010158827	0.015233203	-	-	-	-	-	-
chr19	3162628	3163454	*GNA15*	7	0.00679015	0.012135477	-	-	-	-	-	-	-	-	-	-	-	-	-	-	-	-	-	-
chr10	105677453	105678654	*STN1*	15	0.002730318	-0.008429161	-	-	-	15	0.004193659	-0.008408504	-	-	-	15	0.003437126	-0.00847338	-	-	-	-	-	-
chr3	167813835	167814230	*GOLIM4*	5	0.00711108	-0.00233205	-	-	-	5	0.008802951	-0.002273708	-	-	-	5	0.008622801	-0.002229175	-	-	-	-	-	-
chr16	85427738	85429035	-	5	0.001316604	0.00694872	-	-	-	5	0.00130415	0.006917685	-	-	-	5	0.00135516	0.006927827	-	-	-	-	-	-
chr2	220282914	220284395	*DES*	9	0.009317037	0.011637096	-	-	-	9	0.011732325	0.011408546	-	-	-	9	0.010748592	0.01151288	-	-	-	-	-	-
chr1	34641601	34642609	*C1orf94*	12	0.007491787	0.010901916	-	-	-	12	0.009105407	0.010784609	-	-	-	12	0.008219504	0.011156653	-	-	-	-	-	-
chr4	901785	902074	*GAK*	3	0.005812164	-0.013447335	-	-	-	3	0.006802131	-0.013443916	-	-	-	3	0.003762002	-0.013861539	-	-	-	-	-	-
chr11	67397595	67399029	*NUDT8*																					
chr4	48018263	48019555	*CNGA1*																					
chr2	236577585	236578808	*AGAP1*	8	0.006019733	-0.00049538	-	-	-	8	0.006468074	-0.000455368	-	-	-	8	0.005271041	-0.000396912	-	-	-	-	-	-
chr22	25335346	25336286	*TMEM211*	7	0.00164324	0.013605334	-	-	-	7	0.002114895	0.013529165	-	-	-	7	0.001493175	0.013725139	-	-	-	-	-	-
chr2	61200075	61200229	*PUS10*	3	0.003558699	0.012898533	3	0.001043746	0.017806948	3	0.003177292	0.013073361	-	-	-	3	0.003114649	0.013000331	-	-	-	-	-	-
chr6	12749567	12750392	*PHACTR1*	5	0.000478547	-0.009304402	-	-	-	5	0.000568101	-0.009287432	-	-	-	5	0.000684436	-0.008755131	-	-	-	-	-	-
chr22	50720813	50723019	*PLXNB2*	8	0.002102035	0.007357074	-	-	-	8	0.002620642	0.007291619	-	-	-	8	0.002004576	0.007291221	-	-	-	-	-	-
chr12	132862024	132864302	*GALNT9*	13	0.001761153	-0.009101604	-	-	-	-	-	-	-	-	-	-	-	-	-	-	-	-	-	-
chr12	7818585	7819180	*APOBEC1*	4	0.006827473	0.007572476	-	-	-	4	0.008119953	0.007729042	-	-	-	4	0.007157205	0.007801193	-	-	-	-	-	-
chr1	163037784	163039184	*RGS4*	16	4.03E-06	-0.017332549	-	-	-	16	5.66E-06	-0.017313215	-	-	-	16	8.73E-06	-0.016877697	-	-	-	-	-	-
chr11	117747030	117748236	*FXYD6*																					
chr12	49373240	49374710	*WNT1*	7	0.009799942	-0.001245087	-	-	-	7	0.011474504	-0.001294987	-	-	-	7	0.011417289	-0.001235458	-	-	-	-	-	-
chr11	17297732	17298537	*NUCB2*	10	0.009419193	-0.005640405	-	-	-	10	0.010457422	-0.005664619	10	0.025538393	-0.021145312	10	0.008617376	-0.00582178	-	-	-	-	-	-
chr7	134001087	134002746	*SLC35B4*	15	0.001672749	0.00546195	-	-	-	15	0.001903225	0.005396624	-	-	-	15	0.002448601	0.005400035	-	-	-	-	-	-
chr3	48486890	48487471	*ATRIP*	3	0.006024354	0.016288179	-	-	-	-	-	-	-	-	-	3	0.004506949	0.016458239	-	-	-	-	-	-
chr7	92861286	92861825	*VPS50*	12	0.009046362	-0.004554464	-	-	-	12	0.011848467	-0.004531875	-	-	-	12	0.009283357	-0.004583189	-	-	-	-	-	-
chr7	95025194	95026937	*PON1*																					
chr1	2382748	2383687	-	6	0.004965536	-0.014545518	-	-	-	6	0.005730024	-0.014546634	-	-	-	-	-	-	-	-	-	-	-	-
chr8	13423496	13424483	*C8orf48*	10	0.007462836	-0.003597934	-	-	-	10	0.009309674	-0.003592706	-	-	-	10	0.008340547	-0.003688257	-	-	-	-	-	-
chr7	142630597	142631266	*TRPV5*	5	0.007263024	0.008371306	-	-	-	5	0.007977728	0.008360458	-	-	-	5	0.007946035	0.008188497	-	-	-	-	-	-
chr18	10414355	10414962	-	4	0.007584474	0.013477621	-	-	-	4	0.009387609	0.013317863	-	-	-	4	0.00986465	0.013317751	-	-	-	-	-	-
chr16	66864375	66865626	*NAE1*	10	0.001759419	0.00322204	-	-	-	10	0.002282851	0.003169644	-	-	-	10	0.001277851	0.003184574	-	-	-	-	-	-
chr17	67956864	67957967	-	6	0.001672749	0.005600788	-	-	-	6	0.002004597	0.005654225	-	-	-	6	0.001988365	0.005553668	-	-	-	-	-	-
chr11	71752705	71753240	*NUMA1*	4	0.009811507	-0.012069453	-	-	-	-	-	-	-	-	-	-	-	-	-	-	-	-	-	-
chr13	93878969	93879769	*GPC6*	9	0.001477141	-0.0083831	-	-	-	9	0.001921165	-0.0083011	-	-	-	9	0.002227494	-0.008275939	-	-	-	-	-	-
chr17	77815779	77816711	*CBX4*	3	0.000768023	0.00450012	-	-	-	3	0.000954433	0.004499428	-	-	-	3	0.001026821	0.00446891	-	-	-	-	-	-
chr3	50649994	50650410	*CISH*																					
chr4	108745001	108745771	*SGMS2*	10	0.009865197	0.005149391	-	-	-	-	-	-	-	-	-	10	0.011272115	0.005194886	-	-	-	-	-	-
chr5	1494980	1495356	*LPCAT1*	4	0.004739199	0.009010531	-	-	-	4	0.005090702	0.009125244	-	-	-	4	0.005430283	0.008897241	-	-	-	-	-	-
chr16	67879671	67880355	*CENPT*																					
chr5	35229788	35231272	*PRLR*	13	7.87E-06	-0.010094942	-	-	-	13	6.87E-06	-0.010146865	-	-	-	13	1.21E-05	-0.009880371	-	-	-	-	-	-
chr13	96705123	96706104	*UGGT2*	10	0.002898107	-0.007631618	-	-	-	10	0.003413015	-0.007600162	-	-	-	10	0.005733139	-0.00715149	-	-	-	-	-	-
chr4	100273969	100274565	*ADH1C*	4	0.006016966	0.019428961	-	-	-	4	0.007336595	0.019213706	-	-	-	4	0.008012921	0.019021083	-	-	-	-	-	-
chr1	45138784	45140266	*ARMH1*																					
chr17	80673675	80675235	*FN3KRP*	13	0.000900453	-0.007619171	-	-	-	13	0.000992904	-0.00767219	-	-	-	13	0.000826328	-0.007785113	-	-	-	-	-	-
chr17	4692162	4692790	*GLTPD2*																					
chr6	29594481	29595661	*GABBR1*	13	0.002548837	0.011044366	-	-	-	13	0.002800818	0.011063688	-	-	-	13	0.002037452	0.011237111	-	-	-	-	-	-
chr17	76354621	76355674	*SOCS3*	7	2.52E-05	0.014668819	7	1.02E-06	0.021050423	7	2.52E-05	0.014241363	-	-	-	7	5.43E-05	0.014275712	-	-	-	-	-	-
chr1	58898552	58898793	*DAB1*	3	0.002601555	-0.047451925	-	-	-	3	0.003249337	-0.047145831	-	-	-	3	0.003857067	-0.04633525	-	-	-	3	0.003205348	-0.211267445
chr6	1624386	1625238	*GMDS*	6	0.004857825	0.010801416	-	-	-	6	0.005614218	0.010801782	-	-	-	6	0.004497625	0.010960715	-	-	-	-	-	-
chr9	132934195	132935030	*NCS1*	8	0.007973025	-0.002453656	-	-	-	8	0.009784341	-0.002588606	-	-	-	8	0.008373654	-0.002627113	-	-	-	-	-	-
chr4	57522493	57524770	*HOPX*	15	0.000782661	-0.012605333	-	-	-	15	0.000953153	-0.012609564	-	-	-	15	0.000877332	-0.012584504	-	-	-	-	-	-
chr1	197871470	197872227	*C1orf53*	11	0.00827366	-0.000724556	-	-	-	-	-	-	-	-	-	11	0.006908548	-0.000824329	-	-	-	-	-	-
chr7	27168962	27171528	*HOXA3*																					
chr1	113050539	113051171	*WNT2B*	6	0.000943142	-0.001779789	-	-	-	6	0.001181054	-0.001834977	-	-	-	6	0.001239346	-0.00182694	-	-	-	-	-	-
chr11	118977379	118978508	*C2CD2L*																					
chr3	140796013	140796183	*SPSB4*	4	0.005496451	-0.010879899	-	-	-	4	0.006369953	-0.010890689	-	-	-	4	0.007700873	-0.010556273	4	0.011428585	-0.016523284	-	-	-
chr1	7913081	7914017	*UTS2*	8	0.000115433	0.015396456	-	-	-	8	0.00015582	0.015339163	-	-	-	8	0.000108158	0.015583169	-	-	-	-	-	-
chr3	53700141	53700263	*CACNA1D*	3	0.000280372	-0.052935571	-	-	-	3	0.000227198	-0.053497061	-	-	-	3	0.00023082	-0.053611689	-	-	-	-	-	-
chr17	8012578	8013680	*ALOXE3*	7	0.001775189	0.012560198	-	-	-	7	0.002336877	0.012461826	-	-	-	7	0.002213593	0.012564755	-	-	-	-	-	-
chr9	130454217	130454671	*MIR3911*																					
chr5	96077898	96078834	*CAST*	5	0.005133629	-0.026403508	-	-	-	-	-	-	-	-	-	5	0.005076977	-0.026572615	-	-	-	-	-	-
chr22	32358655	32359081	-	3	0.004569558	0.008043281	-	-	-	3	0.00518279	0.007826517	-	-	-	3	0.00556914	0.008131554	-	-	-	-	-	-
chr13	27745469	27746386	*USP12*	8	0.001172328	-0.019386387	-	-	-	8	0.001457784	-0.019627377	-	-	-	8	0.00105379	-0.019735426	-	-	-	-	-	-
chr16	67430099	67430454	*TPPP3*																					
chr11	128775026	128776216	*C11orf45*																					
chr17	32581466	32582828	*CCL2*	7	0.000736512	-0.006260191	-	-	-	-	-	-	-	-	-	-	-	-	-	-	-	-	-	-
chr12	131715734	131715773	-	3	0.000514574	-0.013332948	3	0.003216888	-0.015052791	3	0.00052688	-0.013474847	-	-	-	3	0.000288037	-0.013772809	-	-	-	-	-	-
chr3	13521634	13522740	*HDAC11*	14	0.002790116	0.002741414	-	-	-	14	0.003311076	0.002744562	-	-	-	14	0.003404647	0.002677655	-	-	-	-	-	-
chr22	22734186	22734960	-	4	0.000139533	0.020591898	-	-	-	4	0.000188302	0.020398949	-	-	-	4	0.000141005	0.020775328	-	-	-	-	-	-
chr3	24535656	24537801	*THRB*																					
chr14	96000633	96001369	*GLRX5*																					
chr6	30719807	30720484	-	6	6.75E-07	0.014711105	6	0.000797901	0.016304486	6	1.03E-06	0.01464463	6	0.000678352	0.05839117	6	8.10E-07	0.014878513	-	-	-	-	-	-
chr8	145103309	145103942	-	5	0.004106987	0.002765455	-	-	-	-	-	-	-	-	-	5	0.00405278	0.002853624	-	-	-	-	-	-

chr6	10412073	10413394	*TFAP2A*																					
chr1	3620638	3621445	*TP73*	5	0.006501995	-0.016117193	-	-	-	5	0.006061069	-0.016461731	5	0.014920336	-0.059887444	5	0.003438453	-0.016685822	-	-	-	-	-	-
chr7	100025489	100026182	*MEPCE*																					
chr11	63764177	63764925	*OTUB1*	5	0.006827473	-0.002078004	-	-	-	5	0.008520182	-0.001969444	-	-	-	5	0.007241937	-0.00207889	-	-	-	-	-	-
chr6	32094845	32096219	*ATF6B*	31	0.004868288	-0.003773863	-	-	-	-	-	-	-	-	-	-	-	-	-	-	-	-	-	-
chr4	172733760	172734843	*GALNTL6*	13	0.00758248	-0.008704695	-	-	-	-	-	-	-	-	-	-	-	-	-	-	-	-	-	-
chr22	37750979	37751263	-	3	0.000953731	0.012017073	-	-	-	3	0.001033195	0.011908086	-	-	-	3	0.001228636	0.011531429	-	-	-	-	-	-
chr1	854766	855649	*LINC02593*																					
chr3	190335248	190336306	*IL1RAP*	3	0.000193905	0.022612063	-	-	-	3	0.000115518	0.022769857	-	-	-	3	0.000210538	0.022989535	-	-	-	-	-	-
chr1	247581408	247582066	*NLRP3*	7	0.004664699	-0.001483574	-	-	-	7	0.005732041	-0.001424804	-	-	-	7	0.00425136	-0.001482735	-	-	-	-	-	-
chr8	145637966	145639652	*CPSF1*																					
chr6	127839822	127840334	*SOGA3*	6	0.006090182	-0.005835561	-	-	-	6	0.007259213	-0.00582468	-	-	-	6	0.007620953	-0.005637457	-	-	-	-	-	-
chr19	42702481	42703852	*DEDD2*	5	0.00255692	0.00216578	-	-	-	5	0.003147809	0.002166257	-	-	-	5	0.003154935	0.002106271	-	-	-	-	-	-
chr4	20253277	20255985	*SLIT2*	20	0.003225038	-0.002044244	-	-	-	20	0.004196159	-0.002000604	-	-	-	-	-	-	-	-	-	-	-	-
chr6	30457711	30458730	*HLA-E*	18	0.006120223	-0.003153817	-	-	-	18	0.007934923	-0.003192452	-	-	-	18	0.004551008	-0.003404249	-	-	-	-	-	-
chr7	157579211	157579880	*PTPRN2*	3	0.007135693	0.015937989	-	-	-	3	0.008771385	0.01570941	-	-	-	3	0.007214078	0.015922578	-	-	-	-	-	-
chr8	145027226	145028886	*PLEC*	13	2.58E-07	0.015913412	-	-	-	13	3.52E-07	0.015891849	-	-	-	13	3.12E-07	0.015935367	-	-	-	-	-	-
chr5	346365	346987	*AHRR*	3	0.005183976	-0.016700038	-	-	-	3	0.006629564	-0.016405652	-	-	-	3	0.00661006	-0.016520746	-	-	-	-	-	-
chr11	84431163	84432312	*DLG2*	8	0.004134592	-0.005113305	-	-	-	8	0.005205806	-0.005080482	-	-	-	8	0.005341166	-0.005034893	-	-	-	-	-	-
chr19	56651829	56652545	*ZNF444*	11	0.005433568	-0.00128829	-	-	-	11	0.007049236	-0.001317832	-	-	-	11	0.005047738	-0.001382273	-	-	-	-	-	-
chr7	47293799	47294330	-	5	0.003478166	-0.002418701	5	0.003424665	-0.001934295	5	0.003938292	-0.002312328	-	-	-	5	0.004477192	-0.002139315	-	-	-	-	-	-
chr5	114937535	114938640	*TICAM2*																					
chr7	71868406	71868494	*CALN1*	3	3.58E-07	-0.027707169	3	0.000263873	-0.029073641	3	2.03E-07	-0.027979648	-	-	-	3	1.97E-07	-0.028134149	-	-	-	-	-	-
chr10	79470169	79471118	-	5	0.001770193	-0.009254766	-	-	-	5	0.001925285	-0.009309634	-	-	-	5	0.002057165	-0.008886426	-	-	-	-	-	-
chr20	57267176	57267680	*NPEPL1*	7	0.007500868	0.016370234	-	-	-	7	0.009778569	0.016075416	-	-	-	7	0.009112018	0.016309546	-	-	-	-	-	-
chr2	241807924	241808757	*AGXT*	7	0.008676328	0.003772825	-	-	-	-	-	-	-	-	-	-	-	-	-	-	-	-	-	-
chr1	43205605	43206309	*CLDN19*	6	0.002908746	0.009829217	-	-	-	6	0.003819994	0.009723345	-	-	-	6	0.004184868	0.009578502	-	-	-	-	-	-
chr10	129845509	129846082	*PTPRE*	6	0.002023429	-0.008268095	-	-	-	6	0.002401684	-0.008312824	-	-	-	6	0.002094616	-0.00837006	-	-	-	-	-	-
chr5	42423947	42425093	*GHR*	13	0.006806946	-0.006048988	-	-	-	13	0.009388703	-0.005940847	-	-	-	13	0.006417245	-0.005951356	-	-	-	-	-	-
chr5	190822	192650	*LRRC14B*	16	5.22E-22	-0.017532387	-	-	-	16	1.76E-21	-0.017411061	-	-	-	16	1.57E-21	-0.017773152	16	4.23E-07	-0.017995263	-	-	-
chr4	186731926	186732207	*SORBS2*	3	0.004886137	0.010211261	-	-	-	3	0.005877833	0.010118211	-	-	-	3	0.00642931	0.009759055	-	-	-	-	-	-
chr3	157019345	157019556	*VEPH1*	3	0.004501588	0.008271393	-	-	-	3	0.005342817	0.008216623	-	-	-	3	0.005690312	0.007928031	-	-	-	-	-	-
chr3	147140880	147142415	-	13	5.09E-05	-0.006263032	-	-	-	13	7.56E-05	-0.006292187	-	-	-	13	7.94E-05	-0.006054905	-	-	-	-	-	-
chr6	31869518	31870840	*C2*																					
chr19	58094517	58095659	*ZIK1*																					
chr19	39522548	39523626	*FBXO27*	10	0.000324175	0.016377779	-	-	-	10	0.000468115	0.016114101	-	-	-	10	0.000243891	0.016567249	-	-	-	-	-	-
chr16	30485296	30485966	*ITGAL*	7	0.005381015	0.01541582	-	-	-	7	0.006930084	0.015260859	-	-	-	7	0.007773292	0.014579619	-	-	-	-	-	-
chr1	239549600	239550303	*CHRM3*	8	0.001229438	-0.009534189	-	-	-	8	0.001070777	-0.009622769	-	-	-	8	0.001155218	-0.009385867	-	-	-	-	-	-
chr9	137966584	137967652	*OLFM1*	12	0.000536331	-0.005899971	-	-	-	12	0.000707888	-0.005883274	-	-	-	12	0.000805075	-0.005401937	-	-	-	-	-	-
chr11	86510915	86512100	*PRSS23*	10	2.27E-12	0.009142299	-	-	-	10	4.27E-12	0.00907588	-	-	-	10	1.32E-12	0.009215103	-	-	-	-	-	-
chr1	247653903	247654811	*OR2W5P*	6	0.007993337	0.006894012	-	-	-	6	0.010157113	0.006939223	-	-	-	6	0.01100951	0.006837477	-	-	-	-	-	-
chr16	21170107	21170814	*DNAH3*																					
chr1	166049757	166050477	*FAM78B*	4	0.001873085	-0.010875808	-	-	-	-	-	-	-	-	-	-	-	-	-	-	-	-	-	-
chr3	172312523	172313107	-	4	0.003033508	-0.029414958	-	-	-	4	0.003454433	-0.029479943	-	-	-	4	0.00405278	-0.029026186	-	-	-	-	-	-
chr9	90112086	90114156	*DAPK1*	21	0.006723832	-0.005728172	-	-	-	21	0.008338184	-0.005725953	-	-	-	21	0.010592395	-0.005420113	-	-	-	-	-	-
chr21	19858957	19859472	-	3	0.006617658	0.001996817	-	-	-	-	-	-	-	-	-	-	-	-	-	-	-	-	-	-
chr1	54881486	54881929	-	6	0.003225951	0.004546897	-	-	-	6	0.003968148	0.004482179	-	-	-	6	0.003813861	0.004462065	-	-	-	-	-	-
chr12	32111966	32113101	*RESF1*	13	0.006653562	-0.002155175	-	-	-	13	0.007991781	-0.002137495	-	-	-	13	0.007178472	-0.002218649	-	-	-	-	-	-
chr5	148017424	148018093	*HTR4*																					
chr6	111195301	111195976	*AMD1*	9	0.001869621	-0.003120012	-	-	-	9	0.002364594	-0.003117142	-	-	-	9	0.001709055	-0.003179935	-	-	-	-	-	-
chr17	75445905	75446661	*SEPTIN9*	9	0.005018118	0.009367473	-	-	-	9	0.006537019	0.009274401	-	-	-	9	0.006817308	0.0091546	-	-	-	-	-	-
chr12	6307048	6309025	*CD9*	7	0.004928118	0.014081145	-	-	-	7	0.005996916	0.014040336	-	-	-	7	0.006343021	0.014012819	-	-	-	-	-	-
chr17	45949677	45949878	-	5	4.08E-07	0.037408698	5	0.008063032	0.033935316	5	4.68E-07	0.037451523	5	0.003528633	0.11041269	5	2.70E-07	0.037871737	-	-	-	-	-	-
chr13	114291735	114292740	*TFDP1*	11	0.008622795	0.000863617	-	-	-	11	0.009307796	0.000896543	-	-	-	11	0.007669126	0.001047634	-	-	-	-	-	-
chr11	8228022	8228508	-	3	0.00066326	-0.009371198	-	-	-	3	0.000781727	-0.009338678	-	-	-	3	0.000858391	-0.009209994	-	-	-	-	-	-
chr18	28622474	28623390	*DSC3*	14	0.004901519	-0.008680049	-	-	-	14	0.006177788	-0.008655778	-	-	-	14	0.008278428	-0.008417242	-	-	-	-	-	-
chr17	79225309	79226323	*SLC38A10*	7	0.007791106	-0.011055029	-	-	-	7	0.010147506	-0.010862492	-	-	-	7	0.011488207	-0.010240042	-	-	-	-	-	-
chr19	52956310	52957180	*ZNF578*	11	0.00452359	-0.000330413	-	-	-	11	0.006056693	-0.000232133	-	-	-	11	0.005813959	0.0002245	-	-	-	-	-	-
chr11	18416535	18418093	*LDHA*	12	0.007457488	-0.001696838	-	-	-	-	-	-	-	-	-	12	0.006640839	-0.001732828	-	-	-	-	-	-
chr4	1750374	1750476	-	3	0.000821295	-0.011793796	-	-	-	3	0.00088269	-0.011930025	-	-	-	3	0.000624069	-0.012092216	-	-	-	-	-	-
chr2	149855950	149856468	*KIF5C*	3	0.008944509	-0.018493581	-	-	-	3	0.010710856	-0.018096294	-	-	-	3	0.009321788	-0.018656565	-	-	-	-	-	-
chr3	170136159	170137240	*CLDN11*	12	0.00048311	-0.008881424	-	-	-	-	-	-	-	-	-	-	-	-	-	-	-	-	-	-
chr4	53727431	53728510	*RASL11B*	7	0.00359499	-0.009911899	-	-	-	7	0.003775043	-0.010157475	-	-	-	7	0.005047738	-0.009553524	-	-	-	-	-	-
chr16	75150456	75150880	*LDHD*	8	4.07E-05	0.024855109	-	-	-	8	5.27E-05	0.024784008	-	-	-	8	0.000110585	0.023864093	8	0.00024868	0.039259328	-	-	-
chr1	156072803	156074182	*LMNA*	4	0.000730077	0.016465183	-	-	-	4	0.000872749	0.016193966	-	-	-	4	0.001079453	0.015957317	-	-	-	-	-	-
chr19	10220013	10220911	*P2RY11*																					
chr1	91190891	91192803	-	17	0.00020892	0.005216748	-	-	-	17	0.000259124	0.005203867	-	-	-	-	-	-	-	-	-	-	-	-
chr16	55512521	55515248	*MMP2*	22	0.005633635	-0.005211354	-	-	-	22	0.007516332	-0.005171259	-	-	-	-	-	-	-	-	-	-	-	-
chr6	41168185	41169391	*TREML2*	8	0.000320254	-0.009009859	-	-	-	8	0.000440318	-0.008864489	-	-	-	8	0.000566703	-0.008365926	-	-	-	-	-	-
chr1	154237531	154238265	*UBAP2L*	3	0.002804509	-0.035874369	-	-	-	3	0.002906595	-0.036578087	-	-	-	3	0.003313243	-0.035520728	-	-	-	-	-	-
chr10	115438155	115439884	*CASP7*	17	6.24E-06	-0.001872104	-	-	-	-	-	-	-	-	-	17	5.81E-06	-0.001915644	-	-	-	-	-	-
chr14	100068637	100070564	*CCDC85C*	8	0.001329002	0.007990615	-	-	-	8	0.00138258	0.008033344	-	-	-	8	0.000370429	0.008419616	-	-	-	-	-	-
chr2	189155551	189156621	*GULP1*	10	0.002930649	-0.005679793	-	-	-	10	0.003648691	-0.005569435	-	-	-	10	0.005286701	-0.005467373	-	-	-	-	-	-
chr12	1939964	1940652	*CACNA2D4*																					
chr5	157001092	157002175	*ADAM19*	5	0.007451956	0.008026186	-	-	-	5	0.00876326	0.007909735	-	-	-	5	0.007245471	0.007808927	-	-	-	-	-	-
chr16	8805422	8807308	*ABAT*	16	1.64E-05	0.021425684	-	-	-	16	6.88E-06	0.021750018	-	-	-	16	3.09E-06	0.022117472	-	-	-	-	-	-
chr22	28838631	28839352	*TTC28*	6	0.000311181	-0.012490585	-	-	-	6	0.000331806	-0.012394174	-	-	-	6	0.000463641	-0.012283682	-	-	-	-	-	-
chr7	25219543	25220412	*C7orf31*	12	0.003655064	-0.007651863	-	-	-	12	0.004026939	-0.007712384	-	-	-	-	-	-	-	-	-	-	-	-
chr12	25403102	25405280	*KRAS*	27	1.89E-05	-0.006882384	-	-	-	27	3.26E-05	-0.006910253	-	-	-	27	1.74E-05	-0.006994253	-	-	-	-	-	-
chr20	57330819	57331560	-	4	0.00030061	0.010921137	-	-	-	4	0.000399931	0.010829277	-	-	-	4	0.000288668	0.010520833	-	-	-	-	-	-
chr20	17943403	17944845	*MGME1*																					
chr3	128690194	128690824	*CFAP92*	3	0.000270516	-0.022350841	-	-	-	3	0.000334769	-0.022080864	-	-	-	3	0.000372386	-0.022054683	-	-	-	-	-	-
chr6	31695599	31697280	*DDAH2*	31	0.000155986	0.0078678	-	-	-	-	-	-	-	-	-	31	5.12E-05	0.008155336	-	-	-	-	-	-
chr17	10600507	10602034	*ADPRM*																					
chr10	88295210	88295591	-	4	0.005626189	0.012452379	-	-	-	4	0.00709373	0.01223241	-	-	-	4	0.005586472	0.012444219	-	-	-	-	-	-
chr16	85783863	85785218	*C16orf74*	7	0.009272857	0.005561107	-	-	-	-	-	-	-	-	-	-	-	-	-	-	-	-	-	-
chr17	79419366	79420424	*BAHCC1*																					
chr3	157260159	157261595	*SLC66A1L*	14	0.001960872	-0.004041874	-	-	-	14	0.002676975	-0.004041572	-	-	-	14	0.003057494	-0.003961	-	-	-	-	-	-
chr17	59476790	59477846	*TBX2*	14	0.007964104	-0.001086315	-	-	-	14	0.009418111	-0.001083474	-	-	-	14	0.006387249	-0.001074609	-	-	-	-	-	-
chr4	3671067	3671697	-	5	0.003092866	0.018055948	-	-	-	5	0.003805847	0.017939871	-	-	-	5	0.002699304	0.018291161	-	-	-	-	-	-
chr3	138763424	138764372	*PRR23C*	12	0.009317037	0.012927687	-	-	-	12	0.011230697	0.012689106	-	-	-	12	0.011508699	0.013038715	-	-	-	-	-	-
chr5	124043354	124044098	*ZNF608*	5	0.004567925	-0.015157925	-	-	-	5	0.004825476	-0.014855088	-	-	-	5	0.00482052	-0.015274925	-	-	-	-	-	-
chr6	111888446	111888870	*TRAF3IP2*																					
chr6	170553133	170555276	-	9	1.27E-07	-0.036130603	-	-	-	9	1.13E-07	-0.036368113	-	-	-	9	1.05E-07	-0.036269563	-	-	-	-	-	-
chr6	42878931	42879409	*PTCRA*	3	0.008385696	0.019578175	-	-	-	3	0.00984824	0.019381801	-	-	-	3	0.009427316	0.019621357	-	-	-	-	-	-
chr7	6048837	6048965	*AIMP2*																					
chr17	27070369	27071119	*TRAF4*	9	0.006603717	0.002375077	-	-	-	-	-	-	-	-	-	9	0.008122071	0.002258443	-	-	-	-	-	-
chr6	30657882	30659043	*NRM*																					
chr12	126467559	126468899	*LINC00939*	9	0.00422921	-0.007966133	-	-	-	9	0.005143698	-0.007989456	-	-	-	9	0.004341449	-0.008049658	-	-	-	-	-	-
chr1	221509067	221509763	*LINC02817*	3	1.35E-05	-0.019248184	-	-	-	3	1.68E-05	-0.019040137	-	-	-	3	1.62E-05	-0.019334935	-	-	-	-	-	-
chr17	1421079	1421517	*INPP5K*																					
chr1	36771493	36773099	*SH3D21*	15	0.005401161	0.001928713	-	-	-	15	0.006484437	0.001918186	-	-	-	15	0.005774494	0.001862565	-	-	-	-	-	-
chr20	34129142	34130163	*ERGIC3*	14	0.003023738	0.002998069	-	-	-	14	0.003952024	0.002958471	-	-	-	14	0.003637105	0.002919802	-	-	-	-	-	-
chr1	158323482	158324471	*CD1E*	4	0.00112526	-0.010712967	-	-	-	4	0.001249768	-0.010657076	-	-	-	4	0.001335548	-0.010816828	-	-	-	-	-	-
chr20	58629939	58630954	*C20orf197*	7	0.005810528	-0.007669198	-	-	-	7	0.007366566	-0.007616766	-	-	-	-	-	-	-	-	-	-	-	-
chr15	31617459	31617764	*KLF13*	5	0.001978069	0.008706541	-	-	-	5	0.002048749	0.008767069	-	-	-	5	0.002603092	0.008656379	-	-	-	-	-	-
chr20	58325159	58326180	*PHACTR3*	3	0.003563008	-0.009712924	-	-	-	3	0.004060112	-0.009902799	-	-	-	3	0.003806848	-0.009837872	-	-	-	-	-	-
chr17	8056492	8057770	*PER1*	5	0.001689885	-0.004197997	-	-	-	5	0.002051527	-0.004190087	-	-	-	5	0.00205921	-0.004126004	-	-	-	-	-	-
chr12	49688844	49689212	*PRPH*	4	0.00893019	0.019944102	-	-	-	4	0.009073147	0.019959959	-	-	-	-	-	-	-	-	-	-	-	-
chr12	4140243	4140993	-	4	0.006874935	0.004557169	-	-	-	-	-	-	-	-	-	4	0.007346664	0.004669336	-	-	-	-	-	-
chr6	21856149	21856613	*CASC15*	3	0.004067202	-0.011569098	3	0.015540939	-0.017287426	3	0.004271909	-0.011260901	-	-	-	3	0.005124982	-0.011413653	-	-	-	-	-	-

chr20	36155979	36157405	*BLCAP*	43	0.0048589	-0.002727026	-	-	-	-	-	-	-	-	-	-	-	-	-	-	-	-	-	-
chr16	85207569	85208316	-	3	0.009246786	0.010369206	-	-	-	-	-	-	-	-	-	-	-	-	-	-	-	-	-	-
chr8	65289139	65290484	*MIR124-2*																					
chr14	34529059	34529242	-	3	0.008836367	0.019876264	-	-	-	3	0.010747653	0.019711466	-	-	-	3	0.009228974	0.020047945	-	-	-	-	-	-
chr6	32811690	32813715	*PSMB8*																					
chr7	116592956	116594430	*ST7*																					
chr17	1956958	1958412	*HIC1*																					
chr22	36903611	36904394	*FOXRED2*	3	0.007763244	-0.003409226	-	-	-	3	0.008874179	-0.003427907	-	-	-	3	0.008507809	-0.003282486	-	-	-	-	-	-
chr13	114846655	114847515	*RASA3*	4	0.004034828	0.005036344	-	-	-	4	0.004986597	0.00506516	-	-	-	4	0.00461078	0.005030004	-	-	-	-	-	-
chr1	153232348	153234037	*LORICRIN*	6	0.000122165	-0.011210561	-	-	-	-	-	-	-	-	-	-	-	-	-	-	-	-	-	-
chr6	106433985	106434623	-	5	0.002418536	0.014705586	-	-	-	5	0.002625356	0.014859259	-	-	-	5	0.00141172	0.015994306	-	-	-	-	-	-
chr13	103451435	103452789	*BIVM*																					
chr6	35181888	35182910	*SCUBE3*	5	0.009677302	0.005820406	-	-	-	5	0.009800029	0.005914697	-	-	-	-	-	-	-	-	-	-	-	-
chr10	6441693	6443025	-	3	0.002423278	-0.012559872	-	-	-	-	-	-	-	-	-	-	-	-	-	-	-	-	-	-
chr12	118498668	118499294	*WSB2*	4	0.003411938	-0.005376305	-	-	-	4	0.003890058	-0.005364397	-	-	-	4	0.002995026	-0.005612071	-	-	-	-	-	-
chr10	135050343	135052004	*VENTX*	27	0.005976156	0.00747605	-	-	-	-	-	-	-	-	-	-	-	-	-	-	-	-	-	-
chr2	241975035	241976658	*SNED1*	8	2.10E-11	-0.03406702	-	-	-	8	3.84E-11	-0.033771545	-	-	-	8	3.32E-12	-0.03472475	-	-	-	-	-	-
chr20	2674548	2675418	*EBF4*	4	0.005171993	0.018596923	-	-	-	4	0.005092481	0.018807734	-	-	-	-	-	-	-	-	-	-	-	-
chr9	136075279	136076059	-	4	0.006970061	-0.007575892	-	-	-	4	0.008306031	-0.007685805	4	0.019012523	-0.055583871	4	0.009575374	-0.007354872	-	-	-	-	-	-
chr19	3785573	3786125	*MATK*	3	0.00581327	-0.009840787	-	-	-	3	0.006636063	-0.009863452	-	-	-	3	0.004401375	-0.010134059	-	-	-	-	-	-
chr6	28602513	28603437	-	34	0.006730098	-0.002707009	-	-	-	34	0.007550595	-0.002704287	-	-	-	34	0.008917351	-0.00253502	-	-	-	-	-	-
chr16	18812258	18813278	*ARL6IP1*	14	0.006600905	-0.003450229	-	-	-	14	0.007489435	-0.00349748	-	-	-	14	0.007268564	-0.003484095	-	-	-	-	-	-
chr20	707906	708659	-	3	0.005673121	0.006082301	-	-	-	3	0.00710351	0.005983906	-	-	-	3	0.006832182	0.006062779	-	-	-	-	-	-
chr12	49730687	49731991	*C1QL4*	12	0.000456974	-0.004428629	-	-	-	12	0.000563353	-0.004387145	-	-	-	12	0.000342446	-0.004404877	-	-	-	-	-	-
chr5	150161299	150163018	*SMIM3*	4	1.47E-05	0.01496976	-	-	-	4	1.92E-05	0.014873474	-	-	-	4	1.14E-05	0.01523502	-	-	-	-	-	-
chr3	49315355	49316322	*C3orf62*																					
chr9	138391055	138393651	*C9orf116*																					
chr7	100136013	100136517	*AGFG2*	5	0.004327518	-0.00167343	-	-	-	5	0.005554543	-0.001748862	-	-	-	5	0.005773138	-0.001724391	-	-	-	-	-	-
chr5	93953562	93955080	*KIAA0825*																					
chr4	13545639	13546673	*NKX3-2*	12	0.00912193	-0.007811775	-	-	-	12	0.011643649	-0.007740191	-	-	-	-	-	-	-	-	-	-	-	-
chr14	77294264	77294668	*LRRC74A*	6	0.001641333	0.015265484	-	-	-	6	0.001927575	0.015393934	-	-	-	6	0.001808122	0.015274321	-	-	-	-	-	-
chr13	79980322	79980793	*RBM26*																					
chr2	204103311	204104171	*CYP20A1*	8	0.002242266	-0.004714489	-	-	-	-	-	-	-	-	-	8	0.002826706	-0.004710015	-	-	-	-	-	-
chr7	27212357	27214296	*HOXA10*																					
chr15	60770518	60771505	*ICE2*	7	0.000447597	0.004330224	-	-	-	-	-	-	-	-	-	-	-	-	-	-	-	-	-	-
chr17	75032801	75033339	-	4	0.001595194	-0.015545895	-	-	-	4	0.001804409	-0.015507457	-	-	-	4	0.001431842	-0.015576979	4	0.022839732	-0.023321411	-	-	-
chr8	41523177	41523968	*ANK1*																					
chr13	46424943	46425929	*SIAH3*	10	5.80E-06	-0.009327744	-	-	-	10	8.25E-06	-0.009272583	-	-	-	10	9.93E-06	-0.009229254	-	-	-	-	-	-
chr7	157405965	157406737	*PTPRN2*	6	9.89E-10	-0.043422181	6	2.07E-06	-0.04713673	6	1.51E-09	-0.043258784	-	-	-	6	2.16E-09	-0.04299108	-	-	-	-	-	-
chr9	110249749	110251983	*KLF4*	8	0.001617123	0.002536416	-	-	-	8	0.00182029	0.002722391	-	-	-	8	0.002376107	0.002579466	-	-	-	-	-	-
chr7	99005923	99006858	*BUD31*																					
chr1	36553577	36555392	*ADPRS*																					
chr9	136131118	136131739	*ABO*	5	0.001505786	0.02753718	-	-	-	5	0.00200659	0.027276394	-	-	-	5	0.001013261	0.028153962	-	-	-	-	-	-
chr12	3393711	3393872	*TSPAN9*	3	0.008918111	0.009158709	-	-	-	3	0.008471598	0.009256866	-	-	-	3	0.009903199	0.009487284	-	-	-	-	-	-
chr11	124737554	124738157	*ROBO3*	3	0.000370986	-0.010002799	-	-	-	3	0.000286613	-0.009703986	-	-	-	3	0.000565179	-0.009730694	-	-	-	-	-	-
chr6	167047310	167047877	*RPS6KA2*	3	0.004421301	-0.020145111	3	0.035027625	-0.025152053	3	0.005472144	-0.019930601	-	-	-	3	0.003093258	-0.02067956	-	-	-	-	-	-
chr19	8578767	8579199	*ZNF414*	9	0.002543502	-0.001826123	-	-	-	9	0.003137399	-0.001838275	-	-	-	9	0.001015696	-0.001849751	-	-	-	-	-	-
chr6	30042137	30043418	*RNF39*	26	0.00562156	-0.001425697	-	-	-	26	0.00722975	-0.001333208	-	-	-	26	0.004481336	-0.001362608	-	-	-	-	-	-
chr11	89867385	89868104	*NAALAD2*	14	0.000460833	-0.012644877	-	-	-	14	0.000791474	-0.012408396	-	-	-	14	0.001013866	-0.012145118	-	-	-	-	-	-
chr6	111926749	111928204	*TRAF3IP2*	7	0.004399737	-0.012470293	-	-	-	7	0.004567283	-0.012547046	-	-	-	7	0.00519996	-0.012494701	-	-	-	-	-	-
chr19	51893804	51894617	*C19orf84*																					
chr19	55692003	55693151	*PTPRH*																					
chr4	7512177	7512528	*SORCS2*	5	0.002385251	-0.019843886	-	-	-	5	0.003053453	-0.019721973	-	-	-	5	0.001507568	-0.020404916	-	-	-	-	-	-
chr1	154475068	154475688	*SHE*																					
chr2	51254102	51255480	*NRXN1*	8	0.000470423	0.016055119	-	-	-	-	-	-	-	-	-	-	-	-	-	-	-	-	-	-
chr21	47169994	47170466	*PCBP3*	5	0.002327381	0.005428195	-	-	-	5	0.002724318	0.005341411	-	-	-	5	0.002617515	0.005151874	-	-	-	-	-	-
chr14	94423111	94424707	*ASB2*	11	0.001518808	0.007320827	-	-	-	11	0.001763314	0.007315795	-	-	-	11	0.00139388	0.007369624	-	-	-	-	-	-
chr17	75765496	75766979	-	3	0.000710338	0.010082673	-	-	-	-	-	-	-	-	-	3	0.000778199	0.010074654	-	-	-	-	-	-
chr2	191878353	191879164	*STAT1*	12	0.001487264	-0.00036927	-	-	-	12	0.001416167	-0.000375719	-	-	-	12	0.001919703	-0.000397184	-	-	-	-	-	-
chr2	97524719	97525009	*ANKRD39*	5	3.49E-06	0.018195091	-	-	-	5	4.39E-06	0.018207667	-	-	-	5	2.59E-06	0.018429816	-	-	-	-	-	-
chr22	33195343	33196425	*SYN3*																					
chr4	5890358	5890751	*CRMP1*	7	0.005673121	-0.005082064	-	-	-	7	0.006533111	-0.005137493	-	-	-	-	-	-	-	-	-	-	-	-
chr16	426536	427756	*LOC100134368*																					
chr9	10611904	10612567	*PTPRD*	3	0.004706817	0.002069857	-	-	-	3	0.005530644	0.001988584	-	-	-	3	0.005756749	0.002265545	-	-	-	-	-	-
chr14	45722289	45723370	*MIS18BP1*	13	0.000335526	0.010405464	-	-	-	13	0.000422939	0.010353653	-	-	-	13	0.000209957	0.010865323	-	-	-	-	-	-
chr3	3151899	3152916	*IL5RA*	6	0.001633743	0.01494043	-	-	-	6	0.001794452	0.014959614	-	-	-	6	0.0022429	0.014593923	-	-	-	-	-	-
chr15	68110824	68112070	*SKOR1*	8	0.003142703	0.018062333	-	-	-	8	0.003695064	0.018047399	-	-	-	8	0.003211248	0.018175213	-	-	-	-	-	-
chr16	12096413	12096947	*SNX29*	3	0.008033773	-0.006386535	-	-	-	3	0.006775664	-0.006565574	-	-	-	3	0.006740631	-0.00651151	-	-	-	-	-	-
chr3	51428079	51428687	*RBM15B*	11	0.003804211	0.000832231	-	-	-	11	0.003813244	0.000993885	-	-	-	11	0.003308781	0.000927272	-	-	-	-	-	-
chr4	111532410	111533547	-	8	0.005930982	-0.004132052	-	-	-	-	-	-	-	-	-	-	-	-	-	-	-	-	-	-
chr6	169422647	169423797	-	5	0.009842513	0.016286033	-	-	-	5	0.010295352	0.01630014	-	-	-	5	0.010821474	0.01611654	-	-	-	-	-	-
chr6	30710462	30712373	*FLOT1*																					
chr14	101348158	101350872	*MIR127*																					
chr4	186456009	186456976	*PDLIM3*	11	0.003045303	-0.003051425	-	-	-	-	-	-	-	-	-	11	0.003504126	-0.002922758	-	-	-	-	-	-
chr11	111168594	111169865	*COLCA1*																					
chr14	37641191	37642356	*SLC25A21*																					
chr7	157451243	157451618	*PTPRN2*	4	0.006394197	0.00408384	-	-	-	4	0.007085652	0.004229406	-	-	-	4	0.00758736	0.003970966	-	-	-	-	-	-
chr11	7535611	7535983	*PPFIBP2*	4	0.00866702	-0.020282835	-	-	-	4	0.010340359	-0.020233547	-	-	-	4	0.010574513	-0.020103479	-	-	-	-	-	-
chr19	6710658	6711092	*C3*	3	0.004673694	0.022160583	3	0.009298347	0.02958175	3	0.004265908	0.022455645	-	-	-	3	0.006003747	0.021808641	-	-	-	-	-	-
chr8	143915496	143916385	*GML*	11	0.006806946	0.014141221	-	-	-	11	0.008557897	0.014040636	-	-	-	11	0.005916751	0.014388481	-	-	-	-	-	-
chr3	52811637	52814643	*ITIH1*	16	7.98E-06	-0.006264133	-	-	-	16	9.99E-06	-0.006182928	-	-	-	16	1.02E-05	-0.006241182	-	-	-	-	-	-
chr18	44789949	44790841	-	4	0.00165769	-0.012823761	4	0.000697643	-0.012968838	4	0.002161106	-0.012603312	-	-	-	4	0.002471014	-0.012248002	-	-	-	-	-	-
chr14	105308891	105309812	-	3	0.00458663	0.007042696	-	-	-	3	0.004823179	0.007007907	-	-	-	3	0.005079949	0.007183139	-	-	-	-	-	-
chr16	31453406	31453948	*ZNF843*	4	0.001177932	0.017726073	-	-	-	4	0.00150569	0.017339162	-	-	-	4	0.001300011	0.017665596	-	-	-	-	-	-
chr13	23412250	23412996	-	5	0.001901717	0.033717308	-	-	-	5	0.001507598	0.034219181	5	4.36E-06	0.19540354	5	0.002386718	0.03344787	-	-	-	-	-	-
chr1	206729034	206729685	*RASSF5*	7	0.009542417	0.006404335	-	-	-	7	0.011831388	0.006356348	-	-	-	7	0.014032221	0.006177551	-	-	-	-	-	-
chr12	50355995	50357142	*AQP5*																					
chr8	26433537	26434069	*DPYSL2*	4	0.006029793	0.02019256	-	-	-	4	0.007223031	0.020088555	-	-	-	4	0.007204194	0.020133759	-	-	-	-	-	-
chr16	75589282	75590036	*TMEM231*	7	0.003585455	-0.001895758	-	-	-	7	0.004424795	-0.001838124	-	-	-	-	-	-	-	-	-	-	-	-
chr1	10487953	10488281	*CENPS*																					
chr7	128864205	128864907	*AHCYL2*	6	0.007395683	-0.000236375	-	-	-	6	0.007812001	-0.000248149	-	-	-	6	0.008782256	-0.000135763	-	-	-	-	-	-
chr3	194118478	194119188	*GP5*	7	0.005878908	-0.006193595	-	-	-	7	0.007146836	-0.006241695	-	-	-	7	0.006823503	-0.00611927	7	0.016529446	-0.001855987	-	-	-
chr1	200009927	200010626	*NR5A2*	4	0.004936136	0.005919159	-	-	-	4	0.005663072	0.006010916	-	-	-	4	0.003650477	0.005712769	-	-	-	-	-	-
chr22	38851884	38852154	*KCNJ4*	4	0.007489488	-0.012034453	-	-	-	4	0.009330315	-0.011982042	-	-	-	4	0.007082098	-0.012169049	-	-	-	-	-	-
chr15	80215376	80216491	*ST20*																					
chr15	37190548	37190998	*MEIS2*	5	0.001263864	0.007987883	-	-	-	5	0.001474664	0.007883116	-	-	-	5	0.001748977	0.00805764	-	-	-	-	-	-
chr8	145741413	145742229	*LRRC14*																					
chr3	111717534	111718457	*TAGLN3*	14	0.005354906	-0.009299046	-	-	-	14	0.007278584	-0.009115411	-	-	-	14	0.01148056	-0.008720631	-	-	-	-	-	-
chr2	220309434	220310232	*SPEG*	6	0.001640571	0.012258057	-	-	-	6	0.001981734	0.012268604	-	-	-	6	0.001752455	0.012190767	-	-	-	-	-	-
chr16	70837763	70838524	*VAC14*	3	4.91E-06	-0.016416531	3	0.000459075	-0.012157509	3	6.33E-06	-0.016464244	-	-	-	3	5.10E-06	-0.016134975	-	-	-	-	-	-
chr2	168150602	168151051	-	5	0.003309804	-0.009333353	-	-	-	5	0.003695064	-0.009333957	-	-	-	5	0.00369446	-0.009197733	-	-	-	-	-	-
chr9	135036217	135037343	*NTNG2*	9	0.002808433	-0.002796471	-	-	-	9	0.003367942	-0.002802963	-	-	-	9	0.003304899	-0.002755092	-	-	-	-	-	-
chr14	105553150	105554214	-	5	0.005817833	0.004515884	-	-	-	-	-	-	-	-	-	-	-	-	-	-	-	-	-	-
chr11	117051786	117052397	*SIDT2*	4	0.001868138	0.013677999	4	0.020525638	0.014354567	4	0.002235749	0.013624614	-	-	-	4	0.002145602	0.013681617	-	-	-	-	-	-
chr16	11680891	11681807	*LITAF*	10	0.000613977	0.003712217	-	-	-	10	0.000834666	0.003642444	-	-	-	10	0.000749294	0.003640843	-	-	-	-	-	-
chr14	24550845	24551212	*NRL*	6	0.00648876	0.010364726	-	-	-	6	0.008644407	0.010228276	-	-	-	6	0.005559595	0.010318547	-	-	-	-	-	-
chr13	113582793	113583686	-	3	0.00620233	-0.022183431	-	-	-	3	0.007210244	-0.022138615	-	-	-	3	0.006094419	-0.02237932	-	-	-	-	-	-
chr11	6440065	6441289	*APBB1*	18	7.95E-06	-0.006968055	-	-	-	18	9.93E-06	-0.006943366	-	-	-	18	1.12E-05	-0.007058649	-	-	-	-	-	-
chr1	230777617	230778096	*COG2*	9	0.001031865	0.006389871	-	-	-	9	0.001215797	0.006365711	-	-	-	9	0.001039434	0.006525704	-	-	-	-	-	-
chr1	151319512	151320381	*RFX5*	10	0.009012305	-0.000957884	-	-	-	10	0.009895361	-0.000948858	-	-	-	10	0.006382023	-0.001021786	-	-	-	-	-	-
chr7	87257356	87258443	*ABCB1*																					

chr16	67464483	67465561	*HSD11B2*	15	0.003411938	-0.000820448	-	-	-	15	0.004451911	-0.000894302	-	-	-	15	0.002982212	-0.000890711	-	-	-	-	-	-
chr3	38691243	38691506	*SCN5A*	6	0.009320811	-0.001038256	6	0.022817223	-0.001739679	6	0.011067847	-0.001063995	-	-	-	6	0.007686373	-0.001210626	-	-	-	-	-	-
chr12	57587701	57588350	*LRP1*																					
chr12	110687347	110687977	-	3	0.009798341	0.007619899	-	-	-	3	0.011312153	0.007500448	-	-	-	3	0.008858922	0.007539139	-	-	-	-	-	-
chr19	42893103	42894930	*CNFN*	10	0.001845255	-0.011439214	-	-	-	-	-	-	-	-	-	-	-	-	-	-	-	-	-	-
chr1	95392125	95393138	*CNN3*																					
chr12	48275709	48276809	*VDR*	8	0.005485878	-0.00646294	-	-	-	8	0.006771394	-0.006357282	-	-	-	8	0.008725437	-0.006167959	-	-	-	-	-	-
chr17	934534	935884	*ABR*	9	3.83E-07	0.028923887	9	4.48E-06	0.030465351	9	1.54E-07	0.029201319	-	-	-	9	1.56E-07	0.029432269	-	-	-	-	-	-
chr5	16737972	16738723	*MYO10*	5	0.006822347	0.013065608	-	-	-	5	0.007988216	0.013022007	-	-	-	5	0.006130234	0.013098731	5	0.012099334	0.023211513	-	-	-
chr6	101846791	101847656	*GRIK2*	13	0.006601991	0.002787613	-	-	-	-	-	-	-	-	-	-	-	-	-	-	-	-	-	-
chr11	3647086	3648013	*TRPC2*	7	0.00256622	-0.00510869	-	-	-	7	0.003029564	-0.004952547	-	-	-	7	0.003892666	-0.004594921	-	-	-	-	-	-
chr16	3013025	3013745	*KREMEN2*	4	0.004936136	0.00534656	-	-	-	4	0.006038827	0.005257776	-	-	-	4	0.004534216	0.005180793	-	-	-	-	-	-
chr11	68637568	68638100	-	3	0.002691042	-0.034597056	-	-	-	3	0.003219171	-0.034526901	-	-	-	3	0.001059435	-0.03612679	-	-	-	-	-	-
chr13	73355442	73356506	*DIS3*																					
chr15	78556178	78557094	*DNAJA4*	12	0.00397773	0.003375181	-	-	-	-	-	-	-	-	-	-	-	-	-	-	-	-	-	-
chr6	30034178	30035234	*PPP1R11*																					
chr11	64405054	64406051	*NRXN2*	8	0.00307053	0.014295221	-	-	-	8	0.003484989	0.014398856	-	-	-	8	0.002432495	0.014593536	-	-	-	-	-	-
chr2	208889973	208890802	*PLEKHM3*	12	0.003574244	-0.008282587	-	-	-	12	0.004284998	-0.008250291	-	-	-	12	0.005254496	-0.008147486	-	-	-	-	-	-
chr12	116756805	116756948	-	3	0.007427494	-0.010970685	-	-	-	3	0.007585726	-0.011038921	-	-	-	3	0.00703777	-0.011154132	-	-	-	-	-	-
chr4	7647940	7649606	*SORCS2*	6	0.001040855	-0.011349352	-	-	-	6	0.001222039	-0.011458176	-	-	-	6	0.000643254	-0.011666353	-	-	-	-	-	-
chr3	187387189	187388737	*SST*	15	1.90E-05	-0.003041652	-	-	-	15	2.68E-05	-0.003067863	-	-	-	15	1.95E-05	-0.002548994	-	-	-	-	-	-
chr16	85539811	85540731	-	3	0.005336297	-0.018908936	-	-	-	3	0.006326689	-0.018824832	-	-	-	3	0.004246352	-0.019210313	-	-	-	-	-	-
chr1	41156520	41157828	*NFYC*																					
chr17	80315917	80316908	*TEX19*	4	0.005897592	-0.006544945	-	-	-	4	0.006224711	-0.006486834	-	-	-	4	0.006954593	-0.006439082	-	-	-	-	-	-
chr16	55866757	55867072	*CES1*	4	0.009779257	-0.050156174	-	-	-	4	0.010658295	-0.050335865	-	-	-	4	0.008260717	-0.051238471	-	-	-	-	-	-
chr6	30644235	30644478	*DHX16*																					
chr1	1981657	1983033	*PRKCZ*	7	0.008227276	0.012026338	-	-	-	-	-	-	-	-	-	7	0.007178472	0.012077575	-	-	-	-	-	-
chr2	218712695	218712871	*TNS1*	4	0.008739714	-0.001321486	-	-	-	4	0.00961493	-0.001437249	-	-	-	4	0.009522231	-0.001464202	-	-	-	-	-	-
chr3	79067596	79069442	*ROBO1*	15	0.000591688	-0.003202526	-	-	-	15	0.000781757	-0.003165503	-	-	-	15	0.001003054	-0.003116229	-	-	-	-	-	-
chr20	62369445	62370310	*LIME1*																					
chr11	14664598	14665355	*PDE3B*																					
chr4	184642625	184642971	-	4	6.52E-05	-0.041666452	4	0.009201103	-0.046213744	4	8.78E-05	-0.041088274	-	-	-	4	9.10E-05	-0.041418786	-	-	-	-	-	-
chr2	121223534	121224327	*LINC01101*	7	0.003019968	0.021352739	-	-	-	7	0.002488737	0.021653357	-	-	-	7	0.003603075	0.021219889	-	-	-	-	-	-
chr15	72978347	72979048	*BBS4*																					
chr19	14016603	14017658	*BRME1*																					
chr1	27989987	27990967	-	3	0.007101192	0.000555753	-	-	-	3	0.008178199	0.000484464	-	-	-	3	0.007757513	0.000523668	-	-	-	-	-	-
chr5	1644520	1645640	-	6	0.003640004	-0.011877996	-	-	-	6	0.003846586	-0.011950858	-	-	-	6	0.0033512	-0.012060268	-	-	-	-	-	-
chr15	89560878	89561135	-	3	0.007466691	-0.009386004	-	-	-	3	0.008450362	-0.009417701	-	-	-	3	0.006710674	-0.009493714	-	-	-	-	-	-
chr7	100423987	100424532	*EPHB4*	3	0.005920898	0.00244131	-	-	-	3	0.006011494	0.002366622	-	-	-	3	0.008390163	0.002401338	-	-	-	-	-	-
chr19	34174345	34175554	*CHST8*	20	0.000478519	-0.003553191	-	-	-	20	0.000628262	-0.003580378	-	-	-	20	0.001042194	-0.003476505	-	-	-	-	-	-
chr20	36887672	36889728	*KIAA1755*	14	0.00033646	0.005565349	-	-	-	-	-	-	-	-	-	-	-	-	-	-	-	-	-	-
chr8	27850178	27850772	*SCARA5*	9	0.000699656	-0.004145203	-	-	-	9	0.000844456	-0.004280821	-	-	-	9	0.000836633	-0.004493849	9	0.002839056	-0.006223713	-	-	-
chr18	77442923	77443784	*CTDP1*	3	0.006970061	-0.024272818	-	-	-	3	0.007980336	-0.024255513	-	-	-	3	0.009021122	-0.02373088	-	-	-	-	-	-
chr13	111317935	111318640	*CARS2*	5	0.00835265	-0.012254162	-	-	-	5	0.010475958	-0.011990969	-	-	-	5	0.008216391	-0.012386378	-	-	-	-	-	-
chr6	44281348	44282149	*AARS2*																					
chr18	57564897	57567904	*PMAIP1*	15	0.001258001	0.010357053	-	-	-	15	0.00121908	0.010615181	-	-	-	15	0.001042195	0.010769551	-	-	-	-	-	-
chr11	45672100	45673410	*CHST1*	9	0.008169198	-0.003497661	-	-	-	9	0.009627413	-0.003642652	9	0.015243259	-0.020712558	9	0.009352774	-0.003395061	-	-	-	-	-	-
chr12	103889042	103890083	*C12orf42*	14	0.003905778	-0.00378965	-	-	-	14	0.004852417	-0.003750218	-	-	-	14	0.004941132	-0.003799973	-	-	-	-	-	-
chr11	134257744	134258597	*B3GAT1*	7	0.000127497	0.013741486	-	-	-	7	0.000170481	0.013711936	-	-	-	7	0.000178185	0.013722237	-	-	-	-	-	-
chr6	3160502	3160634	*TUBB2A*	4	0.008074953	-0.018478361	-	-	-	4	0.009990747	-0.018071791	-	-	-	4	0.010379489	-0.018216038	-	-	-	-	-	-
chr5	138677941	138678409	*PAIP2*	6	0.00122757	0.000815906	-	-	-	6	0.00166724	0.000813815	-	-	-	-	-	-	-	-	-	-	-	-
chr17	78560478	78560916	*RPTOR*	3	0.000410293	-0.025141258	3	0.044370962	-0.024666361	3	0.000510493	-0.024998494	-	-	-	3	0.000441732	-0.025146345	3	0.029771299	-0.032784224	-	-	-
chr16	89181807	89182492	*ACSF3*	7	6.68E-06	-0.008518713	-	-	-	7	8.58E-06	-0.00852183	-	-	-	7	1.06E-05	-0.008493573	-	-	-	-	-	-
chr20	4666423	4666916	*PRNP*	9	0.009552185	0.001647684	-	-	-	9	0.01059227	0.001618152	-	-	-	9	0.007975514	0.001729875	-	-	-	-	-	-
chr1	1027520	1028583	*C1orf159*	6	0.00416514	0.010796592	-	-	-	6	0.00533651	0.010728171	-	-	-	6	0.004329215	0.010685962	-	-	-	-	-	-
chr9	138436220	138437177	*OBP2A*	3	0.0068764	0.007824418	-	-	-	-	-	-	-	-	-	-	-	-	-	-	-	-	-	-
chr5	125514187	125514911	-	3	0.007425751	0.000694155	-	-	-	3	0.00891097	0.000665263	-	-	-	3	0.006924458	0.00027882	-	-	-	-	-	-
chr16	4986962	4987927	*PPL*	12	0.00208175	-0.00696983	-	-	-	12	0.002522301	-0.006915224	-	-	-	12	0.002836404	-0.006893274	-	-	-	-	-	-
chr5	162992841	162993262	-	4	0.001838945	-0.012247702	-	-	-	4	0.002338124	-0.012326434	-	-	-	4	0.001898253	-0.012433125	-	-	-	-	-	-
chr6	32945234	32946524	*BRD2*	9	0.003987352	0.001691898	-	-	-	9	0.004314397	0.00175029	-	-	-	9	0.003603075	0.001877741	-	-	-	-	-	-
chr15	99499605	99499719	*IGF1R*	3	0.005440054	-0.023460965	-	-	-	3	0.005899261	-0.023614577	-	-	-	3	0.007375917	-0.023018718	-	-	-	-	-	-
chr19	3480363	3480940	*SMIM24*	7	1.23E-05	-0.029951742	-	-	-	7	1.69E-05	-0.029769732	-	-	-	7	1.69E-05	-0.029837017	-	-	-	-	-	-
chr3	100053070	100053950	*NIT2*	12	0.00137738	0.002788108	12	0.007829005	0.002821237	12	0.001553273	0.002770228	-	-	-	12	0.001953949	0.002617175	-	-	-	-	-	-
chr19	51774264	51775111	-	7	4.35E-05	0.027961707	-	-	-	7	6.50E-05	0.027622795	-	-	-	7	4.05E-05	0.028278875	7	0.015250674	0.035251133	-	-	-
chr7	101555980	101556938	*CUX1*	8	7.07E-05	-0.012041203	-	-	-	-	-	-	-	-	-	8	9.19E-05	-0.012038734	-	-	-	-	-	-
chr10	15355978	15356822	*FAM171A1*	3	0.004619454	-0.001917502	-	-	-	3	0.005527839	-0.001907183	-	-	-	3	0.00600603	-0.001854036	-	-	-	-	-	-
chr15	60285234	60287537	-	11	0.008336859	0.004164242	-	-	-	11	0.009545534	0.004040294	-	-	-	11	0.008242959	0.00427208	-	-	-	-	-	-
chr20	44419733	44421026	*DNTTIP1*																					
chr14	36989588	36990534	*NKX2-1*																					
chr2	240161815	240162404	*HDAC4*	5	0.003801005	-0.010686518	5	0.010134023	-0.014860372	5	0.004865334	-0.010403775	-	-	-	5	0.00537573	-0.010240356	-	-	-	-	-	-
chr14	74352920	74353709	*ZNF410*	11	0.006692778	0.002317824	-	-	-	11	0.007053725	0.002274432	-	-	-	11	0.007743234	0.002222338	-	-	-	-	-	-
chr6	83072573	83073619	*TPBG*	10	0.005148262	-0.006410955	-	-	-	10	0.006095351	-0.006448505	-	-	-	10	0.006292626	-0.006333753	-	-	-	-	-	-
chr19	3931338	3933856	*NMRK2*	14	0.002254848	-0.001733467	-	-	-	14	0.002623823	-0.001710624	-	-	-	-	-	-	-	-	-	-	-	-
chr2	234215396	234216562	*SAG*	12	0.000256985	-0.001558627	-	-	-	12	0.00030585	-0.001532002	-	-	-	12	0.000244416	-0.001413001	-	-	-	-	-	-
chr5	7348324	7348673	-	3	0.002518769	-0.020372181	-	-	-	3	0.002862392	-0.020270021	-	-	-	3	0.002129855	-0.020793302	-	-	-	-	-	-
chr17	9549496	9550545	*USP43*	8	0.00012745	0.02214183	-	-	-	8	0.000100073	0.022412622	-	-	-	8	8.36E-05	0.022622501	-	-	-	-	-	-
chr5	421317	422114	*AHRR*	3	0.005045934	-0.02354353	-	-	-	3	0.006278801	-0.023186458	-	-	-	3	0.006991097	-0.023075739	-	-	-	-	-	-
chr6	139694265	139694674	*CITED2*	4	0.009370039	-0.002622424	-	-	-	4	0.011352854	-0.002598921	-	-	-	4	0.011040464	-0.002582533	-	-	-	-	-	-
chr12	10324843	10325287	*OLR1*	5	0.009582659	-0.002050179	-	-	-	5	0.011390568	-0.002044637	-	-	-	5	0.011750422	-0.001784558	-	-	-	-	-	-
chr2	219154857	219155790	*PNKD*																					
chr1	56724194	56725058	-	5	0.006574638	0.010331429	-	-	-	5	0.008287238	0.010269977	-	-	-	5	0.007233019	0.01042254	-	-	-	-	-	-
chr10	134844217	134844771	-	5	0.005356794	-0.014377622	-	-	-	5	0.006017241	-0.014326218	-	-	-	5	0.005279354	-0.014480922	-	-	-	-	-	-
chr4	104640560	104641554	*TACR3*	11	0.000837719	-0.00837507	-	-	-	11	0.000999461	-0.008216366	-	-	-	11	0.001298211	-0.008264811	-	-	-	-	-	-
chr19	54023732	54024435	*ZNF331*	13	0.001218854	-0.002301372	-	-	-	13	0.001528473	-0.002383662	-	-	-	13	0.00164862	-0.002282385	-	-	-	-	-	-
chr12	34464089	34464939	-	3	0.005899704	-0.021835354	-	-	-	3	0.006224711	-0.021977632	-	-	-	-	-	-	-	-	-	-	-	-
chr12	131355340	131355834	*RAN*	4	0.001274759	0.013772385	-	-	-	4	0.00162936	0.013553892	-	-	-	4	0.001718267	0.01331464	-	-	-	-	-	-
chr18	45457451	45458243	*SMAD2*	7	0.005530342	-0.011029494	-	-	-	-	-	-	-	-	-	7	0.004481961	-0.011346991	-	-	-	-	-	-
chr10	31288287	31288688	*ZNF438*	3	0.004363662	-0.023644405	-	-	-	3	0.004382457	-0.023815973	-	-	-	3	0.005466398	-0.023561054	-	-	-	-	-	-
chr1	75198211	75199496	*CRYZ*																					
chr16	67927351	67928026	*PSKH1*	3	0.000591276	0.008198654	-	-	-	3	0.000739842	0.008243557	-	-	-	-	-	-	-	-	-	-	-	-
chr8	69243752	69244553	*C8orf34*																					
chr10	130830901	130832393	-	10	1.53E-06	-0.003521773	-	-	-	10	2.35E-06	-0.003548684	-	-	-	10	2.93E-06	-0.003389693	-	-	-	-	-	-
chr9	18473902	18474243	*ADAMTSL1*	5	0.001953826	-0.008448963	-	-	-	5	0.002371764	-0.008357005	-	-	-	5	0.003071671	-0.008156719	-	-	-	-	-	-
chr12	6809127	6810055	*PIANP*	15	0.008880929	-0.00721823	-	-	-	15	0.010010189	-0.007270593	-	-	-	15	0.006823503	-0.007380144	-	-	-	-	-	-
chr6	36644997	36646551	*CDKN1A*	16	0.005031145	0.004985402	-	-	-	16	0.005581969	0.004989122	-	-	-	16	0.00438753	0.004886198	-	-	-	-	-	-
chr2	74668072	74669573	*RTKN*	13	0.005953825	0.017622867	-	-	-	-	-	-	-	-	-	-	-	-	13	0.004240897	0.029786806	-	-	-
chr19	44711560	44711915	*ZNF227*	4	0.009602855	-0.006231823	-	-	-	4	0.011074038	-0.006260931	-	-	-	4	0.010499168	-0.006306668	-	-	-	-	-	-
chr17	80372912	80373608	*HEXD*																					
chr15	90345999	90346923	*ANPEP*	5	1.72E-19	-0.044563113	5	2.19E-11	-0.046582927	5	1.02E-19	-0.044034385	-	-	-	5	3.16E-19	-0.044527608	-	-	-	-	-	-
chr2	179916080	179916205	*CCDC141*	4	0.003858634	0.014296555	-	-	-	4	0.003691688	0.014454206	-	-	-	4	0.003858964	0.014348066	-	-	-	-	-	-
chr4	93225565	93226926	*GRID2*	11	0.000701269	-0.000415779	-	-	-	11	0.000851517	-0.000339808	-	-	-	11	0.000438617	-5.33E-05	-	-	-	-	-	-
chr2	70311984	70313833	*PCBP1*																					
chr22	30818470	30819402	*MTFP1*																					
chr16	87090731	87091652	-	7	0.009076291	0.011991173	-	-	-	7	0.010707802	0.011951628	-	-	-	7	0.011465373	0.011986451	-	-	-	-	-	-
chr12	51403056	51403474	*SLC11A2*	5	0.005729254	-0.006776839	-	-	-	5	0.00705812	-0.006740358	-	-	-	5	0.009127316	-0.006063957	-	-	-	-	-	-
chr21	46492095	46494274	*ADARB1*																					
chr16	81711959	81712102	*CMIP*	3	0.006332359	0.031636215	-	-	-	3	0.006521714	0.031856835	-	-	-	3	0.006550407	0.031758498	-	-	-	-	-	-
chr16	11369940	11370688	*PRM2*																					
chr8	38830814	38831857	*HTRA4*																					
chr2	149894539	149895362	*LYPD6B*	7	0.008103758	0.01183519	-	-	-	7	0.009832242	0.011816072	-	-	-	7	0.009369084	0.011979363	-	-	-	-	-	-
chr2	42968	43812	*FAM110C*	3	0.00512212	-0.009775241	-	-	-	-	-	-	-	-	-	-	-	-	-	-	-	-	-	-

chr10	134818842	134819662	-	6	9.58E-05	-0.01029761	-	-	-	6	0.00011073	-0.010269472	-	-	-	6	8.12E-05	-0.01032728	6	0.035108626	-0.014532565	-	-	-
chr10	134821210	134822001	-	4	0.000466482	0.019628198	-	-	-	4	0.000544518	0.019624693	-	-	-	4	0.000511883	0.019880124	-	-	-	-	-	-
chr7	22765321	22766155	*IL6*	5	0.006020531	0.01161965	-	-	-	5	0.006677603	0.011382639	-	-	-	5	0.006130812	0.011379003	-	-	-	-	-	-
chr16	73099917	73101264	-	6	6.48E-06	0.019478345	-	-	-	6	5.09E-06	0.0197356	-	-	-	6	1.35E-06	0.020140917	-	-	-	-	-	-
chr10	97200985	97201222	*SORBS1*	4	0.004075086	-0.013488124	4	0.030813759	-0.016277945	4	0.005068404	-0.013354952	-	-	-	4	0.005376528	-0.013236003	-	-	-	-	-	-
chr8	143580965	143581481	*ADGRB1*	3	0.006321081	-0.035498697	-	-	-	3	0.007260101	-0.035478137	-	-	-	3	0.006441018	-0.03583505	-	-	-	-	-	-
chr14	77964712	77965757	*ISM2*	8	0.009396011	-0.005212562	-	-	-	-	-	-	-	-	-	8	0.009491527	-0.005315404	-	-	-	-	-	-
chr16	56388908	56390830	*GNAO1*	8	0.004092908	-0.005932087	-	-	-	8	0.004769313	-0.00595139	-	-	-	8	0.004715765	-0.005980328	-	-	-	-	-	-
chr11	2846681	2847778	*KCNQ1*	9	0.000749624	-0.001359011	-	-	-	9	0.000977972	-0.001311965	-	-	-	9	0.001063962	-0.001298759	-	-	-	-	-	-
chr12	14132940	14133887	*GRIN2B*	9	0.007357326	-0.004266192	9	0.026411505	-0.006445579	9	0.009222733	-0.004290895	-	-	-	9	0.009845595	-0.004102894	-	-	-	-	-	-
chr2	119603969	119605441	*EN1*	10	0.002018032	-0.004797143	-	-	-	10	0.002400656	-0.004726221	-	-	-	10	0.002547049	-0.004603814	-	-	-	-	-	-
chr8	142278132	142278585	-	5	0.005760001	0.00532039	-	-	-	5	0.007227929	0.005330408	-	-	-	5	0.006491844	0.005208895	-	-	-	-	-	-
chr10	134790769	134791702	-	10	0.001145806	-0.008015715	-	-	-	10	0.00118181	-0.008106525	10	0.005444476	-0.019238323	10	0.000827537	-0.008441644	-	-	-	-	-	-
chr15	93197833	93199536	*FAM174B*	16	0.005357399	0.000125748	-	-	-	-	-	-	-	-	-	-	-	-	-	-	-	-	-	-
chr1	4193126	4194412	-	5	0.000182821	0.011953529	-	-	-	5	0.000227265	0.011945574	-	-	-	5	0.000224208	0.011746564	-	-	-	-	-	-
chr6	10382800	10384104	-	11	0.001876906	0.002960429	-	-	-	11	0.002138839	0.003019528	-	-	-	11	0.00114876	0.003322982	-	-	-	-	-	-
chr2	206627521	206628773	*NRP2*	12	0.008105759	0.019974143	-	-	-	12	0.007290136	0.020285711	-	-	-	-	-	-	-	-	-	-	-	-
chr7	8481994	8483374	*NXPH1*	9	0.009461984	-0.004216268	-	-	-	9	0.010459682	-0.004138884	-	-	-	9	0.006940042	-0.003831893	-	-	-	-	-	-
chr1	112045871	112047187	*ADORA3*	11	0.000155149	-0.004721138	-	-	-	11	0.000208081	-0.004490622	-	-	-	11	0.000134766	-0.004732047	-	-	-	-	-	-
chr1	76261602	76262984	*MSH4*	11	0.000408907	0.011926125	-	-	-	11	0.000517547	0.011971044	-	-	-	11	0.000534947	0.01214441	-	-	-	-	-	-
chr17	48474379	48475619	*LRRC59*	11	0.005716472	-0.007598907	-	-	-	11	0.006941942	-0.007572202	-	-	-	11	0.004580981	-0.00769297	-	-	-	-	-	-
chr1	45476456	45477041	*HECTD3*																					
chr6	18264287	18265299	*DEK*	10	0.001909447	-0.001202153	-	-	-	10	0.002296838	-0.001157986	-	-	-	-	-	-	-	-	-	-	-	-
chr4	15780238	15781202	*CD38*	7	0.000398091	0.00108881	-	-	-	-	-	-	-	-	-	7	0.000458101	0.00116096	-	-	-	-	-	-
chr19	11093459	11094931	*SMARCA4*	8	0.006677984	-0.001132179	-	-	-	8	0.008028821	-0.001199276	-	-	-	8	0.008247854	-0.001192171	-	-	-	-	-	-
chr14	74226931	74228393	*MIDEAS*	13	1.24E-09	0.006867855	-	-	-	13	2.07E-09	0.006744807	-	-	-	13	2.87E-09	0.006595742	-	-	-	-	-	-
chr2	163695111	163696187	*KCNH7*	9	2.42E-05	-0.012627843	-	-	-	9	2.61E-05	-0.012620208	-	-	-	9	6.58E-05	-0.012135587	-	-	-	-	-	-
chr19	55995052	55996652	*NAT14*																					
chr5	55148279	55148974	*IL31RA*	5	0.0030981	0.006021333	-	-	-	5	0.003629461	0.006088929	-	-	-	5	0.004038786	0.006203492	-	-	-	-	-	-
chr2	167231969	167232696	*SCN9A*	7	0.00299777	-0.003469714	-	-	-	7	0.003670801	-0.003479691	-	-	-	7	0.003251394	-0.003421436	-	-	-	-	-	-
chr22	24819482	24820655	*ADORA2A*																					
chr17	41607535	41608691	*ETV4*	10	0.000202573	-0.008608516	-	-	-	-	-	-	-	-	-	-	-	-	-	-	-	-	-	-
chr10	134900906	134901689	*ADGRA1*	8	0.004219619	-0.010305064	-	-	-	8	0.004473621	-0.010364729	-	-	-	8	0.005684826	-0.010210865	-	-	-	-	-	-
chr5	122758260	122759733	*CEP120*	15	0.000370189	-0.001935496	-	-	-	15	0.000344372	-0.001941342	-	-	-	15	0.000542481	-0.001953688	-	-	-	-	-	-
chr1	2144244	2145655	-	10	1.00E-05	0.012959788	-	-	-	-	-	-	-	-	-	-	-	-	-	-	-	-	-	-
chr1	47691301	47691845	*TAL1*	6	0.00641148	-0.006006681	-	-	-	6	0.007254873	-0.005916199	-	-	-	6	0.00679076	-0.005881435	-	-	-	-	-	-
chr12	52207897	52208899	-	4	8.72E-05	0.024880416	-	-	-	4	0.000113846	0.024658565	-	-	-	4	0.000103378	0.024801431	-	-	-	-	-	-
chr3	147122315	147127662	*ZIC1*																					
chr6	17280551	17283113	*RBM24*	22	0.000687986	0.001714355	-	-	-	22	0.000793117	0.001775766	-	-	-	22	0.000738916	0.001895602	-	-	-	-	-	-
chr2	74728623	74730047	*LBX2*																					
chr4	2469589	2471043	*RNF4*	16	4.78E-05	0.003766886	-	-	-	-	-	-	-	-	-	16	7.91E-05	0.003679902	-	-	-	-	-	-
chr17	46018654	46019184	*PNPO*	14	0.006814354	-0.006886146	14	0.000460971	-0.007766033	14	0.00829152	-0.006907904	-	-	-	14	0.007799465	-0.007040217	-	-	-	-	-	-
chr8	144872060	144872852	-	5	0.004976387	0.008678377	-	-	-	5	0.006197431	0.008613978	5	0.006797969	0.035464777	5	0.005501417	0.008649299	-	-	-	-	-	-
chr17	4900256	4901584	*INCA1*																					
chr14	75535155	75537231	*ACYP1*																					
chr16	30428842	30430044	*ZNF771*	5	0.004033203	0.010461094	-	-	-	5	0.004368889	0.010414502	-	-	-	5	0.003580214	0.010476978	-	-	-	-	-	-
chr20	46286436	46286980	*SULF2*	3	0.009978341	0.003197604	-	-	-	3	0.010058996	0.003246801	-	-	-	3	0.010133677	0.003410283	-	-	-	-	-	-
chr3	112930675	112931496	*BOC*	9	0.00340014	-0.008428866	-	-	-	9	0.004641142	-0.0083713	-	-	-	9	0.005385765	-0.008330142	-	-	-	-	-	-
chr12	57937334	57937454	*DCTN2*	3	0.000453139	-0.020096767	3	1.58E-05	-0.029798302	3	0.000493262	-0.019794991	-	-	-	3	0.000686931	-0.019616077	-	-	-	-	-	-
chr6	83074812	83075187	*TPBG*	3	0.002036346	0.015778156	3	0.003188406	0.025551481	3	0.002715687	0.015725499	-	-	-	3	0.002145602	0.015703374	-	-	-	-	-	-
chr19	44487436	44488612	*ZNF155*	13	3.74E-07	-0.009932575	-	-	-	13	5.51E-07	-0.00987573	-	-	-	13	5.95E-07	-0.009986292	-	-	-	-	-	-
chr5	140580101	140580212	*PCDHB11*	3	0.004683844	-0.026725922	-	-	-	3	0.006002905	-0.026217679	-	-	-	3	0.004908556	-0.026695313	-	-	-	-	-	-
chr12	125633643	125634553	-	6	0.002738604	-0.014035111	-	-	-	6	0.003156541	-0.014081577	-	-	-	-	-	-	-	-	-	-	-	-
chr17	77923971	77925938	*TBC1D16*	15	2.44E-09	0.018436176	-	-	-	15	2.90E-09	0.018407936	-	-	-	15	2.68E-09	0.0185044	-	-	-	-	-	-
chr2	160761085	160762289	*LY75*																					
chr14	38724255	38724945	*CLEC14A*	5	0.001893092	-0.00806063	-	-	-	5	0.002469308	-0.007915729	-	-	-	5	0.002930041	-0.007446126	-	-	-	-	-	-
chr9	99145998	99146400	*SLC35D2*	7	0.004478006	-0.005631715	-	-	-	7	0.005226514	-0.005771787	-	-	-	7	0.006040697	-0.005894689	-	-	-	-	-	-
chr21	47318899	47319633	*PCBP3*	3	0.000221096	0.02060723	-	-	-	3	0.000199099	0.020606086	-	-	-	3	0.000243891	0.020552088	-	-	-	-	-	-
chr22	51065435	51066924	*ARSA*	12	1.15E-09	0.011650886	-	-	-	12	6.17E-10	0.011749403	-	-	-	12	2.74E-09	0.011455557	-	-	-	-	-	-
chr19	12901966	12902806	*JUNB*	8	0.007539824	-0.00344583	-	-	-	8	0.007862641	-0.00343847	-	-	-	8	0.005124119	-0.003643675	-	-	-	-	-	-
chr5	138610977	138611766	*MATR3*																					
chr2	129202959	129203409	-	3	0.007414928	0.009634568	-	-	-	-	-	-	-	-	-	-	-	-	-	-	-	-	-	-
chr5	176307323	176308026	*HK3*																					
chr12	8380001	8380472	*FAM90A1*	5	0.000300152	-0.027899437	-	-	-	5	0.000246278	-0.028257014	-	-	-	5	0.000433675	-0.027520676	-	-	-	-	-	-
chr11	71725245	71725590	*NUMA1*	4	0.008066855	-0.010000034	-	-	-	4	0.010462175	-0.00984707	-	-	-	4	0.010562839	-0.009819094	-	-	-	-	-	-
chr6	30921592	30922043	*MUCL3*	3	0.001549836	-0.008317013	-	-	-	3	0.001737511	-0.008494213	-	-	-	3	0.002133711	-0.008053308	3	0.007233399	-0.01524556	-	-	-
chr20	30795258	30796171	*PLAGL2*																					
chr22	17955450	17956641	*CECR2*	9	0.00035102	-0.013405531	-	-	-	9	0.000433073	-0.013375277	-	-	-	9	0.000495239	-0.013250236	-	-	-	-	-	-
chr8	9762716	9763159	*MIR124-1*																					
chr19	291022	292131	*PLPP2*	10	8.89E-07	-0.015721467	-	-	-	10	1.20E-06	-0.015798645	-	-	-	-	-	-	-	-	-	-	-	-
chr10	130726406	130726701	-	3	0.000287833	-0.052150296	3	0.000352731	-0.064234824	3	0.000336415	-0.052225928	-	-	-	3	0.000138674	-0.053733912	-	-	-	-	-	-
chr16	1160642	1160722	-	3	0.008042856	-0.013944598	-	-	-	3	0.009016338	-0.013961604	3	0.040347577	-0.051598582	3	0.009452649	-0.013786605	-	-	-	-	-	-
chr3	126750108	126751404	*PLXNA1*	4	0.009616411	0.003532147	-	-	-	4	0.010898234	0.003345022	-	-	-	4	0.010527285	0.003407753	-	-	-	-	-	-
chr22	21213387	21214125	*PI4KA*																					
chr8	143858763	143860090	*LYNX1*	18	0.000713901	-0.010485788	-	-	-	-	-	-	-	-	-	18	0.000859419	-0.010714439	-	-	-	-	-	-
chr17	79920132	79921131	*NOTUM*	7	0.003306496	0.015726017	-	-	-	7	0.003866332	0.015724396	-	-	-	7	0.003623855	0.015825609	-	-	-	-	-	-
chr8	22132434	22133356	*PIWIL2*	14	0.00345146	0.009903607	-	-	-	-	-	-	-	-	-	14	0.002897577	0.009985034	-	-	-	-	-	-
chr8	144654887	144655679	*MROH6*	12	0.001918556	-0.007948344	-	-	-	12	0.00214034	-0.008029123	-	-	-	12	0.001432737	-0.008220935	12	0.000331059	-0.014309554	-	-	-
chr5	55007918	55008611	*SLC38A9*	15	0.000212093	-0.00139176	-	-	-	15	0.000263079	-0.001425779	-	-	-	15	0.000247968	-0.001379367	-	-	-	-	-	-
chr15	52861545	52862406	*ARPP19*	8	0.007082289	0.004046584	-	-	-	8	0.008523939	0.004093466	-	-	-	8	0.008669612	0.004063308	-	-	-	-	-	-
chr16	69166316	69168101	*CHTF8*																					
chr10	44806558	44807790	-	4	0.001673176	-0.000932264	-	-	-	4	0.00200056	-0.000900097	-	-	-	4	0.001533056	-0.000845299	-	-	-	-	-	-
chr22	19701108	19702664	*SEPT5-GP1BB*	14	0.00263954	-0.005679508	-	-	-	14	0.003813244	-0.005662579	-	-	-	14	0.002945521	-0.005800232	-	-	-	-	-	-
chr1	165414189	165415170	*RXRG*	15	0.003411938	-0.004351462	-	-	-	15	0.004269411	-0.004330225	-	-	-	15	0.004884178	-0.00432667	-	-	-	-	-	-
chr4	52708582	52709895	*DCUN1D4*	12	0.005579823	-0.004586596	-	-	-	12	0.006840404	-0.004596792	-	-	-	12	0.004140257	-0.004678944	-	-	-	-	-	-
chr11	32355324	32355899	-	4	0.009787958	0.017415288	-	-	-	4	0.011530898	0.017383271	-	-	-	4	0.012969433	0.017221006	-	-	-	-	-	-
chr4	47032325	47034181	*GABRB1*	15	0.001402297	-0.001004205	-	-	-	15	0.001556746	-0.001191132	-	-	-	15	0.001675601	-0.001120952	-	-	-	-	-	-
chr15	86315115	86315211	*KLHL25*																					
chr8	85097031	85097429	*RALYL*	6	0.008713325	-0.008523606	-	-	-	6	0.010502152	-0.008534843	-	-	-	6	0.010649378	-0.008080147	6	0.007013568	-0.021269628	-	-	-
chr2	220264753	220265305	*DNPEP*	4	0.008496258	-0.009475871	-	-	-	-	-	-	-	-	-	-	-	-	-	-	-	-	-	-
chr6	32846924	32847845	-	15	3.84E-05	0.021416406	-	-	-	15	5.53E-05	0.021357902	-	-	-	15	1.61E-05	0.022067167	-	-	-	-	-	-
chr6	27804866	27806842	*H2AC15*																					
chr1	206223701	206224554	*AVPR1B*	7	2.80E-05	0.002330489	-	-	-	7	3.94E-05	0.002276777	-	-	-	7	4.31E-05	0.002184844	-	-	-	-	-	-
chr6	28583655	28584464	-	14	0.002697312	0.011741129	-	-	-	14	0.003582278	0.011196661	-	-	-	14	0.001898748	0.011833224	-	-	-	-	-	-
chr10	116696671	116697985	*TRUB1*	10	0.002598634	-0.004317192	-	-	-	-	-	-	-	-	-	10	0.001982723	-0.004363818	-	-	-	-	-	-
chr10	14995476	14996626	*DCLRE1C*																					
chr1	244093755	244096161	*LINC02774*	7	6.83E-05	0.021756937	-	-	-	-	-	-	-	-	-	7	1.83E-05	0.022321398	-	-	-	-	-	-
chr2	158732186	158733332	*ACVR1*	5	0.003797027	-0.005226633	-	-	-	5	0.003327622	-0.005305158	-	-	-	5	0.00369432	-0.005350941	-	-	-	-	-	-
chr19	4277333	4278055	*SHD*	4	0.007869259	0.00512388	-	-	-	-	-	-	-	-	-	-	-	-	-	-	-	-	-	-
chr5	149996672	149997153	*SYNPO*	4	0.004958271	-0.012088356	-	-	-	-	-	-	-	-	-	-	-	-	-	-	-	-	-	-
chr2	102802929	102804405	*IL1RL2*	11	0.008871277	0.003064161	-	-	-	11	0.009665267	0.002747105	-	-	-	11	0.008558787	0.003185469	-	-	-	-	-	-
chr13	41706227	41708153	*KBTBD6*	11	0.000842278	0.004473947	-	-	-	11	0.001162764	0.004420579	-	-	-	11	0.000632851	0.004502405	-	-	-	-	-	-
chr19	38713050	38713373	*DPF1*	4	0.009654411	-0.018232213	-	-	-	4	0.01075779	-0.018315976	-	-	-	4	0.009494168	-0.018134058	-	-	-	-	-	-
chr12	52827847	52828840	*KRT75*	8	0.00707535	0.001633142	-	-	-	8	0.008750777	0.00147772	-	-	-	8	0.007494011	0.001593356	-	-	-	-	-	-
chr20	57582292	57583709	*CTSZ*	23	0.000116893	-0.003431385	-	-	-	23	0.000139423	-0.003471171	-	-	-	23	0.000115684	-0.003556394	-	-	-	-	-	-
chr17	58225949	58227745	*CA4*	11	0.006458652	0.000246978	-	-	-	11	0.00646687	0.000318292	-	-	-	11	0.008029779	0.000226248	-	-	-	-	-	-
chr12	113795897	113796846	*PLBD2*	9	0.005333839	0.004712014	-	-	-	9	0.005356433	0.004671851	-	-	-	9	0.005115104	0.004605946	-	-	-	-	-	-
chr17	79503292	79504745	*FSCN2*	7	0.000327711	-0.01820661	-	-	-	7	0.000344307	-0.018228092	-	-	-	7	0.000297983	-0.018443932	-	-	-	-	-	-
chr2	14772312	14773274	*LRATD1*	10	0.0030768	-0.006087974	-	-	-	10	0.003816258	-0.006130638	-	-	-	10	0.005355471	-0.005689168	-	-	-	-	-	-
chr17	45918221	45919419	*SCRN2*	11	0.00832315	0.001041195	-	-	-	-	-	-	-	-	-	11	0.006186925	0.001038826	-	-	-	-	-	-
chr3	113160071	113161177	*CFAP44*	14	6.00E-07	0.02060467	-	-	-	14	3.65E-07	0.020863309	-	-	-	14	7.67E-07	0.020652491	-	-	-	-	-	-

chr17	25798180	25799447	*KSR1*	11	0.000436561	0.009225826	-	-	-	11	0.000468824	0.00922198	-	-	-	11	0.000650904	0.008927424	-	-	-	-	-	-
chr14	106320669	106322986	-	16	0.000360271	0.008944743	-	-	-	-	-	-	-	-	-	16	0.000393829	0.008961711	-	-	-	-	-	-
chr15	91499369	91500193	*RCCD1*	6	0.006701219	0.019167047	-	-	-	6	0.00803582	0.019077411	-	-	-	6	0.006496935	0.019224918	-	-	-	-	-	-
chr4	57181855	57182417	*CRACD*	3	0.006019733	0.01387808	-	-	-	3	0.007135418	0.013814161	-	-	-	3	0.00638803	0.013646004	-	-	-	-	-	-
chr10	3507462	3508379	-	3	0.009210031	-0.004129602	-	-	-	3	0.010849567	-0.003899545	-	-	-	3	0.011555463	-0.004049272	-	-	-	-	-	-
chr8	53477881	53479270	*ALKAL1*	10	0.000157831	-0.004378815	-	-	-	10	0.000164136	-0.004408266	-	-	-	10	0.000183856	-0.00410911	-	-	-	-	-	-
chr6	166756218	166756786	*LOC100289495*																					
chr11	116371188	116371487	-	5	0.002693471	-0.026852001	-	-	-	5	0.002962788	-0.027013432	-	-	-	5	0.003695106	-0.026602188	-	-	-	-	-	-
chr18	60051870	60052464	*TNFRSF11A*	5	0.000395505	0.029627717	5	0.021548744	0.0335078	5	0.000169646	0.030359198	5	0.000541769	0.111090818	5	0.00040892	0.029679005	-	-	-	-	-	-
chr14	92587796	92588818	*CPSF2*																					
chr3	120003292	120003884	-	6	0.002850751	0.020880982	-	-	-	6	0.003552741	0.020923815	-	-	-	6	0.003509315	0.020795999	-	-	-	-	-	-
chr6	136172226	136173085	*PDE7B*	7	0.00297616	-0.010664952	-	-	-	-	-	-	-	-	-	7	0.004122635	-0.010593518	-	-	-	-	-	-
chr5	151150029	151151000	*G3BP1*																					
chr2	47382287	47383720	*STPG4*	14	0.003929803	-0.005764151	-	-	-	14	0.004601518	-0.005781518	-	-	-	14	0.006183	-0.005512341	-	-	-	-	-	-
chr3	52001778	52002969	*ABHD14B*																					
chr15	41575421	41576837	*OIP5-AS1*	14	0.009048268	0.00169477	-	-	-	14	0.009888271	0.001659139	-	-	-	14	0.00772649	0.001614745	-	-	-	-	-	-
chr12	52262736	52263387	-	3	0.003524336	0.000532969	-	-	-	3	0.003741029	0.000574359	-	-	-	3	0.004077254	0.000514683	-	-	-	-	-	-
chr3	51976427	51977385	*PARP3*																					
chr12	132939165	132940939	-	7	0.004816602	-0.00023484	-	-	-	7	0.005082281	-0.000252782	-	-	-	7	0.005614089	0.000122692	-	-	-	-	-	-
chr7	47092529	47093015	-	4	0.000729316	0.019524516	-	-	-	4	0.000698971	0.019681586	-	-	-	4	0.000890143	0.01907867	-	-	-	-	-	-
chr3	49044317	49045655	*P4HTM*																					
chr16	15950840	15951230	*MYH11*	6	0.001819587	-0.011937474	-	-	-	6	0.002258315	-0.011894774	-	-	-	6	0.002692151	-0.011707472	-	-	-	-	-	-
chr5	102089184	102090704	-	8	0.004857449	0.006791579	-	-	-	8	0.005193855	0.006871956	-	-	-	8	0.006301178	0.00694542	-	-	-	-	-	-
chr1	48463105	48463807	*TRABD2B*	5	0.00407137	-0.018515333	-	-	-	5	0.005055421	-0.018519745	-	-	-	5	0.003343202	-0.01863853	-	-	-	-	-	-
chr5	149569501	149570189	*SLC6A7*	5	0.003202901	0.002576328	-	-	-	5	0.003407894	0.002720837	-	-	-	5	0.002502697	0.002841029	-	-	-	-	-	-
chr6	42927940	42928773	*GNMT*	25	0.00643739	-0.007355975	-	-	-	25	0.007623705	-0.007376019	-	-	-	25	0.01020677	-0.007258006	25	0.008716971	-0.010466806	-	-	-
chr21	44072610	44073555	*PDE9A*	5	0.006664863	0.008236089	-	-	-	-	-	-	-	-	-	-	-	-	-	-	-	-	-	-
chr8	6795989	6796618	*DEFA4*	6	0.006190687	-0.010714735	6	0.008947838	-0.017047256	6	0.005914976	-0.010870491	-	-	-	6	0.005832773	-0.010919749	-	-	-	-	-	-
chr12	48151974	48153388	*RAPGEF3*	20	0.004243073	-0.001937068	-	-	-	20	0.004819517	-0.001984114	-	-	-	20	0.003018488	-0.001838342	-	-	-	-	-	-
chr11	33758121	33759043	*CD59*	8	0.009899078	-0.006745771	-	-	-	8	0.012701578	-0.006678068	-	-	-	8	0.015024476	-0.006680666	-	-	-	-	-	-
chr20	39799805	39800368	*PLCG1*	3	0.003515432	-0.003775792	-	-	-	3	0.003909183	-0.003832281	-	-	-	3	0.003163084	-0.003720866	-	-	-	-	-	-
chr4	75230391	75231177	*EREG*	9	0.002385305	-0.008080507	-	-	-	9	0.002950436	-0.008059304	-	-	-	9	0.003388668	-0.007993026	-	-	-	-	-	-
chr12	58011837	58013942	*SLC26A10*	25	0.001143213	0.007266215	-	-	-	25	0.001392644	0.007249314	-	-	-	25	0.000983438	0.007330717	-	-	-	-	-	-
chr11	32125186	32125738	*RCN1*	3	0.00295559	-0.016168783	-	-	-	3	0.00330159	-0.016116515	-	-	-	3	0.002755781	-0.016311249	-	-	-	-	-	-
chr1	173174650	173176090	*TNFSF4*	8	1.29E-05	-0.005139747	-	-	-	8	1.78E-05	-0.00511853	-	-	-	8	2.71E-05	-0.004741996	-	-	-	-	-	-
chr7	19183280	19184555	*FERD3L*	8	0.000564726	-0.005600899	-	-	-	-	-	-	-	-	-	-	-	-	-	-	-	-	-	-
chr19	17531239	17531746	*MVB12A*	3	0.008426388	0.006115413	-	-	-	3	0.009691143	0.006037767	-	-	-	3	0.008294291	0.006300797	-	-	-	-	-	-
chr6	70990187	70991229	*COL9A1*	7	0.005195268	0.021173607	-	-	-	7	0.006384901	0.021096281	-	-	-	7	0.00654454	0.020596597	-	-	-	-	-	-
chr4	25865178	25866396	*SEL1L3*	9	8.64E-07	0.008681116	-	-	-	9	1.14E-06	0.008694249	-	-	-	9	3.59E-07	0.008855476	-	-	-	-	-	-
chr5	87962828	87963592	*LINC00461*																					
chr6	131571435	131571669	*AKAP7*	4	0.00371195	-0.014789992	-	-	-	4	0.004347499	-0.014445776	-	-	-	4	0.005999135	-0.013922942	-	-	-	-	-	-
chr16	85110539	85111583	*KIAA0513*	3	0.006194918	0.019103483	-	-	-	3	0.006088524	0.019308057	-	-	-	3	0.008042518	0.018831846	-	-	-	-	-	-
chr17	73641675	73642080	*RECQL5*																					
chr20	17950131	17950965	*MGME1*																					
chr4	171011099	171011760	*AADAT*	15	0.006752832	-0.002457716	-	-	-	15	0.006881783	-0.002412792	-	-	-	15	0.00481766	-0.002417292	-	-	-	-	-	-
chr1	167486978	167488503	*CD247*	8	6.17E-05	-0.01832827	-	-	-	8	7.08E-05	-0.018346292	-	-	-	8	8.79E-05	-0.018313759	-	-	-	-	-	-
chr17	80188598	80190154	*SLC16A3*	14	3.60E-08	0.007425859	-	-	-	14	6.39E-08	0.007312669	-	-	-	14	1.03E-08	0.0076234	-	-	-	-	-	-
chr16	1143566	1144264	*C1QTNF8*	4	0.000436561	-0.025922075	-	-	-	4	0.000436758	-0.026095362	-	-	-	4	0.000557224	-0.025722321	-	-	-	-	-	-
chr4	40631741	40633572	*RBM47*	14	0.000608679	-0.00453237	-	-	-	14	0.000787335	-0.004507845	-	-	-	-	-	-	-	-	-	-	-	-
chr2	51259175	51259703	*NRXN1*	10	0.004713847	-0.009838068	-	-	-	10	0.006369435	-0.009810226	-	-	-	10	0.005723377	-0.009875636	-	-	-	-	-	-
chr10	125732373	125732842	-	4	0.00599939	-0.013052595	-	-	-	4	0.006193634	-0.013107347	-	-	-	4	0.007877496	-0.012667818	-	-	-	-	-	-
chr7	1068125	1068617	*C7orf50*	3	9.09E-05	0.011852922	-	-	-	3	0.000118198	0.011880979	3	0.00100953	0.047746106	3	0.000131134	0.011664073	-	-	-	-	-	-
chr19	12776725	12777903	*MAN2B1*																					
chr16	86852700	86853235	-	3	0.000111949	0.025360569	-	-	-	3	0.000138131	0.02520958	-	-	-	3	6.64E-05	0.02573299	-	-	-	-	-	-
chr16	3311857	3313282	*LINC00921*																					
chr10	126826304	126827067	*CTBP2*	5	9.49E-05	0.012475545	-	-	-	5	0.000116632	0.012458009	-	-	-	5	0.000121211	0.012419338	-	-	-	-	-	-
chr17	37719431	37720009	-	3	0.004652014	-0.014576074	-	-	-	3	0.005146746	-0.014328264	-	-	-	3	0.003624742	-0.013198533	3	0.000662431	-0.038143115	-	-	-
chr18	24219872	24220737	*KCTD1*	3	0.002034958	0.014897312	-	-	-	-	-	-	-	-	-	-	-	-	-	-	-	-	-	-
chr4	101111617	101112395	*DDIT4L*	9	0.005633036	-0.006140492	-	-	-	9	0.007174339	-0.006131759	-	-	-	9	0.007803652	-0.006128611	-	-	-	-	-	-
chr11	61247223	61248887	*PPP1R32*	9	0.003795138	0.006840728	-	-	-	-	-	-	-	-	-	9	0.00419293	0.006776037	-	-	-	-	-	-
chr16	50347766	50347895	*ADCY7*	3	0.003392847	-0.017567118	-	-	-	3	0.003647581	-0.017648572	-	-	-	3	0.004150472	-0.017442573	-	-	-	-	-	-
chr14	104345945	104347395	-	8	0.001705993	-0.009428986	-	-	-	8	0.002129135	-0.009433609	-	-	-	8	0.002862994	-0.009288375	-	-	-	-	-	-
chr7	11871535	11872745	*THSD7A*	10	0.000221437	-0.011878523	-	-	-	-	-	-	-	-	-	-	-	-	-	-	-	-	-	-
chr3	196755973	196756877	*MELTF*	6	0.00699477	-0.008571144	-	-	-	6	0.008328405	-0.008556758	-	-	-	6	0.00759819	-0.008762961	-	-	-	-	-	-
chr6	29691408	29692995	*HLA-F*	24	5.73E-06	0.0101074	-	-	-	24	8.58E-06	0.01005951	-	-	-	24	1.18E-05	0.009802558	-	-	-	-	-	-
chr1	207818003	207819196	*CR1L*	9	0.009219402	-0.000920038	-	-	-	9	0.009799048	-0.001050335	-	-	-	9	0.009147133	-0.000841987	-	-	-	-	-	-
chr12	124414964	124415398	*DNAH10*	3	0.008337979	0.017123823	-	-	-	3	0.009507504	0.01712923	-	-	-	3	0.007736278	0.017392554	-	-	-	-	-	-
chr6	31238388	31239411	*HLA-B*																					
chr18	61088726	61090281	*VPS4B*	14	0.00038615	0.002759326	-	-	-	14	0.000488819	0.002742981	-	-	-	14	0.000540356	0.002607291	14	0.003725888	0.005461133	-	-	-
chr10	112888790	112889349	-	3	0.005045934	-0.022654064	-	-	-	3	0.006049658	-0.022576923	-	-	-	3	0.006019819	-0.022496537	-	-	-	-	-	-
chr17	73823769	73825227	*UNC13D*	7	1.22E-20	-0.039423523	7	8.83E-10	-0.036400501	7	1.33E-20	-0.039518354	-	-	-	7	1.39E-20	-0.039609672	7	4.35E-13	-0.051844308	-	-	-
chr20	33879989	33881762	*FAM83C*	12	0.005403129	0.010473476	-	-	-	12	0.006213515	0.010469869	-	-	-	12	0.007904491	0.010315415	-	-	-	-	-	-
chr12	65218019	65218728	*TBC1D30*	12	0.004622217	-0.001952676	-	-	-	12	0.005820262	-0.001944756	-	-	-	12	0.004738584	-0.001895667	-	-	-	-	-	-
chr5	33440240	33441304	*TARS1*	13	0.008128017	-0.001097088	-	-	-	13	0.010450736	-0.001139552	-	-	-	13	0.008868605	-0.001088227	-	-	-	-	-	-
chr6	35490164	35490818	-	4	0.004308713	0.004141755	-	-	-	4	0.004352945	0.004270451	-	-	-	4	0.004741242	0.004163056	-	-	-	-	-	-
chr16	1034059	1034527	*LMF1*																					
chr19	54693755	54694791	*MBOAT7*																					
chr17	7906521	7908103	*GUCY2D*	6	0.00271819	0.014252683	-	-	-	6	0.003634467	0.014122359	-	-	-	6	0.003081171	0.014349564	-	-	-	-	-	-
chr5	160975347	160976417	*GABRB2*	12	0.004763258	-0.006504189	-	-	-	12	0.006296488	-0.006484671	-	-	-	-	-	-	-	-	-	-	-	-
chr22	37641093	37641970	*RAC2*	4	0.004427254	-0.012600899	-	-	-	4	0.005167441	-0.012564253	-	-	-	4	0.005729786	-0.012441042	-	-	-	-	-	-
chr7	1908250	1908745	*MAD1L1*	5	0.005857899	0.006956925	-	-	-	5	0.007045623	0.006902738	-	-	-	5	0.007823422	0.007089456	-	-	-	-	-	-
chr5	1033518	1034307	*NKD2*	5	0.00581707	-0.000924262	-	-	-	5	0.007080931	-0.00097638	-	-	-	5	0.005975592	-0.001202581	-	-	-	-	-	-
chr17	80407031	80407779	*CYBC1*	8	0.000949888	-0.00917125	-	-	-	8	0.001231032	-0.009130272	-	-	-	8	0.000509336	-0.009557139	-	-	-	-	-	-
chr2	10183603	10184650	*KLF11*	11	0.004687405	0.014267179	-	-	-	-	-	-	-	-	-	11	0.001885321	0.014460089	-	-	-	-	-	-
chr2	5831370	5834638	*SOX11*	13	2.87E-05	-0.006123935	-	-	-	13	3.57E-05	-0.006044125	-	-	-	-	-	-	-	-	-	-	-	-
chr12	62860029	62861114	*MON2*	13	0.001631822	0.001269795	-	-	-	13	0.001905308	0.00125975	-	-	-	13	0.001868899	0.001260054	-	-	-	-	-	-
chr19	7894388	7895393	*EVI5L*	11	0.003465527	-0.013088741	-	-	-	-	-	-	-	-	-	11	0.00283964	-0.013307108	-	-	-	-	-	-
chr5	373378	374252	*AHRR*	4	5.36E-21	0.045739382	-	-	-	4	1.07E-20	0.045705335	-	-	-	4	1.57E-21	0.045942422	-	-	-	-	-	-
chr4	77723057	77723800	-	6	9.61E-06	0.02407009	-	-	-	6	6.87E-06	0.02434332	-	-	-	6	6.49E-06	0.024436599	-	-	-	-	-	-
chr5	92909429	92910052	*NR2F1-AS1*	4	0.009636473	-0.010855904	-	-	-	4	0.011783337	-0.010808106	-	-	-	4	0.011613792	-0.010820668	-	-	-	-	-	-
chr1	203320297	203321087	*FMOD*	10	0.008191524	-0.020739448	-	-	-	10	0.007631244	-0.02082526	-	-	-	10	0.007771405	-0.0207959	-	-	-	-	-	-
chr2	71017541	71018823	*FIGLA*	12	0.003664775	0.011873534	-	-	-	12	0.004272152	0.011828199	-	-	-	12	0.001826137	0.012281744	-	-	-	-	-	-
chr17	56494554	56495283	*RNF43*	5	0.001517257	0.002320314	-	-	-	5	0.001736925	0.002381542	-	-	-	5	0.001079361	0.002436132	-	-	-	-	-	-
chr20	43180850	43181661	*PKIG*	3	0.00208993	0.025166719	-	-	-	3	0.001809033	0.025345986	-	-	-	3	0.001503174	0.02549769	-	-	-	-	-	-
chr3	128721448	128722579	*EFCC1*	5	0.004379081	0.009246167	-	-	-	5	0.004579866	0.009094931	-	-	-	5	0.004785954	0.009085555	-	-	-	-	-	-
chr11	69456412	69457121	*CCND1*	5	0.007938659	-0.003999349	-	-	-	5	0.009827873	-0.003986546	-	-	-	5	0.008374637	-0.004080444	-	-	-	-	-	-
chr20	42983727	42984878	*HNF4A*	13	0.003405482	0.007494531	-	-	-	13	0.0039238	0.007424166	-	-	-	13	0.003962766	0.007414361	-	-	-	-	-	-
chr7	27224700	27226329	*HOXA10*																					
chr10	125754300	125755854	-	7	1.54E-07	-0.019689501	-	-	-	7	1.96E-07	-0.019664016	-	-	-	7	1.13E-07	-0.01973704	-	-	-	-	-	-
chr10	135341443	135343248	*CYP2E1*	12	0.0031751	-0.035726511	-	-	-	12	0.003275784	-0.035777447	-	-	-	-	-	-	-	-	-	-	-	-
chr11	117703496	117704267	*FXYD2*																					
chr7	5426831	5427251	*TNRC18*	3	0.004722932	0.006349246	-	-	-	3	0.005730024	0.00635072	-	-	-	3	0.00581693	0.00630748	-	-	-	-	-	-
chr11	126172682	126174769	*DCPS*	22	2.53E-08	0.011486857	-	-	-	22	3.89E-08	0.011404496	-	-	-	22	7.67E-08	0.010872617	-	-	-	-	-	-
chr5	110427072	110427275	*WDR36*	3	0.005159146	-0.008418069	-	-	-	3	0.006072976	-0.00839308	3	0.02433279	-0.024453468	3	0.004770024	-0.008282435	-	-	-	-	-	-
chr16	31020735	31022521	*STX1B*	11	0.000390745	-0.011270121	-	-	-	11	0.000478501	-0.011300121	-	-	-	11	0.000477001	-0.011398858	-	-	-	-	-	-
chr21	40984285	40985406	*B3GALT5*																					
chr2	46842958	46843974	*CRIPT*																					
chr16	57728173	57729107	*DRC7*	7	0.000180936	-0.005604143	-	-	-	7	0.000218828	-0.005649833	-	-	-	7	0.000328007	-0.005526629	-	-	-	-	-	-
chr10	3145966	3146549	*PFKP*	6	0.007606421	0.010688058	-	-	-	-	-	-	-	-	-	6	0.006817308	0.010853665	-	-	-	-	-	-
chr22	41346948	41348222	*RBX1*																					
chr3	195477854	195478715	*MUC4*	5	0.009240171	0.013901563	-	-	-	5	0.011269613	0.013809011	-	-	-	5	0.010312775	0.013897661	-	-	-	-	-	-

chr8	26370107	26371804	*DPYSL2*																					
chr10	103879827	103880419	*LDB1*	9	0.006577668	-0.004184326	-	-	-	-	-	-	-	-	-	9	0.007504421	-0.004265052	-	-	-	-	-	-
chr17	8868480	8869105	*PIK3R5*	7	0.001213949	0.002316089	-	-	-	7	0.001541076	0.002262852	-	-	-	-	-	-	-	-	-	-	-	-
chr1	45805261	45807214	*MUTYH*																					
chr5	59188112	59188920	*PDE4D*	3	0.000556229	-0.011957144	-	-	-	3	0.000625707	-0.01188595	-	-	-	3	0.000895402	-0.01140543	-	-	-	-	-	-
chr5	133450709	133452188	*TCF7*	11	0.007521281	0.002472368	-	-	-	11	0.009350914	0.002524386	-	-	-	-	-	-	-	-	-	-	-	-
chr10	88728947	88729861	*ADIRF*																					
chr11	64267330	64268315	-	4	0.004834421	-0.012844087	-	-	-	4	0.005948855	-0.012598659	-	-	-	4	0.005780422	-0.012784147	-	-	-	-	-	-
chr5	138289138	138289233	*SIL1*	3	0.008430498	-0.005882973	-	-	-	3	0.00948951	-0.005801466	-	-	-	3	0.011420528	-0.005646089	-	-	-	-	-	-
chr19	52407881	52408518	*ZNF649*	10	0.005027318	-0.006585875	-	-	-	10	0.006717718	-0.006531958	-	-	-	10	0.006816308	-0.006499181	10	0.008493602	-0.011725379	-	-	-
chr19	19416751	19417480	*SUGP1*	5	0.003233359	0.008544177	-	-	-	5	0.003979276	0.008504819	-	-	-	5	0.003812419	0.008650849	-	-	-	-	-	-
chr10	134597884	134601490	*NKX6-2*	36	0.000128749	-0.009496988	-	-	-	36	0.000153604	-0.009453913	-	-	-	36	0.000214493	-0.009060311	-	-	-	-	-	-
chr7	122527351	122527522	*CADPS2*	4	0.006970535	-0.006183421	-	-	-	4	0.008512356	-0.006171566	-	-	-	4	0.009095089	-0.006117294	-	-	-	-	-	-
chr4	8588716	8589313	*CPZ*																					
chr11	32416984	32417943	*WT1*	8	0.000694981	-0.013947304	-	-	-	-	-	-	-	-	-	8	0.000183096	-0.014855612	-	-	-	-	-	-
chr3	126242379	126243401	*CHST13*	13	0.007480481	0.001214413	-	-	-	13	0.008145191	0.001338223	-	-	-	13	0.008260717	0.001475582	-	-	-	-	-	-
chr5	140718283	140719142	*PCDHGA1*																					
chr11	119018927	119019845	*ABCG4*	6	0.004164163	0.012449051	-	-	-	6	0.00492625	0.012557018	-	-	-	6	0.005242119	0.012368726	-	-	-	-	-	-
chr13	111226725	111228197	-	7	2.41E-05	0.006032595	-	-	-	7	2.87E-05	0.006116413	-	-	-	7	2.88E-05	0.00579627	-	-	-	-	-	-
chr2	5865442	5866470	-	6	0.000374238	-0.006030566	-	-	-	6	0.000504266	-0.00599191	-	-	-	6	0.00034913	-0.006172453	-	-	-	-	-	-
chr6	32036530	32038155	*TNXB*	27	0.006591555	0.005356237	-	-	-	-	-	-	-	-	-	-	-	-	-	-	-	-	-	-
chr19	58326009	58326618	*ZNF552*																					
chr2	198364282	198365671	*HSPD1*																					
chr10	8373416	8374526	-	7	1.40E-05	-0.015724415	-	-	-	7	1.69E-05	-0.015734531	-	-	-	7	1.96E-05	-0.015746706	-	-	-	-	-	-
chr12	114843188	114845223	*TBX5*																					
chr5	1122753	1123154	-	5	0.001436365	0.008719538	-	-	-	5	0.00186847	0.008674796	-	-	-	5	0.001017159	0.008924176	-	-	-	-	-	-
chr14	20903410	20904320	*KLHL33*	12	0.004721942	0.009740032	-	-	-	12	0.005875213	0.009731471	-	-	-	12	0.005819622	0.009671704	-	-	-	-	-	-
chr11	119055760	119056215	*PDZD3*	3	0.006688625	0.005213119	-	-	-	3	0.008023744	0.005318791	-	-	-	3	0.007943503	0.005383376	-	-	-	-	-	-
chr7	99195655	99196468	*TMEM225B*	5	0.000279382	0.027025695	-	-	-	5	0.000368852	0.026885459	-	-	-	5	0.000265305	0.027262797	-	-	-	-	-	-
chr10	98588227	98588605	*LCOR*	4	0.005909442	0.006846575	-	-	-	4	0.006454547	0.006716127	-	-	-	4	0.004256035	0.007010147	-	-	-	-	-	-
chr5	140561239	140562562	*PCDHB16*	9	0.000513358	-0.019206295	-	-	-	9	0.00073635	-0.018710783	-	-	-	9	0.000839209	-0.018597664	-	-	-	-	-	-
chr17	45898841	45899862	*OSBPL7*	5	0.00163778	-0.012393574	-	-	-	5	0.001905308	-0.012427658	-	-	-	5	0.001829035	-0.012528873	-	-	-	-	-	-
chr14	62331131	62331920	-	6	0.002170488	0.021727985	-	-	-	6	0.002439415	0.021837818	-	-	-	6	0.002088615	0.022053701	6	0.001897242	0.032367468	-	-	-
chr9	130660399	130661175	*ST6GALNAC6*	6	3.01E-08	0.019426583	6	1.91E-05	0.02001605	6	2.60E-08	0.019195429	-	-	-	6	3.41E-08	0.01921384	6	0.00045978	0.018140373	-	-	-
chr19	3960982	3961765	*DAPK3*																					
chr17	1268443	1269083	*YWHAE*	4	0.006837216	-0.008238117	-	-	-	4	0.006615823	-0.008258088	-	-	-	4	0.006441018	-0.008259235	-	-	-	-	-	-
chr19	821008	821565	*PLPPR3*	3	0.001735153	-0.001341386	-	-	-	3	0.002087672	-0.001331182	-	-	-	3	0.00227917	-0.001200611	-	-	-	-	-	-
chr2	3583570	3583963	-	3	0.001739438	0.028889713	-	-	-	3	0.002176822	0.028716495	-	-	-	3	0.002747154	0.027786386	3	0.013090666	0.046505694	-	-	-
chr2	109236744	109237735	*LIMS1*	5	0.001150743	-0.01035518	-	-	-	-	-	-	-	-	-	5	0.001450631	-0.010364978	-	-	-	-	-	-
chr2	97651765	97652806	*FAM178B*	9	0.006529285	0.007291823	-	-	-	9	0.007752397	0.007227146	-	-	-	9	0.007674744	0.007492459	-	-	-	-	-	-
chr14	29227941	29229249	-	8	0.000705309	-0.009264715	-	-	-	-	-	-	-	-	-	-	-	-	-	-	-	-	-	-
chr1	2426693	2427655	*PLCH2*	4	0.00624941	-0.000451297	-	-	-	4	0.007992432	-0.00048655	-	-	-	4	0.007143805	-0.000579611	-	-	-	-	-	-
chr13	113097140	113097975	-	6	4.75E-07	-0.020235025	-	-	-	6	6.62E-07	-0.020126355	-	-	-	6	5.95E-07	-0.020333073	-	-	-	-	-	-
chr11	17097203	17097511	*RPS13*	3	0.008014511	0.003686319	-	-	-	3	0.009599971	0.003696134	-	-	-	3	0.010454413	0.003447435	-	-	-	-	-	-
chr6	154359967	154360894	*OPRM1*	12	0.004033203	-0.001409827	-	-	-	12	0.004823905	-0.001434098	-	-	-	12	0.005673168	-0.001234717	-	-	-	-	-	-
chr14	60336433	60337477	*RTN1*	7	0.004154606	-0.010070623	-	-	-	7	0.004893747	-0.009957051	-	-	-	7	0.005070672	-0.010080311	-	-	-	-	-	-
chr1	166853278	166853960	-	6	0.007463833	0.005365686	-	-	-	6	0.00679719	0.005502193	-	-	-	6	0.009176722	0.005479709	6	0.001479069	-0.001068911	-	-	-
chr2	198649443	198650603	*BOLL*	13	0.000218356	0.017045096	-	-	-	13	0.000248564	0.017079573	-	-	-	13	8.60E-05	0.017607826	-	-	-	13	0.000923399	0.066728329
chr2	74735875	74736118	*PCGF1*																					
chr22	42679444	42679804	-	3	0.001357524	0.004895865	3	0.000718184	0.006718859	3	0.001622103	0.004861899	-	-	-	3	0.000752625	0.005219159	-	-	-	-	-	-
chr20	45141911	45142576	*ZNF334*	9	0.002008233	-0.006172155	-	-	-	9	0.00242888	-0.006218182	9	0.022582148	-0.003078913	9	0.002692151	-0.006259216	-	-	-	-	-	-
chr1	6313858	6314113	*GPR153*	3	0.002045313	0.019840821	-	-	-	3	0.002536311	0.019783742	-	-	-	3	0.002439134	0.019790198	-	-	-	-	-	-
chr11	94134015	94135029	*GPR83*	13	0.000783861	-0.006475788	-	-	-	13	0.000817689	-0.006583753	-	-	-	13	0.000932196	-0.006309542	-	-	-	-	-	-
chr17	1636606	1637391	*WDR81*	5	0.001298066	0.013645962	-	-	-	-	-	-	-	-	-	-	-	-	-	-	-	-	-	-
chr15	42787188	42788175	*SNAP23*	16	3.83E-07	-0.010333341	-	-	-	16	3.52E-07	-0.010407576	-	-	-	16	2.69E-07	-0.010595577	-	-	-	-	-	-
chr16	57519987	57521330	*DOK4*	11	1.67E-05	-0.010410076	-	-	-	11	1.92E-05	-0.010626586	-	-	-	11	1.34E-05	-0.010546761	-	-	-	-	-	-
chr9	19103069	19104016	*HAUS6*	8	0.002838247	-0.006661527	-	-	-	-	-	-	-	-	-	8	0.002750765	-0.006761837	-	-	-	-	-	-
chr19	49149933	49150388	*CA11*																					
chr19	2307901	2308653	*LINGO3*																					
chr1	53526834	53528224	*PODN*	11	0.003129175	-0.006770286	-	-	-	11	0.004342088	-0.006637246	-	-	-	11	0.004803014	-0.006680954	-	-	-	-	-	-
chr17	201737	203392	*RPH3AL*	12	4.44E-08	-0.012671318	-	-	-	12	7.13E-08	-0.012634023	-	-	-	12	1.11E-07	-0.012155058	-	-	-	-	-	-
chr4	38868940	38869779	*FAM114A1*																					
chr12	6874105	6875642	*MLF2*																					
chr11	61322107	61324205	*SYT7*	10	0.000465828	-0.011058272	-	-	-	10	0.000475113	-0.011030651	-	-	-	10	0.00055816	-0.010947971	-	-	-	-	-	-
chr14	105858487	105859242	*PACS2*	3	4.49E-05	0.013512786	-	-	-	3	5.81E-05	0.013453264	-	-	-	3	5.71E-05	0.013585526	-	-	-	-	-	-
chr19	44668862	44669612	*ZNF226*	13	0.00844547	-0.006333557	-	-	-	13	0.010368373	-0.006318173	-	-	-	13	0.008267752	-0.006494054	-	-	-	-	-	-
chr1	32929715	32931036	*ZBTB8A*																					
chr6	3054085	3054884	-	5	0.00047401	0.009189783	-	-	-	5	0.000605985	0.009085077	-	-	-	5	0.000717786	0.008896795	-	-	-	-	-	-
chr10	102802857	102803372	-	3	0.001657103	-0.007257224	-	-	-	3	0.002076572	-0.007240506	-	-	-	3	0.001999934	-0.007189618	-	-	-	-	-	-
chr9	34457129	34457500	*DNAI1*																					
chr10	106087985	106090233	*ITPRIP*	9	0.001402297	-0.000996205	-	-	-	9	0.001676312	-0.001050742	-	-	-	9	0.001432737	-0.000917042	-	-	-	-	-	-
chr14	102973717	102975405	*ANKRD9*	8	0.002187099	0.011025479	-	-	-	8	0.002526524	0.011010246	-	-	-	8	0.002218978	0.011093039	-	-	-	-	-	-
chr19	18873037	18873268	*CRTC1*	3	0.00051021	-0.019601823	3	0.003773757	-0.023683943	3	0.000646777	-0.019341217	-	-	-	3	0.000500342	-0.019554482	-	-	-	-	-	-
chr1	63988325	63989705	*EFCAB7*																					
chr17	48132714	48133402	*ITGA3*																					
chr1	119531857	119532925	*TBX15*	14	0.007553275	0.000215977	-	-	-	14	0.008192326	0.000238183	-	-	-	14	0.008350807	0.000463899	-	-	-	-	-	-
chr13	53312966	53313529	*CNMD*	3	0.007664958	-0.002943065	-	-	-	3	0.009127137	-0.002976753	-	-	-	3	0.010158091	-0.003056431	-	-	-	-	-	-
chr11	120106598	120107376	*POU2F3*	4	0.004158255	0.000768317	-	-	-	4	0.004944691	0.000840813	-	-	-	4	0.003886294	0.000410637	-	-	-	-	-	-
chr8	67038980	67039461	*TRIM55*	7	0.006283273	0.009802032	-	-	-	7	0.007997407	0.009728489	-	-	-	7	0.009507625	0.009483514	-	-	-	-	-	-
chr10	80008235	80008637	*LINC00856*	4	0.003011281	-0.012459923	-	-	-	4	0.003487211	-0.012568334	-	-	-	4	0.003685471	-0.012547792	-	-	-	-	-	-
chr21	46714226	46715205	*LINC00205*	5	1.60E-05	0.029325826	-	-	-	5	1.94E-05	0.029405508	-	-	-	5	1.79E-05	0.029437755	-	-	-	-	-	-
chr17	53800041	53800484	*TMEM100*	5	0.004036516	-0.010412344	-	-	-	5	0.004990234	-0.010371673	-	-	-	5	0.00461646	-0.010279314	5	0.032367875	-0.013696856	-	-	-
chr2	145090263	145090814	*GTDC1*	8	0.004640908	-0.000638267	-	-	-	8	0.004892363	-0.000682209	-	-	-	8	0.003975774	-0.000754221	-	-	-	-	-	-
chr1	154539523	154540535	*CHRNB2*	7	0.00468928	-0.008829917	-	-	-	7	0.005977436	-0.008758749	-	-	-	7	0.004508445	-0.009158764	-	-	-	-	-	-
chr10	13424851	13425688	-	6	0.006381208	-0.012017446	-	-	-	6	0.008150937	-0.011955764	-	-	-	6	0.009362851	-0.01183631	-	-	-	-	-	-
chr12	56324837	56325736	*DGKA*																					
chr17	21156233	21157499	*NATD1*	12	2.24E-06	-0.01683793	-	-	-	12	3.16E-06	-0.016975248	-	-	-	12	1.10E-06	-0.017199434	-	-	-	-	-	-
chr6	21593587	21594320	*SOX4*	10	0.008087312	-0.001115231	-	-	-	10	0.009646453	-0.001050584	-	-	-	10	0.006491844	-0.0006327	-	-	-	-	-	-
chr3	72226488	72227063	-	4	0.001683016	-0.010933012	-	-	-	4	0.002185497	-0.010825562	-	-	-	4	0.002454325	-0.010765909	-	-	-	-	-	-
chr15	80351213	80352558	*ZFAND6*	17	1.50E-05	-0.010891271	-	-	-	17	2.02E-05	-0.011038429	-	-	-	17	8.73E-06	-0.011093584	-	-	-	-	-	-
chr1	193090350	193091399	*CDC73*	11	0.009702055	-0.000557233	-	-	-	11	0.011772234	-0.000567909	-	-	-	11	0.009352774	-0.000573858	-	-	-	-	-	-
chr15	102189106	102189763	*TM2D3*	3	0.009078263	-0.010003173	-	-	-	3	0.010396556	-0.009949264	-	-	-	3	0.009640763	-0.010343565	-	-	-	-	-	-
chr17	29151515	29152249	*CRLF3*	7	0.009569072	-0.002475058	-	-	-	7	0.011924852	-0.002489282	-	-	-	7	0.006859801	-0.00258336	-	-	-	-	-	-
chr19	36119199	36120543	*RBM42*	13	0.000546488	-0.002375933	-	-	-	13	0.000626081	-0.002374499	-	-	-	13	0.000345799	-0.002396161	-	-	-	-	-	-
chr5	143190915	143192588	*HMHB1*	9	1.18E-09	-0.007025216	-	-	-	9	1.42E-09	-0.007065076	-	-	-	9	3.37E-09	-0.006739966	-	-	-	-	-	-
chr6	30139478	30140713	*TRIM15*	21	0.000738937	-0.002011705	-	-	-	21	0.000841164	-0.002003524	-	-	-	21	0.000987605	-0.001909301	-	-	-	-	-	-
chr3	46617183	46619131	*LRRC2*																					
chr22	42760647	42761604	-	3	0.001243829	0.0237581	-	-	-	3	0.001378474	0.023787864	-	-	-	3	0.00143113	0.023589045	-	-	-	-	-	-
chr15	43809486	43810131	*MAP1A*	7	0.003640004	-0.00737991	-	-	-	7	0.004843364	-0.00721417	-	-	-	7	0.005176105	-0.007029069	-	-	-	-	-	-
chr13	79968174	79968324	*RBM26*	3	0.00188161	0.025576944	-	-	-	3	0.002386582	0.025281213	-	-	-	3	0.002749712	0.024954533	3	0.001054333	0.043958341	-	-	-
chr21	38362230	38363160	*HLCS*	9	0.008390185	0.01796048	-	-	-	9	0.010922877	0.01765503	-	-	-	9	0.009548409	0.017916333	-	-	-	-	-	-
chr10	102510356	102510568	*PAX2*	3	0.005796435	-0.015250466	-	-	-	3	0.006373497	-0.015269942	-	-	-	3	0.005273145	-0.015405991	-	-	-	-	-	-
chr7	100239172	100239764	*TFR2*	4	0.006593109	-0.013140158	-	-	-	4	0.008159279	-0.012771103	-	-	-	4	0.009347663	-0.012730669	-	-	-	-	-	-
chr5	176559334	176560265	*NSD1*	5	0.001614716	0.026537036	-	-	-	5	0.002080496	0.026316662	-	-	-	5	0.000826939	0.027214118	-	-	-	-	-	-
chr3	127633887	127634459	*KBTBD12*	9	0.005654615	0.00979673	-	-	-	9	0.007191929	0.009695315	-	-	-	-	-	-	-	-	-	-	-	-
chr3	107809116	107810687	*CD47*	13	0.005875316	0.002423394	-	-	-	-	-	-	-	-	-	-	-	-	-	-	-	-	-	-
chr11	105479967	105481702	*GRIA4*	21	0.000175034	-0.00537266	-	-	-	-	-	-	-	-	-	-	-	-	-	-	-	-	-	-
chr1	1475143	1476269	*TMEM240*	9	0.005938146	-0.000740059	-	-	-	9	0.00681716	-0.00068762	-	-	-	9	0.004950255	-0.0006075	-	-	-	-	-	-
chr13	28024329	28024921	*MTIF3*	16	0.006862052	0.000156386	-	-	-	16	0.009281571	0.000122162	-	-	-	-	-	-	-	-	-	-	-	-
chr5	133772283	133773142	-	5	0.006472413	-0.009345467	-	-	-	5	0.007897915	-0.009275352	-	-	-	5	0.007343605	-0.009418638	-	-	-	-	-	-
chr2	160568689	160569678	*BAZ2B*																					

chr17	41131552	41132971	*PTGES3L*																					
chr19	294042	295123	*PLPP2*	3	0.0002174	-0.020105226	-	-	-	3	0.000271244	-0.020050702	-	-	-	3	0.000219475	-0.019878397	-	-	-	-	-	-
chr16	20084851	20085897	*GPR139*	10	0.00011585	-0.011299935	10	0.001093773	-0.0142641	-	-	-	-	-	-	10	0.000154101	-0.010907539	-	-	-	-	-	-
chr16	8960833	8962449	*CARHSP1*	13	1.63E-07	0.013642313	-	-	-	-	-	-	-	-	-	-	-	-	-	-	-	-	-	-
chr9	80850389	80850856	*CEP78*	3	0.009767246	-0.008037109	-	-	-	-	-	-	-	-	-	-	-	-	-	-	-	-	-	-
chr19	53661755	53662958	*ZNF347*	11	0.002116703	-0.005880606	-	-	-	11	0.002540049	-0.005901368	-	-	-	11	0.002673758	-0.005694328	-	-	-	-	-	-
chr6	3023598	3024178	*HTATSF1P2*	6	0.006340352	-0.006932732	6	0.024402083	-0.004050366	6	0.006964764	-0.006961438	-	-	-	6	0.006025008	-0.007022569	-	-	-	-	-	-
chr11	22687582	22688474	*GAS2*	9	0.002061078	0.014662784	9	2.47E-05	0.019991344	9	0.002635621	0.014615264	-	-	-	9	0.001506753	0.014996496	-	-	-	-	-	-
chr2	3496821	3497214	-	3	0.006938171	-0.000555259	-	-	-	3	0.007379087	-0.000674816	-	-	-	3	0.007950569	-0.000477489	-	-	-	-	-	-
chr20	58568926	58569565	*CDH26*	4	0.002518769	-0.004203261	-	-	-	4	0.003249337	-0.004160649	-	-	-	4	0.004044742	-0.0040444	-	-	-	-	-	-
chr8	144922348	144923606	*NRBP2*	9	0.007399524	-0.008666314	-	-	-	-	-	-	-	-	-	9	0.005766437	-0.008862926	-	-	-	-	-	-
chr15	93126401	93127379	-	5	0.004562467	0.001511543	-	-	-	5	0.005386115	0.00141395	-	-	-	5	0.005208519	0.001368558	-	-	-	-	-	-
chr7	26577897	26579614	*KIAA0087*	4	0.0009843	0.017221517	-	-	-	4	0.00101193	0.017392167	-	-	-	4	0.001296889	0.016895336	-	-	-	-	-	-
chr11	2720229	2722713	*KCNQ1*																					
chr9	135285195	135286214	*CFAP77*																					
chr10	49892203	49893346	*WDFY4*	11	0.00439604	-0.005263238	-	-	-	-	-	-	-	-	-	11	0.005628589	-0.005300186	-	-	-	-	-	-
chr1	111748053	111748268	*DENND2D*	3	0.009482229	-0.011949566	-	-	-	3	0.011342403	-0.011848884	-	-	-	3	0.012365407	-0.011759437	-	-	-	-	-	-
chr10	32216031	32217076	*ARHGAP12*	8	0.000112584	0.017232332	-	-	-	8	0.00015673	0.017021622	-	-	-	8	0.000131536	0.01721662	-	-	-	-	-	-
chr1	227974177	227975220	-	6	0.005003337	-0.015538843	-	-	-	6	0.005540872	-0.015684786	-	-	-	6	0.006687359	-0.015359784	-	-	-	-	-	-
chr11	122525644	122526355	*UBASH3B*	7	0.00995537	-0.003935058	-	-	-	7	0.011055002	-0.003674266	-	-	-	7	0.010821455	-0.003983503	-	-	-	-	-	-
chr21	27106707	27108257	*ATP5PF*																					
chr9	139948584	139949577	*ENTPD2*	6	0.001493464	0.016095408	-	-	-	6	0.001526773	0.016088411	-	-	-	6	0.001769041	0.016052624	-	-	-	-	-	-
chr8	81397601	81397789	*ZBTB10*	8	0.001723976	-0.006407738	-	-	-	8	0.002330127	-0.006463295	-	-	-	8	0.002042916	-0.006522853	-	-	-	-	-	-
chr5	850745	851101	*ZDHHC11*	3	0.002467217	-0.013786412	-	-	-	3	0.003079582	-0.01353226	-	-	-	3	0.002218978	-0.013863307	-	-	-	-	-	-
chr2	36780691	36780705	*FEZ2*	3	0.004698782	-0.020916364	3	0.017562842	-0.026031414	3	0.005442259	-0.020855066	-	-	-	3	0.006552176	-0.020319238	-	-	-	-	-	-
chr20	1246100	1247058	*SNPH*	9	0.007248175	0.010555	-	-	-	-	-	-	-	-	-	-	-	-	-	-	-	-	-	-
chr4	2401036	2402046	*ZFYVE28*	5	0.00427822	0.012672942	-	-	-	5	0.004982223	0.012648747	-	-	-	5	0.004059192	0.01247455	-	-	-	-	-	-
chr8	144659492	144661051	*MROH6*																					
chr12	131872163	131872391	-	3	0.004934687	-0.015501359	-	-	-	3	0.006034323	-0.015387598	3	0.007878386	-0.043842958	3	0.005848611	-0.015392148	-	-	-	-	-	-
chr14	74178976	74180391	*PNMA1*	6	0.004445571	0.004278439	-	-	-	-	-	-	-	-	-	-	-	-	-	-	-	-	-	-
chr3	51742164	51742878	*GRM2*	4	0.004722932	0.003209832	-	-	-	4	0.005303	0.003062152	-	-	-	4	0.005652341	0.002960763	-	-	-	-	-	-
chr4	567537	568514	-	4	0.000950617	0.003639563	-	-	-	4	0.00115469	0.003567629	-	-	-	4	0.000852961	0.003914134	-	-	-	-	-	-
chr16	2479381	2480552	*CCNF*	6	0.002518769	0.000766977	-	-	-	-	-	-	-	-	-	6	0.002218978	0.000728272	-	-	-	-	-	-
chr17	53341098	53343075	*HLF*	12	0.009905544	-0.005600106	-	-	-	-	-	-	-	-	-	12	0.010765923	-0.005774891	-	-	-	-	-	-
chr6	137815333	137816901	*OLIG3*	11	0.00455534	-0.001986315	-	-	-	-	-	-	-	-	-	-	-	-	-	-	-	-	-	-
chr19	1265302	1266391	*CIRBP*	6	2.15E-08	0.011999164	6	2.53E-11	0.017061555	6	3.47E-08	0.011899039	-	-	-	6	3.23E-08	0.011806995	-	-	-	-	-	-
chr18	23713407	23714084	*PSMA8*	12	0.000216217	0.015831814	-	-	-	12	0.000282705	0.015292268	-	-	-	12	0.000165898	0.016110174	-	-	-	-	-	-
chr22	23160841	23161379	*MIR650*	4	0.001280756	-0.008686835	-	-	-	4	0.001578343	-0.008713283	-	-	-	4	0.001122397	-0.008690706	-	-	-	-	-	-
chr10	108923111	108924867	*SORCS1*	9	0.005501899	-0.012111205	-	-	-	9	0.006920219	-0.01188338	-	-	-	9	0.008446366	-0.011715447	-	-	-	-	-	-
chr4	83719449	83720629	*SCD5*	13	0.001855182	-0.00424157	-	-	-	13	0.002128343	-0.00422437	-	-	-	13	0.002635793	-0.004216081	-	-	-	-	-	-
chr1	156675322	156675879	*CRABP2*	13	0.009234317	0.001102695	-	-	-	13	0.011983671	0.00108758	-	-	-	13	0.012577009	0.000999528	-	-	-	-	-	-
chr2	32490589	32491049	*NLRC4*	6	0.005995378	-0.008732615	-	-	-	6	0.007455212	-0.008620846	-	-	-	6	0.006449144	-0.008660648	-	-	-	-	-	-
chr15	74724562	74725593	*SEMA7A*	3	1.68E-09	0.017398248	-	-	-	3	1.82E-09	0.017298344	-	-	-	3	3.47E-09	0.017117323	-	-	-	-	-	-
chr1	17766238	17767282	*RCC2*	6	1.69E-05	0.003420664	-	-	-	-	-	-	-	-	-	6	2.38E-05	0.003156406	-	-	-	-	-	-
chr12	6304557	6305192	*CD9*	6	0.001152773	0.008644464	6	0.006006683	0.010960051	6	0.001341418	0.008634289	-	-	-	6	0.001771134	0.008456339	-	-	-	-	-	-
chr13	79169714	79171230	*OBI1-AS1*	14	0.007247149	-0.003177439	-	-	-	-	-	-	-	-	-	-	-	-	-	-	-	-	-	-
chr4	184186563	184186934	*WWC2*	4	0.004381358	0.018441726	-	-	-	4	0.005046241	0.018444641	-	-	-	4	0.005252677	0.018375085	-	-	-	-	-	-
chr1	21877524	21877781	*ALPL*	5	0.008499527	0.015584069	-	-	-	5	0.009517182	0.015439415	-	-	-	5	0.010037218	0.015508219	-	-	-	-	-	-
chr20	43378953	43379501	*KCNK15*	4	0.001523403	0.019362778	-	-	-	4	0.002051527	0.01934338	-	-	-	4	0.001805839	0.019428735	-	-	-	-	-	-
chr11	45392566	45393309	-	9	0.00065425	-0.008044389	-	-	-	9	0.000741911	-0.008133653	-	-	-	9	0.000914525	-0.008122839	-	-	-	-	-	-
chr18	67624071	67624393	*CD226*	3	0.002136426	-0.016503316	-	-	-	3	0.001260669	-0.016895492	-	-	-	3	0.002795567	-0.015612925	-	-	-	-	-	-
chr1	870791	871546	*SAMD11*	7	0.000105272	-0.03704767	-	-	-	7	0.000149776	-0.036492549	-	-	-	7	0.000121211	-0.03661666	-	-	-	-	-	-
chr15	34806592	34807221	-	5	0.00202147	-0.006707387	-	-	-	5	0.002128963	-0.006741818	-	-	-	5	0.00242655	-0.006686842	-	-	-	-	-	-
chr8	130950848	130951373	*CYRIB*	3	0.0048149	-0.017064887	-	-	-	3	0.005614633	-0.017080991	-	-	-	3	0.005446936	-0.0172106	-	-	-	-	-	-
chr5	399012	400201	*AHRR*	5	0.000482098	0.007301223	-	-	-	5	0.000522464	0.007264441	-	-	-	5	0.000418338	0.007526066	-	-	-	-	-	-
chr13	20692403	20693122	-	6	0.008101773	0.007083346	-	-	-	6	0.010003813	0.006983788	-	-	-	6	0.008992785	0.007012968	-	-	-	-	-	-
chr3	22412124	22414843	*ZNF385D*	10	4.49E-12	-0.035382375	10	1.97E-10	-0.041869164	10	5.97E-12	-0.035371504	-	-	-	10	7.97E-12	-0.034993682	-	-	-	-	-	-
chr15	23809945	23811572	*MIR4508*																					
chr2	69968414	69969591	*ANXA4*	14	0.00050489	-0.004675091	-	-	-	14	0.00062928	-0.004712923	-	-	-	14	0.000405055	-0.004736415	-	-	-	-	-	-
chr2	220348515	220349208	*SPEG*	6	0.005334716	-0.010366489	6	0.017198379	-0.009512948	6	0.006151557	-0.010344413	-	-	-	6	0.005974717	-0.010260176	-	-	-	-	-	-
chr15	98835851	98837004	-	12	0.001065657	-0.00478684	-	-	-	12	0.001369117	-0.004757966	-	-	-	12	0.001613527	-0.00458743	-	-	-	-	-	-
chr7	149416432	149416973	*KRBA1*	9	0.003197885	0.004753536	-	-	-	9	0.004506356	0.004663323	-	-	-	9	0.004054051	0.004636715	-	-	-	-	-	-
chr8	61496287	61497168	*RAB2A*	4	0.009046649	-0.00926934	-	-	-	4	0.00910888	-0.009343901	-	-	-	4	0.00815337	-0.009549235	-	-	-	-	-	-
chr19	15121199	15121596	*CCDC105*																					
chr18	22932076	22932673	*ZNF521*	5	0.008101773	-0.00411721	-	-	-	5	0.009838042	-0.004095784	-	-	-	5	0.008135778	-0.004103612	-	-	-	-	-	-
chr14	105647194	105648697	*NUDT14*	9	5.73E-05	-0.006467101	-	-	-	9	7.40E-05	-0.006514131	-	-	-	9	7.61E-05	-0.00663932	-	-	-	-	-	-
chr12	129299088	129299702	*SLC15A4*	7	0.004337256	-0.011657442	-	-	-	7	0.005293389	-0.011600648	-	-	-	7	0.004867686	-0.011547008	-	-	-	-	-	-
chr12	58138320	58139148	*AGAP2*																					
chr2	79738545	79739278	*CTNNA2*	6	0.000442101	-0.019705643	-	-	-	6	0.000631927	-0.019566067	-	-	-	6	0.001010133	-0.01866523	-	-	-	-	-	-
chr17	48911141	48913424	*WFIKKN2*	26	8.70E-22	0.018522989	-	-	-	26	2.49E-21	0.018513702	-	-	-	26	2.43E-21	0.018584537	-	-	-	-	-	-
chr3	194905391	194906452	*XXYLT1*	5	0.009800996	-0.002956495	-	-	-	5	0.012161448	-0.002840165	-	-	-	5	0.010634403	-0.002919792	-	-	-	-	-	-
chr1	86047239	86048585	*CCN1*																					
chr20	24973741	24974513	*APMAP*	5	0.000983512	-0.012736438	-	-	-	5	0.001287769	-0.012725188	-	-	-	5	0.001064428	-0.012857727	-	-	-	-	-	-
chr12	122230490	122230972	*RHOF*	3	0.00606801	0.006510861	-	-	-	3	0.005489047	0.006589379	-	-	-	3	0.006935751	0.006492153	-	-	-	-	-	-
chr3	14345227	14346268	-	6	0.009140907	0.014418948	-	-	-	-	-	-	-	-	-	6	0.01062564	0.014247436	-	-	-	-	-	-
chr9	34662282	34663048	*CCL27*	5	0.005118896	0.007885533	-	-	-	5	0.00596976	0.007733864	-	-	-	5	0.006835705	0.007579095	-	-	-	-	-	-
chr1	156163116	156164745	*SLC25A44*	14	0.000551725	0.005029138	-	-	-	14	0.000863261	0.00493228	-	-	-	14	0.000801517	0.004925821	-	-	-	-	-	-
chr12	133185615	133185655	*LRCOL1*	3	0.008065176	0.024514796	-	-	-	3	0.00993633	0.024165165	-	-	-	3	0.010542688	0.023984889	-	-	-	-	-	-
chr6	32115979	32116317	*PPT2*																					
chr10	75007255	75008351	*DNAJC9*																					
chr5	148651771	148652108	*AFAP1L1*	3	0.005700091	-0.004096954	-	-	-	3	0.005640547	-0.004128923	-	-	-	3	0.004735572	-0.004093234	-	-	-	-	-	-
chr2	206546299	206547163	*NRP2*	9	0.007856618	-0.004336596	-	-	-	-	-	-	-	-	-	9	0.008583834	-0.004391658	-	-	-	-	-	-
chr6	56111812	56113034	*COL21A1*	9	4.68E-06	-0.003376419	-	-	-	9	7.33E-06	-0.003426021	-	-	-	9	6.65E-06	-0.002869496	-	-	-	-	-	-
chr14	38063480	38065435	*FOXA1*	18	1.32E-08	-0.003645152	-	-	-	-	-	-	-	-	-	18	3.06E-08	-0.00345286	-	-	-	-	-	-
chr16	73419299	73420528	*LINC01568*	5	0.008957003	0.014107052	-	-	-	5	0.010217511	0.014062725	-	-	-	5	0.005979717	0.01452731	-	-	-	-	-	-
chr1	32665582	32666481	*CCDC28B*																					
chr20	741723	742683	*SLC52A3*	4	0.000732102	0.017702187	-	-	-	4	0.000752487	0.017838073	-	-	-	4	0.000493618	0.018317802	-	-	-	-	-	-
chr1	113216243	113217204	*MOV10*	13	0.002051012	-0.005883886	-	-	-	13	0.002637996	-0.005884663	-	-	-	13	0.002425268	-0.005873176	-	-	-	-	-	-
chr16	87492071	87492801	*ZCCHC14*	5	0.006449139	-0.000800119	-	-	-	5	0.007435229	-0.000812972	-	-	-	5	0.006409343	-0.000677932	-	-	-	-	-	-
chr7	27154537	27155974	*HOXA3*	15	0.001292215	0.011427176	-	-	-	15	0.001566121	0.011432215	-	-	-	15	0.001366485	0.011456917	-	-	-	-	-	-
chr10	135333451	135334122	*SCART1*	7	0.000932104	-0.01169644	-	-	-	-	-	-	-	-	-	-	-	-	-	-	-	-	-	-
chr22	38507451	38508132	*BAIAP2L2*																					
chr1	236557165	236559675	*EDARADD*	19	7.23E-07	0.011232273	-	-	-	19	7.31E-07	0.011088345	-	-	-	19	1.92E-06	0.010620808	-	-	-	-	-	-
chr3	137487770	137489312	-	7	1.05E-05	-0.00184741	-	-	-	7	1.41E-05	-0.001788415	-	-	-	7	1.15E-05	-0.001744755	-	-	-	-	-	-
chr6	16217234	16218436	-	5	0.000852747	-0.027227531	-	-	-	5	0.001099837	-0.027102452	-	-	-	5	0.000688057	-0.027615422	-	-	-	-	-	-
chr8	86350278	86351195	*CA13*																					
chr12	51717674	51719349	*BIN2*	14	0.007314773	-0.009058176	-	-	-	14	0.009263812	-0.009093381	-	-	-	14	0.008649983	-0.009158878	-	-	-	-	-	-
chr11	123524938	123526191	*SCN3B*	10	2.49E-05	-0.01005003	-	-	-	10	3.77E-05	-0.009987008	-	-	-	10	5.04E-05	-0.009835607	-	-	-	-	-	-
chr11	107729479	107730551	*SLC35F2*	13	0.001115086	-0.005983208	-	-	-	13	0.001585598	-0.006005688	-	-	-	13	0.001053916	-0.00613346	-	-	-	-	-	-
chr14	74100086	74100788	-	6	0.009236479	-0.008951063	-	-	-	-	-	-	-	-	-	-	-	-	-	-	-	-	-	-
chr8	68864012	68865187	*PREX2*	13	0.003166367	-0.003928452	-	-	-	13	0.003976867	-0.003829469	-	-	-	13	0.00521504	-0.003682886	-	-	-	-	-	-
chr10	90640622	90641389	*STAMBPL1*	3	0.007383476	0.00282255	-	-	-	3	0.008376116	0.002747924	-	-	-	3	0.007670705	0.002513241	-	-	-	-	-	-
chr5	154998	155199	*PLEKHG4B*	4	3.72E-05	0.022363964	4	8.76E-06	0.031409289	4	4.52E-05	0.022352366	-	-	-	4	5.82E-05	0.02216718	-	-	-	-	-	-
chr5	2751822	2752882	*C5orf38*																					
chr15	41061384	41062224	*C15orf62*																					
chr11	76812596	76813364	*CAPN5*																					
chr2	239139911	239140910	*LINC02610*																					
chr6	28757018	28757569	-	4	0.000748643	-0.028439213	-	-	-	4	0.000788782	-0.028516474	-	-	-	4	0.001029358	-0.027823079	-	-	-	-	-	-
chr19	1961017	1961727	*CSNK1G2*	5	0.001509975	0.0038546	-	-	-	5	0.001814362	0.003861389	-	-	-	5	0.001535829	0.004045669	-	-	-	-	-	-
chr2	121303344	121304074	-	3	0.005882924	0.00049358	-	-	-	3	0.007080931	0.000590817	-	-	-	3	0.006550407	0.000262282	-	-	-	-	-	-
chr6	3258005	3259961	*PSMG4*	15	0.00512349	-0.005604935	-	-	-	15	0.006537019	-0.005567179	-	-	-	15	0.006826096	-0.005592691	-	-	-	-	-	-

chr12	55725479	55725991	*OR6C3*	3	0.001729257	-0.004299554	-	-	-	3	0.002093127	-0.00445771	-	-	-	3	0.002121444	-0.004453283	-	-	-	-	-	-
chr1	95006977	95008479	*F3*	14	0.001608691	-0.00283054	-	-	-	14	0.001282109	-0.002799956	-	-	-	14	0.001200692	-0.002818008	-	-	-	-	-	-
chr15	65203541	65204428	*ANKDD1A*	13	0.006051237	0.001363721	-	-	-	13	0.007910556	0.001383001	-	-	-	13	0.008329471	0.001429092	-	-	-	-	-	-
chr6	29648161	29649084	*ZFP57*	21	1.27E-08	0.046436136	-	-	-	21	2.60E-08	0.046030147	-	-	-	21	1.60E-08	0.046866444	-	-	-	-	-	-
chr5	28809037	28810645	-	9	3.38E-05	0.018034286	-	-	-	9	4.15E-05	0.017870702	9	0.001405297	0.064894105	9	3.16E-05	0.018075932	-	-	-	-	-	-
chr19	42927668	42929067	*LIPE*																					
chr13	67802655	67805065	*PCDH9*	18	2.47E-05	-0.011136684	-	-	-	18	3.63E-05	-0.011038901	-	-	-	18	2.53E-05	-0.011208689	-	-	-	-	-	-
chr8	6481431	6481608	*MCPH1*	3	0.001290472	-0.020241091	3	0.006000936	-0.024892538	3	0.001529528	-0.019999993	-	-	-	3	0.001854196	-0.019663554	3	0.022446629	-0.031679628	-	-	-
chr2	102927397	102927898	*IL18R1*																					
chr14	54421010	54422357	*BMP4*	11	4.31E-07	-0.005645751	-	-	-	-	-	-	-	-	-	11	5.57E-07	-0.005599263	-	-	-	-	-	-
chr11	118965121	118966503	*H2AX*	11	0.000432059	0.000393608	-	-	-	11	0.00045662	0.000380457	-	-	-	11	0.000301821	0.000359481	-	-	-	-	-	-
chr15	88798666	88799707	*NTRK3*																					
chr18	74276822	74277491	-	3	0.002915986	-0.008763901	-	-	-	3	0.00361984	-0.008720667	-	-	-	3	0.003953591	-0.008662014	-	-	-	-	-	-
chr3	101659630	101660066	*LINC02085*	3	0.001498735	-0.015724231	-	-	-	3	0.001782337	-0.01562474	-	-	-	3	0.002175829	-0.015363326	-	-	-	-	-	-
chr1	153721986	153722548	*INTS3*	4	0.000578235	-0.004813206	4	0.016403019	-0.005886868	4	0.000653475	-0.004718327	-	-	-	4	0.000586686	-0.004776651	-	-	-	-	-	-
chr5	137773712	137775041	*REEP2*	13	0.00051318	-0.008444726	-	-	-	13	0.000695361	-0.008442229	13	0.013516665	-0.006449245	13	0.000556641	-0.008482025	-	-	-	-	-	-
chr10	20104935	20106089	*PLXDC2*	11	0.000920541	-0.005051467	-	-	-	-	-	-	-	-	-	-	-	-	-	-	-	-	-	-
chr11	395625	397613	*PKP3*	10	0.001006634	-0.015557992	-	-	-	10	0.001363398	-0.015493591	-	-	-	10	0.000749066	-0.016173064	-	-	-	-	-	-
chr4	56211228	56211433	*SRD5A3*	3	0.003559908	-0.005118637	-	-	-	3	0.004639336	-0.005059276	-	-	-	3	0.00387637	-0.005014125	-	-	-	-	-	-
chr12	130936355	130937365	*RIMBP2*	7	0.006256097	-0.008994032	-	-	-	7	0.006836038	-0.009070317	-	-	-	-	-	-	-	-	-	-	-	-
chr11	115860682	115861018	-	5	0.003357518	-0.024545745	-	-	-	5	0.003776658	-0.024549115	-	-	-	5	0.003887606	-0.024563614	-	-	-	-	-	-
chr6	33047185	33049505	*HLA-DPA1*																					
chr2	26726190	26726995	*OTOF*	5	0.002002139	0.00434033	-	-	-	5	0.00247393	0.004299164	-	-	-	5	0.002276791	0.004354682	-	-	-	-	-	-
chr11	59210634	59211431	*OR5A1*	3	0.006544938	0.008789848	-	-	-	3	0.007313534	0.008794484	-	-	-	3	0.007681292	0.008646177	-	-	-	-	-	-
chr19	17830116	17831088	*MAP1S*	11	0.007462633	-0.00393098	-	-	-	11	0.009082345	-0.003924867	-	-	-	-	-	-	-	-	-	-	-	-
chr14	101499152	101499893	*MIR495*	4	0.005125279	0.019422001	-	-	-	-	-	-	4	0.019082858	0.066514152	4	0.005575021	0.019611923	-	-	-	-	-	-
chr19	10735474	10736448	*SLC44A2*	10	6.69E-05	0.02115741	-	-	-	10	8.46E-05	0.021159153	-	-	-	10	2.51E-05	0.021814847	-	-	-	-	-	-
chr20	60294656	60295700	*CDH4*	6	0.000133006	-0.021734259	-	-	-	6	0.000107797	-0.022006414	6	3.58E-05	-0.092473957	6	0.000123971	-0.022061848	-	-	-	-	-	-
chr17	55938642	55939590	*CUEDC1*	7	0.004332211	-0.001829544	-	-	-	7	0.004739088	-0.001865397	-	-	-	7	0.003930784	-0.001772909	-	-	-	-	-	-
chr2	95831031	95831943	*ZNF2*	10	0.007019807	-0.002104868	-	-	-	-	-	-	-	-	-	-	-	-	-	-	-	-	-	-
chr11	62344463	62344975	*EEF1G*																					
chr17	48636344	48638703	*CACNA1G*																					
chr9	95857577	95858671	*CARD19*	13	0.007110248	-0.007346657	-	-	-	13	0.009342004	-0.007408583	-	-	-	13	0.006859228	-0.007526094	-	-	-	-	-	-
chr8	59571055	59571961	*NSMAF*	5	0.008322249	-0.000589082	-	-	-	-	-	-	-	-	-	-	-	-	-	-	-	-	-	-
chr1	169677313	169677921	*C1orf112*																					
chr17	74072957	74074118	*GALR2*																					
chr11	123300323	123301490	-	10	0.004733401	-0.001090712	-	-	-	10	0.004974282	-0.001119366	-	-	-	10	0.003525017	-0.001133958	-	-	-	-	-	-
chr14	92572097	92573147	*ATXN3*	12	0.004241034	-0.003780345	-	-	-	-	-	-	-	-	-	-	-	-	-	-	-	-	-	-
chr3	196594273	196595444	*SENP5*	11	0.008318586	-0.00426637	-	-	-	11	0.010024082	-0.004174161	-	-	-	11	0.010063415	-0.004267356	-	-	-	-	-	-
chr2	121411622	121412432	-	5	0.007653683	-0.01803478	-	-	-	-	-	-	-	-	-	5	0.007118131	-0.018034082	-	-	-	-	-	-
chr4	72119734	72120167	*SLC4A4*	3	3.19E-07	-0.040238087	3	0.000125245	-0.043268241	3	4.31E-07	-0.040072968	-	-	-	3	5.11E-07	-0.039794874	-	-	-	-	-	-
chr16	401986	403022	*AXIN1*	4	0.004476479	-0.027679705	-	-	-	4	0.005253834	-0.027798902	-	-	-	4	0.004497625	-0.027953795	-	-	-	-	-	-
chr19	11958966	11960208	*ZNF439*	6	0.005936356	-0.001115229	-	-	-	6	0.007499989	-0.001063398	-	-	-	6	0.007896699	-0.000883137	-	-	-	-	-	-
chr16	77468970	77470221	*ADAMTS18*	10	0.004871898	-0.009833795	-	-	-	-	-	-	-	-	-	10	0.005296301	-0.009776361	-	-	-	-	-	-
chr2	220298547	220300568	*SPEG*	16	5.68E-07	0.014405211	-	-	-	16	8.74E-07	0.014289595	-	-	-	16	7.26E-07	0.014348004	-	-	-	-	-	-
chr10	94607768	94608332	*EXOC6*	11	0.008469451	-0.00322636	-	-	-	11	0.010569062	-0.003234896	-	-	-	11	0.008992785	-0.003364653	-	-	-	-	-	-
chr6	31670195	31671391	*ABHD16A*																					
chr21	46798244	46798617	-	3	0.002351952	0.017039429	-	-	-	3	0.00242888	0.017098191	-	-	-	3	0.002728482	0.016984396	-	-	-	-	-	-
chr12	14720100	14722066	*PLBD1*	16	5.75E-09	0.015256198	-	-	-	16	9.70E-09	0.015166061	-	-	-	16	1.60E-09	0.0156661	-	-	-	-	-	-
chr12	52240368	52242166	-	9	0.003092866	0.003547166	-	-	-	9	0.00351518	0.003458677	-	-	-	9	0.003249212	0.003524576	-	-	-	-	-	-
chr13	28395271	28396059	-	7	0.008847177	-0.008384611	-	-	-	7	0.01029337	-0.008344377	-	-	-	7	0.011437983	-0.008398612	-	-	-	-	-	-
chr4	125634258	125635220	*ANKRD50*	5	0.000819483	-0.013004097	-	-	-	5	0.001087965	-0.012858157	5	0.002922498	-0.058511174	5	0.001107792	-0.012789114	-	-	-	-	-	-
chr21	40123882	40124848	*LINC00114*	5	1.53E-07	-0.003996751	5	0.000837722	-0.007508802	5	2.15E-07	-0.003948131	-	-	-	5	6.54E-08	-0.003878719	5	0.005681734	-0.00474029	-	-	-
chr20	33813236	33814184	*EDEM2*																					
chr1	9351985	9352663	*SPSB1*	5	0.005617419	0.008972804	-	-	-	-	-	-	-	-	-	5	0.005927244	0.008985429	-	-	-	-	-	-
chr11	113932106	113934010	*ZBTB16*	9	0.003292992	-0.010493711	-	-	-	9	0.004168631	-0.010345294	-	-	-	9	0.003790494	-0.010499483	-	-	-	-	-	-
chr1	209798721	209799353	*LAMB3*																					
chr3	23244051	23244348	*UBE2E2*	8	0.004442599	0.00333476	-	-	-	8	0.004757791	0.003404428	-	-	-	8	0.003675613	0.00337119	-	-	-	-	-	-
chr1	111217406	111218554	*KCNA3*	11	9.22E-07	0.008432665	-	-	-	11	1.50E-06	0.008327632	-	-	-	11	1.49E-06	0.008283959	-	-	-	-	-	-
chr9	29212927	29213880	*LINGO2*	6	0.001427796	-0.007715192	-	-	-	6	0.001576615	-0.007645679	-	-	-	6	0.001881487	-0.00761221	-	-	-	-	-	-
chr17	19771360	19772219	*ULK2*	8	0.001854711	0.019618289	-	-	-	8	0.002235749	0.019541558	-	-	-	8	0.000640973	0.020198103	-	-	-	-	-	-
chr18	30051012	30051803	*GAREM1*	19	0.009691996	-0.002816583	-	-	-	-	-	-	-	-	-	19	0.009138531	-0.002775577	-	-	-	-	-	-
chr7	151503515	151503608	*PRKAG2*	4	0.00225731	-0.013548243	4	0.040436429	-0.015103807	4	0.002197431	-0.013650981	-	-	-	4	0.002042916	-0.013690937	-	-	-	-	-	-
chr6	27258466	27259237	-	9	0.002760386	0.005319562	-	-	-	9	0.003463137	0.005335411	-	-	-	9	0.002531528	0.005589381	-	-	-	-	-	-
chr4	143766602	143768275	*INPP4B*	13	0.000502104	-0.002303089	-	-	-	13	0.000691478	-0.002287751	-	-	-	13	0.000520794	-0.002376223	-	-	-	-	-	-
chr19	16998493	17001305	*F2RL3*	15	2.99E-21	0.013602103	15	1.20E-15	0.014038409	15	6.62E-21	0.013591883	-	-	-	15	3.90E-21	0.013595077	-	-	-	-	-	-
chr11	6947759	6948211	*ZNF215*	5	0.007869919	-0.000534251	-	-	-	5	0.008811905	-0.000492442	-	-	-	5	0.009004276	-0.000379631	-	-	-	-	-	-
chr12	52416192	52417019	*NR4A1*	8	0.000200914	0.010154109	-	-	-	8	0.00026359	0.010118235	-	-	-	8	0.000345237	0.009946049	8	0.009875533	0.013783228	-	-	-
chr12	39538604	39539558	-	6	0.006479627	0.016246767	-	-	-	6	0.008340932	0.015976417	-	-	-	6	0.00598386	0.016475293	-	-	-	-	-	-
chr17	31254544	31255635	*TMEM98*	12	0.000551855	-0.006941871	-	-	-	12	0.000793692	-0.006900902	-	-	-	12	0.000987605	-0.006818764	-	-	-	-	-	-
chr5	175298571	175298869	*CPLX2*	3	0.007045222	-0.013551148	-	-	-	3	0.008570633	-0.013544726	-	-	-	3	0.008629053	-0.013536019	-	-	-	-	-	-
chr5	155753336	155754265	*SGCD*	5	0.006296295	0.012680442	-	-	-	-	-	-	-	-	-	5	0.005590147	0.012194115	-	-	-	-	-	-
chr5	74807815	74808931	*CERT1*																					
chr2	39187533	39188006	*ARHGEF33*																					
chr11	69631981	69633070	*FGF3*	8	0.003129175	-0.006751878	-	-	-	8	0.003614989	-0.006707055	-	-	-	8	0.003239875	-0.006800755	-	-	-	-	-	-
chr1	25256369	25258332	*RUNX3*	28	0.000190448	0.005341543	-	-	-	28	0.000245012	0.005372709	-	-	-	28	0.000141005	0.005514141	-	-	-	-	-	-
chr3	73610847	73611772	*PDZRN3*	6	0.00082135	-0.003999428	-	-	-	-	-	-	-	-	-	6	0.000699774	-0.004146994	-	-	-	-	-	-
chr3	133748115	133748812	*SLCO2A1*	7	0.003634002	-0.011075471	-	-	-	7	0.004714391	-0.010940211	-	-	-	7	0.004110478	-0.011076832	-	-	-	-	-	-
chr16	89951702	89951949	*TCF25*	3	0.004979043	-0.008262476	-	-	-	3	0.006017305	-0.008160412	-	-	-	3	0.006213897	-0.008181086	-	-	-	-	-	-
chr11	78672632	78673290	*TENM4*	5	0.008473029	0.000125772	-	-	-	5	0.010547801	-8.96E-06	-	-	-	5	0.009209759	0.000439098	-	-	-	-	-	-
chr3	11643341	11643630	*VGLL4*	5	1.49E-05	-0.021926198	-	-	-	5	2.01E-05	-0.021606366	-	-	-	5	2.66E-05	-0.021510385	5	0.046730629	-0.025294823	-	-	-
chr20	61147144	61148218	*MIR1-1*																					
chr4	146401952	146402943	*SMAD1*	6	0.003799339	-0.007853966	-	-	-	-	-	-	-	-	-	6	0.003147635	-0.007737946	-	-	-	-	-	-
chr16	90014962	90016004	*DEF8*	8	0.001744822	-0.010793873	-	-	-	-	-	-	-	-	-	-	-	-	-	-	-	-	-	-
chr2	26205095	26206226	*KIF3C*	10	0.000420039	0.002826659	-	-	-	10	0.000563353	0.002830883	-	-	-	10	0.000304985	0.002855645	-	-	-	-	-	-
chr1	41284582	41285496	*KCNQ4*	5	0.002621946	0.015108936	-	-	-	5	0.003244845	0.014874074	-	-	-	5	0.002189206	0.015275142	-	-	-	-	-	-
chr11	112034801	112035434	*IL18*																					
chr9	130540941	130541531	*SH2D3C*	6	0.004635858	-0.00788124	-	-	-	6	0.006073577	-0.007767857	-	-	-	6	0.005823626	-0.008036927	-	-	-	-	-	-
chr17	7164634	7166281	*CLDN7*	13	7.97E-05	0.00588697	-	-	-	13	0.000107097	0.005854478	-	-	-	-	-	-	-	-	-	-	-	-
chr3	29684415	29685093	*RBMS3*	4	0.001065676	0.004101501	-	-	-	4	0.00110161	0.003929914	-	-	-	4	0.001092781	0.003947499	-	-	-	-	-	-
chr2	236504134	236504635	*AGAP1*	3	0.006632354	-0.008775778	-	-	-	3	0.007709216	-0.008827023	-	-	-	3	0.007667065	-0.008786042	-	-	-	-	-	-
chr16	67261905	67263524	*FHOD1*																					
chr9	118501557	118502187	-	4	0.001575491	-0.010760915	-	-	-	4	0.001702803	-0.010809658	-	-	-	4	0.001187934	-0.010968116	-	-	-	-	-	-
chr14	81425912	81426577	*CEP128*																					
chr10	134150119	134150760	*LRRC27*																					
chr6	144385609	144387124	*PLAGL1*	19	2.25E-05	0.007680264	-	-	-	19	1.82E-05	0.007679211	-	-	-	19	1.69E-05	0.007591634	-	-	-	-	-	-
chr21	37535372	37536685	*DOP1B*	6	0.000971828	-0.00725457	-	-	-	-	-	-	-	-	-	-	-	-	-	-	-	-	-	-
chr5	178957344	178957930	-	4	0.006235153	0.002822701	4	0.002411342	0.003884692	4	0.007161539	0.00286481	-	-	-	4	0.005326049	0.003195571	-	-	-	-	-	-
chr16	1574269	1575414	*IFT140*																					
chr6	53409850	53411302	*GCLC*	12	0.001593751	0.002392635	-	-	-	12	0.002012684	0.002346371	-	-	-	12	0.001087775	0.002424837	-	-	-	-	-	-
chr16	1122630	1123333	*SSTR5*																					
chr8	6665912	6666452	*XKR5*	3	0.006381208	0.012796322	-	-	-	3	0.00731814	0.012612399	-	-	-	3	0.008501206	0.012454607	-	-	-	-	-	-
chr8	10282549	10283275	*MSRA*	6	0.003503288	-0.006179007	-	-	-	-	-	-	-	-	-	-	-	-	-	-	-	-	-	-
chr1	220101368	220102528	*RNU5F-1*																					
chr13	103719706	103720206	*SLC10A2*	3	0.005896745	-0.004553921	-	-	-	-	-	-	-	-	-	-	-	-	-	-	-	-	-	-
chr6	31855695	31857040	*EHMT2*	23	0.004959771	0.004469817	-	-	-	-	-	-	-	-	-	23	0.005382286	0.004406621	-	-	-	-	-	-
chr19	5977966	5978790	*LOC100128568*																					
chr10	7708240	7709160	*ITIH5*	11	0.003708678	-0.011965405	-	-	-	11	0.00585667	-0.011866161	-	-	-	11	0.004295646	-0.011985169	-	-	-	-	-	-
chr11	73498113	73498948	*MRPL48*	8	0.008367236	0.007145731	-	-	-	-	-	-	-	-	-	8	0.010001395	0.006978937	-	-	-	-	-	-
chr8	67940640	67941857	*PPP1R42*	12	0.004705928	-0.00026691	-	-	-	12	0.005772988	-0.000203047	-	-	-	12	0.004419636	-0.000172082	-	-	-	-	-	-
chr18	29598757	29599349	*RNF125*	4	0.00614728	-0.002874498	-	-	-	4	0.008103423	-0.002857715	-	-	-	4	0.006252958	-0.002988257	-	-	-	-	-	-

chr2	240144335	240146162	*HDAC4*	5	0.000974161	-0.016658597	-	-	-	5	0.001187321	-0.016387483	-	-	-	5	0.001328372	-0.016311118	-	-	-	-	-	-
chr6	33172333	33173581	*HSD17B8*																					
chr21	46964226	46965042	*SLC19A1*	7	0.000957846	0.014346153	-	-	-	7	0.001270765	0.014222206	-	-	-	7	0.000960482	0.014579515	-	-	-	-	-	-
chr1	218523325	218524558	*TGFB2*																					
chr5	112072926	112073958	*APC*	16	0.002190045	-0.006311345	-	-	-	16	0.002690474	-0.006309492	-	-	-	16	0.002614	-0.006393438	-	-	-	-	-	-
chr7	751830	753007	*PRKAR1B*	15	0.007567225	-0.003619059	-	-	-	-	-	-	-	-	-	15	0.008483329	-0.003623663	-	-	-	-	-	-
chr3	57331843	57332341	*DNAH12*	3	0.004466452	0.008744005	-	-	-	3	0.004986597	0.008783977	-	-	-	3	0.00549233	0.008705736	-	-	-	-	-	-
chr4	1736433	1737129	*TACC3*	4	0.007977698	0.013672737	-	-	-	-	-	-	-	-	-	4	0.006901309	0.01396716	-	-	-	-	-	-
chr7	154684051	154685308	*DPP6*	11	0.000203417	-0.016884281	-	-	-	11	0.000200073	-0.016903532	-	-	-	11	0.000245793	-0.016706894	-	-	-	-	-	-
chr22	36019252	36019632	*MB*	5	0.005670402	-0.001233801	5	0.006240795	-0.000830462	5	0.006718992	-0.001189318	-	-	-	5	0.004951745	-0.001282995	-	-	-	-	-	-
chr15	90734476	90735422	*SEMA4B*	4	6.28E-07	-0.025183655	4	0.000107428	-0.026080522	4	8.65E-07	-0.025013504	-	-	-	4	4.07E-07	-0.025547425	-	-	-	-	-	-
chr17	79167630	79167836	*CEP131*	3	0.000657272	0.010421495	-	-	-	3	0.00080144	0.010421599	-	-	-	3	0.00068929	0.010376027	-	-	-	-	-	-
chr15	65822313	65822992	*HACD3*	12	0.003562212	0.00176168	-	-	-	-	-	-	-	-	-	12	0.004914748	0.001440213	-	-	-	-	-	-
chr13	92050483	92051154	*GPC5*	11	0.005896745	-0.008453583	-	-	-	11	0.007054524	-0.00850496	-	-	-	11	0.007245101	-0.008504092	-	-	-	-	-	-
chr15	41777661	41778987	-	4	0.00301795	0.017336879	-	-	-	-	-	-	-	-	-	4	0.002762769	0.017667209	-	-	-	-	-	-
chr1	10601389	10601833	*PEX14*	3	0.000835585	0.004894551	-	-	-	3	0.00106623	0.004836231	-	-	-	3	0.000971301	0.005147702	-	-	-	-	-	-
chr13	24825184	24825973	*SPATA13*	6	0.004181504	-0.014934785	-	-	-	6	0.004990234	-0.014885208	-	-	-	6	0.004941132	-0.014859279	-	-	-	-	-	-
chr14	73704007	73704343	*PAPLN*	5	0.008949549	0.008757616	-	-	-	5	0.010524071	0.008612767	-	-	-	5	0.007253366	0.009106706	-	-	-	-	-	-
chr2	21265912	21268152	*APOB*	17	5.76E-17	0.026733677	-	-	-	17	9.90E-17	0.026719441	-	-	-	17	2.38E-16	0.02641135	-	-	-	-	-	-
chr16	85362963	85363209	-	4	1.89E-05	0.036293171	4	0.040313231	0.033271375	4	2.01E-05	0.03544748	4	0.017109815	0.086267463	4	2.36E-05	0.036149461	-	-	-	-	-	-
chr14	21904906	21906134	*CHD8*	11	0.001609326	-0.003695314	-	-	-	11	0.00181385	-0.003746641	-	-	-	11	0.001441933	-0.003796278	-	-	-	-	-	-
chr17	14205803	14208363	*HS3ST3B1*																					
chr8	144797929	144799376	*MAPK15*	14	1.48E-05	0.005294952	-	-	-	14	1.72E-05	0.005411901	-	-	-	14	1.37E-05	0.005264798	-	-	-	-	-	-
chr15	29862036	29862801	*FAM189A1*	5	0.002938502	-0.014241035	-	-	-	5	0.003724126	-0.014050791	-	-	-	5	0.004009495	-0.013709879	-	-	-	-	-	-
chr14	22938675	22939357	-	5	0.004381358	-0.022507492	-	-	-	5	0.005591193	-0.022268426	-	-	-	5	0.004594284	-0.022570869	-	-	-	-	-	-
chr2	45236267	45237431	*SIX2*	9	0.007221844	-0.001835958	-	-	-	9	0.009090837	-0.001823689	-	-	-	9	0.007963239	-0.001718142	-	-	-	-	-	-
chr15	42066071	42066822	*MAPKBP1*	10	0.00595158	0.000517211	-	-	-	10	0.007352695	0.000514377	-	-	-	10	0.00617917	0.000535813	-	-	-	-	-	-
chr1	112259992	112260326	-	3	0.009957212	0.008430497	-	-	-	3	0.011402109	0.008199784	-	-	-	3	0.011123908	0.008491601	-	-	-	-	-	-
chr20	36662217	36663290	*RPRD1B*																					
chr8	74887999	74888597	*ELOC*																					
chr17	19881268	19882222	*AKAP10*	11	7.71E-06	-0.008843135	-	-	-	11	9.07E-06	-0.00885734	-	-	-	-	-	-	-	-	-	-	-	-
chr8	93114951	93116100	*RUNX1T1*	12	1.99E-08	0.004089686	12	7.34E-05	0.002267482	12	2.60E-08	0.004059857	-	-	-	12	1.07E-08	0.004479744	-	-	-	-	-	-
chr5	176078832	176080248	*TSPAN17*	3	0.001275889	0.001618539	-	-	-	3	0.001460084	0.001690424	-	-	-	3	0.001485349	0.001418593	-	-	-	-	-	-
chr13	113199311	113200144	*TUBGCP3*	5	0.005334746	0.009444131	-	-	-	-	-	-	-	-	-	-	-	-	-	-	-	-	-	-
chr2	113992694	113994035	*PAX8*																					
chr5	601475	602552	*LOC100996325*	7	0.000420039	0.018322595	-	-	-	7	0.000556977	0.01820902	-	-	-	7	0.000591778	0.018289983	-	-	-	-	-	-
chr17	79010968	79012396	*BAIAP2*																					
chr5	320888	321681	*AHRR*	3	7.87E-05	0.018014512	-	-	-	3	8.37E-05	0.018238844	3	0.005888475	0.0403383	3	4.07E-05	0.018767297	-	-	-	-	-	-
chr20	57425515	57429025	*GNAS*																					
chr6	32140544	32141146	*AGPAT1*	4	0.004095781	0.006313771	-	-	-	4	0.004716442	0.006149966	-	-	-	4	0.005263385	0.006386205	-	-	-	-	-	-
chr1	41131751	41132022	*RIMS3*	3	0.00439299	-0.00469085	-	-	-	3	0.004977877	-0.004469282	-	-	-	3	0.003588261	-0.004281957	-	-	-	-	-	-
chr8	27468166	27469673	*CLU*	10	0.003362391	-0.006901208	-	-	-	-	-	-	-	-	-	-	-	-	-	-	-	-	-	-
chr4	187064964	187066505	*FAM149A*	16	0.009152231	-0.00578427	-	-	-	-	-	-	-	-	-	-	-	-	-	-	-	-	-	-
chr16	19589790	19590554	*VPS35L*	5	0.00011585	-0.00964568	-	-	-	5	0.000145462	-0.009605068	-	-	-	5	9.29E-05	-0.009671296	5	0.01031031	-0.013243034	-	-	-
chr7	101603298	101603359	*CUX1*	3	0.004749807	-0.016781351	-	-	-	3	0.002493003	-0.017101917	-	-	-	3	0.00446321	-0.016647305	-	-	-	-	-	-
chr17	79952142	79952487	*ASPSCR1*	3	0.008441867	0.013776868	-	-	-	3	0.010551482	0.013700695	-	-	-	3	0.010677319	0.013725342	-	-	-	-	-	-
chr1	92946700	92947961	*GFI1*	6	7.03E-08	0.034577075	-	-	-	6	9.79E-08	0.034123184	6	0.001416486	0.112164131	6	4.06E-08	0.03499066	-	-	-	-	-	-
chr2	99438903	99439997	*CRACDL*	5	0.00188014	0.014629671	-	-	-	-	-	-	-	-	-	5	0.000875655	0.015423653	-	-	-	-	-	-
chr5	1489818	1489889	*LPCAT1*	3	0.002111462	0.021009135	-	-	-	3	0.002328741	0.021116042	-	-	-	3	0.003259176	0.020460029	-	-	-	-	-	-
chr4	99064102	99064904	*STPG2*	9	0.002157865	0.01549373	-	-	-	9	0.003154551	0.01510028	-	-	-	9	0.00386343	0.014888095	-	-	-	-	-	-

Supplemental Table 7 - Lifestyle specific Differentially Methylated Regions (DMRs) after smoking adjustment

Chromosom	Start	End	Number CpGs	Min smoothed FDR	Max difference	Mean difference	UCSC RefGene name
chr6	30038254	30039801	32	1.48E-12	0.26301652	0.101195725	*RNF39*
chr6	32134461	32135326	28	0.011804642	0.135533373	0.032421836	*EGFL8;PPT2-EGFL8*
chr20	57426264	57427103	28	0.018207343	-0.115194403	-0.030073591	*GNAS;GNAS-AS1*
chr12	133065724	133066762	27	0.00896172	-0.136345843	-0.035270375	*FBRSL1*
chr6	28863910	28864787	27	0.019442551	-0.052088872	-0.014174769	*NA*
chr10	135050619	135052004	24	0.015354912	0.111711963	0.039816501	*VENTX*
chr6	28890322	28891121	24	0.028139573	-0.048208715	0.00178324	*TRIM27*
chr17	46679361	46683120	23	1.84E-07	-0.20272025	-0.117526339	*HOXB-AS3;HOXB6*
chr11	2721610	2722713	22	0.005101391	-0.136214077	-0.018553555	*KCNQ1;KCNQ1OT1*
chr7	95025514	95026672	21	0.003688009	-0.145534461	-0.043021568	*PON1;PON3*
chr6	31854757	31855914	20	0.017286655	-0.099036495	0.000225499	*EHMT2*
chr12	47472317	47474055	20	0.033287493	-0.063795483	-0.017388384	*AMIGO2;PCED1B*
chr6	32016100	32016690	18	0.021795963	-0.097851478	-0.034476894	*TNXB*
chr7	86781154	86782128	17	0.004331321	-0.078566984	-0.005844292	*DMTF1*
chr4	1004609	1005405	17	0.009441084	0.091996236	-0.010027755	*FGFRL1*
chr19	42348327	42349472	17	0.011483672	0.177121094	0.041453065	*DMRTC2;LYPD4*
chr17	6543784	6544903	17	0.013600728	-0.049809311	-0.008881663	*KIAA0753;TXNDC17*
chr3	186524196	186525224	17	0.013910837	-0.062590463	-0.012125617	*RFC4*
chr2	27434127	27435253	17	0.015617619	-0.085943385	-0.025127007	*ATRAID;SLC5A6*
chr10	25463757	25464719	16	0.001821993	0.076189181	0.030642284	*GPR158;GPR158-AS1*
chr22	38141419	38143168	15	0.000173663	-0.109242447	-0.027101608	*TRIOBP*
chr5	135415258	135416613	15	0.002724331	-0.259045721	-0.17280631	*VTRNA2-1*
chr9	131084212	131085041	15	0.005481906	0.054454204	-0.002562696	*COQ4;TRUB2*
chr16	82659960	82660873	15	0.0068722	0.147339978	0.02169774	*CDH13*
chr1	27709634	27710270	15	0.010775673	-0.132045484	-0.035718048	*CD164L2*
chr8	144653962	144655679	14	4.29E-12	-0.170012208	-0.048003983	*MROH6*
chr6	142621875	142623767	14	0.0015424	0.14468528	0.066123508	*ADGRG6*
chr10	63808314	63809170	14	0.001707112	-0.079018856	-0.0486046	*ARID5B*
chr18	3450921	3451750	14	0.004196944	-0.03711323	-0.006142553	*TGIF1*
chr14	23298687	23299469	14	0.037205795	0.070843983	0.001816313	*MRPL52*
chr12	47219626	47220197	13	2.27E-05	-0.243884576	-0.121496601	*SLC38A4*
chr1	59042745	59044110	13	0.000190224	-0.366686331	-0.112687749	*TACSTD2*
chr8	144659831	144661051	13	0.000902352	-0.172569917	-0.086241001	*MROH6;NAPRT*
chr2	198649443	198650603	13	0.000923399	0.111795599	0.066728329	*BOLL*

chr14	33403060	33404466	13	0.001707112	0.108578924	0.032048617	*NPAS3*
chr17	8065842	8067323	13	0.001796119	-0.171974667	-0.058444662	*VAMP2*
chr2	21266500	21267334	13	0.001933556	0.163174972	0.08376975	*APOB*
chr10	22604699	22605708	13	0.008434456	-0.060620269	-0.020742989	*3MI1;COMMD3;COMMD3-BMI1*
chr18	11908329	11909634	13	0.008850963	0.095565042	-0.008341645	*MPPE1*
chr11	62495049	62495981	13	0.009990775	0.053981081	0.001539719	*RNPUL2;HNRNPUL2-BSCL2;TTC9C*
chr6	143998869	143999715	13	0.013896562	-0.102846483	0.017201106	*PHACTR2*
chr19	55994506	55996566	13	0.015558243	0.093634347	0.012465281	*NAT14;SSC5D;ZNF628*
chr14	74317981	74318586	13	0.015713405	0.082251142	0.02625064	*PTGR2;ZNF410*
chr13	31505976	31507139	13	0.018249798	-0.13977654	-0.064390807	*TEX26;TEX26-AS1*
chr4	87515184	87515704	13	0.020334008	0.14067437	0.015465099	*MAPK10;PTPN13*
chr5	133561934	133562604	13	0.020787079	-0.086709733	-0.018200012	*CDKL3;PPP2CA*
chr12	48152355	48152951	13	0.025514214	0.091642192	0.004969382	*RAPGEF3*
chr5	132201840	132202607	13	0.02851886	-0.075294802	-0.007129017	*GDF9;UQCRQ*
chr14	24898818	24899306	13	0.038139446	-0.073873007	-0.01324127	*CBLN3;KHNYN*
chr1	247275326	247276552	13	0.038876963	0.09995177	0.001564908	*C1orf229*
chr14	104551481	104552397	12	0.003378588	-0.114166856	-0.049895178	*ASPG*
chr19	33622563	33623230	12	0.006066932	0.102073377	0.057204463	*WDR88*
chr15	75018774	75019376	12	0.006305067	0.209134372	0.082134572	*CYP1A1*
chr11	2017939	2018724	12	0.008850963	0.112997602	0.050331891	*H19;MIR675*
chr15	73925257	73926085	12	0.009141693	-0.099204931	-0.026888671	*NPTN*
chr17	26971809	26972805	12	0.009756918	-0.064198316	-0.021010878	*KIAA0100*
chr20	33999761	34000519	12	0.009952802	-0.162313354	-0.059114786	*GDF5;UQCC1*
chr10	82115885	82116608	12	0.015139172	0.084435685	-0.007453005	*DYDC1;DYDC2*
chr6	29600108	29600642	12	0.021774764	-0.102675431	-0.025608405	*GABBR1*
chr7	75677153	75677766	12	0.026635647	-0.046409798	-0.00549369	*MDH2;STYXL1*
chr16	67203406	67204807	12	0.028503168	-0.070655549	-0.025724664	*HSF4;NOL3*
chr6	167412518	167413049	12	0.028503168	-0.047998634	-0.02194048	*CCR6;CEP43;MIR3939*
chr18	21718735	21719352	12	0.031406099	-0.100019923	-0.0266002	*CABYR*
chr2	55459394	55459861	12	0.038706554	-0.055183402	0.000640702	*CLHC1;RPS27A*
chr3	149375789	149376317	12	0.041485731	-0.07138728	-0.006345137	*WWTR1;WWTR1-AS1*
chr17	17942359	17942860	12	0.043121913	-0.057355415	-0.009696483	*ATPAF2;GID4*
chr11	2398327	2398587	12	0.045182833	-0.083928559	-0.027174465	*CD81*
chr6	28601271	28601519	11	2.86E-05	0.254357604	0.157070528	*NA*

chr1	55180710	55181852	11	0.002932471	0.077539821	-3.37E-05	*MROH7-TTC4;TTC4*
chr9	6412836	6413591	11	0.00955437	-0.061795266	-0.004635054	*UHRF2*
chr1	120254177	120255104	11	0.011702783	0.053260704	0.010795672	*PHGDH*
chr1	1356442	1357361	11	0.015873226	0.098316524	-0.010499811	*ANKRD65;TMEM88B*
chr4	78978537	78979207	11	0.015965145	-0.05766692	0.004153634	*FRAS1*
chr6	111136363	111136979	11	0.017844614	-0.099311799	-0.028952637	*AMD1;CDK19*
chr3	35720482	35721284	11	0.018569178	-0.180130237	-0.031102335	*ARPP21*
chr18	48085453	48086416	11	0.021087391	0.08943874	-0.000958752	*MAPK4*
chr11	57434942	57435627	11	0.021120397	-0.070473237	-0.013518406	*ZDHHC5*
chr3	126242808	126243401	11	0.021287941	0.08803036	0.022313838	*CHST13*
chr19	38396943	38397769	11	0.022808182	-0.070288937	-0.002966208	*SIPA1L3;WDR87*
chr11	67120966	67121634	11	0.023126666	-0.048066771	-0.01252725	*LOC100130987;POLD4*
chr6	30483837	30484429	11	0.024512983	0.039246155	0.002292975	*NA*
chr4	25235280	25235765	11	0.031661094	0.091701542	0.001092065	*PI4K2B*
chr1	1850733	1851165	11	0.033109138	0.057473738	-0.004041445	*TMEM52*
chr6	30294441	30294980	11	0.033389391	-0.104449827	-0.024241452	[illegible]*G17;HCG18;TRIM39;TRIM39-RPP21*
chr6	32975702	32976131	11	0.035004938	0.101091499	0.037900362	*HLA-DOA*
chr3	43731886	43732329	11	0.044622546	-0.052645856	-0.010326288	*ABHD5*
chr7	19156902	19157739	10	5.62E-05	0.090539765	0.016232795	*TWIST1*
chr7	123672780	123673926	10	0.000926147	0.072183541	0.027866358	*TMEM229A*
chr7	45960243	45961126	10	0.00351408	0.09706264	0.026614423	*IGFBP3*
chr6	34433139	34433789	10	0.004237731	0.097806496	0.023051143	*PACSIN1*
chr12	119419106	119419786	10	0.004796315	0.082392967	0.021206573	*SRRM4*
chr1	113051315	113052167	10	0.008985777	0.049679554	-0.003342945	*WNT2B*
chr13	27334723	27335542	10	0.009952802	-0.066917678	-0.007038999	*GPR12*
chr8	74206178	74206966	10	0.010934322	-0.109073169	-0.029651936	*RDH10;RPL7*
chr11	6947552	6948211	10	0.012585344	-0.078745837	-0.014774358	*ZNF215*
chr15	43476923	43477701	10	0.015712425	-0.094750832	-0.017182787	*CCNDBP1;TMEM62*
chr10	43892412	43893073	10	0.01623815	-0.099142707	-0.040102676	*HNRNPF*
chr13	98794353	98795021	10	0.02157135	-0.065115039	-0.021924104	*FARP1*
chr11	110167556	110167871	10	0.025514214	-0.069428206	-0.018568867	*RDX*
chr15	83952309	83953068	10	0.025612292	-0.067226892	-0.017991285	*BNC1*
chr4	171010728	171011502	10	0.028608668	0.056658159	0.020890815	*AADAT*
chr21	34443443	34444245	10	0.03054632	0.076630332	0.014907633	*OLIG1*

chr5	114937648	114938277	10	0.030584774	0.0724346	0.041821949	*TICAM2;TMED7-TICAM2*
chr16	30124803	30125403	10	0.032424872	0.07287271	-0.003825376	*BOLA2;GDPD3*
chr4	7044595	7044990	10	0.036046772	-0.054010136	-0.024867009	*CDC96;LOC100129931;TADA2B*
chr6	33160604	33161107	10	0.036502674	0.06941348	-0.009643855	*COL11A2*
chr11	61129344	61129696	10	0.039639971	-0.08923759	-0.01801855	*CYB561A3;TMEM138*
chr8	101224915	101225902	9	4.91E-06	-0.172220748	-0.10332275	*SPAG1*
chr6	31238388	31239411	9	5.10E-05	0.308505733	0.09685245	*HLA-B;HLA-C*
chr12	104608583	104609980	9	0.002307101	-0.062680604	-0.012487484	*TXNRD1*
chr11	94134015	94134756	9	0.005789294	0.076191152	0.021323954	*GPR83*
chr6	28979210	28979498	9	0.009302724	0.137506691	0.029835735	*NA*
chr6	31587470	31588053	9	0.0098945	-0.106398279	-0.011865661	*PRRC2A;SNORA38*
chr22	17488875	17489367	9	0.011264759	0.154522481	0.068590701	*GAB4*
chr3	113160490	113161177	9	0.012876563	0.111240084	0.072405584	*CFAP44*
chr7	100171610	100172801	9	0.013805048	0.109651623	0.056723712	*LRCH4;SAP25*
chr7	95401695	95402528	9	0.016522733	0.093825475	0.002086164	*DYNC1I1*
chr12	130821962	130822674	9	0.017165502	0.216725374	0.131503087	*PIWIL1*
chr1	242011104	242011587	9	0.020631027	-0.045996923	-0.002199165	*EXO1*
chr20	39765363	39765965	9	0.023163098	0.035628935	-0.005564896	*PLCG1*
chr14	71276348	71277192	9	0.029182312	-0.103872823	-0.046029856	*MAP3K9*
chr7	11012871	11013450	9	0.031262096	0.094839711	-0.012659232	*PHF14*
chr20	19866743	19867423	9	0.033183289	-0.073500786	-0.003342623	*RIN2*
chr17	46507705	46508105	9	0.033911991	-0.066215853	-0.030013996	*SKAP1*
chr8	74791016	74791574	9	0.037071973	0.040484535	0.003297309	*UBE2W*
chr6	31869518	31869588	9	0.041659012	0.028521283	-0.0107471	*C2;EHMT2;ZBTB12*
chr1	27114317	27114614	9	0.046948015	-0.100967558	-0.042147058	*PIGV*
chr1	159869902	159870326	8	0.001707112	-0.118826456	-0.05231167	*CFAP45*
chr4	146401747	146402992	8	0.004019554	0.104284553	0.013129813	*SMAD1*
chr2	731073	731594	8	0.004196944	0.278170015	0.209350176	*NA*
chr16	3155859	3156779	8	0.006442768	-0.135976093	-0.019294934	*NA*
chr16	49888100	49889831	8	0.010775673	-0.091088029	-0.021390446	*ZNF423*
chr2	231728721	231729329	8	0.014793022	-0.171368827	-0.060842406	*ITM2C*
chr22	43040446	43041232	8	0.017844614	-0.119540479	-0.049693223	*ATP5MGL;CYB5R3*
chr4	154387421	154388378	8	0.018708085	-0.061215416	-0.027982417	*TMEM131L*
chr20	17206529	17206999	8	0.019247515	0.062946951	0.019893576	*PCSK2*

chr18	51884797	51885214	8	0.024222294	-0.082634984	-0.020073707	*C18orf54;STARD6*
chr15	90319155	90319680	8	0.02647946	0.076412592	0.036178563	*MESP2*
chr14	21152452	21152897	8	0.030847998	-0.032396742	0.00617328	*ANG;RNASE4*
chr8	810529	811351	8	0.031613936	-0.082368081	-0.005259187	*NA*
chr2	241497412	241497727	8	0.033183289	-0.192827962	-0.080325561	*ANKMY1;DUSP28*
chr19	50879393	50879698	8	0.035555939	0.053633483	0.003563446	*NR1H2*
chr17	35767072	35767582	8	0.036508753	-0.059482547	-0.00810474	*ACACA;TADA2A*
chr17	53828263	53828610	8	0.036682975	-0.058499237	-0.033279795	*PCTP*
chr5	43483681	43484146	8	0.037071973	-0.066316387	-0.006491104	*TMEM267*
chr19	663093	663602	8	0.03763049	-0.056848611	-0.031823062	*RNF126*
chr2	74375128	74375507	8	0.042494977	0.086600113	-0.012003317	*BOLA3;BOLA3-AS1*
chr3	12837899	12838266	8	0.043842809	0.035396297	0.009083647	*CAND2*
chr5	42423228	42424075	7	7.77E-05	0.094547178	0.030842479	*GHR*
chr1	161049147	161050227	7	0.000624399	0.210309215	0.067863023	*NECTIN4*
chr19	10712866	10713543	7	0.001175357	0.111891561	0.038247547	*SLC44A2*
chr19	1465962	1467032	7	0.001176746	-0.180770992	-0.099097318	*APC2;C19orf25*
chr4	1496334	1497223	7	0.005787251	-0.130725142	0.00334205	*NA*
chr19	39402823	39403373	7	0.010661068	-0.13675817	-0.074851436	*CCER2*
chr8	95274566	95274933	7	0.010808047	-0.118547535	-0.026024397	*GEM*
chr12	6976655	6977392	7	0.013138205	-0.03633027	-0.014748903	*TPI1*
chr7	16505094	16505664	7	0.015364516	-0.172379547	-0.10633277	*SOSTDC1*
chr16	31119067	31119650	7	0.018319418	-0.159229681	-0.044900986	*BCKDK*
chr4	122854168	122854532	7	0.01861181	-0.191767291	-0.044647897	*TRPC3*
chr1	3774827	3775206	7	0.019129526	-0.130708007	-0.082782716	*CEP104;DFFB*
chr19	42894425	42894906	7	0.020127952	-0.109667812	-0.058800536	*CNFN*
chr18	21977798	21978109	7	0.020614918	-0.096043681	-0.028976106	*OSBPL1A*
chr2	101223069	101223620	7	0.021120397	-0.114685953	-0.058700076	*NA*
chr9	108005984	108006799	7	0.022844274	0.109088342	-0.015263414	*SLC44A1*
chr17	40428024	40428759	7	0.027378875	-0.086317359	-0.054441294	*STAT5B*
chr20	11871282	11871743	7	0.028373534	0.168045916	0.070976842	*BTBD3*
chr11	107729641	107730039	7	0.031798627	-0.066994979	-0.017970957	*SLC35F2*
chr2	144694581	144695257	7	0.03217355	-0.051798085	-0.011130018	*NA*
chr11	126225276	126225951	7	0.032216587	-0.075056175	-0.017910727	*GSEC;ST3GAL4*
chr6	124124875	124125371	7	0.033183289	-0.061951499	-0.013263551	*NKAIN2*

chr5	111092139	111093469	7	0.033295077	0.083558587	-0.01052088	*NREP*
chr8	120428418	120428898	7	0.034815718	-0.058296848	-0.007742598	*CCN3*
chr20	35807377	35807784	7	0.03745477	-0.061370229	-0.017688034	*MROH8;RPN2*
chr4	83719895	83720102	7	0.042923074	-0.109267576	-0.006166339	*SCD5*
chr14	102771505	102771717	7	0.043746698	-0.090091205	-0.011626841	*MOK*
chr7	92219279	92219567	7	0.045090688	-0.062780979	-0.016675099	*FAM133B*
chr8	55533655	55534103	7	0.046319335	0.113555013	0.053733301	*RP1*
chr11	626729	627612	6	4.94E-05	0.127867502	0.061457867	*CDHR5;SCT*
chr17	79503292	79504180	6	0.001796119	-0.24227794	-0.082873607	*FSCN2*
chr10	34061313	34062018	6	0.002002334	-0.142265158	-0.092242859	*LINC00838*
chr13	19918525	19919394	6	0.003205348	0.202793383	0.106115539	*ANKRD26P3;LINC00421*
chr16	69154611	69155483	6	0.005741612	0.101070672	0.02097762	*CHTF8*
chr10	15210662	15211442	6	0.007996067	-0.131241521	-0.061463584	*NMT2*
chr3	173113110	173113910	6	0.008940976	0.054709444	0.0210243	*NLGN1*
chr5	112770203	112770721	6	0.008945805	-0.066228884	-0.018254742	*MCC;TSSK1B*
chr7	4456202	4456592	6	0.010494913	0.182850555	0.114367948	*NA*
chr2	121301692	121302460	6	0.010757056	-0.136051961	-0.053202384	*NA*
chr15	39871808	39872186	6	0.010775673	-0.226183866	-0.139496903	*THBS1*
chr1	40105664	40106248	6	0.012484492	0.120222411	0.038991082	*HEYL*
chr7	43797547	43798207	6	0.013937261	-0.081812312	-0.036936732	*BLVRA*
chr7	158059704	158060136	6	0.015225012	0.098852582	0.057342987	*PTPRN2*
chr7	87257767	87258443	6	0.015712425	0.094253478	0.019687971	*ABCB1;RUNDC3B*
chr4	2060934	2061512	6	0.017165502	-0.03294622	0.006149591	*NAT8L*
chr3	158450208	158450833	6	0.017346598	0.106590183	0.059150488	*RARRES1*
chr1	40098595	40099015	6	0.018295584	-0.169632011	-0.090756521	*HEYL*
chr12	109162390	109162748	6	0.019008503	0.077690511	0.019288393	*NA*
chr1	228657536	228658646	6	0.020687083	0.160739709	0.087891002	*NA*
chr3	185080816	185081403	6	0.020777476	-0.044487008	-0.007359844	*MAP3K13*
chr3	195622281	195622940	6	0.021795963	-0.062628141	-0.03194915	*TNK2*
chr17	1613239	1613880	6	0.024209174	0.046033889	0.016804802	*TLCD2*
chr6	111303269	111303792	6	0.02783686	-0.086061107	-0.015599693	*RPF2*
chr6	111195301	111195812	6	0.029360815	-0.081797585	-0.017730802	*AMD1*
chr13	110521956	110522572	6	0.030341948	0.257310941	0.134976998	*NA*
chr16	1495040	1495835	6	0.031262096	0.112592576	0.071655672	*CCDC154;CLCN7*

chr18	59992252	59992517	6	0.03217355	-0.053439806	-0.009230285	*TNFRSF11A*
chr12	54359712	54360262	6	0.032559751	0.095181277	-0.005147779	*HOTAIR*
chr19	30303131	30303674	6	0.032993029	-0.045795357	-0.03381184	*CCNE1*
chr17	46659751	46660002	6	0.033183289	0.09274303	0.044423817	*HOXB3;HOXB4;MIR10A*
chr17	1958851	1959132	6	0.033617505	-0.064722785	-0.028806801	*HIC1*
chr7	105264417	105264836	6	0.035203428	0.083246217	0.027758094	*ATXN7L1*
chr1	111743200	111743537	6	0.036205443	-0.074810336	-0.047639161	*DENND2D*
chr9	116102304	116102562	6	0.036343346	-0.09243328	-0.033977341	*WDR31*
chr6	33378502	33379050	6	0.036502674	-0.064512876	-0.024446075	*PHF1*
chr15	57210395	57210921	6	0.037090817	0.039321129	-0.001436146	*LOC145783;TCF12;ZNF280D*
chr6	110300099	110300308	6	0.038819353	0.089155299	0.048124021	*GPR6*
chr20	62439117	62439544	6	0.038930097	0.088135791	0.057128324	*ZBTB46*
chr3	71633196	71633604	6	0.039490364	0.050381725	0.015612789	*FOXP1*
chr7	94138547	94138947	6	0.041182301	-0.036324952	-0.006218044	*CASD1*
chr6	105584691	105585184	6	0.041195903	0.054965288	0.013809348	*BVES;BVES-AS1*
chr1	109585022	109585487	6	0.042367687	0.061432481	0.010113494	*WDR47*
chr8	59572451	59572638	6	0.042923074	0.026313188	0.003755562	*NSMAF*
chr13	78272372	78272443	6	0.043012622	-0.035322594	-0.021968589	*MIR3665;SLAIN1*
chr7	123389157	123389366	6	0.044434161	-0.071502893	-0.028045936	*WASL*
chr2	85811236	85811471	6	0.045879721	-0.062942059	-0.040838129	*VAMP5*
chr6	44310005	44310331	6	0.047115557	-0.060670247	-0.004155439	*SPATS1*
chr17	55173075	55173766	5	0.000508871	-0.111234013	-0.017476135	*AKAP1*
chr2	185463209	185463674	5	0.001742829	0.068821733	0.024308036	*ZNF804A*
chr16	15488936	15489794	5	0.004107446	0.084063346	0.037941491	*MPV17L*
chr1	246859889	246860416	5	0.007371134	0.209774278	0.101812804	*NA*
chr17	35656660	35657046	5	0.008685202	-0.119575976	-0.069619925	*ACACA*
chr16	88821234	88821905	5	0.00966866	-0.151580883	0.010657882	*PIEZO1*
chr11	64146487	64146871	5	0.010775673	-0.100085576	-0.046382962	*NA*
chr19	46801193	46801672	5	0.010991402	-0.177158599	-0.111982635	*HIF3A;RNU6-66P*
chr19	49176277	49176399	5	0.012759079	-0.081218805	-0.050998253	*NTN5;SEC1P*
chr8	144222015	144222455	5	0.013498256	-0.164914748	-0.122093527	*NA*
chr13	111521981	111522651	5	0.013875455	-0.118603002	-0.100180248	*PRECSIT*
chr22	23605330	23605797	5	0.014180864	-0.096450389	0.023792074	*BCR;FBXW4P1*
chr16	89114613	89114919	5	0.017844614	-0.101490115	-0.088256412	*NA*

chr15	99408958	99409506	5	0.018358131	-0.138217802	-0.105134899	*IGF1R*
chr1	209798721	209799353	5	0.018634554	0.076163497	0.040836585	*LAMB3;MIR4260*
chr18	9101864	9102421	5	0.018728525	0.028194066	0.006420702	*NDUFV2*
chr6	7142024	7142638	5	0.021336037	0.108040666	0.06717488	*RREB1*
chr8	106330881	106331317	5	0.021936409	0.110986467	0.0368343	*ZFPM2*
chr12	52346851	52347184	5	0.023781168	-0.107233405	-0.064031967	*ACVR1B*
chr17	14207530	14207968	5	0.024512983	0.102073203	0.065144126	*HS3ST3B1;MGC12916*
chr14	105154557	105154994	5	0.026676169	-0.07816756	-0.049710645	*INF2*
chr1	202778443	202778869	5	0.027271834	0.177972258	0.091092009	*KDM5B;PCAT6*
chr6	157931791	157932180	5	0.027920357	0.133915992	0.072288308	*ZDHHC14*
chr7	102937535	102937788	5	0.028213508	-0.087045186	-0.016756116	*PMPCB*
chr16	5008216	5008608	5	0.028540655	0.09777836	-0.029406353	*SEC14L5*
chr17	73824354	73824897	5	0.030772274	-0.151021044	-0.07842078	*UNC13D*
chr3	87039900	87040452	5	0.031053025	0.0486657	0.028772879	*VGLL3*
chr3	118865365	118865927	5	0.031798627	-0.066569202	-0.002948711	*IGSF11;TEX55*
chr11	43333502	43333988	5	0.031958419	0.031369706	-0.003442522	*API5*
chr10	105428385	105428818	5	0.032156601	-0.123385458	-0.088987174	*SH3PXD2A*
chr4	1322681	1323433	5	0.032194122	0.110628313	0.057803891	*MAEA*
chr12	132269563	132269737	5	0.032993029	0.0937075	0.03523455	*SFSWAP*
chr6	32187918	32188404	5	0.033183289	0.076148398	-0.019737207	*NOTCH4*
chr6	170337747	170338591	5	0.0335179	0.122554543	0.078656319	*NA*
chr1	805102	805746	5	0.034399618	-0.116311535	-0.015339412	*FAM41C*
chr7	158741284	158741817	5	0.035208803	-0.191507887	0.004247693	*NA*
chr12	56236845	56237315	5	0.035936273	0.12160083	0.021249624	*MMP19*
chr5	63461566	63461930	5	0.036202459	0.178244781	0.07584025	*RNF180*
chr16	67966314	67966621	5	0.036682975	0.125530833	0.024963174	*CTRL;PSMB10*
chr19	10823679	10823913	5	0.037205795	0.077925383	0.054120536	*DNM2;QTRT1*
chr5	178368071	178368185	5	0.038282955	0.065546581	0.036944951	*ZNF454*
chr8	4852424	4852654	5	0.039311212	0.074501133	0.034213467	*CSMD1*
chr11	19262474	19262624	5	0.039826819	-0.026156169	-0.005999187	*E2F8*
chr6	31895240	31895598	5	0.040739387	-0.083760309	-0.049591313	*C2;CFB*
chr17	71160864	71161178	5	0.041076	0.106542376	-0.002684612	*SSTR2*
chr3	140770013	140770345	5	0.041076	0.044174232	0.012101214	*SPSB4*
chr11	125366499	125367112	5	0.041650715	-0.080117922	-0.015395577	*FEZ1*

chr3	129147230	129147577	5	0.042133543	0.133986494	0.056296258	*EFCAB12*
chr19	6333547	6333701	5	0.043608533	-0.076133339	-0.027987433	*ACER1*
chr7	135194977	135195151	5	0.044900253	0.035515681	0.00265417	*CNOT4*
chr5	6633481	6633577	5	0.045729919	-0.052139982	-0.006448026	*NSUN2;SRD5A1*
chr15	74466794	74467158	5	0.045746992	0.167323881	0.080038638	*ISLR*
chr19	57630446	57630596	5	0.047115557	0.102696399	0.07937893	*USP29*
chr19	12876846	12877188	4	2.96E-05	-0.613005342	-0.49721534	*HOOK2*
chr12	125465188	125465696	4	0.001821993	0.101947027	0.041933572	*DHX37*
chr5	145215445	145215784	4	0.002307101	-0.106678072	-0.069131916	*PRELID2*
chr6	30525527	30526487	4	0.003028065	-0.083116079	-0.03498628	*GNL1;PRR3*
chr18	72916012	72916776	4	0.004072894	0.214897568	0.157737259	*ZADH2*
chr4	184243582	184243754	4	0.004107446	-0.068361824	-0.041951332	*CLDN22;CLDN24*
chr2	183387885	183388632	4	0.004930342	-0.153479383	-0.0819455	*PDE1A*
chr7	2124459	2125029	4	0.005462329	0.107986736	0.067972251	*MAD1L1*
chr1	58716532	58717107	4	0.005744929	0.128776329	0.028764476	*DAB1*
chr12	2031262	2031922	4	0.005789294	0.255029956	0.13053044	*CACNA2D4*
chr21	16436121	16436497	4	0.010777842	0.037359403	0.018537103	*NRIP1*
chr13	114053863	114054286	4	0.011315933	0.100846557	0.030155368	*NA*
chr12	86658268	86659008	4	0.011318983	0.194435373	0.098590925	*MGAT4C*
chr3	49170498	49170794	4	0.012378905	-0.194277805	-0.139046303	*LAMB2*
chr1	26490540	26491047	4	0.013805048	-0.111103722	-0.091513427	*NA*
chr7	1961785	1962389	4	0.013902841	0.171232219	0.083642807	*MAD1L1*
chr7	102715559	102716072	4	0.015364516	-0.038253225	-0.016803391	*ARMC10;FBXL13*
chr17	43508250	43508894	4	0.015712425	-0.10174792	-0.055466505	*ARHGAP27*
chr11	126294215	126294752	4	0.016862521	-0.084280222	-0.010371504	*KIRREL3;ST3GAL4*
chr1	159435469	159436015	4	0.017346327	-0.100515627	0.009585296	*NA*
chr7	100304043	100304693	4	0.017765518	0.071411563	-0.00323373	*POP7*
chr12	1753440	1754299	4	0.017852799	-0.147519269	-0.105024046	*WNT5B*
chr10	11485460	11485771	4	0.018207705	0.067743333	0.039098865	*NA*
chr16	87492138	87492801	4	0.020770083	0.0620845	0.016828856	*ZCCHC14*
chr14	24583056	24583689	4	0.02157135	-0.074686284	-0.015209254	*DCAF11;NRL*
chr5	307591	307899	4	0.022400932	-0.051077497	-0.030631722	*AHRR;PDCD6*
chr17	7233150	7234027	4	0.023201933	-0.157589383	-0.064895397	*NEURL4*
chr10	112836688	112837240	4	0.026836527	0.02931908	0.014555425	*ADRA2A*

chr9	127054095	127054510	4	0.027559543	0.07937333	0.009171466	*NEK6*
chr10	75911181	75911703	4	0.028373534	-0.059206656	-0.007912137	*ADK;AP3M1*
chr16	15702153	15702420	4	0.028438037	-0.115168064	-0.058874553	*MARF1*
chr19	40948296	40948697	4	0.029071873	0.037452821	0.016820711	*SERTAD3*
chr12	56638534	56638934	4	0.029182312	-0.111865663	-0.03824832	*ANKRD52*
chr16	50186775	50186996	4	0.029416592	-0.078441215	-0.050361612	*TENT4B*
chr16	31117067	31117318	4	0.030371363	0.076271896	0.023177095	*BCKDK*
chr15	91414797	91415532	4	0.030772274	0.119649514	0.02465318	*FURIN*
chr4	103266893	103267084	4	0.031613936	-0.047313965	-0.007770001	*SLC39A8*
chr19	12846438	12846841	4	0.031613936	-0.091238696	0.01678708	*GET3;TRIR*
chr1	92946700	92947332	4	0.033331197	-0.143583145	-0.094310337	*GFI1*
chr1	160313545	160314350	4	0.033389391	-0.049734414	-0.009588829	*COPA;NCSTN*
chr12	133417190	133417338	4	0.034494267	0.124188144	0.097224365	*CHFR*
chr21	45662262	45662813	4	0.035060658	0.061461321	-0.004259087	*ICOSLG*
chr19	54668230	54668565	4	0.035289159	-0.098408519	-0.049998916	*LENG1;TMC4*
chr2	239139911	239140182	4	0.036343346	-0.115202709	-0.089349127	*LINC02610;TARDBPP3*
chr19	49223814	49224033	4	0.036685234	-0.108860551	-0.085803428	*MAMSTR;RASIP1*
chr19	45457189	45457300	4	0.036869736	-0.053163624	-0.03417238	*CLPTM1*
chr13	114927579	114927854	4	0.038067836	-0.04846147	-0.029507056	*NA*
chr6	29895175	29895260	4	0.038346575	-0.27947107	-0.200954025	*HCG4B;HLA-G;HLA-H;HLA-J*
chr9	130742860	130743022	4	0.038706554	-0.074636514	-0.04388924	*FAM102A*
chr19	1815917	1816196	4	0.038790556	0.089400713	0.043502618	*ATP8B3;MIR1909;REXO1*
chr19	2588227	2588479	4	0.03887293	0.12101908	0.006464013	*GNG7*
chr13	21348012	21348215	4	0.03891868	0.089896473	0.010411149	*EEF1AKMT1*
chr16	30042330	30042541	4	0.039639971	-0.045665902	-0.026469998	*BOLA2;TLCD3B*
chr1	15763737	15764095	4	0.039731824	0.047722324	0.003555558	*CTRC*
chr3	113466193	113466425	4	0.040206122	-0.046826389	-0.034024179	*ATP6V1A;NAA50*
chr6	109787078	109787360	4	0.040379765	-0.060313711	-0.019149001	*MICAL1;ZBTB24*
chr14	100842311	100842590	4	0.042380333	-0.034745427	-0.015745906	*WARS1;WDR25*
chr7	5413872	5414174	4	0.042486297	0.104380585	0.086295672	*TNRC18*
chr18	55288998	55289231	4	0.042731413	-0.061594277	-0.029449876	*NARS1*
chr19	8454846	8454938	4	0.043608533	0.023140539	0.002050953	*MIR4999;RAB11B;RAB11B-AS1*
chr14	94640029	94640495	4	0.044900253	-0.070822956	-0.016024214	*PPP4R4*
chr22	30116319	30116489	4	0.045117929	-0.087550232	-0.014887881	*CABP7*

chr2	39351390	39351696	4	0.045726881	-0.032272667	0.002782603	*SOS1*
chr11	62066533	62066669	4	0.046005284	-0.131623578	-0.073396409	*SCGB1D4*
chr8	73449506	73449524	4	0.046319335	-0.061384166	-0.034573656	*KCNB2*
chr7	151574492	151574642	4	0.046888849	-0.050289678	-0.011719877	*PRKAG2;PRKAG2-AS1*
chr2	176995076	176995088	4	0.046922832	0.075277097	0.059609881	*HOXD8*
chr6	28497278	28497370	4	0.04718463	-0.122851728	-0.061701655	*GPX5*
chr5	6868355	6868585	4	0.047689373	0.12411153	0.008522964	*NA*
chr19	49220102	49220235	4	0.048132553	-0.085253927	-0.059800222	*MAMSTR*
chr2	102314462	102314495	4	0.048580985	-0.054230157	-0.013451885	*MAP4K4*
chr17	29649427	29650042	3	0.000607878	-0.115478141	-0.000843546	*EVI2A;NF1*
chr7	2143507	2143942	3	0.001160691	-0.130653602	-0.088760616	*MAD1L1*
chr20	61448534	61449152	3	0.001999273	-0.123871015	-0.057419568	*COL9A3*
chr19	13125726	13125988	3	0.00200662	-0.16356157	-0.089476076	*NFIX*
chr1	58898552	58898793	3	0.003205348	-0.248731165	-0.211267445	*DAB1*
chr7	1233192	1233469	3	0.003643757	-0.175779877	-0.104069623	*NA*
chr1	58525572	58525635	3	0.006305067	-0.139067934	-0.091193059	*DAB1*
chr19	23445741	23446001	3	0.007364431	0.080007874	0.071197034	*NA*
chr18	47429208	47429391	3	0.009952802	0.10640461	0.005862874	*MYO5B*
chr11	115266354	115266960	3	0.011804642	-0.088175312	-0.013929197	*CADM1*
chr20	47364640	47365070	3	0.012838218	0.207486184	0.149866637	*PREX1*
chr15	48253631	48254179	3	0.013220712	0.165488875	0.077579317	*NA*
chr19	44861370	44861706	3	0.013612977	0.141712304	0.107297182	*ZNF112*
chr18	24443262	24443344	3	0.015208609	0.065894759	0.045167163	*AQP4;AQP4-AS1*
chr17	48207508	48207662	3	0.01549907	0.033544198	0.002285202	*SAMD14*
chr6	151668632	151668841	3	0.015712425	-0.113604856	-0.072493817	*AKAP12*
chr11	70395310	70395513	3	0.015859785	-0.057264338	-0.031148783	*SHANK2*
chr12	117100401	117100900	3	0.017241849	-0.088924509	-0.068088066	*NA*
chr7	38903039	38903457	3	0.01728052	0.077174788	-0.015531632	*VPS41*
chr3	124777406	124777847	3	0.018317067	0.076257915	-0.003054701	*HEG1*
chr12	10183850	10184399	3	0.018634554	0.070712844	-0.011943016	*CLEC9A*
chr10	71243495	71243993	3	0.019094061	-0.076566247	-0.029848723	*TSPAN15*
chr2	9778653	9778812	3	0.019445134	-0.070669019	-0.034623412	*NA*
chr20	3063586	3064015	3	0.019923194	-0.038456271	-0.003726242	*AVP*
chr13	114886883	114887369	3	0.020172484	0.093726599	0.034423835	*RASA3*

chr17	78472016	78472290	3	0.02154484	-0.112367856	-0.096362986	*NA*
chr6	79943498	79943796	3	0.022468249	0.030377039	-0.000914654	*HMGN3;HMGN3-AS1*
chr19	19016467	19016789	3	0.023349981	0.093593286	0.053878263	*COPE*
chr12	132979486	132979749	3	0.024041264	-0.190276719	-0.064884185	*NA*
chr12	56754826	56755270	3	0.024926536	-0.116755209	-0.029446609	*APOF;STAT2*
chr9	99212660	99213506	3	0.025091416	0.022858556	0.010690206	*HABP4*
chr3	128590524	128590658	3	0.025602428	0.083291748	0.022482346	*LOC653712*
chr4	3322285	3322833	3	0.025797209	0.125221133	0.035317628	*RGS12*
chr15	34502580	34502993	3	0.027723753	-0.089733117	-0.034860922	*KATNBL1*
chr9	137519102	137519225	3	0.028095708	0.082546408	0.024354329	*NA*
chr7	157130291	157130686	3	0.02841223	-0.133562583	-0.091265657	*DNAJB6*
chr14	21483525	21483641	3	0.028620418	-0.087295261	-0.020627785	*NA*
chr6	45905338	45905524	3	0.029337458	-0.073179958	-0.039156022	*CLIC5*
chr16	616827	616935	3	0.03054632	-0.061808266	-0.04703937	*NHLRC4;PIGQ*
chr6	36515445	36515856	3	0.031661094	-0.067516981	-0.034501581	*STK38*
chr10	114712217	114712695	3	0.032464046	-0.044715479	-0.040463571	*TCF7L2*
chr12	53887752	53887964	3	0.032701752	0.067875469	0.057644945	*MAP3K12*
chr13	86373320	86373792	3	0.032996682	-0.088666048	-0.081677841	*SLITRK6*
chr12	20962210	20962423	3	0.033617505	-0.082132157	-0.026389446	*SLCO1B3*
chr12	101869976	101870079	3	0.035208803	0.190057228	0.033726114	*SPIC*
chr4	180980155	180980552	3	0.035208803	0.035465438	0.034542923	*NA*
chr13	114830713	114831142	3	0.035598923	-0.032487458	-3.12E-05	*RASA3*
chr13	103425389	103425546	3	0.035633936	-0.025919158	-0.013482388	*TEX30*
chr1	22378412	22378629	3	0.036273689	0.08491239	0.042432508	*CDC42*
chr13	53775035	53775410	3	0.036685234	0.119360605	0.054501328	*NA*
chr17	47296912	47297130	3	0.036685234	0.110592896	0.075231312	*ABI3*
chr17	60783129	60783200	3	0.037205795	-0.095984852	-0.011804001	*MARCHF10*
chr1	160160670	160160766	3	0.037274147	-0.072794864	-0.039422974	*CASQ1*
chr3	143692255	143692356	3	0.037687516	0.01791929	0.013161978	*DIPK2A*
chr17	74274956	74275137	3	0.038241185	0.070755635	0.0125242	*QRICH2*
chr10	126308381	126308552	3	0.03843711	-0.138563398	-0.076473217	*FAM53B*
chr14	102230231	102230722	3	0.03843711	-0.085615885	-0.075626588	*PPP2R5C*
chr19	58143734	58144245	3	0.038636358	0.119352345	0.063482587	*ZNF211*
chr17	4901392	4901584	3	0.038876963	-0.028893756	-0.005701415	*INCA1;KIF1C*

chr1	153589781	153590243	3	0.039028691	0.493781568	0.184059643	*S100A14;S100A16*
chr12	54347057	54347286	3	0.03982194	0.06159129	0.052733529	*HOXC12*
chr10	682449	682693	3	0.039987891	-0.107153054	-0.036906692	*DIP2C*
chr4	99917544	99917608	3	0.040196119	-0.079776075	-0.026283354	*METAP1;MIR3684*
chr3	43121422	43121668	3	0.040530154	0.071271704	0.049877835	*POMGNT2*
chr2	230787137	230787493	3	0.040762442	-0.043862649	-0.016638213	*FBXO36;TRIP12*
chr10	6472750	6472833	3	0.041171693	-0.070338085	-0.040887871	*PRKCQ*
chr17	27939261	27939327	3	0.041195903	0.057384342	0.002018358	*ANKRD13B*
chr7	93519220	93519473	3	0.041284085	0.070432762	0.014751347	*GNGT1;TFPI2*
chr17	77834118	77834299	3	0.041297389	-0.081522356	-0.013590732	*NA*
chr6	147828624	147828810	3	0.041329653	0.01881004	0.009182121	*SAMD5*
chr19	35306283	35306369	3	0.041521462	-0.054052665	0.002139337	*NA*
chr15	60690826	60690974	3	0.042012553	0.042273508	-0.00158534	*ANXA2*
chr4	38524849	38524875	3	0.042137903	-0.095100843	-0.065287402	*NA*
chr5	3736618	3736816	3	0.042184045	0.141003266	0.076945392	*NA*
chr16	49666012	49666504	3	0.042322181	0.128032585	0.093709838	*ZNF423*
chr7	1894300	1894688	3	0.042417701	0.113599211	0.066653701	*MAD1L1*
chr8	142310691	142311034	3	0.042494977	0.12170078	0.052673747	*NA*
chr12	10331894	10332199	3	0.043068273	0.10312795	0.081803516	*TMEM52B*
chr5	41968376	41968459	3	0.043608533	0.178475916	0.121705667	*NA*
chr12	656423	656757	3	0.043687895	-0.09655995	-0.044727502	*B4GALNT3*
chr3	189348936	189349021	3	0.04397688	-0.140939681	-0.117293266	*TP63*
chr15	69605276	69605418	3	0.044214906	-0.043317952	-0.032267657	*PAQR5*
chr1	45671987	45672074	3	0.044214906	-0.060476221	-0.027686252	*ZSWIM5*
chr8	142221164	142221243	3	0.044214906	0.077221351	0.035241952	*SLC45A4*
chr6	1615992	1616430	3	0.044548534	-0.145224074	-0.110172293	*NA*
chr20	20036632	20036684	3	0.044578385	-0.154860885	-0.100148811	*CFAP61;CRNKL1*
chr17	1465813	1466142	3	0.045227113	-0.054111956	-0.029164373	*PITPNA*
chr14	102484795	102484867	3	0.045227113	0.052919837	0.023933222	*DYNC1H1*
chr1	109969527	109969611	3	0.04534577	0.043761299	0.003102453	*PSMA5*
chr12	6473299	6473444	3	0.0458537	-0.092677396	-0.072324217	*SCNN1A*
chr5	134181742	134181967	3	0.04612551	-0.068855514	-0.055821058	*C5orf24*
chr11	1773018	1773219	3	0.046270631	-0.092302649	-0.077609294	*IFITM10;MOB2*
chr16	8963642	8963860	3	0.046313053	0.053857473	0.038819204	*CARHSP1*

chr19	5507274	5507344	3	0.046319335	0.077588733	0.063324737	*NA*
chr2	231085667	231085735	3	0.046948015	0.10096481	0.019884529	*SP110;SP140*
chr3	127872092	127872147	3	0.046961223	-0.033390996	-0.017303099	*EEFSEC;RUVBL1*
chr8	68644689	68644801	3	0.047277315	-0.085614515	-0.009617727	*CPA6*
chr18	43547530	43547701	3	0.047722262	-0.030553	-0.013626945	*EPG5*
chr17	76836799	76836835	3	0.04882849	-0.064314042	-0.029184464	*USP36*
chr12	62653704	62653866	3	0.048993504	0.009867213	0.001174514	*TAFA2;USP15*
chr5	139027535	139027722	3	0.049339429	-0.094095068	-0.026819562	*CXXC5*
chr9	130487227	130487287	3	0.049383583	-0.101120025	-0.088909339	*PTRH1;TTC16*

Supplemental Table 8 - All lifestyle specific Differentially Methylated Positions (DMPs)

Log fold change	Average Expression	t-value	*P* -value	Adj. *P* -value	B-value	Chromosom	Position	Strand	CpG-Name	UCSC RefGene name
0.193803612	0.681740677	9.33022951	3.34E-15	2.49E-09	23.6715178	chr5	373378	+	cg05575921	*AHRR*
**0.089458236**	**0.502052675**	**8.74263182**	**6.29E-14**	**2.34E-08**	**20.7513917**	**chr19**	**17000585**	**-**	**cg03636183**	***F2RL3***
0.062807746	0.693318329	8.30515271	5.52E-13	1.37E-07	18.5921592	chr19	16998668	+	cg21911711	*F2RL3*
0.083336995	0.62903076	7.8434826	5.36E-12	7.99E-07	16.3349804	chr2	233284661	-	cg21566642	-
0.07293225	0.467577109	7.86701589	4.78E-12	7.99E-07	16.4493822	chr2	233284934	-	cg01940273	-
0.082181848	0.281667406	7.78234353	7.24E-12	8.91E-07	16.0381351	chr11	86513429	+	cg14391737	*PRSS23*
**0.078959254**	**0.30069194**	**7.75257702**	**8.37E-12**	**8.91E-07**	**15.8938087**	**chr17**	**38477572**	**-**	**cg17739917**	***RARA***
0.041664418	0.686417226	6.66924222	1.51E-09	0.000141032	10.7517395	chr13	30694031	+	cg23508991	-
0.046269909	0.427775422	6.57601105	2.34E-09	0.000193907	10.3215448	chr11	86510915	+	cg11660018	*PRSS23*
0.043914978	0.409300335	6.30203529	8.33E-09	0.00062081	9.07134284	chr5	369969	+	cg01899089	*AHRR*
-0.030493525	0.387702544	-6.1894747	1.40E-08	0.000944988	8.56417525	chr5	169144438	-	cg04730794	*DOCK2*
0.031079071	0.421104216	6.15967141	1.60E-08	0.000992306	8.43055635	chr15	74724918	-	cg00310412	*SEMA7A*
-0.08590341	0.611707079	-5.9981559	3.32E-08	0.001904	7.71147126	chr15	90345999	-	cg06344992	*ANPEP*
0.03256167	0.69207816	5.66391498	1.47E-07	0.007827339	6.2523093	chr3	47054668	-	cg18704979	*LOC100129354*
-0.058856163	0.721651694	-5.6412621	1.62E-07	0.008069468	6.15491762	chr15	90346094	-	cg23432008	*ANPEP*
-0.026479401	0.829494564	-5.5078207	2.91E-07	0.012223739	5.58530531	chr4	17277892	+	cg09568924	-
-0.031123313	0.822418962	-5.5042503	2.95E-07	0.012223739	5.57016236	chr1	108581659	-	cg12754019	-
-0.076464321	0.588248951	-5.5231184	2.72E-07	0.012223739	5.65024464	chr15	90346089	-	cg02008229	*ANPEP*
0.057960459	0.560933206	5.47153733	3.40E-07	0.013342801	5.43166212	chr14	77248049	+	cg10387007	*VASH1*
0.02695419	0.7906757	5.446428	3.79E-07	0.01412785	5.325652	chr14	105859135	+	cg25112002	*PACS2*
-0.078774849	0.42479436	-5.4310114	4.05E-07	0.014379921	5.26069405	chr3	22412124	+	cg05529343	-
0.021272448	0.856615124	5.39148139	4.81E-07	0.014914567	5.09458632	chr22	18436985	+	cg15991089	*MICAL3*
0.017024484	0.869007409	5.39466251	4.74E-07	0.014914567	5.10792928	chr15	102196103	+	cg13151645	*TARSL2*
0.02604807	0.6333338	5.40228	4.59E-07	0.01491457	5.139898	chr1	17914803	+	cg26224358	*ARHGEF10L*
0.043368153	0.479191092	5.36636878	5.35E-07	0.015946256	4.98940362	chr1	111218287	+	cg07421287	*KCNA3*
0.02638869	0.6789015	5.3429208	5.92E-07	0.016951385	4.89143542	chr20	3718325	-	cg07888289	*HSPA12B*
-0.052549464	0.527230073	-5.3154695	6.65E-07	0.018353374	4.77704043	chr17	73824354	+	cg12407791	*UNC13D*
-0.032732513	0.759185943	-5.2894959	7.43E-07	0.019768027	4.66910369	chr12	113502194	-	cg16719605	*DTX1*
0.04777264	0.1050803	5.264262	8.27E-07	0.01991374	4.564524	chr22	51066394	+	cg25575065	*ARSA*
0.036103536	0.633064288	5.27139601	8.02E-07	0.019913743	4.59406096	chr9	130660890	+	cg10529558	*ST6GALNAC6*
0.0265421	0.297246858	5.25632625	8.55E-07	0.019913743	4.53169085	chr4	1858883	+	cg02481307	*LETM1*
-0.018442432	0.116163028	-5.2625915	8.33E-07	0.019913743	4.55760901	chr3	27772705	-	cg05583831	-
0.028341888	0.421004434	5.19880539	1.09E-06	0.021387415	4.29455036	chr14	74227875	+	cg13976502	*C14orf43*
-0.006444261	0.0372681	-5.2070065	1.05E-06	0.021387415	4.32827091	chr19	58962884	+	cg05500574	*ZNF324B*
-0.014244634	0.078078116	-5.2208377	9.94E-07	0.021387415	4.38520872	chr8	26371428	-	cg09656164	*DPYSL2*
-0.041665076	0.825712262	-5.2239772	9.81E-07	0.021387415	4.39814467	chr13	40171521	-	cg13714636	*LHFP*
-0.060472705	0.627160734	-5.2165828	1.01E-06	0.021387415	4.36768357	chr16	70838524	+	cg16450432	-

0.02387941	0.7519493	5.201488	1.08E-06	0.02138742	4.305579	chr8	11308943	+	cg24453881	*FAM167A*
0.024704979	0.129183752	5.18136473	1.17E-06	0.021867346	4.22294021	chr1	236559595	-	cg15677293	*EDARADD*
-0.044899983	0.258387701	-5.1843353	1.16E-06	0.021867346	4.23512754	chr7	71868412	+	cg19757435	*CALN1*
-0.027972017	0.827843678	-5.1727355	1.22E-06	0.022122919	4.18755991	chr1	153722267	-	cg04893758	*INTS3*
0.045352991	0.622877226	5.15832063	1.29E-06	0.022945143	4.1285334	chr1	4193806	-	cg04902529	-
0.031357207	0.282793655	5.13704294	1.41E-06	0.023415339	4.04157748	chr18	2907322	-	cg06856216	*EMILIN2*
-0.039263812	0.685254024	-5.13781	1.41E-06	0.023415339	4.04470881	chr4	144206970	+	cg15348902	-
-0.051711729	0.664475164	-5.1451912	1.37E-06	0.023415339	4.07485279	chr17	73824760	-	cg23470227	*UNC13D*
0.023183388	0.694339982	5.12870568	1.46E-06	0.02372057	4.0075619	chr16	85123342	-	cg07714420	*KIAA0513*
0.02344066	0.731106266	5.09842949	1.66E-06	0.026349178	3.88430532	chr16	67879671	+	cg16436566	*CENPT*
-0.018586938	0.098087663	-5.0884001	1.73E-06	0.02663562	3.8435683	chr18	3594243	+	cg06879567	*DLGAP1*
-0.062518007	0.406780726	-5.0858506	1.75E-06	0.02663562	3.83322023	chr17	73824396	-	cg07010633	*UNC13D*
0.02393518	0.7399326	5.008159	2.42E-06	0.02906683	3.519343	chr22	25390937	+	cg24930618	-
0.01692666	0.1180891	5.036858	2.15E-06	0.02906683	3.634955	chr22	42679444	+	cg25219329	-
-0.02297482	0.2473052	-5.015708	2.34E-06	0.02906683	3.549717	chr6	5507831	+	cg25781385	*FARS2*
0.055033991	0.313881153	5.01740648	2.33E-06	0.029066833	3.55655354	chr1	16554984	+	cg11211795	-
0.04861469	0.480699429	5.01445368	2.36E-06	0.029066833	3.54466736	chr16	86155905	+	cg19472984	-
0.030526302	0.736591647	5.05500538	1.99E-06	0.029066833	3.70826463	chr22	37750979	-	cg09474699	*ELFN2*
0.030123419	0.721488765	5.00838203	2.42E-06	0.029066833	3.52023957	chr22	43044387	+	cg20950843	*CYB5R3*
0.026450953	0.717258033	5.01053839	2.40E-06	0.029066833	3.52891316	chr5	130598541	+	cg17896976	*CDC42SE2*
-0.019092657	0.268081941	-5.0154754	2.35E-06	0.029066833	3.54877954	chr1	221924858	+	cg09341616	-
-0.02114592	0.820590203	-5.014144	2.36E-06	0.029066833	3.54342115	chr17	39184878	+	cg19691337	*KRTAP1-5*
-0.031796079	0.328280819	-5.0401893	2.12E-06	0.029066833	3.64840225	chr10	125754900	+	cg23414330	-
-0.032119434	0.198569088	-5.0090378	2.41E-06	0.029066833	3.52287692	chr1	9488706	+	cg03605773	-
-0.047072886	0.662397401	-5.0594496	1.96E-06	0.029066833	3.7262408	chr4	58027287	-	cg20538211	-
0.086788359	0.554523661	4.99547598	2.55E-06	0.030145161	3.4683736	chr1	92947588	+	cg09935388	*GFI1*
-0.010405455	0.064253414	-4.9881334	2.63E-06	0.030587613	3.43890116	chr18	31802530	-	cg00443596	*NOL4*
0.022667082	0.759221354	4.97389013	2.79E-06	0.031939289	3.38180378	chr18	42467626	+	cg07864342	*SETBP1*
-0.047021896	0.384872426	-4.9647843	2.89E-06	0.032657665	3.34535188	chr10	134650743	-	cg21073126	*CFAP46*
0.052120714	0.482779856	4.95548951	3.01E-06	0.032933064	3.30818513	chr8	144798877	-	cg23562538	*MAPK15*
0.021714884	0.289181234	4.95658248	2.99E-06	0.032933064	3.31255341	chr15	91416060	+	cg10698959	*FURIN*
0.052023185	0.444211256	4.94038157	3.20E-06	0.034041537	3.24786242	chr11	86437953	-	cg05533761	-
-0.02230238	0.090044353	-4.9408938	3.19E-06	0.034041537	3.24990582	chr13	46424943	+	cg10775278	*SIAH3*
0.026986798	0.22417467	4.93550404	3.26E-06	0.034240914	3.22841117	chr12	52416600	-	cg04416566	*NR4A1*
-0.02919867	0.1679837	-4.922392	3.44E-06	0.0350718	3.17618	chr5	191492	+	cg24696500	*LRRC14B*
0.016616236	0.126115661	4.92189207	3.45E-06	0.035071804	3.17418878	chr19	52901431	-	cg03478398	*ZNF528*
-0.01900867	0.865099396	-4.9162887	3.53E-06	0.035071804	3.15189436	chr9	38443393	-	cg13910489	-
-0.019712252	0.132077031	-4.9163415	3.53E-06	0.035071804	3.15210456	chr19	45909670	+	cg03658716	*CD3EAP*
0.060416895	0.182824945	4.90843922	3.65E-06	0.035276471	3.12068915	chr19	14591033	+	cg20742389	*GIPC1*
-0.01366479	0.055355175	-4.9093077	3.63E-06	0.035276471	3.12414007	chr10	25171393	+	cg21757973	*PRTFDC1*

0.023405719	0.869513933	4.90226913	3.74E-06	0.0357144	3.09618141	chr15	40699950	-	cg05294459	*IVD*
0.022086271	0.794667936	4.89901114	3.79E-06	0.035735197	3.08324816	chr12	104074804	+	cg08230697	*STAB2*
0.027282517	0.676969134	4.89060791	3.92E-06	0.036070367	3.04991392	chr20	61161073	+	cg07915635	*C20orf166*
-0.013945602	0.11356745	-4.8911248	3.91E-06	0.036070367	3.0519633	chr6	166402753	+	cg09830769	*LINC00473*
0.024409999	0.767887174	4.88525817	4.01E-06	0.036417334	3.02871048	chr1	14128574	+	cg14947200	*PRDM2*
0.021411367	0.779853681	4.87714108	4.14E-06	0.036747315	2.99656577	chr3	148711966	+	cg07447194	*GYG1*
-0.058989842	0.656861761	-4.8799319	4.10E-06	0.036747315	3.007614	chr17	73824620	-	cg23891399	*UNC13D*
0.019369773	0.741862081	4.87094811	4.25E-06	0.036810458	2.97206275	chr17	78651438	-	cg23700649	*RPTOR*
-0.02132483	0.8718184	-4.872456	4.22E-06	0.03681046	2.978027	chr1	160624409	+	cg25307255	-
0.04074803	0.8060288	4.867141	4.32E-06	0.03695619	2.957009	chr2	223435533	+	cg25817435	-
0.029737783	0.148385881	4.85719597	4.49E-06	0.038045951	2.91771925	chr7	1068125	+	cg20007465	*C7orf50*
-0.055549608	0.643279716	-4.8518896	4.59E-06	0.038439331	2.89677534	chr4	72119734	+	cg13530673	*SLC4A4*
0.030786978	0.558873864	4.83115243	5.00E-06	0.040453885	2.81506273	chr1	46770585	-	cg16906929	*UQCRH*
0.024977972	0.329377279	4.83501396	4.92E-06	0.040453885	2.83026241	chr19	1265877	+	cg09834951	-
-0.03070516	0.163641573	-4.8329502	4.96E-06	0.040453885	2.82213825	chr5	191500	+	cg00607051	*LRRC14B*
-0.05865088	0.2639215	-4.825085	5.12E-06	0.04057961	2.791195	chr2	241976080	-	cg26718213	*SNED1*
-0.022699236	0.104400362	-4.8270427	5.08E-06	0.040579612	2.79889433	chr14	38064493	-	cg10323697	*FOXA1*
-0.058146578	0.501398588	-4.8222174	5.18E-06	0.040621906	2.77992157	chr1	14928945	-	cg08538034	*KIAA1026*
0.038734568	0.643903036	4.80796872	5.49E-06	0.041099286	2.72396487	chr1	17766917	+	cg23913963	*RCC2*
0.029414423	0.648001847	4.80666593	5.52E-06	0.041099286	2.71885376	chr14	93552128	-	cg05284742	*ITPK1*
0.023897695	0.698646096	4.81610055	5.31E-06	0.041099286	2.75588721	chr3	157019345	-	cg17717930	*VEPH1*
-0.031500298	0.087688318	-4.8101776	5.44E-06	0.041099286	2.7326326	chr1	209979470	+	cg22442454	*IRF6*
0.04623477	0.7450017	4.81024	5.44E-06	0.04109929	2.732878	chr6	30720203	-	cg24859433	-
0.022945495	0.771003193	4.80251092	5.61E-06	0.041382061	2.70255846	chr1	144996134	+	cg23306824	*NBPF20*
-0.025163753	0.426907653	-4.7917445	5.86E-06	0.042692025	2.66037474	chr15	86315211	-	cg06527213	*KLHL25*
-0.031130891	0.715690528	-4.785502	6.01E-06	0.042692025	2.63594333	chr20	58940540	+	cg17748896	-
-0.044638855	0.425580238	-4.7851832	6.02E-06	0.042692025	2.63469619	chr7	148001779	-	cg09981964	*CNTNAP2*
-0.02819161	0.4080928	-4.789436	5.92E-06	0.04269203	2.651339	chr10	102760784	+	cg24760467	*LZTS2*
0.03851188	0.651752266	4.78229265	6.09E-06	0.042785444	2.62339029	chr16	11681482	-	cg02051924	*LITAF*
0.02969938	0.7928065	4.777865	6.20E-06	0.0431493	2.606082	chr14	74224613	-	cg25845814	*ELMSAN1*
-0.017271339	0.169131283	-4.7725434	6.33E-06	0.043676875	2.5852896	chr6	12750392	+	cg04950301	*PHACTR1*
0.031621893	0.668710168	4.7699933	6.40E-06	0.043723158	2.57533163	chr20	741937	+	cg00432454	*C20orf54*
0.036838547	0.345553371	4.75938712	6.68E-06	0.045145278	2.53395084	chr11	86510998	-	cg23771366	*PRSS23*
0.02694408	0.445236764	4.75581082	6.77E-06	0.045145278	2.52001062	chr19	3698926	-	cg15200445	*PIP5K1C*
0.026508739	0.468576894	4.75491054	6.80E-06	0.045145278	2.51650242	chr3	39193251	-	cg00501876	*CSRNP1*
0.02650368	0.641597	4.752916	6.85E-06	0.04514528	2.508732	chr7	45120098	+	cg25308878	*NACAD*
0.0211127	0.7754874	4.750896	6.91E-06	0.04514528	2.500862	chr1	155534509	-	cg27628839	-
0.054587653	0.409719017	4.72478889	7.67E-06	0.045637169	2.3993645	chr15	75350380	+	cg18110140	-
0.034586699	0.552575139	4.71755855	7.90E-06	0.045637169	2.37131658	chr1	206224334	-	cg08709672	*AVPR1B*
0.031136263	0.722051718	4.73617987	7.33E-06	0.045637169	2.44360716	chr11	126422954	-	cg00524887	*KIRREL3*

0.025381296	0.777768716	4.74018682	7.21E-06	0.045637169	2.45918606	chr2	99439997	+	cg08517455	*C2orf55*
0.014376437	0.256984094	4.72288926	7.73E-06	0.045637169	2.39199286	chr4	25865291	-	cg19488028	*SEL1L3*
-0.008146577	0.041832833	-4.7335157	7.41E-06	0.045637169	2.43325361	chr17	5095455	-	cg17047734	*ZNF594*
-0.016435216	0.132590975	-4.7321727	7.45E-06	0.045637169	2.42803546	chr15	93388263	-	cg22365178	-
-0.017092821	0.264517177	-4.7328914	7.43E-06	0.045637169	2.43082787	chr2	8007099	-	cg10394117	-
-0.0177893	0.350553495	-4.7312431	7.48E-06	0.045637169	2.42442451	chr4	2691863	-	cg20691469	*FAM193A*
-0.022893892	0.687464108	-4.7181876	7.88E-06	0.045637169	2.37375571	chr4	37841179	+	cg15217637	*PGM2*
-0.0269065	0.787251752	-4.7329523	7.42E-06	0.045637169	2.43106442	chr9	118501562	-	cg20074929	-
-0.02879091	0.84264371	-4.7173847	7.90E-06	0.045637169	2.37064243	chr2	150920147	+	cg13823511	-
-0.02926766	0.184451279	-4.7242309	7.69E-06	0.045637169	2.39719908	chr13	24626904	-	cg14814637	*SPATA13*
-0.030880981	0.837363199	-4.7405193	7.20E-06	0.045637169	2.46047899	chr8	125953232	-	cg02315696	*LOC157381*
-0.030997127	0.4176583	-4.7269997	7.60E-06	0.045637169	2.40794593	chr2	103423288	+	cg19412959	*TMEM182*
0.014299298	0.152601433	4.71173694	8.08E-06	0.045967973	2.34875309	chr17	77816034	+	cg14970515	-
-0.014191343	0.194066784	-4.7133374	8.03E-06	0.045967973	2.35495429	chr15	70929081	-	cg01251686	*SALRNA2*
0.042685712	0.55232304	4.70872914	8.18E-06	0.046172024	2.33710229	chr6	32118312	-	cg08123207	*PRRT1*
0.036113706	0.796693763	4.70282897	8.38E-06	0.046514646	2.31426148	chr10	101914678	+	cg18786841	*ERLIN1*
-0.029639655	0.723986625	-4.7012596	8.43E-06	0.046514646	2.30818906	chr12	131715773	+	cg09717146	-
-0.01756749	0.1048627	-4.704248	8.33E-06	0.04651465	2.319752	chr8	53478706	-	cg26021627	*FAM150A*
0.02698857	0.6825783	4.695248	8.63E-06	0.04729492	2.284942	chr13	111227596	-	cg24670947	-
-0.015899956	0.083717347	-4.6928341	8.72E-06	0.047404507	2.27561056	chr14	38064490	-	cg03026462	*FOXA1*
0.049126206	0.643459049	4.67767456	9.26E-06	0.048501959	2.21708681	chr5	399360	-	cg21161138	*AHRR*
0.026553897	0.83922332	4.68014061	9.17E-06	0.048501959	2.22659889	chr12	105420356	-	cg16527105	*ALDH1L2*
0.018424213	0.806549702	4.67843098	9.23E-06	0.048501959	2.22000415	chr15	78381084	-	cg20001830	-
-0.013513887	0.060036022	-4.6763497	9.31E-06	0.048501959	2.2119778	chr3	157261086	-	cg06827247	*C3orf55*
-0.028142673	0.701805483	-4.6816021	9.12E-06	0.048501959	2.23223781	chr8	49607937	-	cg05151268	*LOC101929268*
-0.035385689	0.455754817	-4.6824087	9.09E-06	0.048501959	2.23535015	chr11	62344917	+	cg16107565	*TUT1*
0.027551943	0.692360701	4.67446707	9.38E-06	0.048527775	2.20471965	chr7	75891115	+	cg11145108	*SRRM3*
0.013745456	0.854736143	4.66838956	9.61E-06	0.049373737	2.1813013	chr17	25819427	-	cg16465695	*KSR1*

Supplemental Table 9 - Lifestyle specific Differentially Methylated Positions (DMPs) correlation analysis

Correlation analysis top DMPs - obesity specific phenotypes and scores *(p-values, correlation coefficiant r)*

total discorvery cohort

		Age (years)		HbA1c (%)		HDL (mmol/l)		LDL (mmol/l)		Apolipoprotein A1 (g/l)		Triglycerides (mmol/l)		Glucose (mmol/l)		Insulin (pmol/l)	
**UCSC RefGene name**	**CpG-name**	**p**	**r**	**p**	**r**	**p**	**r**	**p**	**r**	**p**	**r**	**p**	**r**	**p**	**r**	**p**	**r**
-	cg01940273	0.727661688	-0.03619589	0.197400656	-0.13342198	0.013415853	0.252886733	0.383039221	-0.09051145	0.029975427	0.22282204	0.002423	-0.30764741	0.089637399	-0.17511058	0.027159674	-0.22670208
ANPEP	cg02008229	0.932231335	-0.00884157	0.390394702	0.089125787	0.034350955	-0.21736434	0.331132667	0.100788239	0.058473721	-0.19484284	0.004058452	0.29222754	0.234722728	0.123080254	0.13449391	0.154671214
F2RL3	cg03636183	0.970191362	0.003885326	0.075791342	-0.18305802	0.024677201	0.230417356	0.030878208	-0.22164336	0.024068192	0.231377249	0.007145212	-0.27430441	0.807380056	-0.02534562	0.166425157	-0.14313571
-	cg05529343	0.752148503	0.03282702	0.544378134	-0.06296721	0.230706469	-0.12413477	0.023988043	0.231505115	0.22743616	-0.12500295	0.104500631	0.167597227	0.165370965	-0.14348888	0.47360111	0.074407923
AHRR	cg05575921	0.65918099	0.045833139	0.324445274	-0.10218403	0.03915435	0.212008949	0.16553494	-0.14343384	0.058578607	0.194763379	0.005661684	-0.2818164	0.719942819	-0.03726549	0.240664424	-0.121543
ANPEP	cg06344992	0.644613318	0.047931413	0.059274315	0.194239212	0.009819608	-0.26370382	0.084411859	0.177986749	0.03155802	-0.22077485	0.001861951	0.315217178	0.059318468	0.194206116	0.220674057	0.12682628
UNC13D	cg07010633	0.907049001	0.012139753	0.634376119	0.049417491	0.692027653	-0.04116725	0.033095564	0.21886728	0.925683601	-0.00969817	0.060656032	0.193212902	0.302064182	0.10699482	0.81554703	0.024251145
KIAA1026	cg08538034	0.068250326	0.187889317	0.026459629	0.227719511	0.047451239	-0.20393166	0.015221931	0.248383197	0.061700643	-0.19244957	0.017192183	0.243970446	0.350680994	0.096807823	0.37243161	-0.09253835
GFI1	cg09935388	0.775827301	0.029601323	0.186312066	-0.13676244	0.05766648	0.195458364	0.110579773	-0.1647646	0.014553049	0.249994158	0.024735473	-0.23032657	0.144613003	-0.15081137	0.030075068	-0.22269049
VASH1	cg10387007	0.293833838	-0.10882228	0.037620985	-0.21365568	0.059959376	0.193727952	0.223409524	-0.12608402	0.064644267	0.190353729	0.046173366	-0.2050951	0.47734759	-0.07378074	0.39291137	-0.08865528
SLC4A4	cg13530673	0.199729436	0.132737734	0.569351914	0.059110241	0.010705387	-0.26075261	0.06529465	0.189901152	0.014258956	-0.25072287	0.192596654	0.134852084	0.673580271	0.043777053	0.397773225	0.087751373
PRSS23	cg14391737	0.857137141	0.018715775	0.172010682	-0.14129159	0.073249438	0.184641454	0.364861218	-0.09400649	0.114057386	0.163198242	0.001236247	-0.32661498	0.210472164	-0.12965331	0.207066917	-0.13061858
-	cg16450432	0.343625387	0.098227979	0.921548012	-0.01023953	0.489648111	-0.07173993	0.003679453	0.295222455	0.984421729	0.00203017	0.062312571	0.192007284	0.954155714	-0.00597732	0.34312387	0.098329618
RARA	cg17739917	0.204029227	-0.13148927	0.062346615	-0.19198278	0.040875242	0.210223794	0.264638108	-0.11559355	0.056500309	0.196360132	0.020523879	-0.23741512	0.608012816	-0.05329174	0.206043951	-0.13091077
GIPC1	cg20742389	0.52046166	-0.06674283	0.395106578	-0.08824633	0.833409556	0.021866371	0.500652917	-0.06993699	0.922845048	0.010069716	0.174352762	-0.14053144	0.416790125	0.084277035	0.749726086	-0.03315873
-	cg21566642	0.887236833	0.014742904	0.386415062	-0.08987353	0.093316249	0.17316445	0.559844994	-0.06056876	0.048682122	0.202835262	0.005337341	-0.28368928	0.231587883	-0.12390225	0.069627929	-0.18697563
F2RL3	cg21911711	0.120067258	-0.16057636	0.007648342	-0.27206938	0.081837109	0.179456313	0.010171008	-0.26250618	0.145973385	0.150308174	0.003700876	-0.29504598	0.425176053	-0.08277431	0.297680297	-0.10796408
ANPEP	cg23432008	0.977129927	-0.00298066	0.178680431	0.13914635	0.081205369	-0.17982251	0.572588625	0.058616298	0.057877018	-0.19529715	0.034667694	0.216992455	0.051531153	0.200382708	0.100301283	0.169630036
UNC13D	cg23891399	0.945339637	-0.00712842	0.684639062	-0.04220938	0.322990824	-0.10249003	0.029408224	0.223578031	0.547982057	-0.06240543	0.062001848	0.192231421	0.57690438	0.057959709	0.565245459	-0.05973881
SNED1	cg26718213	0.197696047	-0.13333487	0.907106857	0.012132163	0.15296801	-0.14777518	0.609464461	0.053076552	0.14355197	-0.15120632	0.203905999	0.131524789	0.34993339	0.09695745	0.328519523	0.101331492

		BMI (kg/m2)		Waist circumference (cm)		WHR		Diet score		Physical activity score		Smoking score		Alcohol score		Lifestyle score	
**UCSC RefGene name**	**CpG-name**	**p**	**r**	**p**	**r**	**p**	**r**	**p**	**r**	**p**	**r**	**p**	**r**	**p**	**r**	**p**	**r**
-	cg01940273	0.156247708	-0.14661729	0.003255692	-0.29891718	**8.25E-05**	-0.39278045	**1.87E-08**	-0.53811408	**3.69E-11**	-0.61394982	**2.90E-13**	-0.66162598	0.020386197	-0.23766721	**2.50E-12**	-0.64143973
ANPEP	cg02008229	0.117721007	0.161587614	0.059403189	0.194142667	0.043123689	0.207983471	**4.58E-05**	0.405459172	**5.02E-06**	0.4489945	**1.47E-07**	0.508152416	0.007662448	0.272008615	**4.27E-07**	0.491392465
F2RL3	cg03636183	0.539009967	-0.06380738	0.042295146	-0.20879745	0.003693022	-0.29511057	**1.61E-11**	-0.62270097	**1.93E-10**	-0.59560379	**2.22E-16**	-0.71999288	0.003246008	-0.29900655	**3.66E-14**	-0.67964869
-	cg05529343	0.742258891	-0.03418334	0.499837853	0.070069808	0.059485776	0.194080887	**9.63E-05**	0.389350534	**1.19E-05**	0.432734616	**3.00E-08**	0.531465757	0.058271775	0.194996173	**7.01E-07**	0.483322094
AHRR	cg05575921	0.666993705	-0.04471541	0.08746247	-0.17629106	0.005610834	-0.28210362	**5.52E-11**	-0.60959851	**1.07E-11**	-0.62695853	**0**	-0.75112225	0.006502232	-0.27737244	**2.66E-15**	-0.70037046
ANPEP	cg06344992	0.05855968	0.19477771	0.015091232	0.248693086	0.008022104	0.27049151	**2.38E-05**	0.41895768	**1.34E-06**	0.472449653	**8.75E-08**	0.515935197	0.003030642	0.301058384	**1.34E-07**	0.509563061
UNC13D	cg07010633	0.655661447	-0.04633837	0.611519342	0.052772324	0.308197894	0.105654	0.025878163	0.228582144	**2.41E-06**	0.462250795	**1.42E-05**	0.429250399	0.015339348	0.248106757	**1.39E-05**	0.429744823
KIAA1026	cg08538034	0.277271601	-0.11260508	0.56585557	0.059645289	0.005764239	0.281244013	0.000392139	0.356436851	**2.04E-05**	0.422133312	**2.83E-06**	0.459392875	0.108265483	0.165828317	**7.87E-06**	0.4406245
GFI1	cg09935388	0.158382685	-0.14587326	0.040871132	-0.21022798	0.020687352	-0.23711769	**3.74E-05**	-0.40970506	**0.00010383**	-0.38767077	**9.24E-08**	-0.5151135	0.016145622	-0.24625649	**1.79E-06**	-0.46747212
VASH1	cg10387007	0.984746521	-0.00198784	0.044753124	-0.20641988	**4.07E-05**	-0.40794823	**3.51E-05**	-0.41103268	**1.37E-06**	-0.47209768	**3.33E-09**	-0.56112085	0.000215445	-0.37090577	**2.57E-08**	-0.53362862
SLC4A4	cg13530673	0.553316865	0.061577126	0.26123951	0.116413912	0.026777761	0.227254344	**1.95E-05**	0.422983106	**1.35E-05**	0.43020212	**1.51E-05**	0.428098394	0.008614202	0.268119698	**3.82E-06**	0.454003473
PRSS23	cg14391737	0.721351082	-0.03707006	0.064494084	-0.19045876	0.000690709	-0.3421013	**3.41E-07**	-0.49497987	**1.03E-10**	-0.60276713	**1.84E-12**	-0.64443365	0.000708536	-0.34143978	**5.99E-12**	-0.63280747
-	cg16450432	0.597397686	0.054872244	0.173346487	0.140857113	0.021243458	0.236120741	0.001080872	0.330259758	**6.55E-07**	0.484428138	**4.09E-07**	0.492096833	0.046530273	0.204767496	**9.40E-07**	0.478434379
RARA	cg17739917	0.992035035	-0.00103795	0.06038017	-0.19341627	0.000220471	-0.37036104	**8.52E-08**	-0.51633116	**2.30E-09**	-0.56584489	**1.18E-13**	-0.66963594	0.003472951	-0.29697244	**1.02E-11**	-0.62738651
GIPC1	cg20742389	0.486254391	0.072300232	0.716502034	-0.03774352	0.026293857	-0.22796379	**6.15E-05**	-0.39915523	**4.22E-06**	-0.45218685	**4.03E-06**	-0.45301054	0.295914272	-0.1083572	**4.25E-06**	-0.45205315
-	cg21566642	0.185547809	-0.13699793	0.005687638	-0.28167068	0.000348097	-0.35936983	**5.00E-08**	-0.52414161	**7.52E-10**	-0.57963439	**2.70E-12**	-0.64068112	0.000396404	-0.35616917	**8.92E-12**	-0.62878425
F2RL3	cg21911711	0.721384507	-0.03706542	0.04983652	-0.20182761	0.000351686	-0.35911839	**4.66E-10**	-0.58536067	**1.71E-11**	-0.62205608	**1.05E-13**	-0.67061387	0.018368516	-0.24154007	**4.59E-13**	-0.65744559
ANPEP	cg23432008	0.028438971	0.22489894	0.020273628	0.237874404	0.031064558	0.221403689	**0.00012705**	0.38312561	**1.83E-05**	0.424244633	**2.67E-07**	0.498868281	0.000592431	0.346055319	**7.97E-07**	0.481198887
UNC13D	cg23891399	0.587724086	-0.05632353	0.599807564	0.054512347	0.070904758	0.186141737	0.003032876	0.301036438	**2.23E-05**	0.420339256	0.000173284	0.376002977	0.001416334	0.322878644	**3.32E-05**	0.412153395
SNED1	cg26718213	0.695808214	0.040635598	0.319216868	0.103288179	0.1308622	0.156110305	**8.49E-05**	0.392130334	**4.25E-05**	0.40701809	**3.97E-05**	0.40848263	0.187383843	0.136433376	**2.59E-05**	0.417261854

Supplemental Table 10 - Differentially Methylated Regions (DMRs) of the healthy living subjects

Chromosome	Start	End	Number CpGs	Min smoothed FDR	Max difference	Mean difference	UCSC RefGene name
chr11	368351	369810	22	6.64E-18	0.065163213	0.023335767	*B4GALNT4*
chr7	27142100	27144595	25	4.49E-13	-0.081273886	-0.031216228	*HOXA2*
chr5	1724892	1726753	11	2.45E-12	-0.071439316	-0.048098768	*NA*
chr2	70311984	70313833	12	1.22E-11	0.075512447	0.028335816	*PCBP1;PCBP1-AS1*
chr3	148803510	148805294	30	5.92E-10	-0.050393453	-0.007758352	*HLTF;HLTF-AS1*
chr8	143545390	143546893	10	3.95E-09	-0.066684942	-0.030342854	*ADGRB1*
chr16	302852	304120	5	8.07E-09	0.077970945	0.049768236	*FAM234A;LUC7L*
chr4	5021111	5022001	9	1.44E-08	-0.078323055	-0.03084229	*CYTL1*
chr4	74718978	74719306	6	1.79E-08	-0.079009565	-0.046120369	*PF4V1*
chr16	102920	104661	15	2.05E-08	-0.022923118	-0.011465236	*POLR3K;SNRNP25*
chr18	47019331	47019823	3	2.05E-08	0.247069267	0.082244409	*RPL17;RPL17-C18orf32;SNORD58A;SNORD58B;SNORD58C*
chr17	40949811	40950659	4	3.72E-08	-0.058045636	-0.021454985	*CNTD1;COA3*
chr17	73823769	73825227	7	5.32E-08	-0.066076979	-0.036474972	*UNC13D*
chr1	89459504	89459897	4	7.95E-08	-0.092231368	-0.065402236	*KYAT3;RBMXL1*
chr16	85368682	85369142	4	1.19E-07	0.081358412	0.067701301	*NA*
chr16	1185381	1186286	7	1.22E-07	0.071358273	0.021455503	*NA*
chr13	96204854	96206104	12	1.49E-07	-0.103001215	-0.016609448	*CLDN10*
chr8	49426532	49427684	5	1.62E-07	-0.161721055	-0.093448573	*NA*
chr6	33043138	33044345	7	1.62E-07	-0.035486502	-0.013521517	*HLA-DPA1;HLA-DPB1*
chr15	22833149	22833975	12	1.78E-07	-0.049684644	-0.024253755	*TUBGCP5*
chr11	2292361	2293665	34	2.27E-07	-0.05826388	-0.011684735	*ASCL2*
chr17	81060149	81060259	3	6.42E-07	-0.181799916	-0.156378516	*NA*
chr11	44331978	44333192	37	7.87E-07	0.030608729	0.006881931	*ALX4*
chr10	123356926	123357993	8	8.70E-07	0.046117035	0.011791043	*FGFR2*
chr4	54965109	54965829	3	8.81E-07	0.025923365	0.005993457	*GSX2;PDGFRA*
chr16	90015428	90016551	6	9.41E-07	0.095910751	0.051408837	*DEF8*
chr17	55962155	55963458	7	2.06E-06	-0.089775517	-0.043770979	*CUEDC1*
chr16	54227790	54228582	8	2.28E-06	-0.063343598	-0.031653407	*NA*
chr1	152595322	152596177	8	3.43E-06	0.05777368	0.007426018	*LCE3A*
chr1	203198757	203199023	3	3.49E-06	0.062061134	0.035564294	*CHIT1*
chr16	75299270	75300795	13	3.52E-06	-0.024622473	0.004142489	*BCAR1*
chr6	168630207	168630378	3	3.67E-06	-0.039912491	-0.028450128	*NA*
chr5	54281198	54281733	9	4.27E-06	-0.058174811	-0.036360656	*ESM1*
chr11	72533202	72533664	4	4.31E-06	0.080214369	0.052971526	*ATG16L2*
chr12	122018375	122020205	21	4.46E-06	0.029845887	0.009664682	*KDM2B*
chr16	2042310	2043558	5	4.69E-06	-0.057586683	-0.011061699	*SYNGR3*
chr13	111267397	111268714	19	4.70E-06	-0.04827911	-0.012207826	*NAXD*
chr10	42862876	42863594	8	4.72E-06	-0.085822439	-0.074968077	*LOC441666*
chr11	65313608	65315284	11	4.86E-06	-0.084298445	-0.034713301	*LTBP3*
chr1	167090618	167091161	7	4.91E-06	-0.055826538	-0.020475337	*STYXL2*
chr6	32976131	32977412	12	5.06E-06	-0.059793051	-0.010832415	*HLA-DOA*
chr5	76010472	76011428	8	5.65E-06	-0.038035938	-0.007838482	*F2R*
chr4	184908254	184909509	12	5.74E-06	-0.055417802	-0.025960326	*STOX2*
chr20	3073301	3074231	4	5.81E-06	0.052221009	0.026487131	*NA*
chr11	76749893	76751369	7	5.93E-06	-0.076634895	-0.026654733	*B3GNT6*
chr6	31583231	31584941	8	6.25E-06	0.067538738	0.032452513	*AIF1;PRRC2A*

chr5	176151771	176153865	9	6.75E-06	-0.053137077	-0.018473544	*NA*
chr4	154178264	154179401	4	6.94E-06	-0.058934143	-0.039394613	*TRIM2*
chr16	877559	878753	6	6.94E-06	-0.040776112	-0.011231069	*NA*
chr16	12183789	12185012	10	6.99E-06	-0.050259685	-0.018176721	*SNX29*
chr16	6105700	6106292	3	6.99E-06	-0.060137698	0.001190084	*RBFOX1*
chr5	150004235	150004918	6	1.03E-05	-0.039031406	-0.016991107	*SYNPO*
chr1	110081045	110082294	6	1.07E-05	-0.031892266	-0.005970586	*GPR61*
chr16	90143751	90144788	6	1.11E-05	-0.069894321	-0.050060912	*PRDM7*
chr17	38464730	38465510	10	1.31E-05	0.023706778	0.01345011	*RARA*
chr10	101280750	101283090	15	1.52E-05	-0.097771333	-0.012942116	*NA*
chr11	14993818	14995167	27	1.65E-05	0.044231492	0.008426364	*CALCA*
chr12	14926317	14927542	14	1.70E-05	-0.068710764	-0.022709198	*H2AJ;H4-16*
chr15	44092166	44093429	16	1.70E-05	0.021488169	0.002283906	*ELL3;HYPK;SERF2-C15ORF63;SERINC4*
chr12	131400857	131402040	10	1.76E-05	0.10481621	0.01864437	*NA*
chr5	135415693	135416613	14	1.76E-05	-0.119506699	-0.069593095	*VTRNA2-1*
chr2	21266500	21268152	15	1.83E-05	-0.087105736	-0.034723271	*APOB*
chr4	11369007	11370015	4	1.83E-05	-0.061433354	-0.028134622	*MIR572*
chr2	32491644	32491994	4	1.83E-05	0.03269225	0.011633954	*NLRC4*
chr11	65153757	65154388	11	1.94E-05	-0.01092507	0.000918099	*FRMD8;SLC25A45*
chr2	201828496	201829669	10	2.32E-05	-0.045341695	-0.010204489	*ORC2*
chr17	16472123	16473468	12	2.33E-05	-0.03815289	-0.003654633	*ZNF287*
chr5	176011567	176011783	3	2.34E-05	0.02802341	0.002421465	*CDHR2*
chr21	40823279	40824019	7	2.37E-05	-0.039286756	-0.021056335	*SH3BGR*
chr4	185018391	185019116	4	2.49E-05	-0.098239599	-0.058499666	*ENPP6*
chr21	27106248	27107101	9	2.59E-05	-0.047541296	-0.007298684	*ATP5PF;GABPA*
chr18	74799250	74800080	7	2.63E-05	0.101677456	0.052222941	*MBP*
chr11	32458656	32459428	8	2.65E-05	0.023184776	0.002402127	*WT1;WT1-AS*
chr1	24644925	24646205	17	2.75E-05	0.019744332	0.009527373	*GRHL3*
chr19	41255304	41256395	7	2.76E-05	-0.025011045	-0.013571488	*C19orf54;SNRPA*
chr19	19772686	19773068	3	2.85E-05	-0.02118906	-0.009393532	*ATP13A1*
chr6	27521330	27521477	3	2.93E-05	0.045496843	0.0389347	*NA*
chr8	62199810	62200689	8	2.97E-05	0.09220422	0.024464328	*CLVS1*
chr13	96296708	96297326	11	3.16E-05	0.064597374	0.014049346	*DZIP1*
chr3	126911277	126912011	11	3.24E-05	-0.048020133	-0.02330443	*NA*
chr12	125019756	125020479	8	3.30E-05	-0.026228668	-0.002092329	*NCOR2*
chr10	2978126	2978687	5	3.40E-05	-0.049514601	-0.037195269	*NA*
chr14	102963178	102964872	10	3.70E-05	-0.030427566	-0.009334554	*TECPR2*
chr2	220282281	220283442	9	3.84E-05	-0.050850949	-0.019541439	*DES*
chr2	190743462	190744385	6	3.86E-05	-0.079702648	-0.02314512	*NA*
chr6	32144667	32146071	27	4.32E-05	-0.020854478	0.002313041	*AGPAT1;RNF5*
chr16	80966033	80966860	9	4.34E-05	-0.084897175	-0.023599603	*NA*
chr14	105619511	105620358	4	4.41E-05	-0.049594125	-0.035505112	*JAG2*
chr14	101495758	101496410	9	4.65E-05	0.035728481	0.001794361	*MIR1193;MIR494;MIR495;MIR543*
chr12	71113067	71113529	4	5.09E-05	-0.06529439	-0.044576144	*PTPRR*
chr19	14229237	14229581	4	5.54E-05	0.019542954	0.00928279	*PRKACA*
chr1	162335982	162337375	9	5.54E-05	0.046462439	0.020464423	*NOS1AP*
chr16	87780848	87782144	6	5.54E-05	0.070114488	0.028467647	*KLHDC4*
chr17	59533081	59534102	4	5.62E-05	0.049398674	0.012467179	*TBX4*
chr1	4714974	4715615	8	5.65E-05	-0.0160571	0.000834912	*AJAP1*

chr8	27467783	27469338	10	6.52E-05	0.028415475	0.013984612	*CLU*
chr7	2018435	2018520	4	6.73E-05	-0.025541481	-0.013596861	*MAD1L1*
chr7	157853843	157854772	7	6.92E-05	-0.036783762	0.005659179	*PTPRN2*
chr11	123985614	123986166	10	7.53E-05	0.019300968	0.002903184	*VWA5A*
chr12	99139301	99139867	5	7.56E-05	-0.024925148	-0.010535133	*ANKS1B*
chr2	239047774	239048579	4	7.87E-05	-0.054954241	-0.034166874	*KLHL30*
chr17	79668348	79669841	6	7.95E-05	-0.037486354	-0.011569457	*HGS;MRPL12;SLC25A10*
chr19	613111	613818	3	8.31E-05	-0.117824751	-0.110563755	*HCN2*
chr1	152506173	152506922	3	8.36E-05	-0.059220539	-0.021772006	*NA*
chr15	101389272	101390350	13	8.41E-05	-0.064277235	-0.027205728	*NA*
chr19	49339789	49340765	6	8.74E-05	-0.061270659	-0.037598869	*HSD17B14;PLEKHA4*
chr9	139980676	139982149	8	8.82E-05	0.007760766	0.000496341	*MAN1B1;MAN1B1-DT*
chr1	235805286	235806070	6	8.85E-05	-0.063209969	-0.035602373	*GNG4*
chr1	201687738	201688419	3	9.67E-05	-0.098055875	-0.031050433	*MIR5191;NAV1*
chr4	1686288	1686736	3	0.000109114	0.078850969	0.027990228	*FAM53A*
chr19	51524249	51524763	5	0.000113324	-0.052155534	-0.02605052	*KLK10*
chr3	194867861	194868843	8	0.000116986	0.041390664	0.016211163	*XXYLT1;XXYLT1-AS2*
chr6	87965764	87966747	3	0.000121626	-0.070043264	-0.021641325	*ZNF292*
chr11	65429275	65429987	3	0.00012895	0.057746409	0.024403152	*RELA*
chr19	12443716	12445173	11	0.00013561	0.02044112	0.006528592	*ZNF563*
chr12	118574730	118575033	3	0.0001419	-0.030581455	-0.012939604	*PEBP1*
chr1	37325001	37325677	3	0.000151008	-0.054163673	-0.023332257	*GRIK3*
chr16	88767068	88768194	4	0.000151448	0.035315621	0.021158699	*CTU2;RNF166*
chr2	55746015	55746328	5	0.000154185	-0.059294106	-0.020845493	*CFAP36*
chr16	19896317	19897093	9	0.000154565	0.047895694	0.014202769	*GPRC5B*
chr12	54451877	54452069	3	0.000157087	-0.069587506	-0.047086226	*FLJ12825*
chr8	143324660	143324994	4	0.000185197	-0.08543437	-0.027050448	*TSNARE1*
chr1	178455607	178456270	5	0.000185306	-0.060714974	-0.041268205	*NA*
chr4	183721183	183721778	5	0.000212981	-0.099206235	-0.044084404	*TENM3*
chr9	136149908	136150361	4	0.000215694	-0.088490704	-0.062976756	*ABO*
chr11	1650595	1650863	3	0.000215694	0.07156859	0.046430557	*KRTAP5-5;MOB2*
chr2	2295451	2295969	7	0.000218057	-0.06029634	-0.03176046	*MYT1L*
chr8	96085249	96085994	6	0.000226731	-0.072668009	-0.032969847	*MIR3150B;NDUFAF6*
chr15	67144403	67145855	4	0.000227895	-0.026941768	-0.011439697	*NA*
chr6	36355127	36356244	13	0.000234997	-0.043584108	-0.015032645	*ETV7*
chr3	44802754	44804219	15	0.000239702	0.013689228	0.00316573	*KIAA1143;KIF15*
chr14	100402775	100403675	4	0.000242932	0.034259728	0.004812265	*EML1*
chr4	6107021	6107791	8	0.000243536	0.077530622	0.028797539	*JAKMIP1*
chr11	267613	268116	3	0.000244002	0.021378695	0.016958221	*NA*
chr11	14280333	14281157	6	0.000245283	-0.071920879	-0.043680974	*SPON1*
chr22	22901145	22902237	15	0.000249875	-0.029990762	-0.015169137	*LL22NC03-63E9.3;PRAME*
chr6	31803718	31804883	7	0.000250313	-0.037493735	-0.018120953	*SNHG32;SNORD52*
chr2	27371537	27372132	8	0.000250313	-0.023184524	-0.008384218	*TCF23*
chr16	1728898	1729252	3	0.000252132	-0.023091901	-0.003488497	*CRAMP1;JPT2*
chr13	110438256	110439234	10	0.000253501	0.025203726	0.012706608	*IRS2*
chr19	611107	611506	4	0.000276301	-0.05956778	-0.048901087	*HCN2*
chr2	3605983	3606561	11	0.000276361	0.012484432	0.001832791	*RNASEH1;RNASEH1-AS1*
chr10	6190408	6191207	4	0.0002801	0.02219635	0.009277035	*MIR3155A;PFKFB3*
chr3	38060265	38060691	4	0.000280572	0.0278808	0.009611868	*PLCD1*

chr17	46755422	46756094	6	0.000284657	-0.04527134	-0.012735655	*NA*
chr17	6678929	6679564	11	0.000293813	0.046886522	0.016261681	*FBXO39;XAF1*
chr13	27998168	27999546	13	0.000302893	0.064505125	0.019590198	*GTF3A*
chr1	231376203	231377187	16	0.000303656	-0.014523175	-0.000406571	*C1orf131;GNPAT*
chr12	121533475	121533833	4	0.000309113	-0.066654002	-0.013758723	*NA*
chr5	1875548	1876719	9	0.000317707	0.029855246	0.013000563	*NA*
chr4	155702069	155703987	18	0.000320328	0.070442779	0.017414598	*RBM46*
chr7	29844982	29846259	8	0.000327961	-0.038658066	-0.013032312	*WIPF3*
chr16	58533743	58534708	9	0.000337226	-0.079139721	-0.055643319	*NDRG4*
chr15	93580022	93580846	7	0.000340737	0.065520393	0.028778062	*NA*
chr6	33375357	33375711	4	0.000375101	0.031475943	0.014437144	*KIFC1;PHF1*
chr10	106099036	106100036	4	0.000386808	-0.053136143	-0.028266884	*ITPRIP*
chr8	145652729	145654565	12	0.000402757	0.022062732	0.000702762	*TONSL;VPS28*
chr7	44365023	44366202	13	0.000402757	0.020756784	0.005552571	*CAMK2B*
chr4	47032325	47033423	11	0.00040927	-0.048484976	-0.016570199	*GABRB1*
chr8	95961754	95962463	12	0.000419249	-0.055540436	-0.023548512	*TP53INP1*
chr5	141094626	141095628	4	0.000419249	0.044317635	0.000975492	*NA*
chr6	40400230	40400462	3	0.000419324	-0.034720848	-0.015080491	*LRFN2*
chr1	110949563	110950345	8	0.000459147	-0.036914556	-0.007382054	*LAMTOR5;LAMTOR5-AS1*
chr16	71927972	71928946	13	0.000470799	-0.037779679	-0.00284114	*IST1*
chr19	35530755	35532097	13	0.000470799	0.025941821	0.009712071	*HPN;SCN1B*
chr18	111676	112528	4	0.000470799	0.044406623	0.028283228	*ROCK1P1*
chr5	115697214	115697696	4	0.000470799	0.046194623	0.03585366	*NA*
chr19	42070397	42071240	10	0.000488507	0.027666806	0.013014159	*CEACAM21*
chr17	40550057	40550525	3	0.000488946	-0.031414926	-0.009103201	*NA*
chr19	36119199	36119666	4	0.000488946	0.043028319	0.02078672	*RBM42*
chr15	74423308	74424054	6	0.000506587	0.018975057	0.011072455	*ISLR2;LOC283731*
chr2	109402797	109403565	14	0.000514502	-0.014195331	-0.001670947	*CCDC138*
chr1	26503623	26504548	8	0.000522616	-0.055777289	-0.028496787	*CNKSR1*
chr15	99789622	99791525	16	0.000527156	-0.063848252	-0.026696002	*LRRC28;TTC23*
chr7	36067955	36068970	3	0.000538979	-0.053188734	-0.027366632	*NA*
chr1	845311	846742	6	0.000538979	-0.054745691	-0.026593173	*NA*
chr20	821388	822499	8	0.000538979	0.029891449	0.01229957	*FAM110A*
chr11	133788233	133789946	5	0.00056698	-0.093222033	-0.047853821	*IGSF9B*
chr7	29184994	29187019	18	0.00056698	0.041961147	0.010071981	*CHN2;CPVL*
chr1	11714784	11715247	5	0.000569658	-0.010833709	-0.000817975	*FBXO2;FBXO44*
chr5	172710360	172711094	6	0.00057149	0.036427697	0.01242455	*NA*
chr10	50649723	50650248	4	0.000577186	-0.059770382	-0.049454152	*NA*
chr1	3563415	3564676	6	0.000585679	0.040254553	0.017681217	*TP73;WRAP73*
chr7	751009	751833	5	0.000596812	0.061452832	0.027141645	*PRKAR1B*
chr3	192958565	192959762	9	0.000598181	0.03108194	0.012157954	*MGC2889;PLAAT1*
chr1	2106222	2106732	5	0.000606967	-0.048739489	-0.035280774	*PRKCZ*
chr16	62070219	62071419	13	0.000606967	0.033163893	0.00220867	*CDH8*
chr5	172385451	172387101	22	0.000617248	-0.048406446	-0.005730407	*LOC100268168;RPL26L1*
chr1	15130959	15131772	3	0.000617248	0.033298351	-0.005123772	*KAZN*
chr9	132020061	132021167	7	0.000621328	0.046437461	0.009048076	*NA*
chr10	89263578	89264513	10	0.000629394	0.062264041	0.018288872	*MINPP1;MIR4678*
chr22	39709853	39710068	5	0.000635108	-0.035761966	-0.010737155	*RPL3*
chr17	79465866	79466438	4	0.000674098	-0.075172806	-0.045716682	*NA*

chr8	1764878	1766281	15	0.000680348	0.029395981	0.014041858	*MIR596*
chr15	79851166	79852078	3	0.000680348	0.032538995	0.020326536	*NA*
chr2	240301504	240302888	7	0.000682487	0.04343189	0.011285594	*HDAC4*
chr5	140561239	140563400	15	0.000696283	0.085143436	0.025479001	*PCDHB16*
chr3	195578011	195578280	5	0.000697885	0.054451472	0.020429483	*NA*
chr1	16011710	16012588	3	0.000702141	0.035187896	0.010639714	*PLEKHM2*
chr1	220863127	220864321	16	0.000705297	0.031888864	0.008498706	*C1orf115*
chr4	81117853	81119473	13	0.000714125	0.054309156	0.028698208	*PRDM8*
chr15	29562049	29562542	8	0.000719969	0.015944747	0.003648847	*FAM189A1;NSMCE3*
chr7	35735019	35735464	4	0.000720625	-0.099111387	-0.025905038	*HERPUD2*
chr8	145925258	145926766	11	0.000738013	0.044993871	0.015247443	*NA*
chr1	161368787	161369277	5	0.000742747	-0.088613928	-0.041433866	*NA*
chr5	175995592	175995896	3	0.000742747	-0.042743653	-0.018869938	*CDHR2*
chr6	32380937	32381888	7	0.000742747	0.023006705	0.004941644	*NA*
chr12	124985272	124986124	4	0.000742747	0.044999791	0.018468598	*NCOR2*
chr19	4607057	4607540	7	0.000762382	-0.029367236	-0.009917319	*NA*
chr5	180631001	180631909	4	0.00077512	-0.019529501	-0.004360844	*TRIM7*
chr5	138210550	138211909	15	0.000791555	0.031751142	0.017297474	*CTNNA1;LRRTM2*
chr11	45671015	45671369	4	0.000793662	-0.079946665	-0.05179087	*CHST1*
chr16	66953825	66954273	4	0.000797506	-0.034996529	-0.032578881	*CDH16*
chr7	32338542	32339497	12	0.000801148	-0.043487316	-0.021383745	*PDE1C*
chr22	32058214	32058810	9	0.000827073	-0.053496038	-0.015903577	*PISD*
chr11	120254723	120255253	3	0.000831392	0.036562367	0.000585692	*ARHGEF12*
chr1	204226218	204226671	3	0.000839828	-0.02793239	-0.020560395	*PLEKHA6*
chr1	22137435	22138381	4	0.000850195	0.042839695	-0.004014872	*LDLRAD2*
chr11	43902062	43902786	8	0.000857998	0.034548965	0.015830268	*ALKBH3*
chr15	42565651	42566390	18	0.000863212	0.019187913	0.003874243	*GANC;TMEM87A*
chr7	6615984	6616677	5	0.000865786	-0.11456779	-0.036469312	*ZDHHC4*
chr16	1094653	1096754	8	0.000867732	0.06370825	0.012991511	*NA*
chr21	16435593	16436713	6	0.000879131	0.020628745	0.007941477	*NRIP1*
chr5	161494015	161494810	12	0.000884394	0.064291335	0.013699324	*GABRG2*
chr8	49493865	49494724	3	0.00090067	-0.067589524	-0.030812037	*NA*
chr5	64064244	64065454	13	0.00090623	0.019690402	0.004292871	*CWC27;SREK1IP1*
chr13	111521981	111522932	7	0.00090623	0.048131832	0.010888404	*PRECSIT*
chr4	2277157	2278261	7	0.000917011	0.030040715	0.003957573	*ZFYVE28*
chr18	21718735	21719568	17	0.000927844	-0.048429847	-0.013251299	*CABYR*
chr7	27203430	27204663	8	0.000928485	0.021092874	0.009885148	*HOXA10-AS;HOXA10-HOXA9;HOXA9*
chr1	29101609	29102153	5	0.000950003	0.068609948	0.026202974	*NA*
chr11	126172682	126174329	19	0.000954592	-0.04986137	-0.011945608	*DCPS*
chr12	10182372	10183850	9	0.000985456	0.051227582	0.026084571	*CLEC9A*
chr5	173068163	173068482	3	0.000999032	-0.025336206	-0.013134467	*NA*
chr3	10370264	10370704	3	0.001002893	-0.045738927	-0.040445047	*ATP2B2*
chr18	7566258	7566781	5	0.001009216	-0.017245905	-0.000832405	*PTPRM*
chr17	3819880	3820910	7	0.001037481	0.023230213	0.009328253	*P2RX1*
chr9	96337635	96338308	4	0.00104871	0.048198183	0.01887848	*PHF2*
chr22	20130190	20130698	3	0.001053287	-0.036993476	-0.011985885	*ZDHHC8*
chr3	149094653	149095283	5	0.001058728	-0.098890248	-0.070030312	*TM4SF1*
chr19	52956310	52957180	11	0.001060825	0.049908019	0.011289647	*ZNF578*
chr9	139590572	139591234	3	0.001066035	0.045913517	-0.001331713	*NA*

chr6	117923272	117924280	13	0.00107335	0.035363114	0.009387588	*GOPC*
chr11	5617367	5618408	8	0.001088852	0.081689817	0.059771534	*HBG2;TRIM6;TRIM6-TRIM34*
chr1	177139933	177140609	12	0.001102312	0.020923518	0.006064163	*BRINP2*
chr11	70961596	70962357	6	0.001111247	-0.033682388	-0.009204182	*NA*
chr1	60391699	60392933	14	0.00111231	-0.037363813	-0.016157505	*CYP2J2*
chr17	47840643	47841033	5	0.001129537	-0.008584237	-0.000938637	*FAM117A*
chr19	40732113	40732902	12	0.001129537	0.028131602	0.009266668	*CCNP*
chr15	27127627	27129196	7	0.001130454	-0.026229881	-0.015874937	*GABRA5;GABRB3*
chr12	117317770	117318832	6	0.001130454	0.018569523	0.011933802	*HRK*
chr14	93214468	93215468	12	0.001133307	0.028022854	0.007564009	*LGMN*
chr4	111532679	111533951	10	0.001137943	0.031099841	0.008635239	*NA*
chr17	7283578	7284662	13	0.001153081	-0.061975512	-0.012010693	*TNK1*
chr15	41060822	41062224	11	0.001153081	0.024745236	0.01219701	*C15orf62;DNAJC17*
chr2	242648422	242648761	6	0.001186978	0.039093245	0.003679026	*ING5*
chr1	111742914	111743537	9	0.00119088	0.028731691	0.015504187	*DENND2D*
chr10	129705415	129706313	4	0.001194062	-0.018508233	-0.006230621	*PTPRE*
chr6	33084479	33085063	14	0.001194062	0.070689818	0.030941262	*HLA-DPB2*
chr7	98579466	98579722	4	0.001200851	-0.022814584	-0.007066747	*TRRAP*
chr21	44593642	44593766	3	0.001209692	-0.046808937	-0.027449082	*NA*
chr16	12188962	12189875	5	0.0012164	0.019239541	0.012300341	*SNX29*
chr14	35838051	35838977	5	0.001243266	-0.063159116	-0.031024559	*NA*
chr16	17405193	17406298	3	0.001243266	-0.030466548	-0.021723083	*XYLT1*
chr16	68770944	68772469	15	0.001243266	0.045170495	0.010984283	*CDH1*
chr15	82339920	82340505	5	0.001245845	0.014145559	0.007333251	*MEX3B*
chr11	20625992	20627597	7	0.001248911	0.036748008	0.023880226	*SLC6A5*
chr11	250601	251223	7	0.001249397	0.03175696	0.017833049	*PSMD13*
chr3	71179796	71180415	9	0.001253813	0.021535466	0.011758525	*FOXP1*
chr19	49249932	49250688	9	0.001257384	0.017012078	0.007962556	*IZUMO1*
chr19	55586493	55587193	8	0.001276713	-0.130557883	0.003613019	*EPS8L1*
chr9	4489544	4490325	9	0.001318726	0.074295698	0.01007862	*SLC1A1*
chr6	151646300	151647133	11	0.001318726	0.059713318	0.026168837	*AKAP12*
chr14	105104536	105105634	6	0.001323095	-0.035331525	-0.020090974	*NA*
chr12	110764188	110764634	3	0.001328607	-0.037320923	-0.011115936	*ATP2A2*
chr10	661009	661730	6	0.001343277	0.035682931	-0.000901839	*DIP2C*
chr1	9969424	9970993	17	0.001363066	0.021780164	0.001910691	*CTNNBIP1*
chr5	80594700	80595354	3	0.001364962	-0.033660471	-0.013679408	*CKMT2-AS1;RNU5E-1;ZCCHC9*
chr5	118691007	118692011	13	0.001372127	-0.030669991	-0.010884137	*TNFAIP8*
chr19	17439877	17440072	3	0.001395867	-0.021347187	-0.016886374	*ANO8*
chr4	7638050	7638280	3	0.001402182	-0.025603948	-0.005161488	*SORCS2*
chr11	7041080	7042754	21	0.001417994	0.031085386	0.008154249	*NLRP14;ZNF214*
chr11	32452038	32452839	10	0.001437694	0.034065762	0.015738453	*WT1;WT1-AS*
chr11	17794478	17795033	3	0.001455079	-0.031037868	-0.019858425	*KCNC1*
chr5	140501277	140501812	10	0.00145596	0.044914491	0.022367149	*PCDHB4*
chr8	1320786	1321883	6	0.001471933	-0.095063642	-0.059084693	*NA*
chr1	152485511	152485838	3	0.001479477	-0.025101508	0.001399371	*CRCT1*
chr19	8428144	8429166	12	0.001507038	0.044517797	0.006899286	*ANGPTL4*
chr5	78365647	78366302	8	0.001516028	0.046171115	0.033246165	*BHMT2;DMGDH*
chr1	159915569	159916472	6	0.001541847	-0.035156309	-0.01127555	*IGSF9*
chr19	51917852	51918667	3	0.001549108	-0.029636881	-0.0205819	*SIGLEC10;SIGLEC10-AS1*

chr1	228402036	228402696	3	0.001554646	0.085664851	0.018426752	*OBSCN;OBSCN-AS1*
chr1	20569408	20569715	4	0.001563115	-0.080549439	-0.058098005	*NA*
chr7	149128631	149129312	3	0.001580447	-0.021475404	-0.008590441	*ZNF777*
chr11	45743599	45743936	7	0.001589389	-0.044915667	-0.02143135	*LOC100507384*
chr6	30104295	30104916	8	0.001596261	-0.055779428	-0.020352771	*TRIM40*
chr5	140624586	140625389	12	0.001601501	0.038820961	0.014835788	*PCDHB15*
chr9	134881994	134882463	3	0.001604418	0.065020492	0.030764996	*MED27*
chr16	84327245	84328719	13	0.00162585	-0.022362967	0.000349595	*WFDC1*
chr8	11141355	11141680	4	0.001632438	-0.062878349	-0.042228296	*MTMR9*
chr1	3163341	3164431	7	0.001639848	-0.039660593	-0.02327215	*PRDM16*
chr5	126408756	126409553	13	0.001646382	-0.093147382	-0.040405777	*C5orf63*
chr3	194756646	194757099	4	0.001660021	-0.027794106	-0.025593652	*NA*
chr1	91182215	91182989	9	0.001667798	0.028653038	0.007748176	*BARHL2*
chr20	61001775	61004551	14	0.001677344	-0.048286256	-0.020421233	*RBBP8NL*
chr11	14665739	14666839	7	0.001701595	0.008157008	0.001371377	*PDE3B;PSMA1*
chr8	1496052	1497307	8	0.001716825	-0.055428041	-0.019408406	*DLGAP2*
chr7	101006963	101007603	3	0.001716825	0.042942368	0.01822064	*COL26A1*
chr4	39183160	39183765	4	0.001718108	-0.046261747	-0.018767559	*WDR19*
chr1	19669159	19669527	3	0.001720816	-0.013306625	-0.000705805	*CAPZB*
chr19	58868256	58869424	8	0.001737199	0.043109995	0.021076581	*A1BG;ZNF497*
chr10	84573500	84574204	3	0.001739866	-0.044464398	-0.022427103	*NRG3*
chr10	88294689	88296809	11	0.001750842	-0.021983898	-0.00775802	*NA*
chr17	4486580	4487606	8	0.001754684	-0.090840695	-0.025276342	*SMTNL2*
chr12	6604039	6605071	3	0.001770552	0.048789781	0.038379008	*MRPL51;NCAPD2*
chr4	493489	493833	4	0.001778136	0.062546546	0.025316641	*PIGG;ZNF721*
chr3	122296027	122296613	8	0.001816	-0.02467532	-0.010322068	*PARP15*
chr7	5518392	5519576	5	0.00182064	0.029716171	0.012244613	*FBXL18*
chr16	75241914	75242114	3	0.001824262	-0.049510946	-0.033102665	*CTRB2*
chr15	85177401	85177792	5	0.001878072	0.108014004	0.042748182	*SCAND2P*
chr10	99337734	99338240	6	0.001878334	-0.085936804	-0.045350381	*ANKRD2*
chr6	32972492	32973306	3	0.001884134	-0.026934063	-0.013105409	*HLA-DOA*
chr11	121526264	121526840	5	0.001884134	0.013144484	0.00356213	*NA*
chr18	77838622	77839582	3	0.001897468	0.040688745	0.019948627	*RBFA;RBFADN*
chr16	69353295	69354291	4	0.001900217	-0.029110787	-0.005303071	*VPS4A*
chr17	79950061	79950366	3	0.001907436	-0.030988059	-0.014156942	*ASPSCR1*
chr12	119346746	119347628	6	0.001942179	-0.046723065	-0.009935769	*NA*
chr17	4389722	4389857	5	0.001953314	0.026111188	0.015535389	*SPNS3*
chr6	41715166	41715593	5	0.001966307	-0.024872923	-0.011769477	*PGC*
chr16	70780647	70780947	4	0.001988531	-0.027372925	0.004313405	*VAC14*
chr4	57332196	57333859	14	0.00199873	0.034431596	0.00897944	*SRP72*
chr12	115111557	115112225	3	0.002012181	-0.052455353	-0.039896017	*TBX3*
chr7	45431741	45431891	3	0.002018239	-0.074423894	-0.059182159	*NA*
chr17	19883326	19883716	4	0.00203766	-0.076741563	-0.047474884	*AKAP10*
chr6	31648146	31649638	33	0.002040718	-0.050842321	0.002515958	*LY6G5C*
chr11	4928572	4928760	3	0.002045707	-0.031998507	-0.01593919	*OR51A7*
chr14	77492788	77494451	7	0.002049864	0.023314934	0.008091811	*IRF2BPL*
chr11	117071224	117072280	3	0.002050233	0.064936569	0.028484211	*LOC100652768;TAGLN*
chr3	18391029	18391232	3	0.002050468	0.052997647	0.004060534	*SATB1*
chr6	36953694	36954415	15	0.002065572	-0.007888502	-0.001338873	*MTCH1*

chr6	170589530	170590694	6	0.002087911	-0.087571833	-0.050086546	*NA*
chr7	1362568	1363124	4	0.002089617	-0.043060833	-0.030525251	*NA*
chr2	54949722	54951670	10	0.002117578	0.013102823	0.00248224	*EML6*
chr2	142888868	142889683	9	0.002123649	-0.046981885	-0.017809343	*LRP1B*
chr5	179220545	179221090	6	0.002133156	0.026435678	0.00969012	*LTC4S;MAML1*
chr2	102866879	102867826	5	0.002155469	-0.054397542	-0.024465791	*NA*
chr7	1781918	1782577	6	0.002163865	-0.036220915	-0.022520865	*ELFN1*
chr1	99728691	99730224	13	0.002186804	-0.030506588	-0.006169618	*PLPPR4*
chr3	37494602	37495806	4	0.002190568	0.064009497	0.028554409	*ITGA9*
chr5	1503259	1503979	5	0.002200468	0.023196528	0.013211333	*LPCAT1*
chr17	46799845	46801314	17	0.002250791	-0.026805771	0.001097912	*PRAC1;PRAC2*
chr3	138665654	138666885	16	0.002311434	0.029944443	0.005260513	*FOXL2;FOXL2NB*
chr12	1906835	1906917	3	0.002318874	0.050506493	0.03084608	*CACNA2D4*
chr16	87866696	87866833	3	0.002354426	0.028280383	0.025247623	*SLC7A5*
chr7	42005232	42006120	5	0.002368106	0.041430608	-0.001240974	*GLI3*
chr4	89641534	89642483	3	0.00237717	-0.024620618	-0.01476934	*FAM13A-AS1*
chr18	30352297	30353289	11	0.002389582	0.037248837	0.009405153	*KLHL14*
chr7	7142996	7143299	3	0.002394875	-0.041653245	-0.023100124	*NA*
chr4	1957019	1957864	8	0.002408165	-0.027733054	-0.005596601	*NSD2*
chr4	108745279	108746407	14	0.002431079	-0.019682963	-0.000650699	*SGMS2*
chr16	71264282	71265020	10	0.00246605	0.043570391	-0.001114092	*HYDIN*
chr9	34662773	34663048	4	0.002495743	-0.030415227	-0.012161588	*CCL27*
chr1	184020263	184021360	13	0.002506107	0.017304859	-0.001154007	*TSEN15*
chr12	107381075	107381862	6	0.002523663	-0.042674641	-0.01377838	*MTERF2*
chr7	28530206	28530724	5	0.002523663	-0.024851504	-0.007643879	*CREB5*
chr19	58125386	58126053	15	0.002523663	-0.021767054	-0.00045875	*ZNF134*
chr17	33759512	33760819	14	0.00253227	-0.061075502	-0.033207477	*SLFN12*
chr15	33487289	33487681	4	0.002536214	-0.081399452	-0.052479103	*FMN1*
chr6	74103959	74105065	13	0.002553005	-0.062963695	-0.019460486	*DDX43;OOEP*
chr6	106035279	106035528	4	0.002568784	-0.039063244	-0.019882899	*NA*
chr9	14346276	14346834	3	0.002568784	-0.037172128	-0.011359188	*NFIB*
chr2	70322323	70322659	4	0.002568784	0.020058998	0.009792506	*NA*
chr10	35102505	35103615	4	0.002577536	0.018435627	0.001148596	*PARD3*
chr3	184971652	184972683	10	0.002579487	0.039299528	0.016326984	*EHHADH*
chr15	27113322	27113913	4	0.002579487	0.063871275	0.023539822	*GABRA5;GABRB3*
chr3	185911208	185911885	6	0.002613129	0.060760176	0.022498292	*DGKG*
chr1	234040765	234041845	6	0.002616569	-0.050642386	-0.00847548	*SLC35F3*
chr12	49391363	49392438	6	0.002623057	0.010821612	-0.00065394	*DDN*
chr16	4103161	4103533	5	0.002624403	0.037020938	0.007489774	*ADCY9*
chr8	144098507	144099299	5	0.002635355	-0.074100443	-0.020161407	*LY6E;LY6E-DT*
chr11	17740977	17741925	7	0.00264705	0.021855978	0.015045084	*MYOD1*
chr1	180202256	180202784	3	0.002652502	0.035000405	-0.000671264	*LHX4*
chr1	179544476	179545091	5	0.002676805	-0.051758503	-0.016883734	*NPHS2*
chr16	31499479	31500246	5	0.002703545	0.037171419	0.021594685	*SLC5A2*
chr4	6152248	6152765	6	0.002750165	0.027528217	0.00142923	*JAKMIP1*
chr2	242051650	242052583	8	0.00275143	-0.029936475	-0.015155449	*PASK*
chr14	102705529	102705945	3	0.002770743	0.059298628	0.053823493	*MOK*
chr6	149806273	149806659	7	0.002787686	-0.051098521	-0.03077694	*ZC3H12D*
chr3	42121652	42121982	3	0.002788661	-0.062039564	-0.043794602	*NA*

chr12	133344446	133345322	5	0.002813161	0.017463009	0.013880908	*NA*
chr1	175161785	175162553	15	0.002854544	-0.033157119	-0.009448434	*KIAA0040*
chr19	39997622	39998290	5	0.002866178	-0.072860783	-0.050773067	*DLL3*
chr9	37651177	37651660	3	0.00286852	-0.039109787	-0.015340723	*FRMPD1*
chr13	65532581	65533139	5	0.002875524	-0.024941991	-0.016220884	*NA*
chr19	50372937	50373420	4	0.002875524	0.008289635	-0.000198575	*AKT1S1;PNKP*
chr3	170137240	170138205	6	0.002897129	-0.035207675	-0.010550451	*CLDN11*
chr2	3646077	3646196	3	0.002898292	0.045316141	0.038271285	*COLEC11*
chr8	65290848	65292217	12	0.002898443	0.029720691	0.000398324	*MIR124-2;MIR124-2HG*
chr11	890192	890505	4	0.002976531	0.106259545	0.023511924	*CHID1*
chr8	65489554	65490343	10	0.003004525	0.013017972	0.004399113	*BHLHE22;LOC401463*
chr19	17411666	17412305	5	0.003006734	-0.025796937	-0.007928678	*ABHD8;MRPL34*
chr5	88178977	88179867	16	0.003018479	-0.020952961	0.003801565	*MEF2C*
chr5	95066236	95066845	10	0.003043629	0.046874701	0.00626219	*RHOBTB3*
chr4	142053623	142054254	5	0.003053176	0.009581354	0.006798507	*RNF150*
chr6	28583655	28584464	14	0.00306135	-0.045641836	-0.02152185	*NA*
chr3	30936070	30936955	11	0.003066031	0.033844485	0.004999254	*GADL1*
chr16	10912331	10912718	5	0.003086984	-0.054164406	-0.025788253	*TVP23A*
chr19	51357343	51357857	4	0.003102889	-0.063073745	-0.013893822	*KLK3*
chr3	12597236	12598043	5	0.003123489	-0.051563	-0.019747092	*MKRN2*
chr10	121651945	121652836	11	0.003163557	0.025912423	0.001690502	*SEC23IP*
chr16	3136857	3137690	5	0.003184304	0.041154758	0.022083672	*NA*
chr6	116381904	116382179	5	0.003194724	-0.044808982	-0.035183201	*FRK*
chr6	13614902	13616043	12	0.003202223	0.018179154	-1.09E-05	*NOL7;SIRT5*
chr15	64680186	64680904	3	0.003226168	-0.01793594	-0.002972235	*TRIP4*
chr1	50573970	50574837	9	0.003237914	-0.03230689	-0.01796085	*ELAVL4*
chr19	2546598	2547067	4	0.003406341	0.055456339	0.032472557	*GNG7*
chr9	130860206	130860839	7	0.003443369	0.028573755	0.015608212	*SLC25A25*
chr9	27529519	27530279	4	0.00349332	0.027615796	0.007079247	*MOB3B*
chr17	38231199	38232128	3	0.003517339	-0.01760121	0.001649538	*THRA*
chr10	99330963	99331808	5	0.003583543	-0.018700832	-0.010774525	*ANKRD2*
chr3	26665477	26666855	7	0.003584525	-0.038348595	-0.008544736	*LRRC3B*
chr7	107770791	107771214	5	0.003594348	-0.029990151	0.004813952	*LAMB4*
chr19	15197635	15198451	4	0.003595514	-0.027706224	-0.007921118	*OR1I1*
chr11	43603572	43604219	4	0.003652476	0.036642728	0.01580651	*NA*
chr1	11561489	11562134	7	0.003652936	-0.061594065	-0.033587914	*DISP3*
chr2	95536859	95537475	7	0.0036952	-0.069979661	-0.035950232	*LOC442028;TEKT4*
chr2	85554513	85555559	10	0.003706117	-0.019746039	0.00187171	*TGOLN2*
chr11	64691547	64692208	13	0.003717165	0.020373975	0.002532005	*PPP2R5B*
chr10	124638756	124639782	15	0.003812861	0.0498565	0.016000113	*C10orf88B;FAM24B;FAM24B-CUZD1*
chr4	644572	644973	3	0.003812861	0.047764147	0.023940578	*PDE6B*
chr16	86958417	86959212	3	0.003865907	-0.01717986	0.000422619	*NA*
chr11	128554586	128555529	8	0.003869365	0.029598278	0.01950198	*FLI1*
chr17	72462417	72463080	9	0.003876678	0.033966784	0.019183738	*CD300A*
chr16	88769854	88770361	7	0.003907436	0.009222582	0.004587498	*CTU2;RNF166*
chr20	21377671	21378074	4	0.00394394	0.047083951	0.027139874	*NKX2-4*
chr19	49576955	49577303	5	0.003964147	-0.044339038	-0.026621625	*KCNA7*
chr7	1051703	1052392	3	0.003986234	-0.095240725	-0.064662071	*C7orf50*
chr18	31803067	31804000	11	0.004008932	0.024733603	0.010416823	*NOL4*

chr2	242710665	242711599	6	0.004008932	0.091587079	0.038733586	*GAL3ST2*
chr14	65005951	65006359	10	0.004009524	-0.043468002	-0.011212329	*HSPA2*
chr4	1041664	1042767	4	0.004030686	-0.042002251	-0.017103511	*NA*
chr19	54566838	54567279	6	0.004052166	0.058215176	0.033963481	*VSTM1*
chr8	6663401	6664281	6	0.004083566	-0.023555624	-0.006704318	*NA*
chr1	23695347	23696579	15	0.004097561	0.020788375	0.004374452	*ZNF436;ZNF436-AS1*
chr1	220445206	220446194	12	0.004114438	-0.026362913	-0.010749176	*AURKAP1;RAB3GAP2*
chr11	71951714	71952431	4	0.004117251	0.017336578	0.007040154	*PHOX2A*
chr17	1618295	1619022	4	0.004171027	-0.040526323	-0.008814815	*MIR22;MIR22HG;TLCD2;WDR81*
chr1	108743318	108743879	7	0.004177908	0.017091611	0.008251629	*SLC25A24*
chr12	91331583	91332479	6	0.004190962	-0.017986572	0.001778265	*LINC00615*
chr11	315751	316339	5	0.004215875	-0.043915868	-0.034649014	*NA*
chr1	1982434	1983033	4	0.004215875	-0.059490018	-0.033915493	*PRKCZ*
chr10	83636072	83636782	5	0.004254915	0.013803638	0.003229175	*NRG3*
chr14	64804611	64804911	3	0.004274143	0.022924336	0.010826088	*ESR2*
chr11	366307	366487	4	0.004309681	-0.042498177	-0.021908206	*B4GALNT4*
chr5	52775681	52776694	9	0.004309681	-0.024535669	-0.000858046	*FST*
chr14	23291485	23292648	11	0.004342542	0.054978476	0.005600843	*SLC7A7*
chr5	140762229	140762582	7	0.004361268	-0.042796212	-0.022490183	*CDHGA4;PCDHGA5;PCDHGA6;PCDHGA7;PCDHGA8;PCDHGB1;PCDHGB2;PCDHGB3;PCDHGB4*
chr2	240698166	240698489	3	0.004376293	-0.024867589	-0.01182142	*LOC150935*
chr3	186284448	186285036	5	0.004376293	0.007976935	0.001550201	*DNAJB11;TBCCD1*
chr12	65562873	65563900	10	0.004376802	0.010332217	-3.92E-05	*LEMD3*
chr6	134373485	134373925	6	0.004383722	0.011210654	0.006041139	*SLC2A12*
chr9	139877234	139878174	6	0.004387693	-0.021283662	-0.011799128	*LCNL1*
chr4	103997887	103998717	11	0.004387693	-0.051891655	-0.009225944	*SLC9B2*
chr4	99064102	99064603	6	0.004395668	-0.081138918	-0.027481411	*STPG2*
chr17	50237205	50237844	15	0.00442872	0.044866384	0.016865056	*CA10*
chr20	44803246	44803633	3	0.004445931	-0.02702401	-0.008209174	*CDH22*
chr2	183731407	183732357	10	0.004453116	0.020805806	0.000707076	*FRZB*
chr20	2632942	2633873	12	0.004454235	-0.058745536	-0.003657654	*MIR1292;NOP56;SNORA51;SNORD110*
chr17	72731453	72731753	3	0.004456989	0.036880802	0.029296701	*RAB37*
chr1	233463492	233464510	7	0.004466128	0.009839634	0.003222927	*MAP3K21*
chr1	154842717	154843314	9	0.004471851	0.019142879	0.00574382	*KCNN3*
chr12	82153008	82153736	11	0.004476132	0.058978858	0.01303392	*PPFIA2*
chr14	52183331	52183988	5	0.004481274	-0.039103153	0.004076741	*FRMD6*
chr2	84743142	84743935	12	0.004504197	-0.035550187	-0.018328035	*DNAH6*
chr11	2210061	2210306	3	0.004514061	0.023059414	0.01763325	*NA*
chr8	132052044	132053262	10	0.004515912	0.031486449	0.014680511	*ADCY8*
chr1	1003126	1003851	6	0.004534368	-0.053735469	-0.028303476	*NA*
chr5	140220032	140221364	10	0.004549587	0.060112406	0.027888149	*A1;PCDHA2;PCDHA3;PCDHA4;PCDHA5;PCDHA6;PCDHA7;PCDHA8*
chr6	10320036	10320712	3	0.004651092	-0.025212357	-0.018124946	*NA*
chr19	33716915	33718148	8	0.004687001	0.032211368	0.007164889	*SLC7A10*
chr21	45758982	45759752	13	0.004706744	-0.022968222	0.000135186	*CFAP410*
chr7	97755534	97755792	6	0.004721056	0.031414966	0.021053046	*LMTK2*
chr16	30006637	30007613	16	0.004725405	0.02080049	0.001724967	*BOLA2;HIRIP3;INO80E*
chr3	102474183	102474265	3	0.004741578	-0.029204002	-0.016863738	*NA*
chr15	89904655	89905516	8	0.004786496	0.057353734	0.010029496	*NA*
chr6	50803820	50804174	6	0.004818141	0.018408365	0.013944767	*TFAP2B*
chr8	11557217	11557906	3	0.004837798	0.030963021	0.012765526	*GATA4*

chr6	32375672	32376095	3	0.004838997	-0.108409285	-0.073937555	*BTNL2*
chr5	149889677	149890075	3	0.004860068	-0.045695361	-0.009101225	*NDST1*
chr21	45594765	45595361	3	0.004871492	-0.037780979	-0.029393884	*NA*
chr4	71493251	71494332	4	0.004909623	-0.043748134	-0.012038553	*ENAM*
chr8	1588618	1589365	5	0.004982806	-0.034457675	-7.30E-05	*DLGAP2*
chr2	242448543	242449352	12	0.00499031	0.035228203	0.010287472	*STK25*
chr4	9382765	9383381	3	0.005020852	-0.064145684	-0.02924268	*NA*
chr4	165304259	165304531	6	0.00502775	0.016246327	0.004301536	*MARCHF1*
chr17	76354621	76355288	6	0.005058801	-0.060648509	-0.020873668	*SOCS3*
chr15	67706106	67706575	3	0.005059265	0.027431134	0.006947341	*IQCH;IQCH-AS1*
chr8	110703888	110704800	9	0.005087341	0.046838364	0.017403339	*SYBU*
chr4	55967	56185	4	0.005103273	0.041469809	0.029241498	*ZNF595;ZNF718*
chr2	241807859	241808757	8	0.00511816	-0.035852353	-0.014899495	*AGXT*
chr18	66381343	66381799	3	0.005131028	-0.037727289	-0.021878348	*CCDC102B;TMX3*
chr22	41215824	41216528	6	0.005135055	-0.025500509	-0.002368177	*SLC25A17*
chr13	107026684	107027491	3	0.00513818	-0.027180011	-0.006983413	*LINC00460*
chr15	45670478	45671347	15	0.005165023	-0.031133945	-0.012667997	*GATM*
chr6	32904074	32904889	6	0.00517994	0.049714925	0.027762702	*HLA-DMB*
chr19	47017048	47017629	3	0.005192812	-0.043291309	-0.026549693	*PPP5D1*
chr20	48701877	48702589	3	0.005192812	-0.025928419	0.002761461	*PEDS1-UBE2V1;UBE2V1*
chr20	3219539	3220034	4	0.00521412	-0.014907	-0.002732856	*SLC4A11*
chr14	70883772	70884256	8	0.00521412	-0.016239706	0.001655373	*SYNJ2BP;SYNJ2BP-COX16*
chr6	74354803	74355165	3	0.005234063	-0.040420801	-0.027303246	*SLC17A5*
chr12	89744150	89745673	16	0.005255055	0.034803223	0.006669881	*DUSP6*
chr6	33135269	33137260	18	0.005266361	0.044738538	-0.004743933	*COL11A2*
chr5	140419819	140420926	8	0.005286084	0.044494447	0.022082951	*NA*
chr8	4849827	4851008	5	0.005311281	0.034284116	0.009857911	*CSMD1*
chr18	77138860	77139296	5	0.00533352	-0.054848703	-0.028153853	*NA*
chr10	3456947	3457368	4	0.005341226	0.030548862	-0.000150283	*NA*
chr6	168814631	168815615	4	0.005377696	-0.024584427	-0.017568257	*NA*
chr1	173174650	173174920	6	0.005428215	0.01881669	0.011989063	*TNFSF4*
chr2	172377802	172378791	11	0.005486443	0.037624317	0.012982898	*CYBRD1*
chr13	84456127	84457882	15	0.005489511	0.065601981	0.008012915	*SLITRK1*
chr15	25326233	25326510	3	0.005509707	-0.035983871	-0.009290716	*PW;SNORD116-15;SNORD116-16;SNORD116-18;SNORD116-19*
chr2	239072961	239073223	3	0.00551389	0.041206592	0.027531741	*NA*
chr11	60679546	60681709	17	0.005583394	0.034517054	0.006013295	*TMEM109*
chr11	2889875	2891118	32	0.005590722	0.052979793	0.012448706	*KCNQ1DN*
chr15	66790643	66791435	3	0.005608234	-0.032270484	-0.008341683	*SNAPC5*
chr1	25756455	25757604	15	0.005624996	0.008595257	0.002390471	*MACO1;RHCE*
chr5	161277234	161277813	3	0.005672956	0.052645254	0.027278376	*GABRA1*
chr6	31689459	31689996	3	0.005676964	-0.031006414	-0.020223107	*LY6G6C;MPIG6B*
chr20	238136	238452	4	0.005681791	-0.066365835	-0.019784762	*DEFB132*
chr2	105655035	105655370	5	0.005710882	-0.024056264	-0.010241168	*MRPS9*
chr1	45671361	45671665	3	0.005796373	0.035628491	0.011760024	*ZSWIM5*
chr1	100434874	100436069	21	0.00581573	0.032783374	-0.002431447	*SLC35A3*
chr2	172955512	172955930	3	0.00581573	0.037162953	0.017429808	*NA*
chr1	214158373	214159589	8	0.005819828	-0.022799782	-1.78E-05	*PROX1*
chr7	107301566	107302364	6	0.005831466	-0.017102524	-0.002554516	*SLC26A4;SLC26A4-AS1*
chr4	57371022	57372597	10	0.00587179	-0.035265391	-0.013632803	*ARL9*

chr6	33970123	33970919	3	0.005898216	0.049961865	0.028963591	*MIR1275*
chr1	175712871	175713248	4	0.005930363	-0.081148933	-0.01897398	*TNR*
chr19	45898849	45899615	4	0.006017028	0.049807267	0.022210629	*PPP1R13L*
chr6	108439752	108440339	3	0.006042206	0.015620156	-0.002639939	*NA*
chr8	144161874	144162802	4	0.006055441	0.030823897	0.02258749	*NA*
chr14	77193902	77194291	3	0.006068875	-0.055972883	-0.014502957	*NA*
chr5	170288255	170289430	14	0.006098111	0.066969839	0.018759563	*RANBP17*
chr3	183897878	183898017	3	0.006138724	-0.027762518	-0.009520148	*AP2M1*
chr16	30381677	30382569	6	0.00617204	0.012151426	0.003609281	*MYLPF;TBC1D10B*
chr8	52811374	52812185	12	0.006271688	-0.007818582	0.000932948	*PCMTD1*
chr4	113970506	113971324	8	0.006271688	0.05731554	0.019197927	*ANK2*
chr6	110720501	110721629	6	0.00628265	-0.117068834	-0.060612752	*DDO;METTL24*
chr19	6590801	6591674	8	0.006290099	0.045351389	0.009261382	*CD70*
chr1	86621931	86622737	7	0.006370148	0.017211316	0.001201046	*COL24A1*
chr3	149052364	149053027	4	0.006392388	-0.032930607	-0.017506719	*TM4SF18*
chr6	32054561	32055146	13	0.006396527	0.043888213	0.009446402	*TNXB*
chr22	27620801	27621065	3	0.006421511	-0.067126449	-0.048630375	*NA*
chr4	109540778	109542144	16	0.006421511	-0.026888884	-0.000596232	*RPL34;RPL34-DT*
chr1	2138953	2139657	7	0.00644383	0.020332909	-0.002610524	*FAAP20*
chr12	13196820	13197550	10	0.006474826	-0.067462656	-0.018505793	*FAM234B*
chr11	74950764	74951359	4	0.006484558	-0.032204574	-0.025957283	*TPBGL*
chr14	51562097	51563131	13	0.006490729	-0.018956419	0.004581761	*TRIM9*
chr1	244541634	244541771	3	0.006495434	-0.030164992	-0.008893449	*C1orf100*
chr5	77142469	77143258	3	0.006499626	-0.123445602	-0.097823751	*NA*
chr6	32940489	32943025	28	0.006541649	0.036214195	0.008551458	*BRD2*
chr7	79083054	79084166	16	0.006562045	0.029192154	0.006160508	*MAGI2;MAGI2-AS3*
chr2	189434064	189434667	3	0.006583822	0.035977892	0.010756981	*GULP1*
chr1	110090242	110091625	17	0.006590537	-0.01062039	-0.001033664	*GNAI3*
chr3	113234510	113235258	4	0.006618541	-0.073530954	-0.052235463	*SPICE1*
chr14	67655758	67656241	4	0.006618541	0.043519492	0.030081512	*FAM71D*
chr16	1251787	1252484	6	0.006647928	-0.040933623	-0.00972918	*CACNA1H*
chr21	40751816	40753285	14	0.006650921	-0.038995194	0.004115166	*GET1*
chr3	101220713	101220960	3	0.006663196	-0.148146899	-0.066753757	*SENP7*
chr12	132588323	132588637	4	0.006677384	0.033015274	0.001481158	*EP400P1*
chr10	32218901	32219402	3	0.00668813	0.014188392	0.00946163	*ARHGAP12*
chr5	143978290	143978703	3	0.006695377	-0.058581316	-0.037606546	*NA*
chr3	190610335	190611184	4	0.006702772	-0.02435468	-0.013923838	*NA*
chr7	4456202	4456592	6	0.006726474	-0.061570074	-0.037827576	*NA*
chr8	145011406	145012068	3	0.006732496	-0.031574798	-0.012371998	*PLEC*
chr11	76838498	76838787	6	0.006735704	0.024242395	0.012743628	*MYO7A*
chr19	8650112	8650523	3	0.006751258	-0.021172488	-0.003909626	*ADAMTS10*
chr19	47922909	47923683	5	0.006820446	0.015573656	0.009047243	*MEIS3*
chr1	1897585	1897785	3	0.006821158	-0.043205244	-0.034129611	*CFAP74*
chr12	120031637	120033155	12	0.006837354	0.027773847	0.00767214	*TMEM233*
chr17	34841934	34842453	9	0.006910765	0.015721642	0.002355346	*ZNHIT3*
chr14	75761849	75762018	3	0.006950146	0.012661214	0.00922658	*LINC01220*
chr17	684490	685151	5	0.006954041	-0.024740021	-0.002657617	*GLOD4;MRM3*
chr19	1174154	1175076	10	0.006962104	0.037417903	0.008741477	*SBNO2*
chr6	28549832	28550437	6	0.006962471	0.045059567	0.00505332	*ZBED9*

chr16	89283569	89284213	8	0.006966781	-0.011703005	-0.001344257	*ZNF778*
chr2	119067579	119068497	7	0.006980591	-0.052992321	-0.00644702	*NA*
chr17	35014412	35014676	4	0.006990471	0.01367516	0.000877834	*NA*
chr12	47219626	47220197	13	0.006995976	-0.066429753	-0.033130735	*SLC38A4*
chr1	214776286	214776904	11	0.007027542	-0.022844092	-0.008724308	*CENPF*
chr4	141294522	141295361	12	0.007027542	0.014920616	0.001611148	*SCOC;SCOC-AS1*
chr17	77375343	77375711	4	0.007041813	-0.03969181	-0.024896794	*RBFOX3*
chr18	20839953	20840464	3	0.007067553	0.015435061	0.013473234	*CABLES1;TMEM241*
chr5	172198876	172199667	16	0.007092013	0.012289409	0.001232242	*DUSP1*
chr7	95225339	95225681	3	0.007105052	-0.040672315	-0.02989576	*PDK4*
chr3	46918266	46918866	3	0.007108622	-0.027814951	-0.024826294	*PTH1R*
chr17	42467199	42468131	5	0.007144335	-0.044023589	-0.012525804	*ITGA2B*
chr14	77925696	77925988	4	0.007173674	0.024595503	0.012604911	*AHSA1;VIPAS39*
chr10	115311837	115312553	4	0.007181405	-0.050938071	-0.015106717	*HABP2*
chr17	27920051	27920934	11	0.007185449	-0.019600148	-0.00453838	*ANKRD13B;GIT1*
chr16	2770814	2771425	5	0.007186416	0.026949326	0.007327735	*PRSS27*
chr13	95086046	95086170	4	0.007198065	-0.060388775	-0.038905364	*NA*
chr17	19619905	19620595	5	0.007198065	-0.0314185	-0.014389828	*SLC47A2*
chr3	193852690	193853465	16	0.007198065	0.020899197	0.001752382	*HES1*
chr11	128562003	128562408	7	0.007198065	-0.028371339	0.004777096	*FLI1;SENCR*
chr2	102927278	102927898	5	0.007242051	0.028558027	0.016871457	*IL18R1;IL1RL1*
chr16	88738320	88738418	3	0.007264418	-0.033312325	-0.003132671	*SNAI3-AS1*
chr17	43098904	43099567	5	0.007276817	0.042636215	0.007754218	*NA*
chr10	636076	636390	4	0.007334214	-0.030318028	-0.014719459	*DIP2C*
chr4	2061325	2061930	7	0.007334214	-0.020687651	-0.007004789	*NAT8L*
chr6	5145569	5146008	3	0.007363238	0.049203057	0.021878645	*LYRM4*
chr10	25239164	25239957	4	0.007370663	0.029373979	-0.003311175	*PRTFDC1*
chr7	73921925	73922494	4	0.007458279	-0.031366561	-0.012379605	*GTF2IRD1*
chr13	50702410	50703026	7	0.007467181	-0.033421664	-0.01833188	*DLEU1;DLEU2*
chr8	145638434	145639181	6	0.007555007	-0.040444903	-0.029975849	*CPSF1;SLC39A4*
chr9	116263261	116263669	4	0.007616366	0.041295797	0.011802043	*RGS3*
chr4	57522094	57523055	10	0.007619505	0.020789395	0.001476444	*HOPX*
chr12	46663407	46664096	5	0.007742665	-0.053845595	-0.030892415	*SLC38A1*
chr8	8859549	8860465	11	0.007742665	-0.036094604	-0.008578133	*ERI1*
chr9	114359776	114360581	3	0.007742665	0.035233697	0.011929221	*PTGR1*
chr15	85201039	85201545	5	0.007798484	-0.053147654	-0.010465225	*NMB;WDR73*
chr17	7350001	7350413	4	0.007799936	0.033047113	0.02750645	*CHRNB1*
chr3	63087235	63087266	3	0.007811409	-0.038811079	-0.012561661	*LINC00698*
chr10	134116395	134116651	4	0.007811409	-0.025403973	0.000935354	*STK32C*
chr14	105864312	105864896	8	0.007824226	0.013689141	0.001276876	*PACS2;TEX22*
chr2	177012117	177012562	5	0.007832782	0.025002623	0.015626947	*HOXD4;MIR10B*
chr1	879375	879958	4	0.007862775	-0.033930356	-0.014483998	*NOC2L;SAMD11*
chr17	1733433	1733982	5	0.007893587	0.025977235	0.009624899	*RPA1;SMYD4*
chr16	2535757	2536153	3	0.007917915	0.021780756	0.008074512	*TBC1D24*
chr10	28784890	28785155	4	0.007958826	-0.058116499	-0.031497726	*NA*
chr1	114696350	114697113	14	0.008072158	0.015831444	0.004603126	*SYT6*
chr2	171674179	171675106	11	0.008109807	0.022623758	0.007963071	*GAD1*
chr17	45855265	45855739	3	0.008112544	-0.030965828	-0.020276865	*NA*
chr17	27333108	27333769	13	0.008201845	0.030160075	0.010886274	*SEZ6*

chr17	76100646	76101391	7	0.008203637	-0.029813631	-0.013399489	*TNRC6C*
chr11	107583339	107583988	4	0.008265063	-0.074982764	-0.021792016	*SLN*
chr1	67395544	67396577	11	0.008265063	-0.01406583	0.000702571	*DNAI4;MIER1*
chr9	86151648	86152249	3	0.008365529	0.037926763	0.010678453	*FRMD3*
chr3	125690774	125691192	3	0.008378925	0.023008607	0.012009073	*ROPN1B*
chr17	77710631	77710872	3	0.008384552	-0.028688027	-0.018441304	*ENPP7*
chr1	170501645	170501864	4	0.008388362	0.034921968	0.011543347	*GORAB*
chr10	129923959	129924922	12	0.008417638	0.017531545	0.001866695	*MKI67*
chr1	2250136	2250860	5	0.008429912	-0.026929741	-0.010190902	*NA*
chr2	74006665	74007064	6	0.008454113	0.019731387	0.004174873	*C2orf78;DUSP11*
chr8	20054372	20054715	12	0.008551663	0.048215446	0.009263481	*ATP6V1B2*
chr2	106015518	106016014	9	0.008557799	0.041743193	0.011785218	*FHL2*
chr11	68181218	68181383	3	0.008574902	-0.023178523	-0.009533803	*LRP5*
chr6	41068553	41069048	6	0.008589893	-0.112143281	-0.0678435	*ADCY10P1;NFYA*
chr19	36499326	36499721	3	0.00861381	-0.032475749	-0.021025015	*SYNE4*
chr22	42828125	42828447	6	0.008746821	0.018397292	0.010579405	*NFAM1*
chr11	116969404	116970002	7	0.00876941	-0.033830626	-0.004695877	*SIK3*
chr22	44394166	44394903	6	0.008815437	-0.017414146	-0.000662331	*PARVB*
chr4	178363522	178364342	7	0.008815437	0.030570228	0.007022384	*AGA*
chr10	134983383	134984329	6	0.008826052	-0.039175805	-0.025669852	*KNDC1*
chr8	11664134	11664728	4	0.008826052	-0.070292649	-0.012709491	*FDFT1*
chr18	77905298	77905947	8	0.008827479	0.067158985	0.046388903	*PARD6G-AS1*
chr1	38230948	38231980	7	0.008899921	0.017826307	0.004492959	*EPHA10*
chr16	55357253	55357776	5	0.008930842	0.045770328	0.01033432	*IRX6*
chr19	4455316	4456134	7	0.008961835	0.032583413	-3.61E-05	*UBXN6*
chr3	126134977	126135211	3	0.008965786	-0.058647566	-0.042257145	*CFAP100*
chr8	82542806	82543618	6	0.009107319	0.015286997	-0.001204227	*NA*
chr1	18432400	18433192	4	0.009112341	0.038772446	0.01754024	*IGSF21*
chr8	126479070	126479143	3	0.009186772	0.065862825	0.030800754	*NA*
chr19	39523158	39524492	8	0.0092096	-0.041696455	-0.020908411	*FBXO27*
chr17	46711035	46711341	3	0.0092096	-0.067493666	-0.019296673	*MIR196A1*
chr16	52641156	52641952	4	0.009223835	-0.041942587	-0.008790756	*CASC16*
chr15	49338748	49339393	11	0.009232217	-0.033398521	-0.003906749	*SECISBP2L*
chr5	1241224	1241958	5	0.009411134	0.041001849	0.017223429	*SLC6A18*
chr16	85045118	85045600	9	0.009433081	-0.02216418	-0.001591457	*ZDHHC7*
chr10	104181982	104182490	3	0.009433081	0.023923019	0.014874846	*FBXL15;PSD*
chr17	26699169	26699551	4	0.009569837	-0.027674031	-0.011870997	*SARM1;VTN*
chr1	16302707	16303299	5	0.00958539	-0.055747359	-0.017935787	*ZBTB17*
chr4	41306412	41306578	3	0.009601541	0.06504899	0.024118394	*NA*
chr4	128553912	128554327	8	0.009604976	0.011809563	0.003916146	*INTU*
chr11	9780844	9781231	3	0.009619987	-0.0500783	-0.026293673	*LINC02709;SBF2-AS1*
chr10	118030848	118031654	6	0.009619987	0.026378052	0.008334862	*GFRA1*
chr19	45664198	45664763	5	0.009622222	-0.041377343	-0.018658825	*NKPD1*
chr1	10270439	10270735	7	0.00964953	0.02469445	0.000655343	*KIF1B*
chr1	55229957	55230822	12	0.009682823	-0.051601506	-0.002553655	*PARS2*
chr3	111717557	111718457	13	0.009691659	0.03281121	0.010744028	*TAGLN3*
chr4	81109888	81110459	3	0.009695461	0.040545418	0.035019003	*PRDM8*
chr17	39471940	39472552	8	0.009698844	0.030438724	0.003945027	*KRTAP17-1*
chr11	61129864	61130361	7	0.009750703	-0.011294401	0.002583849	*CYB561A3;TMEM138*

chr1	234367145	234367586	5	0.009770654	-0.084701222	-0.058233636	*SLC35F3*
chr9	123605229	123606119	9	0.00979805	-0.040929159	-0.023044857	*CUTALP;PSMD5*
chr19	56098480	56098985	5	0.009833376	-0.015019094	-0.003073738	*NA*
chr16	29821029	29821173	3	0.009851787	-0.030875229	-0.015267295	*BOLA2;MAZ;PRRT2*
chr2	38603378	38603784	7	0.009855689	-0.008885176	0.000385589	*ATL2*
chr6	29944403	29945728	19	0.009862828	-0.033496424	-0.00577272	*HCG9;HLA-G;HLA-H;HLA-J*
chr19	40909588	40910138	5	0.009869125	-0.026180962	-0.00443123	*PRX*
chr4	39529342	39530224	13	0.009869125	0.016707911	0.00287834	*UGDH;UGDH-AS1*
chr6	170554795	170555276	4	0.009870671	-0.048895717	-0.040768462	*NA*
chr10	134755955	134756707	6	0.009892213	0.037908745	0.024897675	*CFAP46*
chr1	29448625	29449513	8	0.009895761	-0.02685944	-0.00697139	*TMEM200B*
chr7	99953465	99954465	6	0.009915429	0.02089436	0.010134323	*PILRB*
chr12	117593468	117593728	4	0.009942132	-0.026912178	-0.008586821	*FBXO21*
chr22	51176117	51176734	7	0.009973586	-0.026169564	-0.010545283	*ACR*
chr16	29973734	29973951	4	0.010011235	0.012312301	0.000952311	*BOLA2;TMEM219*
chr7	96632294	96633060	6	0.01001561	0.0197594	0.009066553	*DLX6;DLX6-AS1*
chr2	71221537	71222556	15	0.010057563	-0.015522503	4.00E-05	*TEX261*
chr11	111848326	111848638	3	0.010086145	0.029228729	0.020642746	*DIXDC1*
chr2	154336045	154336401	5	0.010090827	-0.044318261	-0.026242754	*RPRM*
chr14	105707628	105708255	3	0.010100826	0.042149375	0.006359199	*BRF1*
chr7	946657	947151	3	0.010249903	-0.041437874	-0.019272074	*ADAP1*
chr16	78179497	78179640	4	0.010258443	-0.026623207	-0.00777838	*WWOX*
chr9	123964477	123964904	7	0.01026638	-0.034532206	-0.010796552	*GSN;RAB14*
chr3	42726640	42727489	9	0.01026638	-0.039336452	0.000364685	*KLHL40*
chr15	89902185	89902570	3	0.01028954	-0.029413948	-0.010083903	*NA*
chr6	168785320	168785970	8	0.010351399	0.03496718	0.008525825	*NA*
chr16	84177597	84178213	4	0.010392524	0.013238461	0.006432043	*DNAAF1;HSDL1*
chr10	133204161	133204297	3	0.010424619	-0.011062142	-0.005211896	*NA*
chr16	17107610	17107812	3	0.010488997	0.020233766	0.014322843	*NA*
chr12	131142316	131142708	4	0.010507192	-0.06076567	-0.022254011	*RIMBP2*
chr3	191216647	191217102	5	0.010571556	0.025598011	-0.005306925	*NA*
chr11	78285542	78286346	12	0.010633132	0.021637751	0.00618006	*NARS2*
chr13	26042382	26043165	6	0.010675761	-0.031263694	0.002778829	*ATP8A2*
chr17	80875493	80876372	6	0.010696619	0.039417749	0.015387103	*TBCD*
chr16	85397637	85398190	3	0.010698032	-0.032249342	-0.009472887	*NA*
chr12	103358649	103358881	5	0.010713598	0.023648649	0.010368554	*NA*
chr14	26674046	26674563	7	0.010778599	-0.010978425	-0.00038186	*NA*
chr7	4901337	4902050	10	0.010778599	0.048442841	0.024197348	*PAPOLB;RADIL*
chr4	84519218	84519514	4	0.010847985	-0.029237137	-0.004164346	*GPAT3*
chr6	43483382	43483748	4	0.010912605	0.03582175	0.013443802	*POLR1C;YIPF3*
chr1	15885816	15886024	4	0.010922952	0.033783607	-0.000974769	*DNAJC16*
chr5	1814073	1814816	5	0.010925406	0.022898137	0.00839021	*NDUFS6*
chr11	62160859	62161093	3	0.011006786	-0.122379125	-0.070518037	*ASRGL1*
chr1	8585852	8586297	4	0.011058359	0.020058144	0.013477052	*RERE*
chr1	235814009	235814814	11	0.011094562	0.031176951	0.010694585	*GNG4*
chr3	40279793	40280043	5	0.011097183	-0.038802134	0.003677484	*EIF1B-AS1;MYRIP*
chr11	126151328	126151408	3	0.011097266	-0.035593378	-0.02217521	*TIRAP*
chr10	129979292	129979375	3	0.011146205	0.017403023	0.009001187	*NA*
chr1	182573613	182574609	9	0.011301715	0.019714976	0.001199086	*RGS16*

chr19	24187194	24187558	4	0.011370022	-0.045335358	-0.011698919	*NA*
chr13	76054909	76055293	3	0.011383832	-0.012989111	0.001298015	*TBC1D4*
chr3	156878512	156879083	9	0.011412289	-0.055257526	-0.014437024	*CCNL1*
chr17	46806445	46806935	6	0.011448614	0.011048925	0.005764849	*HOXB13;MIR3185*
chr7	127291364	127291658	4	0.011469347	0.05319719	0.022033964	*SND1*
chr2	285979	286383	3	0.011502017	0.025456179	0.014620799	*ALKAL2*
chr10	98135085	98135209	4	0.011522124	0.007099631	0.005644886	*TLL2*
chr3	169782040	169782135	3	0.011522124	0.051629098	0.018315575	*GPR160*
chr14	104263779	104264228	3	0.011538176	0.033419829	0.014581753	*PPP1R13B*
chr22	50946582	50947305	5	0.01154242	-0.032415786	-0.010308814	*LMF2;NCAPH2*
chr19	46498049	46498695	10	0.01154242	0.020683676	0.008371279	*CCDC61*
chr22	42765436	42765727	4	0.011569531	0.018367555	0.008443816	*NA*
chr4	680656	681509	6	0.011656135	0.027843782	0.011985039	*SLC49A3*
chr11	128557265	128557589	3	0.011670913	0.01815361	0.003732451	*FLI1*
chr11	111411756	111412172	4	0.011697327	0.028872018	0.007760243	*LAYN*
chr11	47447843	47448534	12	0.011760429	-0.011298674	0.00264638	*PSMC3*
chr5	90675774	90676249	6	0.011760429	0.023027893	0.005443098	*ARRDC3;ARRDC3-AS1*
chr16	27241517	27242311	4	0.01177587	0.027756023	0.013792344	*NSMCE1*
chr7	588478	590098	9	0.011795051	-0.026959893	-0.001487873	*PRKAR1B*
chr12	54446019	54446308	7	0.011832155	-0.053570633	-0.025240372	*HOXC4*
chr17	72208322	72208788	4	0.011832155	-0.021411963	-0.006127151	*MGC16275;TTYH2*
chr6	154677972	154678593	4	0.011860688	0.049867273	0.025630856	*IPCEF1*
chr3	138668931	138669434	5	0.011864343	0.016756105	0.009199046	*FOXL2;FOXL2NB*
chr22	37584018	37584441	3	0.011895504	-0.043270624	-0.02715658	*C1QTNF6*
chr17	66288529	66288795	3	0.011917613	0.013391899	0.003095969	*ARSG;SLC16A6*
chr1	3620638	3621445	5	0.011921465	-0.046788216	-0.024721096	*TP73*
chr1	215740071	215740424	3	0.011967432	-0.030424033	-0.017506268	*KCTD3*
chr6	54130745	54131366	3	0.011980278	-0.03040524	-0.01410755	*MLIP*
chr2	33823673	33825072	16	0.011995173	-0.023411923	-0.000391422	*FAM98A*
chr16	89619402	89620236	4	0.011995173	0.01911306	0.002621401	*SPG7*
chr4	159131393	159132240	8	0.012033408	-0.022835502	0.008116148	*TMEM144*
chr11	326987	327178	3	0.01209012	0.015347998	0.008041941	*NA*
chr11	3817692	3818253	4	0.012126098	0.027415038	0.016453621	*NUP98;PGAP2*
chr11	47587410	47587672	4	0.012203672	0.007798073	0.001876923	*NDUFS3;PTPMT1*
chr15	25334879	25334988	3	0.01220938	-0.038535116	-0.027805439	*IPW;SNORD116-22;SNORD116-23;SNORD116-24*
chr1	18959268	18959891	5	0.012278513	-0.018121785	-0.007620429	*PAX7*
chr12	48577213	48578223	7	0.01233234	0.032491936	0.002973932	*CCDC184*
chr16	23724428	23724901	8	0.012336161	-0.050109733	-0.009497878	*ERN2*
chr4	185317908	185318230	3	0.012336161	0.023373302	0.008256745	*IRF2*
chr3	66311011	66311429	3	0.012347686	-0.031487539	-0.020314921	*SLC25A26*
chr20	13201193	13201844	15	0.01236167	0.013091614	0.005456837	*ISM1*
chr11	113844828	113846017	8	0.012417302	-0.032736449	-0.017161531	*HTR3A*
chr2	2829723	2829759	3	0.012449107	-0.02950197	-0.007682313	*NA*
chr1	38260810	38261401	7	0.012449107	0.042035882	0.019142792	*MANEAL*
chr6	31145302	31146471	7	0.012503031	-0.049941892	-0.017793537	*PSORS1C3*
chr6	33561099	33561449	7	0.012503357	-0.058793156	-0.022375067	*LINC00336*
chr8	145725402	145726136	5	0.012525591	-0.031035299	-0.023784194	*GPT;PPP1R16A*
chr5	14440757	14441074	4	0.012542877	0.036176787	0.027817987	*TRIO*
chr6	112374876	112375870	10	0.012544933	0.045147591	-0.000585219	*CCN6*

chr3	191046380	191047058	9	0.012560936	0.029552364	0.010884359	*CCDC50;UTS2B*
chr11	115630531	115631600	13	0.012573604	0.023653049	0.000841578	*LINC00900*
chr4	15657657	15657883	3	0.012573604	0.048259782	0.024503613	*FBXL5*
chr14	77767644	77767800	4	0.012620914	0.042197706	0.023640212	*POMT2*
chr1	223565773	223566794	11	0.012652392	0.057635429	0.024391796	*CCDC185*
chr14	86000942	86001111	3	0.012652765	0.025077769	0.017603144	*FLRT2*
chr2	134785392	134786158	5	0.012670692	-0.044654707	-0.020200535	*NA*
chr7	158550241	158550318	3	0.012670692	0.017941173	0.004639828	*ESYT2*
chr11	64057807	64058325	3	0.012686091	-0.025960728	-0.018149863	*KCNK4*
chr13	100641243	100641900	7	0.01272936	-0.030574291	-0.00047517	*NA*
chr12	72148251	72148853	10	0.012742923	-0.040707708	-0.009139982	*RAB21*
chr19	17516282	17517008	9	0.012761472	0.009991082	0.005228258	*BST2*
chr12	46660255	46660338	3	0.012806374	-0.031384217	-0.027936592	*SLC38A1*
chr12	110316883	110317274	3	0.012875769	-0.02867265	-0.02347919	*GLTP*
chr15	72598286	72599028	6	0.012875769	-0.02908465	-0.014217706	*CELF6*
chr12	50419238	50420033	13	0.012900231	-0.019179382	-0.002275536	*RACGAP1*
chr8	681212	681730	10	0.012942749	-0.018801236	-0.004345281	*ERICH1*
chr11	2323247	2324005	9	0.012942749	0.026040888	0.010302423	*C11orf21;TSPAN32*
chr3	155838317	155838625	3	0.012954024	-0.064337375	-0.047782146	*KCNAB1*
chr22	30901249	30901707	5	0.012954024	-0.027823352	-0.018913983	*SEC14L4*
chr3	108836912	108837661	8	0.013001293	-0.020434421	-0.007640182	*MORC1*
chr10	104613613	104614136	9	0.013063828	0.04250503	0.014846236	*BORCS7;BORCS7-ASMT*
chr11	1216305	1217528	6	0.013114445	-0.039759741	-0.00524022	*MUC5B*
chr4	1367596	1367848	3	0.013147592	0.01633355	0.003757582	*UVSSA*
chr15	25929201	25929574	3	0.013224475	-0.025208071	-0.014702136	*ATP10A*
chr10	63808748	63809170	13	0.013252159	-0.024860724	-0.016338315	*ARID5B*
chr4	87812668	87813594	6	0.013270587	0.009653996	0.003431289	*C4orf36*
chr20	34286194	34286564	3	0.013308328	0.011056633	0.000469189	*NFS1;ROMO1*
chr6	39758343	39758998	3	0.013343576	-0.021930709	-0.016649843	*DAAM2*
chr3	112929672	112930438	5	0.013379215	0.020099089	0.008359667	*BOC*
chr17	40170084	40170622	3	0.013379215	0.046231678	0.026342975	*DNAJC7;NKIRAS2*
chr2	242749271	242750355	13	0.013383398	0.092695566	0.007035317	*NEU4*
chr9	107688675	107690075	8	0.01338728	-0.054829485	-0.003709594	*ABCA1*
chr2	130940427	130940706	3	0.013409765	-0.035141168	-0.021148141	*MZT2B;SMPD4*
chr6	89791122	89792677	6	0.013443993	0.048495271	0.015299443	*PNRC1*
chr5	176304609	176305077	3	0.01345495	-0.049153241	-0.006007873	*UNC5A*
chr2	106681998	106682888	6	0.013479257	-0.043076489	-0.0028979	*ECRG4*
chr12	16499963	16500795	5	0.013485294	0.042546982	0.022030956	*MGST1*
chr12	72058171	72058809	6	0.013505458	0.009440806	0.00438656	*THAP2;ZFC3H1*
chr9	128819840	128819969	3	0.013524061	0.037395262	0.01316392	*NA*
chr16	712818	713101	5	0.013570177	-0.024315205	0.002245963	*RHOT2;WDR90*
chr3	122746985	122748086	5	0.013615311	-0.034864926	-0.004386045	*SEMA5B*
chr7	99775043	99775862	13	0.01362466	0.040304899	0.012961666	*GPC2;STAG3*
chr5	4230502	4230872	6	0.013691436	-0.029408612	-0.017751308	*NA*
chr10	116636356	116637130	5	0.013715881	0.06924653	0.03437035	*FAM160B1*
chr16	28996021	28997041	12	0.013763029	-0.012034847	0.00189143	*LAT;SPNS1*
chr21	44898090	44898596	5	0.013768591	-0.034273806	-0.020872099	*LINC00313*
chr13	41593385	41593582	4	0.013788699	-0.02232408	0.001023429	*ELF1*
chr2	177015812	177016490	8	0.013866233	0.045421686	0.015770118	*HOXD4*

chr7	94294798	94295390	4	0.013886287	-0.021999013	-0.004991646	*PEG10*
chr7	27207996	27208347	6	0.013918117	0.022905712	0.008972068	*HOXA10-AS;HOXA10-HOXA9;HOXA9*
chr7	126988120	126988783	6	0.013920621	0.037512145	-0.000331431	*NA*
chr9	77230369	77230509	3	0.013925184	0.035281476	0.011109394	*RORB*
chr4	6299306	6300073	4	0.013946927	0.027960601	0.00582873	*WFS1*
chr11	66335975	66336293	7	0.013969384	-0.020323435	-0.003849984	*CTSF*
chr1	42921356	42921931	9	0.013997213	-0.021615367	-0.005859202	*PPCS;ZMYND12*
chr12	52346851	52347184	5	0.014054947	0.042151822	0.017976943	*ACVR1B*
chr13	100740707	100741197	10	0.01408501	0.019184639	0.000481188	*PCCA*
chr6	33400021	33401542	11	0.014090964	0.038510492	0.018661097	*MIR5004;SYNGAP1*
chr7	91763433	91764605	18	0.014096873	-0.017435482	-0.003201755	*CYP51A1*
chr19	863054	863244	4	0.014107091	-0.053768882	-0.042317621	*CFD*
chr16	34979252	34979671	3	0.014130377	0.060390596	0.035918397	*LINC02167;RNA5SP411*
chr19	30432806	30433293	7	0.014151363	0.01107599	0.004397474	*URI1*
chr11	122848098	122848518	4	0.014203236	0.018704739	0.002769937	*BSX*
chr17	43339476	43340243	8	0.014253378	0.021553471	-0.002929467	*MAP3K14-AS1;SPATA32*
chr4	76911810	76912761	12	0.014392145	0.023296359	0.000757902	*SDAD1*
chr12	69201477	69202047	8	0.01451669	-0.015995538	-0.002621793	*LOC100130075;MDM2*
chr13	50707937	50708420	3	0.01451669	-0.018824907	-0.002367893	*DLEU1*
chr3	137717665	137718043	3	0.014542084	-0.033720614	-0.003369611	*CLDN18*
chr6	32164927	32165321	4	0.014542705	-0.030059467	-0.021018207	*GPSM3;NOTCH4*
chr12	131616937	131617481	5	0.014567791	-0.034478469	-0.021784856	*ADGRD1*
chr16	50321678	50322156	5	0.014575248	0.029749175	0.019790011	*ADCY7*
chr16	1257807	1257884	3	0.014650769	-0.035957253	-0.001727394	*CACNA1H*
chr4	109087419	109088046	7	0.014724516	-0.030402065	-0.009682269	*LEF1;LEF1-AS1*
chr3	10182524	10183112	6	0.014754266	-0.035477898	-0.011197035	*VHL*
chr6	131571435	131571669	4	0.014865391	0.033006698	0.015403202	*AKAP7*
chr16	89381904	89382370	5	0.014922321	0.026915888	0.014138152	*ANKRD11*
chr2	170681271	170682287	9	0.014953815	-0.013200369	0.003150609	*METTL5;UBR3*
chr16	2314361	2314929	4	0.014963368	-0.022967259	-0.009057282	*RNPS1*
chr7	98099806	98100419	6	0.014963368	-0.044403246	0.000949976	*NA*
chr14	38724255	38724945	5	0.015049598	0.038130254	0.01235018	*CLEC14A*
chr5	140551968	140552600	8	0.015057402	0.065302666	0.016751129	*PCDHB7;PCDHB8*
chr11	2150902	2151349	4	0.015105789	0.035626754	-0.000332991	*IGF2;INS-IGF2*
chr3	42306737	42307193	4	0.015115706	0.020490911	0.015882477	*CCK*
chr8	143330497	143331321	3	0.015152819	-0.024820883	-0.023117002	*TSNARE1*
chr9	87282548	87283470	8	0.015160876	0.02025282	0.005970286	*NTRK2*
chr8	80679670	80680899	7	0.01516332	0.021468211	0.00357273	*HEY1*
chr12	122461295	122461621	3	0.015229998	0.019442688	0.011544787	*BCL7A*
chr20	34204831	34205488	8	0.015263245	-0.034549462	-0.00306093	*SPAG4*
chr10	73849124	73849626	4	0.015272798	-0.03613183	-0.010142265	*ANAPC16;SPOCK2*
chr2	241562424	241562757	5	0.015284112	-0.054140085	-0.034141902	*GPR35*
chr19	1568534	1569132	5	0.015320628	0.068770167	0.019744886	*MEX3D*
chr12	77155453	77156412	4	0.015414444	-0.016688732	-0.005219129	*ZDHHC17*
chr18	44777736	44778021	3	0.015497839	0.044992377	0.022025861	*SKOR2*
chr11	61891341	61891725	5	0.015523596	0.007407603	-0.000978097	*INCENP*
chr20	44650062	44650449	9	0.015547779	0.033356821	0.011881369	*SLC12A5*
chr12	6898500	6898975	5	0.015553208	-0.014565806	0.000593989	*CD4*
chr12	69604618	69605100	3	0.015575945	0.06021789	0.022339553	*NA*

chr11	15962841	15963167	5	0.01565407	0.063679907	0.024997066	*NA*
chr5	3597311	3597760	4	0.015678111	0.030511289	0.007231099	*IRX1*
chr12	16108919	16109561	4	0.015766804	-0.023333025	-0.004795467	*DERA*
chr1	182361557	182362757	5	0.015781113	0.058443805	0.002799107	*GLUL*
chr14	23775663	23776482	7	0.015842387	0.017410025	0.009989892	*BCL2L2;BCL2L2-PABPN1;PPP1R3E*
chr11	124709577	124709925	4	0.01584788	0.009078193	0.004299894	*NA*
chr1	113161296	113161717	5	0.015927763	0.017028412	0.004457933	*CAPZA1;ST7L*
chr6	18122474	18123305	13	0.015936075	-0.047361592	-0.00982758	*NHLRC1*
chr19	33210464	33210851	9	0.015970017	-0.034816273	-0.001846558	*TDRD12*
chr8	1860385	1861136	5	0.016074916	-0.068535659	-0.031911664	*ARHGEF10*
chr9	78504994	78506294	9	0.016077241	0.011028104	0.004869508	*PCSK5*
chr5	37837463	37837788	3	0.016309819	-0.015773317	-0.014015803	*GDNF;GDNF-AS1*
chr18	24443729	24444196	4	0.016333599	-0.036108964	-0.006265339	*AQP4;AQP4-AS1*
chr12	95867190	95867833	12	0.016347296	-0.018005434	-0.000815514	*METAP2*
chr9	124133073	124133556	4	0.016383762	-0.050126728	-0.031421481	*STOM*
chr19	12941257	12941875	11	0.016409971	0.024898462	0.003342547	*RTBDN*
chr19	1752160	1752327	4	0.016466639	0.00905398	0.001775513	*ONECUT3*
chr13	33859636	33860133	10	0.016492228	0.024175787	0.009345467	*STARD13*
chr1	247511364	247511469	3	0.016495597	-0.034865011	-0.034125733	*NA*
chr7	73507241	73507610	7	0.016506676	0.016588752	0.009464845	*LIMK1*
chr11	31822243	31822341	3	0.016541219	0.045678459	0.019112698	*PAX6*
chr18	7116421	7116977	4	0.016655152	0.020846948	0.006475401	*LAMA1*
chr20	58514201	58514806	4	0.016700038	-0.024378445	0.004582785	*FAM217B;PPP1R3D*
chr5	1201033	1201978	12	0.016734506	-0.041258616	-0.01797588	*SLC6A19*
chr14	78708634	78709039	4	0.016744444	-0.0277212	-0.015567325	*NRXN3*
chr1	161337679	161337828	3	0.016778338	-0.041296214	-0.030380846	*CFAP126*
chr17	19551345	19551893	7	0.016801947	0.01147914	0.001472109	*ALDH3A2*
chr13	95358476	95358924	4	0.016801947	0.020895137	0.013359023	*NA*
chr2	38301143	38301756	3	0.016829253	0.019570317	0.010826673	*CYP1B1*
chr1	28241317	28241765	10	0.016892099	0.030585435	0.00469611	*RPA2*
chr2	150443536	150444173	7	0.016892705	0.016123947	0.006121105	*MMADHC*
chr17	4642047	4642647	4	0.016907797	-0.011097559	-0.004386192	*CXCL16;ZMYND15*
chr16	18813117	18813519	6	0.016931069	-0.016086451	-0.000153658	*ARL6IP1*
chr12	6960214	6960807	8	0.016939233	0.015213101	0.004816559	*CDCA3;USP5*
chr12	79257496	79258313	8	0.016957039	0.022804353	0.004288395	*SYT1*
chr6	32765117	32765402	6	0.016957617	0.018038318	0.008210196	*NA*
chr6	169351108	169351479	3	0.01703575	-0.024983235	-0.013252172	*NA*
chr2	24397539	24398170	9	0.017050777	0.060172517	0.022787265	*FAM228A;FAM228B*
chr5	13944491	13945371	5	0.017090779	-0.050810006	-0.016839704	*DNAH5*
chr16	25702044	25702614	4	0.017118897	-0.047330155	-0.018655762	*HS3ST4*
chr1	155239301	155239493	6	0.017118897	-0.030207191	-0.002849294	*CLK2*
chr1	151512592	151513502	11	0.017217051	0.026441653	0.003808439	*MIR554;TUFT1*
chr1	6303728	6304499	8	0.017287514	0.028683416	0.015026332	*HES3*
chr9	131579977	131580113	3	0.017332261	-0.018348417	-0.009455061	*ENDOG*
chr13	28498384	28499045	6	0.017365737	0.024877662	0.011697577	*PDX1*
chr4	80329284	80329633	10	0.01744074	-0.031322663	-0.000942135	*GK2*
chr6	136611553	136611664	3	0.017496784	0.011142114	0.004380516	*BCLAF1*
chr1	111991797	111992392	10	0.017496784	0.02157396	0.005563106	*ATP5PB;WDR77*
chr17	73266070	73266642	4	0.017522193	0.019281759	0.007790055	*LOC100287042;MIF4GD*

chr11	968423	969000	6	0.017607033	0.034947087	0.017324155	*AP2A2*
chr22	44463707	44464536	3	0.017609212	-0.018544232	0.002534878	*PARVB*
chr1	181451906	181452048	4	0.017638287	0.019903678	0.00928045	*CACNA1E*
chr6	170452864	170453283	3	0.017742453	-0.105735517	-0.059487427	*NA*
chr12	52915214	52915646	3	0.017766562	-0.046565639	-0.01911449	*KRT5*
chr6	28540442	28541039	5	0.017771632	-0.039892522	-0.008953316	*ZBED9*
chr7	150721518	150721864	3	0.017799663	-0.031238529	-0.015974283	*ABCB8;ATG9B*
chr6	100841663	100842099	3	0.017899038	0.01632085	0.005368959	*SIM1*
chr11	47436290	47436675	3	0.017907771	-0.022467213	-0.016616789	*SLC39A13*
chr17	73873529	73873924	4	0.017928263	0.017576032	0.009190842	*TRIM47*
chr3	190580898	190581232	3	0.017958577	0.024340517	0.003645673	*GMNC*
chr15	30918595	30919020	3	0.017977693	0.02739236	0.01109468	*ARHGAP11B*
chr11	64563983	64564322	4	0.018032606	0.021009124	0.011083116	*MAP4K2*
chr17	51900238	51900887	3	0.018086843	-0.020110336	-0.005221338	*KIF2B*
chr16	5117386	5117565	3	0.01812555	-0.023035118	-0.018926807	*ALG1;C16orf89*
chr10	88730324	88730946	5	0.018163925	0.043837546	0.020852333	*ADIRF;AGAP11*
chr7	922165	923365	9	0.018168318	0.036213457	0.007557154	*GET4*
chr16	86610230	86611049	3	0.018173421	-0.027010088	-0.008168326	*FOXL1*
chr2	11809997	11810683	10	0.018210583	-0.051796222	0.008867958	*NTSR2*
chr13	52769289	52769951	5	0.018212197	-0.064852447	-0.025203641	*MRPS31P5*
chr7	12726089	12726431	7	0.01823128	-0.020838051	-0.005789851	*ARL4A*
chr15	91498953	91499023	3	0.018260091	-0.044851463	-0.038727031	*RCCD1*
chr7	64407275	64407743	3	0.018268252	-0.046982651	-0.026630535	*NA*
chr20	61590751	61591209	4	0.018268252	0.111568374	0.060161573	*SLC17A9*
chr7	155369184	155369424	3	0.018480252	-0.042403162	-0.006481922	*NA*
chr1	227071861	227072161	3	0.018562573	0.031789039	0.018656445	*PSEN2*
chr5	146258354	146259003	12	0.01869486	0.018799074	0.005186449	*PPP2R2B*
chr5	2642885	2643299	5	0.018708626	-0.047818203	-0.024041555	*NA*
chr11	65194933	65195039	4	0.018708626	0.03129381	0.023479017	*NEAT1*
chr8	144635316	144635610	6	0.018708626	0.040001192	0.024293841	*GSDMD*
chr6	170730413	170730693	3	0.018761766	-0.036066788	-0.023814564	*NA*
chr2	171570700	171571151	5	0.018767964	0.008332572	-0.000198671	*LINC01124;SP5*
chr3	194836302	194836847	3	0.01878098	0.073082439	0.029822016	*XXYLT1*
chr10	98946778	98946943	3	0.018807837	-0.032738282	-0.021027563	*ARHGAP19-SLIT1;SLIT1*
chr6	167189543	167190226	4	0.018815135	0.040445847	0.023201789	*RPS6KA2*
chr7	135346500	135347371	12	0.018834693	-0.062878604	-0.009145929	*STMP1*
chr11	74022326	74023050	11	0.018834693	0.024066469	-0.000812873	*P4HA3*
chr2	1711759	1711966	3	0.018834693	0.031846737	0.027618157	*PXDN*
chr3	192289245	192289293	3	0.01889601	-0.072383518	-0.06539587	*FGF12*
chr14	53418316	53419010	5	0.01889601	0.025359172	0.00438134	*FERMT2*
chr2	176950273	176950736	3	0.018904926	-0.031958044	-0.014567913	*EVX2*
chr11	59323070	59323620	5	0.018916918	-0.039455567	-0.02404128	*NA*
chr9	27297089	27297406	3	0.018989306	-0.059844413	-0.027017093	*EQTN*
chr15	101418967	101419518	13	0.019026747	-0.039925477	0.00322313	*ALDH1A3*
chr2	66803345	66804145	5	0.019045482	-0.00900993	-0.002642612	*NA*
chr8	22091132	22091294	3	0.01904579	-0.020555649	-0.014957053	*PHYHIP*
chr6	138725069	138725799	8	0.019105854	0.014098709	0.002015568	*HEBP2*
chr1	20208405	20208636	4	0.019125843	0.018009937	0.006046708	*OTUD3*
chr8	23100643	23101221	4	0.019125843	0.034385945	0.020399271	*CHMP7*

chr8	1649853	1650172	4	0.019152837	-0.04872224	-0.032825357	*DLGAP2*
chr2	129659018	129659946	6	0.019225106	-0.043447769	-0.035159392	*NA*
chr6	26987575	26987810	3	0.019225752	-0.038654526	-0.021408158	*LINC00240;LOC100270746*
chr4	153699460	153700211	3	0.019225752	-0.028492673	-0.001081499	*ARFIP1;TIGD4*
chr15	85923174	85923765	7	0.01925728	0.009563377	0.001389125	*AKAP13*
chr5	8786239	8786867	3	0.019305562	0.018419011	0.005957576	*NA*
chr2	131673771	131674494	11	0.019310284	-0.052622248	-0.01839003	*ARHGEF4*
chr10	85974018	85974172	4	0.019348165	-0.049788244	-0.043910902	*CDHR1*
chr22	21984177	21984483	6	0.019395288	0.00676693	-0.000897164	*CCDC116;YDJC*
chr12	110778514	110778935	4	0.019508543	-0.021368742	-0.005292355	*ATP2A2*
chr14	75981868	75981936	3	0.019536274	-0.054773922	-0.047180009	*NA*
chr18	47013515	47014260	9	0.019536274	-0.041628538	-0.011024448	*C18orf32;MIR1539;RPL17-C18orf32*
chr18	28681689	28682497	8	0.019536274	0.019510887	0.004422898	*DSC2*
chr6	168841257	168841574	4	0.019571666	0.03900033	0.02285801	*SMOC2*
chr6	168613889	168614227	3	0.019607965	-0.041805644	-0.020682735	*NA*
chr12	108078821	108079012	3	0.019631076	-0.078375328	-0.051430055	*PWP1*
chr11	32008659	32009163	5	0.019631076	0.03914099	0.01036314	*RCN1*
chr3	61550379	61550635	3	0.019638134	0.033364006	0.022534342	*PTPRG*
chr17	32483640	32484259	12	0.019663103	0.023480259	0.007448043	*ASIC2*
chr17	48242355	48242743	5	0.019711515	-0.017464493	-0.013820871	*SGCA*
chr2	196933266	196934154	10	0.019770336	0.065327646	0.011790485	*DNAH7*
chr8	22550720	22551274	9	0.019770729	0.013710523	0.003930715	*EGR3*
chr13	113739251	113739577	4	0.019772045	-0.022703172	-0.007159556	*MCF2L*
chr1	159556946	159557632	4	0.019834125	-0.035221474	-0.019101247	*APCS*
chr14	94546481	94546857	3	0.01987795	-0.016720813	-0.000606241	*DDX24;IFI27L1*
chr2	171782647	171783942	3	0.019903524	0.031055663	-0.000447773	*GORASP2*
chr7	157293538	157293606	3	0.019904981	-0.040798581	-0.022217928	*NA*
chr1	228195522	228195794	3	0.019933544	-0.022942088	-0.009429453	*WNT3A*
chr5	43191672	43192025	6	0.019944773	0.049592105	0.014624864	*NIM1K;ZNF131*
chr5	171594998	171595487	4	0.019969259	0.028195604	0.016667813	*STK10*
chr1	156047061	156047509	5	0.019976405	0.025089682	0.010881224	*LMNA;MEX3A*
chr5	1183037	1183424	4	0.01999664	-0.033177734	-0.011879911	*NA*
chr3	195488725	195489306	3	0.020128512	0.057311711	0.039006333	*MUC4*
chr8	82643667	82644012	3	0.020191286	-0.038026676	-0.02548643	*CHMP4C*
chr14	93388800	93389249	7	0.020191286	-0.031989453	-0.011988399	*CHGA*
chr1	9258678	9259066	5	0.020220158	-0.02812446	-0.013593748	*NA*
chr2	236619445	236619825	5	0.020239149	0.03713291	0.007881352	*AGAP1*
chr6	99396000	99396345	8	0.020276865	0.048138254	0.011762623	*FBXL4*
chr11	3121024	3121590	5	0.020287685	0.031158132	0.00924909	*OSBPL5*
chr17	73780035	73780729	11	0.020386629	0.026943982	-0.000245312	*H3-3B;MIR4738;UNK*
chr1	38471336	38472003	7	0.020406812	0.020441063	0.010352916	*FHL3*
chr12	54133422	54133730	5	0.020441454	0.01958574	0.004026022	*NA*
chr13	79234144	79234435	4	0.020482526	0.080294363	0.055252153	*OBI1*
chr2	105858730	105859083	4	0.020530939	-0.029828845	-0.006836562	*GPR45*
chr11	2161846	2162363	6	0.020618043	-0.021528498	-0.005000539	*IGF2;IGF2-AS;INS-IGF2*
chr17	78962984	78963290	4	0.020641081	-0.070397791	-0.031300911	*CHMP6*
chr3	197475824	197476591	14	0.020720756	0.012298156	0.003086592	*FYTTD1;RUBCN*
chr17	20799408	20799532	5	0.020725226	0.074583058	0.047950841	*CCDC144NL*
chr12	113787113	113787384	3	0.020732983	-0.047829967	-0.042656959	*NA*

chr16	28986645	28986829	4	0.020770554	0.027567142	0.012953397	*SPNS1*
chr14	61113963	61114476	3	0.020775267	-0.031673198	-0.012940558	*SIX1*
chr17	202588	203392	8	0.020790578	0.036582546	0.0076714	*RPH3AL*
chr9	130540941	130541531	6	0.020888727	0.017614949	0.007732008	*SH2D3C*
chr7	70597351	70598282	10	0.021047784	-0.016584387	0.002310025	*GALNT17*
chr11	126453681	126453743	3	0.021177659	0.030652279	-0.000769003	*KIRREL3*
chr20	57463572	57464002	18	0.021185132	-0.03310932	-0.010875921	*GNAS*
chr5	3285935	3286296	6	0.021208298	-0.046933947	-0.010268744	*NA*
chr13	33112842	33113343	10	0.021228336	0.025277579	0.008658923	*N4BP2L2*
chr14	104019386	104019746	4	0.021228336	0.021578602	0.009239658	*NA*
chr8	143086298	143086459	3	0.021229227	0.023994407	0.00837839	*NA*
chr12	118541055	118541585	4	0.021255866	0.007013155	0.002502063	*VSIG10*
chr3	123603316	123603624	6	0.021264899	0.028900048	0.010032683	*MYLK*
chr15	96868430	96869220	10	0.021278748	-0.023197358	0.005403233	*NR2F2;NR2F2-AS1*
chr8	145580712	145581006	6	0.021323875	-0.030606044	-0.010396294	*FBXL6;SLC52A2;TMEM249*
chr17	19651983	19652535	5	0.021344859	-0.030092283	-0.011634153	*ALDH3A1*
chr11	93430585	93430980	3	0.021402912	0.042151902	0.017476278	*CEP295*
chr5	31193844	31194749	9	0.021411174	-0.013236796	0.002777589	*CDH6*
chr10	11784058	11784473	7	0.021548434	0.016445743	0.005279311	*ECHDC3*
chr12	132303409	132303735	4	0.021548771	-0.034102327	-0.006245412	*NA*
chr21	43528205	43528868	4	0.021560694	0.078280575	0.020528533	*UMODL1;UMODL1-AS1*
chr22	18592265	18592555	3	0.021584878	-0.026493255	-0.010679312	*TUBA8*
chr22	39745530	39746054	7	0.021740192	0.020277219	0.00835966	*SYNGR1;TAB1*
chr1	155053851	155054210	3	0.021752524	-0.016070586	-0.005386751	*EFNA3*
chr18	5197327	5197632	8	0.021806644	0.025729495	0.012127394	*AKAIN1*
chr16	85393998	85394259	3	0.021815162	-0.024740848	-0.01520611	*NA*
chr2	121791173	121792073	3	0.021823735	0.024051688	0.004724195	*NA*
chr1	205391381	205391881	4	0.021857008	-0.031872158	-0.016281468	*LEMD1*
chr14	24777708	24778279	6	0.021863135	-0.028554685	-0.014226357	*CIDEB;LTB4R;LTB4R2*
chr12	50306291	50306547	3	0.021863135	0.025068531	0.002039659	*LINC02396*
chr6	111888446	111888870	6	0.02187874	0.019131408	0.011662572	*TRAF3IP2;TRAF3IP2-AS1*
chr22	37266498	37266803	4	0.022001593	-0.025245128	-0.014053075	*NCF4*
chr1	160642715	160642975	3	0.022028049	-0.049576111	-0.03356809	*NA*
chr12	52399713	52400663	8	0.022124239	-0.037529971	-0.002333639	*TAMALIN*
chr10	135090997	135091571	4	0.022125074	0.041008677	0.016472813	*ADAM8*
chr17	7328520	7328921	7	0.022129147	0.034355615	0.003254353	*SPEM2*
chr12	13363855	13364374	5	0.02214266	0.064381225	-0.004179311	*EMP1*
chr21	34305156	34305354	3	0.022249822	-0.035209985	-0.017552127	*NA*
chr2	24712657	24713283	4	0.022249822	0.054204326	0.022710488	*NA*
chr12	56881865	56882535	9	0.022443542	-0.014225834	0.003398976	*GLS2*
chr19	49296684	49296857	5	0.022598541	0.045336673	-0.000311653	*NA*
chr20	1471884	1472419	6	0.022679169	0.021884	0.011208665	*SIRPB2*
chr15	25123287	25123688	4	0.022691805	0.057994045	0.036139917	*SNRPN*
chr4	158142677	158142891	5	0.022707336	0.01940577	0.008425743	*GRIA2*
chr20	33033099	33033157	3	0.02272061	0.025116997	0.02322378	*ITCH*
chr15	72667883	72668275	7	0.022726354	0.010772704	0.001525627	*CELF6;HEXA;HEXA-AS1*
chr14	23835870	23836047	3	0.022731236	-0.043409534	-0.011338528	*EFS*
chr8	496128	496739	6	0.02275252	-0.034214706	-0.002950077	*TDRP*
chr11	116661043	116661678	5	0.022762862	-0.038199235	-0.020768039	*APOA5;ZPR1*

chr15	29213736	29213860	3	0.022762862	0.028183811	0.021407833	*APBA2*
chr11	404596	404762	3	0.022813436	-0.019447911	-0.01456826	*PKP3*
chr16	52108906	52109206	3	0.022817156	-0.023655107	0.003895725	*C16orf97*
chr8	101965385	101965903	10	0.022863394	-0.009678475	0.000325887	*YWHAZ*
chr17	16118138	16118806	4	0.022875297	0.011830731	0.004757439	*NCOR1;PIGL*
chr11	125133120	125133668	5	0.022899434	-0.022657776	0.000365521	*PKNOX2*
chr4	105411270	105411756	3	0.022905494	-0.048522119	-0.034182578	*CXXC4*
chr6	52110012	52110447	3	0.022918434	0.020478818	0.011484165	*IL17F*
chr17	74911940	74912348	3	0.022980137	-0.04640207	-0.015488787	*MGAT5B*
chr14	65409452	65410479	8	0.022995695	0.019007929	0.004094588	*CHURC1-FNTB;GPX2*
chr9	19464676	19465241	3	0.023079217	0.042619423	0.023995942	*NA*
chr19	46170845	46171507	8	0.023115806	0.014834267	0.006228901	*GIPR*
chr9	97402555	97403129	3	0.02314294	-0.024695078	-0.021684236	*FBP1*
chr2	44587949	44588074	4	0.023225857	0.0217331	0.014296306	*CAMKMT;PREPL*
chr12	12185594	12186137	4	0.023243395	-0.01208105	0.000354224	*ETV6*
chr6	32178017	32178603	5	0.023266676	-0.018276763	-0.011363245	*NOTCH4*
chr14	89016862	89017615	5	0.023348745	0.033515943	-0.003354923	*PTPN21*
chr19	52598653	52599329	9	0.023373546	0.029027302	0.00970716	*ZNF841*
chr7	106508373	106508732	3	0.023400687	-0.017445262	-0.015185591	*PIK3CG*
chr1	39492366	39492570	4	0.023406072	0.007008673	0.003377416	*NDUFS5*
chr5	125935836	125936553	11	0.023532654	0.025986287	0.006059819	*ALDH7A1;PHAX*
chr6	167317118	167317799	4	0.023564465	-0.043101109	-0.020642761	*NA*
chr16	1464758	1465405	6	0.023564465	0.017749618	0.006211646	*UNKL*
chr1	1261040	1261485	4	0.023649141	-0.026023616	-0.01656113	*CPTP;INTS11*
chr2	176947764	176947940	3	0.023674895	0.020364621	0.006114645	*EVX2*
chr2	45168507	45168924	6	0.023683999	-0.007801087	-0.001222593	*SIX3*
chr20	17822840	17823154	4	0.023693464	-0.046464128	-0.002018904	*NA*
chr12	54943700	54943915	6	0.023711605	0.028062217	0.011552395	*PDE1B*
chr1	83450735	83450929	3	0.023713827	0.021810324	0.004219194	*NA*
chr10	16478471	16479192	11	0.023754407	-0.037196429	-0.001791577	*PTER*
chr19	4636208	4636457	3	0.023836196	-0.015030853	-0.002141031	*TNFAIP8L1*
chr5	141016102	141016960	9	0.023851437	-0.01236553	-0.003091708	*HDAC3;RELL2*
chr8	144451413	144451924	5	0.023878133	0.029806916	0.01472412	*RHPN1;RHPN1-AS1*
chr1	23888963	23889092	3	0.023896949	0.025652412	0.016534971	*ID3*
chr6	170249394	170249553	3	0.023998131	-0.022627538	-0.016335257	*NA*
chr14	104394430	104394831	6	0.024019313	0.089471006	0.036537013	*TDRD9*
chr6	33039396	33039500	5	0.024058589	-0.037796361	-0.021043238	*HLA-DPA1;HLA-DPB1*
chr1	203317248	203317913	7	0.024092207	0.030135337	0.00032323	*FMOD*
chr15	92612836	92613280	3	0.024098082	-0.033647209	-0.007819202	*SLCO3A1*
chr19	28237512	28237993	3	0.024120822	0.057346622	0.04544144	*NA*
chr11	125496092	125496752	7	0.024159204	0.021980646	0.006678548	*CHEK1*
chr22	36011843	36012221	4	0.024285242	0.026117115	0.015427575	*MB*
chr1	110752047	110752307	4	0.024286671	-0.078972663	-0.037325465	*KCNC4*
chr19	38924045	38924436	10	0.024286671	0.011466878	0.005161612	*RYR1*
chr5	34657262	34657458	3	0.024345737	-0.016202052	-0.008558062	*RAI14*
chr1	47010056	47010223	3	0.024345737	0.029854663	0.009030805	*MKNK1-AS1*
chr3	52719141	52719354	4	0.024436017	0.020467557	0.006408857	*GNL3;PBRM1;SNORD19*
chr11	103766022	103766274	3	0.024466681	-0.030581463	-0.015776525	*NA*
chr17	37921981	37922420	3	0.02451487	-0.02100726	-0.001453977	*IKZF3*

chr8	38853964	38854671	11	0.02451487	0.009027686	0.00159969	*ADAM9;TM2D2*
chr1	161184305	161185092	7	0.02451487	0.031936457	0.014176873	*FCER1G*
chr12	114842700	114845223	18	0.024591482	0.048781914	0.003902481	*TBX5;TBX5-AS1*
chr6	24646676	24647210	5	0.024650081	0.099228194	0.043088854	*KIAA0319*
chr17	35084975	35085134	3	0.024655108	0.034097	0.021352108	*NA*
chr13	112721712	112721950	7	0.024662474	0.015758581	0.009592974	*SOX1*
chr14	95429455	95429750	3	0.024677834	-0.028502528	-0.015300771	*NA*
chr5	37952598	37953410	4	0.024746792	-0.042873331	-0.027041366	*NA*
chr2	182547401	182548308	7	0.024752211	0.017032391	0.007770214	*CERKL;NEUROD1*
chr5	32099080	32099275	3	0.024766582	0.01942187	0.012061164	*PDZD2*
chr5	119798890	119799531	3	0.024880928	-0.056879743	-0.017131867	*PRR16*
chr1	165512980	165513361	6	0.024884072	0.022397583	0.012432044	*LRRC52;LRRC52-AS1*
chr5	140515039	140515675	5	0.024916363	0.061011817	0.006644391	*PCDHB5*
chr7	131377001	131377089	3	0.024927178	0.016180235	0.008902132	*NA*
chr6	71276074	71276807	11	0.024927301	0.022741616	0.006311998	*SDHAF4*
chr8	143824243	143824586	4	0.025017228	-0.030013331	-0.008547401	*SLURP1*
chr9	100850334	100850391	3	0.025017228	0.045672215	0.038316006	*TRIM14*
chr5	6411286	6411510	3	0.025032372	-0.032656339	-0.016572113	*NA*
chr11	32450244	32450692	4	0.025120186	0.024028611	0.017250414	*WT1*
chr22	46449981	46450707	7	0.025160502	-0.040170169	-0.016098671	*MIRLET7BHG;PRR34;PRR34-AS1*
chr6	30313380	30313742	8	0.025184088	0.028525862	0.005430512	*RPP21;TRIM39-RPP21*
chr15	25522119	25522378	3	0.025226568	0.057388763	0.014609246	*SNORD109B*
chr11	12695497	12695578	3	0.02527706	0.013056382	0.004072279	*TEAD1*
chr5	124043109	124043436	3	0.025315783	0.033143796	0.006538068	*ZNF608*
chr15	40846077	40846513	4	0.025335158	0.038675377	0.011567243	*CCDC32*
chr8	142437128	142437236	4	0.025410364	-0.014984974	-0.010747116	*PTP4A3*
chr2	27072271	27072641	5	0.025416615	0.01601962	0.008407228	*DPYSL5*
chr3	142666108	142666759	5	0.025431932	-0.05223753	-0.040759409	*NA*
chr9	4299861	4300432	7	0.025431932	0.019734734	0.003296244	*GLIS3*
chr4	6998522	6998625	4	0.025551261	-0.01680152	-0.007015803	*TBC1D14*
chr9	21974871	21975497	10	0.02556429	0.010285324	0.004653956	*CDKN2A;MTAP*
chr1	61547131	61547758	8	0.025575738	-0.024327743	-0.008121035	*NFIA*
chr6	5132887	5133630	7	0.025593667	0.034169191	0.015205004	*LYRM4*
chr10	75119387	75119844	4	0.02559934	-0.020951259	-0.01481073	*CFAP70*
chr18	55108852	55109254	5	0.025624932	0.020217626	0.006203969	*ONECUT2*
chr6	28641651	28642279	10	0.025648535	0.014627668	0.00620672	*NA*
chr15	91642909	91643381	11	0.02572656	-0.027098222	-0.002171689	*SV2B*
chr1	217310572	217311014	3	0.025785077	0.01661747	0.009532718	*ESRRG*
chr2	3698421	3698862	4	0.025785077	0.019339039	0.011915336	*NA*
chr1	26735606	26736154	8	0.025820322	0.016014874	0.004300058	*LIN28A*
chr11	74424489	74424521	3	0.025836647	-0.022538949	-0.013075695	*CHRDL2*
chr1	76251537	76252291	10	0.025876342	0.014538526	0.002960669	*RABGGTB;SNORD45A;SNORD45B;SNORD45C*
chr20	17206529	17206999	8	0.025895299	0.021829044	0.003923424	*PCSK2*
chr22	50984767	50985117	3	0.025979439	-0.050474164	-0.032078622	*KLHDC7B*
chr22	50353996	50354243	6	0.026043279	-0.009681924	-0.002981231	*PIM3*
chr15	45003321	45003957	12	0.026091059	0.017633964	0.003561171	*B2M;PATL2*
chr4	85416865	85417312	4	0.026119239	0.023095438	0.011582708	*NKX6-1*
chr22	19136844	19137222	3	0.02613693	0.040896533	0.021104974	*ESS2;GSC2*
chr1	2574031	2574349	3	0.026268762	0.006080382	0.004791678	*TTC34*

chr11	2011341	2012180	5	0.026291223	-0.022978672	-0.004223189	*MRPL23-AS1*
chr15	28341714	28342256	6	0.026291223	0.009525506	0.004057453	*OCA2*
chr2	180130081	180130591	3	0.026341532	-0.034036101	-0.01849387	*SESTD1*
chr9	112213987	112214232	3	0.026341532	0.054679894	0.024633835	*PTPN3*
chr15	85525384	85525744	4	0.026368502	0.017262609	0.00738586	*PDE8A*
chr9	35096622	35096980	7	0.026392458	0.041568991	0.002595048	*PIGO*
chr3	55515017	55515232	4	0.026411637	-0.036233227	-0.014323345	*WNT5A*
chr15	29967531	29968195	6	0.026527108	-0.054312737	-0.030798082	*NA*
chr1	3059626	3059826	4	0.026551097	-0.013027713	-0.00353888	*PRDM16*
chr14	105070998	105071714	4	0.026602183	0.014988288	0.010854215	*TMEM179*
chr6	64346520	64346784	3	0.026618316	0.007833165	0.006343505	*PHF3*
chr6	170581690	170581971	3	0.026672527	-0.018557991	-0.005403425	*NA*
chr1	48176494	48176755	3	0.026707951	-0.018820214	-0.010828987	*NA*
chr6	30122388	30123034	6	0.026779227	0.057411012	0.028195137	*TRIM10*
chr1	7074500	7074719	3	0.026798878	-0.026452575	-0.004427897	*CAMTA1*
chr11	66085250	66085453	3	0.026853216	0.01545596	0.008081731	*CD248*
chr12	57943007	57943421	5	0.02687898	-0.029214225	-0.010227936	*DCTN2;KIF5A*
chr9	5811764	5812604	3	0.026914375	-0.046796259	-0.022903236	*ERMP1*
chr5	140479979	140480770	8	0.026914375	0.056735988	0.023927405	*PCDHB3*
chr3	11079491	11079879	4	0.026978592	-0.040531897	-0.018589551	*SLC6A1*
chr6	27342941	27343415	3	0.026985882	0.038424501	0.019754972	*ZNF204P*
chr10	30724020	30724374	4	0.026987792	0.011854131	0.003745507	*MAP3K8*
chr8	25316023	25316123	4	0.02699685	0.011985539	0.005693529	*CDCA2;KCTD9;PPP2R2A*
chr2	132439921	132440047	3	0.02699685	0.058074186	0.032634191	*NA*
chr16	2008723	2009157	4	0.027034581	0.034369142	0.019874673	*NDUFB10;RPL3L*
chr2	236961065	236961189	3	0.027060999	-0.028070156	-0.005062193	*AGAP1*
chr10	12110577	12110799	7	0.027074058	-0.028558716	-0.003236898	*DHTKD1*
chr10	127512288	127512596	3	0.027179236	0.006331234	0.000928336	*BCCIP;UROS*
chr16	31054328	31054411	3	0.027198657	-0.016523414	-0.00754951	*NA*
chr1	110420044	110420800	3	0.027211809	0.034268241	0.011590246	*NA*
chr19	46318918	46319398	4	0.027221056	0.018325299	0.012533421	*RSPH6A;SYMPK*
chr17	81041756	81042023	4	0.027287295	-0.032012312	-0.00697	*METRNL*
chr1	222011091	222011349	4	0.027287295	0.025607738	0.015653393	*NA*
chr15	81426347	81426820	9	0.027288752	-0.046884841	-0.023041827	*CFAP161*
chr7	1265635	1266180	3	0.027293356	-0.038750906	-0.02760816	*NA*
chr3	40519358	40519626	3	0.027360656	-0.035568749	-0.017526059	*ZNF619*
chr16	66955873	66956242	6	0.027388639	0.054524555	0.031340158	*CDH16;RRAD*
chr6	31855906	31856502	14	0.027405142	-0.018528863	-0.004760602	*EHMT2*
chr1	145610622	145611278	17	0.027405142	-0.008697759	-0.000337869	*NBPF10;NBPF20;POLR3C;RNF115*
chr14	24520788	24521326	4	0.027405142	0.01511479	0.007347077	*CARMIL3*
chr3	113615491	113616031	3	0.027444433	-0.051079851	-0.012781113	*GRAMD1C*
chr17	48280257	48280432	4	0.02752504	-0.037499484	-0.017148691	*COL1A1*
chr8	140643119	140643434	3	0.027541398	0.035004235	0.017289078	*KCNK9*
chr12	64238325	64238719	9	0.027738897	0.008904474	0.003094841	*SRGAP1*
chr9	100617710	100618115	4	0.027738897	0.039175613	0.017452089	*FOXE1*
chr8	22799470	22799734	3	0.027747836	-0.023451685	-0.016321841	*NA*
chr1	21501248	21501736	7	0.027780678	-0.042314639	-0.017035453	*EIF4G3*
chr11	72294904	72295168	3	0.027868195	-0.029061897	-0.007055535	*PDE2A*
chr4	3735966	3736042	3	0.027894434	-0.050198335	-0.015859668	*NA*

chr22	38241182	38241410	3	0.027904789	0.03328146	0.000575852	*ANKRD54;EIF3L;MIR658*
chr11	76849101	76849564	4	0.027937139	-0.029968382	-0.023470762	*MYO7A*
chr16	89100786	89100903	3	0.027959647	0.024864664	0.001230378	*NA*
chr4	7195002	7195466	3	0.027982094	-0.038297471	-0.014963624	*SORCS2*
chr6	170102889	170103058	3	0.028055734	-0.01387047	-0.010915622	*C6orf120;WDR27*
chr1	53308654	53309019	4	0.028055734	0.02948447	0.0188387	*ZYG11A*
chr15	57998371	57998781	10	0.028068001	-0.056553833	-0.008237595	*GCOM1;MYZAP;POLR2M*
chr17	36105029	36105517	9	0.028073981	0.026962804	0.005926166	*HNF1B*
chr5	41509849	41510325	5	0.028081176	-0.066076662	-0.03800563	*PLCXD3*
chr14	81999463	81999924	3	0.028081176	-0.045160235	-0.01363466	*SEL1L*
chr8	97247529	97248129	11	0.02830311	-0.02553003	0.000417901	*UQCRB*
chr3	182971025	182971663	7	0.028487964	-0.007650609	-0.000657622	*B3GNT5;MCF2L2*
chr2	217658323	217658498	3	0.028630089	-0.021304179	0.000336495	*NA*
chr2	185462847	185463218	6	0.028669336	-0.017084556	0.00029003	*ZNF804A*
chr15	89911148	89911317	3	0.028689048	0.012820907	0.009718586	*MIR9-3;MIR9-3HG*
chr1	19991237	19992024	5	0.028702277	0.021850802	-0.004224142	*HTR6*
chr1	53792560	53792939	3	0.028718332	0.005950184	0.002744323	*LRP8*
chr17	76713041	76713517	7	0.028718332	0.028804223	0.021711862	*CYTH1*
chr6	163746145	163746319	3	0.028767448	0.039324607	0.027523046	*PACRG-AS1*
chr19	37328842	37328990	3	0.028773429	0.010310071	0.006507676	*ZNF790*
chr6	150219292	150219675	3	0.028977372	-0.033334848	-0.019643023	*RAET1E;RAET1E-AS1*
chr3	193973035	193973521	3	0.029027313	0.010354607	0.004387213	*NA*
chr11	124311399	124311550	4	0.029138269	-0.023279658	-0.002776575	*OR8B8*
chr1	53579371	53579547	3	0.029232361	-0.024357588	-0.013161679	*SLC1A7*
chr6	24774713	24775047	6	0.02927862	-0.048254325	-0.014160241	*GMNN*
chr17	79094415	79094934	3	0.02931293	-0.02078737	-0.008889646	*AATK;MIR3065*
chr17	78774609	78774723	3	0.029352618	-0.014187866	-0.010935132	*RPTOR*
chr6	30139686	30140231	9	0.029375403	-0.027781867	-0.011924865	*TRIM15*
chr20	43439545	43439706	3	0.029385697	-0.074181254	-0.046454932	*RIMS4*
chr5	159626770	159627003	3	0.029392427	0.026905169	0.021201041	*FABP6*
chr4	57396219	57396890	11	0.029410415	0.034895586	0.005754488	*THEGL*
chr4	53728923	53729461	4	0.029451498	0.027379096	0.015911458	*RASL11B*
chr19	52205761	52206258	4	0.029473847	0.039475047	0.015769342	*SPACA6*
chr5	2537496	2537834	6	0.029473847	0.069772198	0.040276609	*NA*
chr12	125023590	125023878	4	0.02948424	0.04595523	0.012809122	*NCOR2*
chr8	104512877	104513083	4	0.029487563	0.012649762	0.006217775	*RIMS2*
chr2	227700114	227700467	5	0.02952953	0.035842736	0.02201009	*RHBDD1*
chr17	37123638	37123949	9	0.029547441	-0.060652729	-0.029859439	*FBXO47*
chr6	31852503	31852982	8	0.029686912	-0.030452792	-0.003562915	*EHMT2*
chr6	31112957	31113086	4	0.02971155	-0.024485805	-0.014296999	*CCHCR1*
chr1	203097234	203097628	7	0.029737275	0.026516028	0.006904199	*ADORA1*
chr17	7452043	7452342	7	0.029745328	-0.008683564	-0.000147404	*TNFSF12;TNFSF12-TNFSF13*
chr8	514736	514821	3	0.029786733	-0.036088087	-0.031581865	*NA*
chr6	25218855	25219327	5	0.029793703	0.051038129	0.012767096	*NA*
chr22	25201812	25202086	7	0.029801017	-0.024945537	-0.009811597	*SGSM1*
chr15	70390440	70391035	6	0.029828889	-0.026045407	-0.002799787	*TLE3*
chr4	128651206	128651707	10	0.029892799	-0.028323731	-0.005522235	*SLC25A31*
chr2	121107018	121107495	4	0.029905695	0.073126976	0.022386841	*INHBB*
chr6	53530503	53530944	7	0.029954055	-0.044409666	0.013368806	*KLHL31*

chr1	34643098	34643124	3	0.029960342	0.035080042	0.020846612	*C1orf94*
chr4	1504989	1505266	4	0.030009683	-0.031011779	-0.019280728	*NA*
chr5	63258311	63258592	3	0.030069762	0.031545908	0.020687242	*HTR1A*
chr12	54393442	54393980	13	0.030112232	-0.024113011	0.00325911	*HOXC9*
chr21	22368940	22369485	3	0.030123185	-0.023699921	-0.017094397	*NCAM2*
chr2	128051708	128051925	5	0.030217453	-0.01451796	-0.003875827	*ERCC3*
chr11	7695165	7695528	8	0.030306072	-0.032282688	-0.008909062	*CYB5R2*
chr7	81400307	81400745	3	0.030309811	-0.029162117	0.00328892	*HGF*
chr12	51449781	51450025	3	0.030405659	-0.019362349	-0.012337065	*LETMD1*
chr9	131181156	131181539	4	0.030429538	0.016697993	0.006751288	*CERCAM*
chr19	5978140	5978790	12	0.030467025	0.01471788	0.00298657	*LOC100128568;RANBP3*
chr1	179555635	179555662	3	0.030471879	0.042255652	0.015454922	*NA*
chr2	241171798	241172573	5	0.030473538	0.038232537	0.003193304	*NA*
chr8	57232395	57232898	5	0.030512084	0.033436624	0.017071524	*SDR16C5*
chr5	172572037	172572253	5	0.030526861	0.015335984	0.008798657	*BNIP1*
chr2	86038423	86038802	4	0.03055858	-0.043388234	-0.024938758	*LOC284950*
chr5	102201490	102202139	9	0.030574679	0.026244809	0.010049167	*PAM*
chr13	47125801	47126551	5	0.03058409	-0.038270152	-0.019618308	*LRCH1*
chr2	9865047	9865477	3	0.030619694	0.023219212	0.002474584	*NA*
chr13	46038653	46039090	7	0.030699699	0.035013058	0.006187605	*COG3*
chr13	112627458	112627641	3	0.030717904	0.080638075	0.034899903	*NA*
chr2	11101549	11102598	6	0.030747967	-0.045235488	-0.020633385	*NA*
chr12	123756720	123756791	4	0.0308123	0.010183554	0.002534152	*CDK2AP1*
chr5	19988501	19988944	11	0.030843842	0.046654817	0.004827582	*CDH18*
chr15	27109950	27110089	3	0.030858243	0.010608039	0.006067718	*GABRA5;GABRB3*
chr6	114177341	114178220	4	0.030939783	0.009897668	0.001963638	*MARCKS*
chr5	172185151	172185217	3	0.030981986	-0.034074553	-0.020697977	*NA*
chr10	4193830	4194452	6	0.03099637	-0.025261539	-0.009166583	*NA*
chr8	117768886	117769417	3	0.031034208	-0.047946275	-0.021239177	*EIF3H*
chr3	16524387	16524574	4	0.031173299	0.027019691	0.018551076	*RFTN1*
chr17	56744069	56744413	5	0.031206347	-0.050082492	-0.022719932	*TEX14*
chr7	157932715	157932971	4	0.031289132	-0.07007438	-0.032669172	*PTPRN2*
chr14	51940694	51940935	3	0.031290197	-0.05607325	-0.031752352	*FRMD6-AS2*
chr2	148602793	148602993	3	0.031303238	-0.013173545	-0.001966569	*ACVR2A*
chr3	54154438	54154926	5	0.031363269	-0.030798144	-0.020729189	*CACNA2D3*
chr12	54346784	54347286	5	0.031363269	0.043958534	0.021365457	*HOXC12*
chr2	119916431	119916807	7	0.031382396	0.033143823	0.002272945	*C1QL2*
chr7	6413861	6414209	7	0.031412714	-0.023075961	-0.003675847	*RAC1*
chr6	134272191	134272463	3	0.031421582	0.034551034	-0.004214901	*TBPL1*
chr22	42378942	42379527	3	0.031435867	0.03418586	0.025663254	*SEPTIN3*
chr13	27316791	27317381	3	0.03153374	-0.081423203	-0.052120528	*NA*
chr22	31500397	31500940	3	0.03153374	-0.034030727	-0.026371139	*INPP5J;SELENOM;SMTN*
chr1	47081524	47082039	3	0.031651632	0.034959822	0.022938741	*MKNK1;MOB3C*
chr17	75494607	75494669	4	0.031739758	-0.029850326	-0.007365919	*SEPTIN9*
chr10	124913209	124913724	8	0.031757103	0.024908509	0.004126251	*BUB3*
chr5	10306947	10307744	5	0.031758248	-0.014938632	-0.000177154	*CMBL*
chr1	155107462	155108011	8	0.031829614	-0.037282777	-0.006854194	*SLC50A1*
chr11	31848828	31849262	6	0.031880329	0.020477892	0.009729607	*PAX6-AS1;RCN1*
chr1	201508712	201509415	6	0.031912674	0.025519602	0.006064861	*NA*

chr10	64578186	64578476	3	0.031915411	-0.036380766	-0.015964249	*EGR2*
chr7	73731621	73732108	5	0.031915411	-0.024386174	-0.007467202	*CLIP2*
chr15	66084798	66085298	8	0.031929705	-0.015982077	-0.00255147	*DENND4A*
chr22	46372501	46372744	5	0.031934739	-0.029992554	-0.008215118	*WNT7B*
chr4	175132842	175133103	3	0.031954547	0.012651967	0.011331164	*NA*
chr8	18577100	18577488	3	0.031993981	0.030513771	-0.001734622	*PSD3*
chr20	50349254	50349428	4	0.032012049	0.030984959	0.016098575	*ATP9A*
chr14	72053146	72053361	4	0.032048302	0.044717787	0.030619992	*SIPA1L1*
chr8	27449907	27450279	5	0.03213529	-0.016254534	-0.000966914	*NA*
chr6	27533825	27534346	4	0.03214867	0.034564978	0.009139847	*NA*
chr7	106808956	106809324	7	0.032149906	0.007003254	0.001507164	*HBP1*
chr19	4102976	4103401	5	0.032197917	-0.025092806	-0.014245852	*MAP2K2*
chr16	75252355	75252973	8	0.032450983	-0.040654233	-0.012427958	*CTRB1*
chr5	125757888	125758506	3	0.032472475	-0.030663964	-0.022376966	*GRAMD2B*
chr3	128204813	128205496	7	0.032476857	0.02749941	0.013119154	*GATA2*
chr6	156718177	156718546	5	0.032708223	0.042763904	0.029497265	*NA*
chr1	153940985	153941285	3	0.032868271	0.046519325	0.02727012	*CREB3L4;SLC39A1*
chr17	7487593	7487853	3	0.032987522	-0.045557073	-0.019688357	*MPDU1*
chr10	134226361	134226547	3	0.033058242	0.035604183	0.021037948	*PWWP2B*
chr3	36756049	36756085	3	0.033070964	-0.02654684	0.002612309	*DCLK3*
chr19	59069988	59070754	11	0.033102862	-0.011297057	-0.000920556	*CHMP2A;MZF1-AS1;UBE2M*
chr20	44098223	44098724	7	0.033123884	-0.032772241	-0.004932198	*WFDC2*
chr6	30884915	30885205	5	0.033257518	0.02170076	0.00253642	*VARS2*
chr16	77270057	77270312	3	0.033262521	-0.023119003	-0.006358761	*NA*
chr10	97453638	97454076	10	0.033321177	-0.018125501	-0.003851481	*TCTN3*
chr4	84035891	84036081	3	0.033321177	0.012320453	0.009071478	*PLAC8*
chr13	41556498	41556827	3	0.03333674	0.032608694	0.023678669	*ELF1*
chr12	54329854	54330354	5	0.033369543	0.042294608	0.015971427	*HOXC13;HOXC13-AS*
chr6	10425553	10425648	3	0.033466602	0.020562479	0.014320902	*NA*
chr20	45279985	45280466	9	0.033556618	0.048402106	0.007611352	*SLC13A3*
chr20	62205530	62205981	8	0.033618675	0.015781389	0.003298557	*HELZ2*
chr19	31842873	31843396	3	0.03364172	0.023872244	0.014718548	*TSHZ3*
chr1	107682283	107682504	3	0.033720758	0.017590275	0.014139746	*NTNG1*
chr8	144328500	144328928	4	0.033854685	-0.014368791	0.001585159	*ZFP41*
chr3	48263670	48264021	4	0.03385559	-0.015997802	-0.013359976	*CAMP*
chr13	113121107	113121280	3	0.03385559	-0.017710355	-0.004500661	*NA*
chr12	12849443	12850082	5	0.033863793	-0.019441492	-0.015697684	*GPR19*
chr12	104680384	104681435	10	0.03387988	0.013673776	-0.001919241	*TXNRD1*
chr4	66535655	66535890	5	0.033924739	0.02396325	0.007207766	*EPHA5;EPHA5-AS1*
chr1	38412519	38412711	4	0.03394875	0.018173837	0.014430627	*INPP5B*
chr6	17393365	17393825	9	0.034003367	0.048311058	0.013889945	*CAP2*
chr5	68485177	68485690	8	0.034010711	0.012064064	0.002079929	*CENPH*
chr3	147131146	147131617	5	0.034137195	0.021251098	0.011123481	*ZIC1*
chr11	6291447	6291927	5	0.034153507	-0.063907877	-0.042852241	*CCKBR*
chr11	66488638	66489022	7	0.034191101	-0.022309187	-0.002879784	*SPTBN2*
chr2	150176791	150177277	4	0.034201271	-0.053523858	-0.033373302	*NA*
chr5	74162771	74162924	6	0.034243176	-0.025233654	0.000982657	*FAM169A*
chr11	8710564	8710910	5	0.034303305	-0.010314017	-0.004718268	*RPL27A*
chr10	50747578	50747793	6	0.034303305	0.007205017	0.003654319	*ERCC6*

chr13	61989597	61990113	11	0.034310049	-0.027984275	-0.000649389	*PCDH20*
chr5	174159294	174159705	3	0.034323416	-0.026125551	-0.015464155	*NA*
chr2	191085958	191086075	3	0.034379517	-0.022786258	-0.01271785	*HIBCH*
chr1	229568930	229569101	4	0.034379517	0.051386991	0.026510666	*ACTA1*
chr10	134504552	134504836	3	0.034536011	-0.017735227	-0.001813493	*INPP5A*
chr15	89630450	89630886	3	0.034576789	-0.01424676	-0.013078736	*ABHD2*
chr17	17464853	17465476	7	0.034578891	0.031852756	0.021715009	*PEMT*
chr6	100911526	100912168	8	0.034583305	0.038500069	0.005639505	*SIM1*
chr8	15096560	15096879	3	0.034601896	-0.036286362	-0.025705126	*SGCZ*
chr7	128469741	128469891	4	0.03464205	-0.037721931	-0.007140058	*FLNC*
chr17	72848535	72848918	3	0.03464205	0.021450388	0.011425534	*GRIN2C*
chr10	81074718	81075165	4	0.0347151	-0.023185771	-0.001592317	*ZMIZ1*
chr17	41607535	41608535	9	0.034743082	0.023260266	0.013161255	*ETV4*
chr18	23805713	23806263	6	0.034905313	-0.024379671	-0.002064749	*TAF4B*
chr4	175443752	175444181	6	0.034935875	0.010933081	0.003583708	*HPGD*
chr6	41336966	41337238	3	0.034952062	0.011490235	0.009467983	*NA*
chr19	58095011	58095206	3	0.034952062	0.023301388	0.012349036	*ZIK1;ZNF416*
chr2	39351157	39351732	6	0.034994475	-0.011012987	0.000214776	*SOS1*
chr16	30034426	30034487	3	0.035043225	-0.024019425	-0.015312365	*BOLA2;C16orf92*
chr17	59478953	59479139	3	0.035136788	0.022678949	0.016016157	*TBX2*
chr6	33216195	33216663	15	0.035152274	0.028303132	0.003196379	*HCG25*
chr9	95838464	95838971	3	0.035232425	0.018252706	0.001299167	*SUSD3*
chr8	55370171	55370407	5	0.035250412	-0.025786588	-0.010702467	*SOX17*
chr5	50674729	50675114	3	0.035259207	0.015133364	0.007705621	*ISL1;LOC642366*
chr1	8485376	8485666	4	0.035432327	-0.028606052	0.002621052	*RERE*
chr8	33864284	33864644	3	0.035471558	0.028712045	-0.003196618	*NA*
chr12	50504698	50505163	3	0.035513388	0.021513171	0.005044514	*COX14;GPD1*
chr2	85838476	85838835	5	0.035564637	0.037052954	0.009031413	*C2orf68;USP39*
chr16	27215225	27215372	4	0.035584804	-0.022615705	-0.005821004	*KDM8*
chr19	375831	376532	7	0.035599845	-0.039760274	-0.008223149	*THEG*
chr8	82598608	82598903	6	0.035654923	0.021742384	0.006190213	*IMPA1*
chr12	132841141	132841405	3	0.035676326	-0.044302713	-0.027911904	*GALNT9*
chr5	122425156	122425425	4	0.035685286	0.017734628	0.004453786	*PRDM6*
chr13	114890016	114890416	4	0.035696067	0.015163525	0.010864777	*RASA3*
chr2	242628842	242629078	3	0.035830337	-0.04833677	-0.041877452	*DTYMK*
chr7	101754758	101755186	4	0.035855196	-0.021352509	0.002257869	*CUX1*
chr1	3567550	3568004	9	0.035961826	0.022710786	0.010300147	*TP73;WRAP73*
chr16	48201121	48201296	4	0.035977388	-0.021290263	-0.000616059	*ABCC11*
chr14	74486312	74486664	4	0.036135089	0.021066516	0.004811677	*BBOF1;ENTPD5*
chr17	8378756	8379225	5	0.036146011	0.021463545	0.000200315	*MYH10*
chr16	10775706	10775897	3	0.036168541	0.044175612	0.008725337	*TEKT5*
chr9	140095212	140095555	7	0.036314395	0.009313549	0.003652914	*NDOR1;TPRN*
chr1	27560800	27560916	7	0.036330429	0.007459802	0.000853216	*WDTC1*
chr22	46637754	46638155	3	0.036330429	0.034708457	0.019006338	*PPARA*
chr21	46340905	46341304	6	0.036392815	0.019941608	0.009447747	*ITGB2;ITGB2-AS1*
chr18	35065040	35065386	3	0.036392815	0.030441902	0.019086841	*CELF4*
chr5	150827158	150827728	6	0.036413102	0.007131406	0.000312844	*SLC36A1*
chr2	183943319	183943938	9	0.036570281	0.021589578	0.010944602	*DUSP19*
chr4	147034625	147034848	3	0.036722731	-0.023669822	-0.01470599	*LINC01095*

chr7	123198173	123198553	7	0.036726581	0.043604769	0.012668596	*NDUFA5*
chr12	48722474	48722754	4	0.036768419	0.021582024	0.008471835	*H1-7*
chr1	228346181	228346347	3	0.036810837	-0.026999911	-0.00818036	*GJC2*
chr9	18472617	18473018	4	0.036834193	-0.04134545	-0.014884036	*ADAMTSL1*
chr1	99470550	99470801	5	0.036860459	0.017486104	0.007574696	*LOC100129620;PLPPR5*
chr6	33288180	33288599	8	0.036905033	-0.023148767	-0.006443934	*DAXX;ZBTB22*
chr20	33734751	33735232	9	0.036982035	0.008959429	0.002876244	*EDEM2*
chr4	185329653	185329758	3	0.036984354	-0.014718264	-0.008696671	*IRF2*
chr3	160475035	160475336	5	0.037038036	-0.035518574	-0.017487363	*PPM1L*
chr10	6962119	6962454	6	0.037084948	0.028521908	0.008902593	*NA*
chr12	56551917	56552132	8	0.037117736	-0.011771426	0.002761995	*MYL6*
chr17	48912591	48912952	5	0.037149923	-0.041666324	-0.014926964	*WFIKKN2*
chr1	157811303	157811905	5	0.037149923	-0.018418552	-0.012614678	*CD5L*
chr8	135725535	135725874	3	0.037152474	-0.003624629	0.000736032	*ZFAT*
chr3	69134395	69134502	3	0.037198404	0.005491167	0.002751335	*ARL6IP5;UBA3*
chr3	129261686	129262055	5	0.037217159	-0.020378118	-0.009759591	*H1-8*
chr3	180042484	180042726	3	0.037234371	-0.03229245	-0.019600869	*NA*
chr9	38392470	38393112	7	0.037234371	0.013570941	0.003973544	*ALDH1B1*
chr5	7816713	7817324	3	0.037268868	0.022209047	0.005657694	*ADCY2*
chr14	70833186	70833404	3	0.037283695	0.031113185	-0.003227934	*SYNJ2BP;SYNJ2BP-COX16*
chr19	50379397	50379958	8	0.037283695	0.009941519	0.001550408	*AKT1S1;TBC1D17*
chr21	47456404	47456916	5	0.037366146	-0.026756968	-0.007221897	*NA*
chr10	13769634	13770043	4	0.037488219	-0.022593042	-0.010580865	*FRMD4A*
chr11	76368494	76368694	3	0.037488219	0.111725438	0.081328998	*LRRC32*
chr22	40573054	40573204	3	0.037505203	-0.028068345	-0.015338885	*TNRC6B*
chr18	48346448	48346843	9	0.037512369	0.023964124	0.005986008	*MRO*
chr16	57770483	57770866	3	0.037575782	0.013021776	0.006375881	*KATNB1*
chr2	71211940	71212157	4	0.037587362	0.035584468	0.024125619	*ANKRD53*
chr20	31823137	31823545	4	0.037604217	-0.033961804	-0.004530169	*BPIFA1*
chr1	102462590	102463043	9	0.037609867	0.042261348	0.008682328	*OLFM3*
chr17	71258589	71259141	6	0.037719518	0.028954331	0.00921535	*CPSF4L*
chr7	38948782	38949140	4	0.037734909	0.024665446	0.011036741	*VPS41*
chr22	50524374	50524691	4	0.037802018	0.032003735	0.011411943	*MLC1;MOV10L1*
chr1	161136002	161136321	9	0.037831207	-0.005304305	0.000102093	*PPOX*
chr20	22564325	22564947	3	0.037831207	0.015241147	0.005297566	*FOXA2*
chr16	56672387	56672722	7	0.038179052	0.026269511	0.008969595	*MT1A;MT1DP*
chr7	1952518	1952807	3	0.038321748	0.048255358	0.0279387	*MAD1L1*
chr6	37474771	37474946	3	0.038340402	-0.022099494	0.001054151	*NA*
chr8	37553131	37553339	7	0.038360492	-0.017214013	-0.001893439	*ZNF703*
chr1	35324574	35324844	5	0.038360492	0.022069672	0.009363129	*SMIM12*
chr6	34003954	34004360	3	0.038415014	-0.026039277	-0.005693867	*GRM4*
chr5	178772203	178772390	4	0.038627165	-0.016959241	-0.01035634	*ADAMTS2*
chr5	95767724	95767946	5	0.03866162	0.02655554	0.011911912	*PCSK1*
chr11	2321770	2322050	8	0.038687647	0.023356101	0.009058778	*C11orf21;TSPAN32*
chr11	59522279	59523399	14	0.038737669	0.00985826	0.003039315	*STX3*
chr14	23352029	23352504	5	0.038760452	-0.046577336	-0.027413854	*REM2*
chr10	27547436	27547704	4	0.038760452	-0.028615731	-5.10E-05	*NA*
chr7	157451243	157451363	3	0.038760452	0.03612822	0.020491898	*PTPRN2*
chr16	72821353	72821665	4	0.038830847	0.023874194	0.005897535	*ZFHX3*

chr1	201252324	201252974	9	0.038872544	-0.021252763	-0.001327358	*PKP1*
chr18	60264247	60264554	3	0.038907159	-0.024762794	-0.011050641	*NA*
chr22	18258244	18258664	3	0.038934777	0.019766614	0.014173041	*BID*
chr13	40107605	40107878	3	0.039040086	0.022066989	-0.003428105	*LHFPL6*
chr13	111973089	111973143	3	0.039183406	-0.057218602	-0.0471512	*TEX29*
chr17	17603531	17603837	4	0.03921204	-0.042215911	-0.022749296	*RAI1*
chr19	14671371	14671512	3	0.03921204	0.033179643	0.012986166	*TECR*
chr21	44720919	44721077	3	0.039385668	-0.030218498	-0.021790395	*NA*
chr1	18700891	18700971	3	0.039481849	0.056399554	0.031365786	*IGSF21*
chr6	105584551	105584780	5	0.039658571	0.022509711	0.012042588	*BVES;BVES-AS1*
chr7	99102011	99102090	3	0.039779536	0.025886537	0.0072533	*ZKSCAN5;ZNF394*
chr9	138966848	138967347	4	0.03992751	-0.028124043	-0.022356401	*NACC2*
chr6	31554558	31554946	4	0.039977425	0.01569364	0.006283799	*LST1;LTB*
chr5	149669194	149669368	3	0.040159208	-0.036572524	-0.033694864	*CAMK2A*
chr3	126423056	126423272	7	0.040203367	-0.009268911	0.000597946	*CHCHD6*
chr16	69965257	69965788	5	0.040239321	0.027174159	0.008523406	*MIR140;WWP2*
chr17	80747189	80747240	3	0.040266715	-0.024539285	-0.008443056	*TBCD*
chr14	70655803	70656116	8	0.040277645	-0.017887987	-0.000437896	*SLC8A3*
chr17	13210168	13210241	3	0.04044504	-0.05232048	-0.004464018	*NA*
chr4	863878	864100	3	0.04044504	0.042554465	0.017730088	*GAK*
chr4	8359269	8359499	3	0.040454203	-0.031119808	-0.029498668	*NA*
chr1	160854954	160855200	4	0.040840743	0.028496815	0.023308137	*ITLN1*
chr17	17184440	17184692	7	0.040890687	0.013065481	0.003405474	*COPS3*
chr2	149895023	149895553	4	0.040947057	0.013803451	-0.000396689	*LYPD6B*
chr1	159796231	159796434	3	0.040970529	0.016057995	0.010573356	*SLAMF8*
chr20	60582478	60582830	4	0.041094323	-0.026304412	-0.007722361	*TAF4*
chr12	132905735	132906010	3	0.041400903	-0.024760322	-0.000349538	*GALNT9*
chr11	45939458	45939543	3	0.041657131	-0.011591138	-0.000801236	*LARGE2;PEX16*
chr16	73093296	73093652	4	0.041788727	-0.018758623	0.003826829	*ZFHX3*
chr20	62318426	62318588	5	0.041817162	0.022488356	0.011910319	*RTEL1;RTEL1-TNFRSF6B*
chr2	12858298	12859103	7	0.04182885	0.023081192	0.000943224	*TRIB2*
chr19	39440462	39440924	6	0.041879328	0.045486357	0.011436177	*FBXO17;SARS2*
chr9	6565526	6565777	3	0.041919731	-0.020873956	0.006416565	*GLDC*
chr19	39369277	39369353	3	0.041969591	0.019796273	0.014687098	*RINL;SIRT2*
chr6	17281483	17281626	5	0.042073599	0.018564994	0.007074832	*RBM24*
chr8	142427930	142428240	3	0.042155774	-0.01264932	-0.006552417	*PTP4A3*
chr2	233246136	233246568	3	0.042238149	-0.06955283	-0.044345428	*ALPP*
chr6	33130918	33131336	5	0.042296519	-0.019515682	-0.004780677	*COL11A2*
chr5	10413341	10413382	3	0.042361363	0.02693549	0.01401922	*MARCHF6*
chr14	94595914	94596337	9	0.042388697	0.007546754	0.003754979	*IFI27L2*
chr8	125985352	125985468	3	0.0424692	0.015225543	0.006797513	*ZNF572*
chr17	46816036	46816347	3	0.042473292	0.018555749	0.015954742	*NA*
chr1	61542546	61542979	11	0.042509787	0.029796029	0.00293844	*NFIA*
chr10	37940493	37940899	3	0.042725259	-0.052806269	-0.047970414	*NA*
chr12	51566569	51566914	7	0.043219104	-0.022011053	-0.002870736	*TFCP2*
chr4	111480175	111480200	3	0.043254351	-0.014982227	-0.005722105	*ENPEP*
chr1	1296956	1297240	5	0.043321194	0.045920491	0.030291177	*MXRA8*
chr20	23113228	23113340	3	0.043520932	0.033740281	0.022326245	*LINC00656*
chr14	34077052	34077154	3	0.043566592	-0.03961816	-0.021001846	*NPAS3*

chr17	18854560	18854799	5	0.043630302	-0.017232879	-0.001709554	*SLC5A10*
chr1	179335022	179335216	4	0.043642125	-0.008278479	0.001321418	*AXDND1*
chr9	139247370	139247436	3	0.043676695	-0.03712294	-0.019264869	*GPSM1*
chr1	151763981	151764147	3	0.043901881	-0.031324845	-0.022644543	*TDRKH*
chr2	97427027	97427102	3	0.044186986	-0.011029679	-0.008175425	*CNNM4*
chr12	89413599	89413666	3	0.044280541	0.061629098	0.030846927	*LINC02458*
chr2	1859035	1859146	3	0.04437071	-0.039501635	-0.031838752	*MYT1L*
chr1	76080684	76081039	4	0.04444906	-0.024085177	-0.003971611	*SLC44A5*
chr1	2082315	2082848	5	0.044459845	0.018822929	0.003507067	*PRKCZ*
chr10	80961305	80961382	4	0.04447365	0.022689399	0.004049222	*ZMIZ1*
chr12	32111257	32111398	3	0.044511835	-0.047408506	-0.012279591	*RESF1*
chr10	70847350	70847430	3	0.0445715	0.006415038	0.002624074	*SRGN*
chr12	120907301	120907610	4	0.04473103	-0.009008605	-0.003018768	*DYNLL1;SRSF9*
chr20	11873064	11873102	3	0.044855569	0.007854351	-0.000587084	*BTBD3*
chr8	55243060	55243181	3	0.04489513	-0.028888327	-0.005044687	*NA*
chr12	109569059	109569180	5	0.044897988	0.039552511	0.022180523	*ACACB*
chr10	11934150	11934469	5	0.044940881	0.032414207	0.011016736	*PROSER2-AS1*
chr6	887704	887821	3	0.045098182	-0.06840693	-0.044008675	*NA*
chr7	27225058	27225342	10	0.045166551	-0.010173318	-0.003632867	*HOXA11;HOXA11-AS*
chr3	47517621	47517819	4	0.045166838	-0.027828351	-0.014987758	*SCAP*
chr17	33913801	33913985	4	0.045166838	0.051161095	0.016597199	*AP2B1*
chr8	56056357	56056609	3	0.045314378	0.029161224	0.027256249	*XKR4*
chr11	10750870	10750973	3	0.045330043	-0.039634431	-0.019675708	*NA*
chr12	10955358	10955577	4	0.045510057	0.042043913	0.003379491	*TAS2R7*
chr13	112553087	112553309	3	0.045746781	-0.027971709	-0.007736589	*NA*
chr6	139116947	139117182	4	0.045781249	-0.035292968	-0.022556062	*ECT2L*
chr15	63334937	63335016	3	0.045844556	-0.009658461	-0.005192043	*TPM1*
chr2	198268382	198268478	3	0.045958727	-0.038822601	-0.028769419	*SF3B1*
chr11	18610297	18610557	8	0.045958727	-0.019424327	-0.00285955	*UEVLD*
chr4	57688173	57688337	4	0.046005233	0.020310929	0.007998607	*SPINK2*
chr9	99540614	99540647	3	0.046284601	0.03108417	0.018053428	*ZNF510*
chr6	30419051	30419135	5	0.046331026	-0.02571422	-0.001561038	*NA*
chr16	2031324	2031785	3	0.046416314	-0.029132505	-0.008667477	*GFER;NOXO1*
chr10	23216299	23216695	3	0.046416314	-0.016022731	0.000940879	*ARMC3*
chr8	67405257	67405484	4	0.04648175	0.035524511	0.002932097	*VXN*
chr6	31940467	31940692	8	0.046595211	0.024735602	0.004789512	*DXO;STK19*
chr1	185014181	185014395	7	0.046733784	0.016314977	-0.002060273	*RNF2*
chr7	910388	910479	3	0.047008079	0.011671693	0.009722705	*GET4;SUN1*
chr3	197676952	197677174	6	0.047048145	0.032822823	0.01670325	*IQCG;RPL35A*
chr10	111682808	111682948	4	0.047055301	-0.006879082	0.00102742	*XPNPEP1*
chr17	79895300	79895532	9	0.047065369	0.013353905	0.00279911	*PYCR1*
chr4	53588360	53588397	3	0.047129054	-0.018759225	-0.00745573	*NA*
chr11	117134326	117134453	3	0.047196406	-0.038809889	-0.007064357	*RNF214*
chr5	175793484	175793541	3	0.047196406	0.021335136	-0.003060352	*ARL10;KIAA1191*
chr2	220119321	220119493	4	0.047611147	-0.009197379	-0.004171225	*TUBA4A;TUBA4B*
chr6	84419189	84419360	7	0.047681779	0.020764516	0.013098188	*SNAP91*
chr11	1954966	1955023	3	0.047742101	0.021270573	-0.000505863	*TNNT3*
chr10	6263432	6263606	3	0.047876055	0.016165533	0.008904962	*PFKFB3*
chr18	28622912	28623090	6	0.048189261	0.038149175	0.004074731	*DSC3*

chr10	44071073	44071220	3	0.048399782	-0.030098104	-0.007098963	*ZNF239*
chr7	151565595	151565731	4	0.048423474	-0.02326405	-0.009956314	*PRKAG2*
chr14	95047591	95047616	3	0.048510248	0.025942151	0.014066735	*SERPINA5*
chr15	41624609	41624823	5	0.048690825	0.011575209	0.003602943	*NUSAP1;OIP5*
chr2	10545728	10545842	3	0.048762059	0.018359589	0.002600169	*HPCAL1*
chr16	56623105	56623215	5	0.048894263	-0.020369158	-0.003100256	*MT3*
chr11	112096999	112097073	5	0.049287294	0.006195493	0.002279008	*PTS*

Supplemental Table 11 - Differentially Methylated Regions (DMRs) of the unhealthy living subjects

Chromosome	Start	End	Number CpGs	Min smoothed FDR	Max difference	Mean difference	UCSC RefGene name
chr19	55972504	55973879	14	2.87E-10	0.070785226	0.033558233	*ISOC2*
chr3	195538675	195539463	13	1.10E-09	0.056128467	0.018726509	*MUC4*
chr6	33047944	33049360	18	1.16E-06	0.072196942	0.045654839	*HLA-DPA1;HLA-DPB1*
chr11	287938	290447	11	2.55E-06	0.147092657	0.009104691	*PGGHG*
chr18	23713407	23714084	12	9.91E-06	-0.061564222	-0.02995451	*PSMA8*
chr19	51017311	51018414	9	1.01E-05	0.047387994	0.028508778	*ASPDH;JOSD2*
chr20	13975439	13976782	16	1.51E-05	0.071706685	0.022461694	*MACROD2;SEL1L2*
chr8	49825415	49826049	3	2.77E-05	-0.046066582	-0.033946588	*NA*
chr17	37893638	37894413	10	3.94E-05	0.05012333	0.01612182	*GRB7*
chr7	39170497	39171113	6	5.87E-05	0.088542102	0.07574728	*POU6F2*
chr19	44083688	44084933	7	0.000164183	-0.035675122	-0.016062865	*PINLYP;XRCC1*
chr2	157183755	157184978	10	0.000208432	0.034798967	0.021531783	*NR4A2*
chr1	175162044	175163165	14	0.000334664	0.047428479	0.015933478	*KIAA0040*
chr2	97536097	97536620	4	0.000370904	-0.015103046	-0.00294426	*SEMA4C*
chr10	81964234	81964782	6	0.000385096	0.028486452	0.015714829	*ANXA11;LINC00857*
chr10	816636	817083	3	0.000548282	0.040113087	0.03263143	*NA*
chr19	55660514	55661387	9	0.00111322	-0.066084531	-0.03607225	*TNNT1*
chr4	74847100	74848392	9	0.001216075	-0.084619386	-0.041157753	*PF4*
chr5	148928885	148929352	4	0.00128476	-0.04970126	-0.028365955	*CSNK1A1*
chr1	152161237	152162025	7	0.001516235	0.109763198	0.063258436	*NA*
chr3	57112957	57113767	8	0.001516235	-0.027971517	-0.012507132	*ARHGEF3*
chr6	100915395	100915805	3	0.002225024	0.026908364	0.021342066	*SIM1*
chr17	42733368	42733994	11	0.002225024	-0.036122312	-0.014453254	*MEIOC*
chr2	9613721	9614225	6	0.002225024	-0.039489736	-0.028153718	*IAH1*
chr13	27999177	27999546	4	0.002225024	-0.056829261	-0.044608354	*GTF3A*
chr10	133796164	133796559	6	0.002479151	-0.038966559	-0.018949861	*BNIP3*
chr19	17905332	17905966	8	0.002490081	0.098576262	0.034064315	*B3GNT3*
chr14	34420102	34421172	14	0.002621856	0.036159802	0.004122311	*EGLN3*
chr5	172710181	172710766	4	0.00285111	0.055943695	0.028399881	*NA*
chr20	36148375	36149455	33	0.002975425	0.038587855	0.02264364	*BLCAP;NNAT*
chr17	7253189	7253720	8	0.004836382	0.053859024	0.033659823	*ACAP1;KCTD11;TMEM95*
chr2	20870812	20871401	6	0.005134558	0.098580662	0.077144573	*GDF7*
chr22	51016386	51016703	7	0.005217864	-0.064737215	-0.040599729	*CHKB-CPT1B;CHKB-DT;CPT1B*
chr15	42566151	42566390	9	0.005570324	-0.04710759	-0.011768291	*GANC;TMEM87A*
chr10	104613613	104614136	9	0.00677745	-0.050440385	-0.016587167	*BORCS7;BORCS7-ASMT*

chr15	49716247	49716645	3	0.007232806	0.061602889	0.048002674	*FAM227B;FGF7*
chr11	69001490	69001551	3	0.008479918	0.038011612	0.025970061	*NA*
chr16	85699435	85699754	3	0.008599968	0.01981288	0.01377646	*GSE1*
chr6	38682982	38683221	5	0.009159337	0.051039721	0.02107384	*DNAH8*
chr16	85813931	85814166	3	0.009159337	0.038375739	0.017853698	*EMC8*
chr6	6007259	6007523	4	0.009159337	-0.025391706	-0.001366854	*NRN1*
chr16	3114847	3115286	6	0.009563444	-0.033192361	-0.020138715	*IL32*
chr3	48694246	48695065	6	0.010108117	-0.065221325	-0.036217824	*CELSR3*
chr19	59084588	59085030	9	0.011066527	0.021712096	-0.000633246	*CENPBD1P1;MZF1;MZF1-AS1*
chr6	31683051	31683352	6	0.011270643	0.035220869	0.023491502	*ABHD16A;LY6G6D;LY6G6E;LY6G6F*
chr7	5646827	5647332	4	0.012006205	0.027548471	0.014094085	*NA*
chr15	69744390	69744850	6	0.012076657	-0.057497918	-0.038431003	*RPLP1*
chr19	55549414	55549907	9	0.012076657	-0.066246498	-0.03962099	*GP6;RDH13*
chr20	49609650	49609852	3	0.012143799	-0.065363796	-0.03890605	*NA*
chr7	4746131	4746462	3	0.01292798	0.048130549	0.026962852	*FOXK1*
chr5	1849983	1850261	3	0.014001454	0.082060281	0.057560399	*NA*
chr20	39995539	39995782	7	0.014001454	0.019203519	0.00214305	*EMILIN3*
chr22	22090796	22090816	3	0.014001454	-0.048570568	-0.044953063	*YPEL1*
chr5	176754987	176755542	5	0.016117543	-0.048916036	-0.034284864	*NA*
chr21	43655256	43655919	3	0.016141899	0.029192007	0.023415273	*ABCG1*
chr14	32029920	32030686	11	0.018788863	-0.032984941	-0.003535851	*NUBPL*
chr11	31825756	31826272	5	0.018788863	-0.051037036	-0.018543277	*PAX6*
chr9	34370781	34371380	5	0.018925854	0.072310563	0.039149332	*MYORG*
chr1	160254873	160255036	4	0.020523063	-0.015646648	-0.008981092	*DCAF8;PEX19*
chr10	29697905	29698462	8	0.021618425	-0.042020791	-0.019079925	*PTCHD3P1*
chr7	27204005	27204349	4	0.022844831	0.026624772	0.011267243	*HOXA10-AS;HOXA10-HOXA9;HOXA9*
chr1	230404249	230404313	3	0.023356295	0.023797847	0.007858082	*GALNT2*
chr1	159869902	159870326	8	0.024154007	0.041232843	0.026256937	*CFAP45*
chr8	109094793	109095264	3	0.024201371	0.018786655	0.008494278	*RSPO2*
chr5	137224904	137225509	8	0.024880325	-0.029378903	-0.018093596	*PKD2L2*
chr1	154475068	154475173	3	0.025495774	0.033931174	0.016525354	*SHE;TDRD10*
chr6	31734106	31734580	9	0.027889161	0.056075982	0.031970956	*VWA7*
chr17	72948349	72948705	3	0.028326213	0.054681263	0.042170405	*HID1*
chr5	133702676	133702899	7	0.029310762	-0.029101329	0.001947841	*CDKL3;UBE2B*
chr3	195578011	195578280	5	0.029657503	0.058899581	0.023622064	*NA*
chr15	45028083	45028595	5	0.029802042	0.041972964	0.024322363	*TRIM69*
chr12	4380586	4381019	6	0.031264055	0.041843822	-0.003207697	*CCND2*
chr19	55314542	55314972	5	0.033793329	-0.045333659	-0.017791801	*KIR2DL4;KIR2DS4;KIR3DL1*

chr20	388519	388903	10	0.034081645	-0.055622439	-0.013765744	*RBCK1*
chr21	34185960	34186122	4	0.037943435	0.028312462	0.016198014	*C21orf62*
chr16	68003120	68003289	3	0.037943435	-0.007586598	-0.003268796	*SLC12A4*
chr18	60052048	60052464	4	0.038197296	0.044848129	0.039709693	*TNFRSF11A*
chr9	138628212	138628332	6	0.040127203	-0.021185058	-0.001987984	*KCNT1*
chr12	110888828	110888956	3	0.040553074	0.033732267	0.025298139	*ARPC3*
chr11	68607622	68607737	4	0.04102295	-0.013757161	-0.009294782	*CPT1A*
chr14	75746793	75747047	3	0.043191832	0.045599914	0.035002417	*FOS*
chr6	30882994	30883074	3	0.043191832	0.048439031	0.033092494	*VARS2*
chr8	101224915	101225361	7	0.043191832	0.072738476	0.027808064	*SPAG1*
chr16	20085047	20085214	4	0.043905228	0.035853591	0.020077634	*GPR139*
chr3	6902824	6902845	3	0.045287758	0.059436076	0.029474252	*GRM7*

Supplemental Table 12 - KEGG pathway enrichment analysis of the healthy living subjects

ID	Description	Gene ratio	Bg ratio	*P*-value	FDR	*q*-value	Gene ID	Count	Fold enrichment
hsa05032	Morphine addiction	20/564	91/8105	3.09E-06	0.00098	0.000837	PRKACA/GNG4/GNG7/GABRA1/GABRA5/GABRB3/PDE2A/ADCY2/ADCY9/PDE8A/ADCY7/PDE3B/PDE1B/ADORA1/ADCY8/GABRB1/PDE1C/GABRG2/GNAI3/GNAS	20	3.158366456
hsa04727	GABAergic synapse	19/564	89/8105	8.56E-06	0.00135	0.001158	PRKACA/SLC38A1/GNG4/GNG7/GABRA1/GABRA5/GABRB3/SLC6A1/ADCY2/ADCY9/GLUL/ADCY7/ADCY8/GABRB1/GLS2/SLC12A5/GABRG2/GAD1/GNAI3	19	3.067873934
hsa05414	Dilated cardiomyopathy	17/564	96/8105	0.0002926	0.03082	0.026389	ITGA9/CACNA2D4/PRKACA/ATP2A2/ADCY2/ADCY9/LAMA1/SGCA/ADCY7/ITGA2B/DES/CACNA2D3/TPM1/LMNA/ADCY8/SLC8A3/GNAS	17	2.544787973
hsa04261	Adrenergic signaling in cardiomyocytes	22/564	150/8105	0.0006539	0.04361	0.037335	ATP2B2/CACNA2D4/PRKACA/ATP2A2/PIK3CG/PPP2R2A/CAMK2A/CREB5/CREB3L4/ADCY2/ADCY9/ADCY7/CACNA2D3/TPM1/ADCY8/SCN1B/CAMK2B/SLC8A3/PPP2R2B/PPP2R5B/GNAI3/GNAS	22	2.107683215
hsa00410	beta-Alanine metabolism	8/564	30/8105	0.0007774	0.04361	0.037335	HIBCH/ALDH3A1/ALDH1B1/ALDH3A2/EHHADH/GADL1/GAD1/ALDH7A1	8	3.8321513
hsa04724	Glutamatergic synapse	18/564	114/8105	0.0008281	0.04361	0.037335	GRIK3/PRKACA/SLC38A1/GNG4/GNG7/SLC1A7/GRIN2C/ADCY2/ADCY9/GLUL/GRM4/ADCY7/GRIA2/SLC1A1/ADCY8/GLS2/GNAI3/GNAS	18	2.269036954
hsa04713	Circadian entrainment	16/564	97/8105	0.0009817	0.04432	0.037941	PRKACA/GNG4/GNG7/CACNA1H/GRIN2C/CAMK2A/ADCY2/ADCY9/ADCY7/NOS1AP/GRIA2/ADCY8/CAMK2B/RYR1/GNAI3/GNAS	16	2.370402866
hsa04020	Calcium signaling pathway	30/564	240/8105	0.0011929	0.04712	0.040338	PDGFRA/ATP2B2/PRKACA/ATP2A2/PLCD1/CACNA1H/GDNF/FGFR2/GRIN2C/CAMK2A/F2R/HGF/ADCY2/CACNA1E/ADCY9/ADCY7/P2RX1/MYLK/LTB4R2/CCKBR/PDE1B/NTRK2/HTR6/ADCY8/PDE1C/CAMK2B/SLC8A3/RYR1/SLC25A31/GNAS	30	1.796320922

Supplemental Table 13 - Expression Quantitative Trait Methylation Analysis (cis-eQTM)

	DMP ID	Probe ID	t-value	*P*-value	FDR	Beta	DMP_chromosom	DMP_position	DMP_strand	DMP UCSC RefGene name	Probe annotation	Probe chromosom	Probe start	Probe end
**Total discovery set (N=48)**	**cg23432008**	**ILMN_1763837**	**4.011366462**	**0.000219916**	**0.016053858**	**3.045970198**	**chr15**	**90346094**	**-**	***ANPEP***	***ANPEP***	**chr15**	**90328125**	**90358083**
	**cg02008229**	**ILMN_1763837**	**3.538783851**	**0.000931759**	**0.034009196**	**2.298813443**	**chr15**	**90346089**	**-**	***ANPEP***	***ANPEP***	**chr15**	**90328125**	**90358083**
	cg15677293	ILMN_1761820	-2.723482443	0.009098786	0.166052839	-1.32954999	chr1	236559595	-	*EDARADD*	*EDARADD*	chr1	236557679	236648007
	cg06344992	ILMN_1763837	2.781392138	0.007820373	0.166052839	1.618370612	chr15	90345999	-	*ANPEP*	*ANPEP*	chr15	90328125	90358083
	cg05500574	ILMN_1700766	2.539852299	0.014532508	0.212174618	2.862360519	chr19	58962884	+	*ZNF324B*	*ZNF324B*	chr19	58962970	58969199
	cg05529343	ILMN_1710268	-2.46303655	0.017579474	0.213883595	-0.411070474	chr3	22412124	+	*NA*	*ZNF385D*	chr3	21459910	22414131
	cg09981964	ILMN_1690223	2.141502188	0.037563297	0.3865465	1.958751849	chr7	148001779	-	*CNTNAP2;MIR548T*	*CNTNAP2*	chr7	145813298	148118089
	cg13976502	ILMN_2412922	2.088016709	0.04236126	0.3865465	0.968230859	chr14	74227875	+	*C14orf43*	*ELMSAN1*	chr14	74181824	74256987

	DMP ID	Probe ID	t-value	*P*-value	FDR	Beta	DMP_chromosom	DMP_position	DMP_strand	DMP UCSC RefGene name	Probe annotation	Probe chromosom	Probe start	Probe end
**Healthy subgroup (N=25)**	cg09981964	ILMN_1690223	2.619851078	0.01531306	0.489471819	3.145442891	chr7	148001779	-	*CNTNAP2;MIR548T*	*CNTNAP2*	chr7	145813298	148118089
	cg19757435	ILMN_1778121	-2.497260403	0.02011528	0.489471819	-2.258203853	chr7	71868412	+	*CALN1*	*CALN1*	chr7	71244475	71969255
	cg23771366	ILMN_1797776	-2.638485037	0.014684931	0.489471819	-4.058361065	chr11	86510998	-	*PRSS23*	*PRSS23*	chr11	86502100	86663951
	cg03636183	ILMN_2127298	2.177470156	0.039957348	0.729221606	0.883490511	chr19	17000585	-	*F2RL3*	*F2RL3*	chr19	16999670	17003410

	DMP ID	Probe ID	t-value	*P*-value	FDR	Beta	DMP_chromosom	DMP_position	DMP_strand	DMP UCSC RefGene name	Probe annotation	Probe chromosom	Probe start	Probe end
**Unhealthy subgroup (N=23)**	cg16436566	ILMN_1655046	2.633294959	0.015538127	0.338539797	3.624340089	chr16	67879671	+	*CENPT;NUTF2*	*NUTF2*	chr16	67880634	67906469
	cg05500574	ILMN_1700766	2.240017298	0.03603567	0.338539797	3.746437312	chr19	58962884	+	*ZNF324B*	*ZNF324B*	chr19	58962970	58969199
	cg07421287	ILMN_1702604	-2.58906758	0.017120763	0.338539797	-0.555528047	chr1	111218287	+	*KCNA3*	*KCNA3*	chr1	111196181	111217654
	cg05529343	ILMN_1710268	-2.225951704	0.037100252	0.338539797	-0.702050446	chr3	22412124	+	*NA*	*ZNF385D*	chr3	21459910	22414131
	cg00310412	ILMN_1756312	-2.253220952	0.035061806	0.338539797	-1.068893382	chr15	74724918	-	*SEMA7A*	*SEMA7A*	chr15	74701624	74726299
	cg15677293	ILMN_1761820	-2.288662004	0.032565701	0.338539797	-2.16095061	chr1	236559595	-	*EDARADD*	*EDARADD*	chr1	236557679	236648007
	cg25817435	ILMN_1765696	2.307248943	0.031322836	0.338539797	1.353367121	chr2	223435533	+	*NA*	*FARSB*	chr2	223436161	223521073
	cg00443596	ILMN_2087629	-2.531491732	0.019408678	0.338539797	-2.122845202	chr18	31802530	-	*NOL4*	*NOL4*	chr18	31431063	31804920
